# Insights into intraspecific variation and genotyping of *Ganoderma lingzhi* through pan-mitogenome analysis

**DOI:** 10.3897/imafungus.17.184941

**Published:** 2026-06-03

**Authors:** Jingling Li, Chang Zhang, Yang Ni, Lei Sun, Xianhao Cheng, Chang Liu

**Affiliations:** 1 State Key Laboratory for Quality Ensurance and Sustainable Use of Dao-di Herbs, Institute of Medicinal Plant Development, Chinese Academy of Medical Sciences & Peking Union Medical College, Beijing 100093, China School of Agriculture, Ludong University Yantai China; 2 Shandong Key Laboratory of Edible Mushroom Technology, School of Agriculture, Ludong University, Yantai 264025, China Institute of Medicinal Plant Development, Chinese Academy of Medical Sciences & Peking Union Medical College Beijing China

**Keywords:** *
Ganoderma
*, *Ganoderma lingzhi*, Intron variation, Pan-mitogenome, Strain identification

## Abstract

*Ganoderma lingzhi* is a medicinal fungus characterized by its large fruiting bodies. In this study, we collected 151 cultivated *Ganoderma* strains from across China and performed whole-genome resequencing. By integrating 30 publicly available *G. lingzhi* datasets, we successfully assembled a total of 181 complete *Ganoderma* mitochondrial genomes. We conducted a systematic analysis of their genomic features, intron distribution, gene order, non-synonymous/synonymous substitution rates (Ka/Ks), and phylogenetic relationships. Our results revealed that among the 151 strains we collected, 19 exhibited discordances between genetic identity and labeled names, highlighting the prevalent issue of strain misidentification in the current commercial market of *G. lingzhi*. The size of *G. lingzhi* mitogenomes ranged from 49,233 to 70,498 bp. We identified 20 distinct introns whose presence/absence was highly dynamic across the *G. lingzhi* samples. Based on intron distribution patterns, the samples were classified into two major groups. Comparative genomics revealed a conserved gene order across the genus, and Ka/Ks analysis indicated that the 15 core protein-coding genes were under purifying selection compared to neutral expectations. Phylogenetic analysis based on the mitogenome confirmed the monophyly of *G. lingzhi*. This study presents the first large-scale pan-mitogenomic analysis of *G. lingzhi*, revealing that intron dynamics are the primary driver of intraspecific genomic variation and differentiation. These results can be used for the precise identification, traceability, and breeding of *G. lingzhi* strains.

## Introduction

*Ganoderma lingzhi* is a macro-fungus belonging to the *Basidiomycota*. Based on the revised familial-level phylogeny of *Polyporales* (Justo et al. 2017), the genus *Ganoderma* is placed within *Polyporaceae*. This species has been used medicinally and as food in China for over two thousand years (Cao et al. 2012; Ma et al. 2018b), traditionally regarded as a superior tonic. Historically, it has been known as “Ruizhi,” “Shenzhi,” and “Xiancao,” with the meaning of good fortune and mysterious power, and was traditionally believed to enhance physical constitution and promote longevity (Paterson 2006; Lin 2019). The ancient pharmaceutical text *Shen Nong Ben Cao Jing* (Divine Farmer’s Materia Medica) from the Eastern Han Dynasty classified *G. lingzhi* as a superior drug, recording its properties for “benefiting the heart energy,” “calming the essential spirit,” and enabling one to “feel lightened, resist aging, and prolong life” with long-term use (Lin 2019). For a long time, the *G. lingzhi* used in East Asia was widely classified as the European species *G. lucidum*. However, molecular phylogenetic studies have confirmed that the *G. lingzhi* widely cultivated and used in East Asia is actually a distinct species, which was formally named *G. lingzhi* in 2012 (Cao et al. 2012). Therefore, in this study, we consistently use *G. lingzhi* to describe the Lingzhi mushroom found in East Asia.

Modern research has revealed that the medicinal value of *G. lingzhi* is primarily attributed to its diverse bioactive compounds, chiefly polysaccharides (Ma et al. 2018a) and triterpenoids (such as ganoderic acids) (Yang and Yang 2019; Zhao et al. 2023; Baba et al. 2024), alongside nucleotides, sterols, and alkaloids (Wei-Ye et al. 2024). Pharmacological studies have demonstrated that these compounds contribute to preventive and therapeutic effects against conditions including hypertension, hepatitis, and diabetes (Tran et al. 2014; Amen et al. 2017; Meng and Yang 2019). In China, *G. lingzhi* is utilized not only as a traditional Chinese medicine but also as a common raw material for nutritional and industrial applications (Loyd et al. 2018; Li et al. 2019; Fraile-Fabero et al. 2021). Particularly significant recently is its pilot inclusion as a substance recognized for both medicinal and food uses in China, which is expected to substantially boost the development of the related industry.

Currently, the *G. lingzhi* industry faces challenges related to considerable confusion in the application of strains during production (He et al. 2022). Prevalent issues include inconsistent strain nomenclature and misidentification, leading to instability in the quality and yield of cultivated *G. lingzhi*. Although the identification based on Internal Transcribed Spacer (ITS) sequences demonstrates strong discriminatory power at the interspecific level, for instance, in distinguishing *G. lingzhi* from *G. sinense* using the ITS2 sequence (Liao et al. 2015), its identification efficiency decreases significantly at the intraspecific level. Furthermore, the commercial names of some cultivated strains do not correspond to their actual taxonomic species names, which not only affects consumer experience but also poses risks to the sustainable development of the industry.

Mitochondria are crucial organelles in fungal cells, intimately involved in cellular energy metabolism (Ždralević et al. 2024). They contain their own genetic material, the mitochondrial genome (mitogenome), which is distinct from the nuclear DNA. Fungal mitogenomes are typically circular, double-stranded DNA molecules, whose size varies considerably among species (Paquin et al. 1997; Sandor et al. 2018). They encode a relatively conserved set of core genes, usually including 14 protein-coding genes (PCGs) involved in energy metabolism (e.g., cytochrome c oxidase subunits *cox1*–*cox3*; NADH dehydrogenase subunits *nad1*–*nad6*, etc.), two ribosomal RNA genes (*rnL* and *rnS*), and a set of tRNA genes. Although these core genes are themselves conserved, major differences in mitogenomes among species arise from variations in gene order, the number and distribution of introns, and the content of repetitive sequences (Li et al. 2021; Chang et al. 2024; Wang et al. 2025a). The fungal mitogenome is inherited and evolves independently of the nuclear genome. Its characteristics hold significant value for phylogenetic studies, species identification, and population genetics (Zhang et al. 2023; Feng et al. 2024). For economically and medicinally important macro-fungi like *Ganoderma*, the mitogenome provides a unique molecular perspective for clarifying complex interspecific relationships.

Within the genus *Ganoderma*, the mitogenome shows great potential for interspecies identification. Previous studies indicate that *Ganoderma* mitogenome can range in size from approximately 60 kb to over 100 kb (Wu et al. 2023). Among the features contributing to diversity, introns, particularly Group I introns (Paukstelis and Lambowitz 2008), are a key factor driving genome size variation and generating species-specific profiles. For instance, the number of introns within the *cox1* gene varies significantly among species, and these introns often encode homing endonucleases such as LAGLIDADG or GIY-YIG (Ogawa et al. 1997; Schäfer and Wolf 1999). Furthermore, tRNA gene composition, insertion/deletion events, and the content of repetitive sequences in intergenic regions provide rich sources of discriminatory information (Liu et al. 2020). Using the mitogenome as a DNA barcode or super-barcode for *Ganoderma* species identification offers distinct advantages over traditional morphological methods or single nuclear gene markers, like the ITS region (Gunnels et al. 2020). The mitogenome provides a greater amount of genetic information, has a clear evolutionary history, and, being generally haploid and not undergoing recombination, can help resolve the identification of closely related species and cryptic species (Kortsinoglou et al. 2020; Sato et al. 2020). With the increasing accessibility and decreasing cost of sequencing technologies, obtaining complete mitogenome sequences is becoming more feasible. Integrating mitogenome data with nuclear gene markers (e.g., ITS) and other biological information for multi-omics integrated analysis represents a crucial future direction for achieving accurate species identification and elucidating phylogenetic relationships within the genus *Ganoderma*.

In this study, we collected 151 cultivated *Ganoderma* strains from across China, and most of them are claimed to be the *G. lingzhi* species. Using whole-genome resequencing (WGS) technology, we successfully assembled their complete mitogenome sequences. By combining these data with publicly available WGS data (30 sets), we conducted a comparative analysis of the mitogenome characteristics of *G. lingzhi* and investigated genomic variations at the intraspecific level. Our findings provide theoretical insights for the taxonomic identification of *G. lingzhi* strains and offer guidance for future breeding efforts in this species.

## Materials and methods

### Abbreviations

**ML** Maximum Likelihood method

**nrRNAs** nuclear ribosomal RNA sequences

**ITS** Internal Transcribed Spacer

***rnL*** ribosomal large subunit

***rnS*** ribosomal small subunit

**PCGs** Protein-Coding Genes

**Ka** Non-synonymous substitution rates

**Ks** Synonymous substitution rates

**tRNA** transfer ribonucleic acid

**rRNA** ribosomal ribonucleic acid

**NCBI** National Center for Biotechnology Information

**WGS** Whole Genome Sequencing

**BIC** Bayesian Information Criterion

**GFA** Graphical Fragment Assembly

**SNP** Single Nucleotide Polymorphisms

**ctg** contig

**SRA** Sequence Read Archive

### Collection of whole genome sequencing data for *Ganoderma* samples

*Ganoderma* samples and resequencing data were obtained from our previously published study (Sun et al. 2024). Additionally, we downloaded available whole genome sequencing (WGS) data for *G. lingzhi* from public NCBI databases, totaling 30 datasets designated as P01–P30. Specific SRA (Sequence Read Archive) accession numbers are provided in Suppl. material 1: table SS1. The links and references for these data can be found in Table 1. Ultimately, we compiled a dataset comprising 181 WGS datasets.

**Table 1.** Sources and reference list of public data used in this study.

Organism	Public database	Accession number	Data accession link	Reference
*Ganoderma sinense*	GenBank	OP453745	https://www.ncbi.nlm.nih.gov/nuccore/OP453745	Wang Z (unpublished)
*Ganoderma sinense*	GenBank	PQ301463	https://www.ncbi.nlm.nih.gov/nuccore/PQ301463	(Chen et al. 2024)
*Ganoderma sinense*	GenBank	NC_022933.1	https://www.ncbi.nlm.nih.gov/nuccore/NC_022933	Qian J (unpublished)
*Ganoderma sinense*	GenBank	PV230469	https://www.ncbi.nlm.nih.gov/nuccore/PV230469	Meng C (unpublished)
*Ganoderma subamboinense*	GenBank	NC_060422.1	https://www.ncbi.nlm.nih.gov/nuccore/NC_060422	(Li et al. 2022)
*Ganoderma resinaceum*	GenBank	PP780022	https://www.ncbi.nlm.nih.gov/nuccore/PP780022	(He and Chen. 2024)
*Ganoderma weberianum*	GenBank	NC_085596.1	https://www.ncbi.nlm.nih.gov/nuccore/NC_085596	Liang L (unpublished)
*Ganoderma tsugae*	GenBank	NC_037936.1	https://www.ncbi.nlm.nih.gov/nuccore/NC_037936	(Li et al. 2019)
*Ganoderma leucocontextum*	GenBank	PP212909	https://www.ncbi.nlm.nih.gov/nuccore/PP212909	Tang F (unpublished)
*Ganoderma leucocontextum*	GenBank	NC_037937.1	https://www.ncbi.nlm.nih.gov/nuccore/NC_037937	(Li et al. 2019)
*Ganoderma leucocontextum*	GenBank	PP790945	https://www.ncbi.nlm.nih.gov/nuccore/PP790945	(Chen et al. 2024)
*Ganoderma applanatum*	GenBank	NC_027188.1	https://www.ncbi.nlm.nih.gov/nuccore/NC_027188	(Wang et al. 2016a)
*Ganoderma calidophilum*	GenBank	NC_037938.1	https://www.ncbi.nlm.nih.gov/nuccore/NC_037938	(Li et al. 2019)
*Ganoderma meredithae*	GenBank	NC_026782.1	https://www.ncbi.nlm.nih.gov/nuccore/NC_026782	(Wang et al. 2016b)
*Ganoderma multipileum*	GenBank	NC_061293.1	https://www.ncbi.nlm.nih.gov/nuccore/NC_061293	(Meng et al. 2020)
*Ganoderma pseudoferreum*	GenBank	PP778499	https://www.ncbi.nlm.nih.gov/nuccore/PP778499	(Lu et al. 2025)
*Ganoderma flexipes*	GenBank	NC_068513.1	https://www.ncbi.nlm.nih.gov/nuccore/NC_068513	Meng G (unpublished)
*Ganoderma shanxiense*	GenBank	PV867420	https://www.ncbi.nlm.nih.gov/nuccore/PV867420	(Wang et al. 2026)
*Perenniporia subacida*	GenBank	NC_086786.1	https://www.ncbi.nlm.nih.gov/nuccore/NC_086786	Tang F (unpublished)
*Trametes betulina*	GenBank	NC_086787.1	https://www.ncbi.nlm.nih.gov/nuccore/NC_086787	Tang F (unpublished)
*Ganoderma lingzhi*	SRA	DRR087045	https://www.ncbi.nlm.nih.gov/sra/?term=DRR087045	Unpublished
*Ganoderma lingzhi*	SRA	DRR087046	https://www.ncbi.nlm.nih.gov/sra/?term=DRR087046	Unpublished
*Ganoderma lingzhi*	SRA	SRR15329772	https://www.ncbi.nlm.nih.gov/sra/?term=SRR15329772	(Jiang et al. 2021)
*Ganoderma lingzhi*	SRA	SRR15329773	https://www.ncbi.nlm.nih.gov/sra/?term=SRR15329773	(Jiang et al. 2021)
*Ganoderma lingzhi*	SRA	SRR15329774	https://www.ncbi.nlm.nih.gov/sra/?term=SRR15329774	(Jiang et al. 2021)
*Ganoderma lingzhi*	SRA	SRR15329775	https://www.ncbi.nlm.nih.gov/sra/?term=SRR15329775	(Jiang et al. 2021)
*Ganoderma lingzhi*	SRA	SRR15329776	https://www.ncbi.nlm.nih.gov/sra/?term=SRR15329776	(Jiang et al. 2021)
*Ganoderma lingzhi*	SRA	SRR15329777	https://www.ncbi.nlm.nih.gov/sra/?term=SRR15329777	(Jiang et al. 2021)
*Ganoderma lingzhi*	SRA	SRR15329778	https://www.ncbi.nlm.nih.gov/sra/?term=SRR15329778	(Jiang et al. 2021)
*Ganoderma lingzhi*	SRA	SRR15329779	https://www.ncbi.nlm.nih.gov/sra/?term=SRR15329779	(Jiang et al. 2021)
*Ganoderma lingzhi*	SRA	SRR15329780	https://www.ncbi.nlm.nih.gov/sra/?term=SRR15329780	(Jiang et al. 2021)
*Ganoderma lingzhi*	SRA	SRR15329781	https://www.ncbi.nlm.nih.gov/sra/?term=SRR15329781	(Jiang et al. 2021)
*Ganoderma lingzhi*	SRA	SRR15329783	https://www.ncbi.nlm.nih.gov/sra/?term=SRR15329783	(Jiang et al. 2021)
*Ganoderma lingzhi*	SRA	SRR15329784	https://www.ncbi.nlm.nih.gov/sra/?term=SRR15329784	(Jiang et al. 2021)
*Ganoderma lingzhi*	SRA	SRR15329785	https://www.ncbi.nlm.nih.gov/sra/?term=SRR15329785	(Jiang et al. 2021)
*Ganoderma lingzhi*	SRA	SRR15329786	https://www.ncbi.nlm.nih.gov/sra/?term=SRR15329786	(Jiang et al. 2021)
*Ganoderma lingzhi*	SRA	SRR15329787	https://www.ncbi.nlm.nih.gov/sra/?term=SRR15329787	(Jiang et al. 2021)
*Ganoderma lingzhi*	SRA	SRR15329788	https://www.ncbi.nlm.nih.gov/sra/?term=SRR15329788	(Jiang et al. 2021)
*Ganoderma lingzhi*	SRA	SRR15329789	https://www.ncbi.nlm.nih.gov/sra/?term=SRR15329789	(Jiang et al. 2021)
*Ganoderma lingzhi*	SRA	SRR15329790	https://www.ncbi.nlm.nih.gov/sra/?term=SRR15329790	(Jiang et al. 2021)
*Ganoderma lingzhi*	SRA	SRR16605591	https://www.ncbi.nlm.nih.gov/sra/?term=SRR16605591	(Sun et al. 2022)
*Ganoderma lingzhi*	SRA	SRR17595711	https://www.ncbi.nlm.nih.gov/sra/?term=SRR17595711	(Yuan et al. 2022)
*Ganoderma lingzhi*	SRA	SRR19350612	https://www.ncbi.nlm.nih.gov/sra/?term=SRR19350612	(Jiang et al. 2025)
*Ganoderma lingzhi*	SRA	SRR22226939	https://www.ncbi.nlm.nih.gov/sra/?term=SRR22226939	(Wu et al. 2022)
*Ganoderma lingzhi*	SRA	SRR25208127	https://www.ncbi.nlm.nih.gov/sra/?term=SRR25208127	(Yu et al. 2024)
*Ganoderma lingzhi*	SRA	SRR25208174	https://www.ncbi.nlm.nih.gov/sra/?term=SRR25208174	(Yu et al. 2024)
*Ganoderma lingzhi*	SRA	SRR25208175	https://www.ncbi.nlm.nih.gov/sra/?term=SRR25208175	(Yu et al. 2024)
*Ganoderma lingzhi*	SRA	SRR25208176	https://www.ncbi.nlm.nih.gov/sra/?term=SRR25208176	(Yu et al. 2024)
*Ganoderma lingzhi*	SRA	SRR25299243	https://www.ncbi.nlm.nih.gov/sra/?term=SRR25299243	Unpublished
*Ganoderma lingzhi*	SRA	SRR25605478	https://www.ncbi.nlm.nih.gov/sra/?term=SRR25605478	Unpublished

Note. SRA: Sequence Read Archive.

### Assembly of nuclear ribosomal RNA and preliminary species identification

For each WGS dataset, we used GetOrganelle (v1.7.7.0) (Jin et al. 2020) software to assemble nuclear ribosomal RNA sequences (nrRNAs). The command used was: get_organelle_from_reads.py -1 forward.fq -2 reverse.fq -R 10 -k 21,45,65,85,105 -F fungus_nr -o fungus_nr_out. All datasets yielded complete or partial nrRNAs containing the 18S-ITS1-5.8S-ITS2-28S fragment, with ITS1 and ITS2 regions commonly used as barcodes for species identification. For the obtained nrRNAs, we performed sequence alignment using MAFFT (v7.525) (Katoh and Standley 2013) and conducted phylogenetic analysis using IQTREE2 (v2.2.5) (Minh et al. 2020) with the parameters: —alrt 1000 -B 1000, combining ModelFinder (Kalyaanamoorthy et al. 2017), tree search, ultrafast bootstrap, and SH-aLRT test. Maximum likelihood trees were constructed, with the best-fit model selected based on Bayesian Information Criterion (BIC) being TIM+F+I+G4.

### Mitogenome assembly and annotation

For each WGS dataset, mitogenome assembly was performed using GetOrganelle with the command: get_organelle_from_reads.py -1 forward.fq -2 reverse.fq -R 10 -k 21,45,65,85,105 -F fungus_mt -o fungus_mt_out. Among the 181 assembled datasets, 176 successfully yielded circular sequences (Suppl. material 1: table SS1). Due to repetitive sequences in some samples, GetOrganelle generated multiple circular configurations: 132 datasets produced two different configurations, one dataset produced three configurations, and six datasets produced six configurations. However, 36 datasets yielded unique circular mitogenome configurations. Through pairwise comparisons, we confirmed that these 36 datasets shared consistent genome configurations. The remaining datasets with multiple configurations were standardized to match these 36 reference configurations. Five datasets failed to produce circular genomes. Manual inspection revealed that while SPAdes (v3.15.5) (Bankevich et al. 2012) successfully generated assembly results, the subsequent simple graph script failed to resolve repetitive sequences. We manually processed SPAdes results, using completed mitogenomes as references to resolve repetitive sequences. Specifically, L48, L75, and Z40 (*G. oregonense*) were referenced against L11, while L75 and P30 (*G. lingzhi*) were referenced against L01. Ultimately, we successfully obtained mitogenome data representing all 181 samples. The start point of the mitogenome was standardized based on previously published data (e.g., OR286997.1, MT765267.1–MT765268.1, MT843208.1–MT843222.1, MT765269.1–MT765270.1), with the *rnL* gene uniformly set as the first gene of the genome and located on the positive strand.

We employed two annotation tools for mitogenome annotation: Mfannot (Lang et al. 2023) and MITOS2 (Bernt et al. 2013). Mfannot used genetic code table “4: Mold, Protozoan, and Coelenterate Mitochondrial; Mycoplasma/Spiroplasma,” while MITOS2 used the “refseq89 Fungi” database with genetic code table “Mold, Protozoan, Coelenterate (4).” We integrated results from both tools, as Mfannot lacked rRNA gene and *rps3* annotations in most samples, which were well-annotated by MITOS2. Manual integration yielded complete annotations. Based on the five-column tabular annotation files (.tbl), we submitted data to NCBI’s BankIt platform, generating GenBank format annotation files for each sample’s mitogenome. Genome maps were generated using OGDRAW (v1.3.1) (Greiner et al. 2019).

In this study, the nomenclature for introns in mitochondrial protein-coding genes follows the standard system proposed previously (Zhang and Zhang 2019). The core rule is that each intron name consists of three parts in sequence: the host gene name, a letter indicating the type (“P” for group I, “S” for group II, and “U” for introns of unknown type), and the intron insertion site. The determination of the insertion site is the key step. It is defined by aligning the target gene sequence with the corresponding gene from an intron-free reference genome, namely *Tolypocladium inflatum* (accession NC_036382.1), and identifying the specific nucleotide position in that reference sequence where the intron is inserted.

### Analysis of mitochondrial gene arrangement in *Ganoderma* species

For each species’ mitogenome, we extracted protein-coding gene positions and orientations from GenBank files and visualized them using gggenes (https://github.com/wilkox/gggenes). Since mitogenomes are circular and starting points vary among species, we standardized gene arrangements using *G. lingzhi* (L01) as reference, with *rnL* gene positioned first.

### Analysis of repetitive sequences in *Ganoderma* species

We identified repetitive sequences in the mitogenomic sequence using the ROUSFinder software (Wynn and Christensen 2019), with the minimum repeat unit length set to 50 and the remaining parameters at their default values.

### Analysis of mitochondrial intron variation in *G. lingzhi*

To investigate intraspecific mitochondrial intron variation in *G. lingzhi*, we extracted all mitochondrial intron sequences using custom scripts. Introns were numbered and classified based on sequence variation and positional differences within coding regions. We created GFA format files by manually defining intronic and non-intronic regions, visualized using Bandage software. Intron distribution heatmaps were generated using TBtools-II (Chen et al. 2023). Based on intron distribution patterns, we classified *G. lingzhi* mitogenomes. Specifically, we compiled a binary matrix with samples as columns and introns as rows, where “+” indicates presence and “-” indicates absence. Each unique combination of “+” and “-” patterns was defined as a distinct mitochondrial haplotype.

### Identification of intraspecific single nucleotide polymorphisms (SNPs) in the Mitochondrial coding regions of *G. lingzhi*

We used the L01 strain of *G. lingzhi* as the reference, and employed Snippy (v4.6.0) (https://github.com/tseemann/snippy) to compare the sequence variations between other strains and L01, specifically calculating the SNPs located within the coding regions. This is mainly due to the fact that the non-coding regions of *G. lingzhi* are not conserved, and the presence/absence of introns is highly dynamic. The command used for each pairwise comparison was: snippy —cpus 16 —ref L01.gb —ctgs test.fasta. Here, L01.gb is the GenBank annotation file of our reference strain L01, and test.fasta represents the complete mitogenome sequence of another strain being compared to L01. For each of the other strains, this command was run separately to generate independent SNP calling results for each pairwise comparison. Lastly, we used snippy-core to merge the result files generated from all the individual Snippy runs.

### Ka/Ks analysis of mitochondrial coding genes in *Ganoderma* species

Using PhyloSuite (Zhang et al. 2020), we extracted annotated genes from GenBank files, identifying 15 conserved mitochondrial protein-coding genes (PCGs): *atp6*, *atp8*, *atp9*, *cob*, *cox1*, *cox2*, *cox3*, *nad1*, *nad2*, *nad3*, *nad4*, *nad4L*, *nad5*, *nad6*, and *rps3*. Coding sequences were aligned using MAFFT (v7.525), and synonymous/nonsynonymous substitution rates (Ka and Ks) with their ratios (Ka/Ks) were calculated using KaKs_Calculator (v3.0) (Zhang 2022).

### Phylogenetic analysis

We downloaded available mitogenomes of other *Ganoderma* species from NCBI (Suppl. material 1: table SS2), using *Perenniporia subacida* (NC_086786.1) and *Trametes betulina* (NC_086787.1) as outgroups. The download links and references for these mitogenomes can be found in Table 1. A concatenated dataset of 15 conserved mitochondrial PCGs was aligned using MAFFT (v7.525) (Katoh and Standley 2013). Phylogenetic analysis was conducted using IQTREE2 (v2.2.5) (Minh et al. 2020) with parameters —alrt 1000 -B 1000, combining ModelFinder, tree search, ultrafast bootstrap, and SH-aLRT test. Maximum likelihood trees were constructed with best-fit model HKY+F+I+R5.

## Results

### Species identification based on nuclear ribosomal RNA

Phylogenetic analysis of nrRNAs from 181 samples confirmed 157 samples as *G. lingzhi*, while 24 samples belonged to other species (Suppl. material 2). Among the 151 strains we collected, 132 were correctly identified as expected, meaning their identity matched the claimed species. However, we noted that 19 strains had been misidentified. Specifically, 13 strains (L36, L42, L43, L45, L46, L48, L52, L58, L59, L80, Z04, Z11, and Z49) originally labeled as *G. lingzhi* did not cluster within the *G. lingzhi* clade, while 6 strains (L01, L33, L57, Z15, Z32, and Z33) previously not considered to be *G. lingzhi* were grouped within the *G. lingzhi* clade. In contrast, all 30 *G. lingzhi* strains obtained from public databases clustered within the expected *G. lingzhi* clade, consistent with their reported identity (Suppl. material 1: table SS1).

### Characteristics of *G. lingzhi* mitogenomes

**Figure 1.**
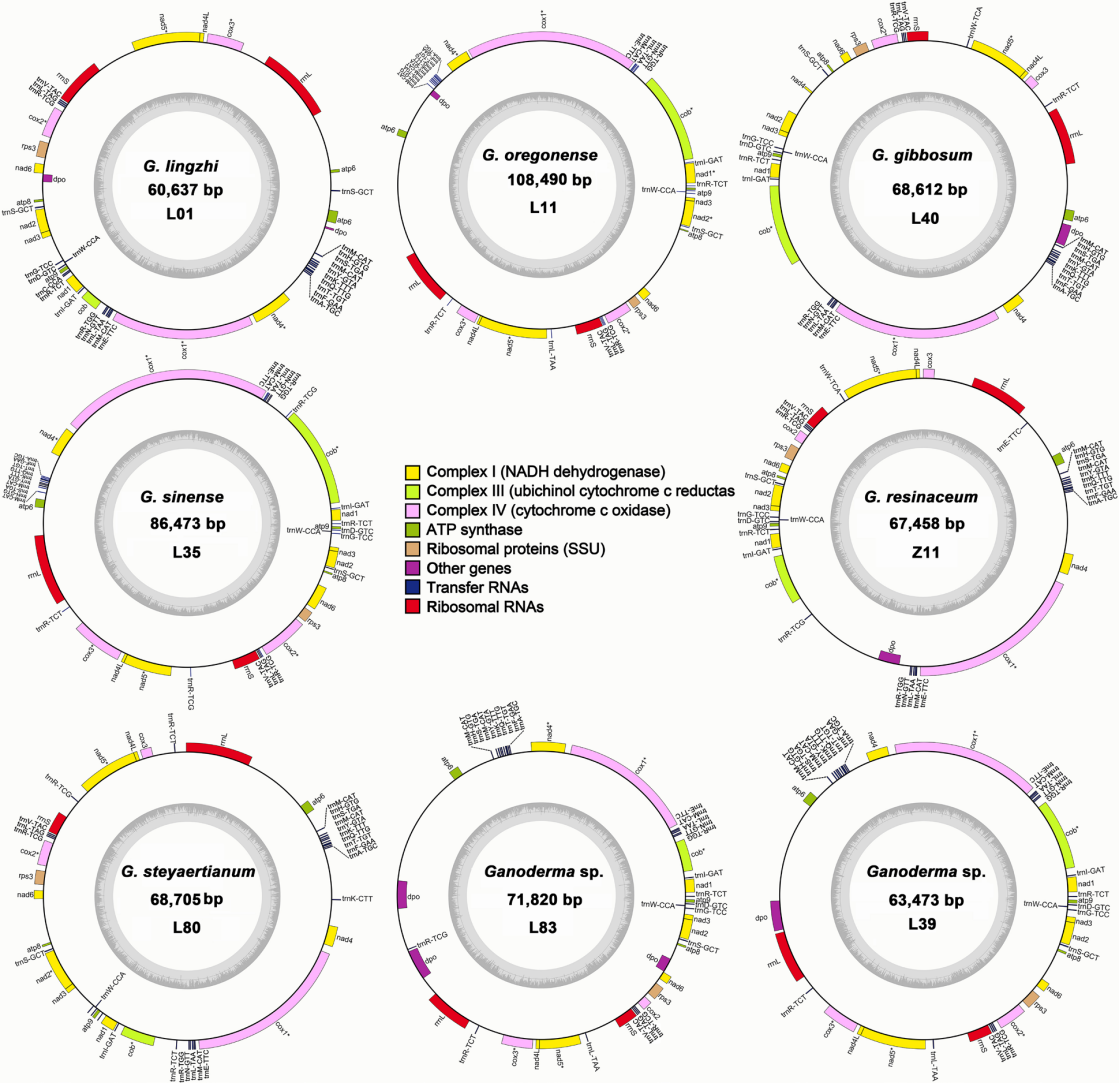
Mitogenome maps of *Ganoderma* species. The circular maps, generated by OGDRAW, display the complete mitochondrial genome architecture of each species arranged in concentric rings. Each ring corresponds to one species, as labeled in the center with its name and total genome size range in base pairs. Gene modules are plotted on both sides of the outermost ring, with colors representing distinct functional categories. The position and length of each colored block indicate the physical location and span of the corresponding gene on the genome. The inner ring shows the GC content of the mitogenome.

*G. lingzhi* mitogenomes ranged from 49,233 to 70,498 bp (average 57,671.8 bp), representing the shortest in the *Ganoderma* genus. Other sequenced species included *G. oregonense* (108,490–108,595 bp), *G. sinense* (76,882–86,473 bp), *G. gibbosum* (68,612 bp), *G. resinaceum* (67,458 bp), *G. steyaertianum* (68,705–74,661 bp), and two unidentified species (71,820 bp and 63,473 bp). Annotation revealed 15 protein-coding genes (*atp6*, *atp8*, *atp9*, *cob*, *cox1*, *cox2*, *cox3*, *nad1*, *nad2*, *nad3*, *nad4*, *nad4L*, *nad5*, *nad6*, and *rps3*), 2 rRNA genes (*rnL*, *rnS*), and 23-24 tRNA genes across all 157 *G. lingzhi* samples (Suppl. material 3). Additional open reading frames (ORFs) were primarily annotated within intronic regions, varying from 32-60 due to differences in intron content. Fig. 1 displays the mitogenome map of a representative sample from each *Ganoderma* species. Considering the intraspecific diversity within each species, we also generated maps for every individual sample, with details provided in Suppl. material 3.

### Mitochondrial intron analysis in *G. lingzhi*

**Figure 2.**
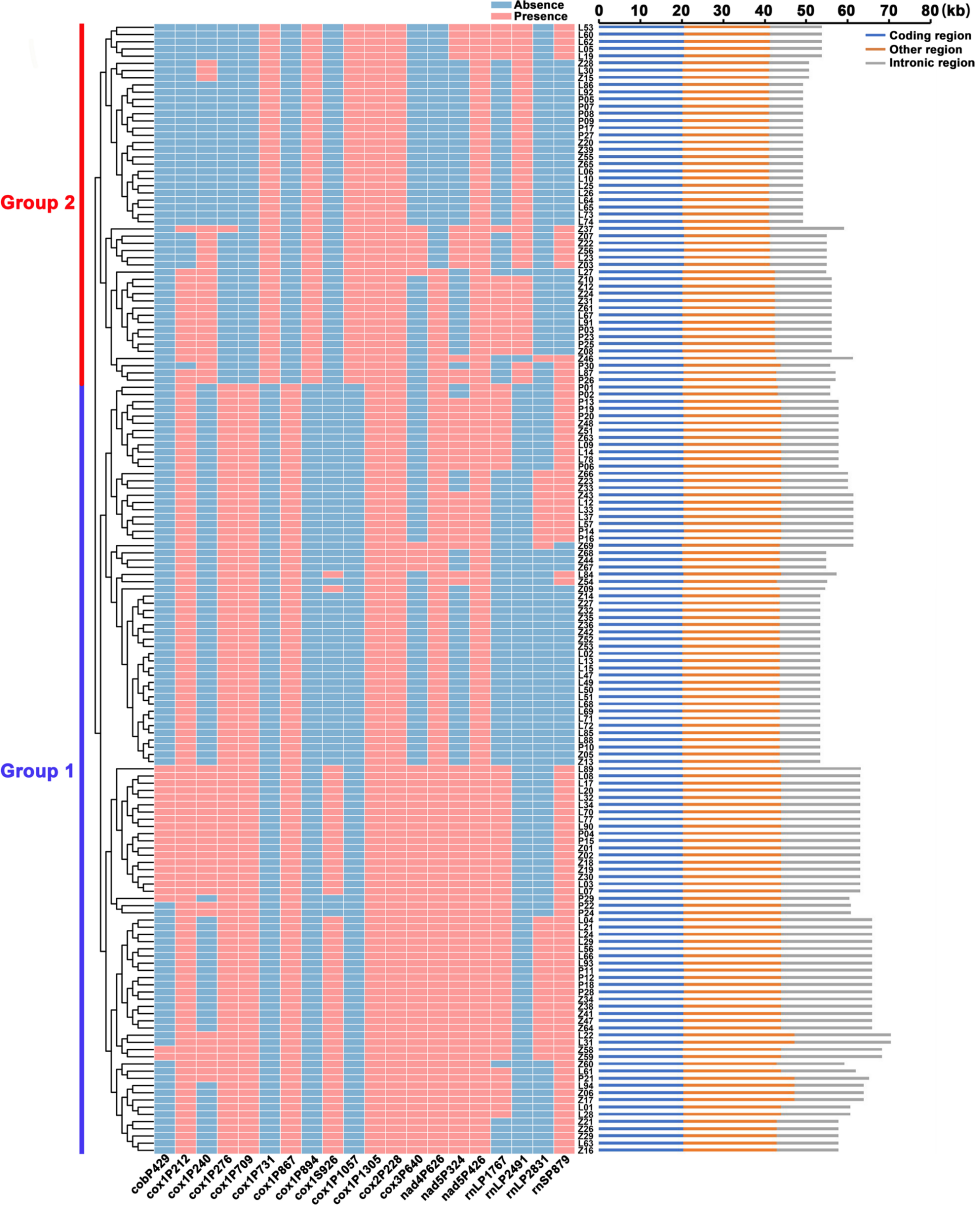
Intron distribution and genomic features in the mitogenomes of *G. lingzhi*. The heatmap on the left displays the presence or absence of introns in the mitogenomes of 157 *G. lingzhi* samples. Blue indicates the absence of an intron, while red denotes its presence. Cluster analysis based on this distribution reveals that these samples can be categorized into two major groups. The graph on the right illustrates the lengths of different regions within the mitogenomes, including the coding regions, intron regions, and other intergenic regions.

**Figure 3.**
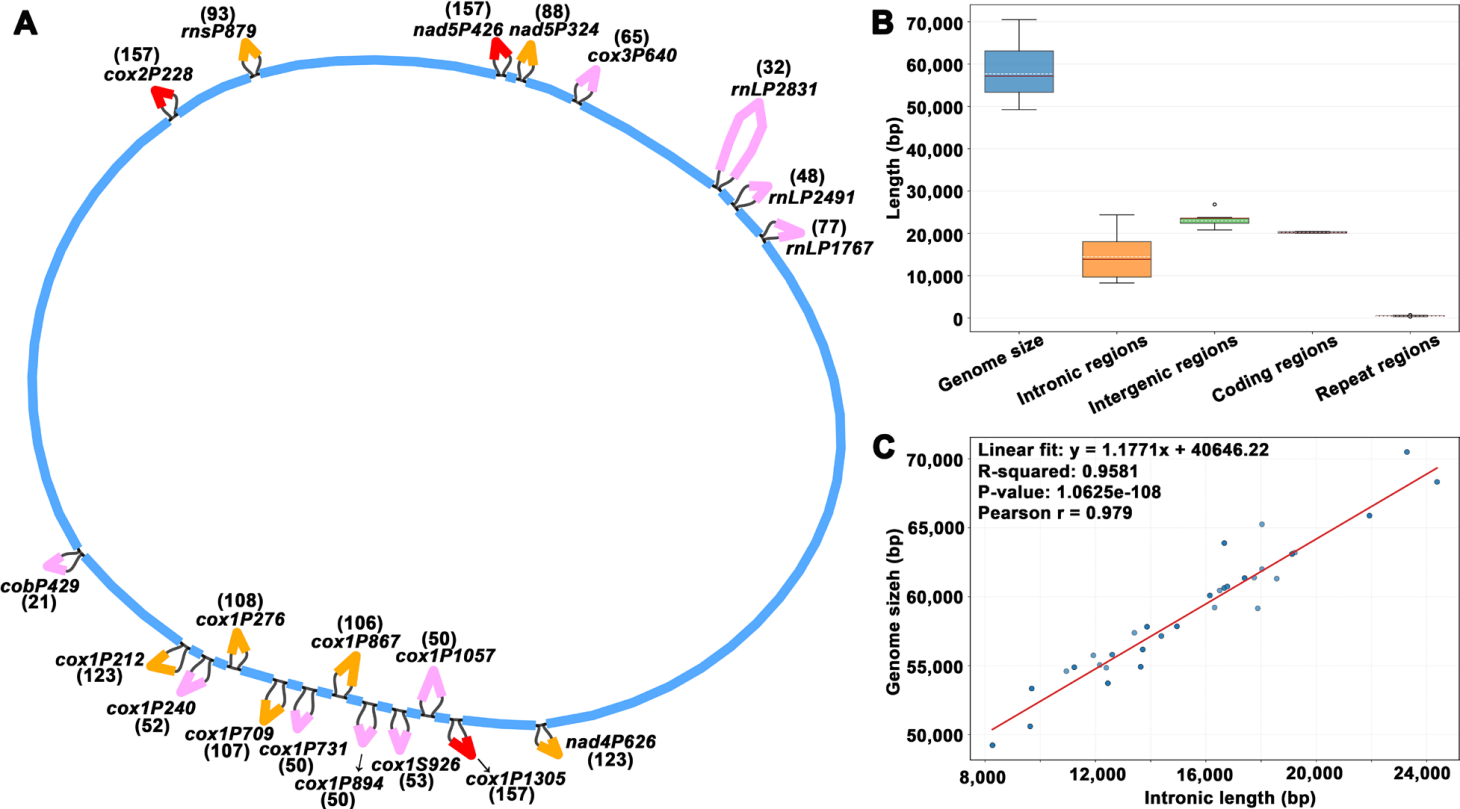
Pan-genomic landscape of intron distribution in *G. lingzhi* mitogenomes. **A**. This circular diagram visualizes the pan-genomic structure of the mitogenome across multiple *G. lingzhi* strains. The blue fragment represents the core, conserved genomic backbone sequences, which comprises the essential, single-copy genes and intergenic regions present in all individuals. Fragments with other colors are the variable, accessory regions corresponding to introns inserted at specific loci within conserved genes. The intron name is shown near the color fragment, and the number in the parentheses denotes its frequency within the population. Red fragments indicate introns present in 100% of the analyzed individuals. Orange fragments indicate introns present in > 50% of individuals. Pink fragments indicate introns present in < 50% of individuals. This pan-mitogenome diagram highlights the dynamic intron composition and defines intraspecific genomic diversity. **B**. Boxplots of various regions (genome size, intronic length, intergenic regions, coding region, and repeat length) in the mitogenomes of 157 *G. lingzhi* samples. In the figure, the white dashed lines represent the means, and the dark red solid lines represent the medians. **C**. Scatter plot and correlation analysis between the mitogenome size and mitochondrial intron length in 157 *G. lingzhi* samples. The fitted linear curve has an R^2^ value of 0.9581, indicating a strong correlation between the two variables.

We identified 20 distinct mitochondrial introns across 157 samples. Gene *cob* contained one intron (*cobP429*); *cox1* contained the most introns (10): *cox1P212*, *cox1P240*, *cox1P276*, *cox1P709*, *cox1P731*, *cox1P867*, *cox1P894*, *cox1S926*, *cox1P1057*, and *cox1P1305*. It is worth noting that *cox1S926* is the only Group II intron. Gene *cox2* contained one intron (*cox2P228*); *cox3* contained one intron (*cox3P640*); *nad4* contained one intron (*nad4P626*); *nad5* contained two introns: *nad5P324* and *nad5P426*. rRNA genes also contained introns: *rnL* had three: *rnLP1767*, *rnLP2491* and *rnLP2831*, while *rnS* had one intron (*rnSP879*). Three introns (*cox2P228*, *nad5P426*, *cox1P1305*) were highly conserved, present in all 157 samples, and considered core introns (Figs 2, 3). Seven introns (*cox1P212*, *cox1P276*, *cox1P709*, *cox1P867*, *nad4P626*, *nad5P324*, *rnLP1767*) were detected in most samples (>50%) and considered major introns (Fig. 3A). The remaining introns were detected in fewer than half of samples, with *cobP429* being the least frequent (21 samples, 13.38%) (Fig. 3A), classified as minor or low-frequency introns. The statistics on the presence/absence of introns are presented in detail in Suppl. material 1: table S3.

Among these introns, open reading frames (ORFs) were annotated. For the ORFs within the introns, we used the CD-search tool (https://www.ncbi.nlm.nih.gov/Structure/cdd/cdd.shtml) to identify their domains. As shown in Table 2, four ORFs possess GIY-YIG domains, and fifteen ORFs contain LAGLIDADG domains. Surprisingly, the *rnLP2831* intron encodes three ORFs, two of which did not have any conserved domains identified, while the remaining one was found to contain a DNA polymerase type-B family (PolB) domain, rather than a homing endonuclease domain. The lengths of these ORFs are largely conserved, exhibiting little variation among different individuals. Only a few individuals show small insertions or deletions (indels) in the ORFs, resulting in differences in the length of the coding sequences.

**Table 2.** Open reading frames identified in intronic regions and conserved domains of their encoded proteins.

Introns	ORFs	Conserved domain	Length range (bp)
*cobP429*	orf238	LAGLIDADG	717
*cox1P212*	orf330	GIY-YIG	783-993
*cox1P240*	orf345	LAGLIDADG	1,038
*cox1P276*	orf321	LAGLIDADG	966
*cox1P709*	orf282	LAGLIDADG	849
*cox1P731*	orf323	LAGLIDADG	972
*cox1P867*	orf356	LAGLIDADG	1,071-1,194
*cox1P894*	orf215	LAGLIDADG	648
*cox1S926*	orf260	LAGLIDADG	783
*cox1P1057*	orf358	GIY-YIG	1,113
*cox1P1305*	orf267	GIY-YIG	660-804
*cox2P228*	orf303	GIY-YIG	912
*cox3P640*	orf407	LAGLIDADG	1,224
*nad4P626*	orf248	LAGLIDADG	747
*nad5P324*	orf410	LAGLIDADG	1,233
*nad5P426*	orf250	LAGLIDADG	753
*rnLP1767*	orf319	LAGLIDADG	915-963
*rnLP2491*	orf130	LAGLIDADG	696
*rnLP2831*	orf879	DNA polymerase type-B	2,646
	orf152	NA	459
	orf413	NA	1,242
*rnSP879*	orf290	LAGLIDADG	861

Note. NA: Not Available.

Cluster analysis based on intron presence/absence divided *G. lingzhi* samples into two major groups: Group 1 (107 samples) and Group 2 (50 samples) (Fig. 2). *cox1P731*, *cox1P894*, and *cox1P1057* were specific to Group 2, while *cox1P276*, *cox1P709*, and *cox1P867* were specific to Group 1. Analysis of coding, intronic, and other regions revealed minimal variation in coding and non-intronic regions (Figs 2, 3B, Suppl. material 1: table S4). Furthermore, the length of the repetitive sequences shows almost no variation among the 157 *G. lingzhi* samples (Fig. 3B, Suppl. material 1: table S5). Correlation analysis revealed a significant positive relationship between mitochondrial intron length and mitogenome size in *G. lingzhi*, with an R-squared value of 0.9581 (Fig. 3C). Therefore, the length variation in *G. lingzhi* mitogenomes was primarily attributed to differences in intron number and length, with non-intronic regions being relatively conserved.

### SNPs in the mitochondrial coding regions of *G. lingzhi*

A total of 32 SNPs were identified within the mitochondrial coding regions of *G. lingzhi* (Suppl. material 1: table S6). Among them, 7 were located in rRNA genes (3 in *rnL* and 4 in *rnS*), and one was found in the tRNA gene *trnL*-*TAA.* The remaining 24 SNPs were situated in PCGs, with one SNP found in each of the following genes: *atp9*, *cox1*, *cob*, and *atp6*; two SNPs in each of *cox3*, *cox2*, and *nad1*; three in each of *nad2* and *nad4*; and four in each of *nad5* and *rps3*. Notably, 20 of these 24 SNPs in protein-coding genes were synonymous substitutions. The four exceptions that resulted in amino acid changes included an A-to-G transition at position 1,720 in the *nad5* gene, changing a serine (AGT) to glycine (GGT); a tri-allelic SNP at position 866 in the *rps3* gene where an A-to-G change resulted in a lysine (AAA) to arginine (AGA) substitution and an A-to-T change led to a lysine (AAA) to isoleucine (ATA) substitution; an A-to-T transversion at position 196 in the *nad2* gene, causing a threonine (ACC) to serine (TCC) change; and a T-to-A transversion at position 36 in the *nad1* gene, resulting in an asparagine (AAT) to lysine (AAA) substitution.

### Mitochondrial gene rearrangement in *G. lingzhi* and related species

**Figure 4.**
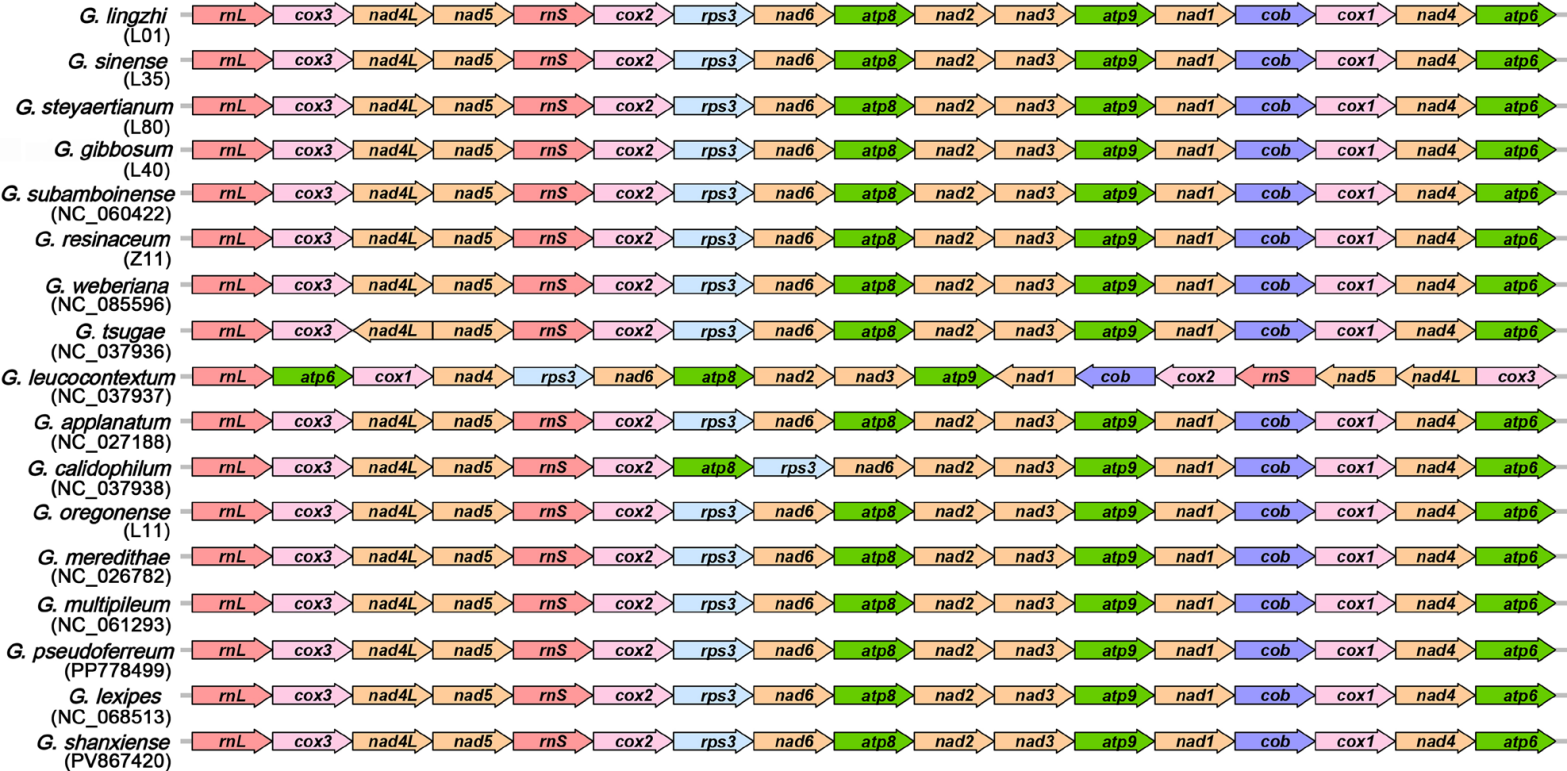
Gene order of mitogenomes of 17 different *Ganoderma* species. The 15 core PCGs and 2 rRNA genes are included in the gene arrangement, starting from the *rnL* gene. The colored arrow blocks indicate different categories of genes. The direction of the arrow indicates the direction of the gene.

Comparison of mitochondrial gene arrangements revealed that 14 of 17 *Ganoderma* species shared conserved gene order and orientation: *rnL*-*cox3*-*nad4L*-*nad5*-*rnS*-*cox2*-*rps3*-*nad6*-*atp8*-*nad2*-*nad3*-*atp9*-*nad1*-*cob*-*cox1*-*nad4*-*atp6* (Fig. 4). Three exceptions were noted: *G. tsugae* showed reversed orientation of *nad4L*, suggesting inversion events; *G. calidophilum* exhibited rearrangement with exchanged positions of *rps3* and *nad6* with *atp8*; *G. leucocontextum* showed extensive rearrangements with four conserved gene clusters: (1) *rps3*-*nad6*-*atp8*-*nad2*-*nad3*-*atp9*; (2) *nad1*-*cob*; (3) *nad4L*-*nad5*-*rns*-*cox2*; (4) *cox1*-*nad4*, but with disrupted order and orientation compared to other species. No gene duplication events were observed in any of the *Ganoderma* species.

### Ka/Ks of mitochondrial PCGs in *Ganoderma* species

**Figure 5.**
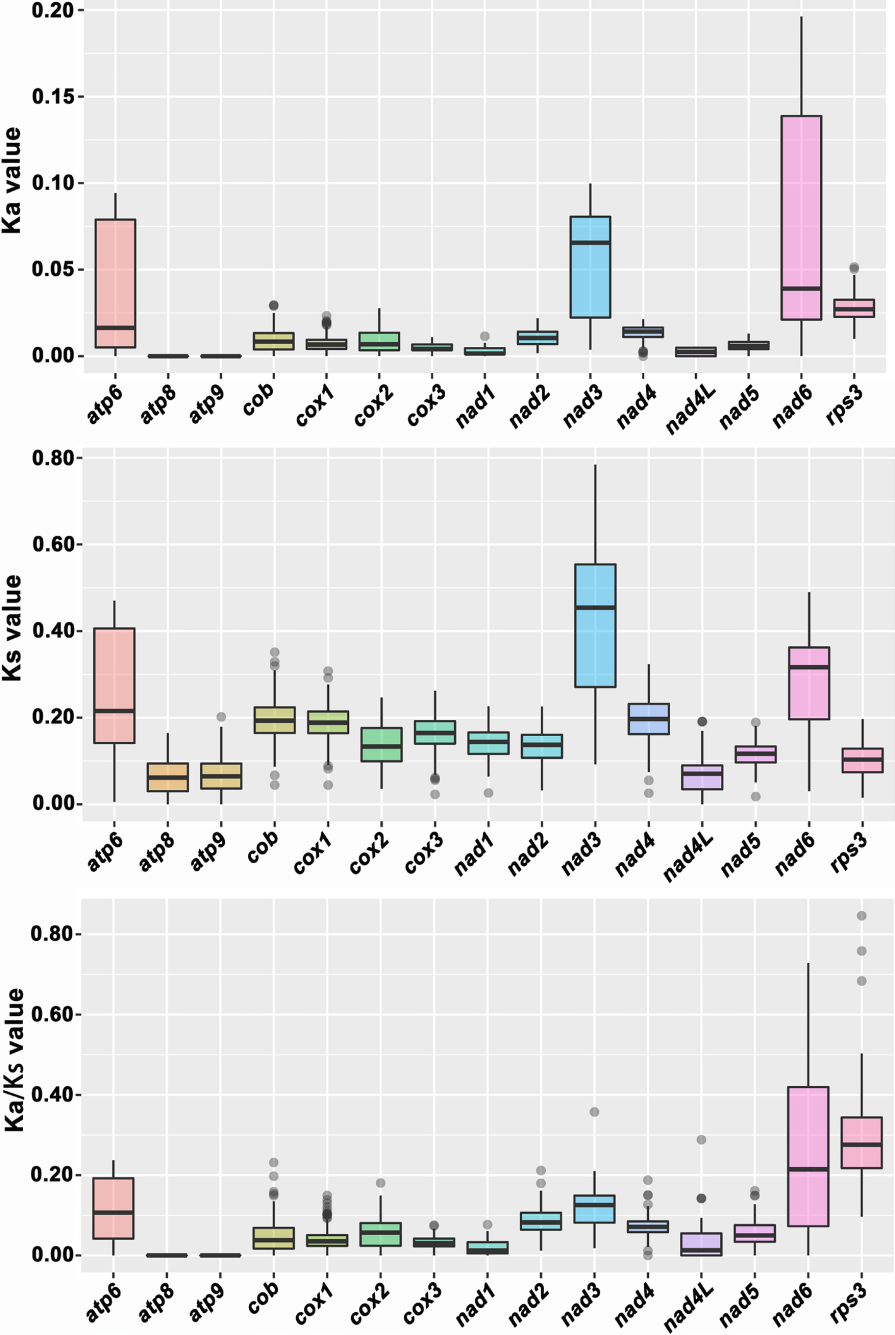
Genetic analysis of 15 core PCGs amongst 17 *Ganoderma* mitogenomes. Ka means the number of non-synonymous substitutions per non-synonymous site; Ks means the number of synonymous substitutions per synonymous site. Ka/Ks represents the ratio of Ka to Ks. The horizontal axis represents different genes, and the vertical axis represents specific values. We used box plots to represent the data of each gene.

Among the 15 core mitochondrial PCGs analyzed, *nad6* exhibited the highest Ka value (Fig. 5, Suppl. material 1: table S7), with a maximum value of 0.1963 and the highest average Ka value (0.0798). In contrast, *atp8* and *atp9* showed the lowest Ka values (both 0), indicating no nonsynonymous substitution sites were observed across the species. The Ks value was highest in *nad3*, with a maximum of 0.7846, while *atp8*, *atp9*, and *nad4L* had the lowest Ks values, each with a minimum of 0. The highest Ka/Ks ratio was observed in *rps3*, reaching a maximum of 0.8462, with an average ratio of 0.2941. These findings collectively indicate that the mitochondrial PCGs in *Ganoderma* species have generally low Ka, Ks, and Ka/Ks values, suggesting that these genes have predominantly undergone purifying selection.

### Phylogenetic analysis

**Figure 6.**
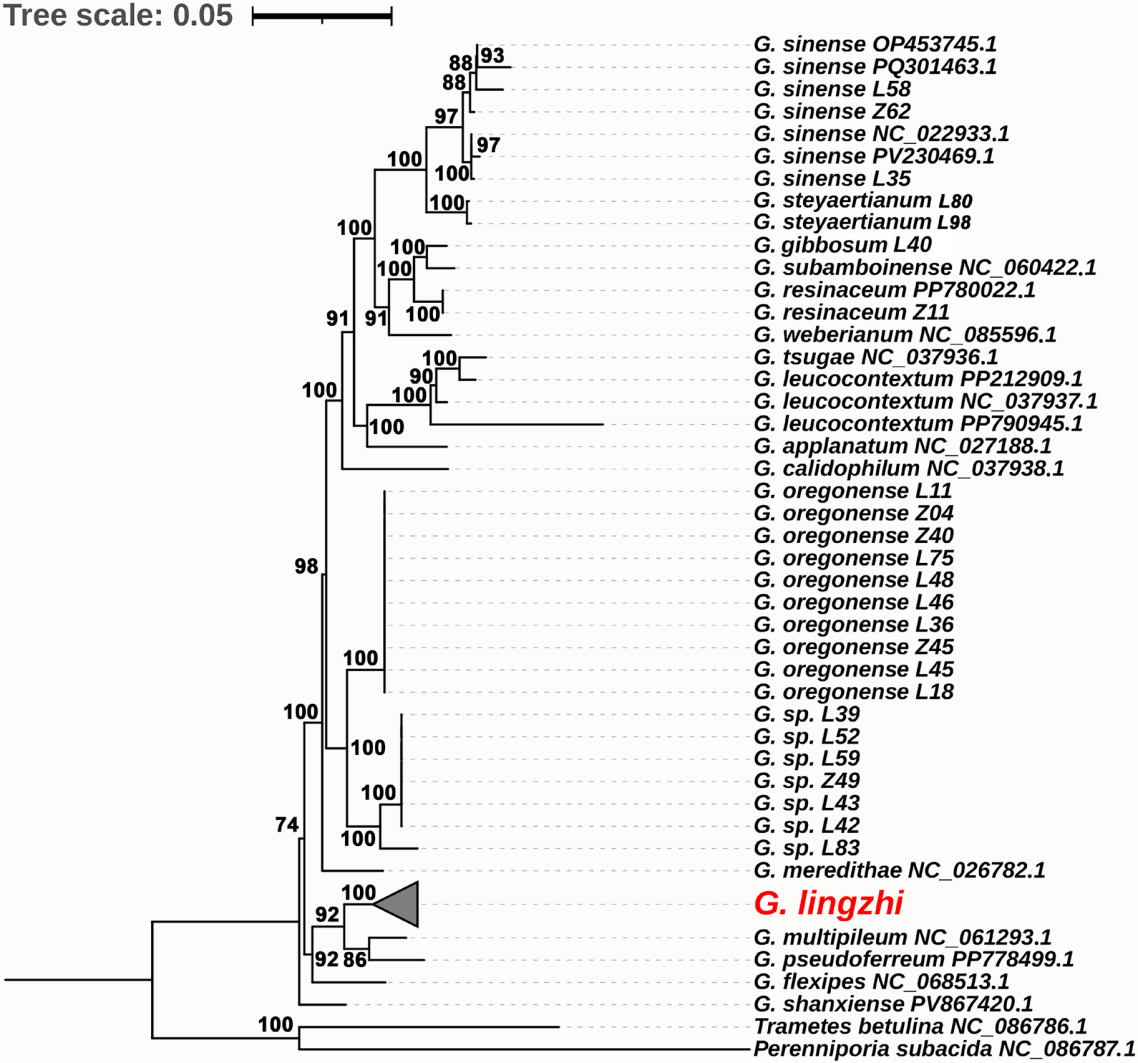
Phylogenetic relationships of *Ganoderma* species. We constructed a maximum likelihood (ML) phylogenetic tree using IQ-TREE2 based on the nucleotide sequences of 15 mitochondrial core PCGs, which were aligned with MAFFT. The bootstrap value was set to 1,000 replicates to assess branch support. *Perenniporia subacida* and *Trametes betulina* were designated as the outgroups. The detailed list of species used in this analysis is provided in Suppl. material 1: table SS2. The scale bar in the figure represents the evolutionary distance, which quantifies the number of substitutions per site. A scale value of 0.05 indicates that a branch length of 1.0 corresponds to an estimated 5 substitutions per 100 sites. All *G. lingzhi* samples formed a monophyletic clade, which was collapsed and is highlighted in red in the figure.

Based on 15 shared mitochondrial PCGs, we conducted a phylogenetic analysis of *Ganoderma* species. The results show that all *Ganoderma* species clustered into a single clade, indicating clear monophyly (Fig. 6). Among them, *G. shanxiense* was placed at the most basal position within the genus, while *G. pseudoferreum* and *G. multipileum* were the most closely related, forming a distinct cluster. All *G. lingzhi* samples grouped together into a separate clade, which appeared as a sister group to the clade containing *G. pseudoferreum* and *G. multipileum*, indicating that among the species analyzed, *G. pseudoferreum* and *G. multipileum* are the closest relatives to *G. lingzhi*.

We also observed that the mitochondrial DNA based phylogenetic tree revealed that, among the 181 samples collected, 24 did not cluster with *G. lingzhi*. This result is consistent with the topology of the nrRNAs-based phylogenetic tree and also corroborates the previous clustering results from nuclear genome SNP analysis (Sun et al. 2024). Together, these three lines of evidence confirm that these 8 samples were previously misidentified.

**Figure 7.**
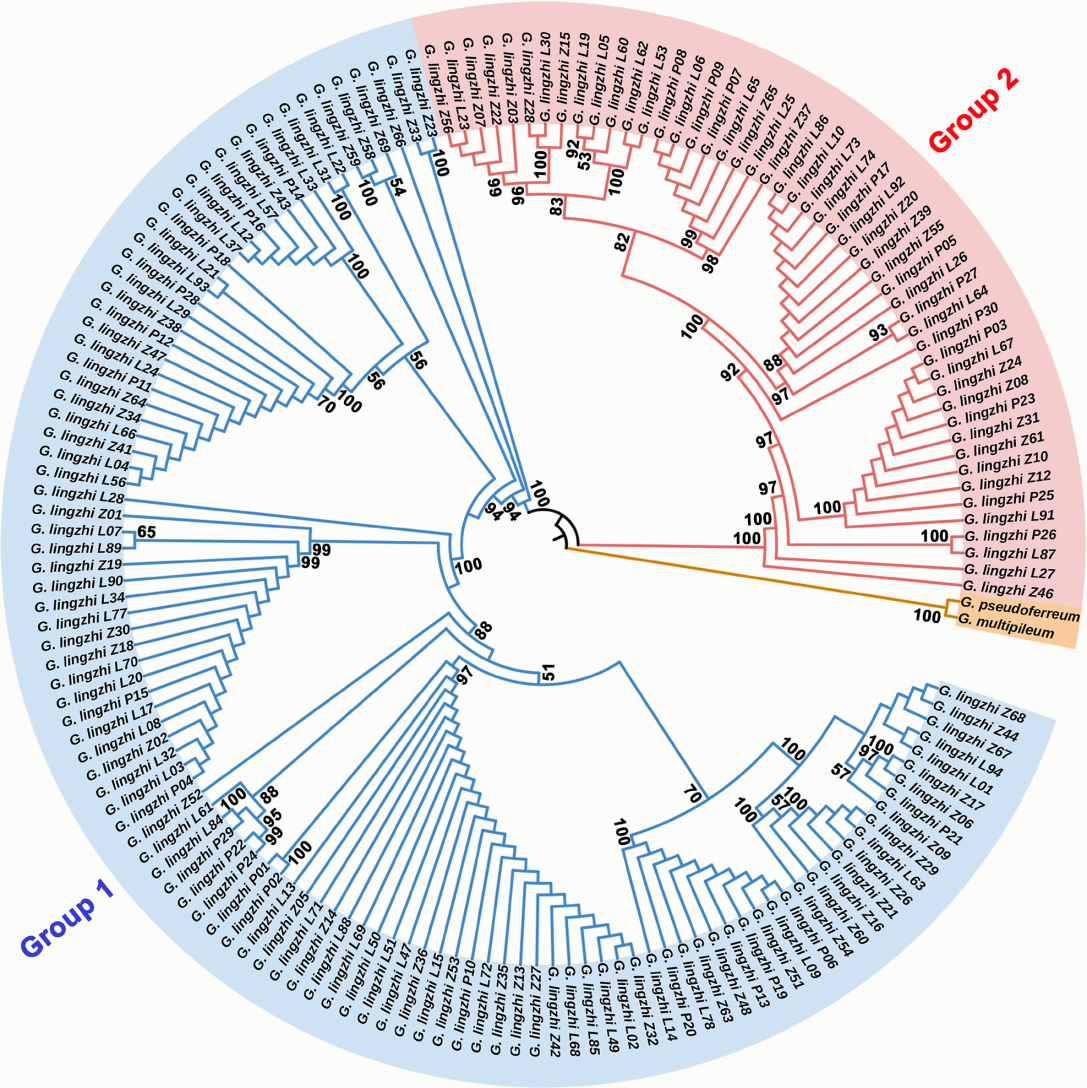
Phylogenetic relationships within *G. lingzhi*. The intraspecific phylogeny of *G. lingzhi* reveals two major clades, a division consistent with the presence/absence clustering pattern of introns.

We observed that the 157 *G. lingzhi* samples were mainly divided into two major groups: Group 1, comprising 107 samples, and Group 2, comprising 50 samples (Fig. 7). This clustering pattern of the two main groups is entirely consistent with the grouping based on intron distribution patterns.

### Intron presence/absence patterns and their phylogenetic implications

Analysis of the *G. lingzhi* mitogenome revealed that its introns are highly dynamic, with their presence/absence states exhibiting rich variation. We systematically recorded the presence (“+”) or absence (“-”) patterns of specific introns in each sample, an approach termed Intron Presence/Absence Variation (IPAV) profiling. As shown in Suppl. material 1: table S8, each unique combination defined a distinct mitochondrial haplotype. Among the 157 samples, this revealed 30 unique mitochondrial haplotypes (MtHap-GL01 to MtHap-GL30), highlighting the remarkable structural diversity within the G. lingzhi population’s mitogenomes. The presence/absence of introns in the *cox1* gene constituted the primary source of this variation and effectively distinguished two major clusters of variation patterns.

However, the evolutionary interpretation of these diversity patterns requires caution. Although we observed 30 haplotypes (Suppl. material 1: table S8), in-depth phylogenetic analysis indicated that the presence/absence states of the vast majority of introns did not show clear lineage specificity. Only a few introns at the basal nodes of the phylogeny, such as the ones shared by all samples (*cox2P228*, *nad5P426*, *cox1P1305*) and those specific to either Group 1 or Group 2 (see Suppl. material 4), could be reliably traced to ancient vertical inheritance events or lineage-specific acquisition events post-diversification. The distribution patterns of most other introns are more likely associated with incomplete lineage sorting, recent horizontal transfer events, or independent loss events across multiple lineages. This complex dynamic background implies that the presence/absence state of a single intron, as a trait of a mobile genetic element, may evolve at a rate far exceeding that of core protein-coding sequences. Consequently, it is not suitable as a robust marker for inferring deep phylogenetic relationships on its own.

## Discussion

In this study, we collected a total of 151 *Ganoderma* strains. Among them, 134 were identified as *G. lingzhi* by growers or culture collections, while the remaining 17 were labeled as other *Ganoderma* species. Initial species identification based on the complete nuclear ribosomal RNA (18S-ITS1-5.8S-ITS2-28S) sequences revealed that, of the 151 strains, only 127 were confirmed to be true *G. lingzhi*, whereas 24 did not belong to this species. Specifically, 13 strains previously regarded as *G. lingzhi* were in fact not, while 6 strains not originally considered to be *G. lingzhi* were found to cluster within the *G. lingzhi* clade. This result indicates that misidentification of strains is not uncommon in practical production and application. Among the 19 strains whose molecular identification contradicted their claimed identity, 7 were obtained from culture collections, 4 from growers, and 8 from various research institutions or companies. The morphological similarity of *G. lingzhi* strains has contributed to confusion in variety identification, especially at the mycelial stage where distinctive taxonomic features are lacking. Our findings demonstrate that nrRNAs-based sequencing can effectively discriminate *G. lingzhi* from its closely related species at the interspecific level. However, given the short length and limited informative sites of the nrRNAs, further strain differentiation within *G. lingzhi* using this marker is challenging (Schoch et al. 2012). This underscores the need to identify new, stable genetic loci for developing reliable intraspecific molecular markers.

We also compared mitogenome differences between *G. lingzhi* and its closely related species. Species within the genus *Ganoderma* exhibited even greater size variation, with the smallest (*G. lingzhi* at 49,233 bp) being only 45.34% the size of the largest (*G. oregonense* at 108,595 bp). Despite this, their gene order was conserved. Large-scale genomic rearrangement was observed in only one species, *G. leucocontextum*, where the gene order was substantially disrupted. Two other species showed minor rearrangements, but overall, gene order was largely conserved across most species. Ka/Ks analysis indicated that the mitochondrial PCGs in *Ganoderma* have undergone purifying selection, as neither the Ka values, Ks values, nor the Ka/Ks ratios exceeded 1. This result further underscores the sequence conservation at the gene level among different *Ganoderma* species.

The mitogenome, with its high copy number, haploid nature, and ease of specific amplification (Otten and Smeets 2015; Liu and Huang 2022), represents an ideal genetic resource for marker development (Croll et al. 2008). Among the 157 *G. lingzhi* samples we examined, we observed considerable variation in mitogenome size, ranging from 49,233 bp to 70,498 bp, with a difference of over 21,265 bp, reflecting substantial intraspecific size diversity. Overall, the lengths of the core PCGs in these samples were conserved (20,062–20,472 bp), and the lengths of intergenic regions also varied, though not as markedly (20,806–26,841 bp). Analysis of repetitive sequences indicated that the mitogenomes of *G. lingzhi* largely lack dispersed repeat elements, with their number generally limited to 1–4 and their length ranging from 304 to 746 bp. In contrast, variation in the number and length of introns emerged as the primary contributor to genome size differences. Across the 157 samples, we detected intron insertions at 20 distinct sites within eight genes. The number of mitochondrial introns per strain ranged from 7 to 16, and their individual lengths varied from 8,270 to 24,374 bp. Correlation analysis revealed an exceptionally strong positive relationship between total intron length and genome size (R^2^ = 0.9581), clearly indicating that dynamic changes in introns are the principal factor driving mitogenome size variation in *G. lingzhi*.

The underlying mechanism for intron gains and loss involves the proliferation of mobile genetic elements and the accumulation of sequences under neutral evolution (Dujon et al. 1986; Kolesnikova et al. 2019). Many Group I and II introns encode homing endonucleases, such as the LAGLIDADG (Toor and Zimmerly 2002; Megarioti and Kouvelis 2020) or GIY-YIG families (Paquin et al. 1995; Van Roey et al. 2002), which can mediate the replication and hopping of intron sequences to new genomic sites, leading to substantial accumulation and expansion of intronic regions. Because non-coding regions such as introns are under relatively weak purifying selection, repetitive or non-functional sequences can accumulate there without lethal consequences for the cell, thereby significantly increasing both genomic and intronic lengths. This phenomenon is not unique to *G. lingzhi* and has been documented in diverse fungi, such as yeast (Tao et al. 2019), *Chrysoporthe* (Kanzi et al. 2016), and *Cordyceps militaris* (Zhang et al. 2015).

Among the 20 unique mitochondrial introns we identified, 19 possess the typical LAGLIDADG or GIY-YIG domains. This result strongly supports the classification of these introns as mobile introns, and the presence of homing endonuclease domains provides direct molecular evidence for their dissemination within the genome or horizontal transfer via the “homing” mechanism. However, a notable exception is the intron located at *rnLP2831*. This intron encoded three ORFs: two of which lacked any known conserved domains, while the third contained a family B DNA polymerase (PolB) domain. This finding markedly differs from the typical pattern of mobile introns encoding homing endonucleases. We propose that this likely reflects a more complex evolutionary event. A plausible explanation is that the *PolB* gene is not intrinsic to the intron itself but originates from an integration event of a mitochondrial plasmid. In fungi, mitochondrial plasmids often carry a *PolB* gene for their own replication. This exceptional case reveals that, beyond frequent intron mobility, the *G. lingzhi* mitogenome may also incorporate other types of mobile genetic elements (e.g., plasmids), thereby increasing its complexity and plasticity. This phenomenon is not an isolated case, for example, in the edible basidiomycete *Agrocybe aegerita*, a 1,716–nucleotide mitochondrial ORF has also been identified as encoding a family B DNA polymerase (Bois et al. 1999).

Analysis of the intron distribution pattern in the *G. lingzhi* mitogenome revealed that three introns (*cox2P228*, *nad5P426*, and *cox1P1305*) were present in all 157 strains. This suggests that they are likely ancient introns acquired in the most recent common ancestor of the current *G. lingzhi* population. Our data indicate that they have been fixed through strict vertical inheritance, possibly stabilized by tight coupling with the splicing machinery, and thus represent conserved features of the species’ mitogenome. In contrast, *cox1P731*, *cox1P894*, and *cox1P1057* were specific to Group 2, whereas *cox1P276*, *cox1P709*, and *cox1P867* were specific to Group 1. These lineage-specific introns strongly suggest that novel acquisition events have continued to occur following the initial major burst of intron gains. They were likely acquired independently in Group 1 and Group 2 after their divergence, via mechanisms such as horizontal transfer or homing, and therefore serve as robust lineage-specific molecular markers, consistent with the phylogeny reconstructed from protein-coding sequences.

Most of the remaining introns did not exhibit strict lineage specificity. A plausible explanation is incomplete lineage sorting, whereby these introns were acquired around the time of lineage divergence and have not yet had sufficient time to become fixed within the populations, resulting in a distribution that does not clearly correspond to the major lineage boundaries. Another possibility is that some introns retain high mobility and can undergo recent horizontal transfer between different lineages or even strains, leading to the observed complex distribution pattern. Additionally, the independent loss of some ancient, shared introns in multiple lineages cannot be ruled out. Together, these mechanisms contribute to a dynamic genomic background in which intron gain and loss are ongoing processes.

Multiple studies have confirmed that intron content is a major determinant of mitogenome size variation in fungi (Zhang et al. 2015; Kanzi et al. 2016; Tao et al. 2019; Wang et al. 2025b). Consequently, intron dynamics can be regarded as a focal point for understanding the population-level evolution of the *G. lingzhi* mitogenome. The marked variation in intron number (7–16) and length among different *G. lingzhi* strains suggests that specific intron presence/absence patterns and length characteristics hold potential as efficient DNA fingerprints for distinguishing intraspecific strains. Clustering based on intron states divided the strains into two major groups, a clustering pattern supported by phylogenetic inference derived from sequences of 15 conserved PCGs. This supports the subdivision of *G. lingzhi* strains into two intraspecific subgroups. In practical terms, these haplotypes can serve as taxonomic labels and molecular markers for inferring population evolutionary history and dispersal dynamics (Li et al. 2024). Furthermore, this haplotype system establishes a reliable molecular benchmark for protecting intellectual property, authenticating varieties, and tracing commercial strains, effectively addressing the industry’s issues of synonymy and misnaming, thereby ensuring product quality and the rational use of germplasm resources.

## Conclusion

In summary, this research provides valuable genomic resources for the germplasm evaluation and molecular breeding of *G. lingzhi*. This large-scale pan-mitogenomic analysis provides deep insights into the patterns and drivers of intraspecific genetic diversity in *G. lingzhi*. It also serves as a significant case study for understanding the micro-evolutionary mechanisms of fungal mitogenomes. Future research should focus on exploring the biological functions of these intron variations and their potential effects on the physiology and secondary metabolite production of *G. lingzhi*.

## Additional information

### Artificial Intelligence (AI) use

Regarding the use of AI in the preparation of this manuscript, the authors declare the following:

**Description**: ChatGPT (OpenAI) was used for English proofreading.

### Adherence to national and international regulations

All the fungal strains used in this study have been legally obtained, respecting the Convention on Biological Diversity (Rio Convention).

### Author ORCIDs

J. Li https://orcid.org/0000-0002-1498-0260

C. Liu https://orcid.org/0000-0003-3879-7302

### Data availability

The assembled mtDNA sequences have been submitted to GenBank (https://www.ncbi.nlm.nih.gov/nuccore/) with accession number: PX713229–PX713385; PX705422–PX705445.

## Supplementary materials

Supplementary material 1 Supplementary tables Data type xlsx https://binary.pensoft.net/file/1667773This dataset is made available under the Open Database License (http://opendatacommons.org/licenses/odbl/1.0/). The Open Database License (ODbL) is a license agreement intended to allow users to freely share, modify, and use this Dataset while maintaining this same freedom for others, provided that the original source and author(s) are credited. Jingling Li, Chang Zhang, Yang Ni, Lei Sun, Xianhao Cheng, Chang Liu, Chang Liu

Supplementary material 2 Supplementary image 1 Data type pdf https://binary.pensoft.net/file/1667774This dataset is made available under the Open Database License (http://opendatacommons.org/licenses/odbl/1.0/). The Open Database License (ODbL) is a license agreement intended to allow users to freely share, modify, and use this Dataset while maintaining this same freedom for others, provided that the original source and author(s) are credited. Jingling Li, Chang Zhang, Yang Ni, Lei Sun, Xianhao Cheng, Chang Liu

Supplementary material 3 Supplementary image 2 Data type pdf https://binary.pensoft.net/file/1667775This dataset is made available under the Open Database License (http://opendatacommons.org/licenses/odbl/1.0/). The Open Database License (ODbL) is a license agreement intended to allow users to freely share, modify, and use this Dataset while maintaining this same freedom for others, provided that the original source and author(s) are credited. Jingling Li, Chang Zhang, Yang Ni, Lei Sun, Xianhao Cheng, Chang Liu

Supplementary material 4 Supplementary image 3 Data type pdf https://binary.pensoft.net/file/1667776This dataset is made available under the Open Database License (http://opendatacommons.org/licenses/odbl/1.0/). The Open Database License (ODbL) is a license agreement intended to allow users to freely share, modify, and use this Dataset while maintaining this same freedom for others, provided that the original source and author(s) are credited. Jingling Li, Chang Zhang, Yang Ni, Lei Sun, Xianhao Cheng, Chang Liu
