# Supplementary material for: Insights into intraspecific variation and genotyping of Ganoderma lingzhi through pan-mitogenome analysis
Source: IMA Fungus. 2026 Jun 3;17:e184941. doi: 10.3897/imafungus.17.184941 (PMC13254553; doi:10.3897/imafungus.17.184941)
Supplement: Supplementary material 2 — Supplementary image 1 [file imafungus-17-e184941-s002.pdf]

Figure S1 Phylogenetic tree based on the nuclear ribosomal RNA sequences. A. The phylogenetic tree that preserve the original branch lengths. **B.** The phylogenetic tree that ignore the original branch lengths. Our dataset comprised sequences (18S-ITS1-5.8S-ITS2-28S) assembled from 181 samples, with the aligned nucleotide sequences reaching a length of 12,512 bp. The results showed that 24 of our samples clustered into one distinct clade (highlighted in red), while the remaining 157 samples formed another clade (highlighted in blue). The genetic distances among the 157 samples were minimal, indicating a very close phylogenetic relationship among them. Combined with BLAST evidence from the NCBI database (data not shown), we identified these 157 samples as belonging to the species *G. lingzhi*. The other 24 samples, which clustered into a separate branch, are likely different species within the genus *Ganoderma*. A scale bar of 0.05 is shown, indicating an evolutionary distance of 0.05 substitutions per site per unit of horizontal branch length.

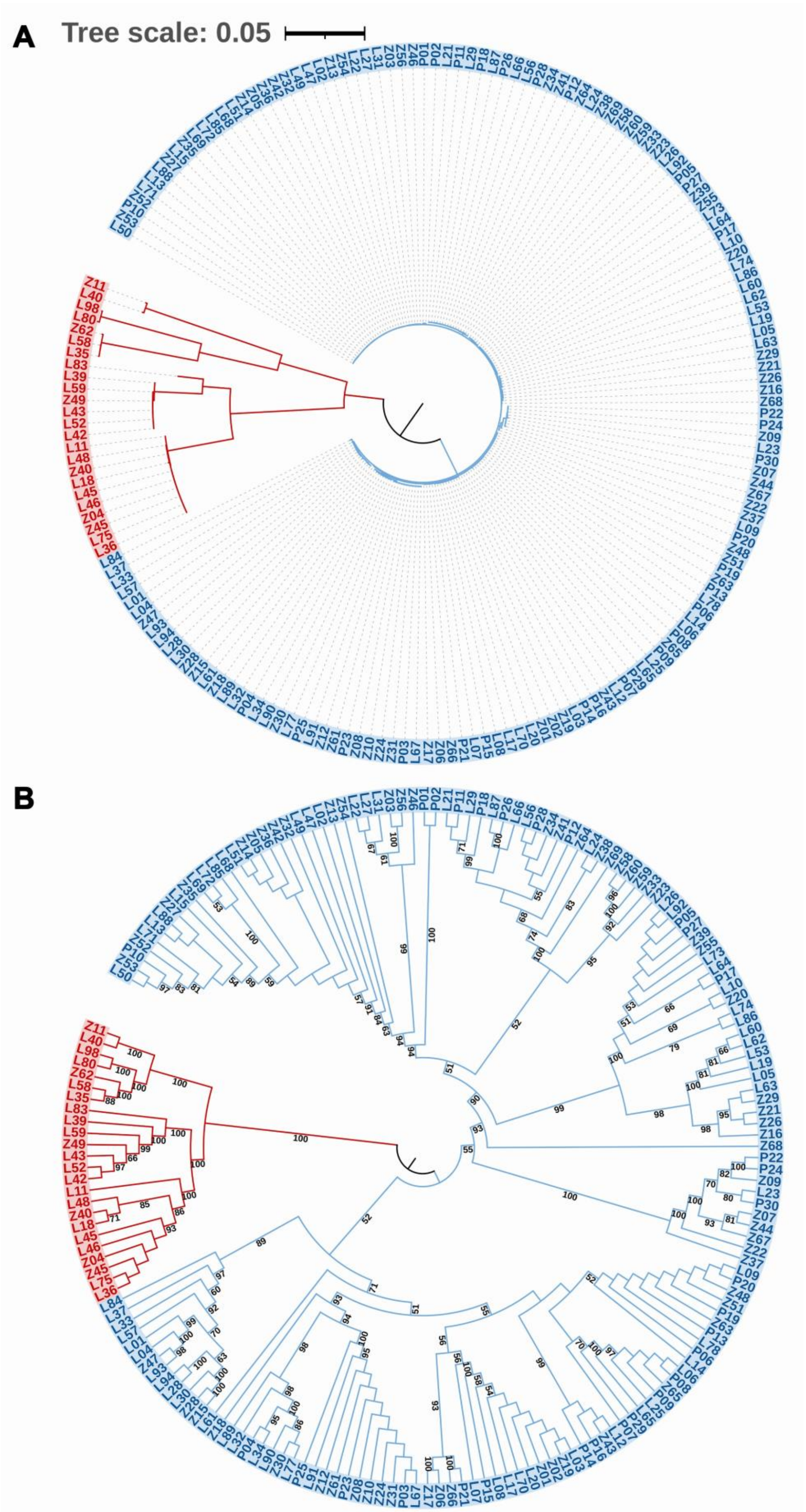
A
Tree scale: 0.05
B
