# Supplementary material for: Insights into intraspecific variation and genotyping of Ganoderma lingzhi through pan-mitogenome analysis
Source: IMA Fungus. 2026 Jun 3;17:e184941. doi: 10.3897/imafungus.17.184941 (PMC13254553; doi:10.3897/imafungus.17.184941)
Supplement: Supplementary material 3 — Supplementary image 2 [file imafungus-17-e184941-s003.pdf]

Figure S2 181 Mitogenome maps of *Ganoderma* species.

The circular maps, generated by OGDRAW, display the complete mitochondrial genome architecture of each sample arranged in concentric rings. Each ring corresponds to one species, as labeled in the center with its name and total genome size in base pairs. Gene modules are plotted on the both side of the outermost ring, with colors representing distinct functional categories. The position and length of each colored block indicate the physical location and span of the corresponding gene on the genome. The inner ring shows the GC content of the mitogenome.

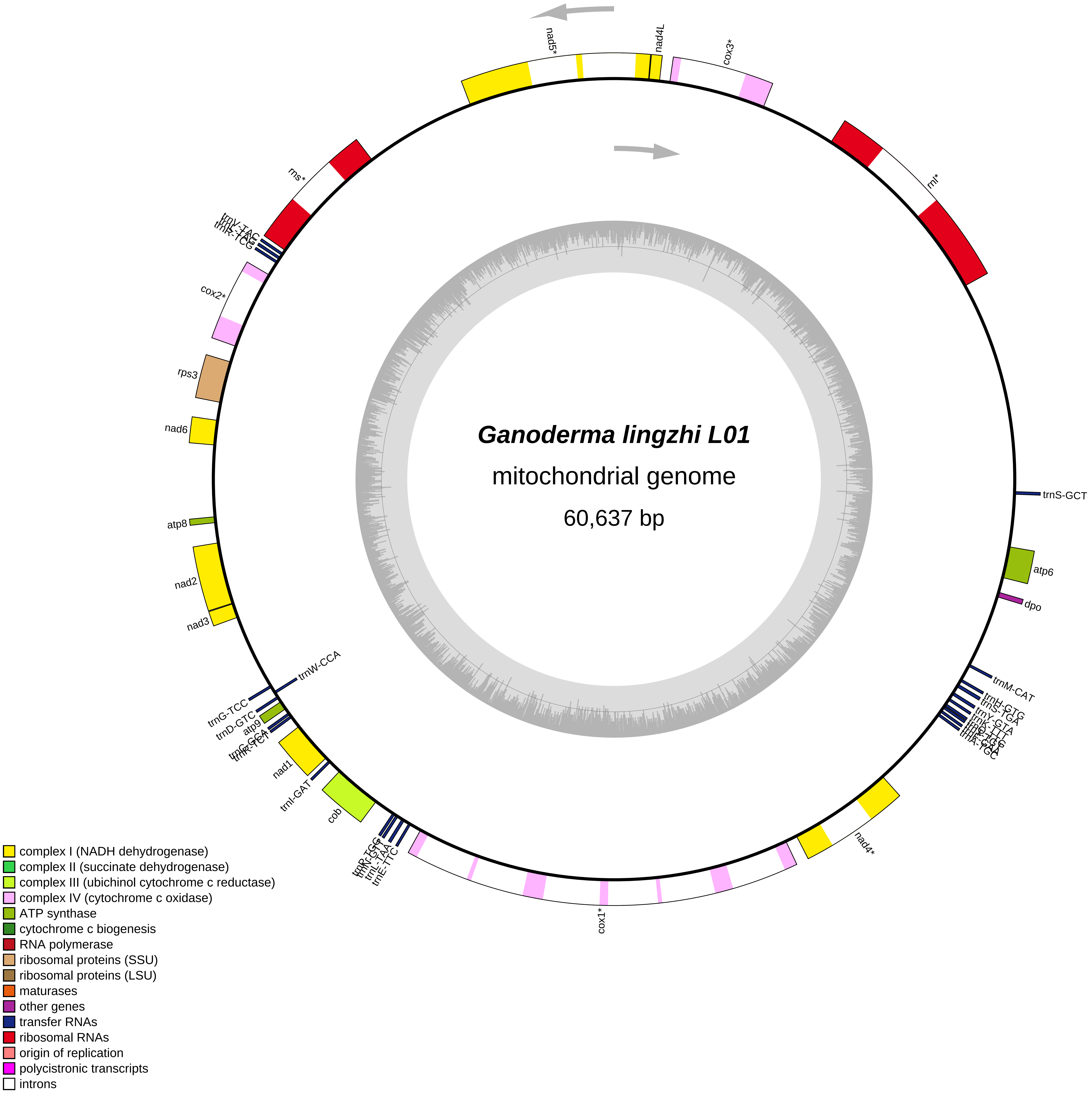

Ganoderma lingzhi L01
mitochondrial genome
60,637 bp
nad5*
nad4L
cox3*
rnl*
rns*
trnV-TAC
trnR-TCG
cox2*
rps3
nad6
trnS-GCT
atp6
dpo
atp8
nad2
nad3
trnW-CCA
trnG-TCC
trnD-GTC
atp9
trnC-GCA
trnR-TCT
nad1
trnI-GAT
cob
trnP-TGG
trnL-TAA
trnE-TTC
cox1*
nad4*
trnM-CAT
trnH-GTG
trnS-TGA
trnY-GTA
complex I (NADH dehydrogenase)
complex II (succinate dehydrogenase)
complex III (ubichinol cytochrome c reductase)
complex IV (cytochrome c oxidase)
ATP synthase
cytochrome c biogenesis
RNA polymerase
ribosomal proteins (SSU)
ribosomal proteins (LSU)
maturases
other genes
transfer RNAs
ribosomal RNAs
origin of replication
polycistronic transcripts
introns

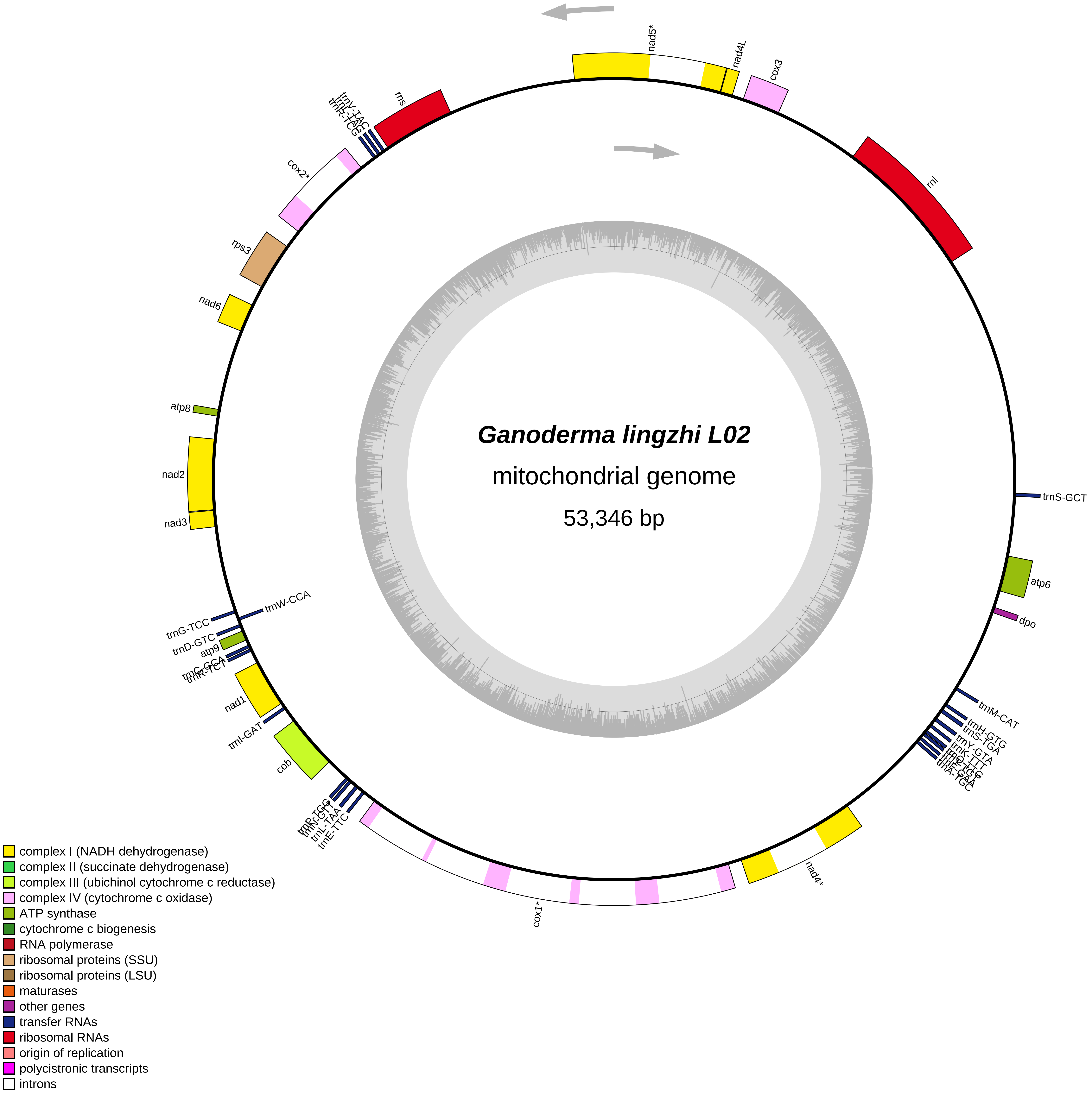

Ganoderma lingzhi L02
mitochondrial genome
53,346 bp
nad5*
nad4L
cox3
rnl
trnS-GCT
atp6
dpo
trnM-CAT
trnH-GTG
trnS-TGA
trnY-GTA
nad4*
cox1*
trnE-TTC
trnL-TAA
cob
trnI-GAT
nad1
trnR-TCT
atp9
trnD-GTC
trnG-TCC
trnW-CCA
nad3
nad2
atp8
nad6
rps3
cox2*
rns
trnV-TAC
complex I (NADH dehydrogenase)
complex II (succinate dehydrogenase)
complex III (ubichinol cytochrome c reductase)
complex IV (cytochrome c oxidase)
ATP synthase
cytochrome c biogenesis
RNA polymerase
ribosomal proteins (SSU)
ribosomal proteins (LSU)
maturases
other genes
transfer RNAs
ribosomal RNAs
origin of replication
polycistronic transcripts
introns

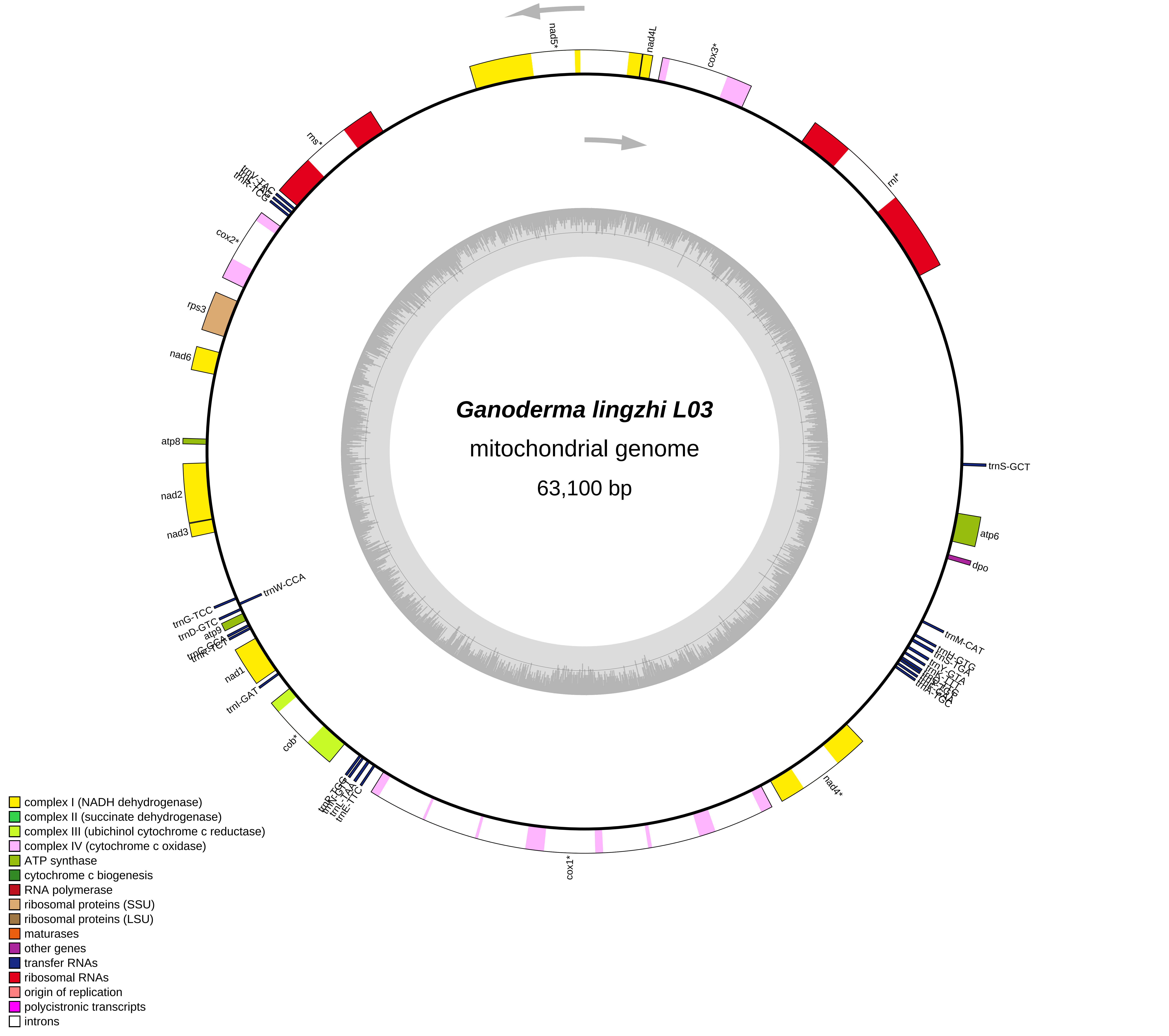
Ganoderma lingzhi L03
mitochondrial genome
63,100 bp
nad5*
nad4L
cox3*
rnl*
trnS-GCT
atp6
dpo
trnM-CAT
trnH-GTG
trnS-TGA
trnY-GTA
nad4*
cox1*
trnE-TTC
trnL-TAA
trnP-TGG
cob*
trnI-GAT
nad1
trnR-TCT
trnC-GCA
atp9
trnD-GTC
trnG-TCC
trnW-CCA
nad3
nad2
atp8
nad6
rps3
cox2*
trnR-TCG
trnV-TAC
rns*
complex I (NADH dehydrogenase)
complex II (succinate dehydrogenase)
complex III (ubichinol cytochrome c reductase)
complex IV (cytochrome c oxidase)
ATP synthase
cytochrome c biogenesis
RNA polymerase
ribosomal proteins (SSU)
ribosomal proteins (LSU)
maturases
other genes
transfer RNAs
ribosomal RNAs
origin of replication
polycistronic transcripts
introns

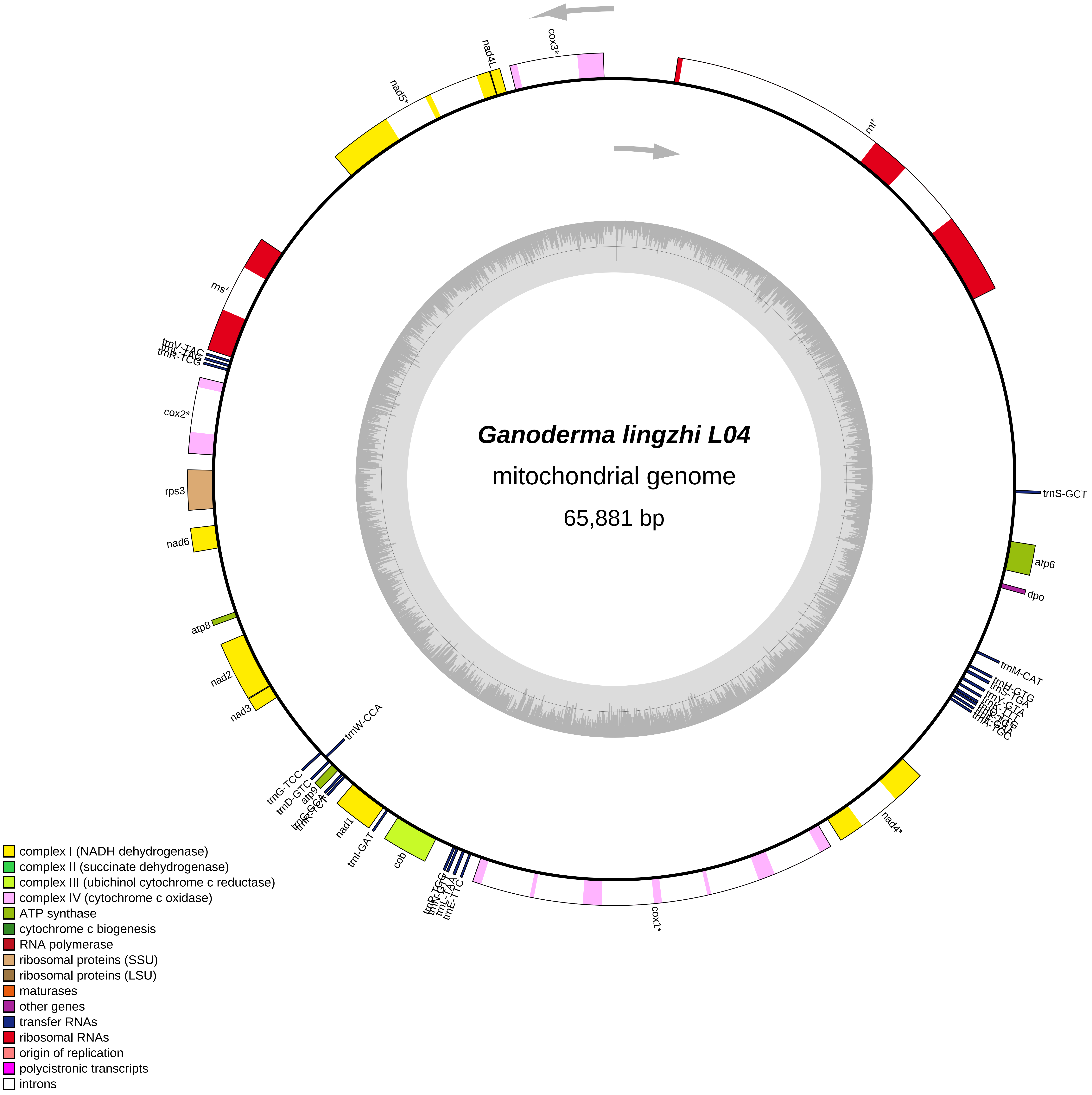

Ganoderma lingzhi L04
mitochondrial genome
65,881 bp
rnl*
trnS-GCT
atp6
dpo
trnM-CAT
trnH-GTG
nad4*
cox1*
trnE-TTC
cob
trnI-GAT
nad1
atp9
trnD-GTC
trnG-TCC
trnW-CCA
nad3
nad2
atp8
nad6
rps3
cox2*
trnR-TCG
trnV-TAC
rns*
nad5*
nad4L
cox3*
complex I (NADH dehydrogenase)
complex II (succinate dehydrogenase)
complex III (ubichinol cytochrome c reductase)
complex IV (cytochrome c oxidase)
ATP synthase
cytochrome c biogenesis
RNA polymerase
ribosomal proteins (SSU)
ribosomal proteins (LSU)
maturases
other genes
transfer RNAs
ribosomal RNAs
origin of replication
polycistronic transcripts
introns

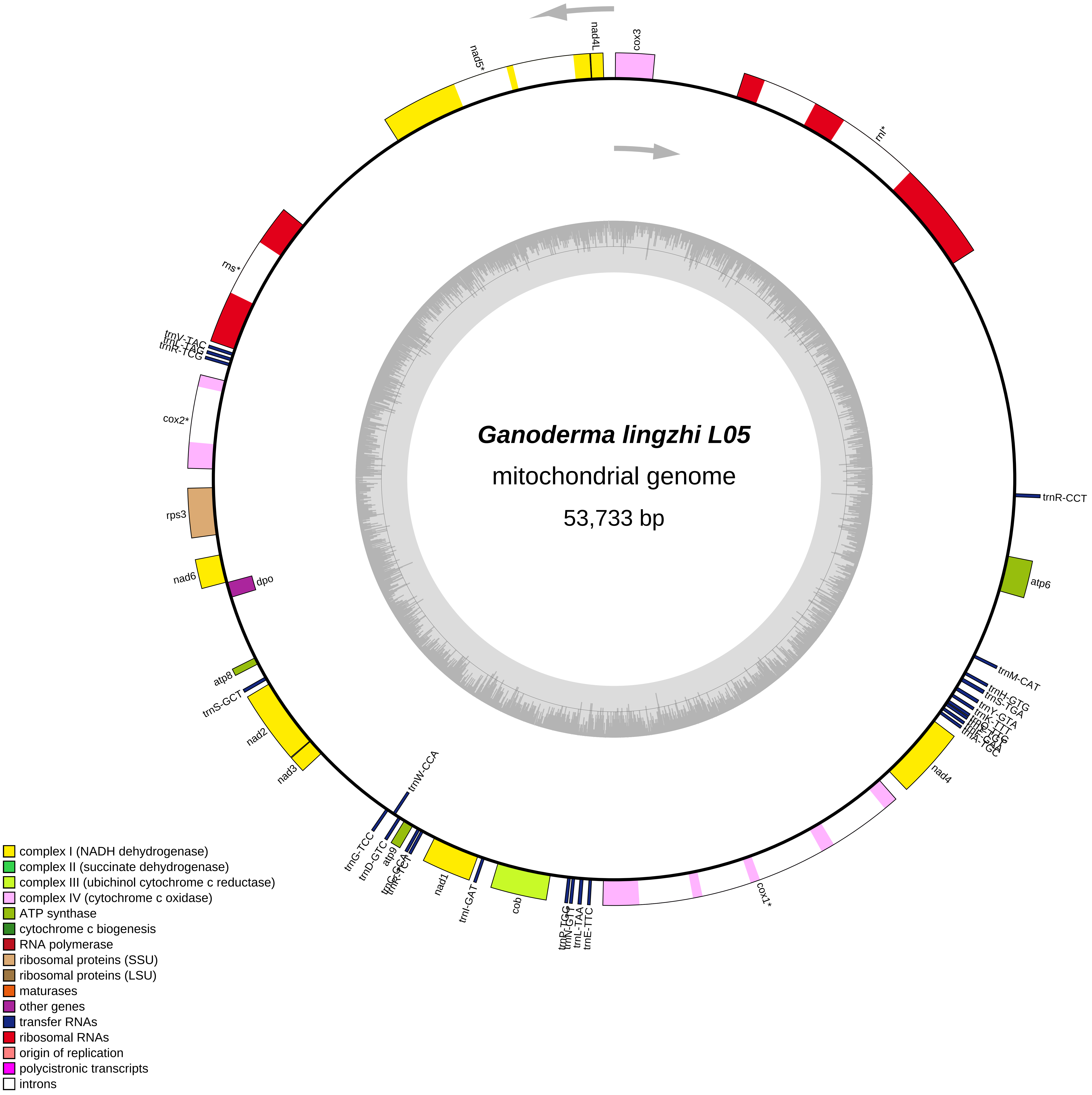

Ganoderma lingzhi L05
mitochondrial genome
53,733 bp
nad5*
nad4L
cox3
rnl*
trnR-CCT
atp6
trnM-CAT
trnH-GTG
trnS-TGA
trnY-GTA
nad4
cox1*
trnE-TTC
trnL-TAA
cob
trnI-GAT
nad1
atp9
trnD-GTC
trnG-TCC
trnW-CCA
nad3
nad2
trnS-GCT
atp8
dpo
nad6
rps3
cox2*
trnV-TAC
rns*
complex I (NADH dehydrogenase)
complex II (succinate dehydrogenase)
complex III (ubichinol cytochrome c reductase)
complex IV (cytochrome c oxidase)
ATP synthase
cytochrome c biogenesis
RNA polymerase
ribosomal proteins (SSU)
ribosomal proteins (LSU)
maturases
other genes
transfer RNAs
ribosomal RNAs
origin of replication
polycistronic transcripts
introns

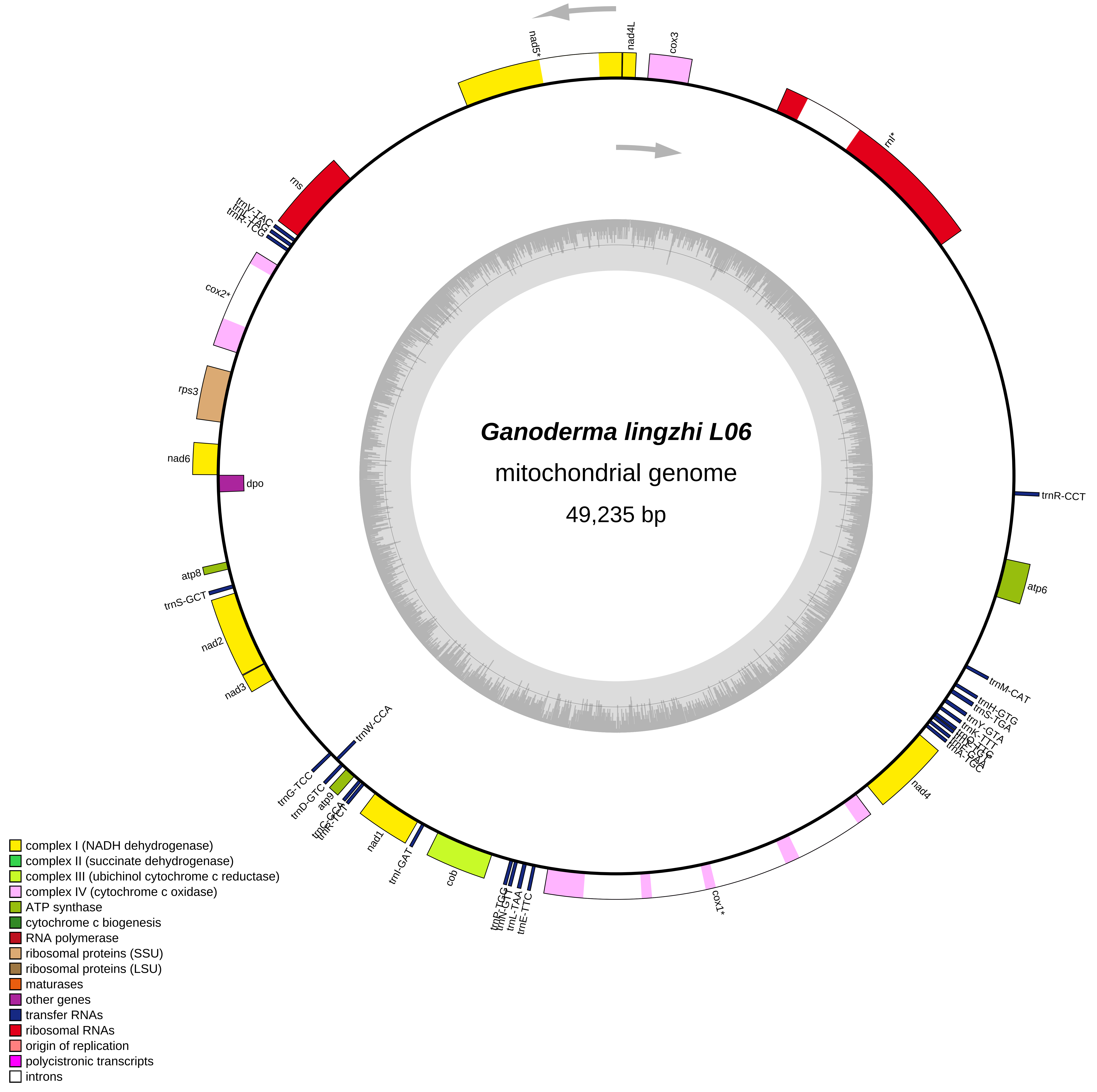

Ganoderma lingzhi L06
mitochondrial genome
49,235 bp
nad5*
nad4L
cox3
rnl*
trnR-CCT
atp6
trnM-CAT
trnH-GTG
trnS-TGA
trnY-GTA
trnK-TTT
trnA-TGC
nad4
cox1*
trnE-TTC
trnL-TAA
trnN-GTT
trnP-TGG
cob
trnI-GAT
nad1
atp9
trnD-GTC
trnG-TCC
trnW-CCA
nad3
nad2
trnS-GCT
atp8
dpo
nad6
rps3
cox2*
trnR-TCG
trnL-TAG
trnV-TAC
rns
complex I (NADH dehydrogenase)
complex II (succinate dehydrogenase)
complex III (ubichinol cytochrome c reductase)
complex IV (cytochrome c oxidase)
ATP synthase
cytochrome c biogenesis
RNA polymerase
ribosomal proteins (SSU)
ribosomal proteins (LSU)
maturases
other genes
transfer RNAs
ribosomal RNAs
origin of replication
polycistronic transcripts
introns

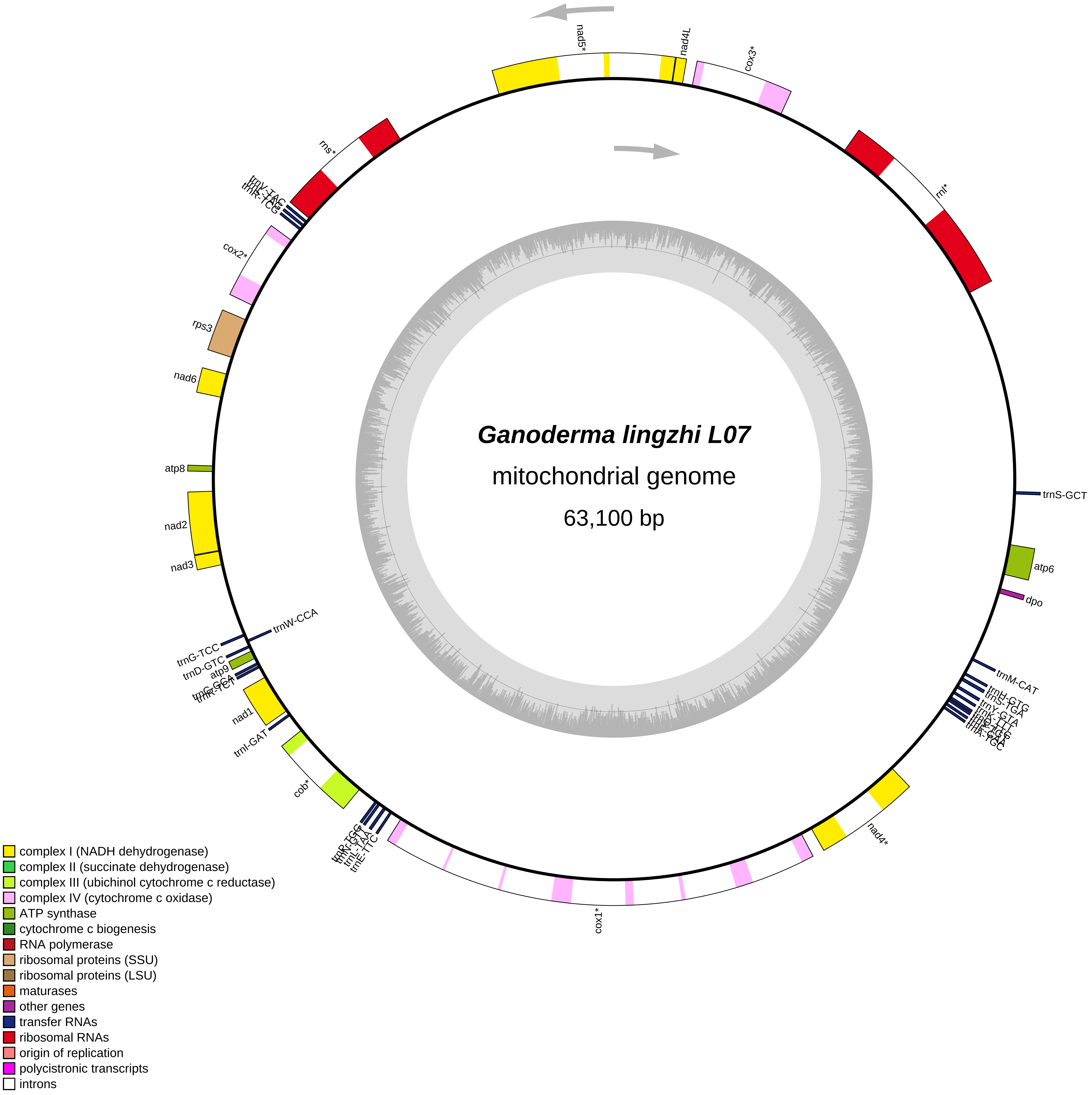
Ganoderma lingzhi L07
mitochondrial genome
63,100 bp
nad5*
nad4L
cox3*
rnl*
trnS-GCT
atp6
dpo
trnM-CAT
trnH-GTG
trnS-TGA
nad4*
cox1*
trnE-TTC
cob*
trnI-GAT
nad1
atp9
trnD-GTC
trnG-TCC
trnW-CCA
nad3
nad2
atp8
nad6
rps3
cox2*
rns*
trnV-TAC
complex I (NADH dehydrogenase)
complex II (succinate dehydrogenase)
complex III (ubichinol cytochrome c reductase)
complex IV (cytochrome c oxidase)
ATP synthase
cytochrome c biogenesis
RNA polymerase
ribosomal proteins (SSU)
ribosomal proteins (LSU)
maturases
other genes
transfer RNAs
ribosomal RNAs
origin of replication
polycistronic transcripts
introns

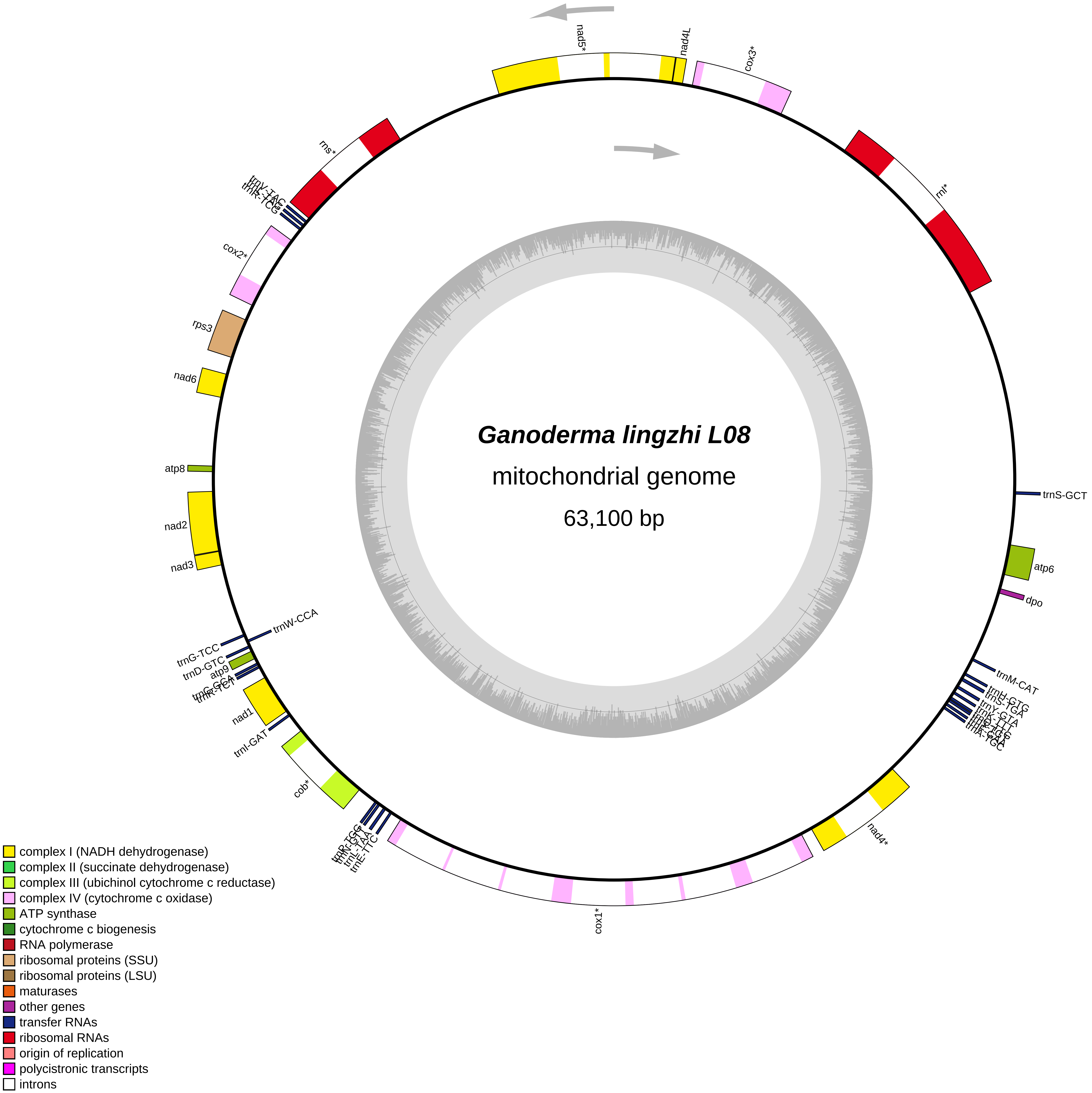

Ganoderma lingzhi L08
mitochondrial genome
63,100 bp
nad5*
nad4L
cox3*
rnl*
trnS-GCT
atp6
dpo
trnM-CAT
trnH-GTG
trnS-TGA
trnY-GTA
nad4*
cox1*
trnE-TTC
trnL-TAA
cob*
trnI-GAT
nad1
trnR-TCT
trnC-GCA
atp9
trnD-GTC
trnG-TCC
trnW-CCA
nad3
nad2
atp8
nad6
rps3
cox2*
trnR-TCG
trnV-TAC
rns*
complex I (NADH dehydrogenase)
complex II (succinate dehydrogenase)
complex III (ubichinol cytochrome c reductase)
complex IV (cytochrome c oxidase)
ATP synthase
cytochrome c biogenesis
RNA polymerase
ribosomal proteins (SSU)
ribosomal proteins (LSU)
maturases
other genes
transfer RNAs
ribosomal RNAs
origin of replication
polycistronic transcripts
introns

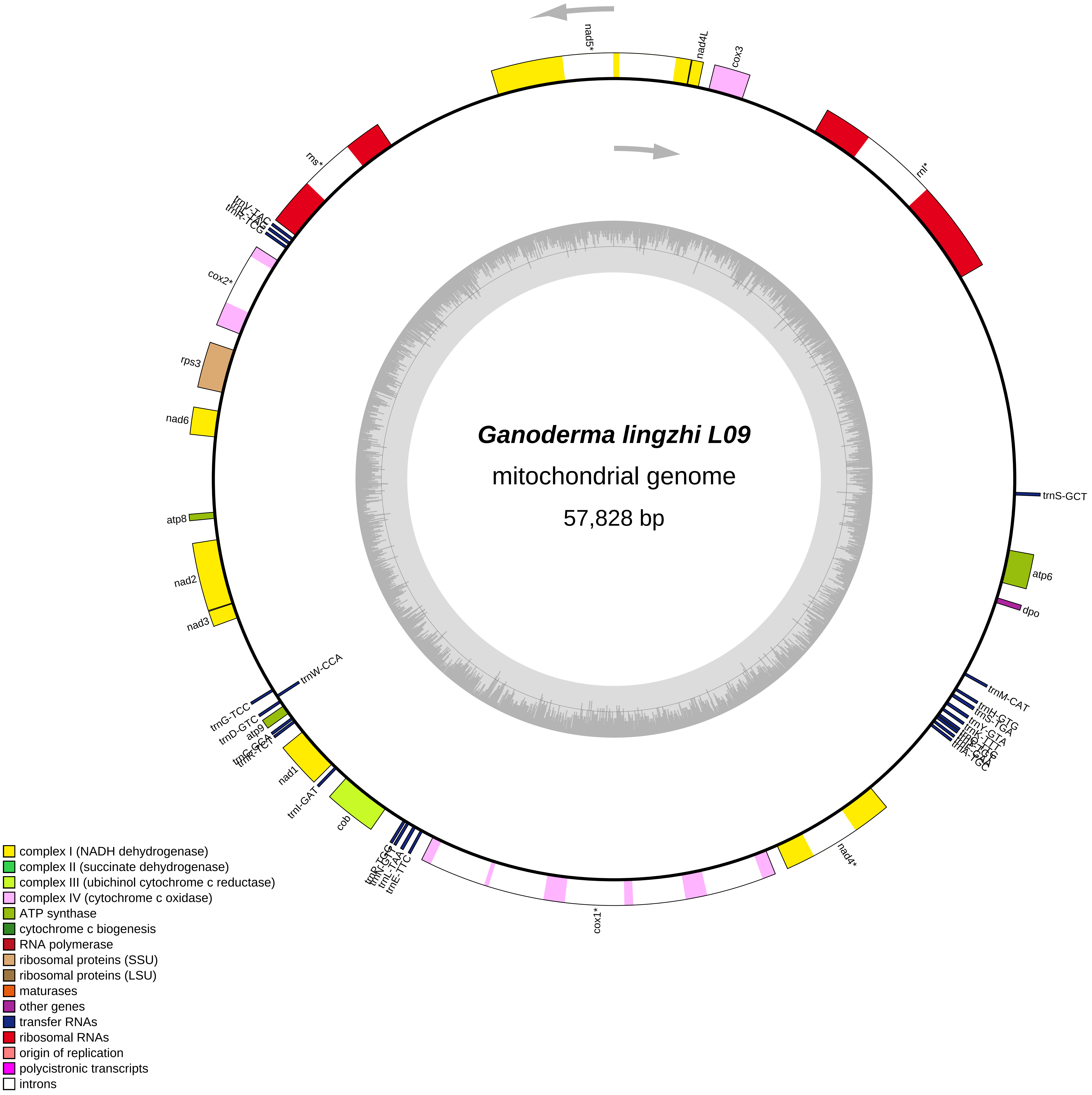

Ganoderma lingzhi L09
mitochondrial genome
57,828 bp
nad5*
nad4L
cox3
rnl*
trnS-GCT
atp6
dpo
trnM-CAT
trnH-GTG
trnS-TGA
trnY-GTA
nad4*
cox1*
trnE-TTC
trnL-TAA
cob
trnI-GAT
nad1
trnR-TCT
trnC-GCA
atp9
trnD-GTC
trnG-TCC
trnW-CCA
nad3
nad2
atp8
nad6
rps3
cox2*
trnR-TCG
trnV-TAC
rns*
complex I (NADH dehydrogenase)
complex II (succinate dehydrogenase)
complex III (ubichinol cytochrome c reductase)
complex IV (cytochrome c oxidase)
ATP synthase
cytochrome c biogenesis
RNA polymerase
ribosomal proteins (SSU)
ribosomal proteins (LSU)
maturases
other genes
transfer RNAs
ribosomal RNAs
origin of replication
polycistronic transcripts
introns

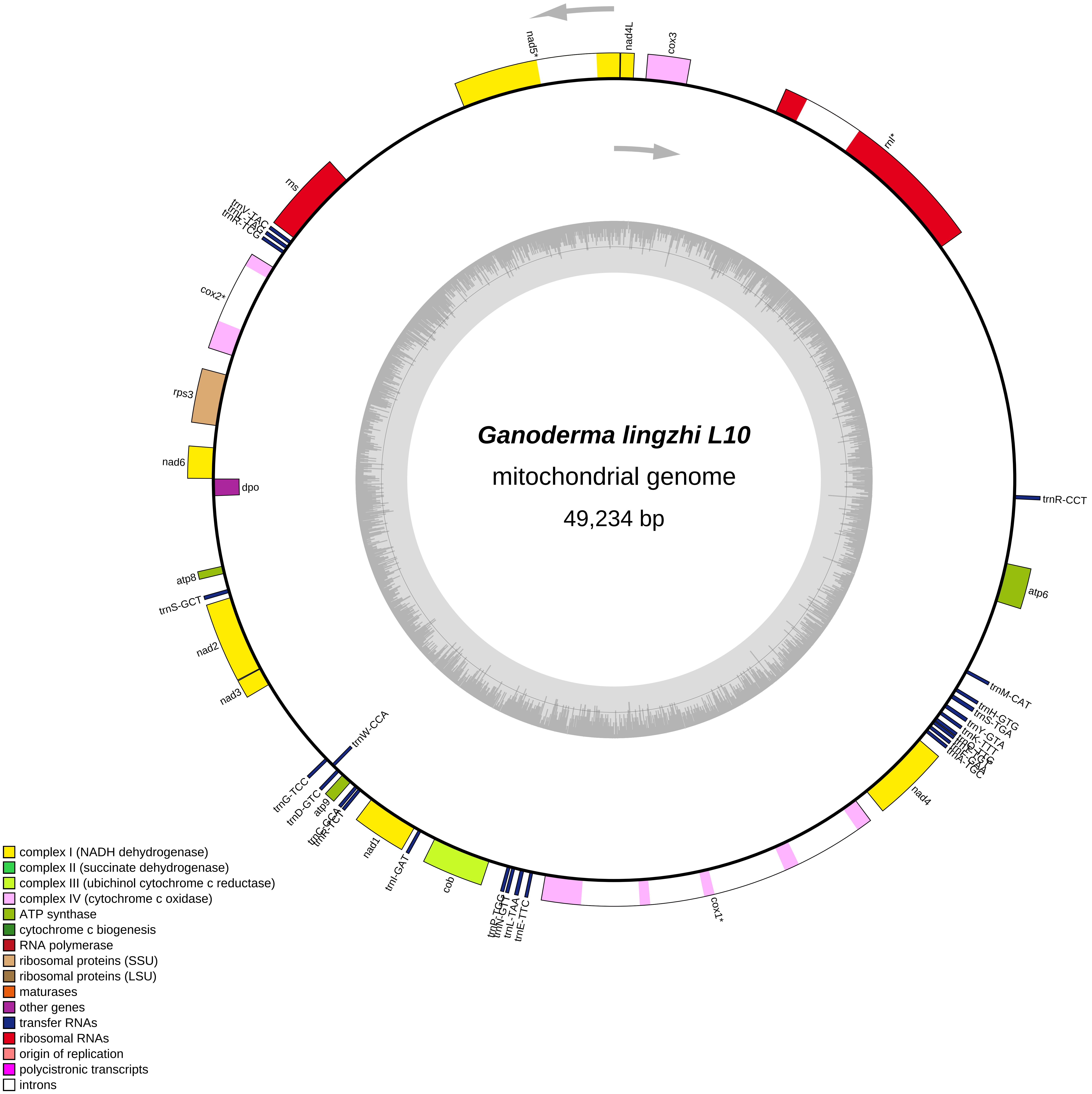
Ganoderma lingzhi L10
mitochondrial genome
49,234 bp
nad5*
nad4L
cox3
rnl*
rns
trnV-TAC
trnR-TCG
cox2*
rps3
nad6
dpo
trnR-CCT
atp6
atp8
trnS-GCT
nad2
nad3
trnM-CAT
trnH-GTG
trnS-TGA
trnY-GTA
trnK-TTT
nad4
trnW-CCA
trnG-TCC
trnD-GTC
atp9
nad1
trnI-GAT
cob
trnP-TGG
trnL-TAA
trnE-TTC
cox1*
complex I (NADH dehydrogenase)
complex II (succinate dehydrogenase)
complex III (ubichinol cytochrome c reductase)
complex IV (cytochrome c oxidase)
ATP synthase
cytochrome c biogenesis
RNA polymerase
ribosomal proteins (SSU)
ribosomal proteins (LSU)
maturases
other genes
transfer RNAs
ribosomal RNAs
origin of replication
polycistronic transcripts
introns

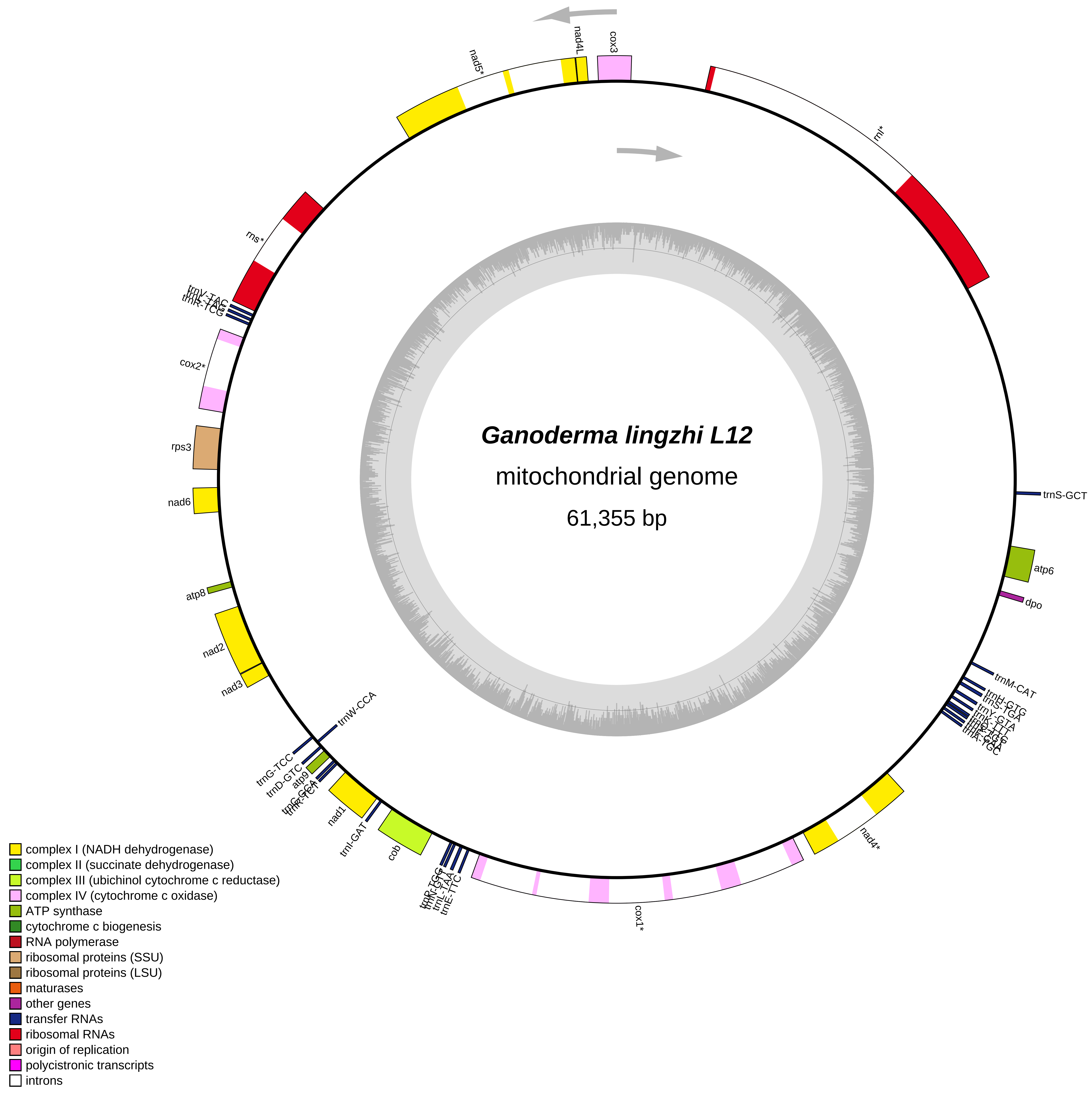
Ganoderma lingzhi L12
mitochondrial genome
61,355 bp
nad5*
nad4L
cox3
rnl*
trnS-GCT
atp6
dpo
trnM-CAT
trnH-GTG
nad4*
cox1*
trnE-TTC
trnL-TAA
cob
trnI-GAT
nad1
atp9
trnD-GTC
trnG-TCC
trnW-CCA
nad3
nad2
atp8
nad6
rps3
cox2*
trnR-TCG
trnV-TAC
rns*
complex I (NADH dehydrogenase)
complex II (succinate dehydrogenase)
complex III (ubichinol cytochrome c reductase)
complex IV (cytochrome c oxidase)
ATP synthase
cytochrome c biogenesis
RNA polymerase
ribosomal proteins (SSU)
ribosomal proteins (LSU)
maturases
other genes
transfer RNAs
ribosomal RNAs
origin of replication
polycistronic transcripts
introns

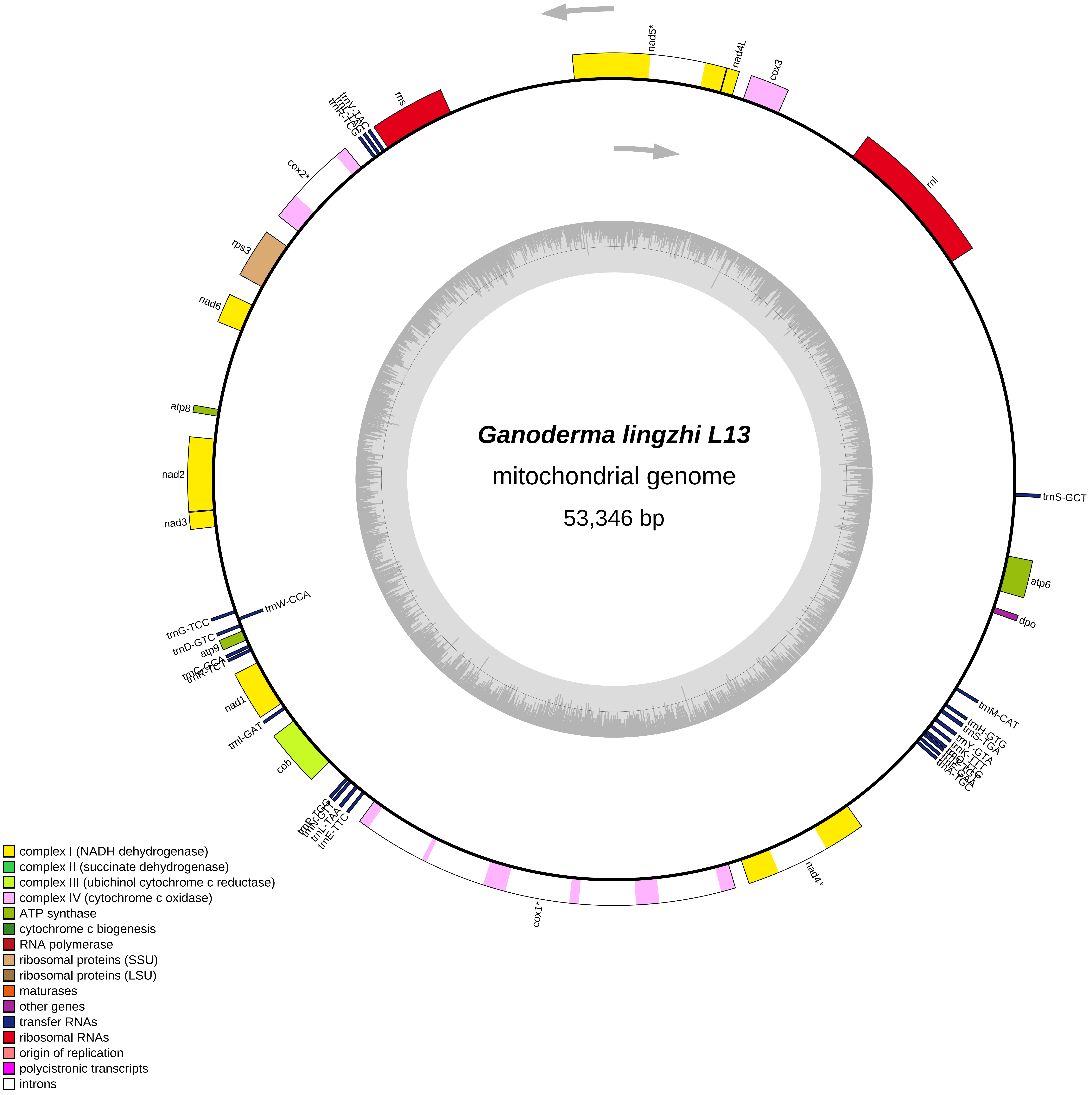
Ganoderma lingzhi L13
mitochondrial genome
53,346 bp
nad5*
nad4L
cox3
rnl
trnS-GCT
atp6
dpo
trnM-CAT
trnH-GTG
trnS-TGA
trnY-GTA
nad4*
cox1*
trnE-TTC
trnL-TAA
cob
trnI-GAT
nad1
trnR-TCT
atp9
trnD-GTC
trnG-TCC
trnW-CCA
nad3
nad2
atp8
nad6
rps3
cox2*
rns
complex I (NADH dehydrogenase)
complex II (succinate dehydrogenase)
complex III (ubichinol cytochrome c reductase)
complex IV (cytochrome c oxidase)
ATP synthase
cytochrome c biogenesis
RNA polymerase
ribosomal proteins (SSU)
ribosomal proteins (LSU)
maturases
other genes
transfer RNAs
ribosomal RNAs
origin of replication
polycistronic transcripts
introns

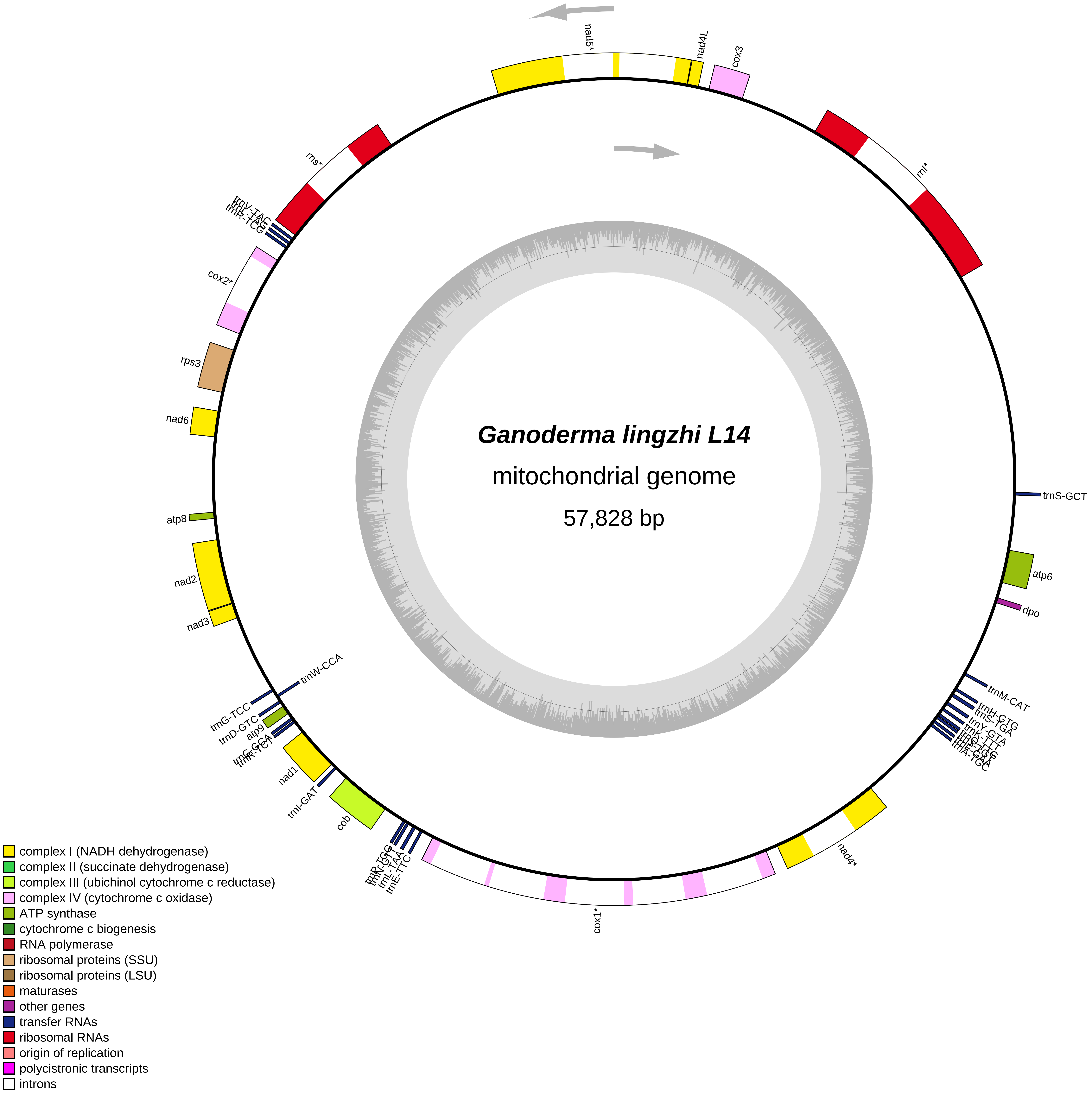

Ganoderma lingzhi L14
mitochondrial genome
57,828 bp
nad5*
nad4L
cox3
rnl*
rns*
trnV-TAC
cox2*
rps3
nad6
atp8
nad2
nad3
trnW-CCA
trnG-TCC
trnD-GTC
atp9
nad1
trnI-GAT
cob
trnL-TAA
trnE-TTC
cox1*
nad4*
trnM-CAT
trnH-GTG
trnS-TGA
trnY-GTA
dpo
atp6
trnS-GCT
complex I (NADH dehydrogenase)
complex II (succinate dehydrogenase)
complex III (ubichinol cytochrome c reductase)
complex IV (cytochrome c oxidase)
ATP synthase
cytochrome c biogenesis
RNA polymerase
ribosomal proteins (SSU)
ribosomal proteins (LSU)
maturases
other genes
transfer RNAs
ribosomal RNAs
origin of replication
polycistronic transcripts
introns

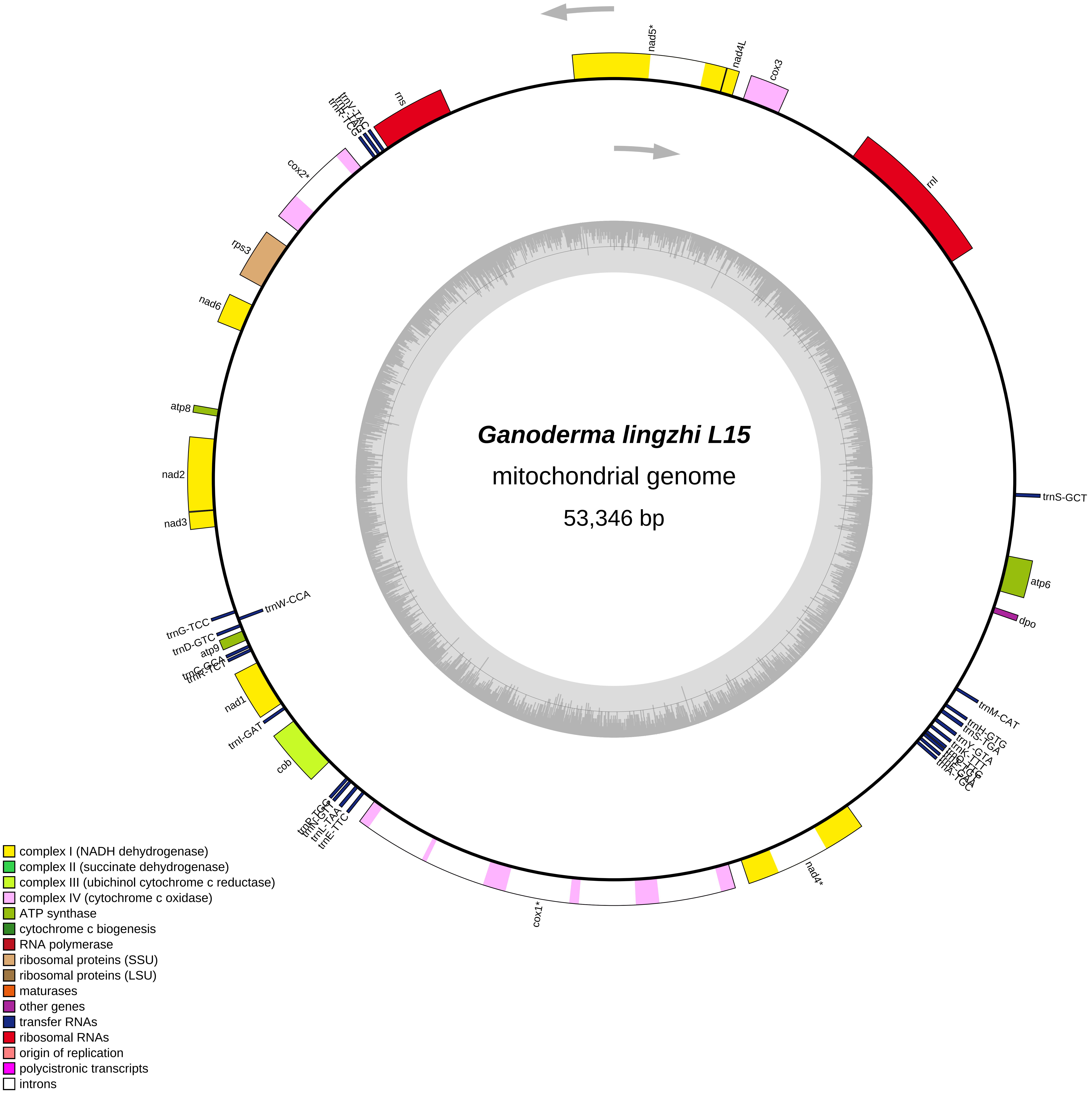
Ganoderma lingzhi L15
mitochondrial genome
53,346 bp
nad5*
nad4L
cox3
rnl
trnS-GCT
atp6
dpo
trnM-CAT
trnH-GTG
trnS-TGA
trnY-GTA
trnK-TTT
nad4*
cox1*
trnE-TTC
trnL-TAA
trnR-TCG
cob
trnI-GAT
nad1
trnR-TCT
atp9
trnD-GTC
trnG-TCC
trnW-CCA
nad3
nad2
atp8
nad6
rps3
cox2*
rns
trnV-TAC
complex I (NADH dehydrogenase)
complex II (succinate dehydrogenase)
complex III (ubichinol cytochrome c reductase)
complex IV (cytochrome c oxidase)
ATP synthase
cytochrome c biogenesis
RNA polymerase
ribosomal proteins (SSU)
ribosomal proteins (LSU)
maturases
other genes
transfer RNAs
ribosomal RNAs
origin of replication
polycistronic transcripts
introns

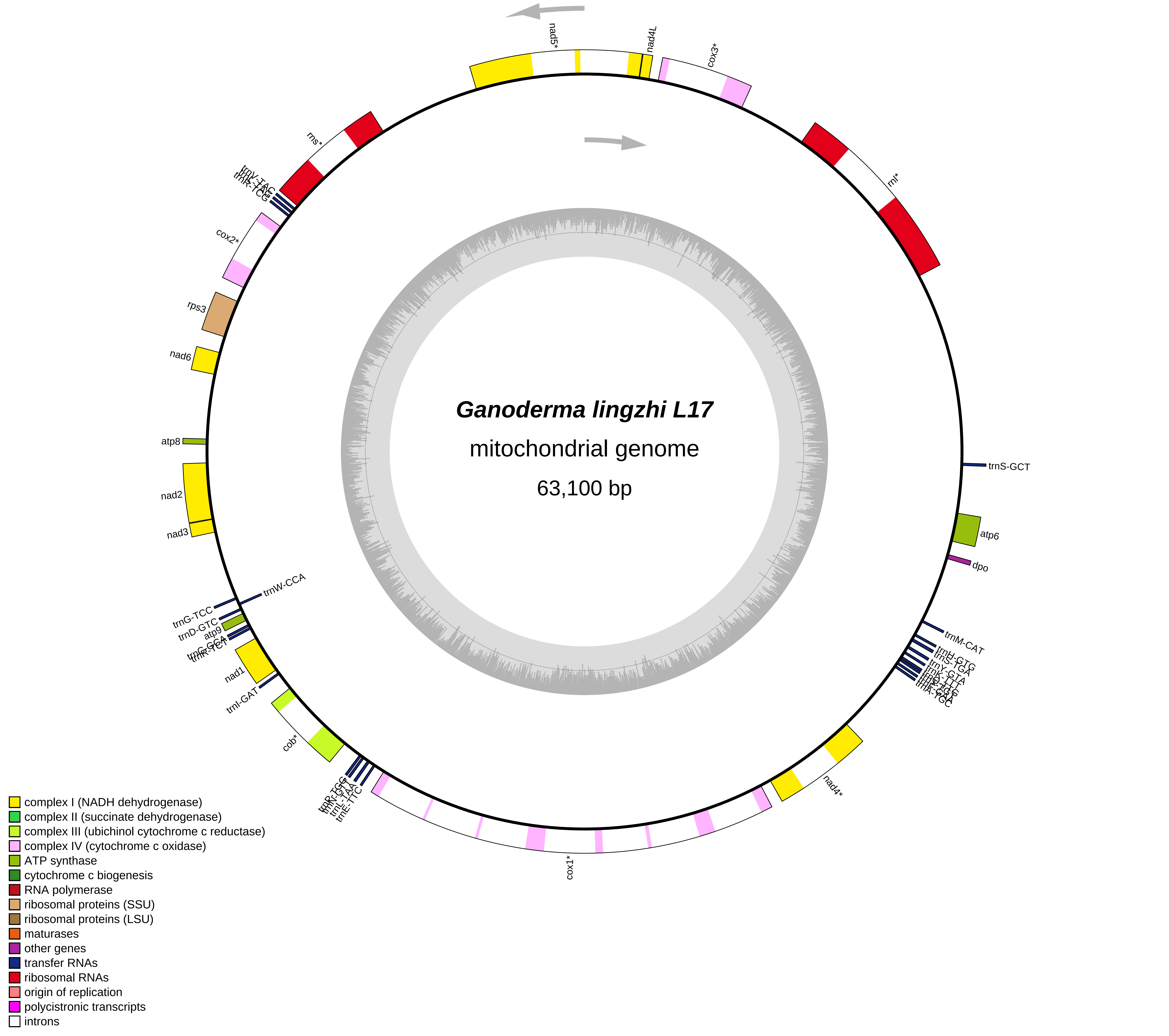
Ganoderma lingzhi L17
mitochondrial genome
63,100 bp
nad5*
nad4L
cox3*
rnl*
trnS-GCT
atp6
dpo
trnM-CAT
trnH-GTG
trnS-TGA
trnY-GTA
nad4*
cox1*
trnE-TTC
trnL-TAA
cob*
trnI-GAT
nad1
atp9
trnD-GTC
trnG-TCC
trnW-CCA
nad3
nad2
atp8
nad6
rps3
cox2*
trnV-TAC
rns*
complex I (NADH dehydrogenase)
complex II (succinate dehydrogenase)
complex III (ubichinol cytochrome c reductase)
complex IV (cytochrome c oxidase)
ATP synthase
cytochrome c biogenesis
RNA polymerase
ribosomal proteins (SSU)
ribosomal proteins (LSU)
maturases
other genes
transfer RNAs
ribosomal RNAs
origin of replication
polycistronic transcripts
introns

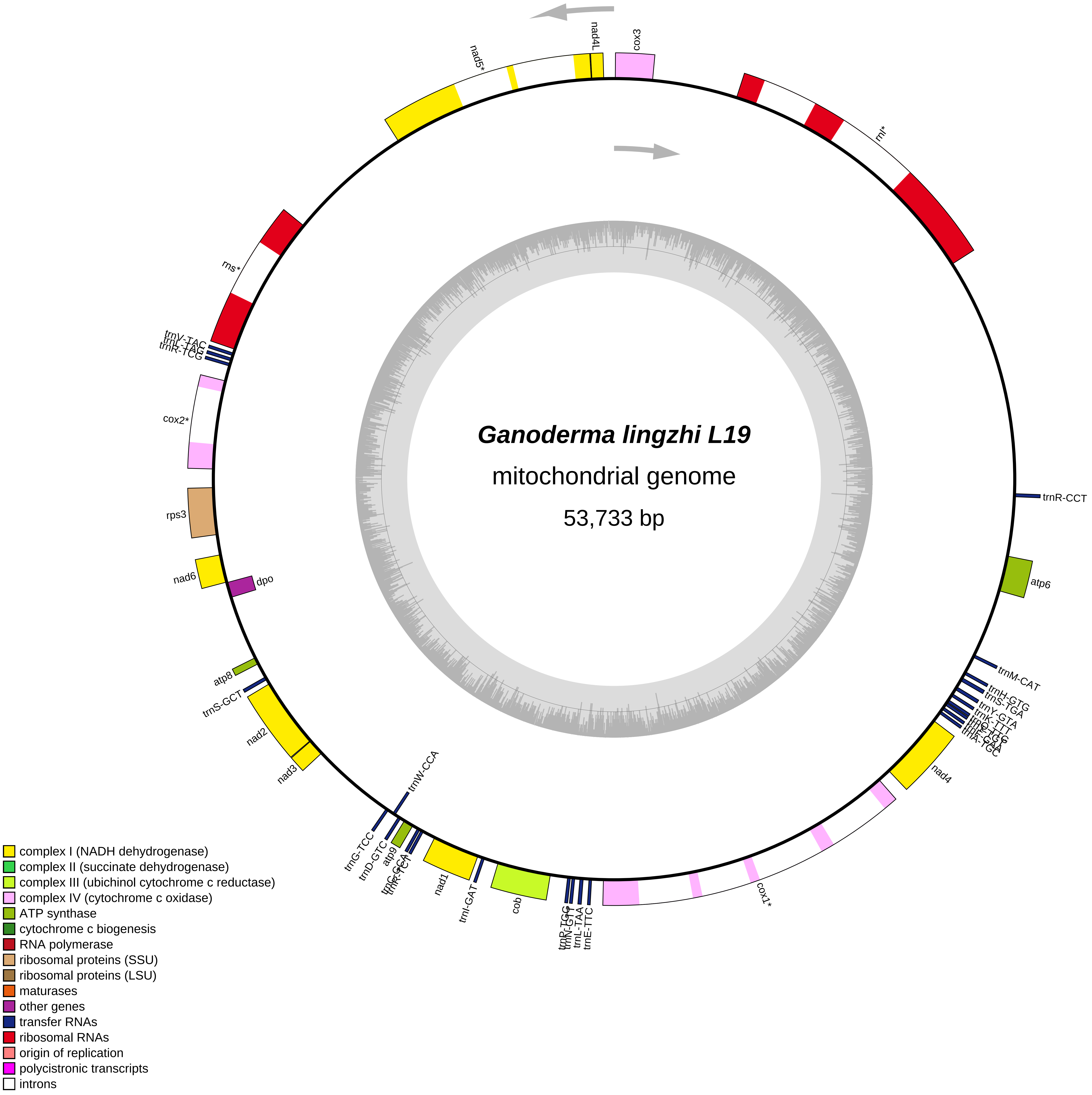

Ganoderma lingzhi L19
mitochondrial genome
53,733 bp
nad5*
nad4L
cox3
rnl*
trnR-CCT
atp6
trnM-CAT
trnH-GTG
trnS-TGA
trnY-GTA
nad4
cox1*
trnE-TTC
trnL-TAA
cob
trnI-GAT
nad1
atp9
trnD-GTC
trnG-TCC
trnW-CCA
nad3
nad2
trnS-GCT
atp8
dpo
nad6
rps3
cox2*
trnV-TAC
trnR-TCG
rns*
complex I (NADH dehydrogenase)
complex II (succinate dehydrogenase)
complex III (ubichinol cytochrome c reductase)
complex IV (cytochrome c oxidase)
ATP synthase
cytochrome c biogenesis
RNA polymerase
ribosomal proteins (SSU)
ribosomal proteins (LSU)
maturases
other genes
transfer RNAs
ribosomal RNAs
origin of replication
polycistronic transcripts
introns

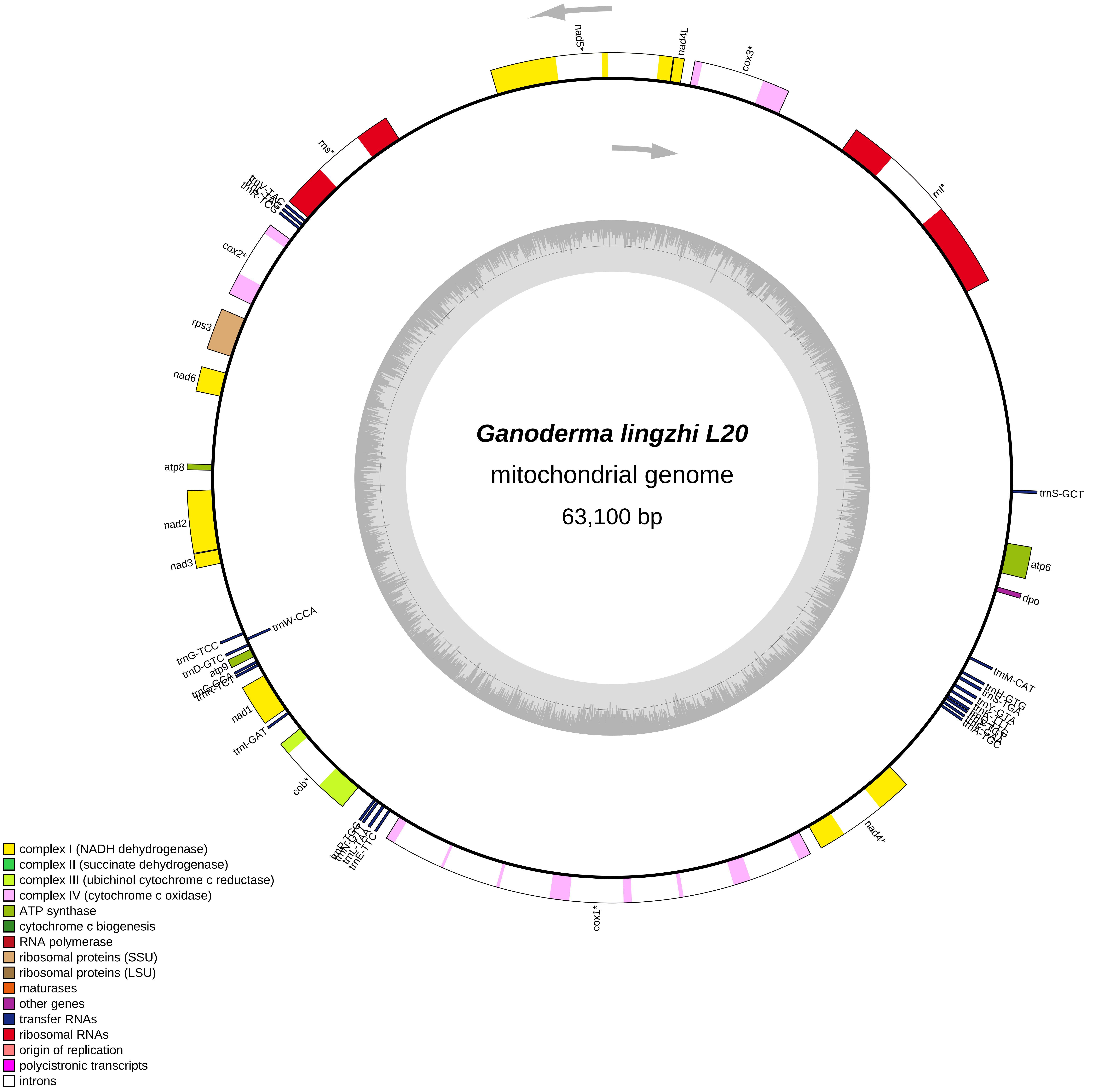

Ganoderma lingzhi L20
mitochondrial genome
63,100 bp
nad5*
nad4L
cox3*
rnl*
trnS-GCT
atp6
dpo
trnM-CAT
trnH-GTG
trnS-TGA
trnY-GTA
nad4*
cox1*
trnE-TTC
cob*
trnI-GAT
nad1
trnR-TCT
atp9
trnD-GTC
trnG-TCC
trnW-CCA
nad3
nad2
atp8
nad6
rps3
cox2*
trnR-TCG
trnV-TAC
rns*
complex I (NADH dehydrogenase)
complex II (succinate dehydrogenase)
complex III (ubichinol cytochrome c reductase)
complex IV (cytochrome c oxidase)
ATP synthase
cytochrome c biogenesis
RNA polymerase
ribosomal proteins (SSU)
ribosomal proteins (LSU)
maturases
other genes
transfer RNAs
ribosomal RNAs
origin of replication
polycistronic transcripts
introns

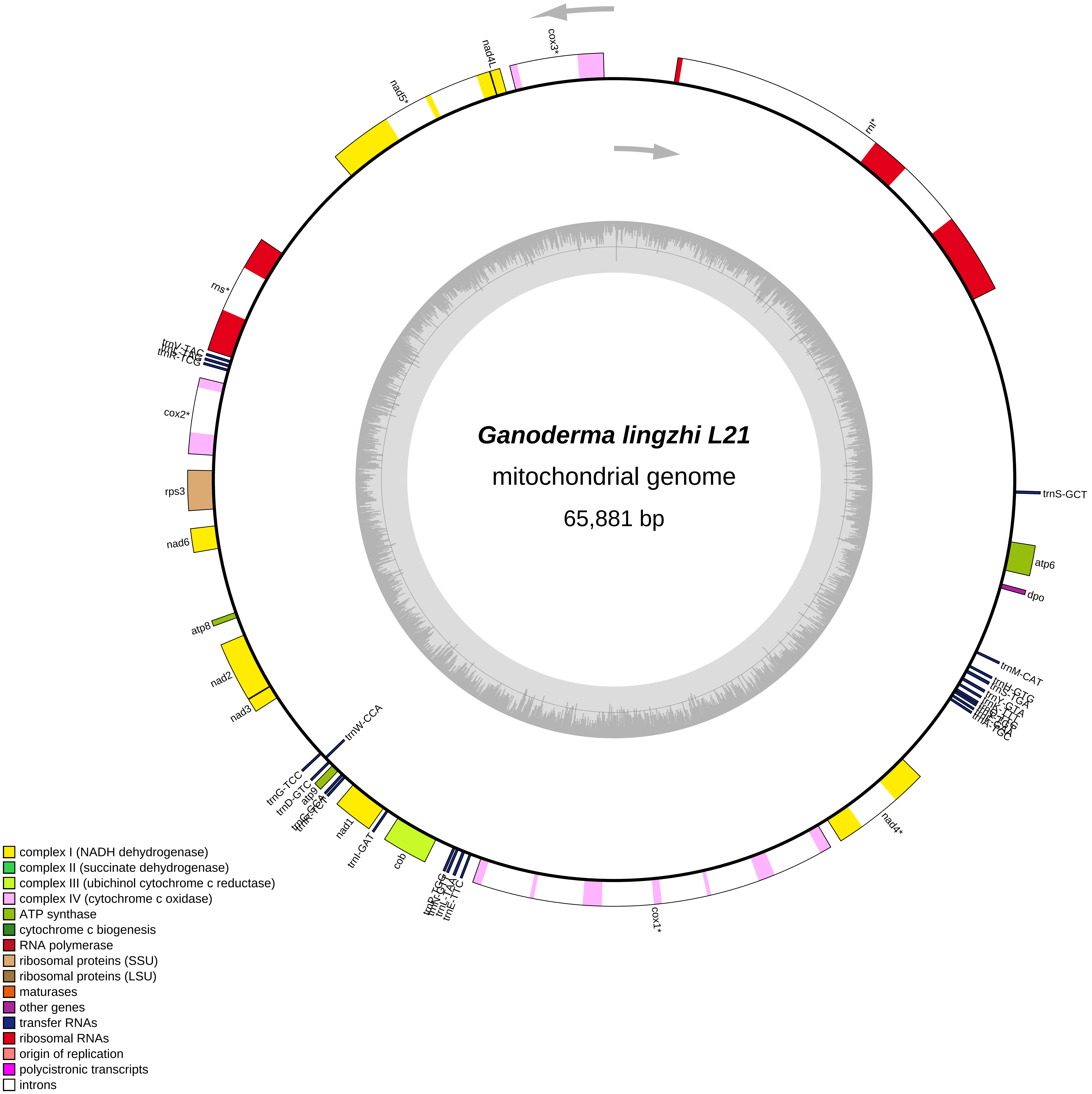
Ganoderma lingzhi L21
mitochondrial genome
65,881 bp
cox3*
nad4L
nad5*
rnl*
rns*
trnV-TAC
trnR-TCG
cox2*
rps3
nad6
atp8
nad2
nad3
trnW-CCA
trnG-TCC
trnD-GTC
atp9
nad1
trnI-GAT
cob
trnE-TTC
cox1*
nad4*
trnM-CAT
trnH-GTG
trnS-TGA
dpo
atp6
trnS-GCT
complex I (NADH dehydrogenase)
complex II (succinate dehydrogenase)
complex III (ubichinol cytochrome c reductase)
complex IV (cytochrome c oxidase)
ATP synthase
cytochrome c biogenesis
RNA polymerase
ribosomal proteins (SSU)
ribosomal proteins (LSU)
maturases
other genes
transfer RNAs
ribosomal RNAs
origin of replication
polycistronic transcripts
introns

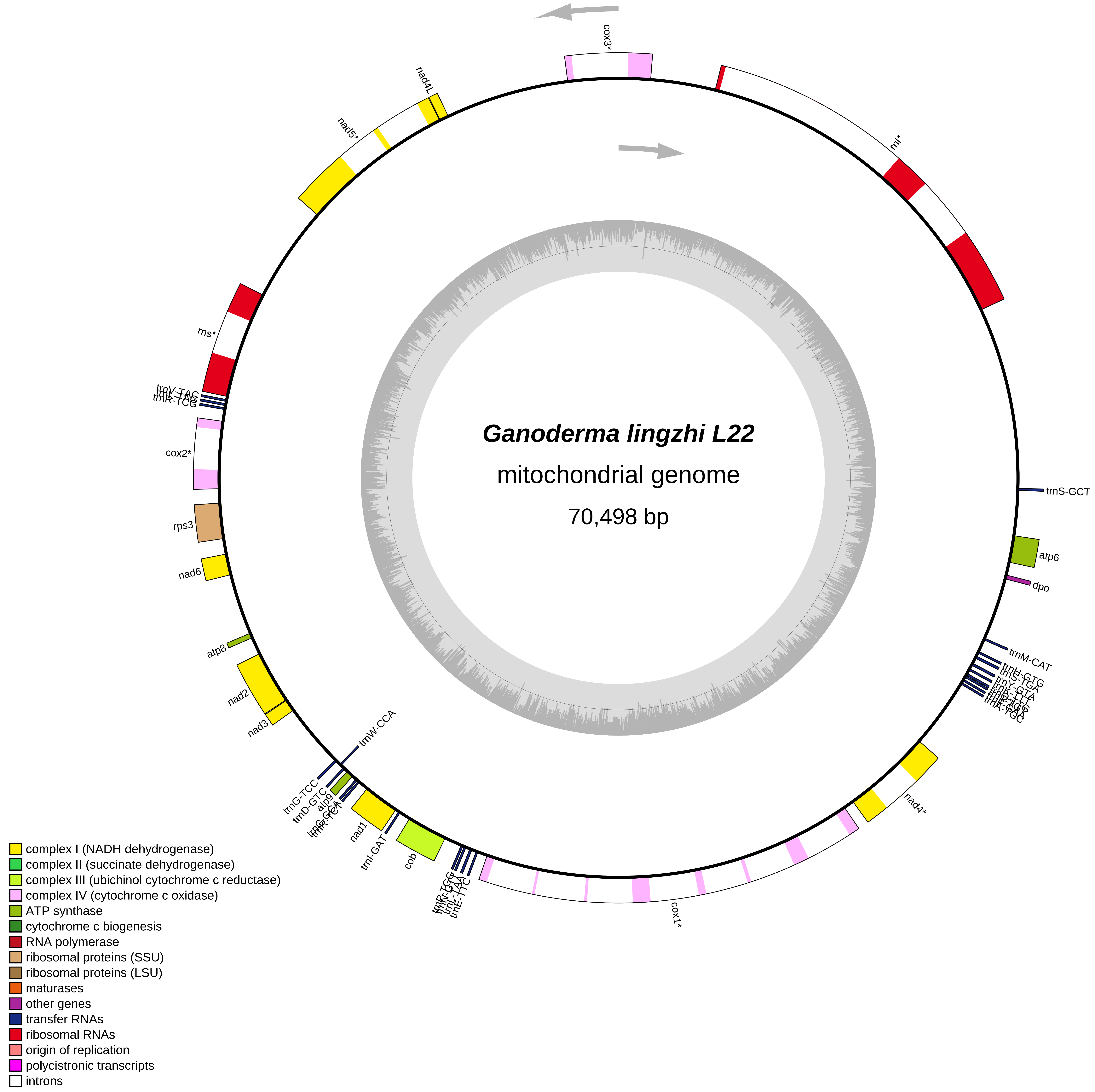

Ganoderma lingzhi L22
mitochondrial genome
70,498 bp
cox3*
rnl*
trnS-GCT
atp6
dpo
trnM-CAT
nad4*
cox1*
cob
trnI-GAT
nad1
trnW-CCA
trnG-TCC
trnD-GTC
atp9
nad3
nad2
atp8
nad6
rps3
cox2*
trnR-TCG
trnV-TAC
rns*
nad5*
nad4L
complex I (NADH dehydrogenase)
complex II (succinate dehydrogenase)
complex III (ubichinol cytochrome c reductase)
complex IV (cytochrome c oxidase)
ATP synthase
cytochrome c biogenesis
RNA polymerase
ribosomal proteins (SSU)
ribosomal proteins (LSU)
maturases
other genes
transfer RNAs
ribosomal RNAs
origin of replication
polycistronic transcripts
introns

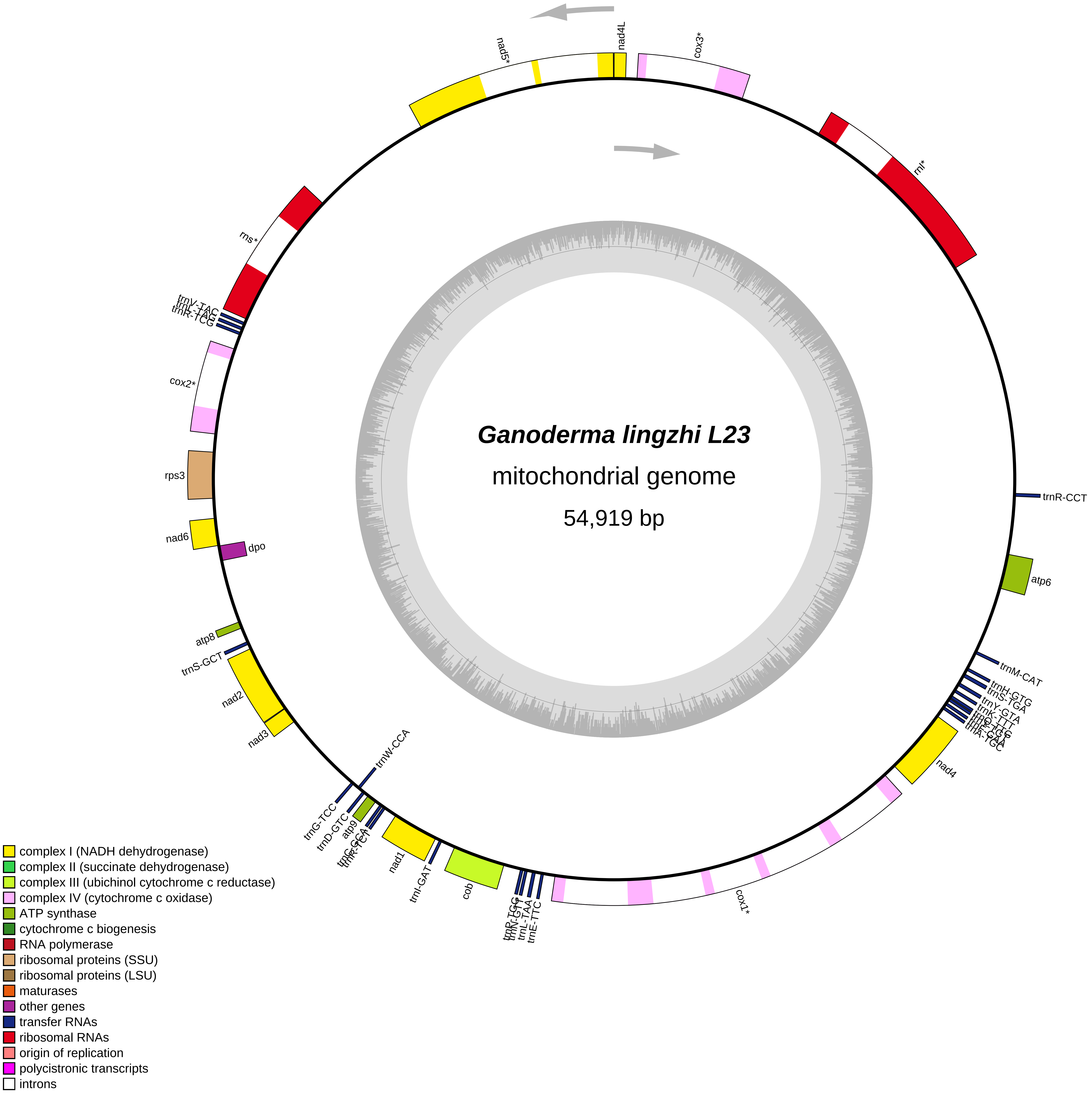
Ganoderma lingzhi L23
mitochondrial genome
54,919 bp
nad5*
nad4L
cox3*
rnl*
trnR-CCT
atp6
trnM-CAT
trnH-GTG
trnS-TGA
trnY-GTA
nad4
cox1*
trnE-TTC
trnL-TAA
cob
trnI-GAT
nad1
atp9
trnD-GTC
trnG-TCC
trnW-CCA
nad3
nad2
trnS-GCT
atp8
dpo
nad6
rps3
cox2*
trnR-TCG
trnV-TAC
rns*
complex I (NADH dehydrogenase)
complex II (succinate dehydrogenase)
complex III (ubichinol cytochrome c reductase)
complex IV (cytochrome c oxidase)
ATP synthase
cytochrome c biogenesis
RNA polymerase
ribosomal proteins (SSU)
ribosomal proteins (LSU)
maturases
other genes
transfer RNAs
ribosomal RNAs
origin of replication
polycistronic transcripts
introns

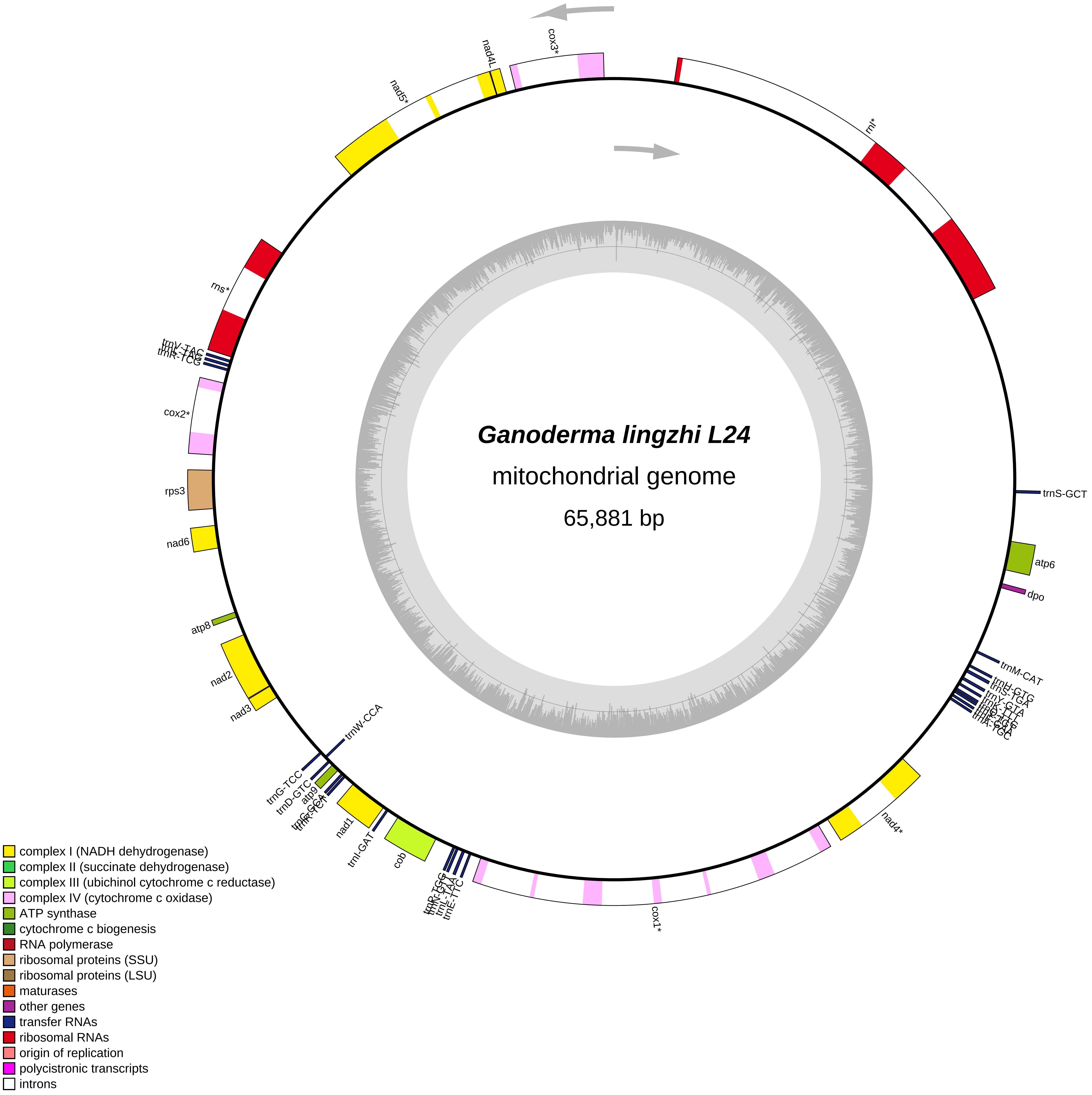

Ganoderma lingzhi L24
mitochondrial genome
65,881 bp
cox3*
nad4L
nad5*
rnl*
rns*
trnR-TCG
cox2*
rps3
nad6
atp8
nad2
nad3
trnW-CCA
trnG-TCC
trnD-GTC
atp9
nad1
trnI-GAT
cob
trnE-TTC
cox1*
nad4*
trnM-CAT
trnH-GTG
trnS-GCT
atp6
dpo
complex I (NADH dehydrogenase)
complex II (succinate dehydrogenase)
complex III (ubichinol cytochrome c reductase)
complex IV (cytochrome c oxidase)
ATP synthase
cytochrome c biogenesis
RNA polymerase
ribosomal proteins (SSU)
ribosomal proteins (LSU)
maturases
other genes
transfer RNAs
ribosomal RNAs
origin of replication
polycistronic transcripts
introns

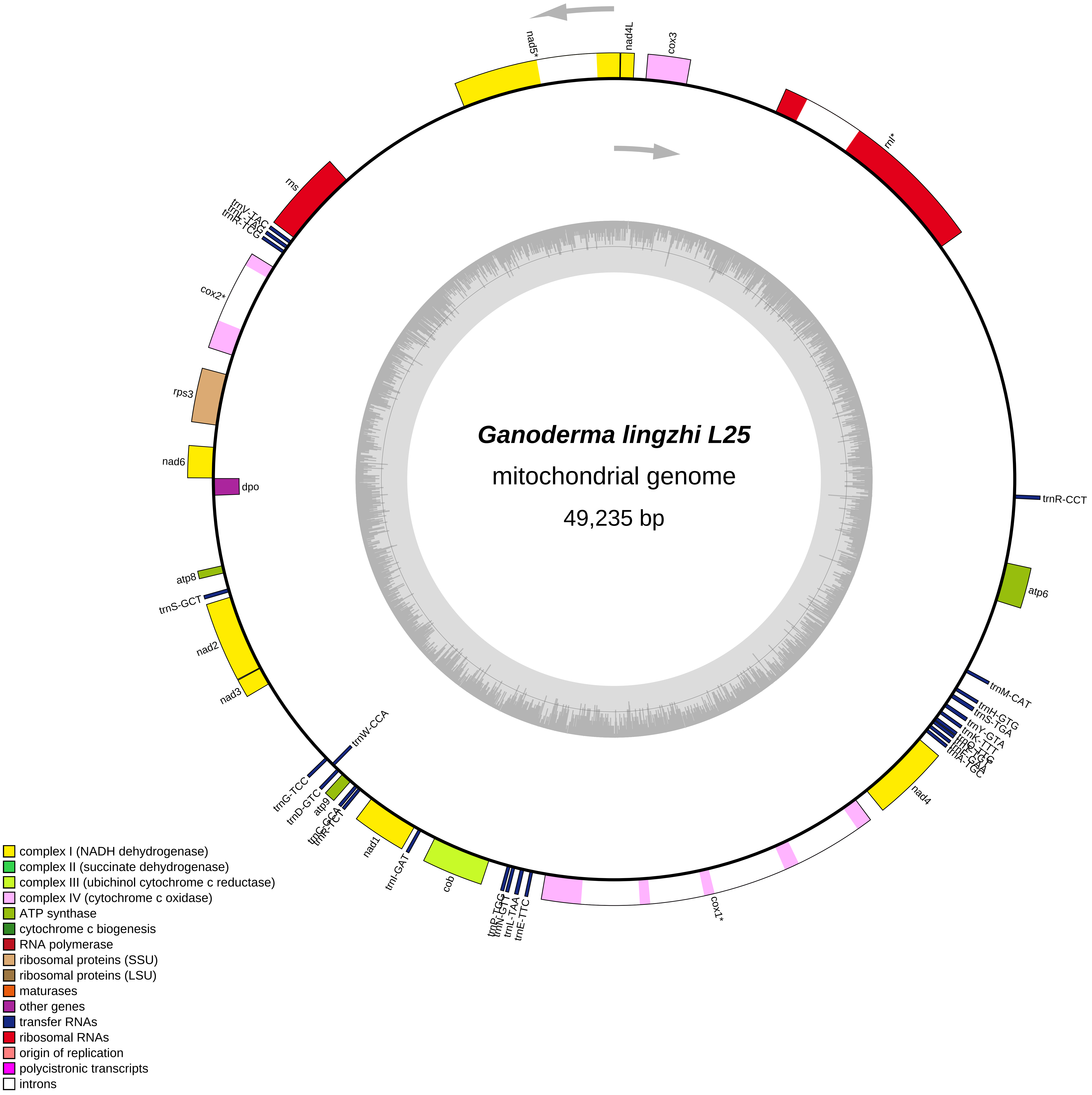
Ganoderma lingzhi L25
mitochondrial genome
49,235 bp
nad5*
nad4L
cox3
rnl*
rns
trnV-TAC
trnL-TAG
trnR-TCG
cox2*
rps3
nad6
dpo
trnR-CCT
atp6
atp8
trnS-GCT
nad2
nad3
trnM-CAT
trnH-GTG
trnS-TGA
trnY-GTA
trnK-TTT
trnA-TGC
nad4
trnW-CCA
trnG-TCC
trnD-GTC
atp9
trnC-GCA
trnR-TCT
nad1
trnI-GAT
cob
trnP-TGG
trnN-GTT
trnL-TAA
trnE-TTC
cox1*
complex I (NADH dehydrogenase)
complex II (succinate dehydrogenase)
complex III (ubichinol cytochrome c reductase)
complex IV (cytochrome c oxidase)
ATP synthase
cytochrome c biogenesis
RNA polymerase
ribosomal proteins (SSU)
ribosomal proteins (LSU)
maturases
other genes
transfer RNAs
ribosomal RNAs
origin of replication
polycistronic transcripts
introns

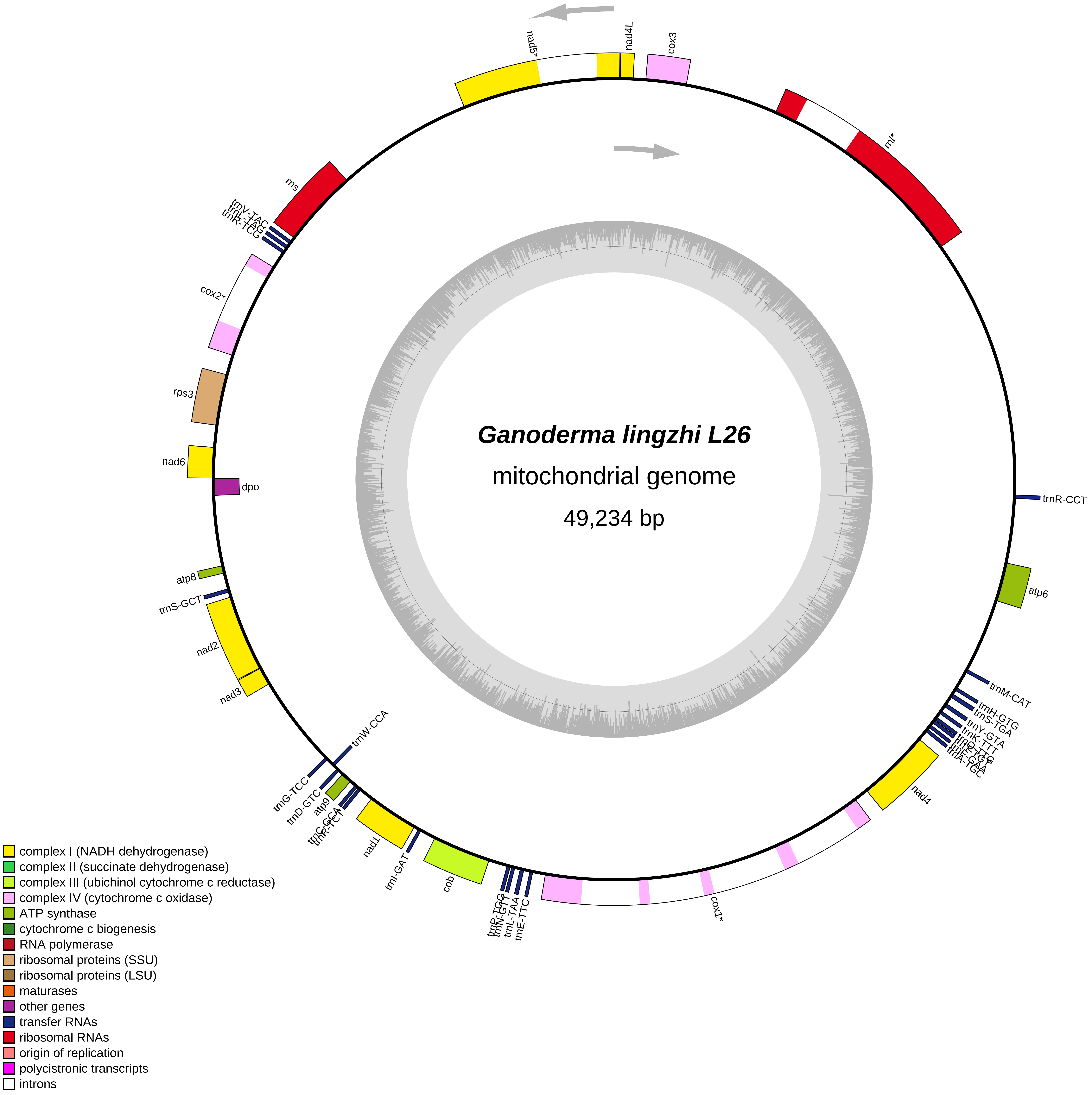

Ganoderma lingzhi L26
mitochondrial genome
49,234 bp
nad5*
nad4L
cox3
rnl*
rns
trnV-TAC
trnL-TAG
trnR-TCG
cox2*
rps3
nad6
dpo
trnR-CCT
atp6
atp8
trnS-GCT
nad2
nad3
trnM-CAT
trnH-GTG
trnS-TGA
trnY-GTA
trnK-TTT
trnA-TGC
nad4
trnW-CCA
trnG-TCC
trnD-GTC
atp9
nad1
trnI-GAT
cob
trnP-TGG
trnN-GTT
trnL-TAA
trnE-TTC
cox1*
complex I (NADH dehydrogenase)
complex II (succinate dehydrogenase)
complex III (ubichinol cytochrome c reductase)
complex IV (cytochrome c oxidase)
ATP synthase
cytochrome c biogenesis
RNA polymerase
ribosomal proteins (SSU)
ribosomal proteins (LSU)
maturases
other genes
transfer RNAs
ribosomal RNAs
origin of replication
polycistronic transcripts
introns

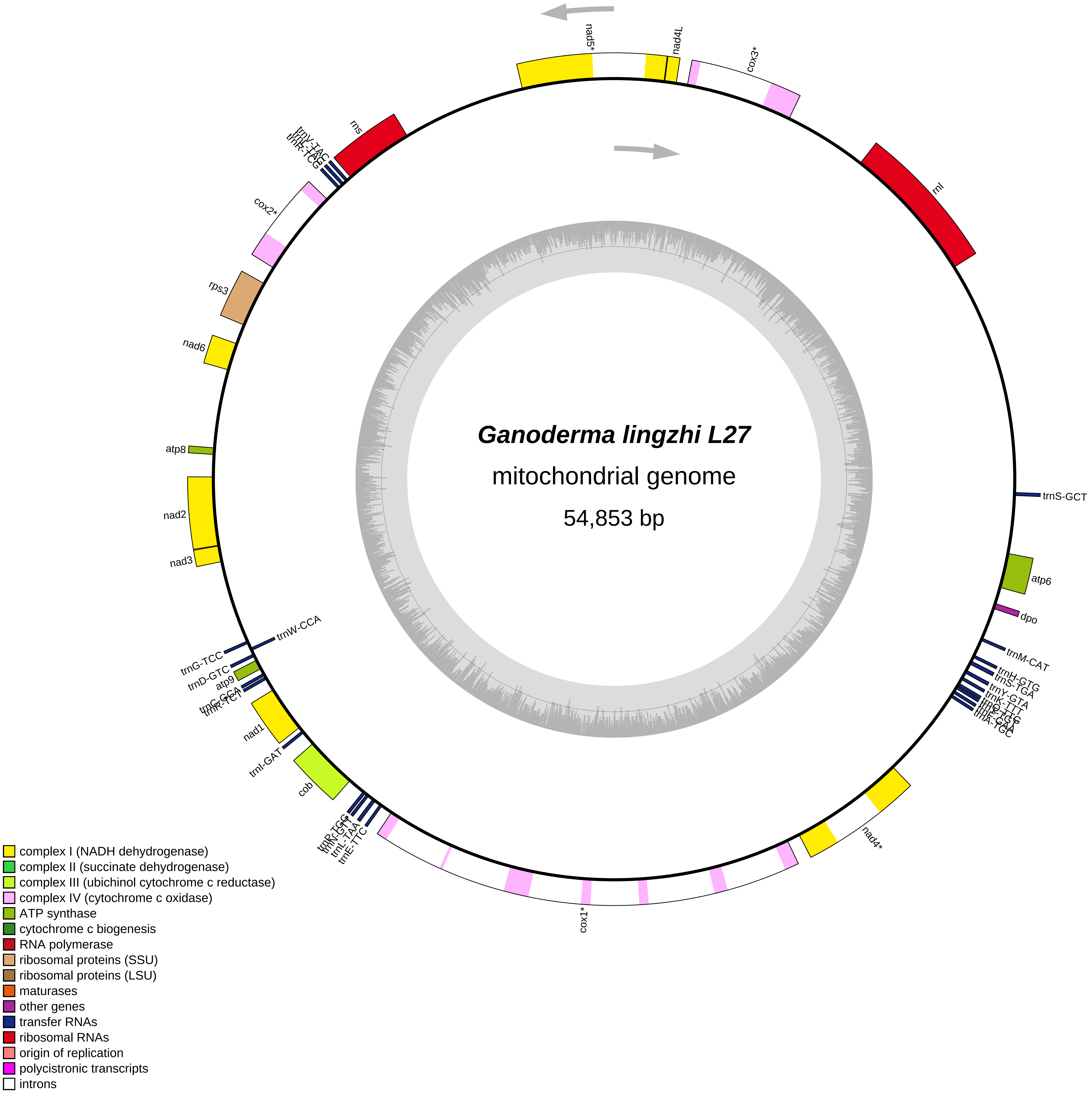
Ganoderma lingzhi L27
mitochondrial genome
54,853 bp
nad5*
nad4L
cox3*
rnl
trnS-GCT
atp6
dpo
trnM-CAT
trnH-GTG
trnS-TGA
trnY-GTA
nad4*
cox1*
trnE-TTC
trnL-TAA
cob
trnI-GAT
nad1
trnR-TCT
trnC-GCA
atp9
trnD-GTC
trnG-TCC
trnW-CCA
nad3
nad2
atp8
nad6
rps3
cox2*
trnV-TAC
trnR-TCG
rns
complex I (NADH dehydrogenase)
complex II (succinate dehydrogenase)
complex III (ubichinol cytochrome c reductase)
complex IV (cytochrome c oxidase)
ATP synthase
cytochrome c biogenesis
RNA polymerase
ribosomal proteins (SSU)
ribosomal proteins (LSU)
maturases
other genes
transfer RNAs
ribosomal RNAs
origin of replication
polycistronic transcripts
introns

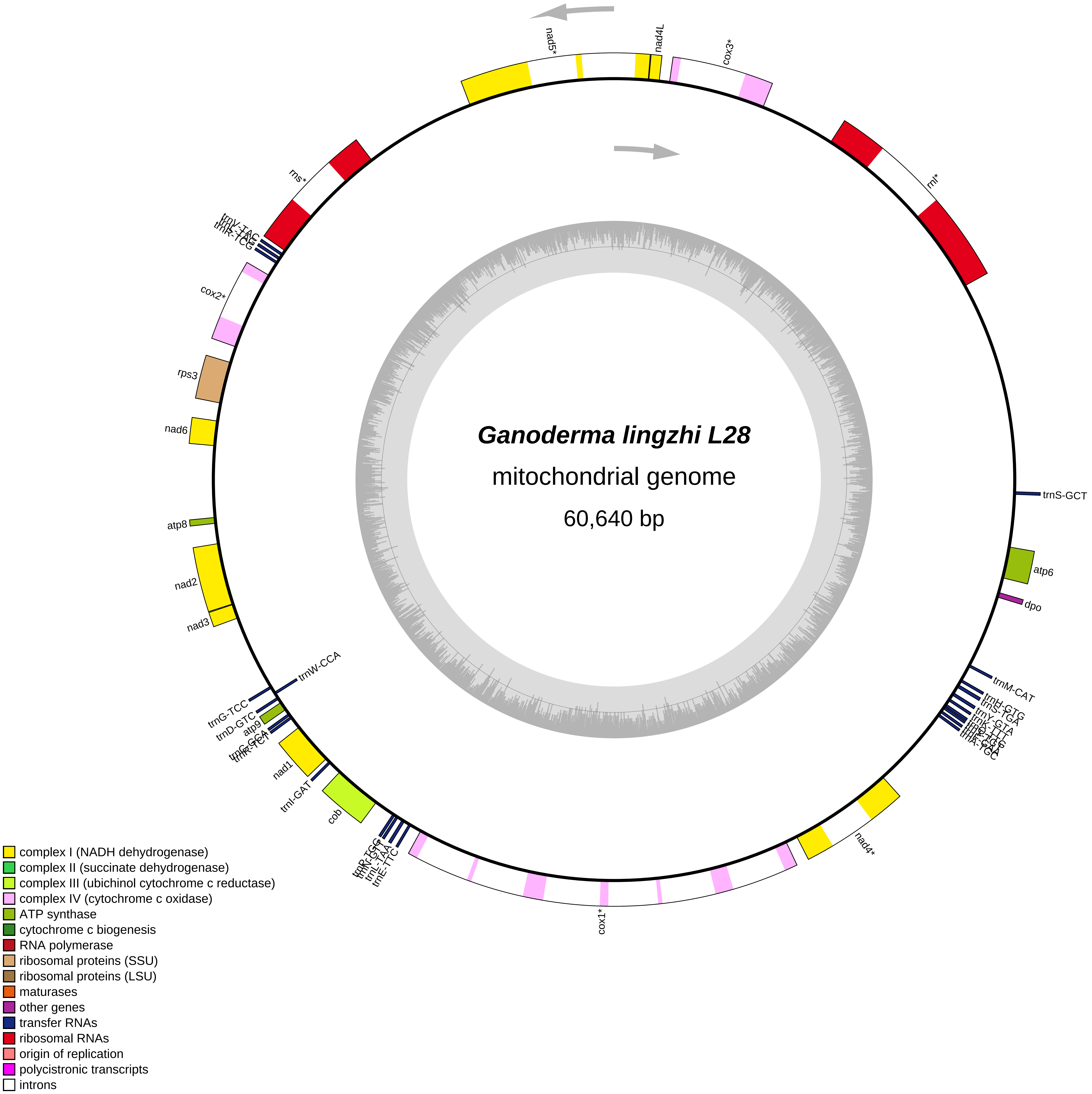
Ganoderma lingzhi L28
mitochondrial genome
60,640 bp
nad5*
nad4L
cox3*
rnl*
rns*
trnV-TAC
trnR-TCG
cox2*
rps3
nad6
trnS-GCT
atp8
atp6
dpo
nad2
nad3
trnW-CCA
trnM-CAT
trnH-GTG
trnG-TCC
trnD-GTC
atp9
nad1
trnI-GAT
cob
nad4*
trnE-TTC
cox1*
complex I (NADH dehydrogenase)
complex II (succinate dehydrogenase)
complex III (ubichinol cytochrome c reductase)
complex IV (cytochrome c oxidase)
ATP synthase
cytochrome c biogenesis
RNA polymerase
ribosomal proteins (SSU)
ribosomal proteins (LSU)
maturases
other genes
transfer RNAs
ribosomal RNAs
origin of replication
polycistronic transcripts
introns

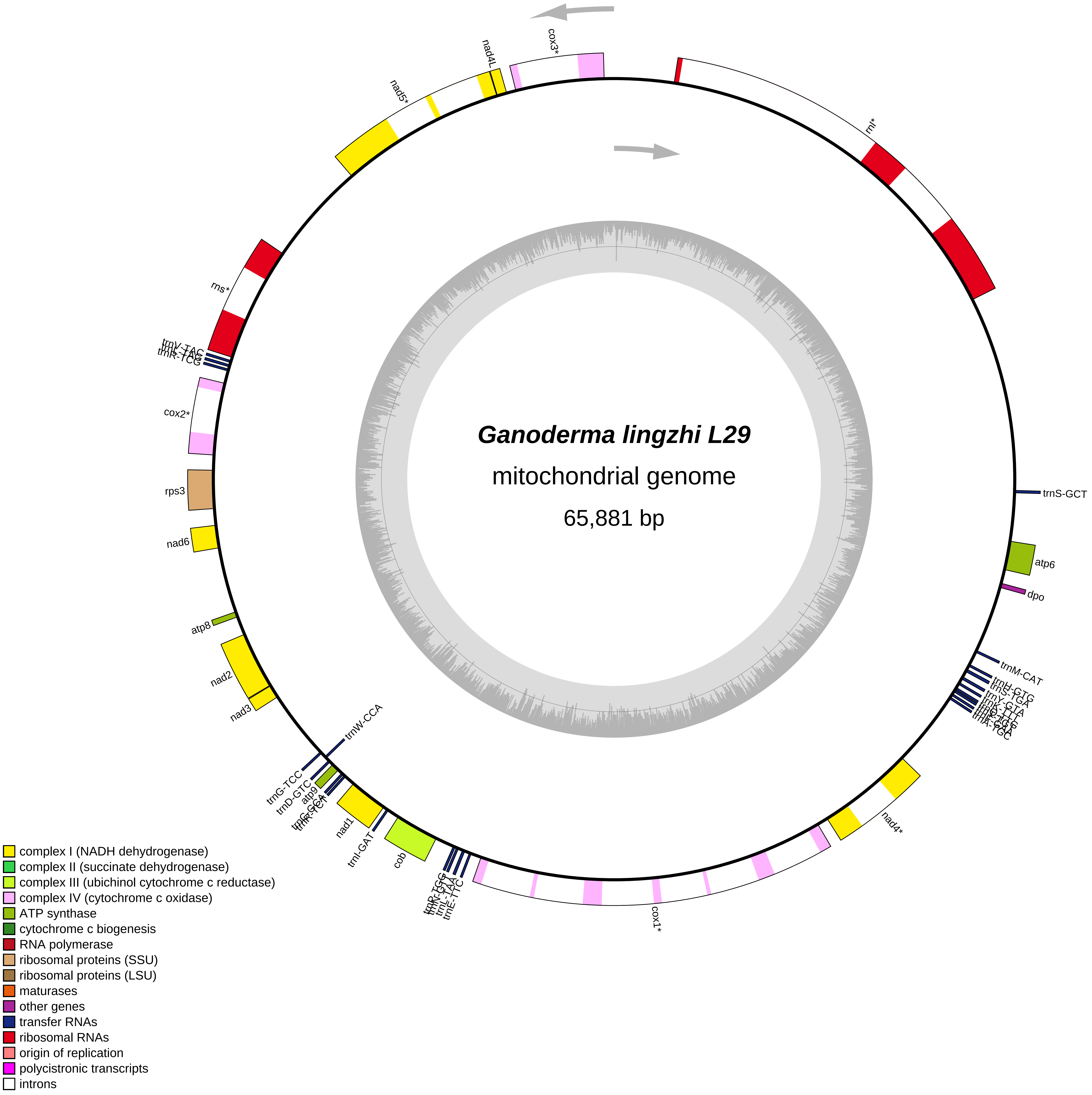

Ganoderma lingzhi L29
mitochondrial genome
65,881 bp
cox3*
nad4L
nad5*
rnl*
rns*
trnV-TAC
trnR-TCG
cox2*
rps3
nad6
atp8
nad2
nad3
trnW-CCA
trnG-TCC
trnD-GTC
atp9
nad1
trnI-GAT
cob
trnE-TTC
cox1*
nad4*
trnM-CAT
trnH-GTG
trnS-TGA
trnA-TGC
dpo
atp6
trnS-GCT
complex I (NADH dehydrogenase)
complex II (succinate dehydrogenase)
complex III (ubichinol cytochrome c reductase)
complex IV (cytochrome c oxidase)
ATP synthase
cytochrome c biogenesis
RNA polymerase
ribosomal proteins (SSU)
ribosomal proteins (LSU)
maturases
other genes
transfer RNAs
ribosomal RNAs
origin of replication
polycistronic transcripts
introns

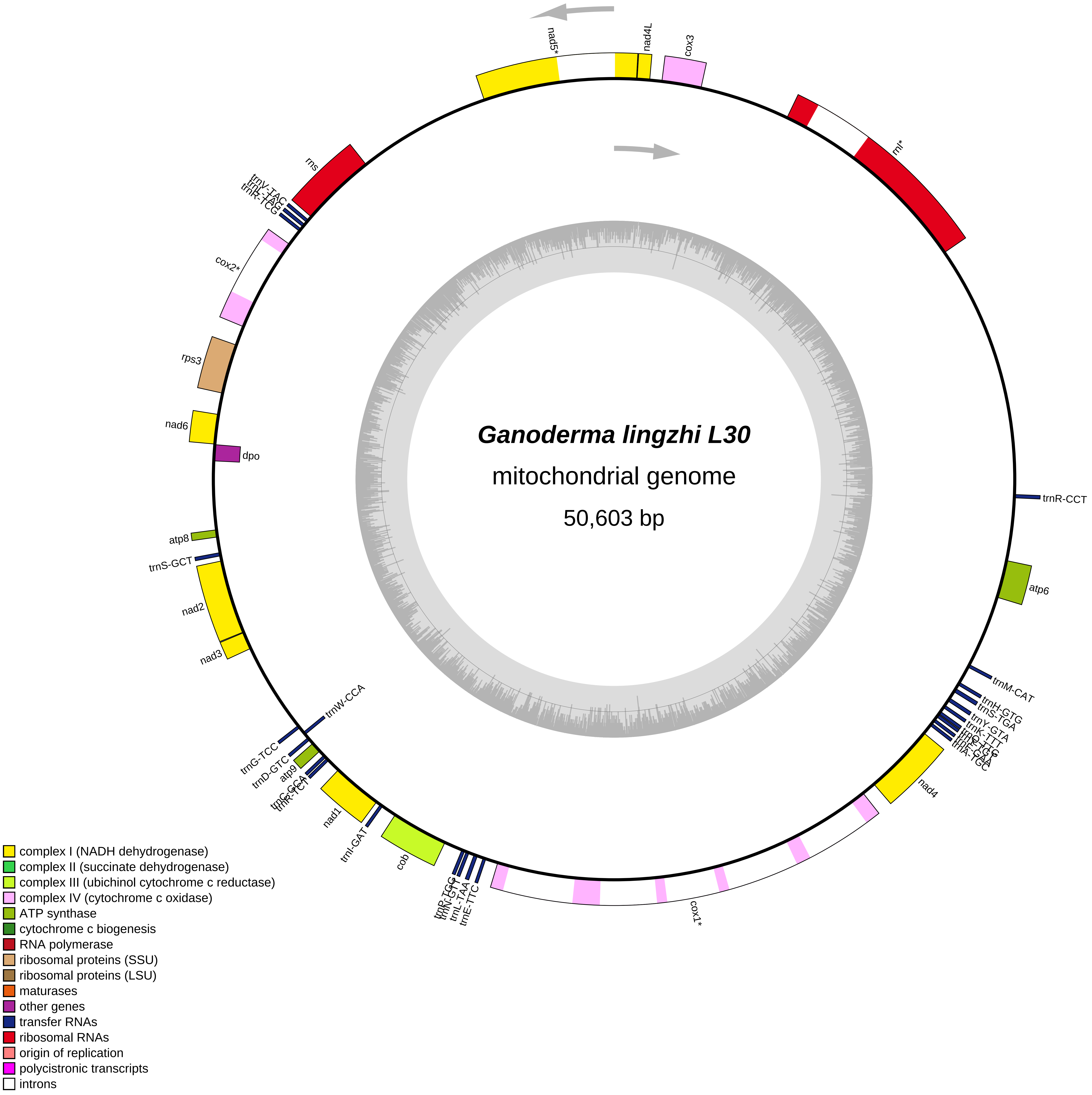
Ganoderma lingzhi L30
mitochondrial genome
50,603 bp
nad5*
nad4L
cox3
rnl*
trnR-CCT
atp6
trnM-CAT
trnH-GTG
trnS-TGA
trnY-GTA
trnK-TTT
nad4
cox1*
trnE-TTC
trnL-TAA
cob
trnI-GAT
nad1
atp9
trnD-GTC
trnG-TCC
trnW-CCA
nad3
nad2
trnS-GCT
atp8
dpo
nad6
rps3
cox2*
trnR-TCG
trnV-TAC
rns
complex I (NADH dehydrogenase)
complex II (succinate dehydrogenase)
complex III (ubichinol cytochrome c reductase)
complex IV (cytochrome c oxidase)
ATP synthase
cytochrome c biogenesis
RNA polymerase
ribosomal proteins (SSU)
ribosomal proteins (LSU)
maturases
other genes
transfer RNAs
ribosomal RNAs
origin of replication
polycistronic transcripts
introns

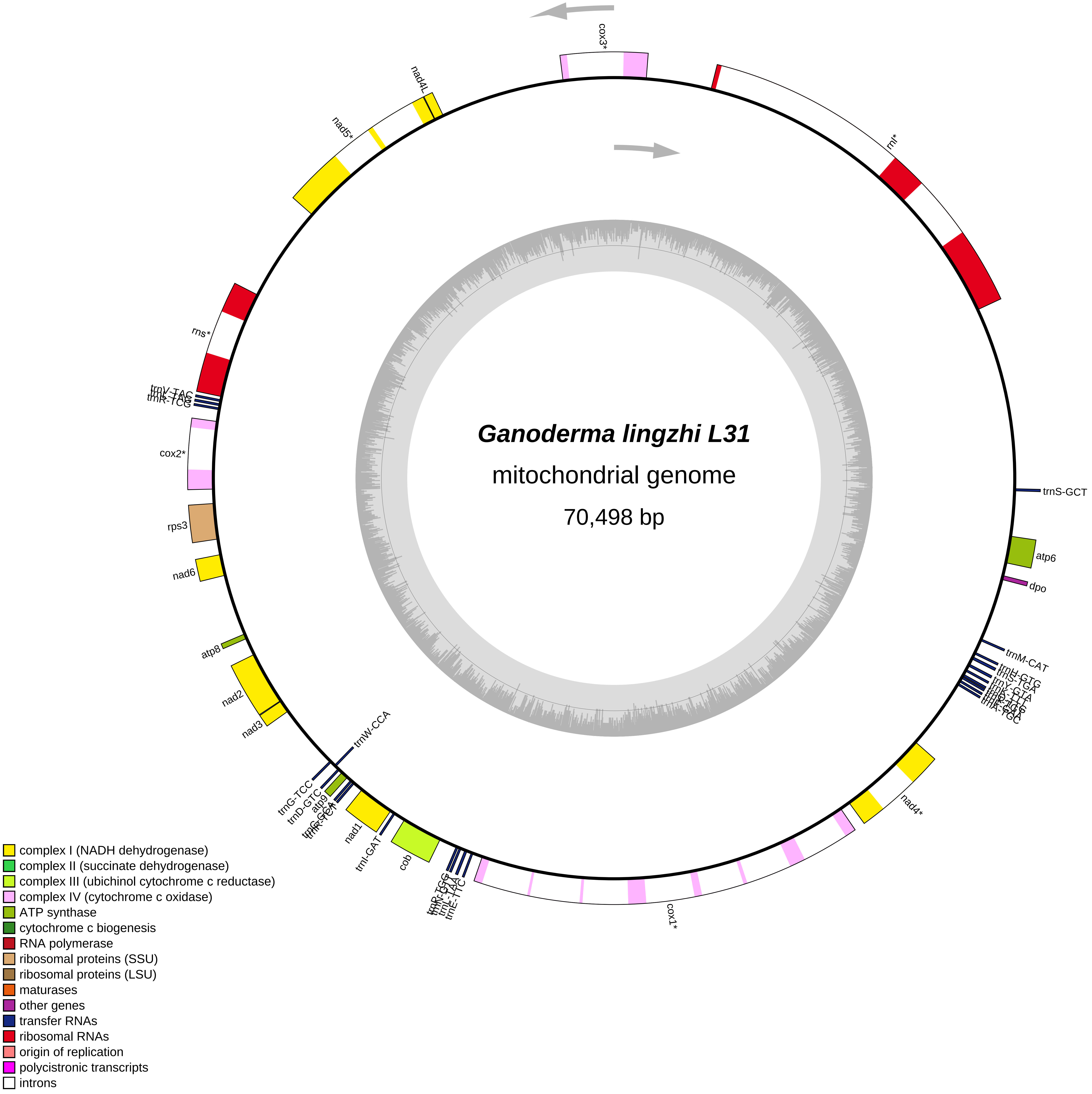
Ganoderma lingzhi L31
mitochondrial genome
70,498 bp
cox3*
rnl*
trnS-GCT
atp6
dpo
trnM-CAT
nad4*
cox1*
trnE-TTC
cob
trnI-GAT
nad1
atp9
trnD-GTC
trnG-TCC
trnW-CCA
nad3
nad2
atp8
nad6
rps3
cox2*
trnR-TCG
trnV-TAC
rns*
nad5*
nad4L
complex I (NADH dehydrogenase)
complex II (succinate dehydrogenase)
complex III (ubichinol cytochrome c reductase)
complex IV (cytochrome c oxidase)
ATP synthase
cytochrome c biogenesis
RNA polymerase
ribosomal proteins (SSU)
ribosomal proteins (LSU)
maturases
other genes
transfer RNAs
ribosomal RNAs
origin of replication
polycistronic transcripts
introns

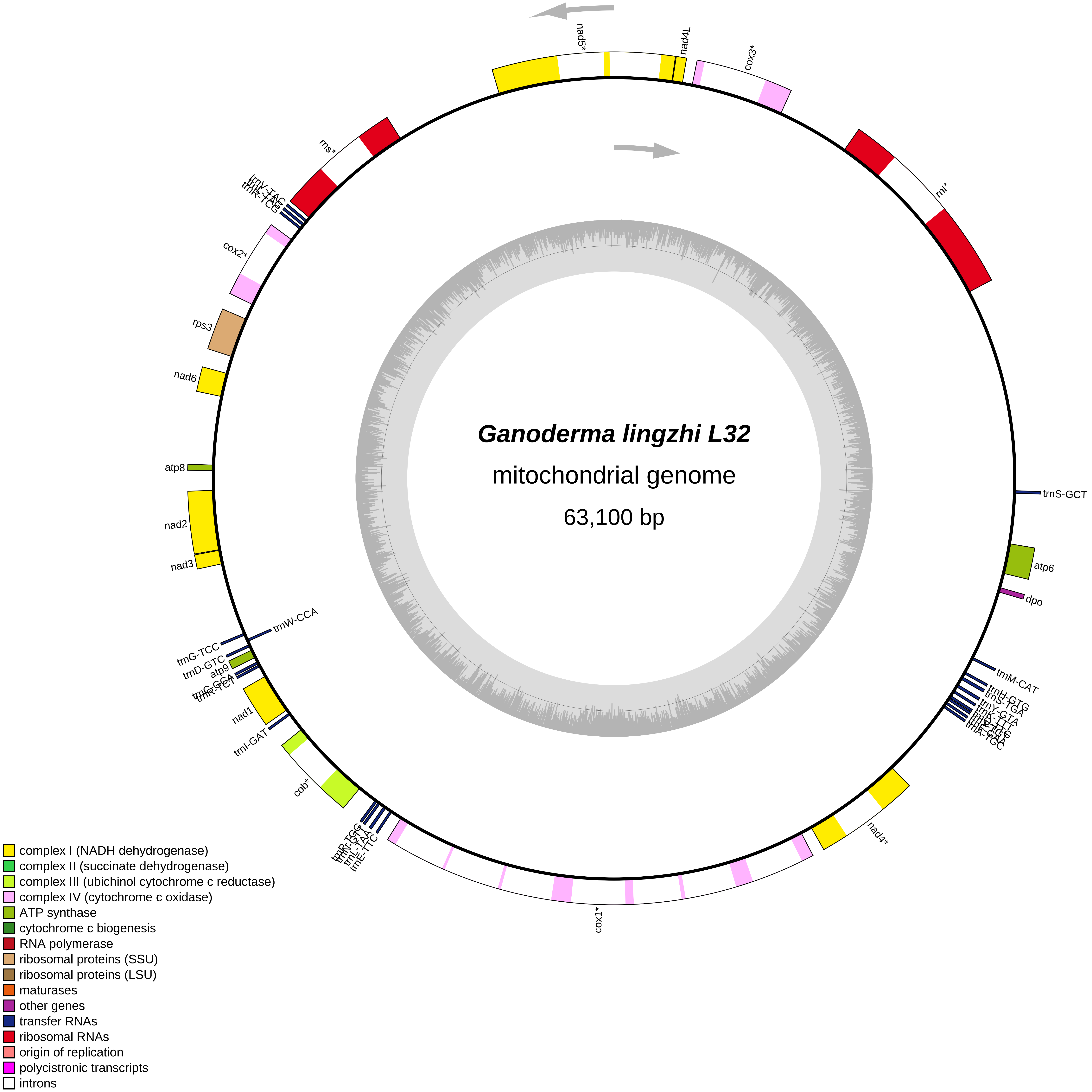

Ganoderma lingzhi L32
mitochondrial genome
63,100 bp
nad5*
nad4L
cox3*
rnl*
trnS-GCT
atp6
dpo
trnM-CAT
trnH-GTG
trnS-TGA
nad4*
cox1*
trnE-TTC
cob*
trnI-GAT
nad1
atp9
trnD-GTC
trnG-TCC
trnW-CCA
nad3
nad2
atp8
nad6
rps3
cox2*
trnV-TAC
rns*
complex I (NADH dehydrogenase)
complex II (succinate dehydrogenase)
complex III (ubichinol cytochrome c reductase)
complex IV (cytochrome c oxidase)
ATP synthase
cytochrome c biogenesis
RNA polymerase
ribosomal proteins (SSU)
ribosomal proteins (LSU)
maturases
other genes
transfer RNAs
ribosomal RNAs
origin of replication
polycistronic transcripts
introns

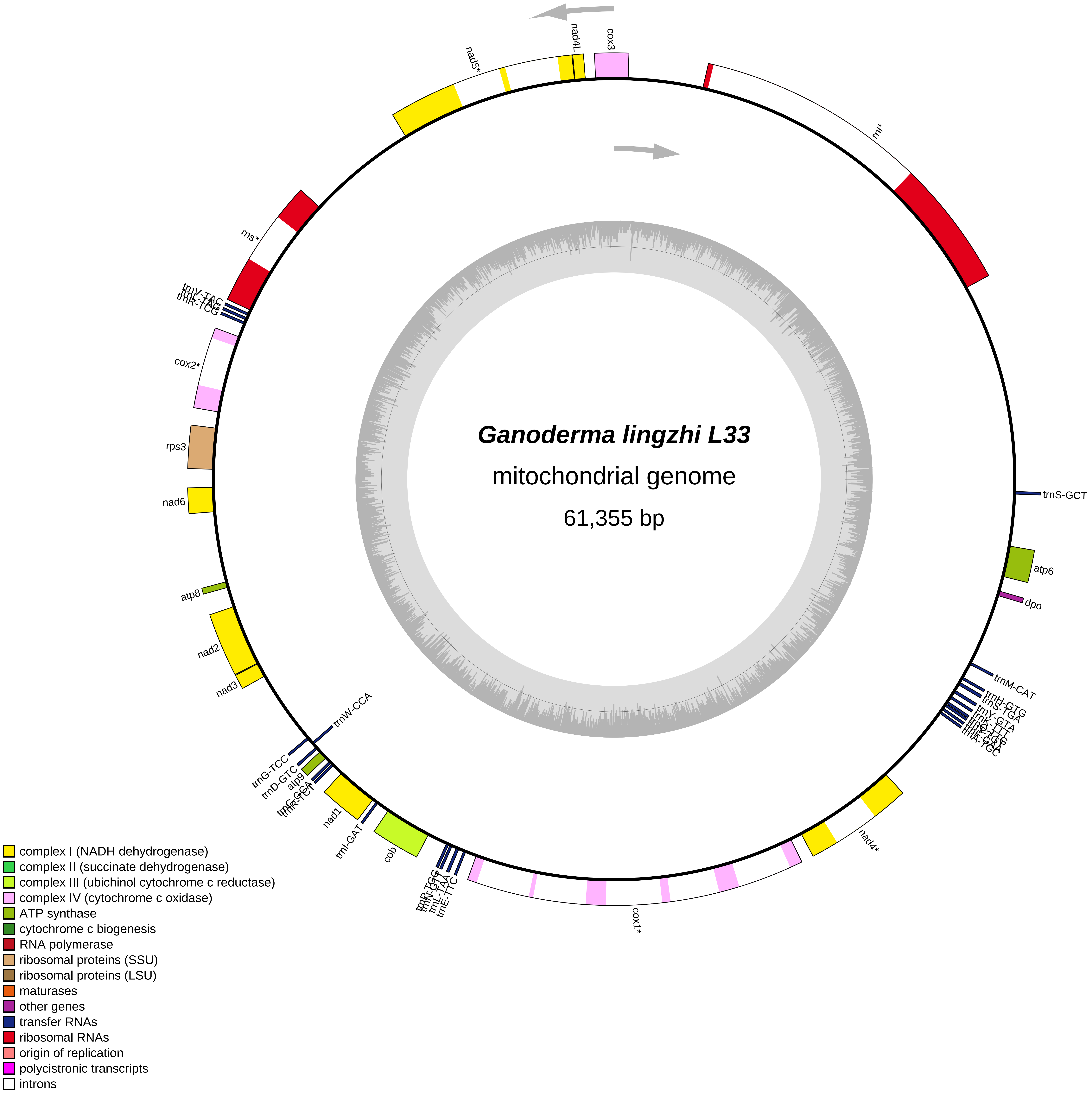
Ganoderma lingzhi L33
mitochondrial genome
61,355 bp
nad5*
nad4L
cox3
rnl*
trnS-GCT
atp6
dpo
trnM-CAT
trnH-GTG
trnS-TGA
nad4*
cox1*
trnE-TTC
trnL-TAA
cob
trnI-GAT
nad1
atp9
trnD-GTC
trnG-TCC
trnW-CCA
nad3
nad2
atp8
nad6
rps3
cox2*
trnR-TCG
trnV-TAC
rns*
complex I (NADH dehydrogenase)
complex II (succinate dehydrogenase)
complex III (ubichinol cytochrome c reductase)
complex IV (cytochrome c oxidase)
ATP synthase
cytochrome c biogenesis
RNA polymerase
ribosomal proteins (SSU)
ribosomal proteins (LSU)
maturases
other genes
transfer RNAs
ribosomal RNAs
origin of replication
polycistronic transcripts
introns

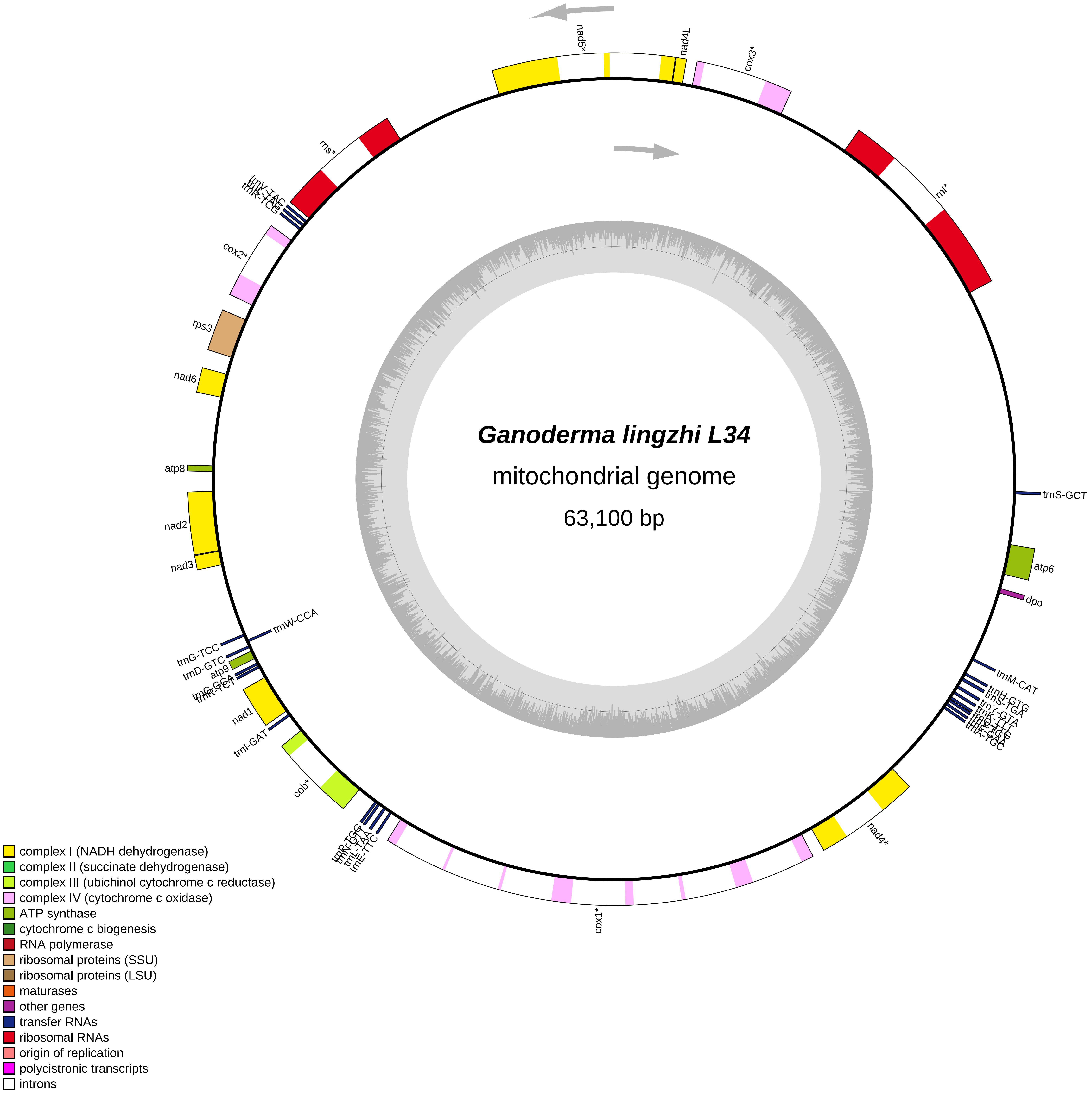

Ganoderma lingzhi L34
mitochondrial genome
63,100 bp
nad5*
nad4L
cox3*
rnl*
trnS-GCT
atp6
dpo
trnM-CAT
trnH-GTG
trnS-TGA
trnY-GTA
nad4*
cox1*
trnE-TTC
cob*
trnI-GAT
nad1
trnR-TCT
trnC-GCA
atp9
trnD-GTC
trnG-TCC
trnW-CCA
nad3
nad2
atp8
nad6
rps3
cox2*
trnR-TCG
trnV-TAC
rns*
complex I (NADH dehydrogenase)
complex II (succinate dehydrogenase)
complex III (ubichinol cytochrome c reductase)
complex IV (cytochrome c oxidase)
ATP synthase
cytochrome c biogenesis
RNA polymerase
ribosomal proteins (SSU)
ribosomal proteins (LSU)
maturases
other genes
transfer RNAs
ribosomal RNAs
origin of replication
polycistronic transcripts
introns

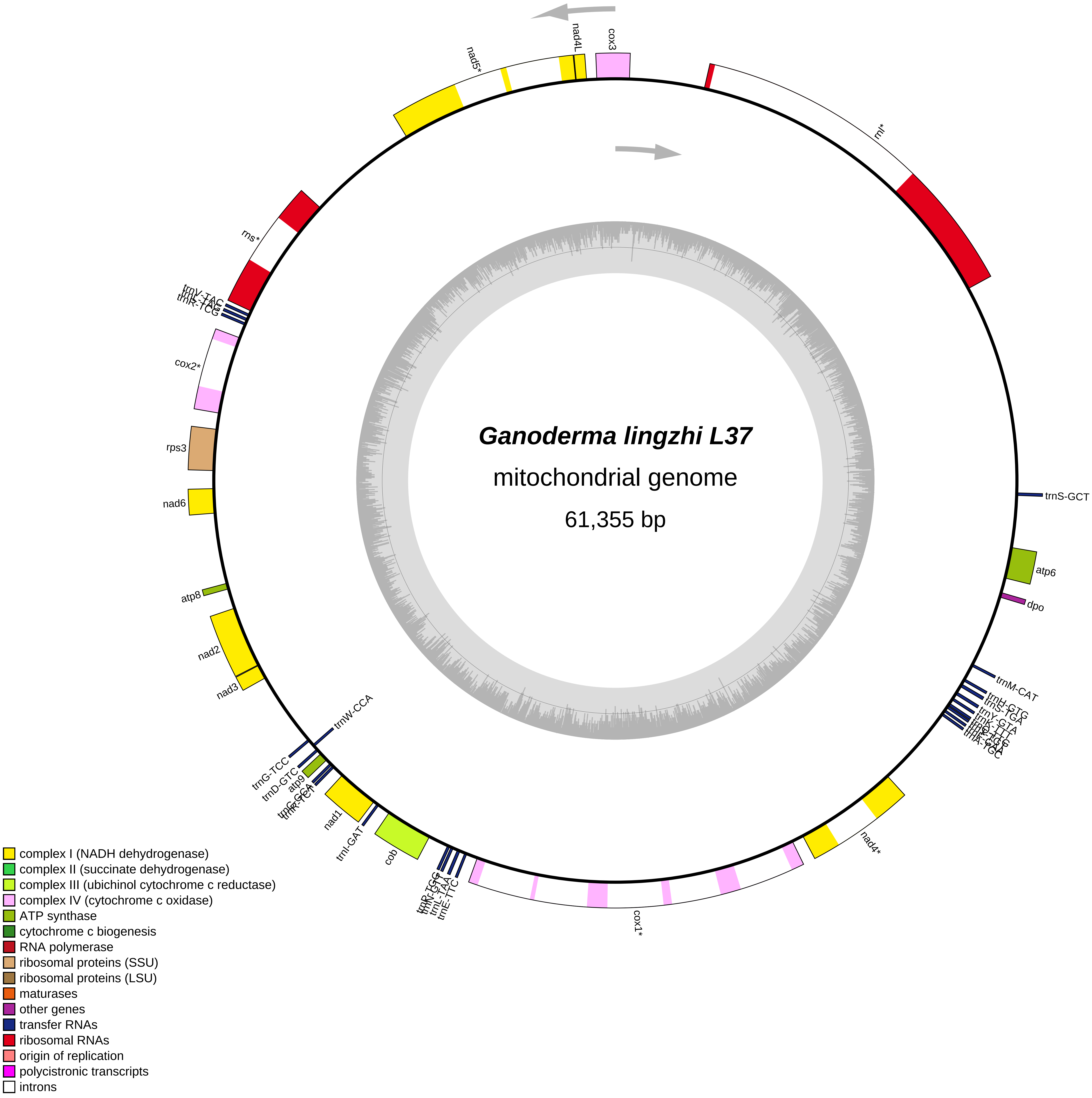

Ganoderma lingzhi L37
mitochondrial genome
61,355 bp
nad5*
nad4L
cox3
rnl*
rns*
trnV-TAC
trnR-TCG
cox2*
rps3
nad6
atp8
nad2
nad3
trnW-CCA
trnG-TCC
trnD-GTC
atp9
nad1
trnI-GAT
cob
trnE-TTC
cox1*
nad4*
trnM-CAT
trnH-GTG
trnS-TGA
dpo
atp6
trnS-GCT
complex I (NADH dehydrogenase)
complex II (succinate dehydrogenase)
complex III (ubichinol cytochrome c reductase)
complex IV (cytochrome c oxidase)
ATP synthase
cytochrome c biogenesis
RNA polymerase
ribosomal proteins (SSU)
ribosomal proteins (LSU)
maturases
other genes
transfer RNAs
ribosomal RNAs
origin of replication
polycistronic transcripts
introns

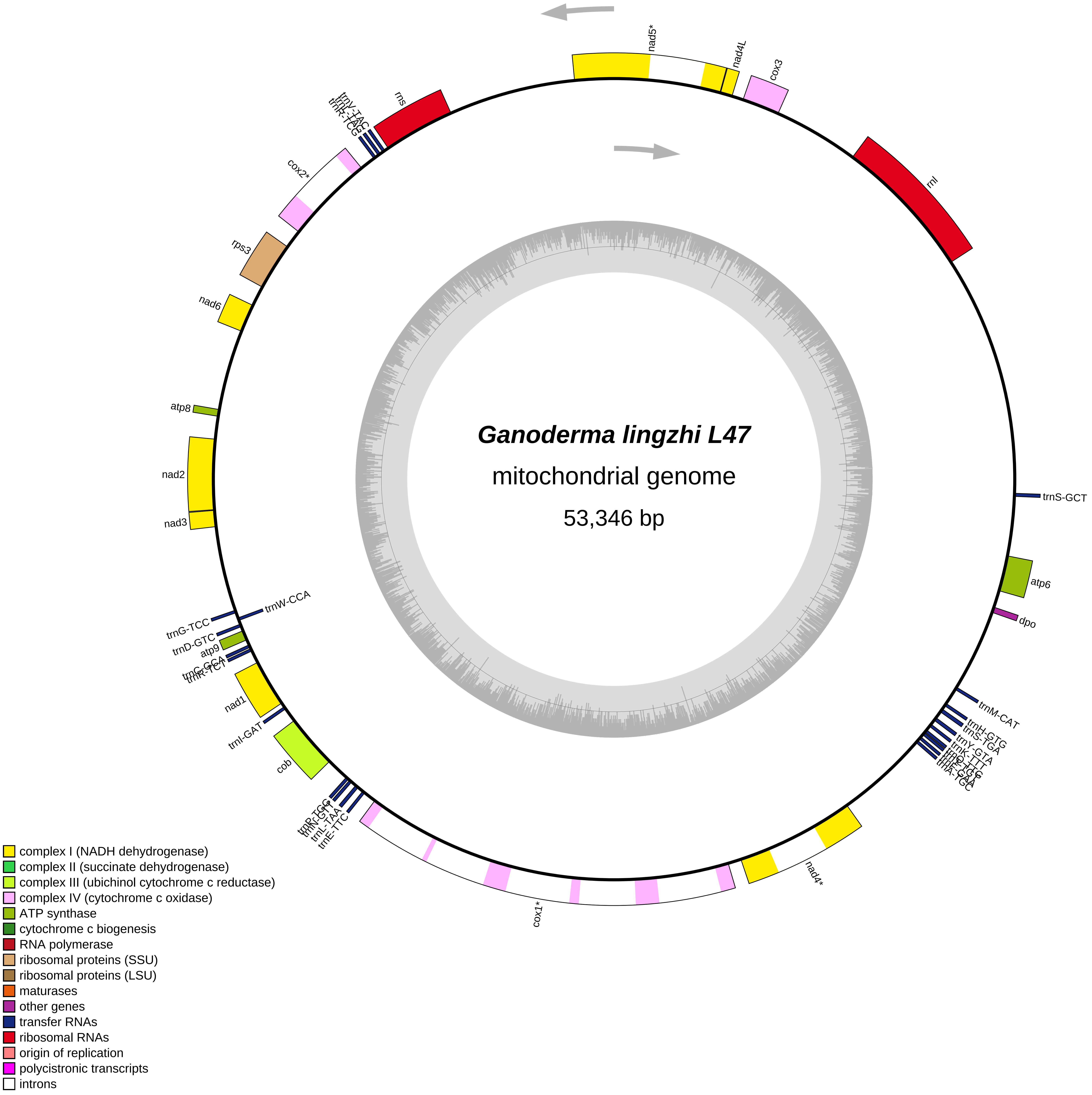

Ganoderma lingzhi L47
mitochondrial genome
53,346 bp
nad5*
nad4L
cox3
rnl
trnS-GCT
atp6
dpo
trnM-CAT
trnH-GTG
trnS-TGA
trnY-GTA
trnK-TTT
nad4*
cox1*
trnE-TTC
trnL-TAA
trnN-GTT
trnP-TGG
cob
trnI-GAT
nad1
trnR-TCT
trnC-GCA
atp9
trnD-GTC
trnG-TCC
trnW-CCA
nad3
nad2
atp8
nad6
rps3
cox2*
rns
trnV-TAC
complex I (NADH dehydrogenase)
complex II (succinate dehydrogenase)
complex III (ubichinol cytochrome c reductase)
complex IV (cytochrome c oxidase)
ATP synthase
cytochrome c biogenesis
RNA polymerase
ribosomal proteins (SSU)
ribosomal proteins (LSU)
maturases
other genes
transfer RNAs
ribosomal RNAs
origin of replication
polycistronic transcripts
introns

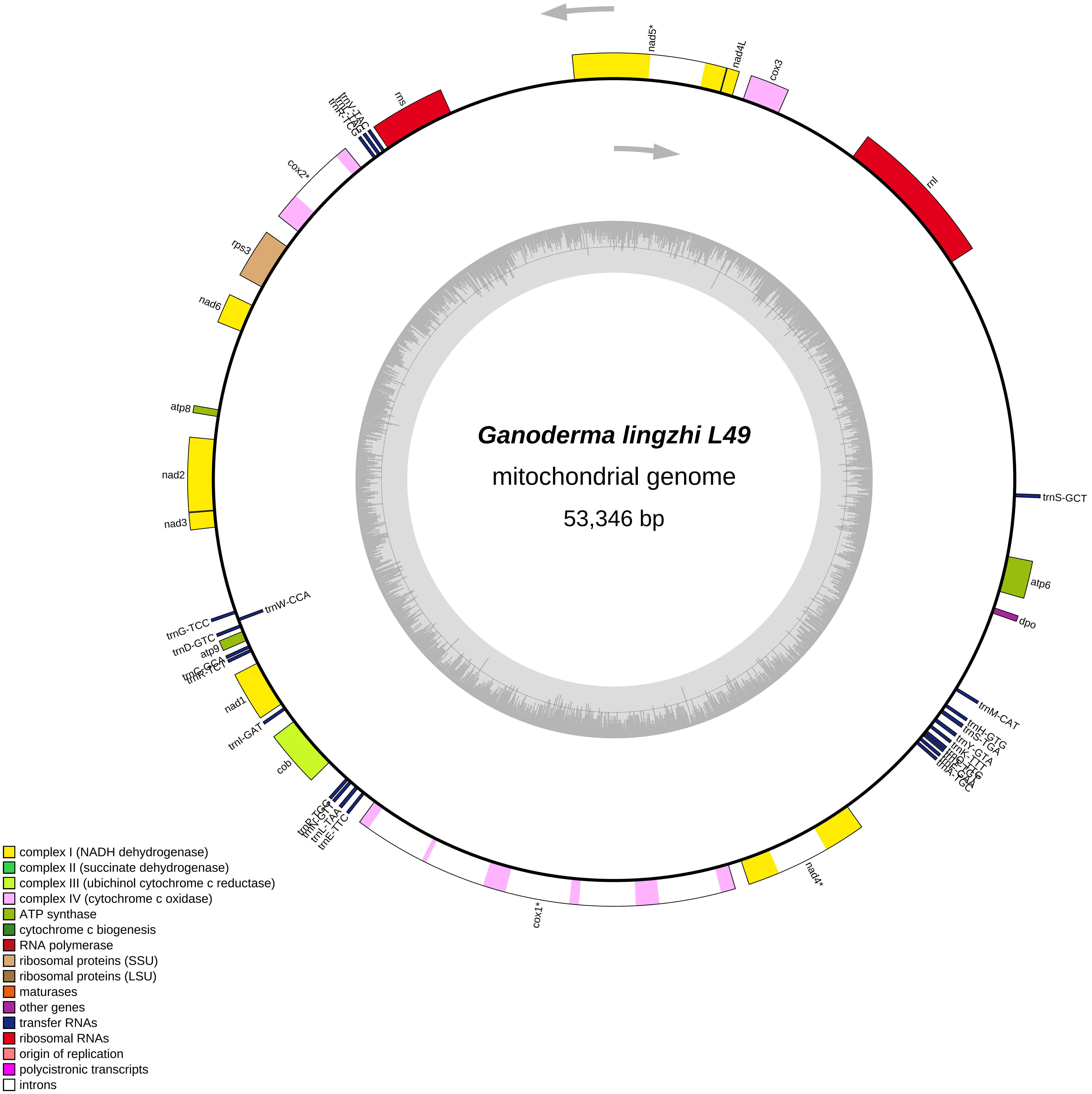
Ganoderma lingzhi L49
mitochondrial genome
53,346 bp
nad5*
nad4L
cox3
rnl
trnS-GCT
atp6
dpo
trnM-CAT
trnH-GTG
trnS-TGA
nad4*
cox1*
trnE-TTC
cob
trnI-GAT
nad1
trnR-TCT
atp9
trnD-GTC
trnG-TCC
trnW-CCA
nad3
nad2
atp8
nad6
rps3
cox2*
rns
complex I (NADH dehydrogenase)
complex II (succinate dehydrogenase)
complex III (ubichinol cytochrome c reductase)
complex IV (cytochrome c oxidase)
ATP synthase
cytochrome c biogenesis
RNA polymerase
ribosomal proteins (SSU)
ribosomal proteins (LSU)
maturases
other genes
transfer RNAs
ribosomal RNAs
origin of replication
polycistronic transcripts
introns

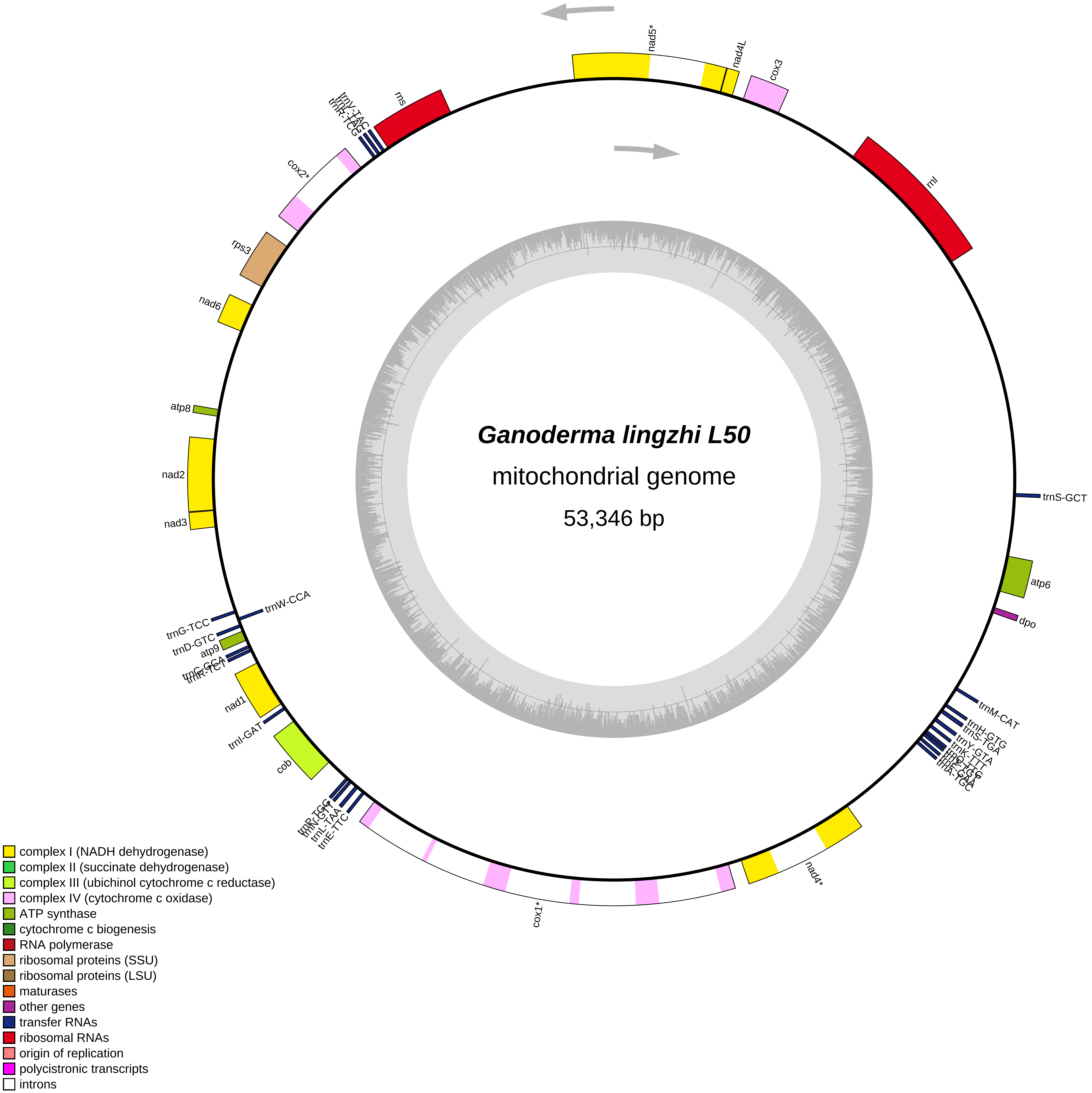

Ganoderma lingzhi L50
mitochondrial genome
53,346 bp
nad5*
nad4L
cox3
rnl
rns
cox2*
rps3
nad6
atp8
nad2
nad3
trnW-CCA
trnG-TCC
trnD-GTC
atp9
nad1
trnI-GAT
cob
trnE-TTC
cox1*
nad4*
trnM-CAT
trnH-GTG
trnS-GCT
atp6
dpo
complex I (NADH dehydrogenase)
complex II (succinate dehydrogenase)
complex III (ubichinol cytochrome c reductase)
complex IV (cytochrome c oxidase)
ATP synthase
cytochrome c biogenesis
RNA polymerase
ribosomal proteins (SSU)
ribosomal proteins (LSU)
maturases
other genes
transfer RNAs
ribosomal RNAs
origin of replication
polycistronic transcripts
introns

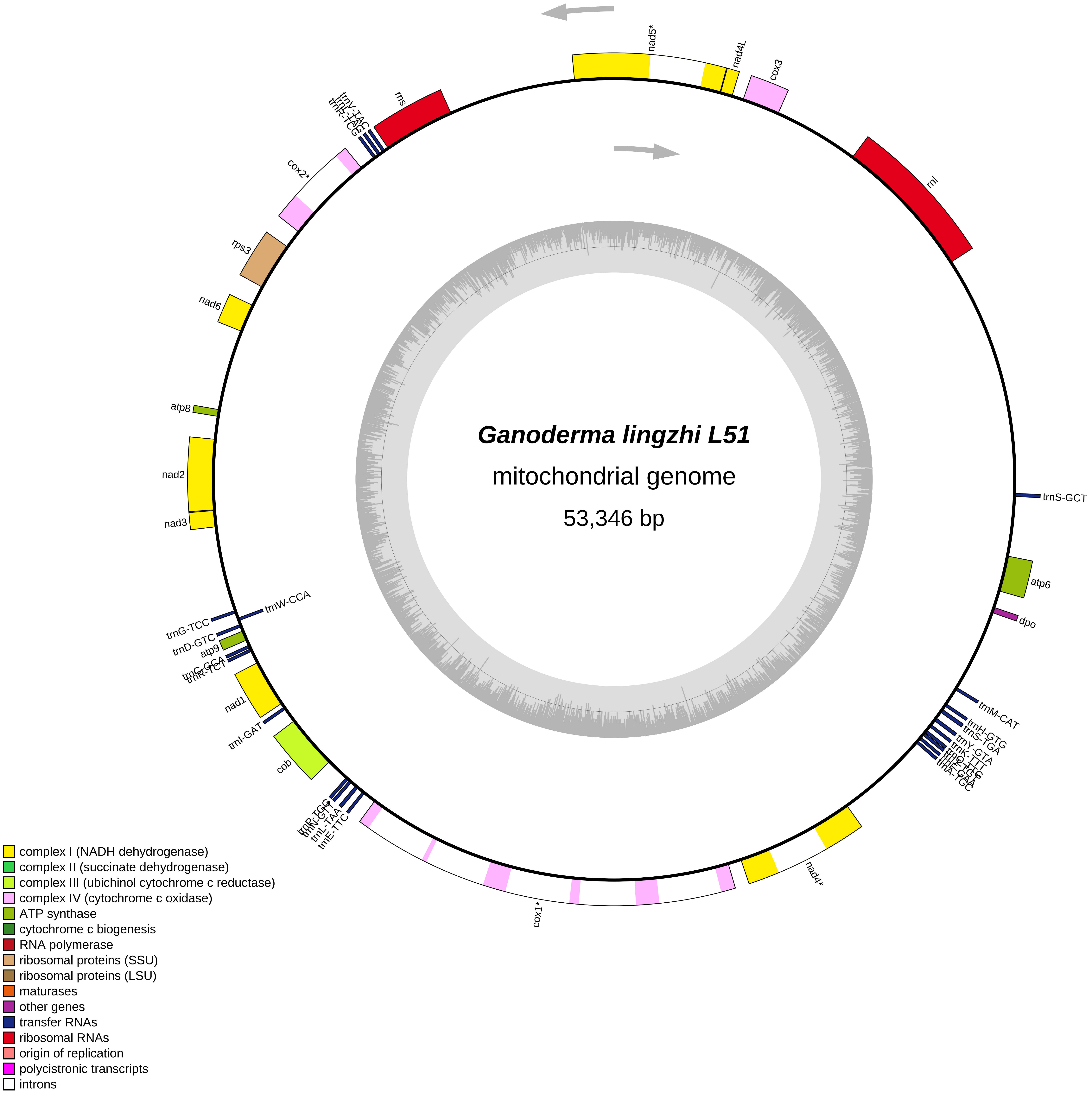
Ganoderma lingzhi L51
mitochondrial genome
53,346 bp
nad5*
nad4L
cox3
rnl
trnS-GCT
atp6
dpo
trnM-CAT
trnH-GTG
trnS-TGA
trnY-GTA
nad4*
cox1*
trnE-TTC
trnL-TAA
cob
trnI-GAT
nad1
atp9
trnD-GTC
trnG-TCC
trnW-CCA
nad3
nad2
atp8
nad6
rps3
cox2*
rns
complex I (NADH dehydrogenase)
complex II (succinate dehydrogenase)
complex III (ubichinol cytochrome c reductase)
complex IV (cytochrome c oxidase)
ATP synthase
cytochrome c biogenesis
RNA polymerase
ribosomal proteins (SSU)
ribosomal proteins (LSU)
maturases
other genes
transfer RNAs
ribosomal RNAs
origin of replication
polycistronic transcripts
introns

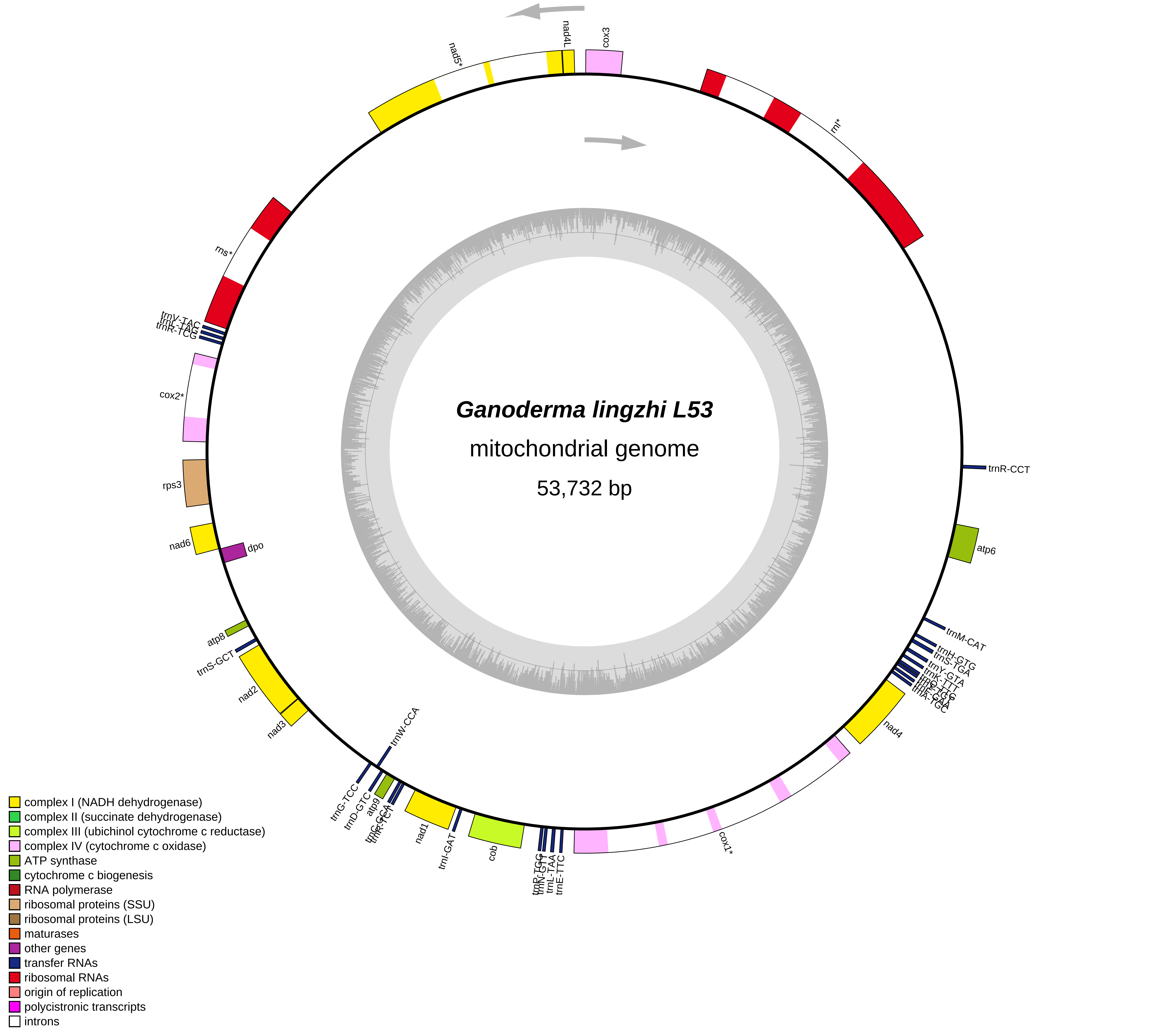
Ganoderma lingzhi L53
mitochondrial genome
53,732 bp
nad5*
nad4L
cox3
rnl*
trnR-CCT
atp6
trnM-CAT
trnH-GTG
trnS-TGA
trnY-GTA
nad4
cox1*
trnE-TTC
trnL-TAA
cob
trnI-GAT
nad1
atp9
trnD-GTC
trnG-TCC
trnW-CCA
nad3
nad2
trnS-GCT
atp8
nad6
dpo
rps3
cox2*
trnV-TAC
trnR-TCG
rns*
complex I (NADH dehydrogenase)
complex II (succinate dehydrogenase)
complex III (ubichinol cytochrome c reductase)
complex IV (cytochrome c oxidase)
ATP synthase
cytochrome c biogenesis
RNA polymerase
ribosomal proteins (SSU)
ribosomal proteins (LSU)
maturases
other genes
transfer RNAs
ribosomal RNAs
origin of replication
polycistronic transcripts
introns

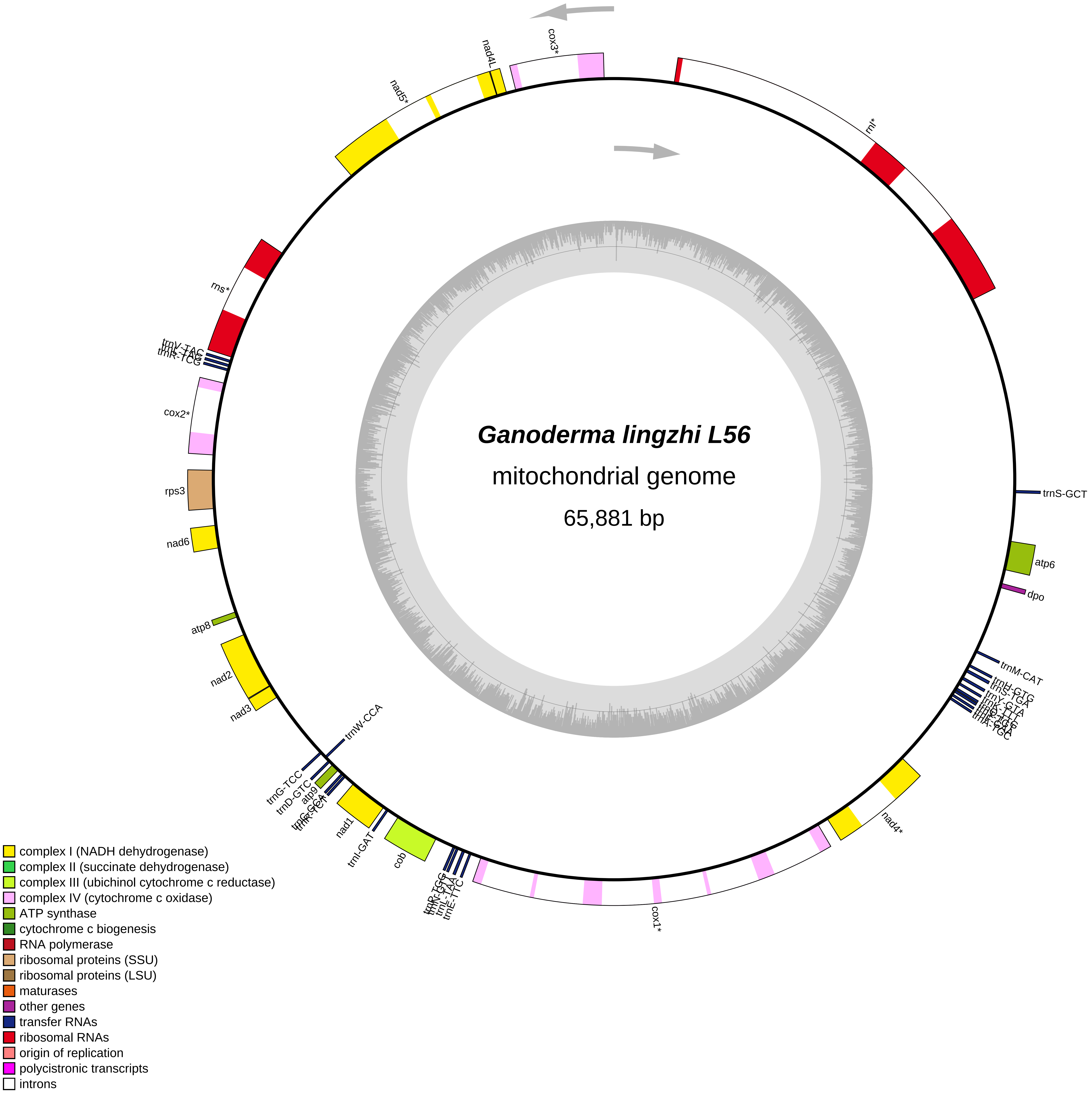

Ganoderma lingzhi L56
mitochondrial genome
65,881 bp
cox3*
nad4L
nad5*
rnl*
rns*
trnV-TAC
trnR-TCG
cox2*
rps3
nad6
atp8
nad2
nad3
trnW-CCA
trnG-TCC
trnD-GTC
atp9
nad1
trnI-GAT
cob
cox1*
nad4*
trnS-GCT
atp6
dpo
trnM-CAT
trnH-GTG
trnA-TGC
complex I (NADH dehydrogenase)
complex II (succinate dehydrogenase)
complex III (ubichinol cytochrome c reductase)
complex IV (cytochrome c oxidase)
ATP synthase
cytochrome c biogenesis
RNA polymerase
ribosomal proteins (SSU)
ribosomal proteins (LSU)
maturases
other genes
transfer RNAs
ribosomal RNAs
origin of replication
polycistronic transcripts
introns

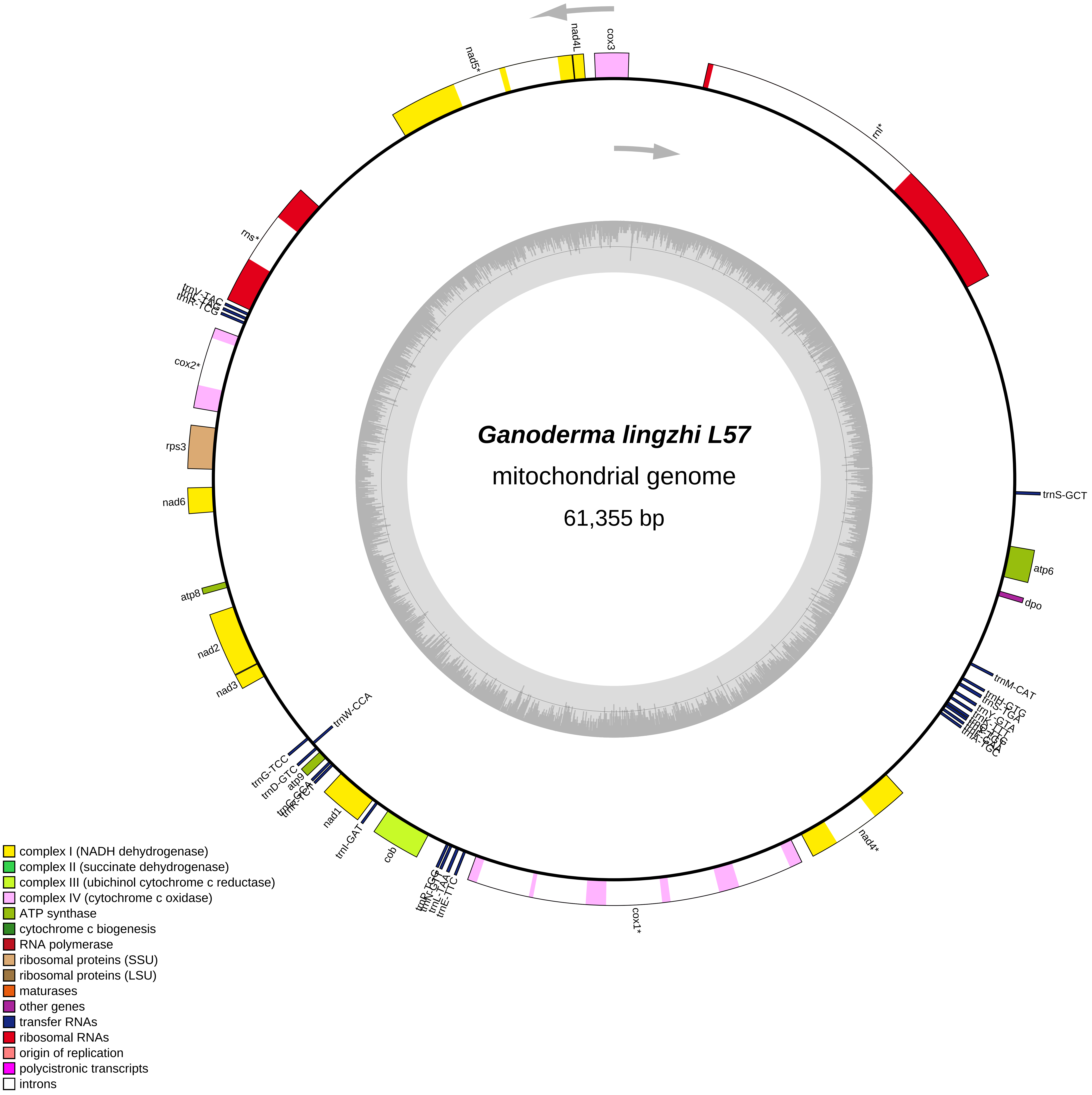
Ganoderma lingzhi L57
mitochondrial genome
61,355 bp
nad5*
nad4L
cox3
rnl*
rns*
trnV-TAC
trnR-TCG
cox2*
rps3
nad6
atp8
nad2
nad3
trnW-CCA
trnG-TCC
trnD-GTC
atp9
nad1
trnI-GAT
cob
trnE-TTC
cox1*
nad4*
trnS-GCT
atp6
dpo
trnM-CAT
trnH-GTG
complex I (NADH dehydrogenase)
complex II (succinate dehydrogenase)
complex III (ubichinol cytochrome c reductase)
complex IV (cytochrome c oxidase)
ATP synthase
cytochrome c biogenesis
RNA polymerase
ribosomal proteins (SSU)
ribosomal proteins (LSU)
maturases
other genes
transfer RNAs
ribosomal RNAs
origin of replication
polycistronic transcripts
introns

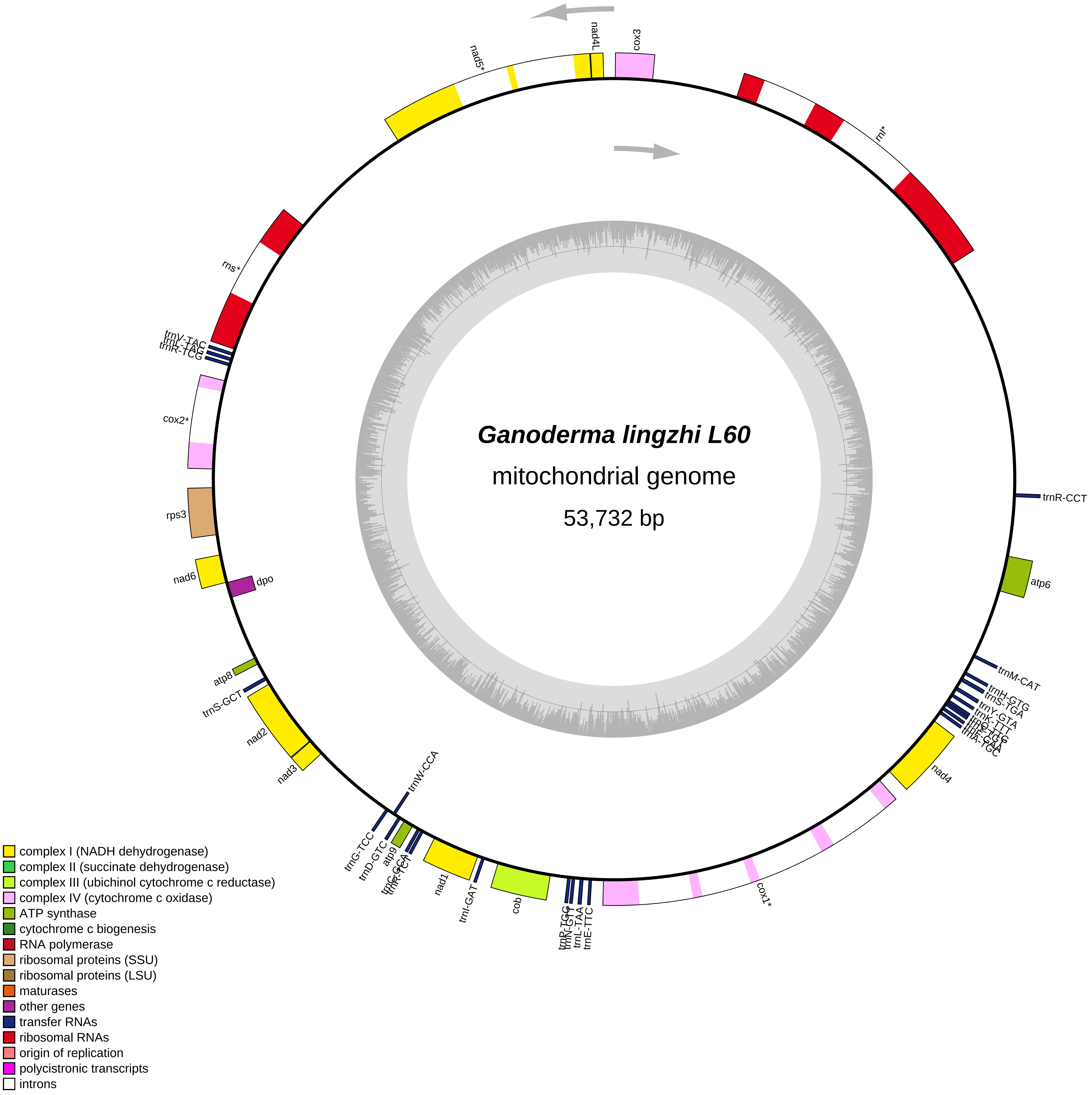

Ganoderma lingzhi L60
mitochondrial genome
53,732 bp
nad5*
nad4L
cox3
rnl*
rns*
trnV-TAC
trnR-TCG
cox2*
rps3
nad6
dpo
trnR-CCT
atp6
trnM-CAT
trnH-GTG
trnS-TGA
trnY-GTA
nad4
cox1*
trnE-TTC
trnL-TAA
cob
trnI-GAT
nad1
atp9
trnD-GTC
trnG-TCC
trnW-CCA
nad3
nad2
trnS-GCT
atp8
complex I (NADH dehydrogenase)
complex II (succinate dehydrogenase)
complex III (ubichinol cytochrome c reductase)
complex IV (cytochrome c oxidase)
ATP synthase
cytochrome c biogenesis
RNA polymerase
ribosomal proteins (SSU)
ribosomal proteins (LSU)
maturases
other genes
transfer RNAs
ribosomal RNAs
origin of replication
polycistronic transcripts
introns

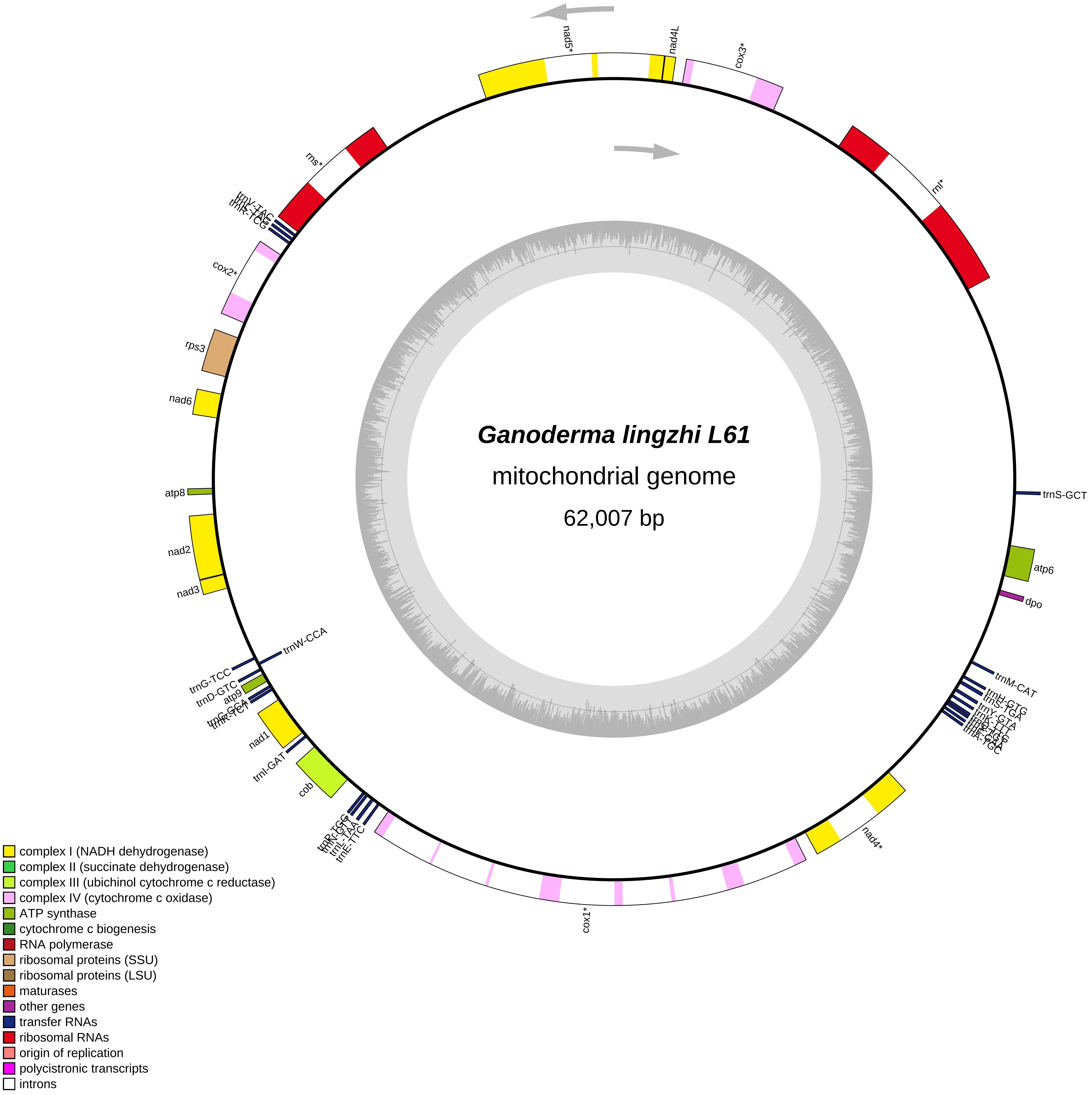

Ganoderma lingzhi L61
mitochondrial genome
62,007 bp
nad5*
nad4L
cox3*
rnl*
rns*
trnR-TCG
cox2*
rps3
nad6
atp8
nad2
nad3
trnW-CCA
trnG-TCC
trnD-GTC
atp9
nad1
trnI-GAT
cob
trnL-TAA
trnE-TTC
cox1*
nad4*
trnM-CAT
trnH-GTG
trnS-TGA
trnS-GCT
atp6
dpo
complex I (NADH dehydrogenase)
complex II (succinate dehydrogenase)
complex III (ubichinol cytochrome c reductase)
complex IV (cytochrome c oxidase)
ATP synthase
cytochrome c biogenesis
RNA polymerase
ribosomal proteins (SSU)
ribosomal proteins (LSU)
maturases
other genes
transfer RNAs
ribosomal RNAs
origin of replication
polycistronic transcripts
introns

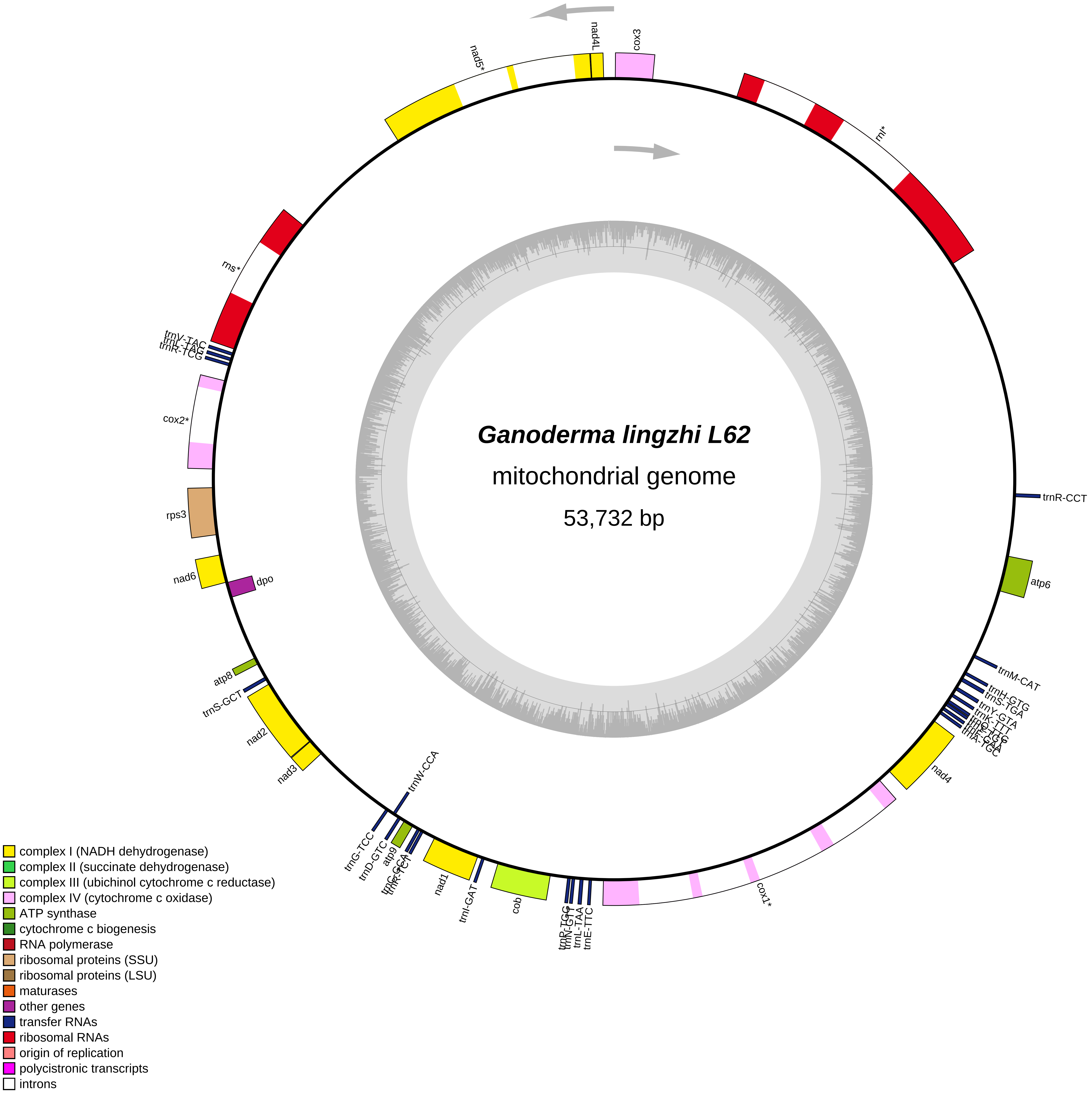

Ganoderma lingzhi L62
mitochondrial genome
53,732 bp
nad5*
nad4L
cox3
rnl*
trnR-CCT
atp6
trnM-CAT
trnH-GTG
trnS-TGA
trnY-GTA
nad4
cox1*
trnE-TTC
trnL-TAA
cob
trnI-GAT
nad1
atp9
trnD-GTC
trnG-TCC
trnW-CCA
nad3
nad2
trnS-GCT
atp8
nad6
dpo
rps3
cox2*
trnR-TCG
trnV-TAC
rns*
complex I (NADH dehydrogenase)
complex II (succinate dehydrogenase)
complex III (ubichinol cytochrome c reductase)
complex IV (cytochrome c oxidase)
ATP synthase
cytochrome c biogenesis
RNA polymerase
ribosomal proteins (SSU)
ribosomal proteins (LSU)
maturases
other genes
transfer RNAs
ribosomal RNAs
origin of replication
polycistronic transcripts
introns

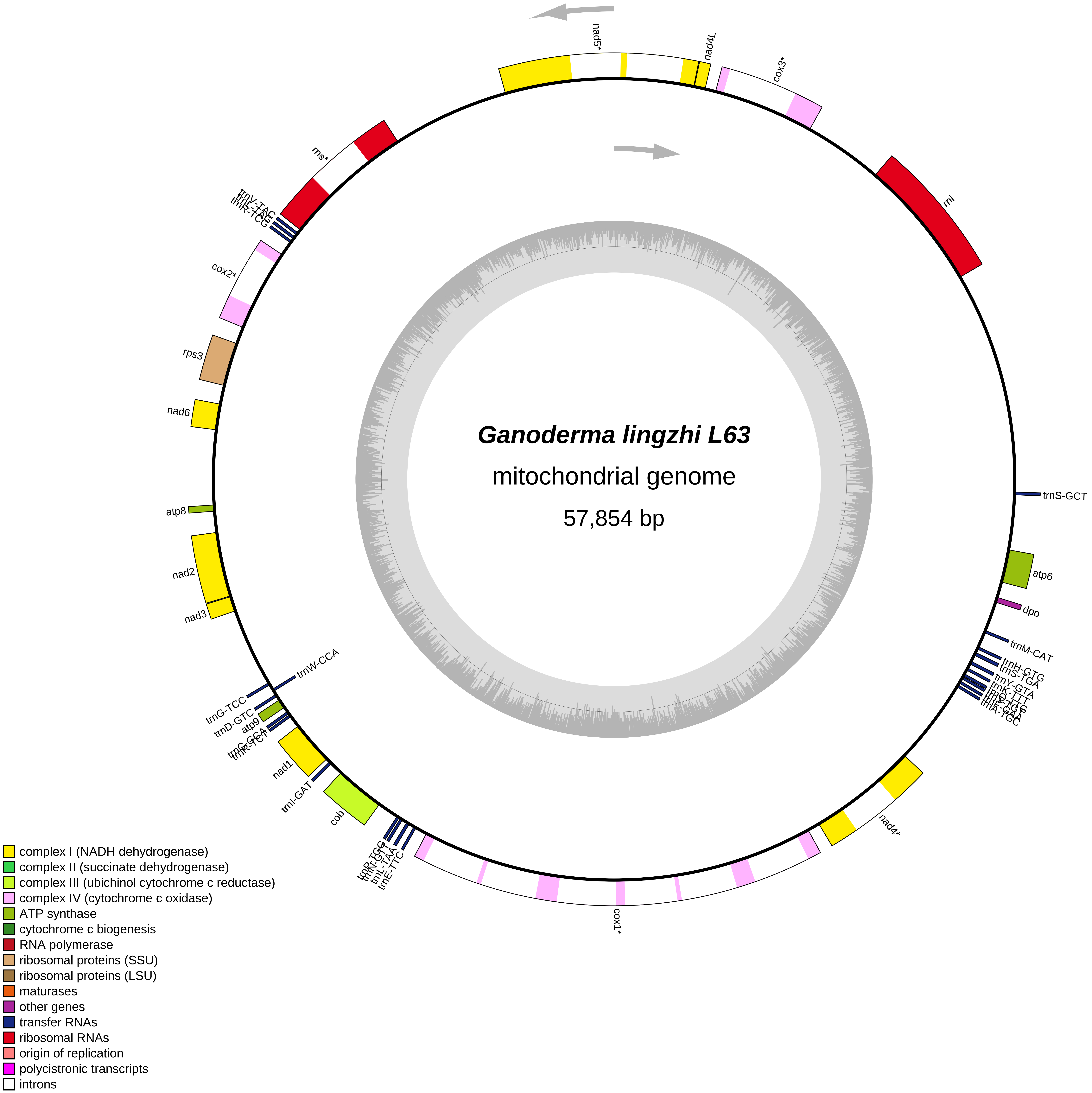
Ganoderma lingzhi L63
mitochondrial genome
57,854 bp
nad5*
nad4L
cox3*
rnl
trnS-GCT
atp6
dpo
trnM-CAT
trnH-GTG
trnS-TGA
trnY-GTA
nad4*
cox1*
trnE-TTC
trnL-TAA
cob
trnI-GAT
nad1
atp9
trnD-GTC
trnG-TCC
trnW-CCA
nad3
nad2
atp8
nad6
rps3
cox2*
trnR-TCG
trnV-TAC
rns*
complex I (NADH dehydrogenase)
complex II (succinate dehydrogenase)
complex III (ubichinol cytochrome c reductase)
complex IV (cytochrome c oxidase)
ATP synthase
cytochrome c biogenesis
RNA polymerase
ribosomal proteins (SSU)
ribosomal proteins (LSU)
maturases
other genes
transfer RNAs
ribosomal RNAs
origin of replication
polycistronic transcripts
introns

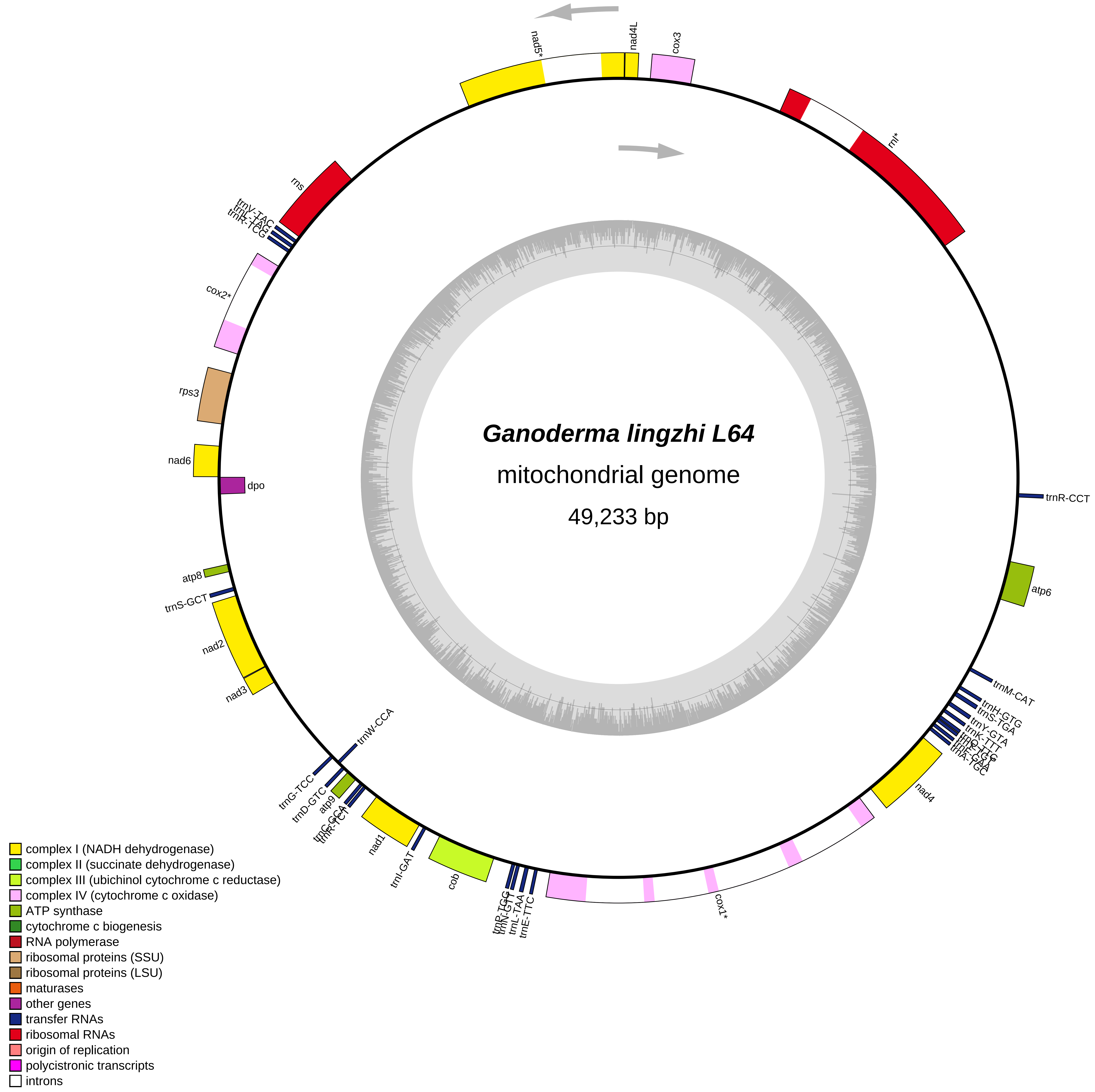

Ganoderma lingzhi L64
mitochondrial genome
49,233 bp
nad5*
nad4L
cox3
rnl*
rns
trnV-TAC
trnL-TAG
trnR-TCG
cox2*
rps3
nad6
dpo
trnR-CCT
atp6
trnM-CAT
trnH-GTG
trnS-TGA
trnY-GTA
trnK-TTT
trnA-TGC
nad4
cox1*
trnP-TGG
trnL-TAA
trnE-TTC
cob
trnI-GAT
nad1
trnC-GCA
atp9
trnD-GTC
trnG-TCC
trnW-CCA
nad3
nad2
trnS-GCT
atp8
complex I (NADH dehydrogenase)
complex II (succinate dehydrogenase)
complex III (ubichinol cytochrome c reductase)
complex IV (cytochrome c oxidase)
ATP synthase
cytochrome c biogenesis
RNA polymerase
ribosomal proteins (SSU)
ribosomal proteins (LSU)
maturases
other genes
transfer RNAs
ribosomal RNAs
origin of replication
polycistronic transcripts
introns

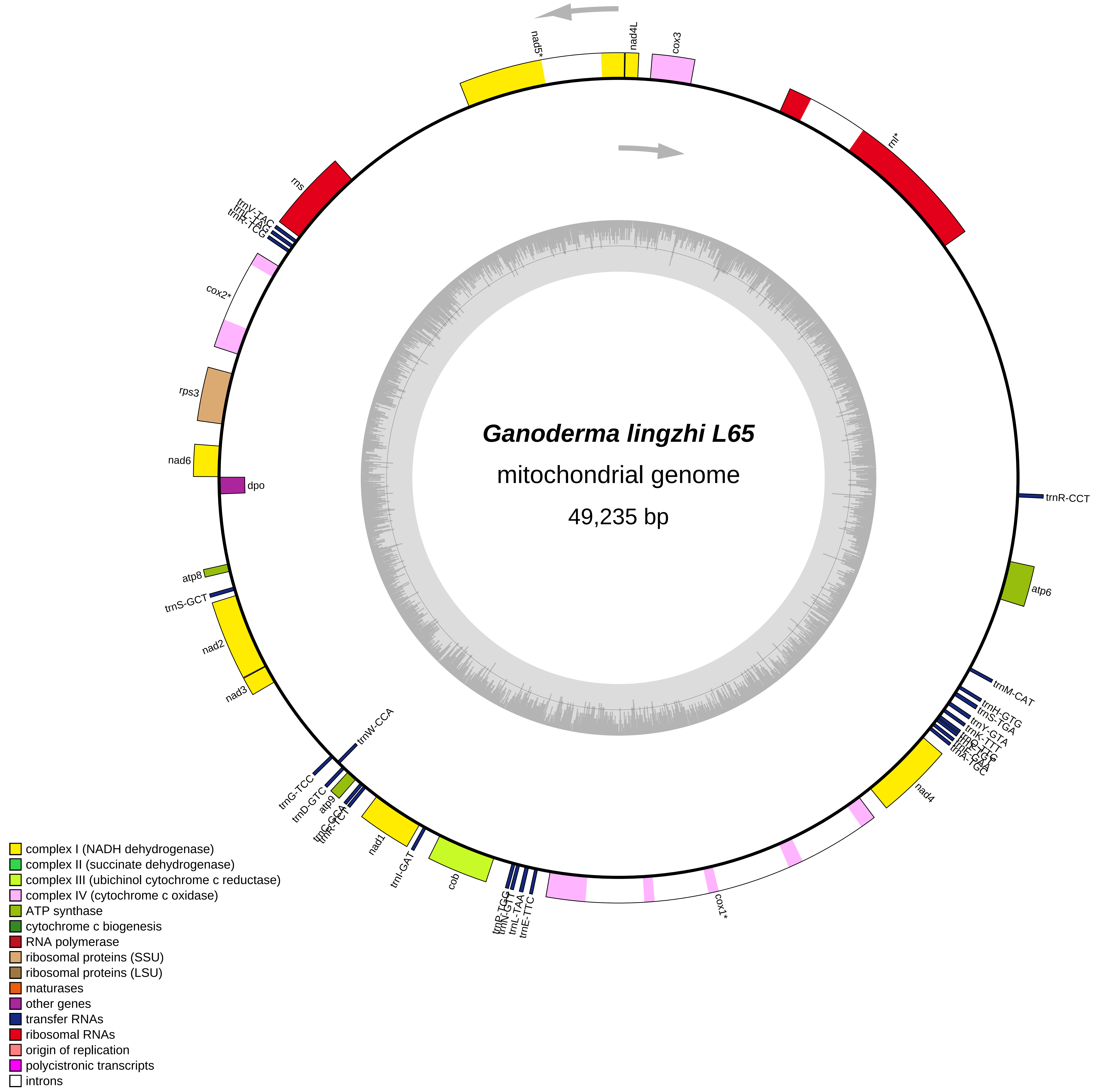
Ganoderma lingzhi L65
mitochondrial genome
49,235 bp
nad5*
nad4L
cox3
rnl*
trnR-CCT
atp6
trnM-CAT
trnH-GTG
trnS-TGA
trnY-GTA
trnK-TTT
trnA-TGC
nad4
cox1*
trnE-TTC
trnL-TAA
trnP-TGG
cob
trnI-GAT
nad1
trnR-TCT
atp9
trnD-GTC
trnG-TCC
trnW-CCA
nad3
nad2
trnS-GCT
atp8
dpo
nad6
rps3
cox2*
trnR-TCG
trnV-TAC
rns
complex I (NADH dehydrogenase)
complex II (succinate dehydrogenase)
complex III (ubichinol cytochrome c reductase)
complex IV (cytochrome c oxidase)
ATP synthase
cytochrome c biogenesis
RNA polymerase
ribosomal proteins (SSU)
ribosomal proteins (LSU)
maturases
other genes
transfer RNAs
ribosomal RNAs
origin of replication
polycistronic transcripts
introns

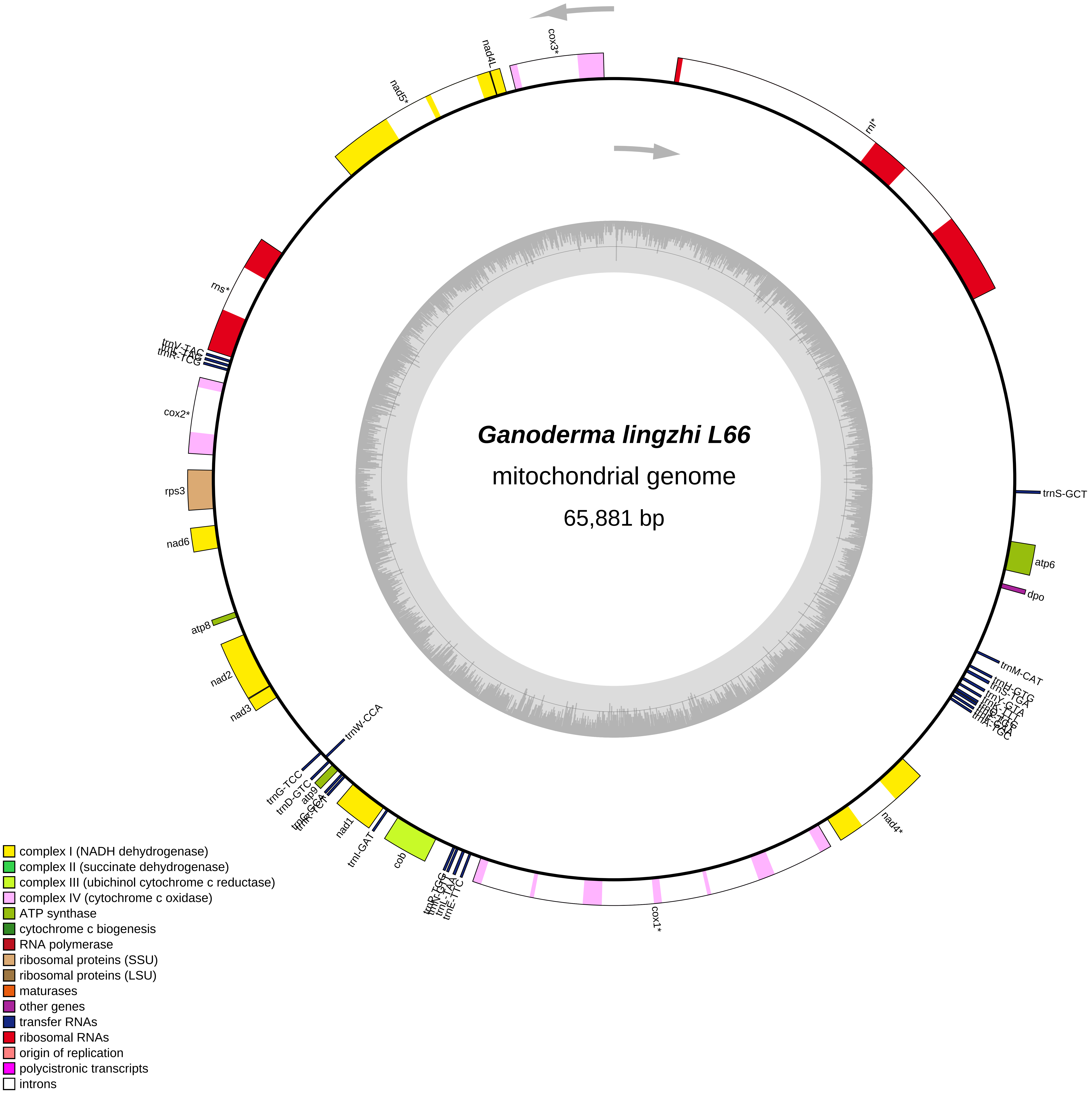
Ganoderma lingzhi L66
mitochondrial genome
65,881 bp
cox3*
nad4L
nad5*
rnl*
rns*
cox2*
rps3
nad6
atp8
nad2
nad3
trnW-CCA
trnG-TCC
trnD-GTC
atp9
nad1
trnI-GAT
cob
cox1*
nad4*
trnM-CAT
trnH-GTG
trnS-GCT
atp6
dpo
trnR-TCG
trnY-TAC
trnE-TTC
complex I (NADH dehydrogenase)
complex II (succinate dehydrogenase)
complex III (ubichinol cytochrome c reductase)
complex IV (cytochrome c oxidase)
ATP synthase
cytochrome c biogenesis
RNA polymerase
ribosomal proteins (SSU)
ribosomal proteins (LSU)
maturases
other genes
transfer RNAs
ribosomal RNAs
origin of replication
polycistronic transcripts
introns

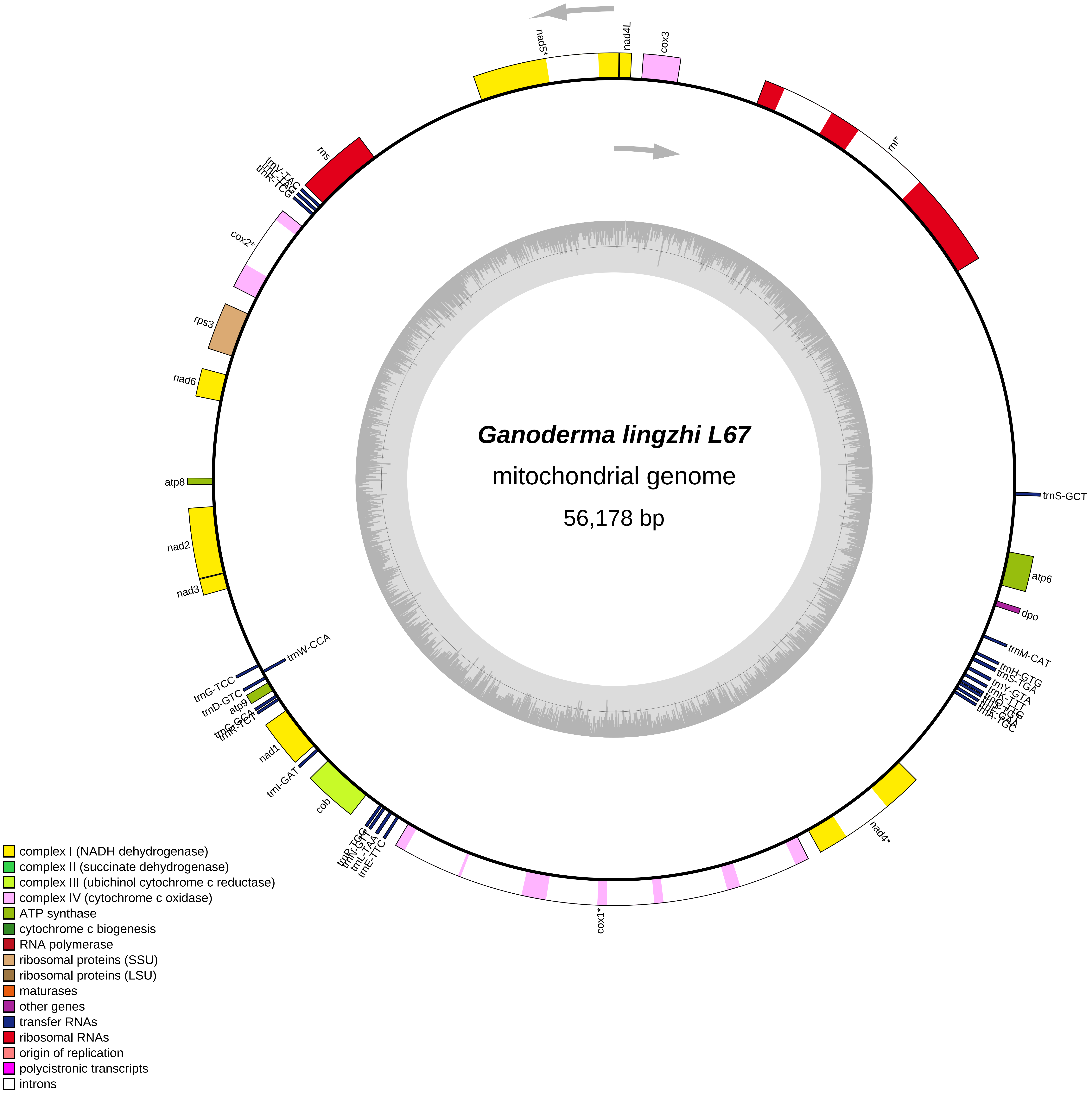
Ganoderma lingzhi L67
mitochondrial genome
56,178 bp
nad5*
nad4L
cox3
rnl*
rns
trnV-TAC
trnR-TCG
cox2*
rps3
nad6
atp8
nad2
nad3
trnW-CCA
trnG-TCC
trnD-GTC
atp9
trnC-GCA
trnR-TCT
nad1
trnI-GAT
cob
trnR-TCG
trnN-GTT
trnL-TAA
trnE-TTC
cox1*
nad4*
trnS-GCT
atp6
dpo
trnM-CAT
trnH-GTG
trnS-TGA
trnY-GTA
trnK-TTT
trnA-TGC
complex I (NADH dehydrogenase)
complex II (succinate dehydrogenase)
complex III (ubichinol cytochrome c reductase)
complex IV (cytochrome c oxidase)
ATP synthase
cytochrome c biogenesis
RNA polymerase
ribosomal proteins (SSU)
ribosomal proteins (LSU)
maturases
other genes
transfer RNAs
ribosomal RNAs
origin of replication
polycistronic transcripts
introns

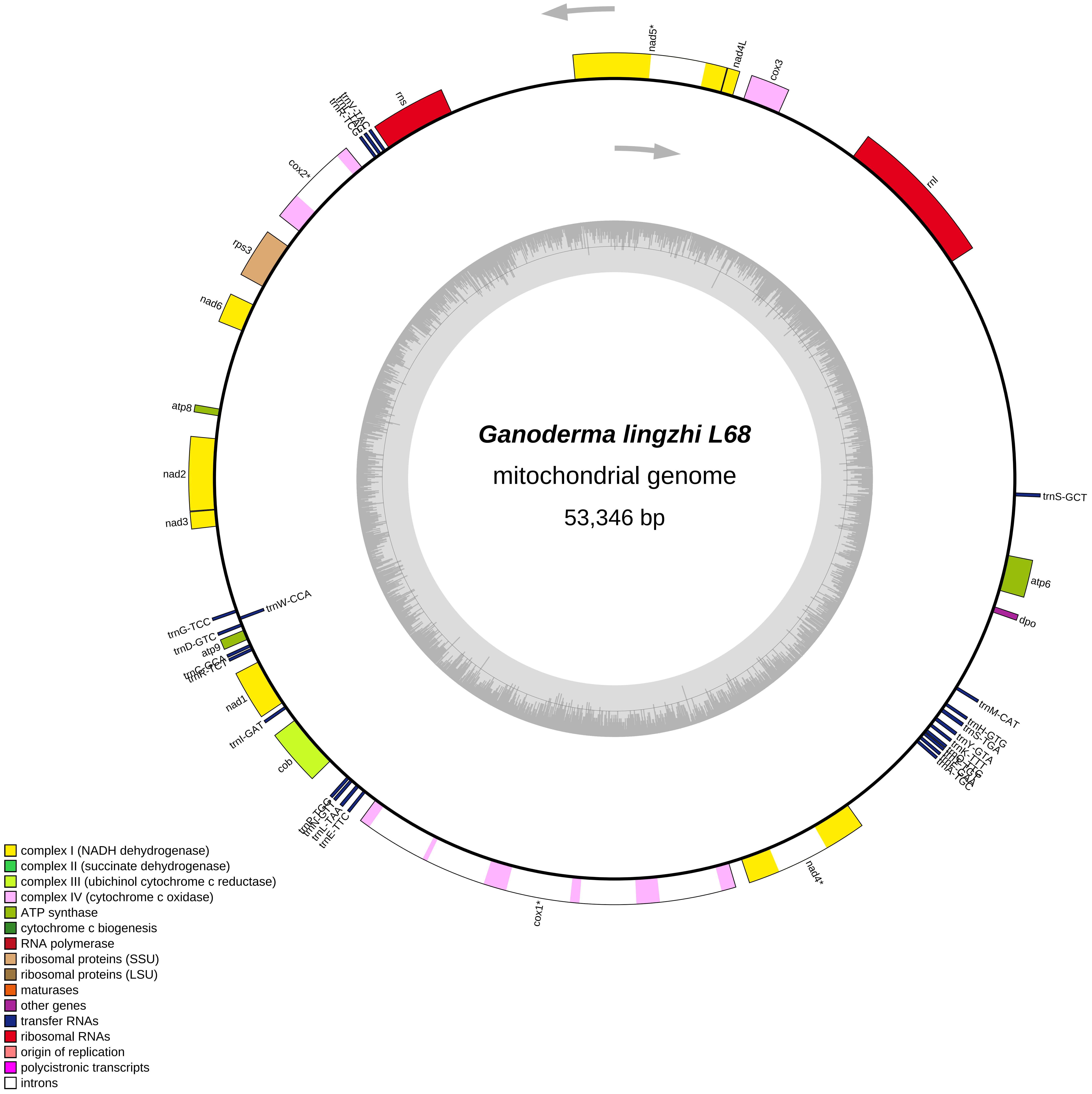

Ganoderma lingzhi L68
mitochondrial genome
53,346 bp
nad5*
nad4L
cox3
rnl
trnS-GCT
atp6
dpo
trnM-CAT
trnH-GTG
trnS-TGA
trnY-GTA
nad4*
cox1*
trnE-TTC
trnL-TAA
cob
trnI-GAT
nad1
trnR-TCT
atp9
trnD-GTC
trnG-TCC
trnW-CCA
nad3
nad2
atp8
nad6
rps3
cox2*
rns
trnV-TAC
complex I (NADH dehydrogenase)
complex II (succinate dehydrogenase)
complex III (ubichinol cytochrome c reductase)
complex IV (cytochrome c oxidase)
ATP synthase
cytochrome c biogenesis
RNA polymerase
ribosomal proteins (SSU)
ribosomal proteins (LSU)
maturases
other genes
transfer RNAs
ribosomal RNAs
origin of replication
polycistronic transcripts
introns

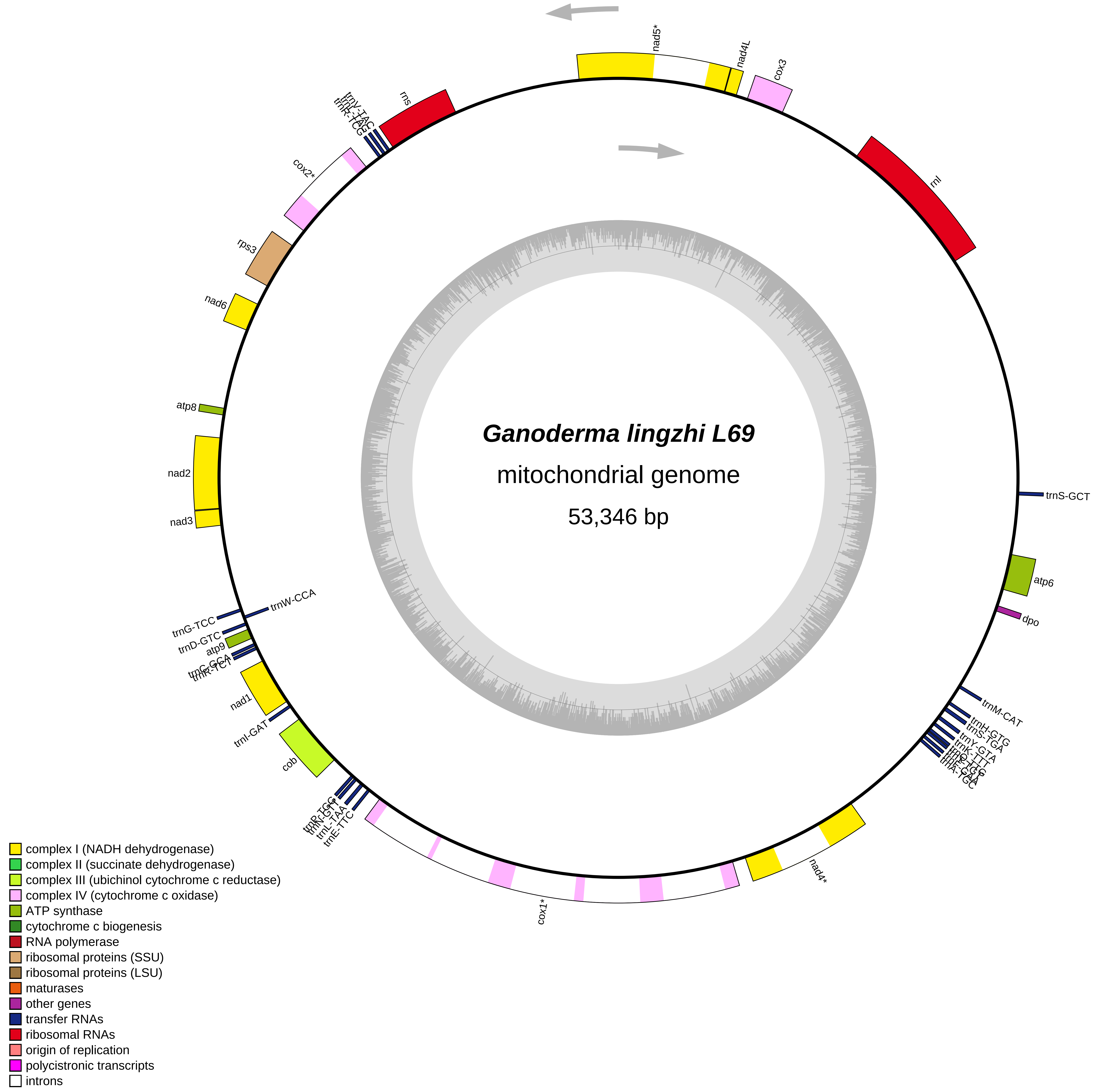

Ganoderma lingzhi L69
mitochondrial genome
53,346 bp
nad5*
nad4L
cox3
rnl
trnS-GCT
atp6
dpo
trnM-CAT
trnH-GTG
trnS-TGA
trnY-GTA
nad4*
cox1*
trnE-TTC
trnL-TAA
cob
trnI-GAT
nad1
trnR-TCT
atp9
trnD-GTC
trnG-TCC
trnW-CCA
nad3
nad2
atp8
nad6
rps3
cox2*
rns
trnV-TAC
complex I (NADH dehydrogenase)
complex II (succinate dehydrogenase)
complex III (ubichinol cytochrome c reductase)
complex IV (cytochrome c oxidase)
ATP synthase
cytochrome c biogenesis
RNA polymerase
ribosomal proteins (SSU)
ribosomal proteins (LSU)
maturases
other genes
transfer RNAs
ribosomal RNAs
origin of replication
polycistronic transcripts
introns

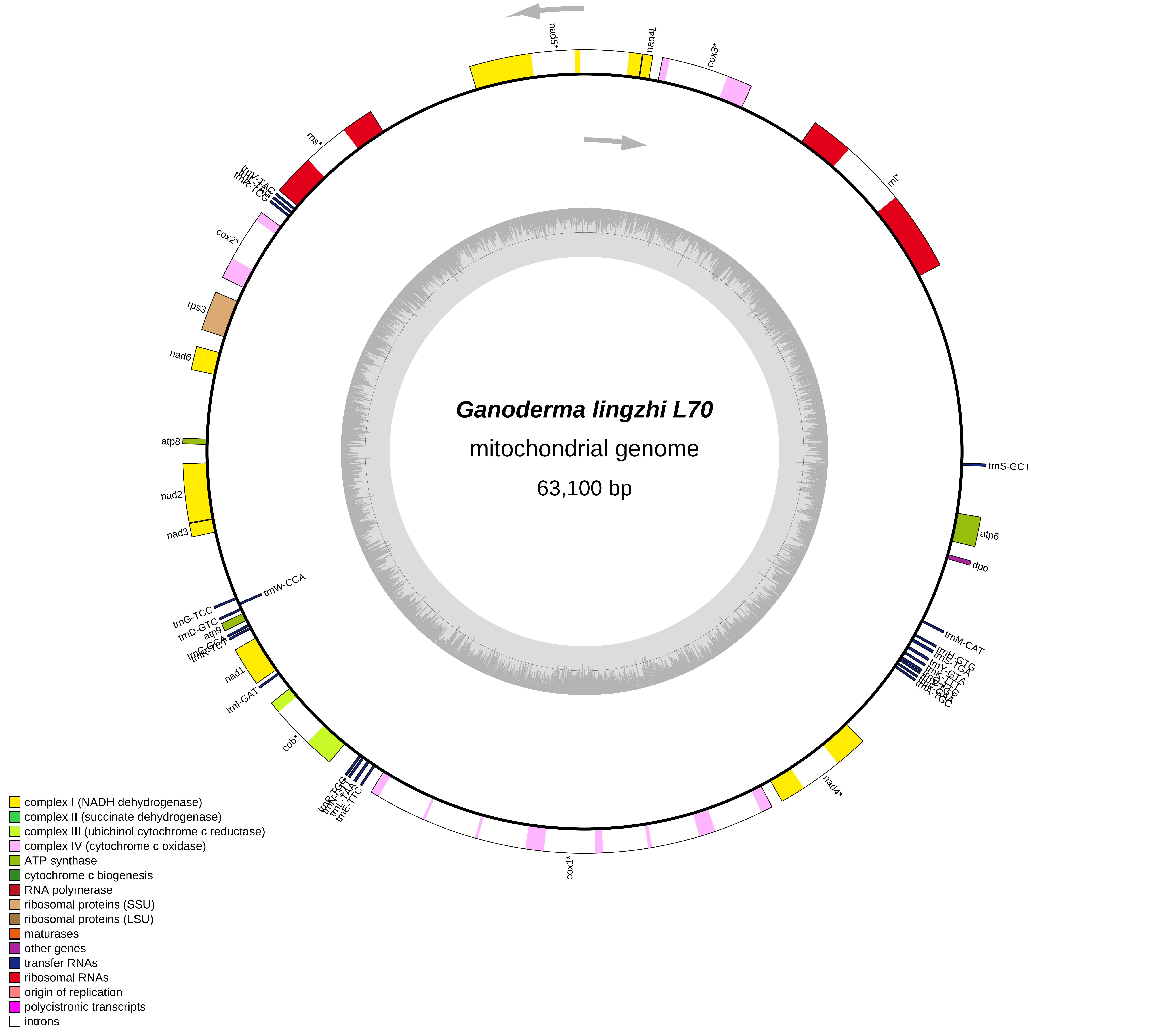

Ganoderma lingzhi L70
mitochondrial genome
63,100 bp
nad5*
nad4L
cox3*
rnl*
trnS-GCT
atp6
dpo
trnM-CAT
trnH-GTG
trnS-TGA
nad4*
cox1*
trnE-TTC
cob*
trnI-GAT
nad1
atp9
trnD-GTC
trnG-TCC
trnW-CCA
nad3
nad2
atp8
nad6
rps3
cox2*
rns*
trnV-TAC
complex I (NADH dehydrogenase)
complex II (succinate dehydrogenase)
complex III (ubichinol cytochrome c reductase)
complex IV (cytochrome c oxidase)
ATP synthase
cytochrome c biogenesis
RNA polymerase
ribosomal proteins (SSU)
ribosomal proteins (LSU)
maturases
other genes
transfer RNAs
ribosomal RNAs
origin of replication
polycistronic transcripts
introns

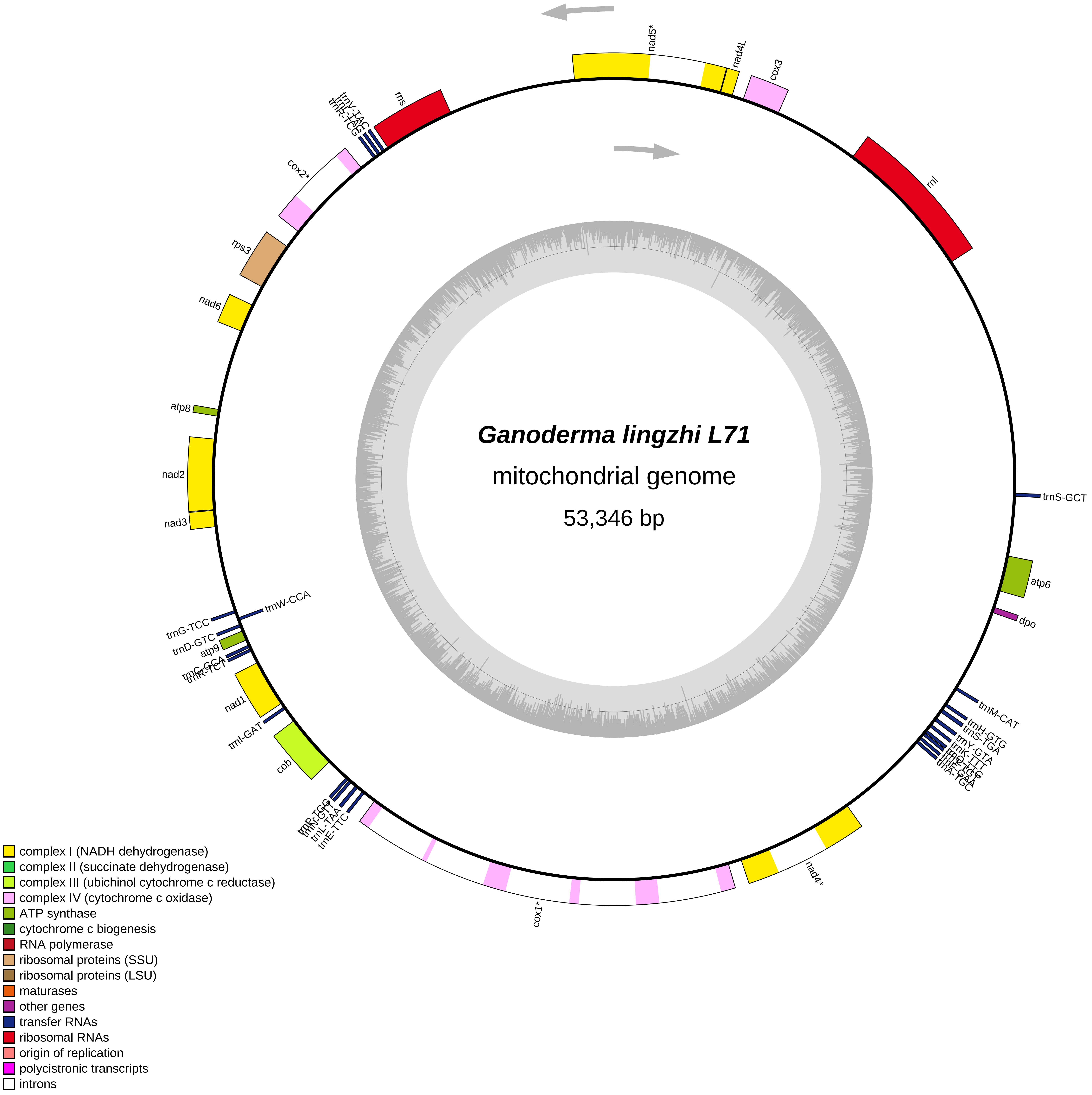

Ganoderma lingzhi L71
mitochondrial genome
53,346 bp
nad5*
nad4L
cox3
rnl
trnS-GCT
atp6
dpo
trnM-CAT
trnH-GTG
trnS-TGA
trnY-GTA
trnK-TTT
nad4*
cox1*
trnE-TTC
trnL-TAA
cob
trnI-GAT
nad1
trnR-TCT
atp9
trnD-GTC
trnG-TCC
trnW-CCA
nad3
nad2
atp8
nad6
rps3
cox2*
rns
complex I (NADH dehydrogenase)
complex II (succinate dehydrogenase)
complex III (ubichinol cytochrome c reductase)
complex IV (cytochrome c oxidase)
ATP synthase
cytochrome c biogenesis
RNA polymerase
ribosomal proteins (SSU)
ribosomal proteins (LSU)
maturases
other genes
transfer RNAs
ribosomal RNAs
origin of replication
polycistronic transcripts
introns

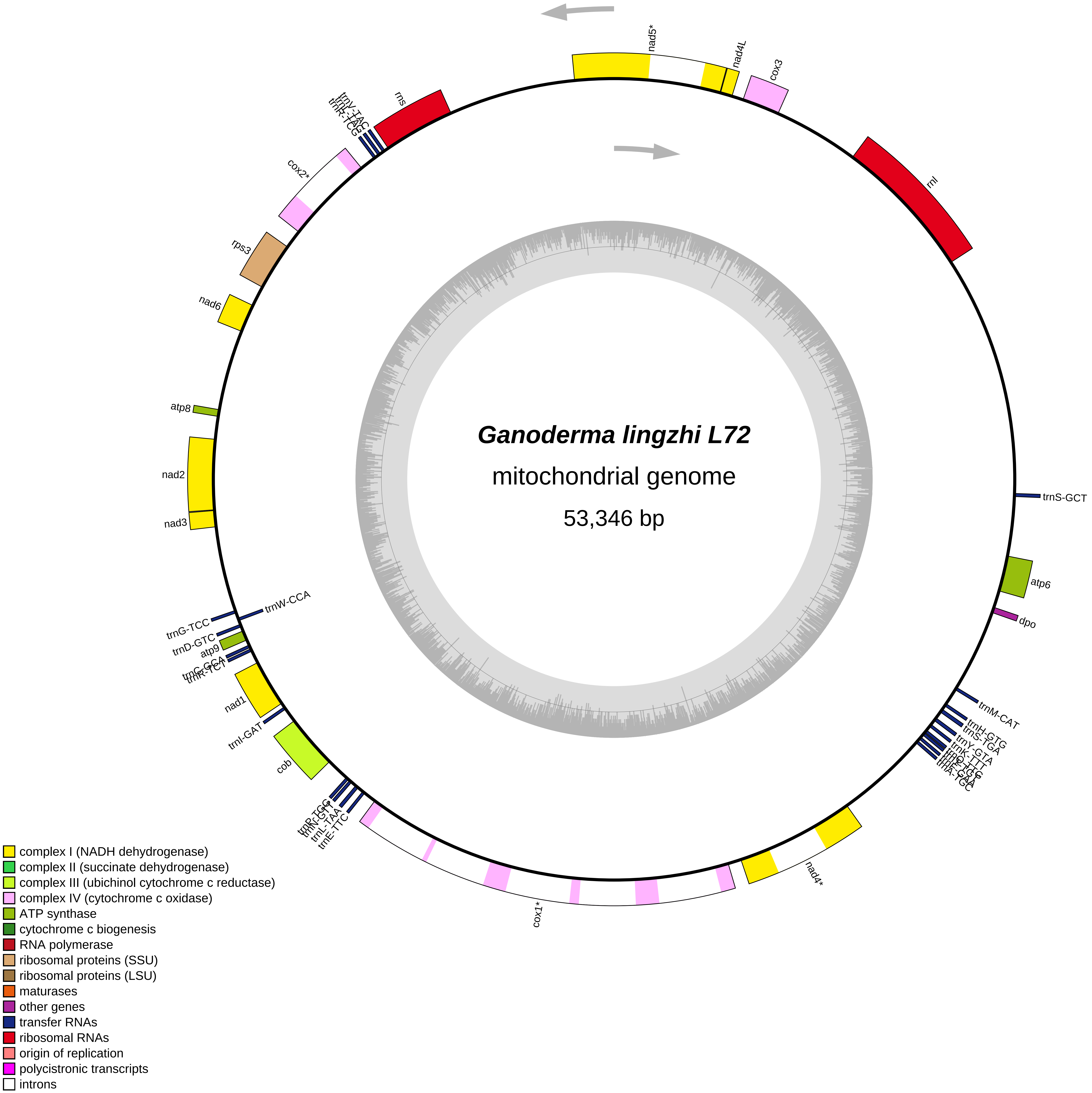

Ganoderma lingzhi L72
mitochondrial genome
53,346 bp
nad5*
nad4L
cox3
rnl
trnS-GCT
atp6
dpo
trnM-CAT
trnH-GTG
trnS-TGA
trnY-GTA
nad4*
cox1*
trnL-TAA
trnE-TTC
cob
trnI-GAT
nad1
trnR-TCT
atp9
trnD-GTC
trnG-TCC
trnW-CCA
nad3
nad2
atp8
nad6
rps3
cox2*
rns
trnV-TAC
complex I (NADH dehydrogenase)
complex II (succinate dehydrogenase)
complex III (ubichinol cytochrome c reductase)
complex IV (cytochrome c oxidase)
ATP synthase
cytochrome c biogenesis
RNA polymerase
ribosomal proteins (SSU)
ribosomal proteins (LSU)
maturases
other genes
transfer RNAs
ribosomal RNAs
origin of replication
polycistronic transcripts
introns

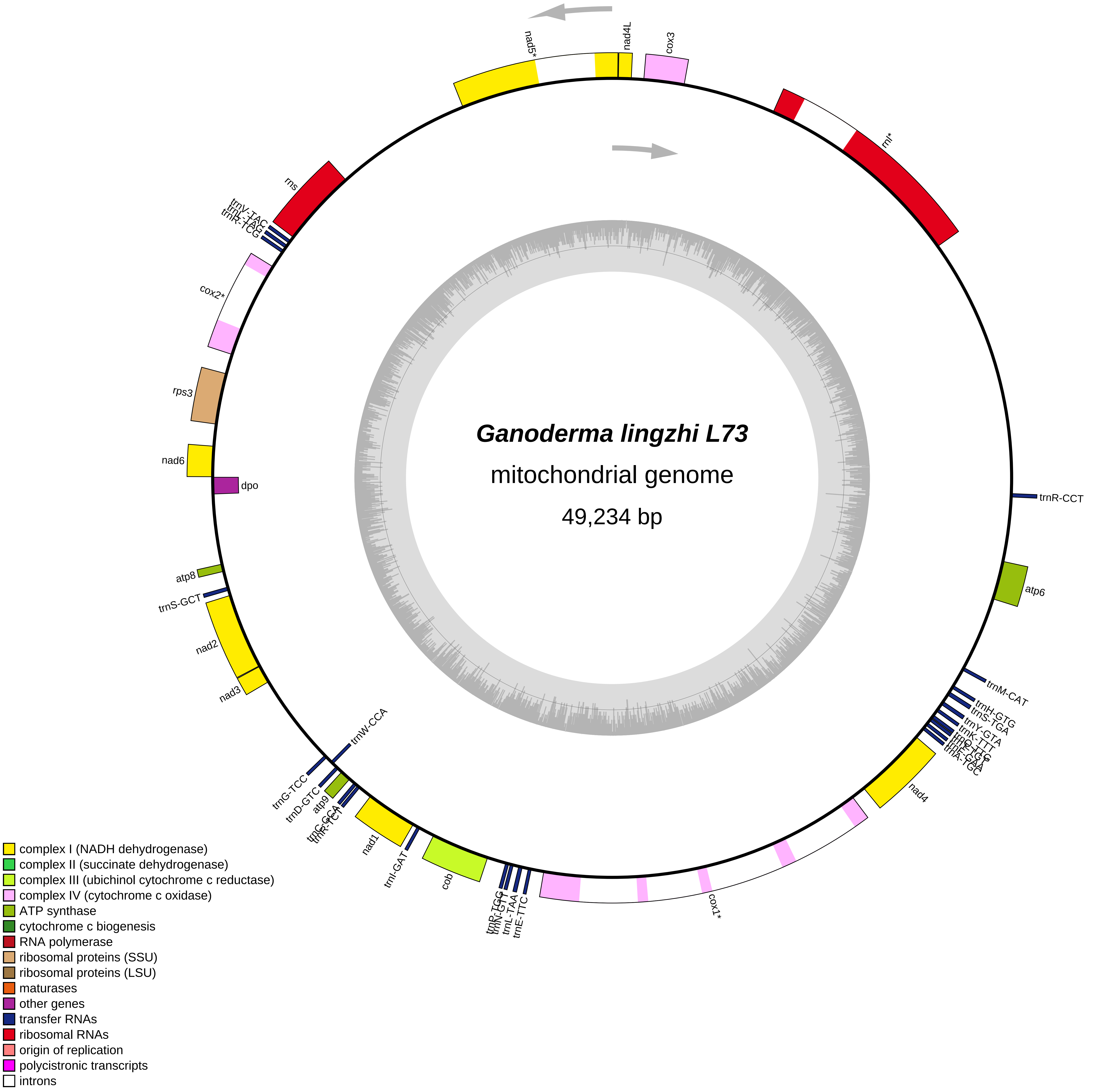

Ganoderma lingzhi L73
mitochondrial genome
49,234 bp
nad5*
nad4L
cox3
rnl*
rns
trnV-TAC
trnL-TAG
trnR-TCG
cox2*
rps3
nad6
dpo
trnR-CCT
atp6
trnM-CAT
trnH-GTG
trnS-TGA
trnY-GTA
trnK-TTT
trnA-TGC
nad4
cox1*
trnE-TTC
trnL-TAA
trnP-TGG
cob
trnI-GAT
nad1
trnW-CCA
trnG-TCC
trnD-GTC
atp9
trnC-GCA
atp8
trnS-GCT
nad2
nad3
complex I (NADH dehydrogenase)
complex II (succinate dehydrogenase)
complex III (ubichinol cytochrome c reductase)
complex IV (cytochrome c oxidase)
ATP synthase
cytochrome c biogenesis
RNA polymerase
ribosomal proteins (SSU)
ribosomal proteins (LSU)
maturases
other genes
transfer RNAs
ribosomal RNAs
origin of replication
polycistronic transcripts
introns

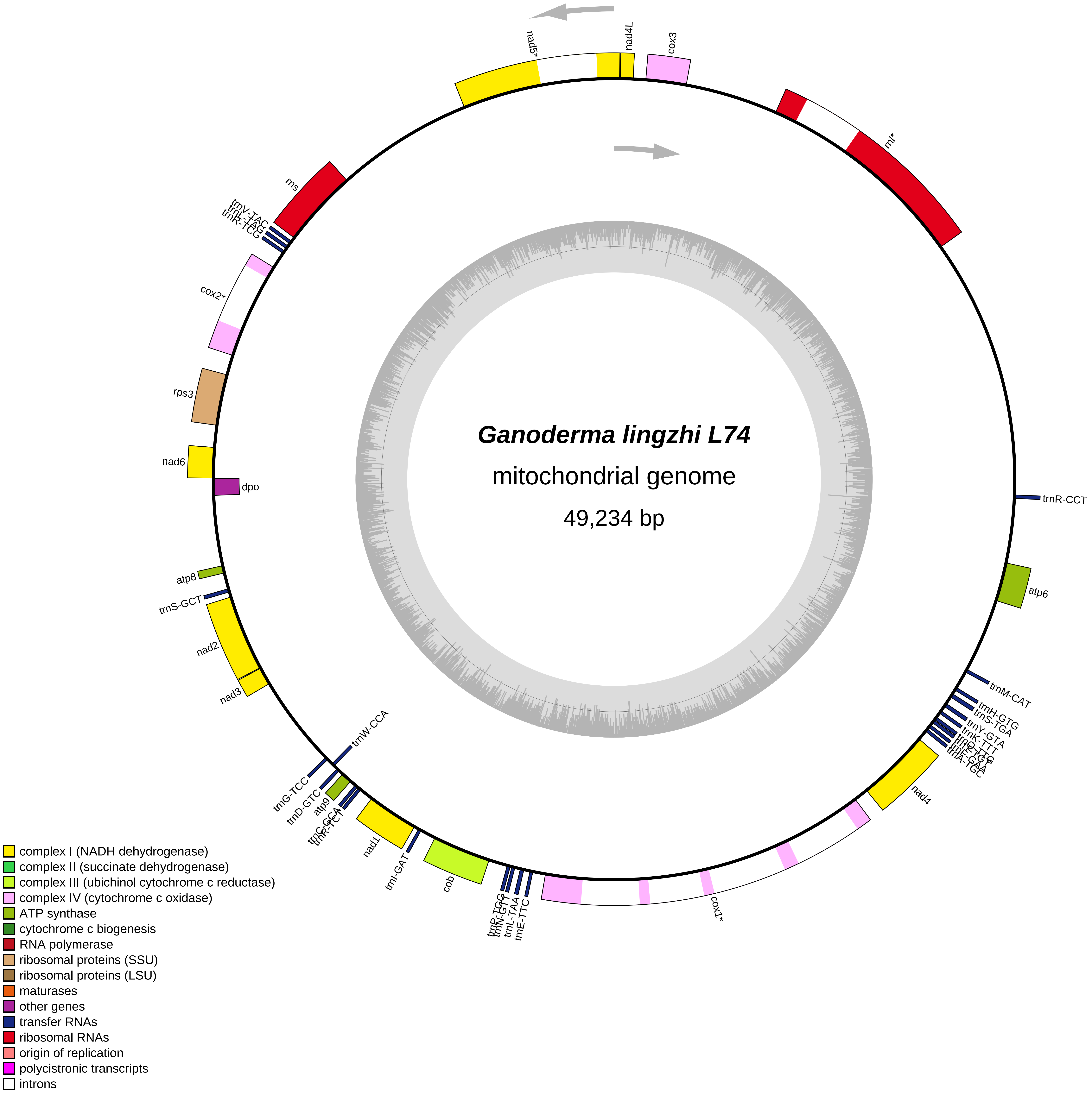

Ganoderma lingzhi L74
mitochondrial genome
49,234 bp
nad5*
nad4L
cox3
rnl*
rns
trnV-TAC
cox2*
rps3
nad6
dpo
trnR-CCT
atp6
atp8
trnS-GCT
nad2
nad3
trnM-CAT
trnH-GTG
trnS-TGA
trnY-GTA
trnK-TTT
trnA-TGC
nad4
trnW-CCA
trnG-TCC
trnD-GTC
atp9
nad1
trnI-GAT
cob
trnL-TAA
trnE-TTC
cox1*
complex I (NADH dehydrogenase)
complex II (succinate dehydrogenase)
complex III (ubichinol cytochrome c reductase)
complex IV (cytochrome c oxidase)
ATP synthase
cytochrome c biogenesis
RNA polymerase
ribosomal proteins (SSU)
ribosomal proteins (LSU)
maturases
other genes
transfer RNAs
ribosomal RNAs
origin of replication
polycistronic transcripts
introns

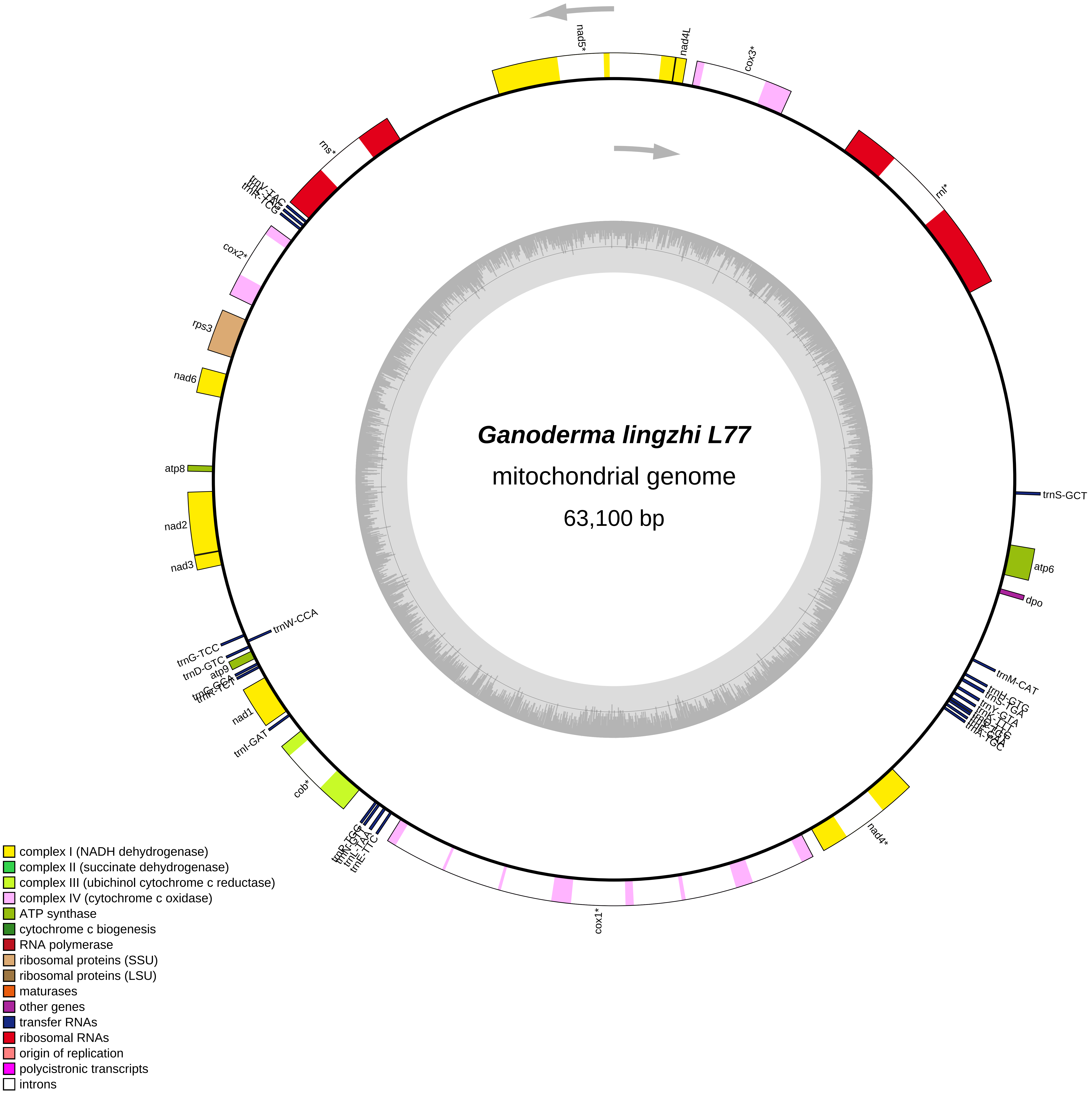
Ganoderma lingzhi L77
mitochondrial genome
63,100 bp
nad5*
nad4L
cox3*
rnl*
rns*
trnV-TAC
trnR-TCG
cox2*
rps3
nad6
atp8
nad2
nad3
trnW-CCA
trnG-TCC
trnD-GTC
atp9
trnC-GCA
trnR-TCT
nad1
trnI-GAT
cob*
trnP-TGG
trnL-TAA
trnE-TTC
cox1*
nad4*
trnS-GCT
atp6
dpo
trnM-CAT
trnH-GTG
trnS-TGA
trnY-GTA
complex I (NADH dehydrogenase)
complex II (succinate dehydrogenase)
complex III (ubichinol cytochrome c reductase)
complex IV (cytochrome c oxidase)
ATP synthase
cytochrome c biogenesis
RNA polymerase
ribosomal proteins (SSU)
ribosomal proteins (LSU)
maturases
other genes
transfer RNAs
ribosomal RNAs
origin of replication
polycistronic transcripts
introns

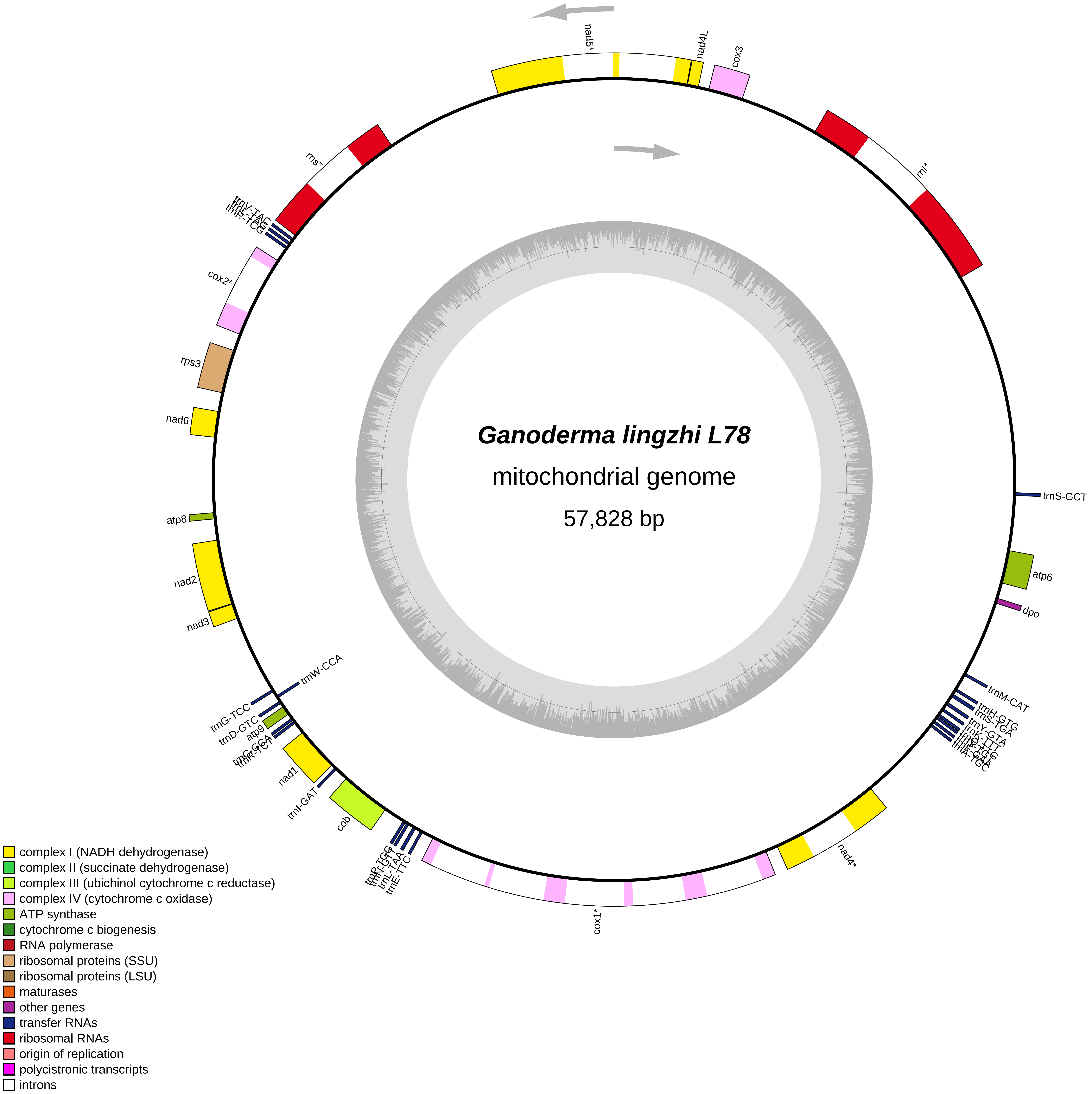

Ganoderma lingzhi L78
mitochondrial genome
57,828 bp
nad5*
nad4L
cox3
rnl*
rns*
cox2*
rps3
nad6
atp8
nad2
nad3
trnW-CCA
trnG-TCC
trnD-GTC
atp9
nad1
trnI-GAT
cob
trnE-TTC
cox1*
nad4*
trnS-GCT
atp6
dpo
trnM-CAT
trnH-GTG
trnS-TGA
trnY-GTA
trnA-TGC
complex I (NADH dehydrogenase)
complex II (succinate dehydrogenase)
complex III (ubichinol cytochrome c reductase)
complex IV (cytochrome c oxidase)
ATP synthase
cytochrome c biogenesis
RNA polymerase
ribosomal proteins (SSU)
ribosomal proteins (LSU)
maturases
other genes
transfer RNAs
ribosomal RNAs
origin of replication
polycistronic transcripts
introns

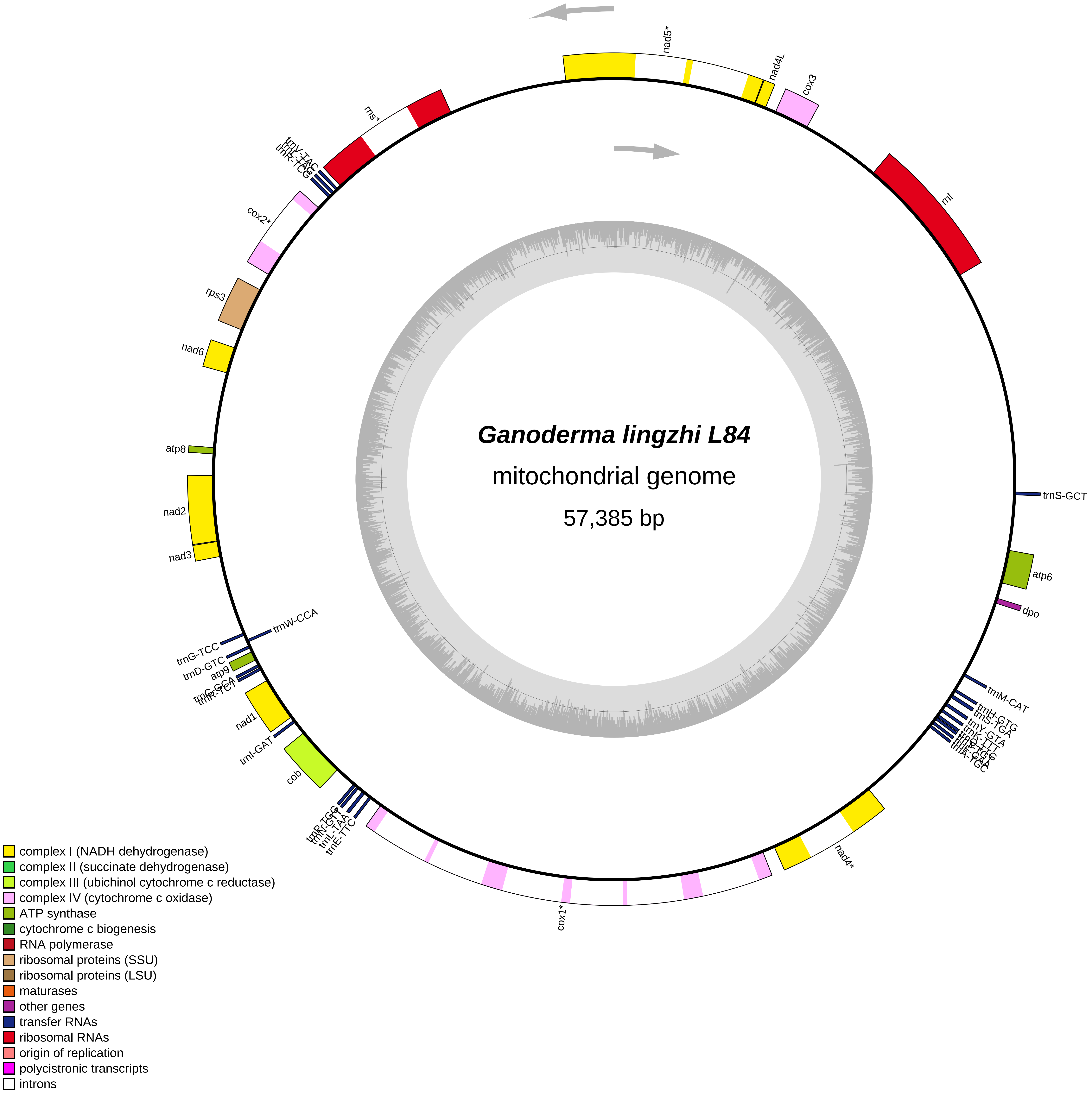
Ganoderma lingzhi L84
mitochondrial genome
57,385 bp
nad5*
nad4L
cox3
rnl
trnS-GCT
atp6
dpo
trnM-CAT
trnH-GTG
trnS-TGA
trnY-GTA
nad4*
cox1*
trnE-TTC
trnL-TAA
cob
trnI-GAT
nad1
trnR-TCT
atp9
trnD-GTC
trnG-TCC
trnW-CCA
nad3
nad2
atp8
nad6
rps3
cox2*
rns*
trnV-TAC
complex I (NADH dehydrogenase)
complex II (succinate dehydrogenase)
complex III (ubichinol cytochrome c reductase)
complex IV (cytochrome c oxidase)
ATP synthase
cytochrome c biogenesis
RNA polymerase
ribosomal proteins (SSU)
ribosomal proteins (LSU)
maturases
other genes
transfer RNAs
ribosomal RNAs
origin of replication
polycistronic transcripts
introns

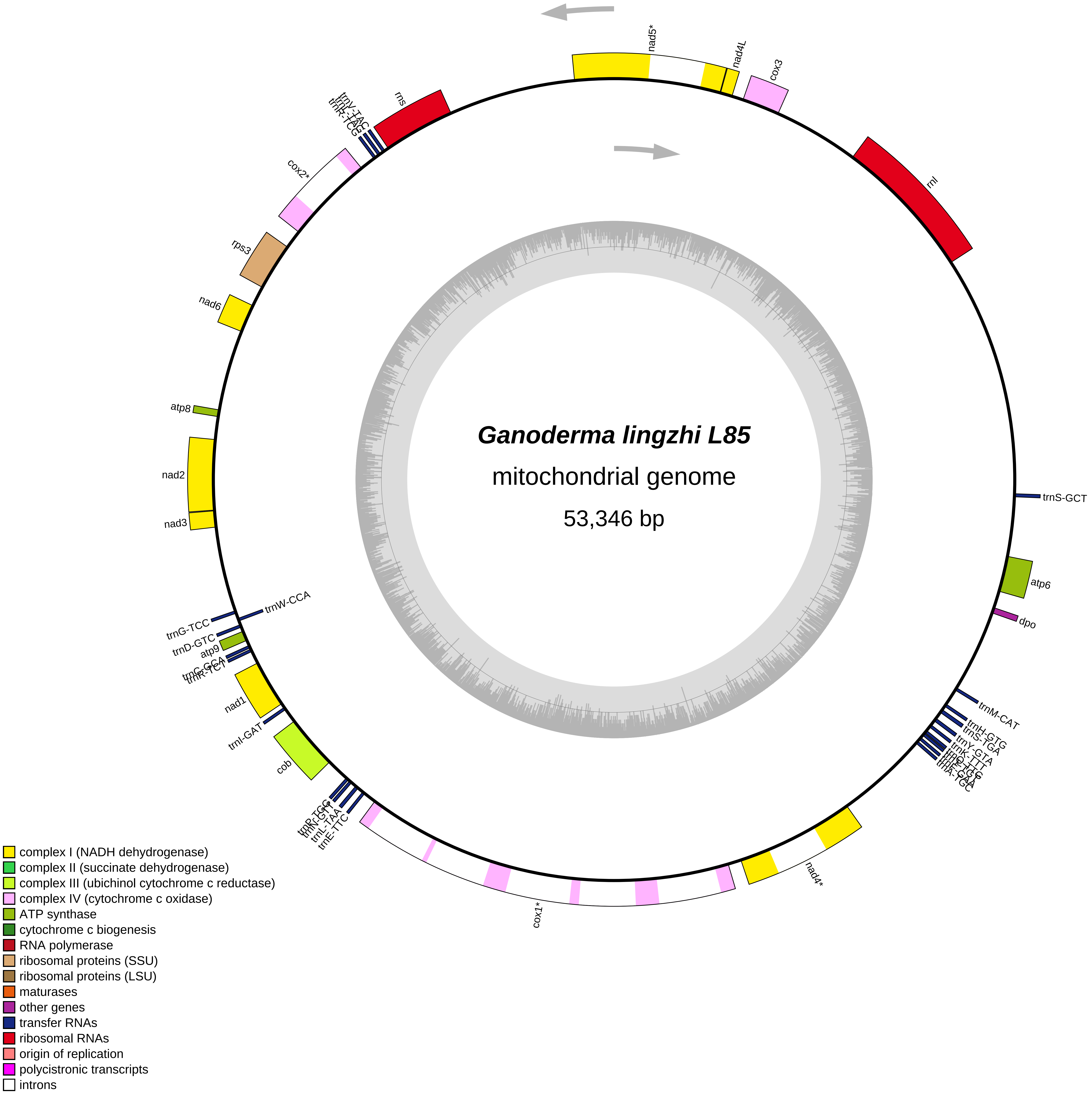

Ganoderma lingzhi L85
mitochondrial genome
53,346 bp
nad5*
nad4L
cox3
rnl
trnS-GCT
atp6
dpo
trnM-CAT
trnH-GTG
trnS-TGA
trnY-GTA
nad4*
cox1*
trnE-TTC
trnL-TAA
cob
trnI-GAT
nad1
trnR-TCT
atp9
trnD-GTC
trnG-TCC
trnW-CCA
nad3
nad2
atp8
nad6
rps3
cox2*
rns
trnV-TAC
complex I (NADH dehydrogenase)
complex II (succinate dehydrogenase)
complex III (ubichinol cytochrome c reductase)
complex IV (cytochrome c oxidase)
ATP synthase
cytochrome c biogenesis
RNA polymerase
ribosomal proteins (SSU)
ribosomal proteins (LSU)
maturases
other genes
transfer RNAs
ribosomal RNAs
origin of replication
polycistronic transcripts
introns

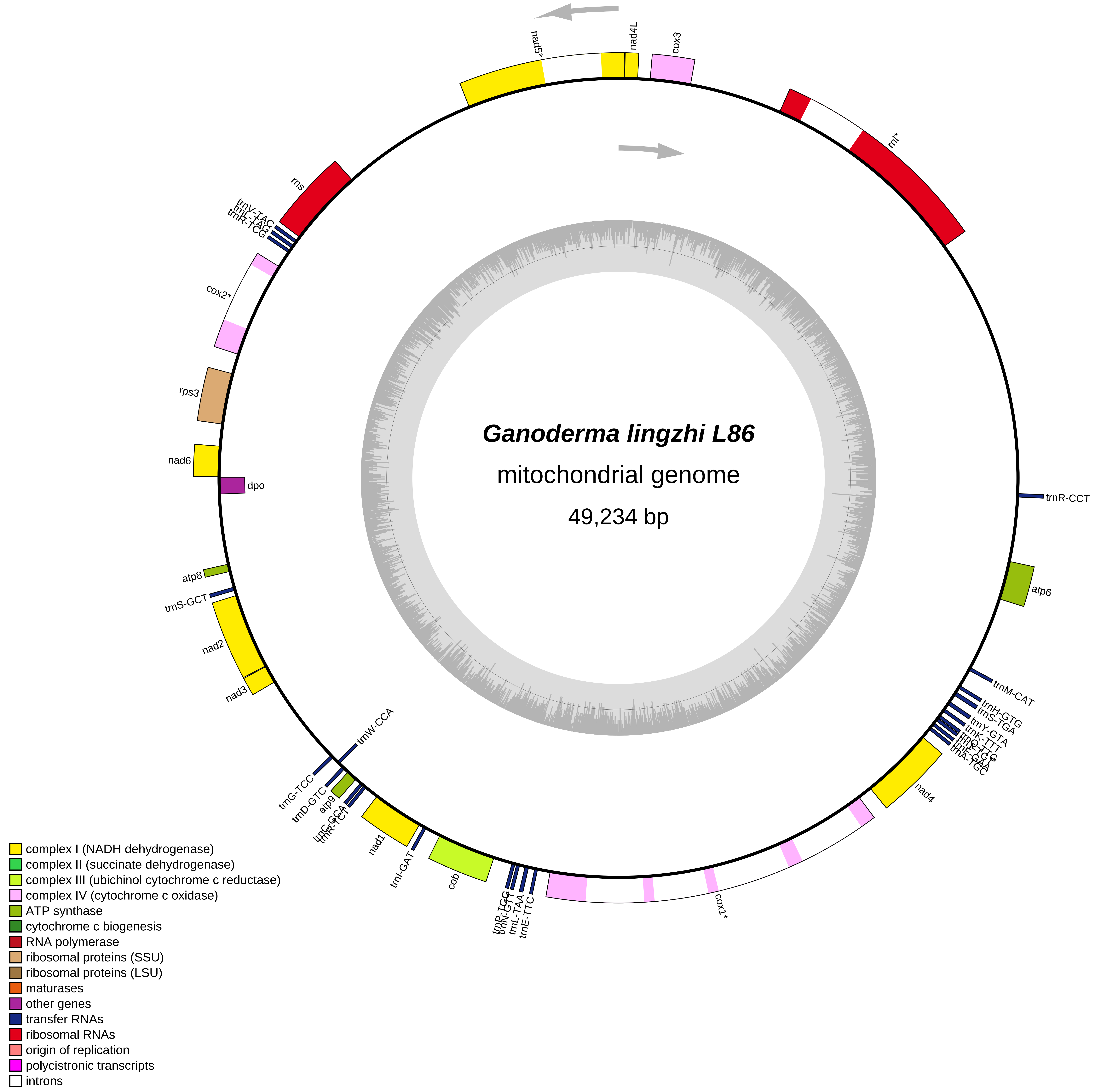

Ganoderma lingzhi L86
mitochondrial genome
49,234 bp
nad5*
nad4L
cox3
rnl*
rns
trnV-TAC
trnL-TAG
trnR-TCG
cox2*
rps3
nad6
dpo
trnR-CCT
atp6
atp8
trnS-GCT
nad2
nad3
trnM-CAT
trnH-GTG
trnS-TGA
trnY-GTA
trnK-TTT
trnA-TGC
nad4
trnW-CCA
trnG-TCC
trnD-GTC
atp9
trnC-GCA
trnR-TCT
nad1
trnI-GAT
cob
trnP-TGG
trnN-GTT
trnL-TAA
trnE-TTC
cox1*
complex I (NADH dehydrogenase)
complex II (succinate dehydrogenase)
complex III (ubichinol cytochrome c reductase)
complex IV (cytochrome c oxidase)
ATP synthase
cytochrome c biogenesis
RNA polymerase
ribosomal proteins (SSU)
ribosomal proteins (LSU)
maturases
other genes
transfer RNAs
ribosomal RNAs
origin of replication
polycistronic transcripts
introns

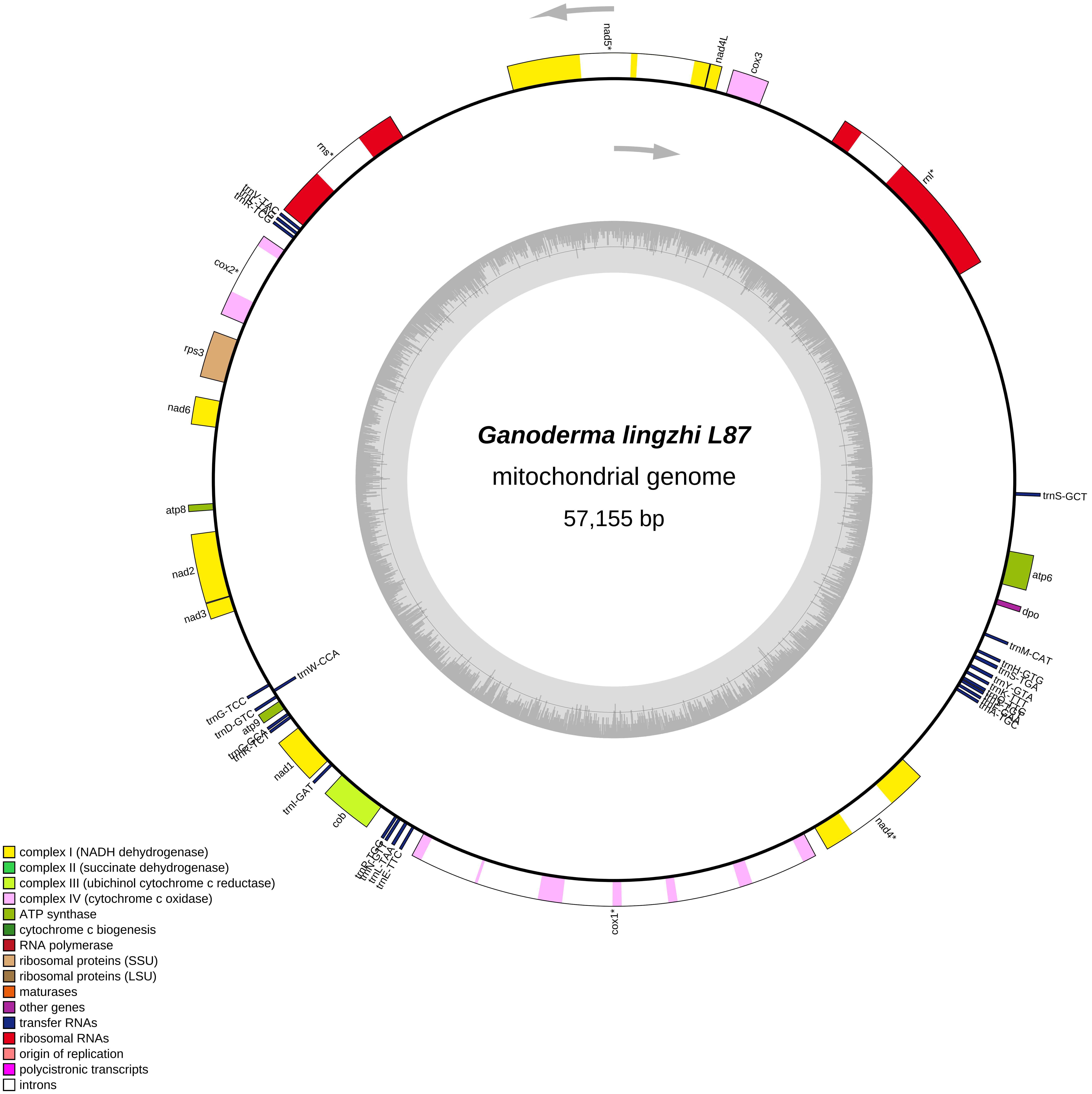
Ganoderma lingzhi L87
mitochondrial genome
57,155 bp
nad5*
nad4L
cox3
rnl*
trnS-GCT
atp6
dpo
trnM-CAT
trnH-GTG
trnS-TGA
trnY-GTA
nad4*
cox1*
trnL-TAA
trnE-TTC
cob
trnI-GAT
nad1
atp9
trnD-GTC
trnG-TCC
trnW-CCA
nad3
nad2
atp8
nad6
rps3
cox2*
trnV-TAC
rns*
complex I (NADH dehydrogenase)
complex II (succinate dehydrogenase)
complex III (ubichinol cytochrome c reductase)
complex IV (cytochrome c oxidase)
ATP synthase
cytochrome c biogenesis
RNA polymerase
ribosomal proteins (SSU)
ribosomal proteins (LSU)
maturases
other genes
transfer RNAs
ribosomal RNAs
origin of replication
polycistronic transcripts
introns

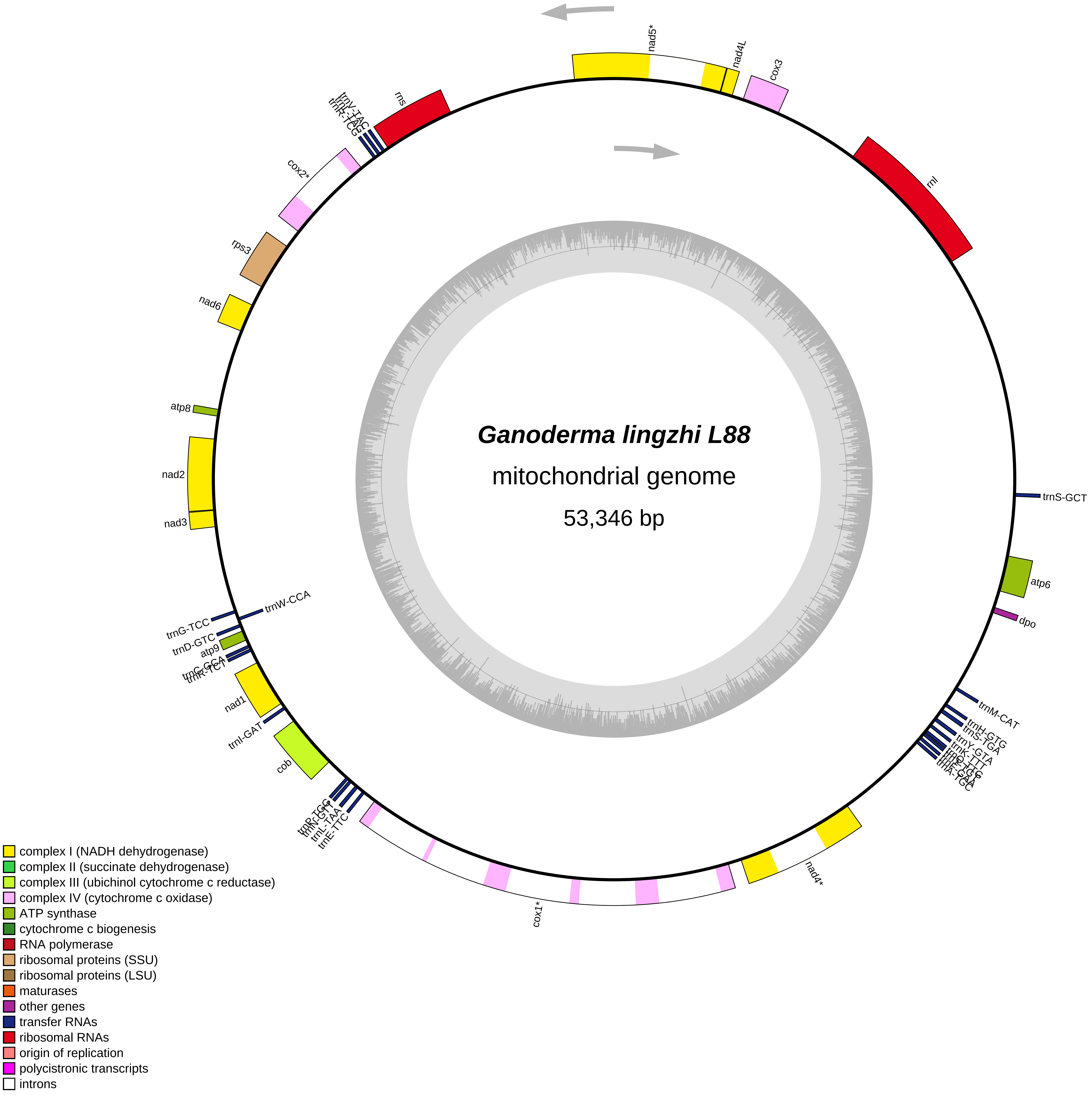
Ganoderma lingzhi L88
mitochondrial genome
53,346 bp
nad5*
nad4L
cox3
rnl
trnS-GCT
atp6
dpo
trnM-CAT
trnH-GTG
trnS-TGA
trnY-GTA
trnK-TTT
nad4*
cox1*
trnE-TTC
trnL-TAA
cob
trnI-GAT
nad1
trnR-TCT
atp9
trnD-GTC
trnG-TCC
trnW-CCA
nad3
nad2
atp8
nad6
rps3
cox2*
rns
trnV-TAC
complex I (NADH dehydrogenase)
complex II (succinate dehydrogenase)
complex III (ubichinol cytochrome c reductase)
complex IV (cytochrome c oxidase)
ATP synthase
cytochrome c biogenesis
RNA polymerase
ribosomal proteins (SSU)
ribosomal proteins (LSU)
maturases
other genes
transfer RNAs
ribosomal RNAs
origin of replication
polycistronic transcripts
introns

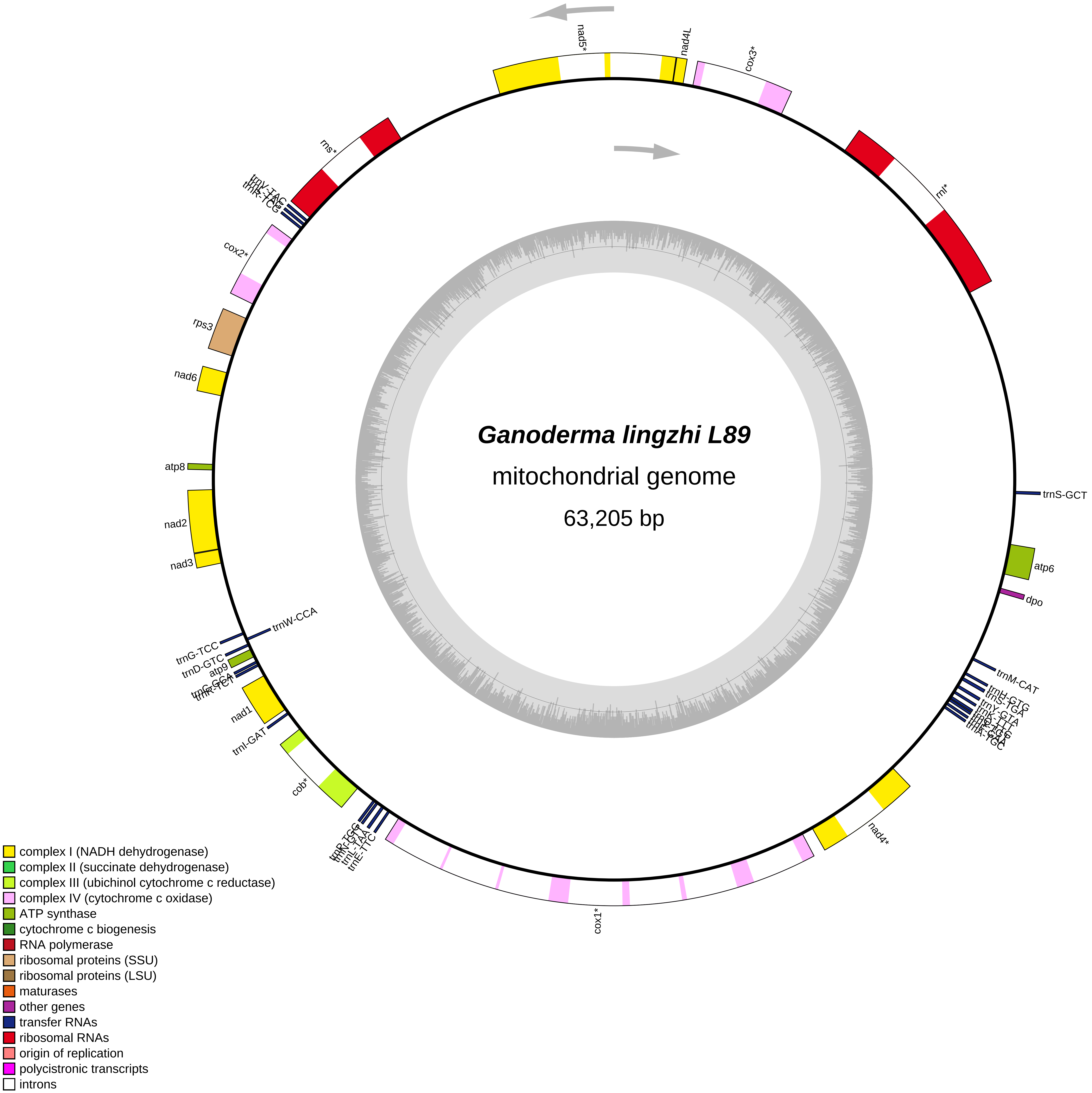
Ganoderma lingzhi L89
mitochondrial genome
63,205 bp
nad5*
nad4L
cox3*
rnl*
trnS-GCT
atp6
dpo
trnM-CAT
trnH-GTG
trnS-TGA
trnY-GTA
nad4*
cox1*
trnE-TTC
cob*
trnI-GAT
nad1
trnR-TCT
atp9
trnD-GTC
trnG-TCC
trnW-CCA
nad3
nad2
atp8
nad6
rps3
cox2*
trnR-TCG
trnV-TAC
rns*
complex I (NADH dehydrogenase)
complex II (succinate dehydrogenase)
complex III (ubichinol cytochrome c reductase)
complex IV (cytochrome c oxidase)
ATP synthase
cytochrome c biogenesis
RNA polymerase
ribosomal proteins (SSU)
ribosomal proteins (LSU)
maturases
other genes
transfer RNAs
ribosomal RNAs
origin of replication
polycistronic transcripts
introns

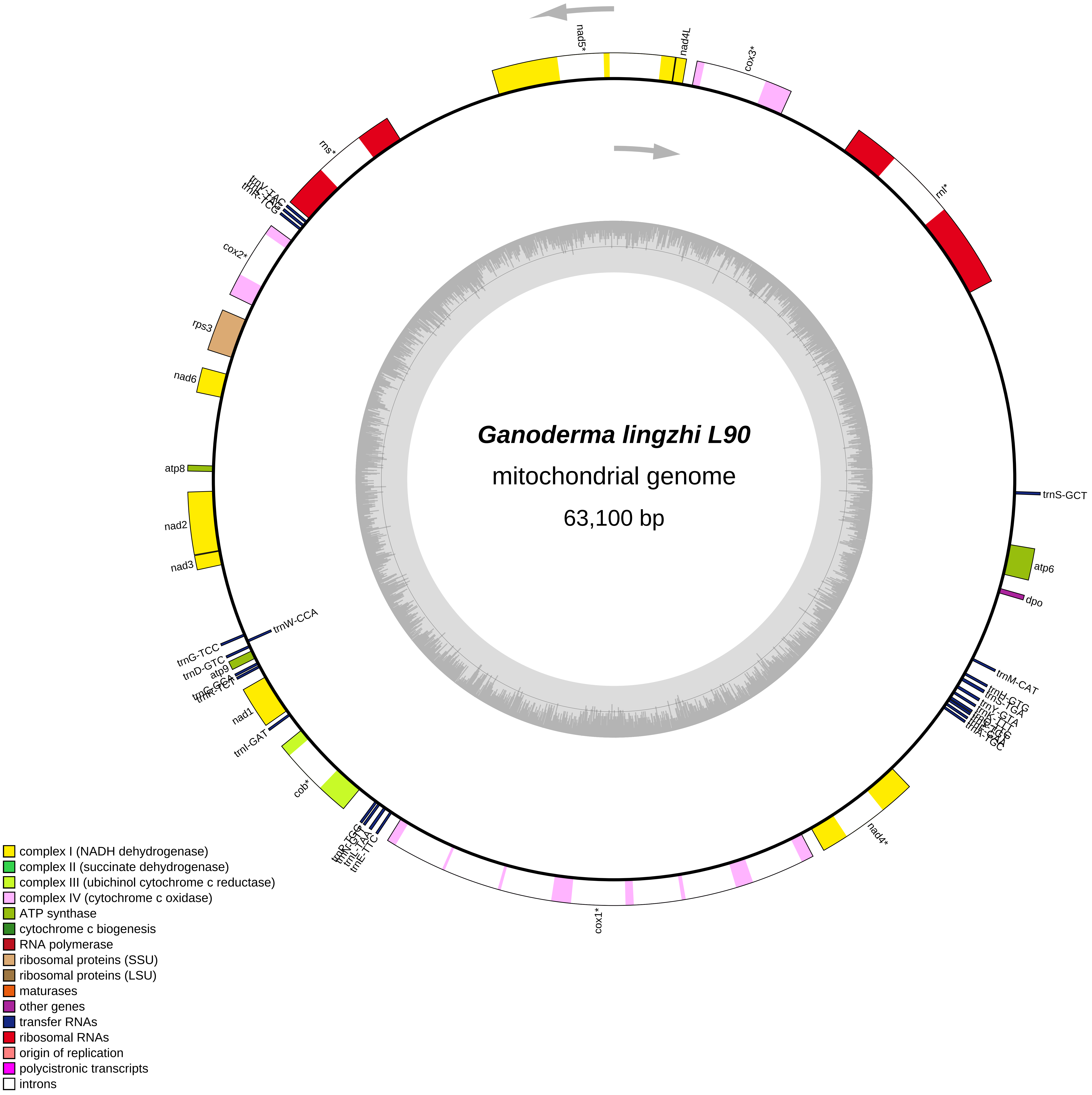

Ganoderma lingzhi L90
mitochondrial genome
63,100 bp
nad5*
nad4L
cox3*
rnl*
trnS-GCT
atp6
dpo
trnM-CAT
trnH-GTG
trnS-TGA
nad4*
cox1*
trnE-TTC
cob*
trnI-GAT
nad1
atp9
trnD-GTC
trnG-TCC
trnW-CCA
nad3
nad2
atp8
nad6
rps3
cox2*
trnV-TAC
rns*
complex I (NADH dehydrogenase)
complex II (succinate dehydrogenase)
complex III (ubichinol cytochrome c reductase)
complex IV (cytochrome c oxidase)
ATP synthase
cytochrome c biogenesis
RNA polymerase
ribosomal proteins (SSU)
ribosomal proteins (LSU)
maturases
other genes
transfer RNAs
ribosomal RNAs
origin of replication
polycistronic transcripts
introns

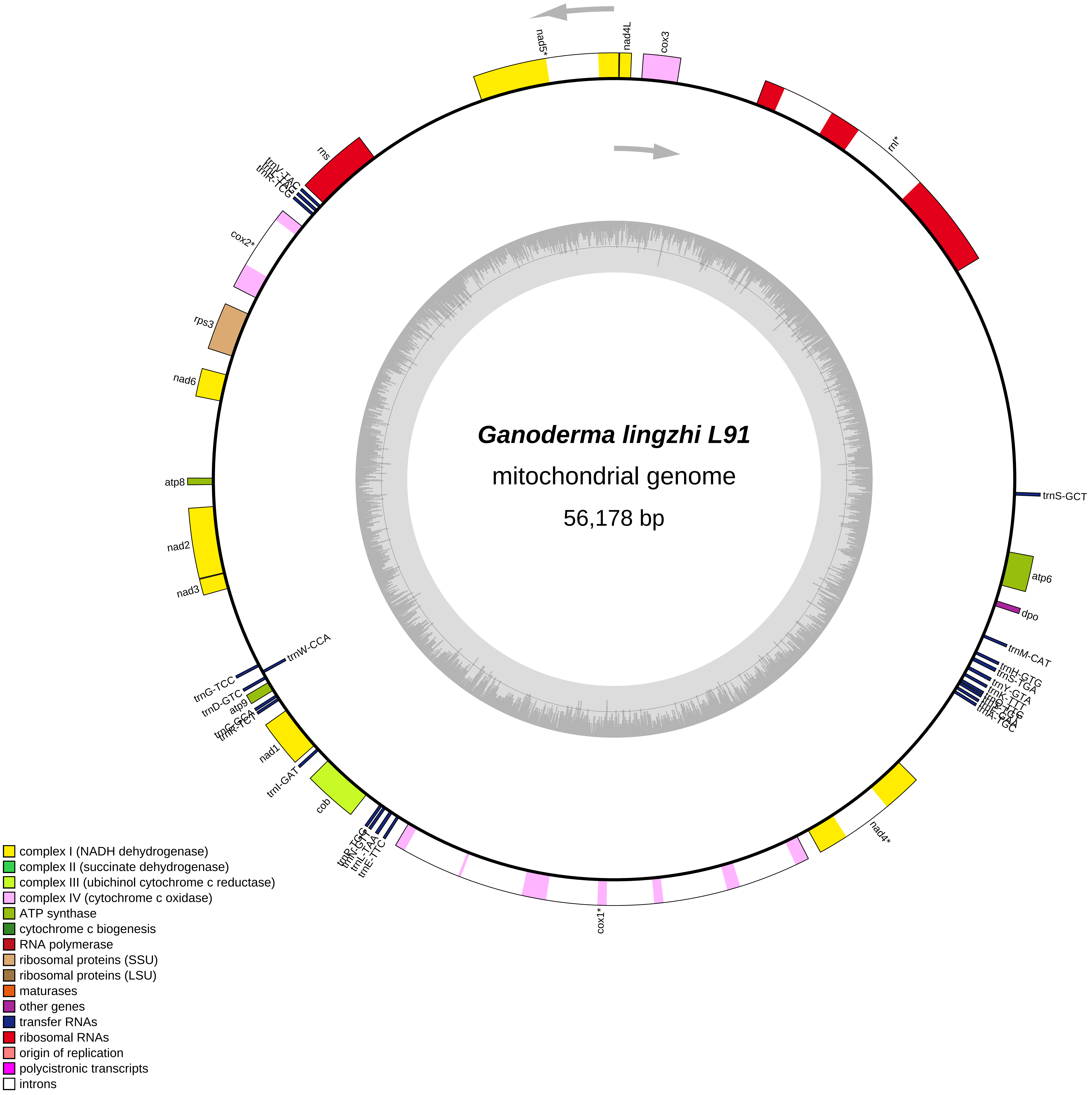

Ganoderma lingzhi L91
mitochondrial genome
56,178 bp
nad5*
nad4L
cox3
rnl*
rns
cox2*
rps3
nad6
atp8
nad2
nad3
trnW-CCA
trnG-TCC
trnD-GTC
atp9
nad1
trnI-GAT
cob
cox1*
nad4*
trnS-GCT
atp6
dpo
trnM-CAT
trnH-GTG
trnS-TGA
trnY-GTA
trnA-TGC
trnE-TTC
trnL-TAA
complex I (NADH dehydrogenase)
complex II (succinate dehydrogenase)
complex III (ubichinol cytochrome c reductase)
complex IV (cytochrome c oxidase)
ATP synthase
cytochrome c biogenesis
RNA polymerase
ribosomal proteins (SSU)
ribosomal proteins (LSU)
maturases
other genes
transfer RNAs
ribosomal RNAs
origin of replication
polycistronic transcripts
introns

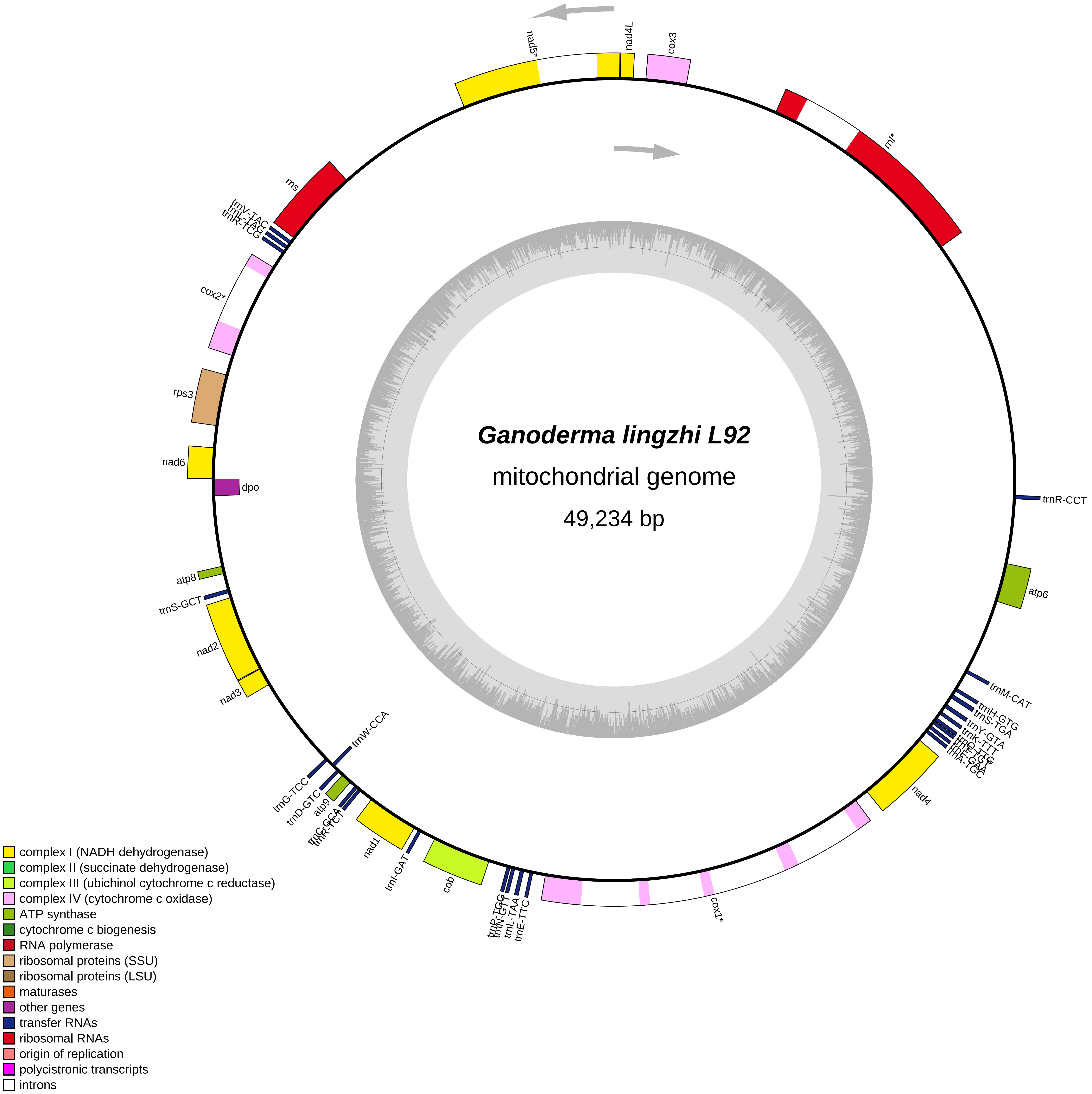

Ganoderma lingzhi L92
mitochondrial genome
49,234 bp
nad5*
nad4L
cox3
rnl*
rns
trnV-TAC
cox2*
rps3
nad6
dpo
trnR-CCT
atp6
atp8
trnS-GCT
nad2
nad3
trnM-CAT
trnH-GTG
trnS-TGA
trnY-GTA
trnK-TTT
trnA-TGC
nad4
cox1*
trnE-TTC
trnL-TAA
trnN-GTT
trnP-TGG
cob
trnI-GAT
nad1
trnC-GCA
atp9
trnD-GTC
trnG-TCC
trnW-CCA
complex I (NADH dehydrogenase)
complex II (succinate dehydrogenase)
complex III (ubichinol cytochrome c reductase)
complex IV (cytochrome c oxidase)
ATP synthase
cytochrome c biogenesis
RNA polymerase
ribosomal proteins (SSU)
ribosomal proteins (LSU)
maturases
other genes
transfer RNAs
ribosomal RNAs
origin of replication
polycistronic transcripts
introns

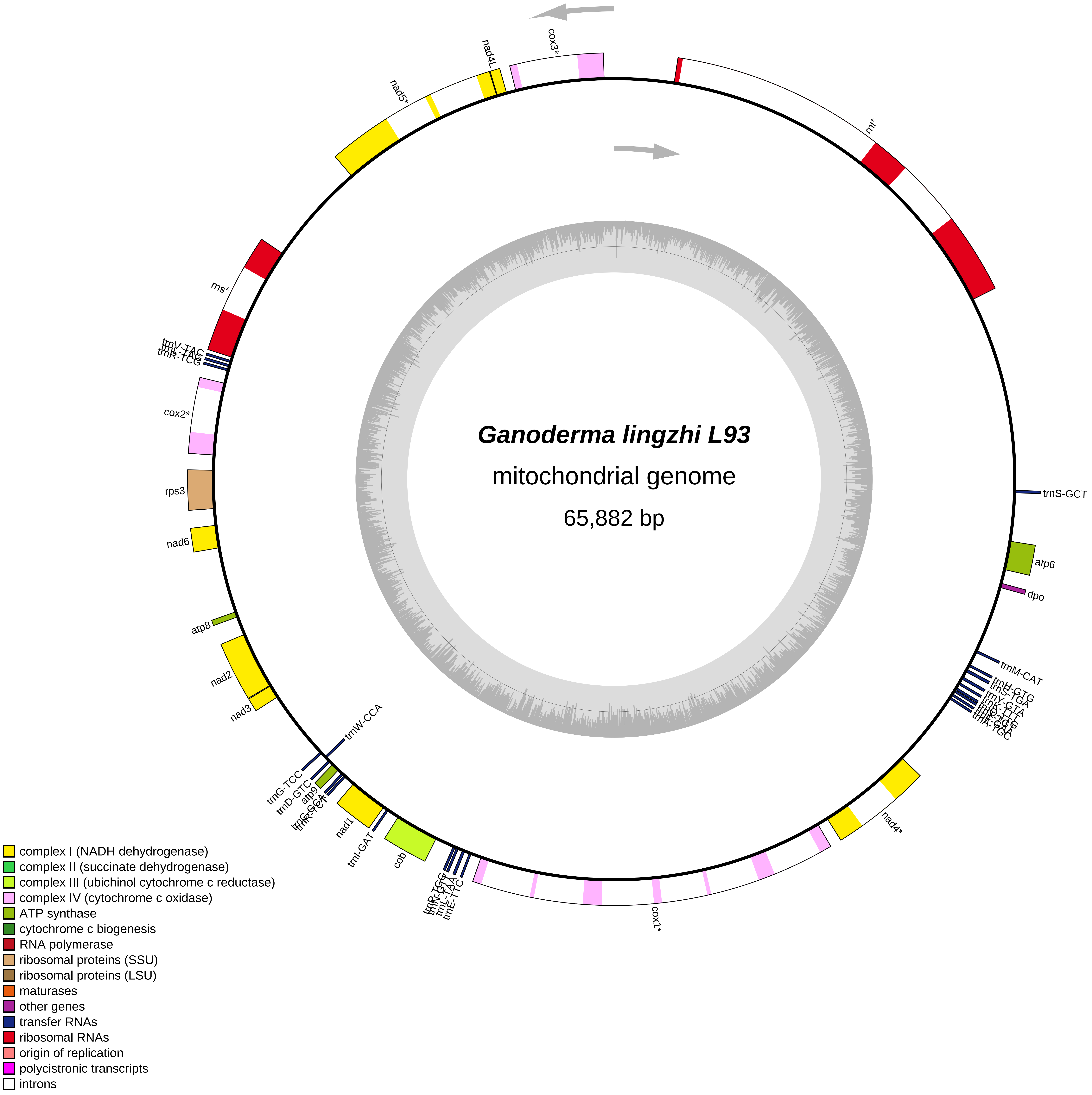
Ganoderma lingzhi L93
mitochondrial genome
65,882 bp
cox3*
nad4L
nad5*
rnl*
rns*
trnV-TAC
trnR-TCG
cox2*
rps3
nad6
atp8
nad2
nad3
trnW-CCA
trnG-TCC
trnD-GTC
atp9
nad1
trnI-GAT
cob
trnE-TTC
cox1*
nad4*
trnM-CAT
trnH-GTG
trnS-GCT
atp6
dpo
complex I (NADH dehydrogenase)
complex II (succinate dehydrogenase)
complex III (ubichinol cytochrome c reductase)
complex IV (cytochrome c oxidase)
ATP synthase
cytochrome c biogenesis
RNA polymerase
ribosomal proteins (SSU)
ribosomal proteins (LSU)
maturases
other genes
transfer RNAs
ribosomal RNAs
origin of replication
polycistronic transcripts
introns

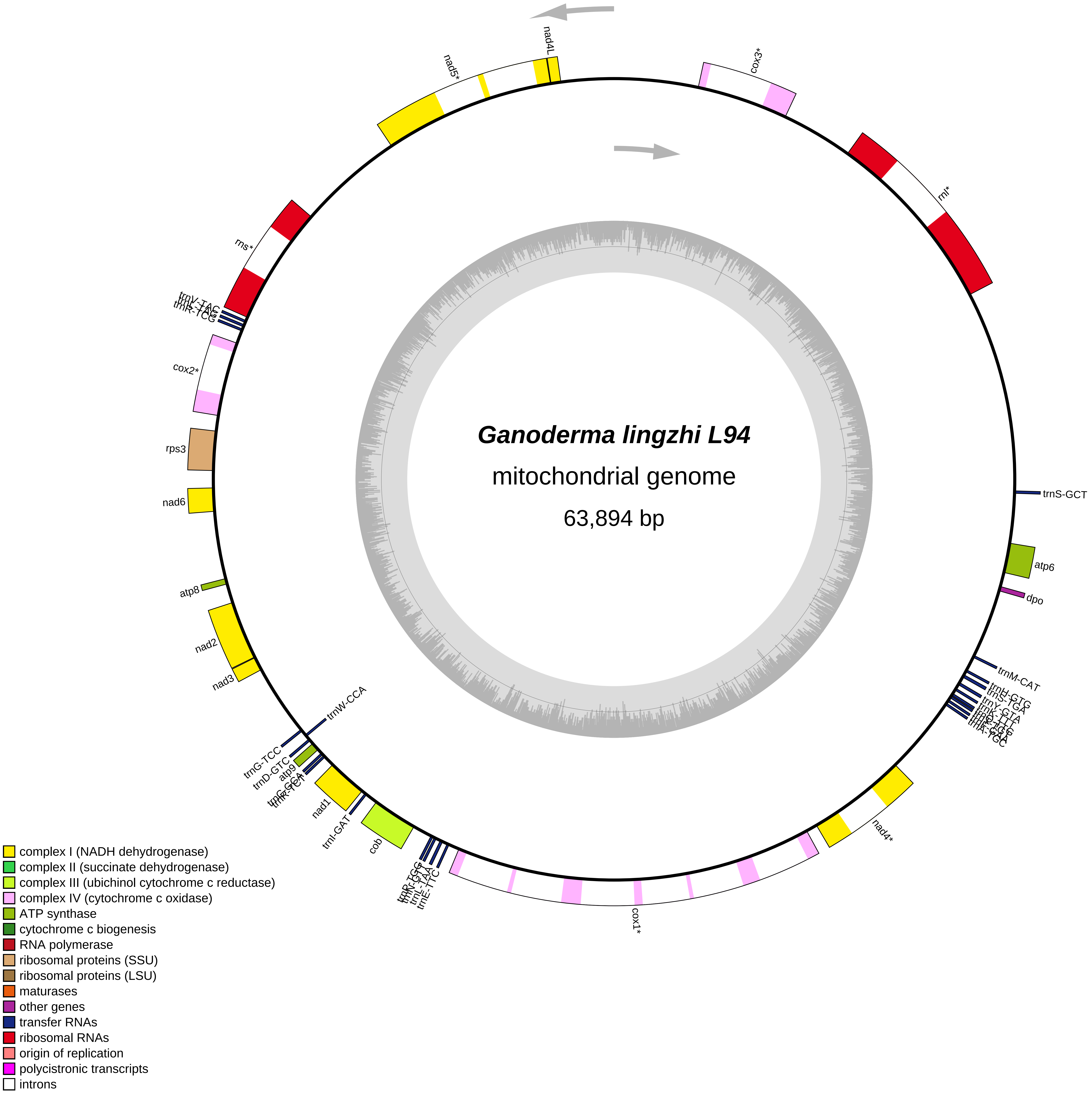

Ganoderma lingzhi L94
mitochondrial genome
63,894 bp
nad4L
nad5*
cox3*
rnl*
rns*
cox2*
rps3
nad6
atp8
nad2
nad3
trnW-CCA
trnG-TCC
trnD-GTC
atp9
nad1
trnI-GAT
cob
cox1*
nad4*
trnS-GCT
atp6
dpo
trnM-CAT
trnH-GTG
trnS-TGA
trnY-GTA
trnE-TTC
trnL-TAA
trnR-TCG
trnV-TAC
complex I (NADH dehydrogenase)
complex II (succinate dehydrogenase)
complex III (ubichinol cytochrome c reductase)
complex IV (cytochrome c oxidase)
ATP synthase
cytochrome c biogenesis
RNA polymerase
ribosomal proteins (SSU)
ribosomal proteins (LSU)
maturases
other genes
transfer RNAs
ribosomal RNAs
origin of replication
polycistronic transcripts
introns

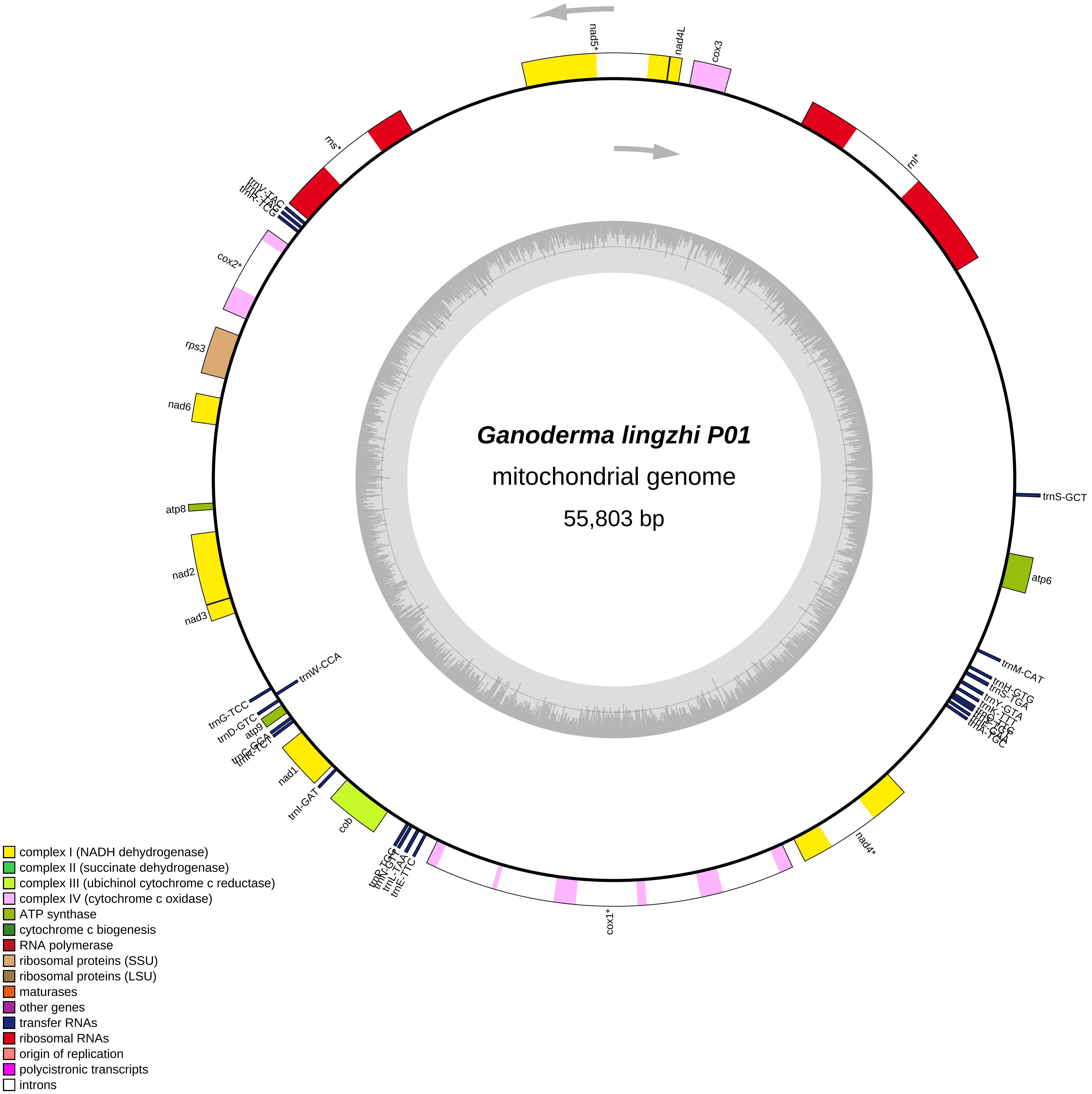
Ganoderma lingzhi P01
mitochondrial genome
55,803 bp
nad5*
nad4L
cox3
rnl*
trnS-GCT
atp6
trnM-CAT
trnH-GTG
trnS-TGA
trnY-GTA
nad4*
cox1*
trnP-TGG
trnN-GTT
trnL-TAA
trnE-TTC
cob
trnI-GAT
nad1
trnR-TCT
trnC-GCA
atp9
trnD-GTC
trnG-TCC
trnW-CCA
nad3
nad2
atp8
nad6
rps3
cox2*
trnR-TCG
trnV-TAC
rns*
complex I (NADH dehydrogenase)
complex II (succinate dehydrogenase)
complex III (ubichinol cytochrome c reductase)
complex IV (cytochrome c oxidase)
ATP synthase
cytochrome c biogenesis
RNA polymerase
ribosomal proteins (SSU)
ribosomal proteins (LSU)
maturases
other genes
transfer RNAs
ribosomal RNAs
origin of replication
polycistronic transcripts
introns

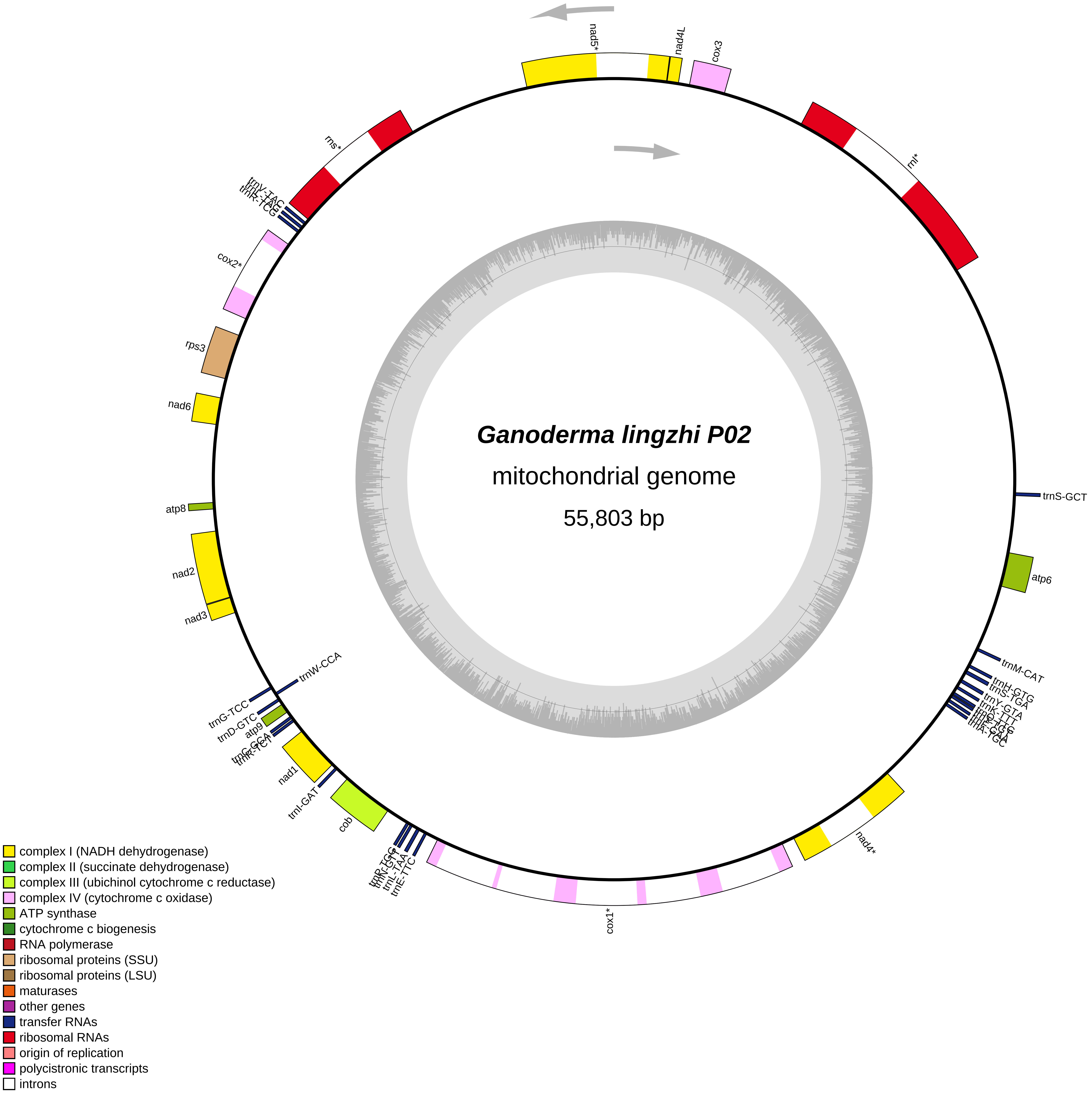

Ganoderma lingzhi P02
mitochondrial genome
55,803 bp
nad5*
nad4L
cox3
rnl*
trnS-GCT
atp6
trnM-CAT
trnH-GTG
trnS-TGA
trnY-GTA
nad4*
cox1*
trnE-TTC
trnL-TAA
cob
trnI-GAT
nad1
trnR-TCT
atp9
trnD-GTC
trnG-TCC
trnW-CCA
nad3
nad2
atp8
nad6
rps3
cox2*
trnR-TCG
trnV-TAC
rns*
complex I (NADH dehydrogenase)
complex II (succinate dehydrogenase)
complex III (ubichinol cytochrome c reductase)
complex IV (cytochrome c oxidase)
ATP synthase
cytochrome c biogenesis
RNA polymerase
ribosomal proteins (SSU)
ribosomal proteins (LSU)
maturases
other genes
transfer RNAs
ribosomal RNAs
origin of replication
polycistronic transcripts
introns

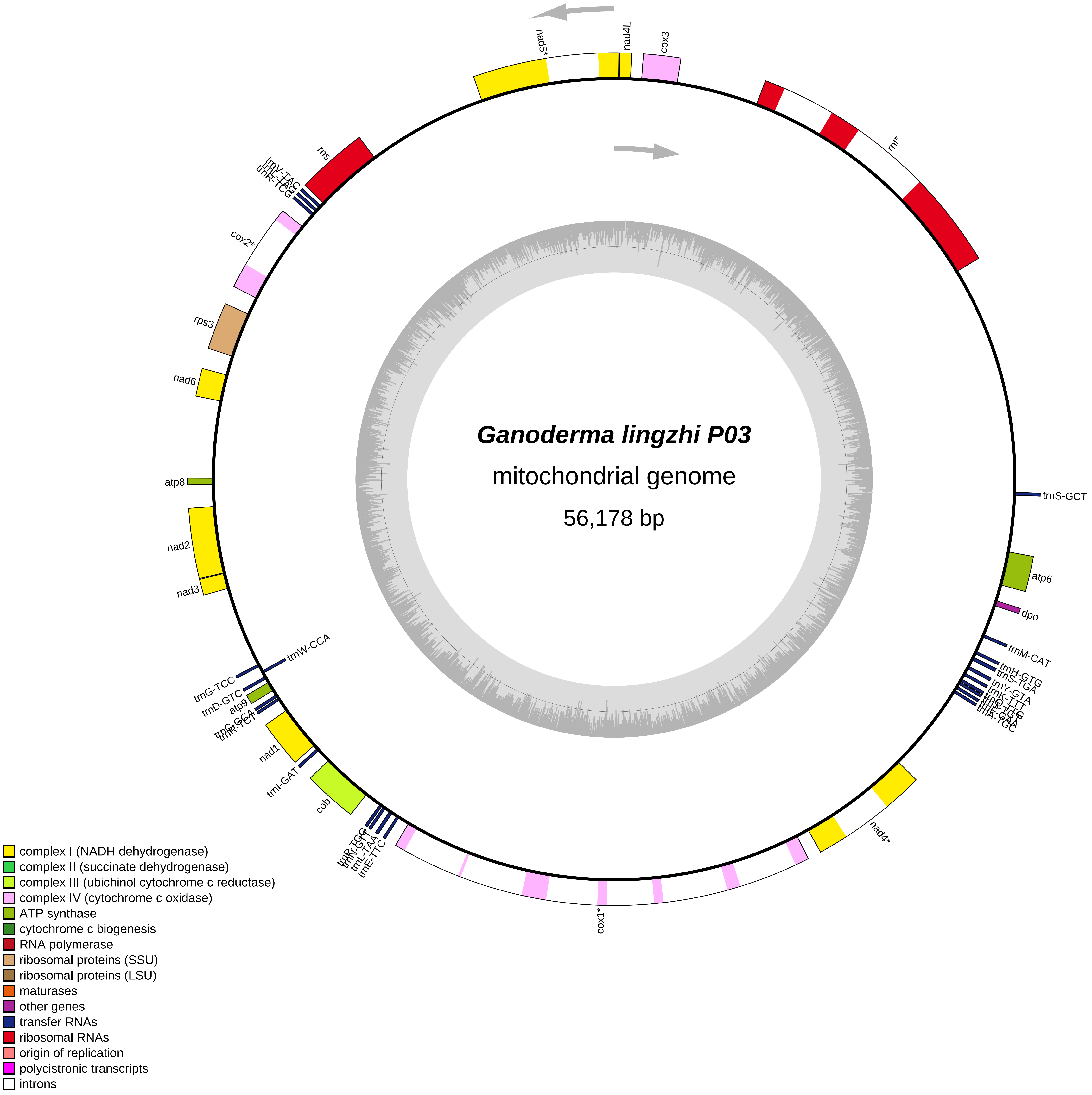

Ganoderma lingzhi P03
mitochondrial genome
56,178 bp
nad5*
nad4L
cox3
rnl*
rns
trnV-TAC
trnR-TCG
cox2*
rps3
nad6
atp8
nad2
nad3
trnW-CCA
trnG-TCC
trnD-GTC
atp9
trnC-GCA
trnR-TCT
nad1
trnI-GAT
cob
trnR-TCG
trnN-GTT
trnL-TAA
trnE-TTC
cox1*
nad4*
trnS-GCT
atp6
dpo
trnM-CAT
trnH-GTG
trnS-TGA
trnY-GTA
trnK-TTT
trnA-TGC
complex I (NADH dehydrogenase)
complex II (succinate dehydrogenase)
complex III (ubichinol cytochrome c reductase)
complex IV (cytochrome c oxidase)
ATP synthase
cytochrome c biogenesis
RNA polymerase
ribosomal proteins (SSU)
ribosomal proteins (LSU)
maturases
other genes
transfer RNAs
ribosomal RNAs
origin of replication
polycistronic transcripts
introns

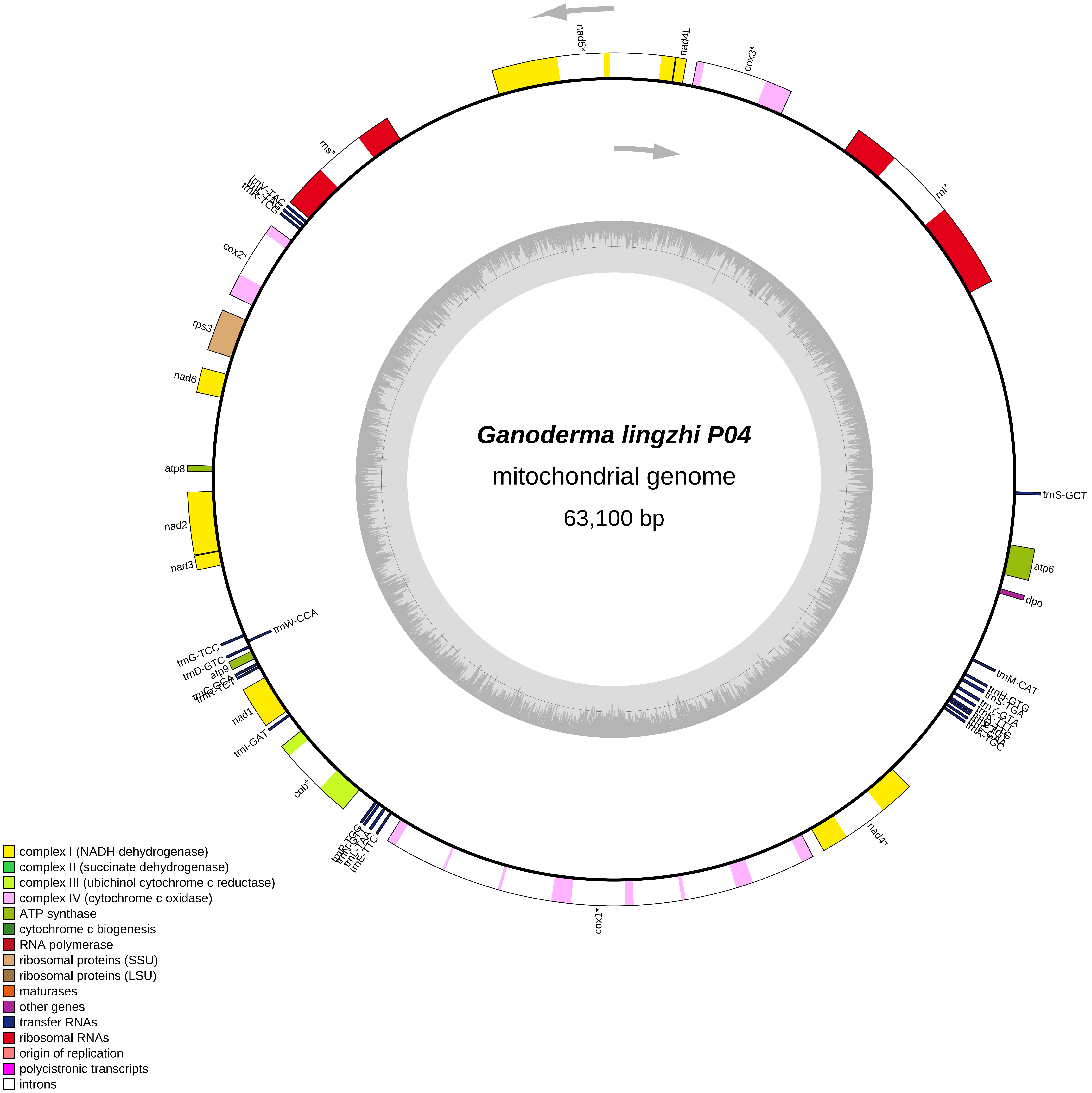
Ganoderma lingzhi P04
mitochondrial genome
63,100 bp
nad5*
nad4L
cox3*
rnl*
trnS-GCT
atp6
dpo
trnM-CAT
trnH-GTG
trnS-TGA
trnY-GTA
nad4*
cox1*
trnE-TTC
trnL-TAA
cob*
trnI-GAT
nad1
atp9
trnD-GTC
trnG-TCC
trnW-CCA
nad3
nad2
atp8
nad6
rps3
cox2*
rns*
trnV-TAC
complex I (NADH dehydrogenase)
complex II (succinate dehydrogenase)
complex III (ubichinol cytochrome c reductase)
complex IV (cytochrome c oxidase)
ATP synthase
cytochrome c biogenesis
RNA polymerase
ribosomal proteins (SSU)
ribosomal proteins (LSU)
maturases
other genes
transfer RNAs
ribosomal RNAs
origin of replication
polycistronic transcripts
introns

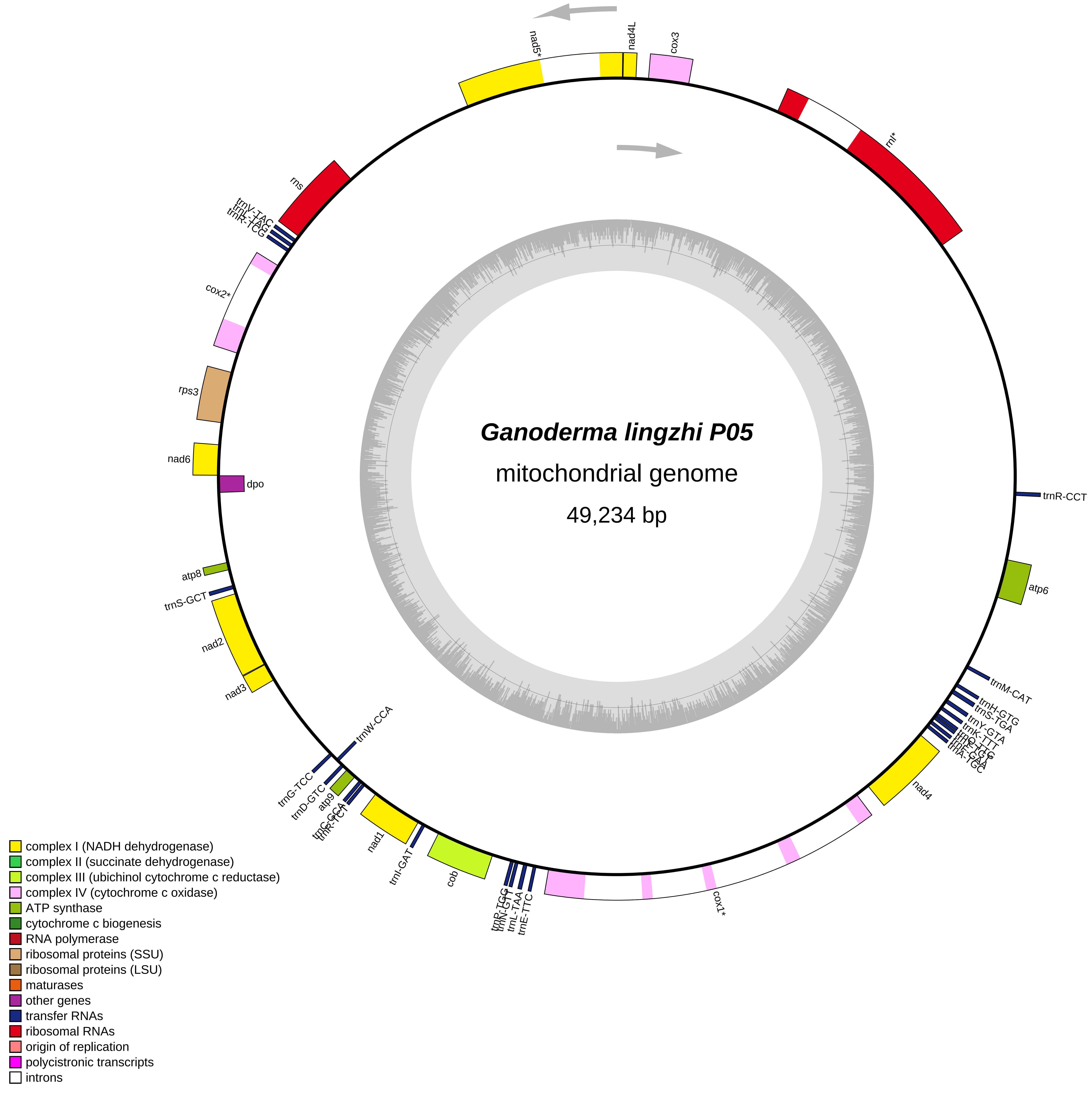

Ganoderma lingzhi P05
mitochondrial genome
49,234 bp
nad5*
nad4L
cox3
rnl*
rns
trnV-TAC
trnR-TCG
cox2*
rps3
nad6
dpo
trnR-CCT
atp8
trnS-GCT
atp6
nad2
nad3
trnM-CAT
trnH-GTG
trnS-TGA
trnY-GTA
trnK-TTT
trnA-TGC
nad4
trnW-CCA
trnG-TCC
trnD-GTC
atp9
trnC-GCA
nad1
trnI-GAT
cob
trnP-TGG
trnL-TAA
trnE-TTC
cox1*
complex I (NADH dehydrogenase)
complex II (succinate dehydrogenase)
complex III (ubichinol cytochrome c reductase)
complex IV (cytochrome c oxidase)
ATP synthase
cytochrome c biogenesis
RNA polymerase
ribosomal proteins (SSU)
ribosomal proteins (LSU)
maturases
other genes
transfer RNAs
ribosomal RNAs
origin of replication
polycistronic transcripts
introns

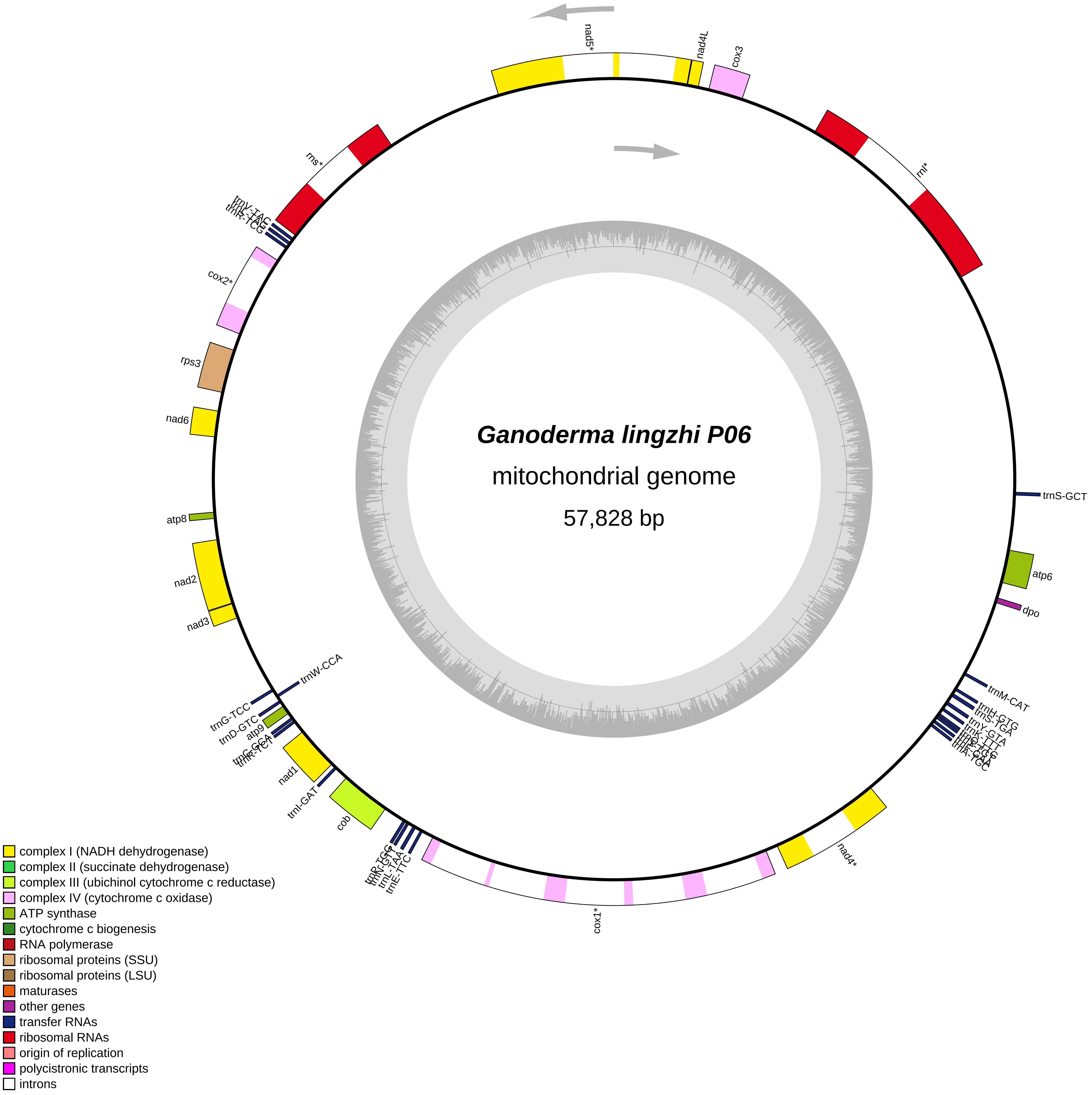
Ganoderma lingzhi P06
mitochondrial genome
57,828 bp
nad5*
nad4L
cox3
rnl*
trnS-GCT
atp6
dpo
trnM-CAT
trnH-GTG
trnS-TGA
trnY-GTA
nad4*
cox1*
trnE-TTC
trnL-TAA
cob
trnI-GAT
nad1
atp9
trnD-GTC
trnG-TCC
trnW-CCA
nad3
nad2
atp8
nad6
rps3
cox2*
trnV-TAC
rns*
complex I (NADH dehydrogenase)
complex II (succinate dehydrogenase)
complex III (ubichinol cytochrome c reductase)
complex IV (cytochrome c oxidase)
ATP synthase
cytochrome c biogenesis
RNA polymerase
ribosomal proteins (SSU)
ribosomal proteins (LSU)
maturases
other genes
transfer RNAs
ribosomal RNAs
origin of replication
polycistronic transcripts
introns

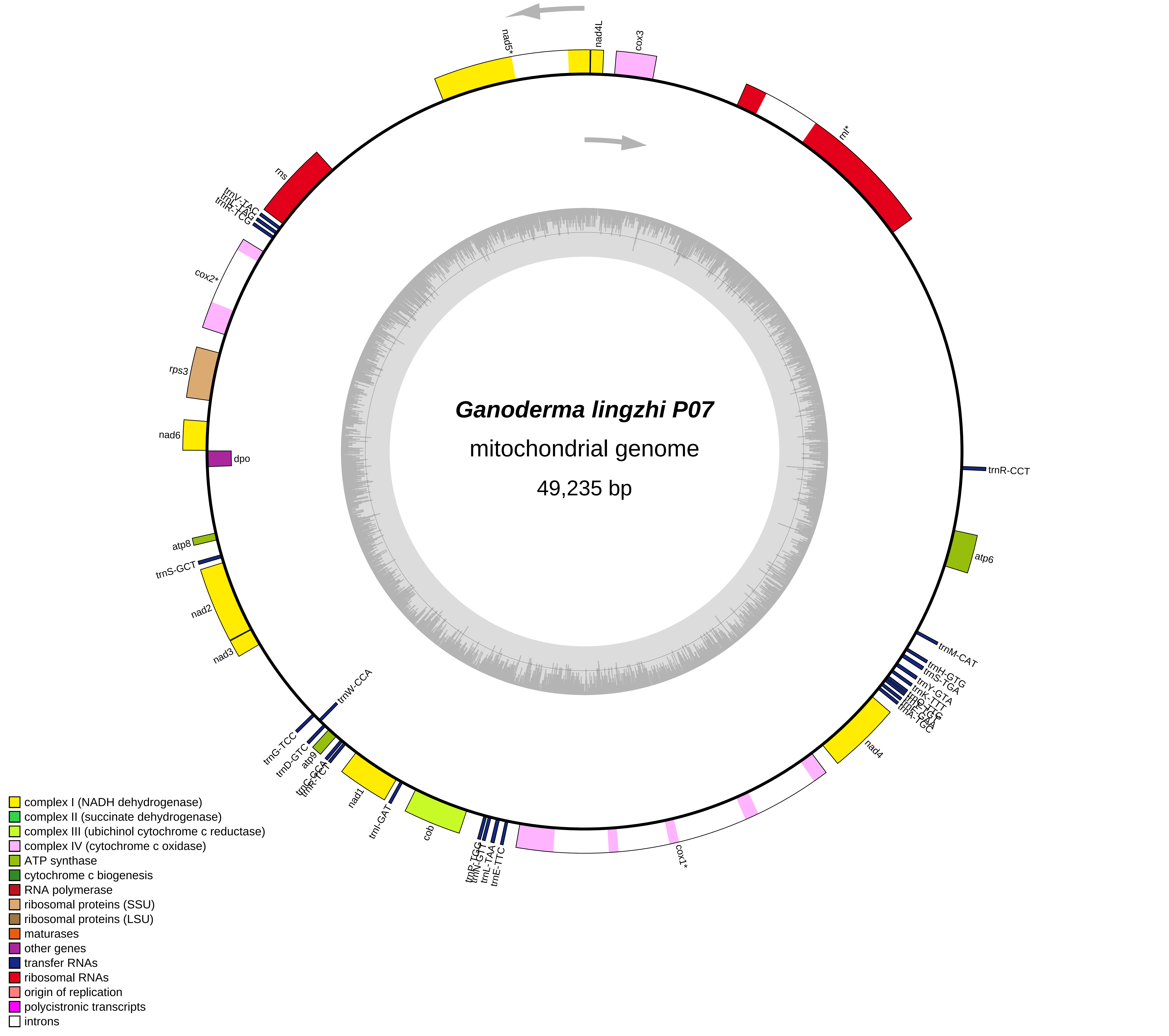

Ganoderma lingzhi P07
mitochondrial genome
49,235 bp
nad5*
nad4L
cox3
rnl*
rns
trnV-TAC
trnL-TAG
trnR-TCG
cox2*
rps3
nad6
dpo
trnR-CCT
atp6
atp8
trnS-GCT
nad2
nad3
trnM-CAT
trnH-GTG
trnS-TGA
trnY-GTA
trnK-TTT
trnA-TGC
nad4
trnW-CCA
trnG-TCC
trnD-GTC
atp9
trnC-GCA
trnR-TCT
nad1
trnI-GAT
cob
trnP-TGG
trnN-GTT
trnL-TAA
trnE-TTC
cox1*
complex I (NADH dehydrogenase)
complex II (succinate dehydrogenase)
complex III (ubichinol cytochrome c reductase)
complex IV (cytochrome c oxidase)
ATP synthase
cytochrome c biogenesis
RNA polymerase
ribosomal proteins (SSU)
ribosomal proteins (LSU)
maturases
other genes
transfer RNAs
ribosomal RNAs
origin of replication
polycistronic transcripts
introns

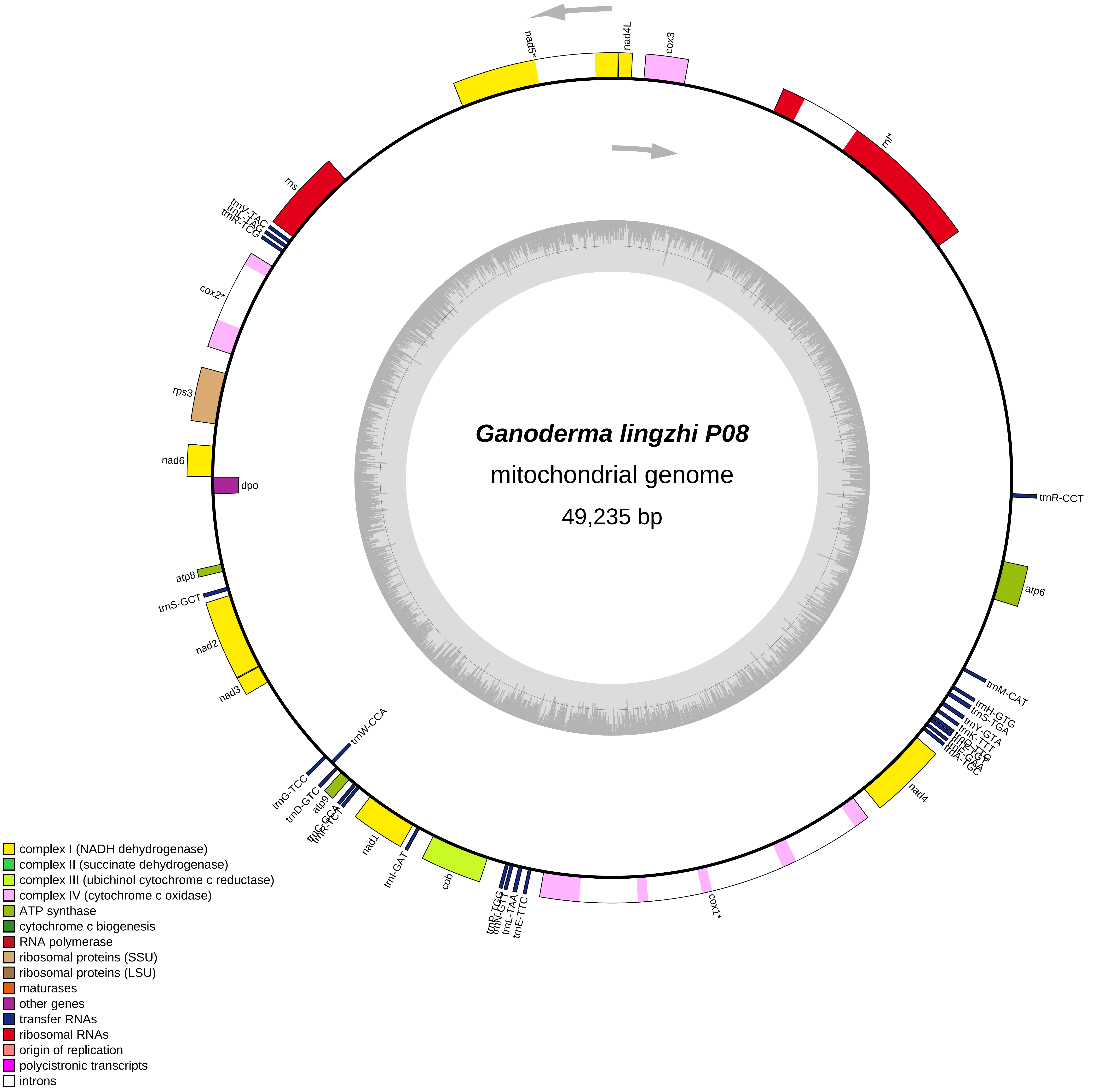
Ganoderma lingzhi P08
mitochondrial genome
49,235 bp
nad5*
nad4L
cox3
rnl*
rns
trnV-TAC
trnR-TCG
cox2*
rps3
nad6
dpo
trnR-CCT
atp6
atp8
trnS-GCT
nad2
nad3
trnM-CAT
trnH-GTG
trnS-TGA
trnY-GTA
trnK-TTT
trnA-TGC
nad4
trnW-CCA
trnG-TCC
trnD-GTC
atp9
nad1
trnI-GAT
cob
trnP-TGG
trnL-TAA
trnE-TTC
cox1*
complex I (NADH dehydrogenase)
complex II (succinate dehydrogenase)
complex III (ubichinol cytochrome c reductase)
complex IV (cytochrome c oxidase)
ATP synthase
cytochrome c biogenesis
RNA polymerase
ribosomal proteins (SSU)
ribosomal proteins (LSU)
maturases
other genes
transfer RNAs
ribosomal RNAs
origin of replication
polycistronic transcripts
introns

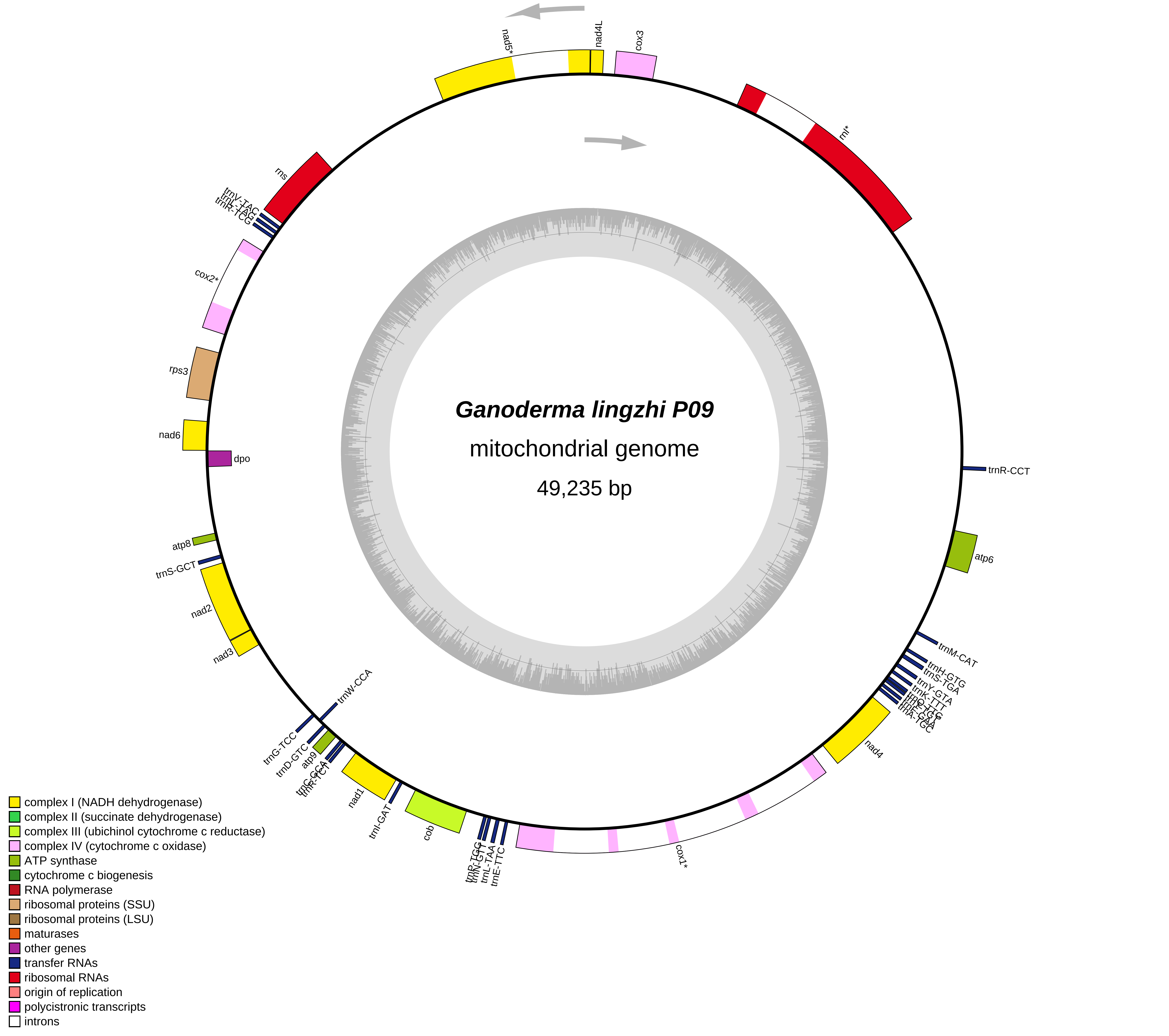

Ganoderma lingzhi P09
mitochondrial genome
49,235 bp
nad5*
nad4L
cox3
rnl*
rns
trnV-TAC
trnL-TAG
trnR-TCG
cox2*
rps3
nad6
dpo
trnR-CCT
atp6
atp8
trnS-GCT
nad2
nad3
trnW-CCA
trnG-TCC
trnD-GTC
atp9
trnC-GCA
trnR-TCT
nad1
trnI-GAT
cob
trnP-TGG
trnN-GTT
trnL-TAA
trnE-TTC
cox1*
nad4
trnM-CAT
trnH-GTG
trnS-TGA
trnY-GTA
trnK-TTT
trnA-TGC
complex I (NADH dehydrogenase)
complex II (succinate dehydrogenase)
complex III (ubichinol cytochrome c reductase)
complex IV (cytochrome c oxidase)
ATP synthase
cytochrome c biogenesis
RNA polymerase
ribosomal proteins (SSU)
ribosomal proteins (LSU)
maturases
other genes
transfer RNAs
ribosomal RNAs
origin of replication
polycistronic transcripts
introns

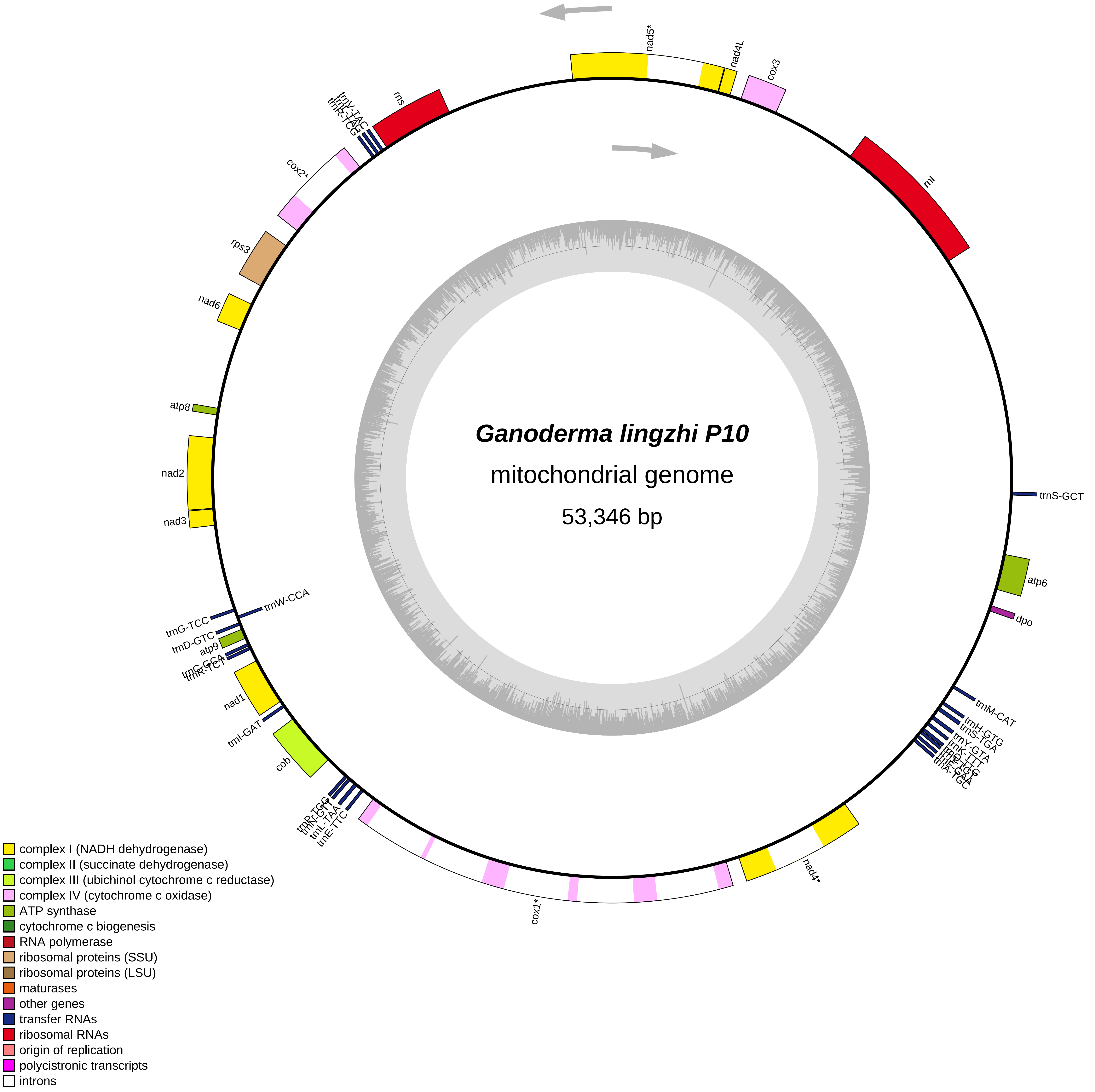
Ganoderma lingzhi P10
mitochondrial genome
53,346 bp
nad5*
nad4L
cox3
rnl
trnS-GCT
atp6
dpo
trnM-CAT
trnH-GTG
trnS-TGA
trnY-GTA
nad4*
cox1*
trnL-TAA
trnE-TTC
cob
trnI-GAT
nad1
trnR-TCT
atp9
trnD-GTC
trnG-TCC
trnW-CCA
nad3
nad2
atp8
nad6
rps3
cox2*
rns
trnV-TAC
complex I (NADH dehydrogenase)
complex II (succinate dehydrogenase)
complex III (ubichinol cytochrome c reductase)
complex IV (cytochrome c oxidase)
ATP synthase
cytochrome c biogenesis
RNA polymerase
ribosomal proteins (SSU)
ribosomal proteins (LSU)
maturases
other genes
transfer RNAs
ribosomal RNAs
origin of replication
polycistronic transcripts
introns

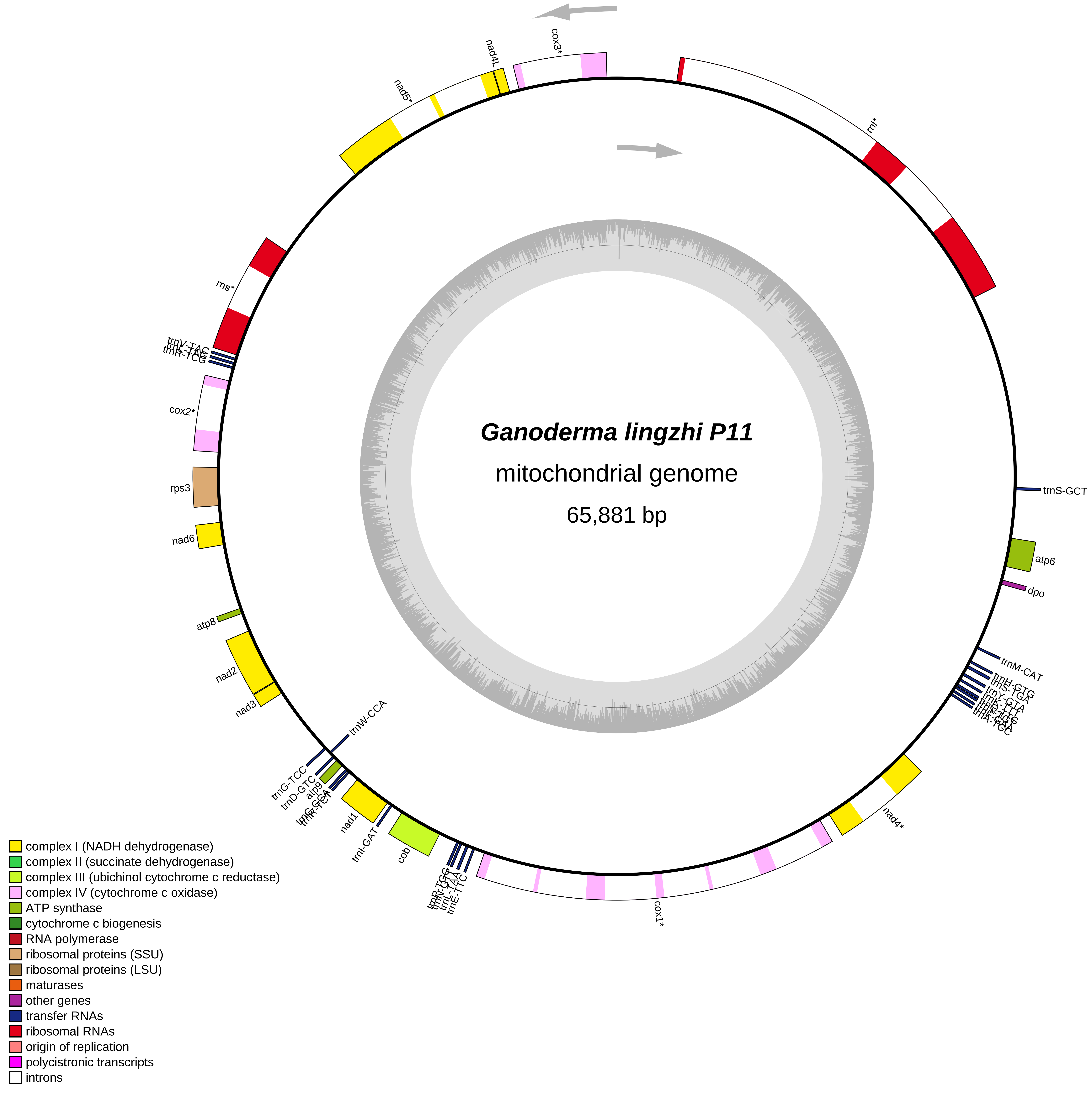
Ganoderma lingzhi P11
mitochondrial genome
65,881 bp
rnl*
trnS-GCT
atp6
dpo
trnM-CAT
nad4*
cox1*
cob
trnI-GAT
nad1
trnW-CCA
trnG-TCC
trnD-GTC
atp9
nad3
nad2
atp8
nad6
rps3
cox2*
trnR-TCG
rns*
nad5*
nad4L
cox3*
complex I (NADH dehydrogenase)
complex II (succinate dehydrogenase)
complex III (ubichinol cytochrome c reductase)
complex IV (cytochrome c oxidase)
ATP synthase
cytochrome c biogenesis
RNA polymerase
ribosomal proteins (SSU)
ribosomal proteins (LSU)
maturases
other genes
transfer RNAs
ribosomal RNAs
origin of replication
polycistronic transcripts
introns

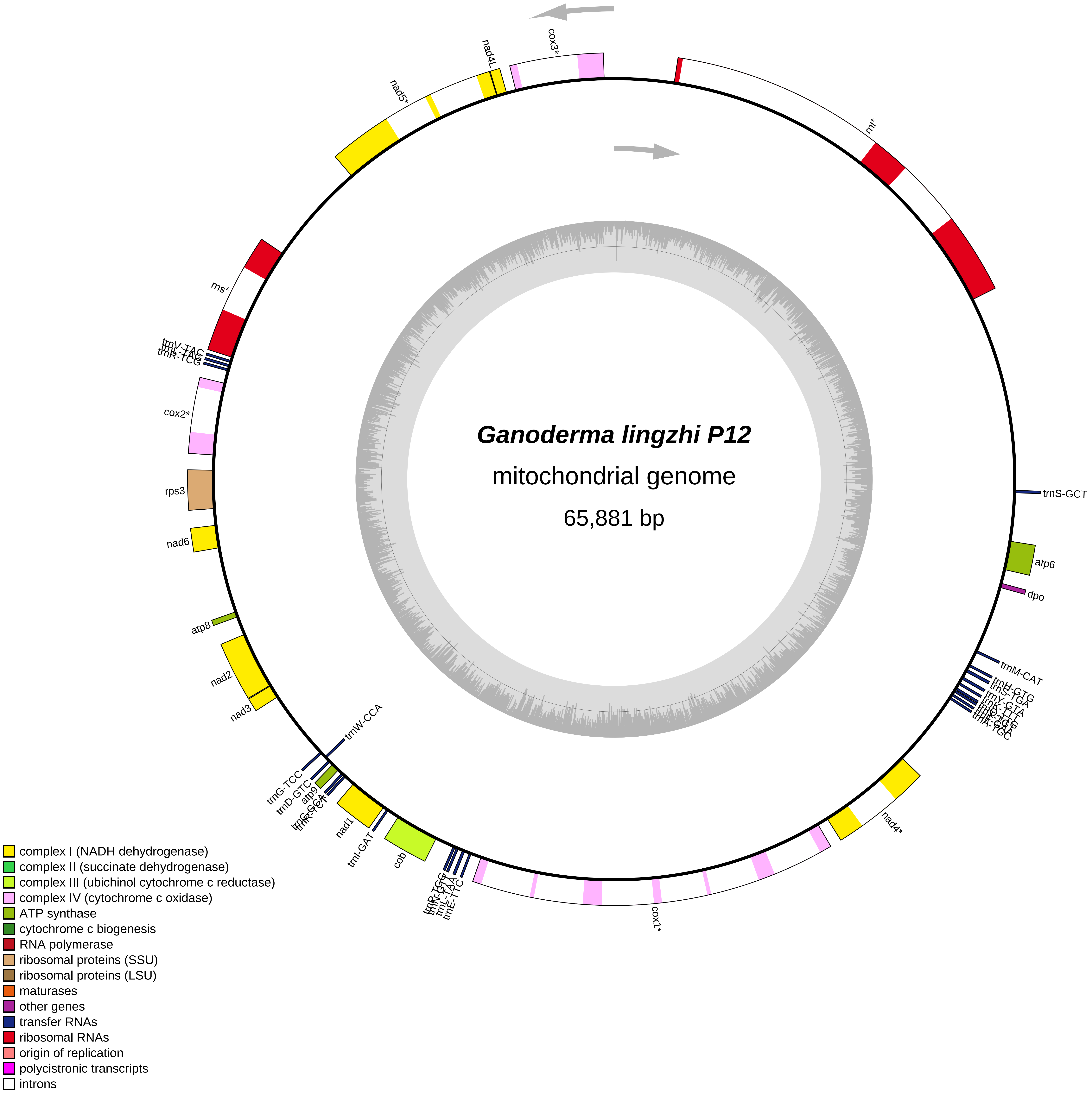

Ganoderma lingzhi P12
mitochondrial genome
65,881 bp
cox3*
nad4L
nad5*
rnl*
rns*
trnV-TAC
trnR-TCG
cox2*
rps3
nad6
atp8
nad2
nad3
trnW-CCA
trnG-TCC
trnD-GTC
atp9
nad1
trnI-GAT
cob
trnR-TCG
trnE-TTC
cox1*
nad4*
trnM-CAT
trnH-GTG
trnA-TGC
dpo
atp6
trnS-GCT
complex I (NADH dehydrogenase)
complex II (succinate dehydrogenase)
complex III (ubichinol cytochrome c reductase)
complex IV (cytochrome c oxidase)
ATP synthase
cytochrome c biogenesis
RNA polymerase
ribosomal proteins (SSU)
ribosomal proteins (LSU)
maturases
other genes
transfer RNAs
ribosomal RNAs
origin of replication
polycistronic transcripts
introns

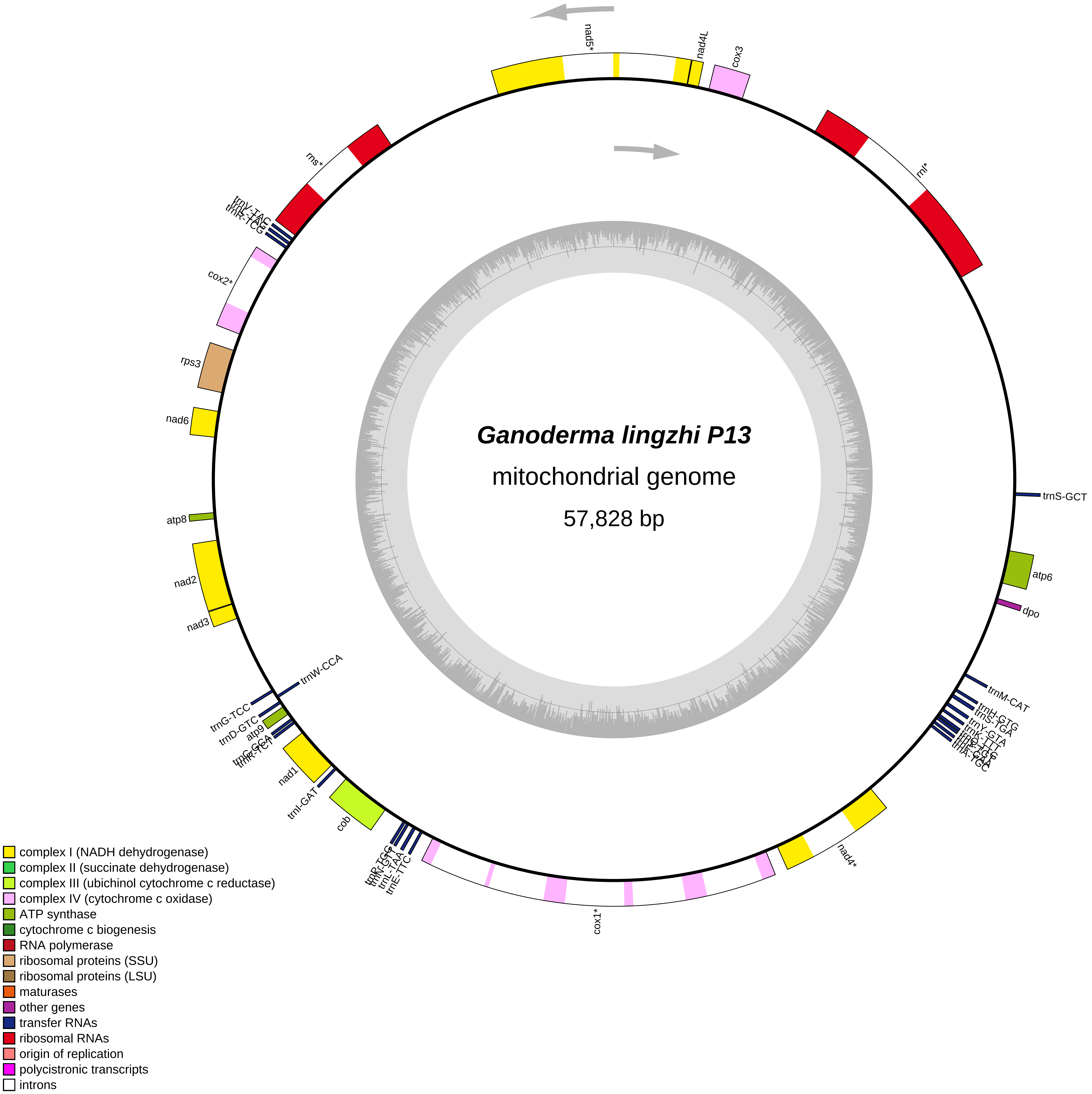

Ganoderma lingzhi P13
mitochondrial genome
57,828 bp
nad5*
nad4L
cox3
rnl*
trnS-GCT
atp6
dpo
trnM-CAT
trnH-GTG
trnS-TGA
trnY-GTA
nad4*
cox1*
trnE-TTC
trnL-TAA
cob
trnI-GAT
nad1
trnW-CCA
trnG-TCC
trnD-GTC
atp9
nad3
nad2
atp8
nad6
rps3
cox2*
rns*
trnV-TAC
complex I (NADH dehydrogenase)
complex II (succinate dehydrogenase)
complex III (ubichinol cytochrome c reductase)
complex IV (cytochrome c oxidase)
ATP synthase
cytochrome c biogenesis
RNA polymerase
ribosomal proteins (SSU)
ribosomal proteins (LSU)
maturases
other genes
transfer RNAs
ribosomal RNAs
origin of replication
polycistronic transcripts
introns

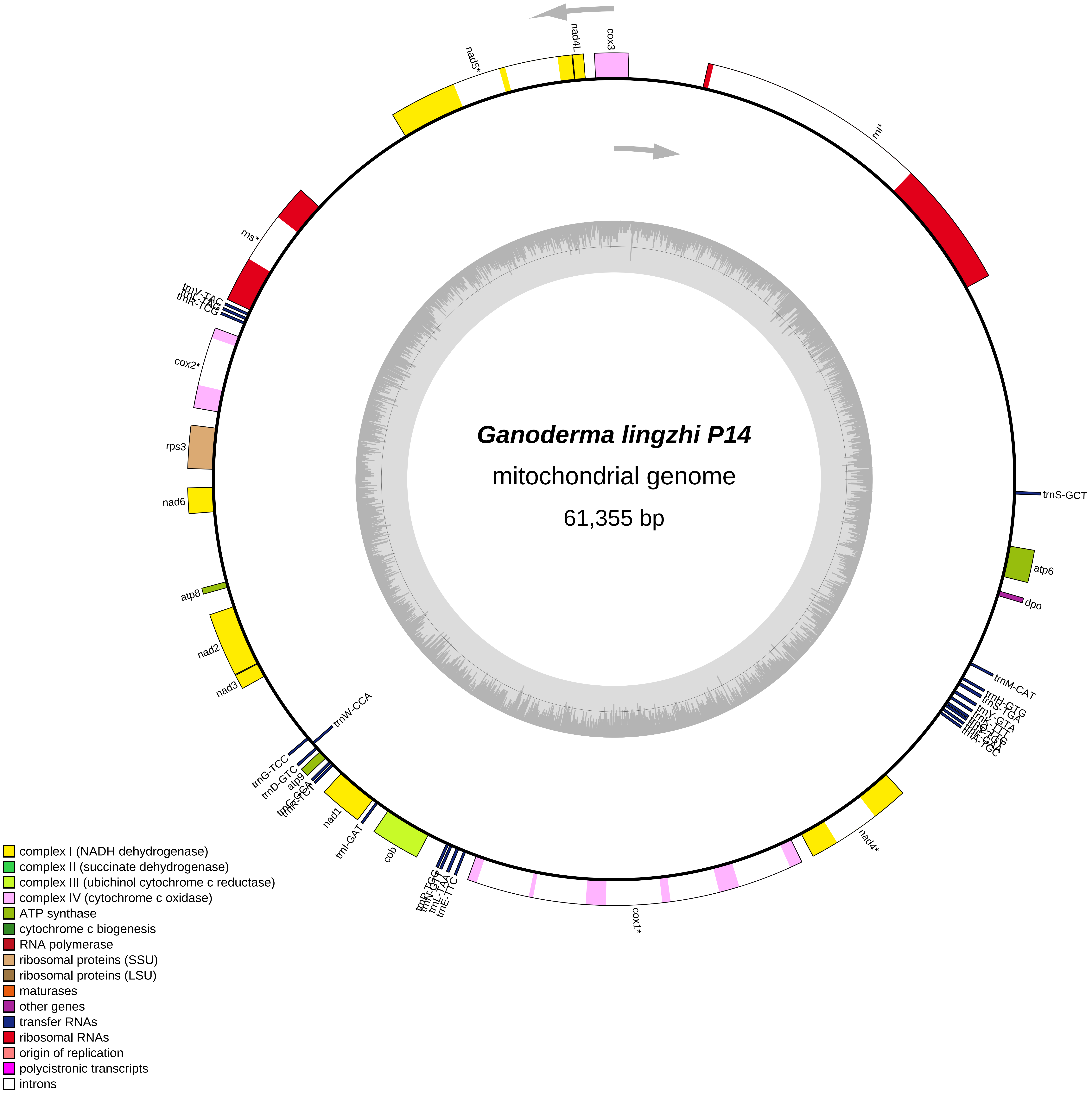
Ganoderma lingzhi P14
mitochondrial genome
61,355 bp
nad5*
nad4L
cox3
rnl*
rns*
trnV-TAC
trnR-TCG
cox2*
rps3
nad6
atp8
nad2
nad3
trnW-CCA
trnG-TCC
trnD-GTC
atp9
nad1
trnI-GAT
cob
trnE-TTC
cox1*
nad4*
trnM-CAT
trnH-GTG
trnS-TGA
trnS-GCT
atp6
dpo
complex I (NADH dehydrogenase)
complex II (succinate dehydrogenase)
complex III (ubichinol cytochrome c reductase)
complex IV (cytochrome c oxidase)
ATP synthase
cytochrome c biogenesis
RNA polymerase
ribosomal proteins (SSU)
ribosomal proteins (LSU)
maturases
other genes
transfer RNAs
ribosomal RNAs
origin of replication
polycistronic transcripts
introns

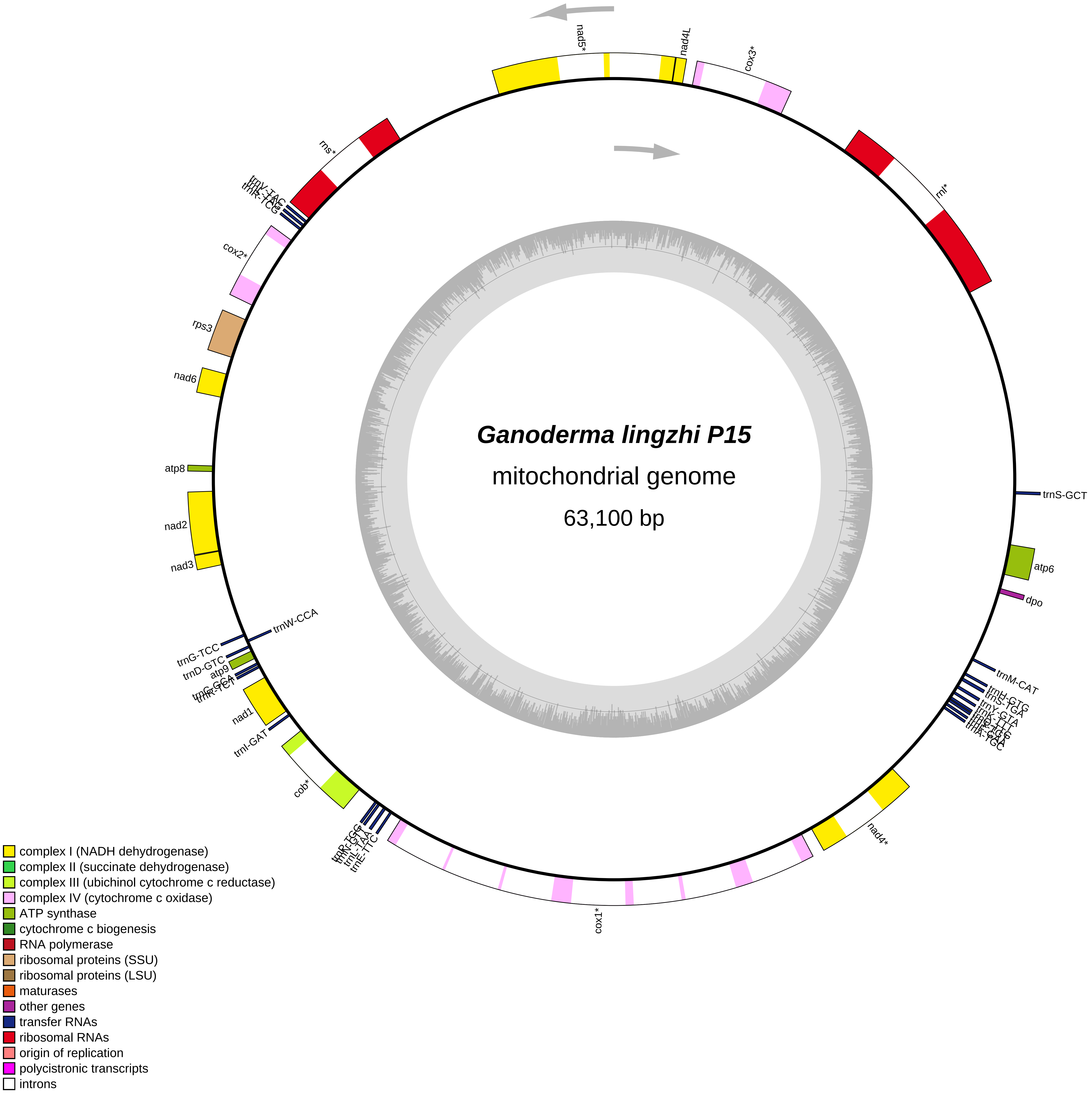

Ganoderma lingzhi P15
mitochondrial genome
63,100 bp
nad5*
nad4L
cox3*
rnl*
trnS-GCT
atp6
dpo
trnM-CAT
trnH-GTG
trnS-TGA
nad4*
cox1*
trnE-TTC
cob*
trnI-GAT
nad1
atp9
trnD-GTC
trnG-TCC
trnW-CCA
nad3
nad2
atp8
nad6
rps3
cox2*
rns*
complex I (NADH dehydrogenase)
complex II (succinate dehydrogenase)
complex III (ubichinol cytochrome c reductase)
complex IV (cytochrome c oxidase)
ATP synthase
cytochrome c biogenesis
RNA polymerase
ribosomal proteins (SSU)
ribosomal proteins (LSU)
maturases
other genes
transfer RNAs
ribosomal RNAs
origin of replication
polycistronic transcripts
introns

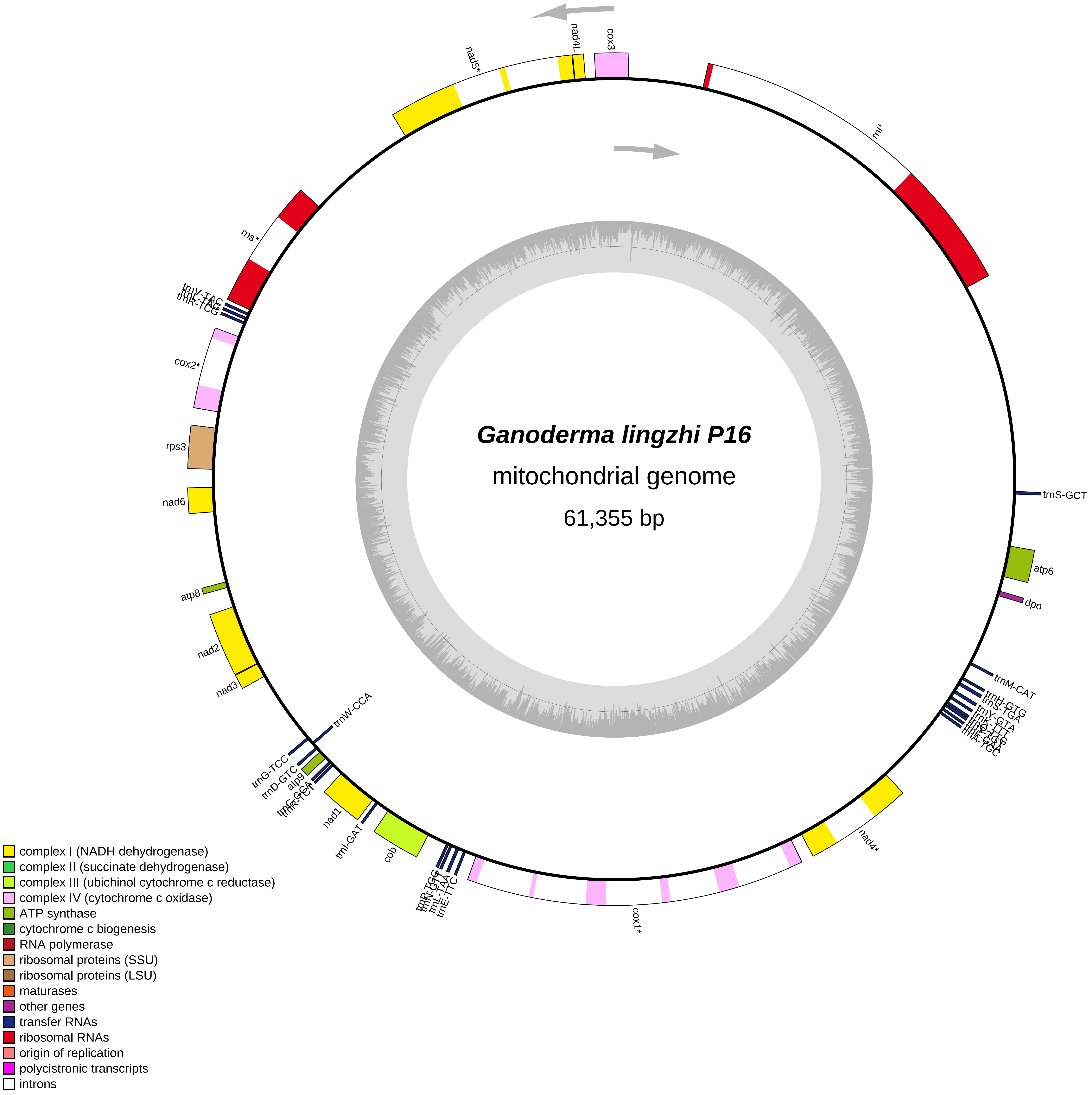

Ganoderma lingzhi P16
mitochondrial genome
61,355 bp
nad5*
nad4L
cox3
rnl*
rns*
trnV-TAC
trnR-TCG
cox2*
rps3
nad6
atp8
nad2
nad3
trnW-CCA
trnG-TCC
trnD-GTC
atp9
nad1
trnI-GAT
cob
trnL-TAA
trnE-TTC
cox1*
nad4*
trnM-CAT
trnH-GTG
trnS-TGA
trnS-GCT
atp6
dpo
complex I (NADH dehydrogenase)
complex II (succinate dehydrogenase)
complex III (ubichinol cytochrome c reductase)
complex IV (cytochrome c oxidase)
ATP synthase
cytochrome c biogenesis
RNA polymerase
ribosomal proteins (SSU)
ribosomal proteins (LSU)
maturases
other genes
transfer RNAs
ribosomal RNAs
origin of replication
polycistronic transcripts
introns

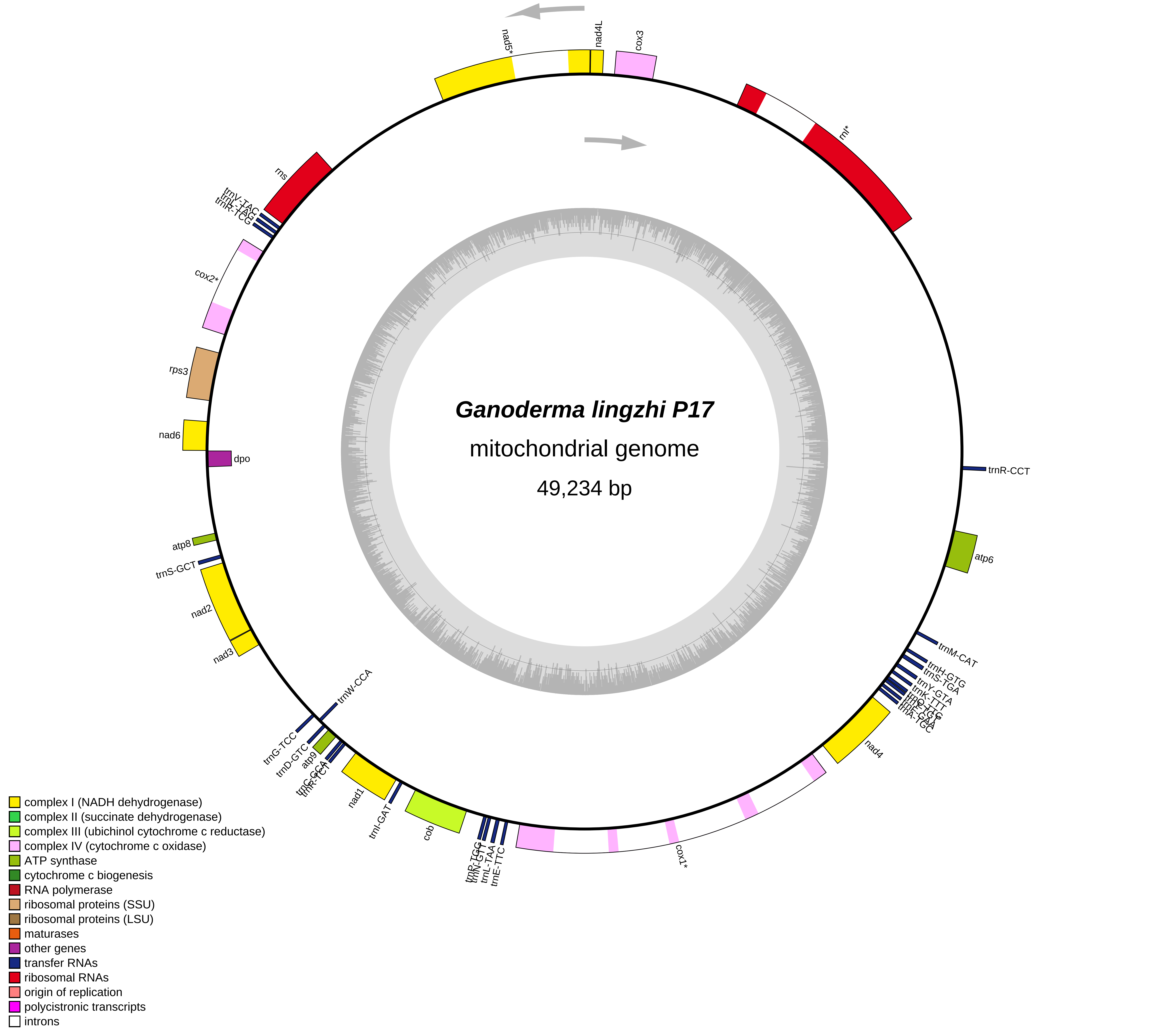

Ganoderma lingzhi P17
mitochondrial genome
49,234 bp
nad5*
nad4L
cox3
rnl*
rns
trnV-TAC
cox2*
rps3
nad6
dpo
trnR-CCT
atp6
atp8
trnS-GCT
nad2
nad3
trnM-CAT
trnH-GTG
trnS-TGA
trnY-GTA
trnK-TTT
nad4
trnW-CCA
trnG-TCC
trnD-GTC
atp9
nad1
trnI-GAT
cob
trnP-TGG
trnL-TAA
trnE-TTC
cox1*
complex I (NADH dehydrogenase)
complex II (succinate dehydrogenase)
complex III (ubichinol cytochrome c reductase)
complex IV (cytochrome c oxidase)
ATP synthase
cytochrome c biogenesis
RNA polymerase
ribosomal proteins (SSU)
ribosomal proteins (LSU)
maturases
other genes
transfer RNAs
ribosomal RNAs
origin of replication
polycistronic transcripts
introns

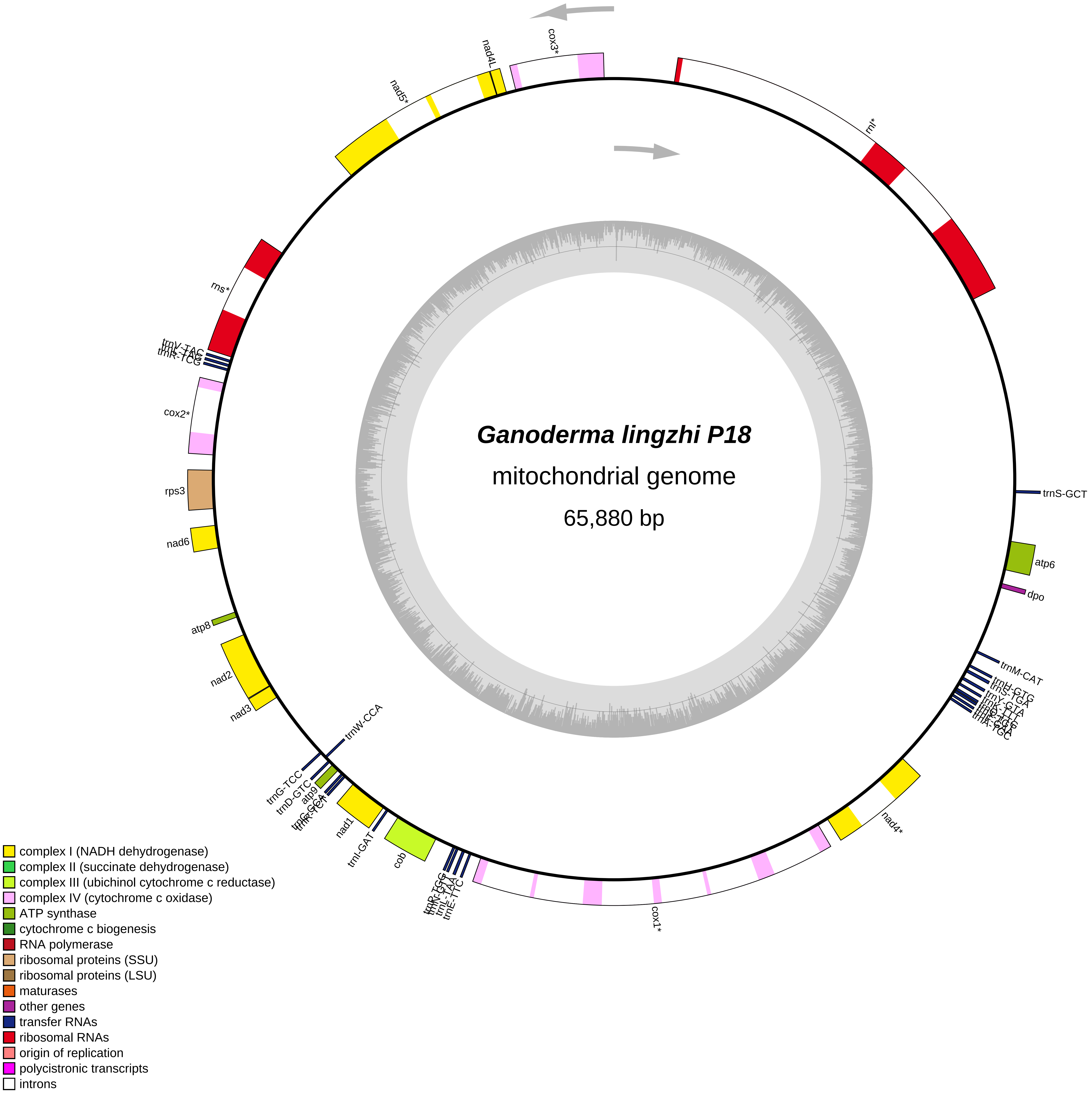
Ganoderma lingzhi P18
mitochondrial genome
65,880 bp
cox3*
nad4L
nad5*
rnl*
rns*
trnV-TAC
trnR-TCG
cox2*
rps3
nad6
atp8
nad2
nad3
trnW-CCA
trnG-TCC
trnD-GTC
atp9
nad1
trnI-GAT
cob
trnE-TTC
cox1*
nad4*
trnM-CAT
dpo
atp6
trnS-GCT
complex I (NADH dehydrogenase)
complex II (succinate dehydrogenase)
complex III (ubichinol cytochrome c reductase)
complex IV (cytochrome c oxidase)
ATP synthase
cytochrome c biogenesis
RNA polymerase
ribosomal proteins (SSU)
ribosomal proteins (LSU)
maturases
other genes
transfer RNAs
ribosomal RNAs
origin of replication
polycistronic transcripts
introns

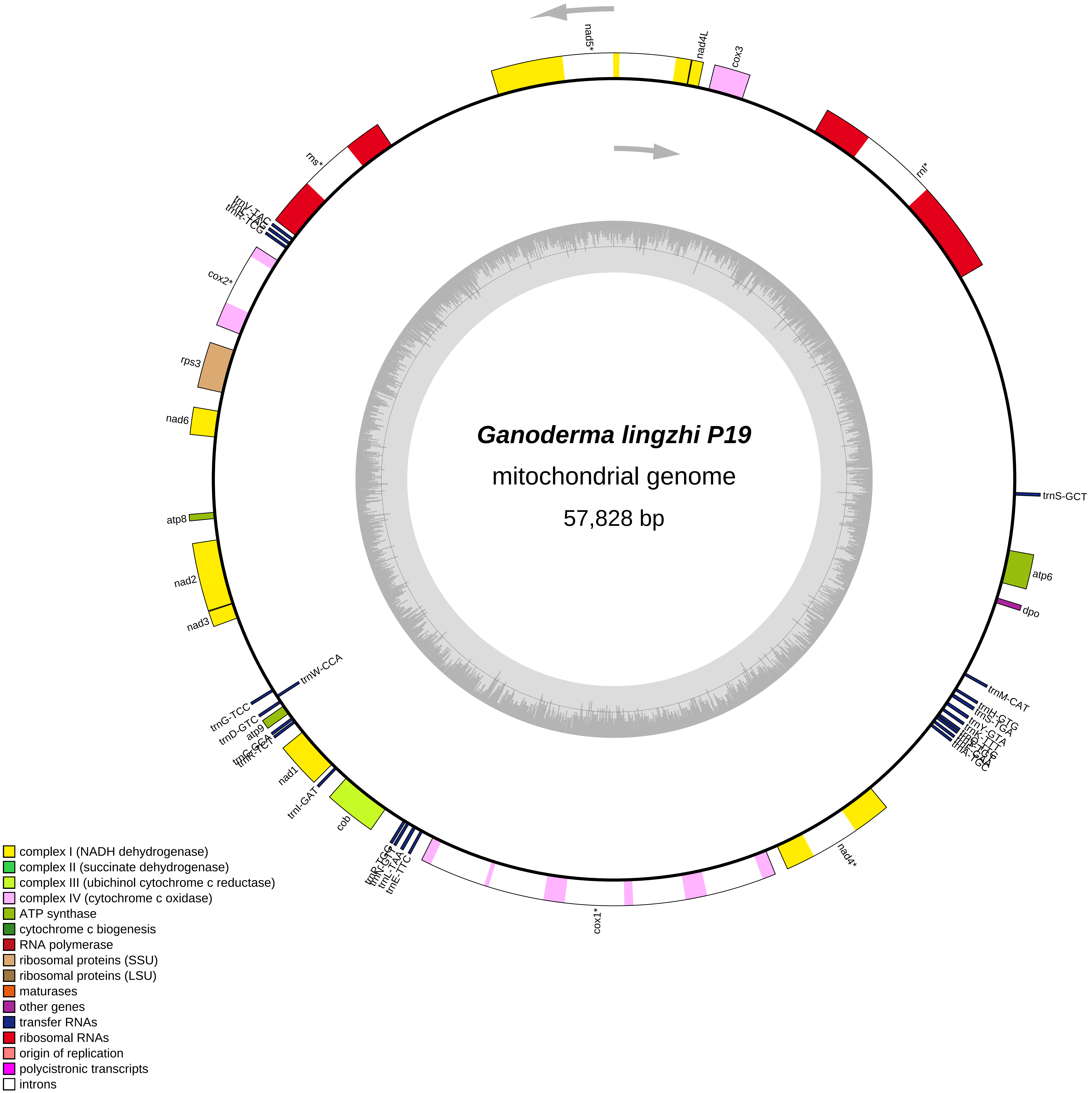
Ganoderma lingzhi P19
mitochondrial genome
57,828 bp
nad5*
nad4L
cox3
rnl*
trnS-GCT
atp6
dpo
trnM-CAT
trnH-GTG
trnS-TGA
trnY-GTA
nad4*
cox1*
trnE-TTC
trnL-TAA
cob
trnI-GAT
nad1
atp9
trnD-GTC
trnG-TCC
trnW-CCA
nad3
nad2
atp8
nad6
rps3
cox2*
trnV-TAC
rns*
complex I (NADH dehydrogenase)
complex II (succinate dehydrogenase)
complex III (ubichinol cytochrome c reductase)
complex IV (cytochrome c oxidase)
ATP synthase
cytochrome c biogenesis
RNA polymerase
ribosomal proteins (SSU)
ribosomal proteins (LSU)
maturases
other genes
transfer RNAs
ribosomal RNAs
origin of replication
polycistronic transcripts
introns

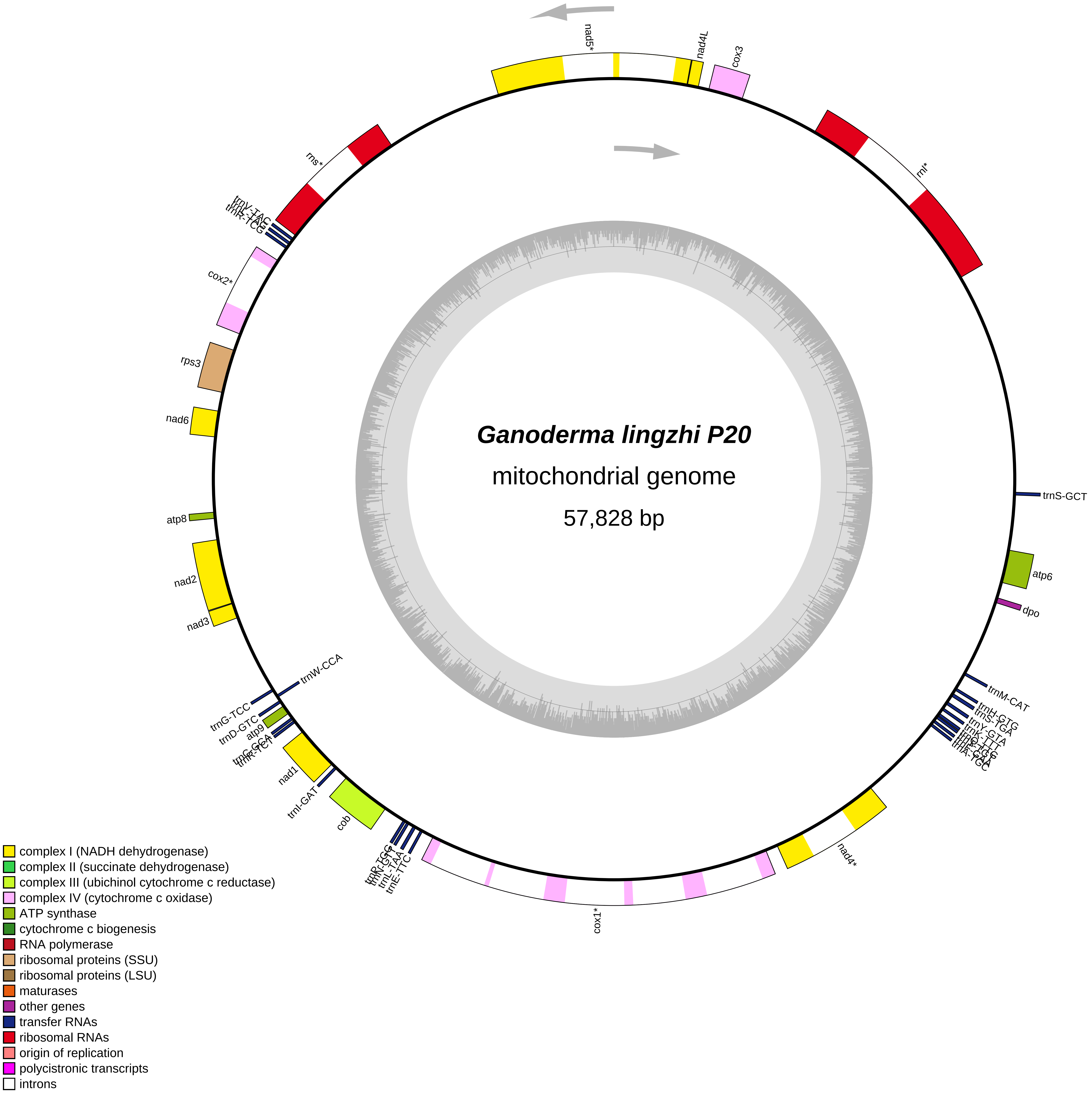
Ganoderma lingzhi P20
mitochondrial genome
57,828 bp
nad5*
nad4L
cox3
rnl*
trnS-GCT
atp6
dpo
trnM-CAT
trnH-GTG
trnS-TGA
trnY-GTA
nad4*
cox1*
trnE-TTC
trnL-TAA
cob
trnI-GAT
nad1
trnR-TCT
trnC-GCA
atp9
trnD-GTC
trnG-TCC
trnW-CCA
nad3
nad2
atp8
nad6
rps3
cox2*
trnR-TCG
trnV-TAC
rns*
complex I (NADH dehydrogenase)
complex II (succinate dehydrogenase)
complex III (ubichinol cytochrome c reductase)
complex IV (cytochrome c oxidase)
ATP synthase
cytochrome c biogenesis
RNA polymerase
ribosomal proteins (SSU)
ribosomal proteins (LSU)
maturases
other genes
transfer RNAs
ribosomal RNAs
origin of replication
polycistronic transcripts
introns

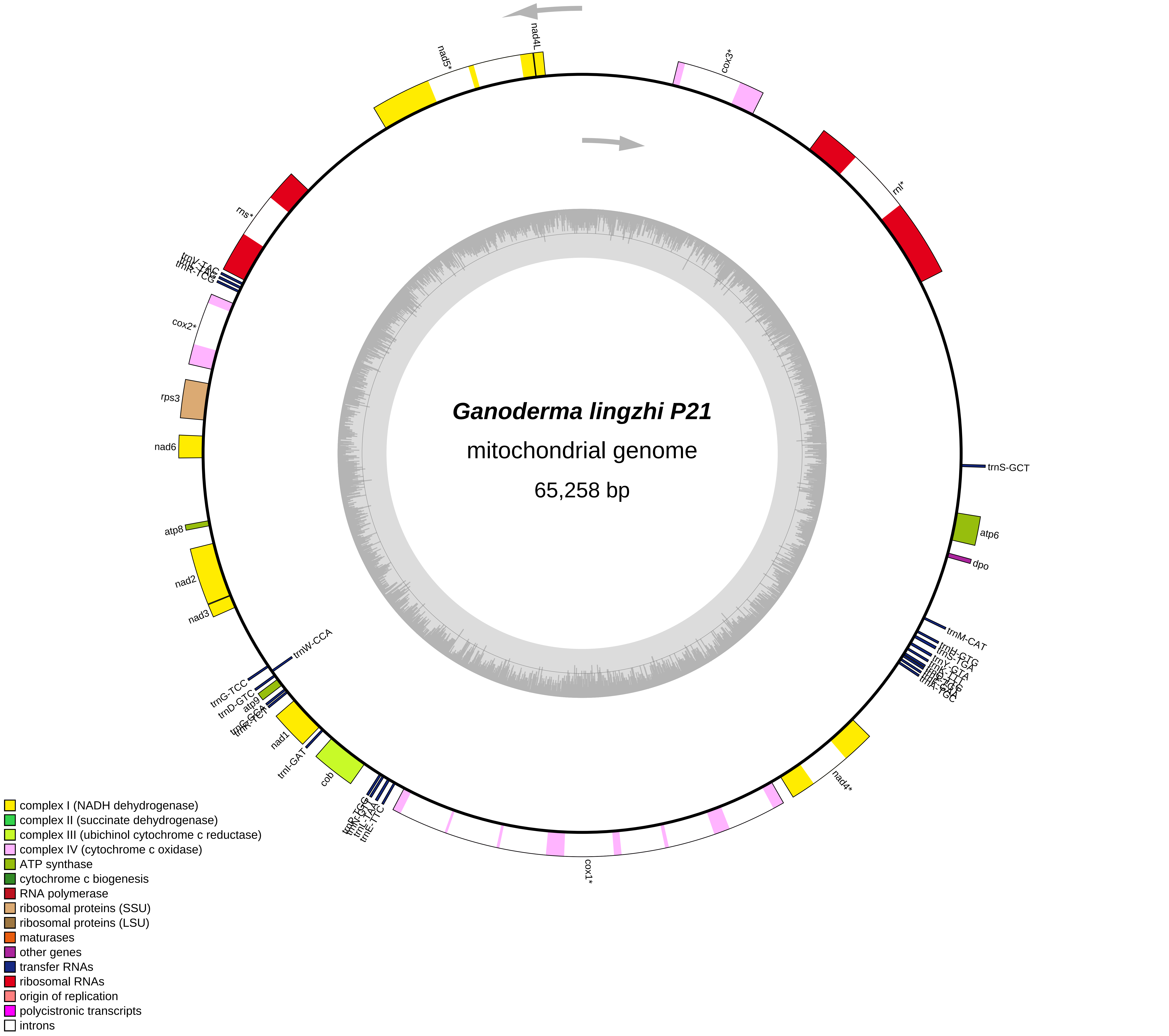

Ganoderma lingzhi P21
mitochondrial genome
65,258 bp
nad4L
nad5*
cox3*
rnl*
rns*
trnV-TAC
trnR-TCG
cox2*
rps3
nad6
trnS-GCT
atp6
dpo
atp8
nad2
nad3
trnM-CAT
trnH-GTG
trnW-CCA
trnG-TCC
trnD-GTC
atp9
nad1
trnI-GAT
cob
nad4*
trnE-TTC
cox1*
complex I (NADH dehydrogenase)
complex II (succinate dehydrogenase)
complex III (ubichinol cytochrome c reductase)
complex IV (cytochrome c oxidase)
ATP synthase
cytochrome c biogenesis
RNA polymerase
ribosomal proteins (SSU)
ribosomal proteins (LSU)
maturases
other genes
transfer RNAs
ribosomal RNAs
origin of replication
polycistronic transcripts
introns

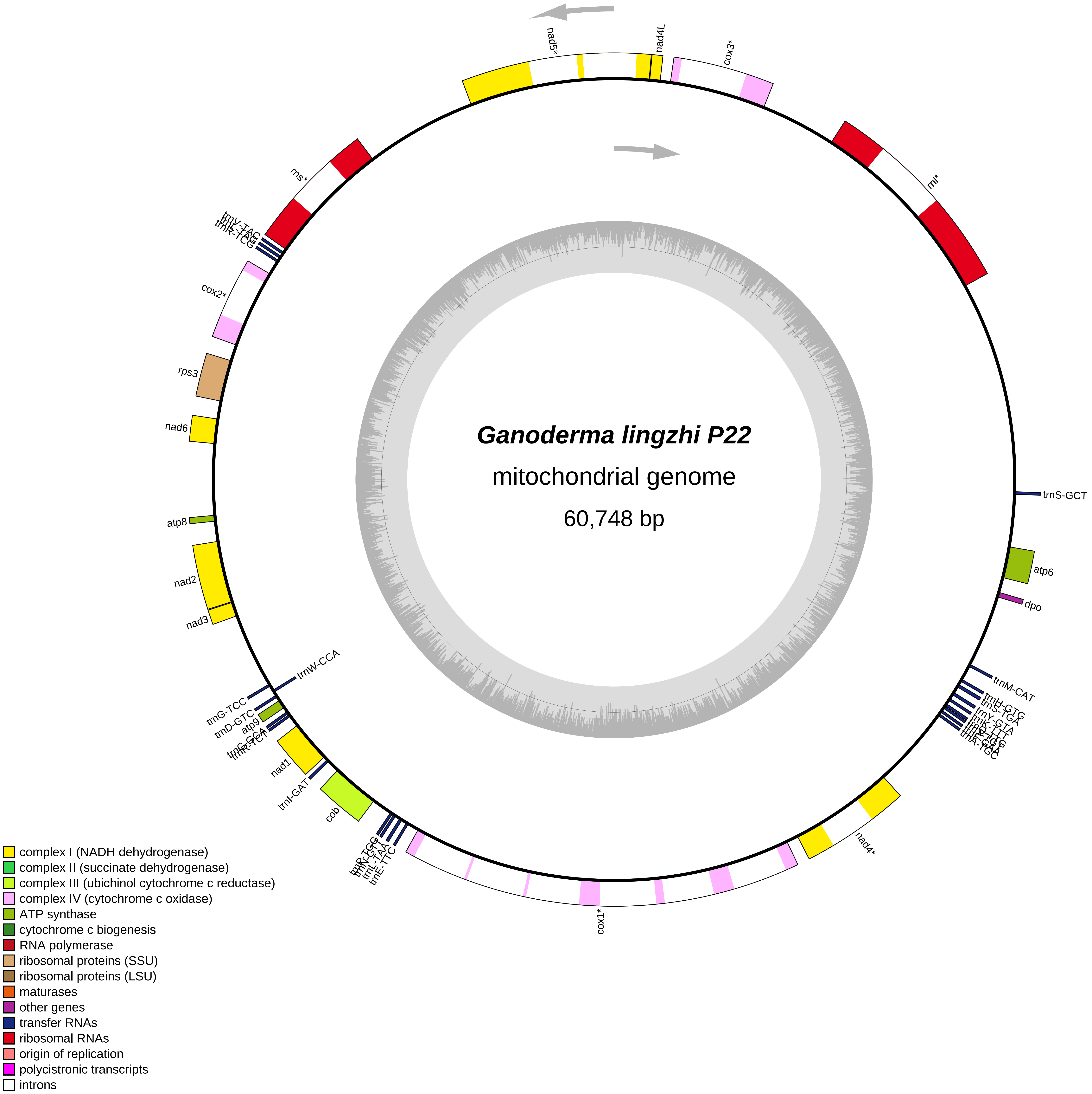
Ganoderma lingzhi P22
mitochondrial genome
60,748 bp
nad5*
nad4L
cox3*
rnl*
rns*
trnV-TAC
cox2*
rps3
nad6
atp8
nad2
nad3
trnW-CCA
trnG-TCC
trnD-GTC
atp9
nad1
trnI-GAT
cob
trnE-TTC
cox1*
nad4*
trnM-CAT
trnH-GTG
trnS-TGA
trnS-GCT
atp6
dpo
complex I (NADH dehydrogenase)
complex II (succinate dehydrogenase)
complex III (ubichinol cytochrome c reductase)
complex IV (cytochrome c oxidase)
ATP synthase
cytochrome c biogenesis
RNA polymerase
ribosomal proteins (SSU)
ribosomal proteins (LSU)
maturases
other genes
transfer RNAs
ribosomal RNAs
origin of replication
polycistronic transcripts
introns

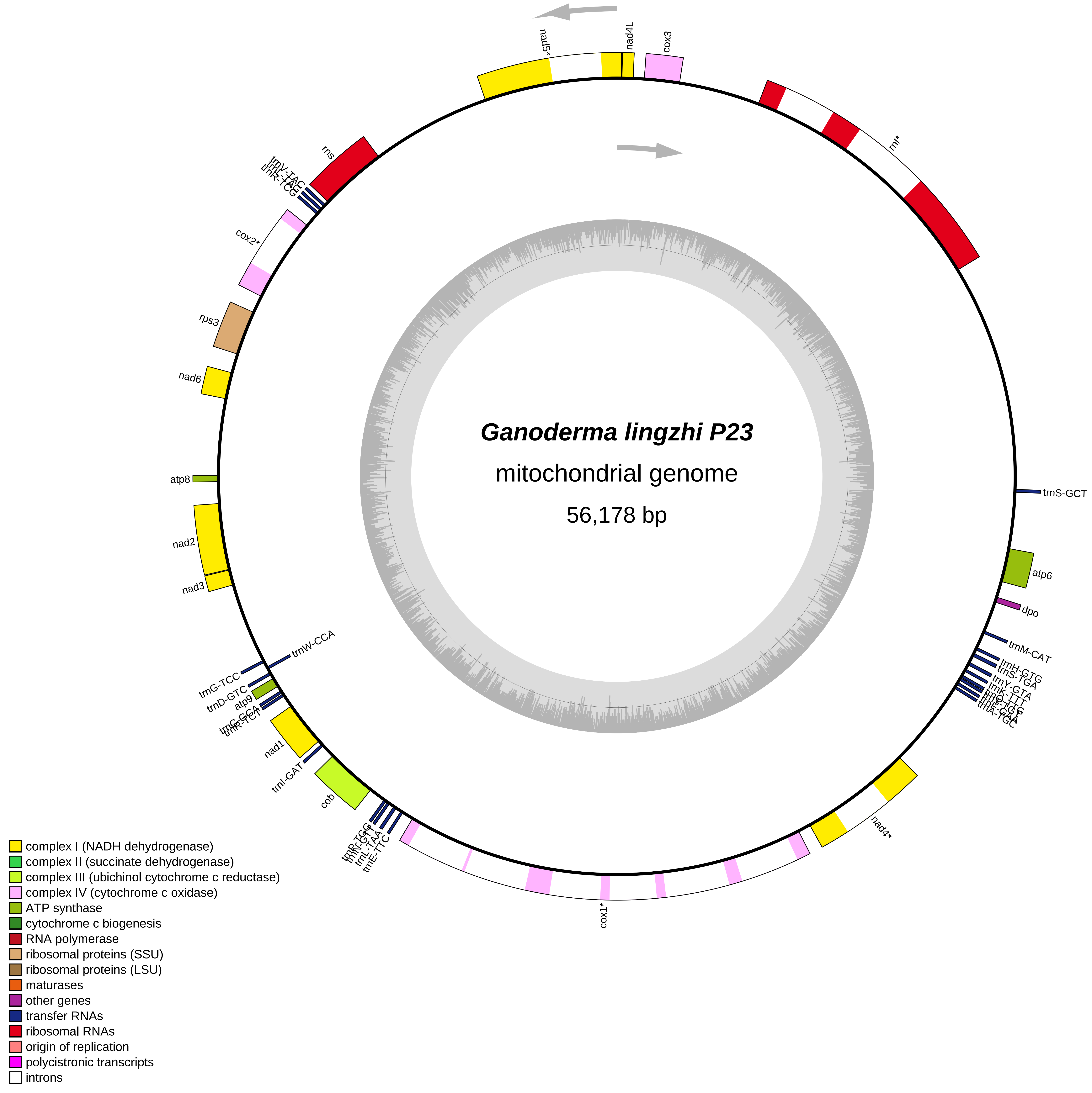

Ganoderma lingzhi P23
mitochondrial genome
56,178 bp
nad5*
nad4L
cox3
rnl*
trnS-GCT
atp6
dpo
trnM-CAT
trnH-GTG
trnS-TGA
trnY-GTA
trnA-TGC
nad4*
cox1*
trnE-TTC
trnL-TAA
cob
trnI-GAT
nad1
atp9
trnD-GTC
trnG-TCC
trnW-CCA
nad3
nad2
atp8
nad6
rps3
cox2*
trnV-TAC
rns
complex I (NADH dehydrogenase)
complex II (succinate dehydrogenase)
complex III (ubichinol cytochrome c reductase)
complex IV (cytochrome c oxidase)
ATP synthase
cytochrome c biogenesis
RNA polymerase
ribosomal proteins (SSU)
ribosomal proteins (LSU)
maturases
other genes
transfer RNAs
ribosomal RNAs
origin of replication
polycistronic transcripts
introns

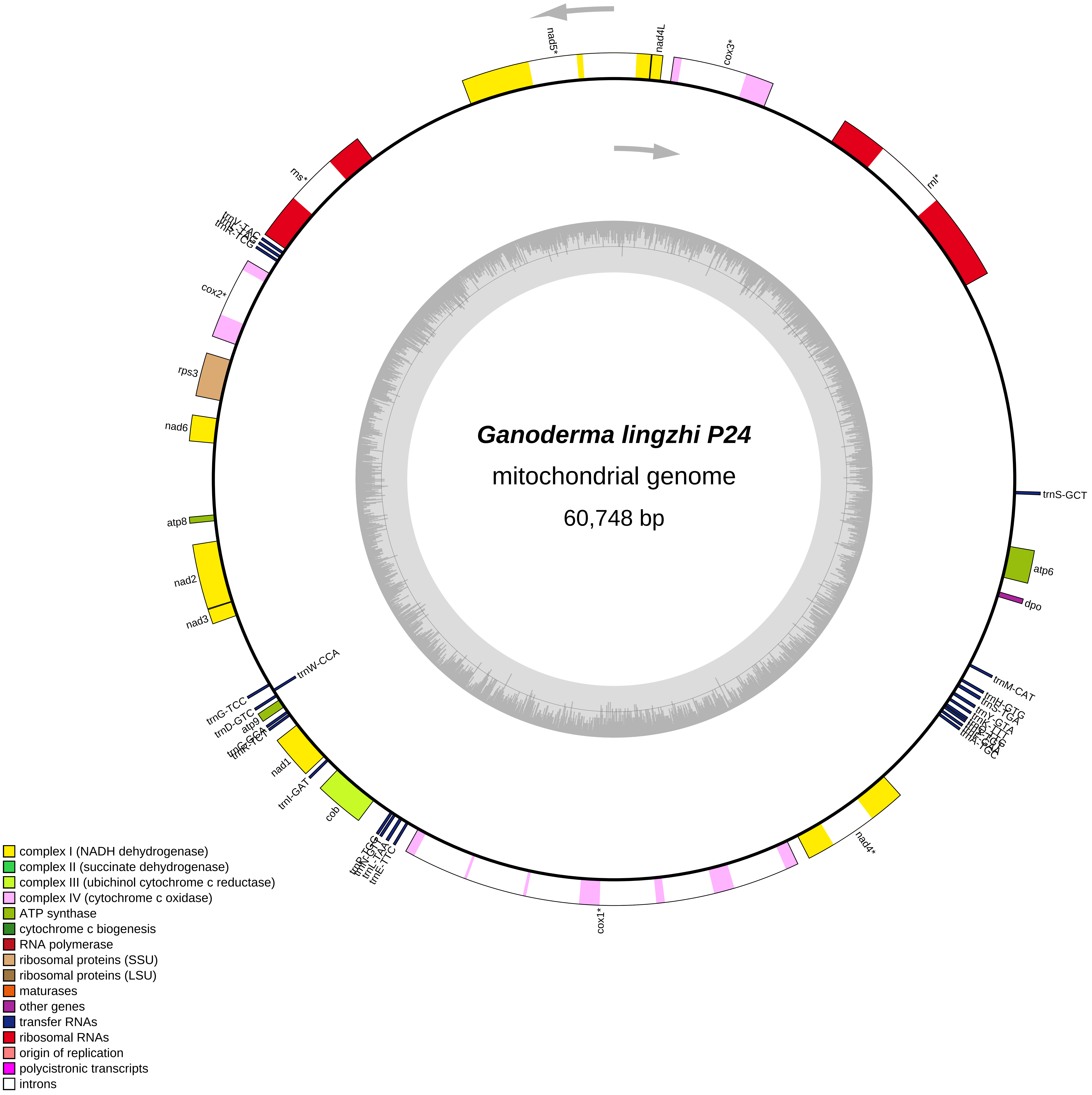

Ganoderma lingzhi P24
mitochondrial genome
60,748 bp
nad5*
nad4L
cox3*
rnl*
trnS-GCT
atp6
dpo
trnM-CAT
trnH-GTG
trnS-TGA
trnY-GTA
nad4*
cox1*
trnE-TTC
trnL-TAA
cob
trnI-GAT
nad1
atp9
trnD-GTC
trnG-TCC
trnW-CCA
nad3
nad2
atp8
nad6
rps3
cox2*
rns*
trnV-TAC
trnR-TCG
complex I (NADH dehydrogenase)
complex II (succinate dehydrogenase)
complex III (ubichinol cytochrome c reductase)
complex IV (cytochrome c oxidase)
ATP synthase
cytochrome c biogenesis
RNA polymerase
ribosomal proteins (SSU)
ribosomal proteins (LSU)
maturases
other genes
transfer RNAs
ribosomal RNAs
origin of replication
polycistronic transcripts
introns

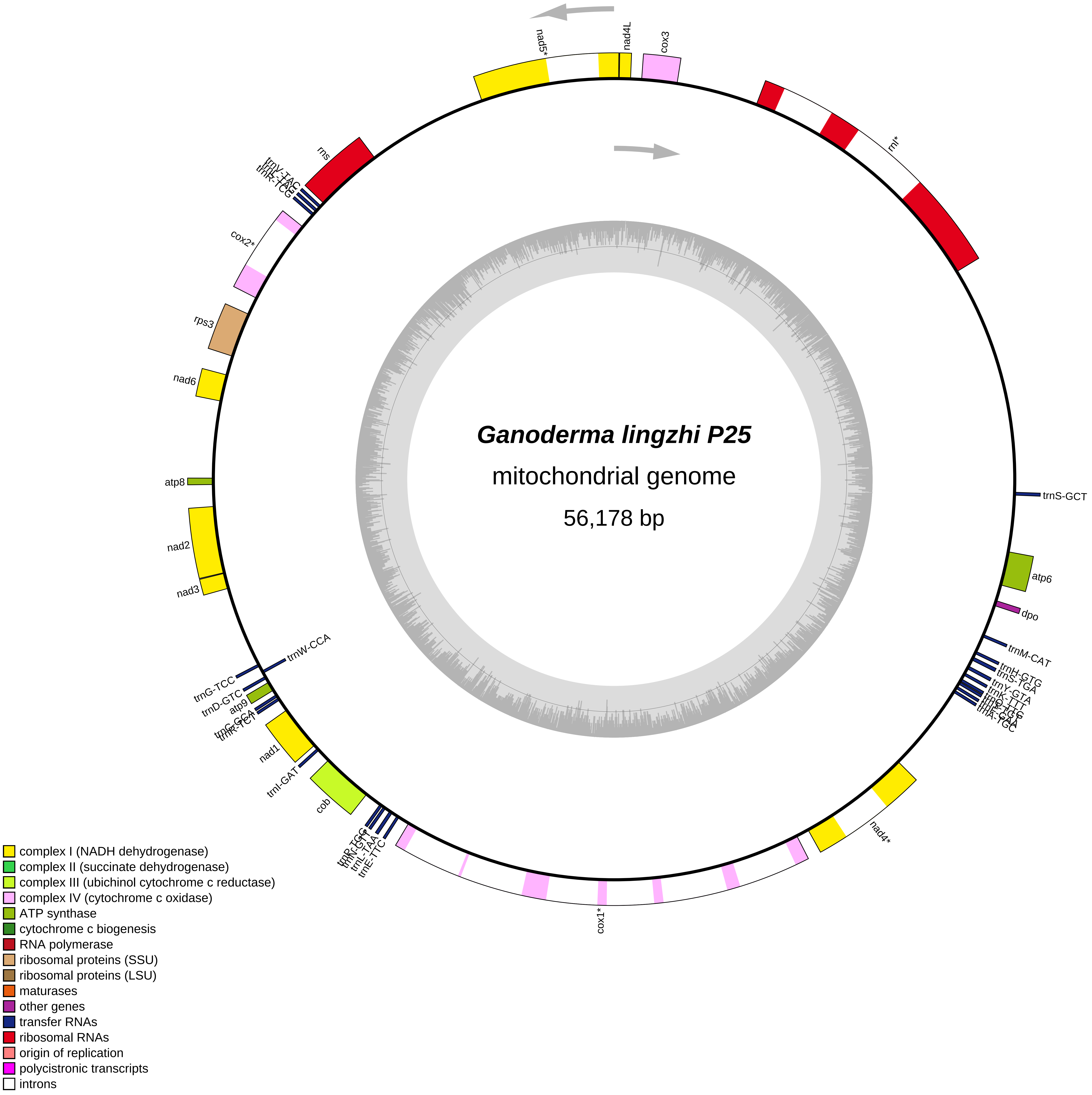
Ganoderma lingzhi P25
mitochondrial genome
56,178 bp
nad5*
nad4L
cox3
rnl*
rns
trnV-TAC
cox2*
rps3
nad6
atp8
nad2
nad3
trnW-CCA
trnG-TCC
trnD-GTC
atp9
trnC-GCA
trnR-TCT
nad1
trnI-GAT
cob
trnL-TAA
trnE-TTC
cox1*
nad4*
trnS-GCT
atp6
dpo
trnM-CAT
trnH-GTG
trnS-TGA
trnY-GTA
trnK-TTT
trnA-TGC
complex I (NADH dehydrogenase)
complex II (succinate dehydrogenase)
complex III (ubichinol cytochrome c reductase)
complex IV (cytochrome c oxidase)
ATP synthase
cytochrome c biogenesis
RNA polymerase
ribosomal proteins (SSU)
ribosomal proteins (LSU)
maturases
other genes
transfer RNAs
ribosomal RNAs
origin of replication
polycistronic transcripts
introns

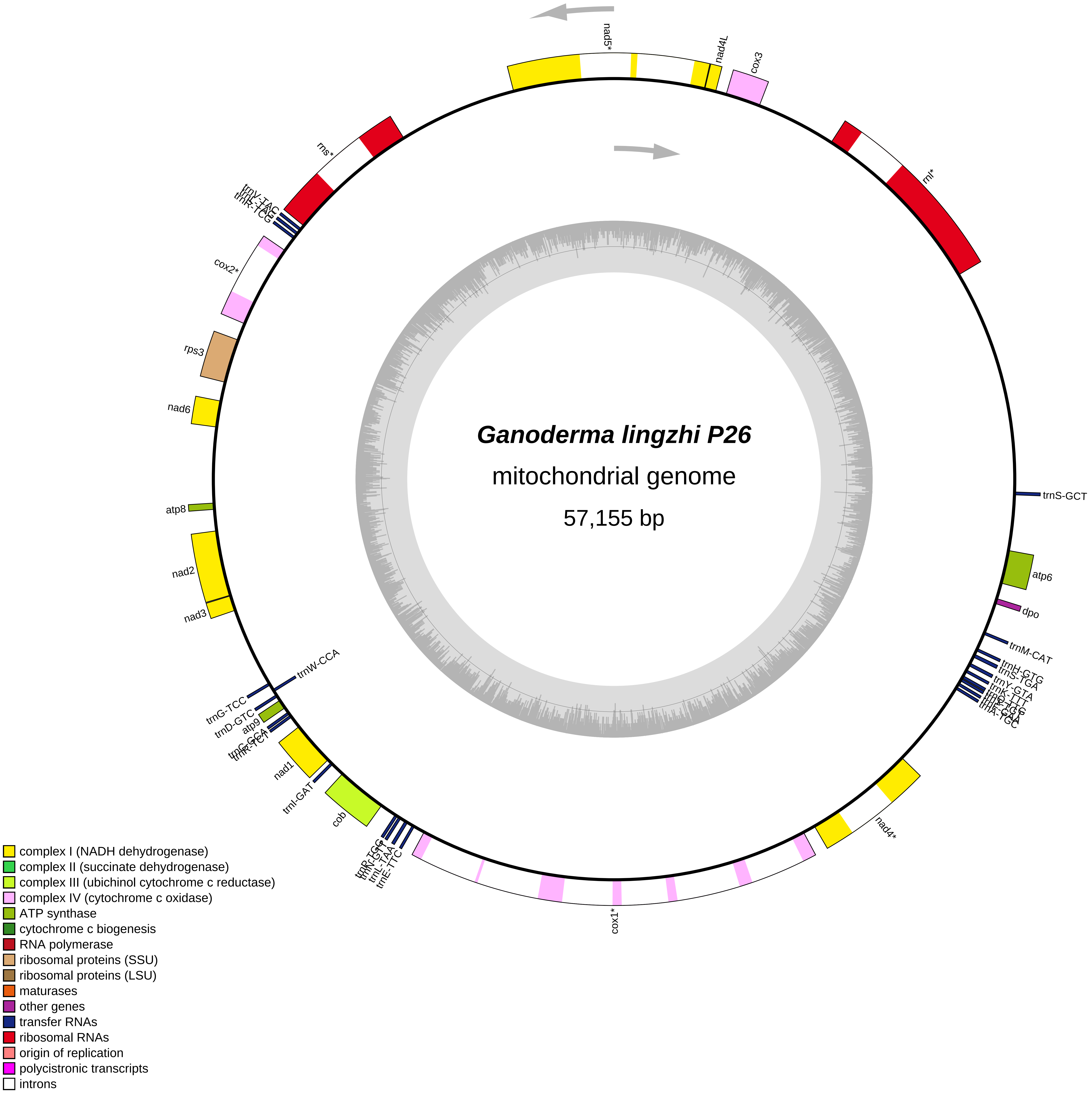
Ganoderma lingzhi P26
mitochondrial genome
57,155 bp
nad5*
nad4L
cox3
rnl*
rns*
trnV-TAC
cox2*
rps3
nad6
atp8
nad2
nad3
trnW-CCA
trnG-TCC
trnD-GTC
atp9
nad1
trnI-GAT
cob
trnE-TTC
cox1*
nad4*
trnS-GCT
atp6
dpo
trnM-CAT
trnH-GTG
trnS-TGA
trnY-GTA
trnA-TGC
complex I (NADH dehydrogenase)
complex II (succinate dehydrogenase)
complex III (ubichinol cytochrome c reductase)
complex IV (cytochrome c oxidase)
ATP synthase
cytochrome c biogenesis
RNA polymerase
ribosomal proteins (SSU)
ribosomal proteins (LSU)
maturases
other genes
transfer RNAs
ribosomal RNAs
origin of replication
polycistronic transcripts
introns

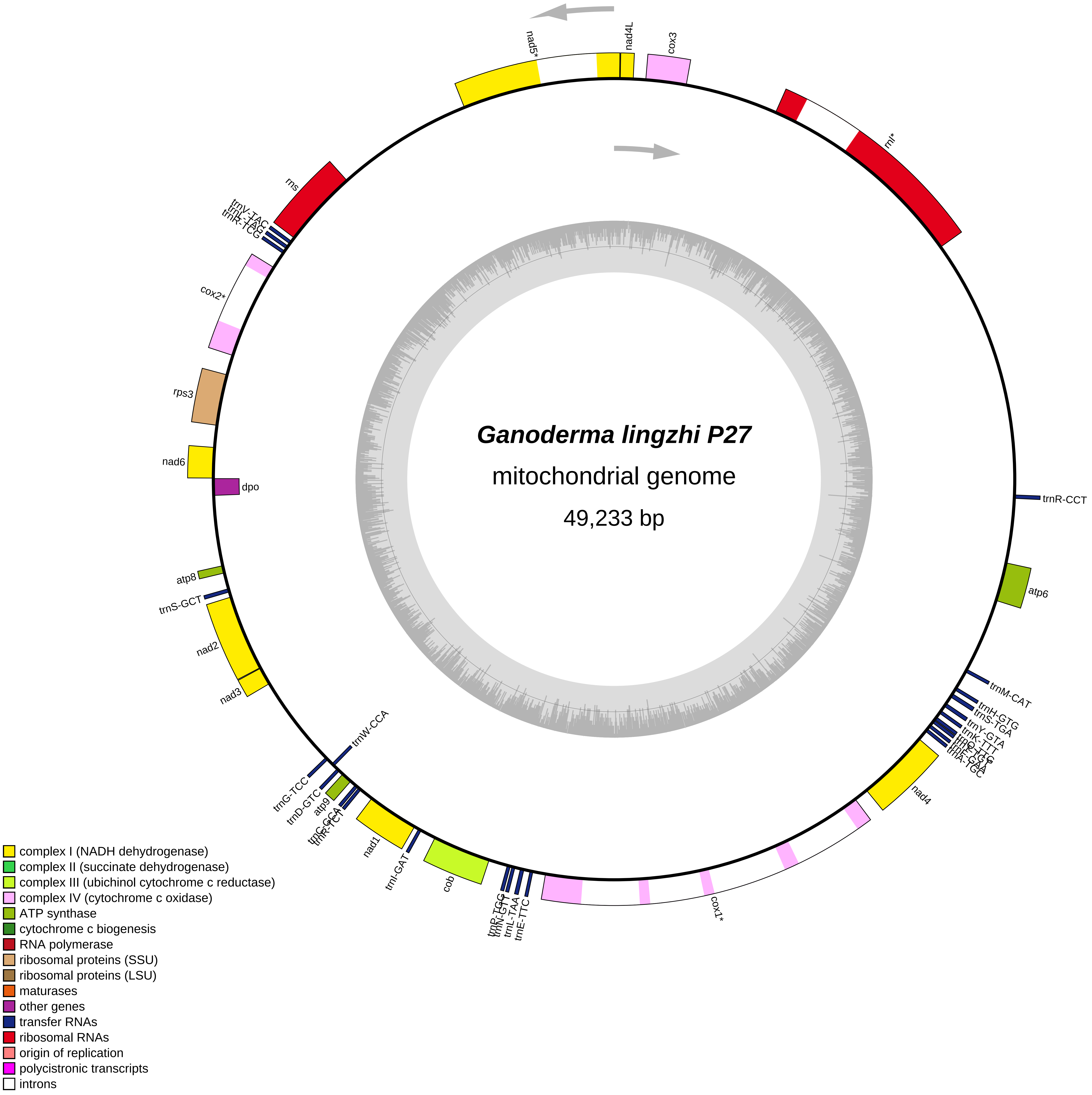

Ganoderma lingzhi P27
mitochondrial genome
49,233 bp
nad5*
nad4L
cox3
rnl*
rns
trnV-TAC
trnL-TAG
trnR-TCG
cox2*
rps3
nad6
dpo
trnR-CCT
atp6
atp8
trnS-GCT
nad2
nad3
trnM-CAT
trnH-GTG
trnS-TGA
trnY-GTA
trnK-TTT
trnA-TGC
nad4
trnW-CCA
trnG-TCC
trnD-GTC
atp9
trnC-GCA
nad1
trnI-GAT
cob
trnL-TAA
trnE-TTC
cox1*
complex I (NADH dehydrogenase)
complex II (succinate dehydrogenase)
complex III (ubichinol cytochrome c reductase)
complex IV (cytochrome c oxidase)
ATP synthase
cytochrome c biogenesis
RNA polymerase
ribosomal proteins (SSU)
ribosomal proteins (LSU)
maturases
other genes
transfer RNAs
ribosomal RNAs
origin of replication
polycistronic transcripts
introns

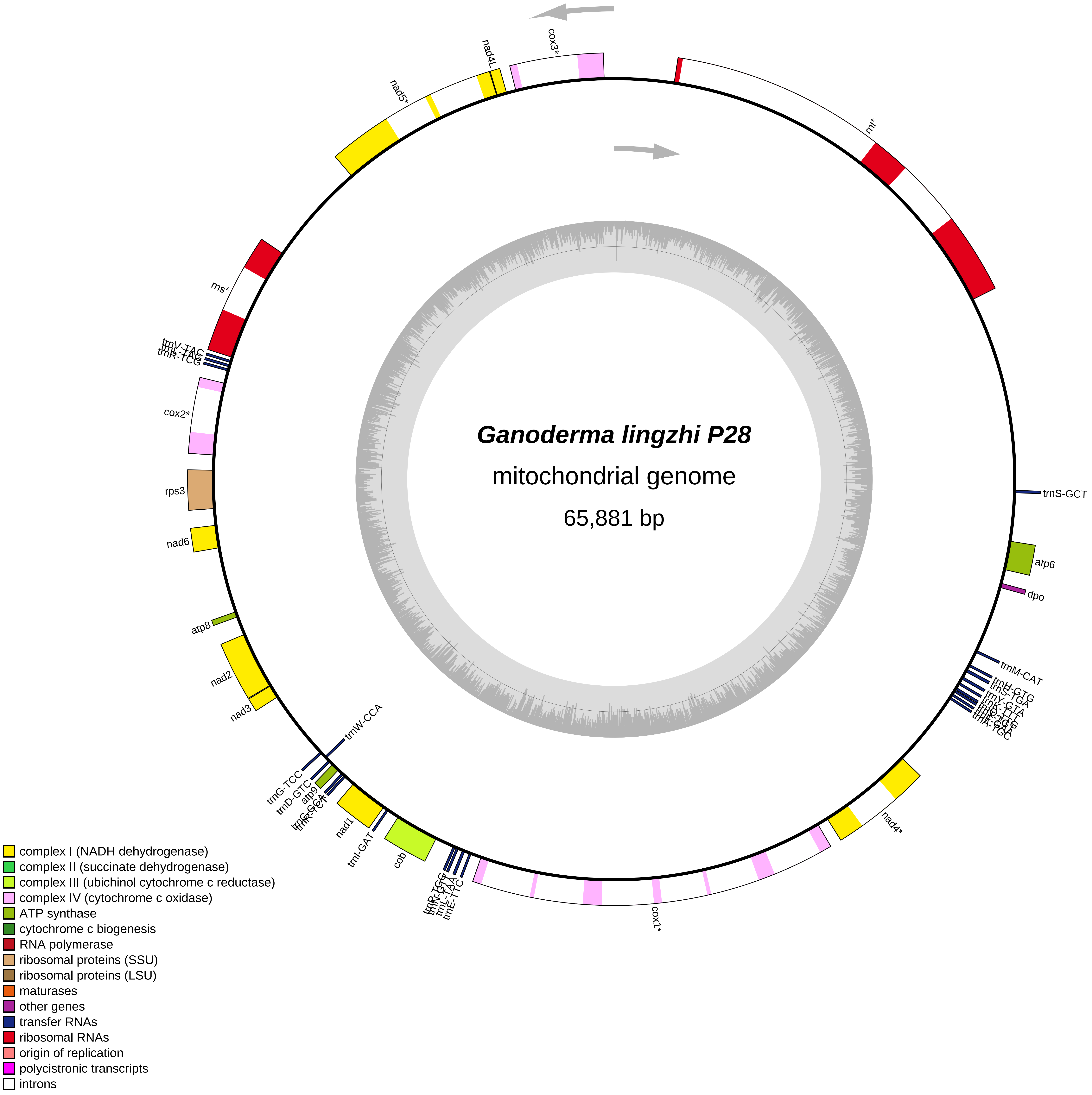
Ganoderma lingzhi P28
mitochondrial genome
65,881 bp
cox3*
nad4L
nad5*
rnl*
rns*
trnR-TCG
cox2*
rps3
nad6
atp8
nad2
nad3
trnW-CCA
trnG-TCC
trnD-GTC
atp9
nad1
trnI-GAT
cob
trnE-TTC
cox1*
nad4*
trnM-CAT
trnH-GTG
dpo
atp6
trnS-GCT
complex I (NADH dehydrogenase)
complex II (succinate dehydrogenase)
complex III (ubichinol cytochrome c reductase)
complex IV (cytochrome c oxidase)
ATP synthase
cytochrome c biogenesis
RNA polymerase
ribosomal proteins (SSU)
ribosomal proteins (LSU)
maturases
other genes
transfer RNAs
ribosomal RNAs
origin of replication
polycistronic transcripts
introns

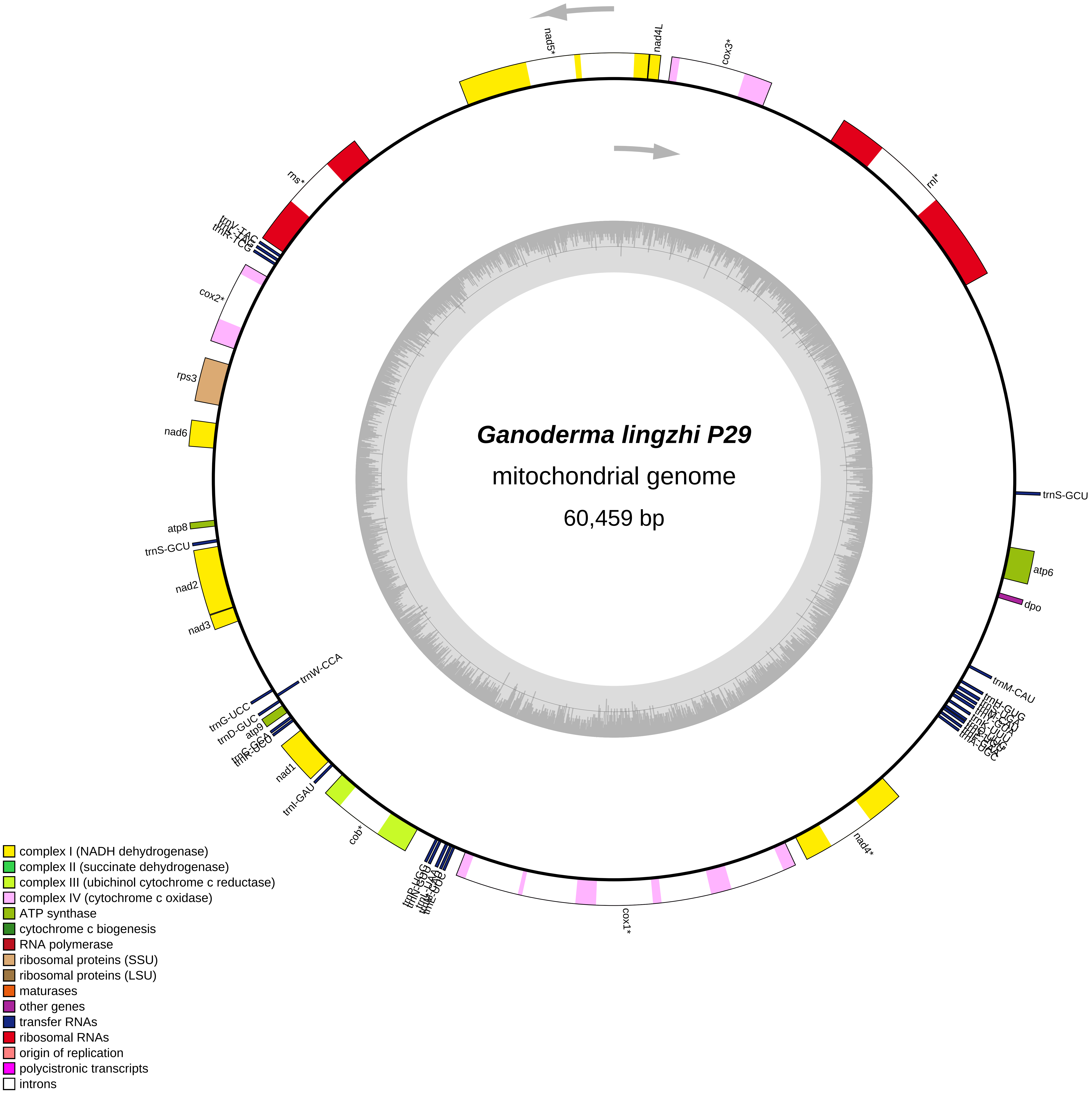

Ganoderma lingzhi P29
mitochondrial genome
60,459 bp
nad5*
nad4L
cox3*
rnl*
rns*
cox2*
rps3
nad6
atp8
trnS-GCU
nad2
nad3
trnW-CCA
trnG-UCC
trnD-GUC
atp9
nad1
trnI-GAU
cob*
cox1*
nad4*
trnM-CAU
trnH-GUG
dpo
atp6
trnS-GCU
complex I (NADH dehydrogenase)
complex II (succinate dehydrogenase)
complex III (ubichinol cytochrome c reductase)
complex IV (cytochrome c oxidase)
ATP synthase
cytochrome c biogenesis
RNA polymerase
ribosomal proteins (SSU)
ribosomal proteins (LSU)
maturases
other genes
transfer RNAs
ribosomal RNAs
origin of replication
polycistronic transcripts
introns

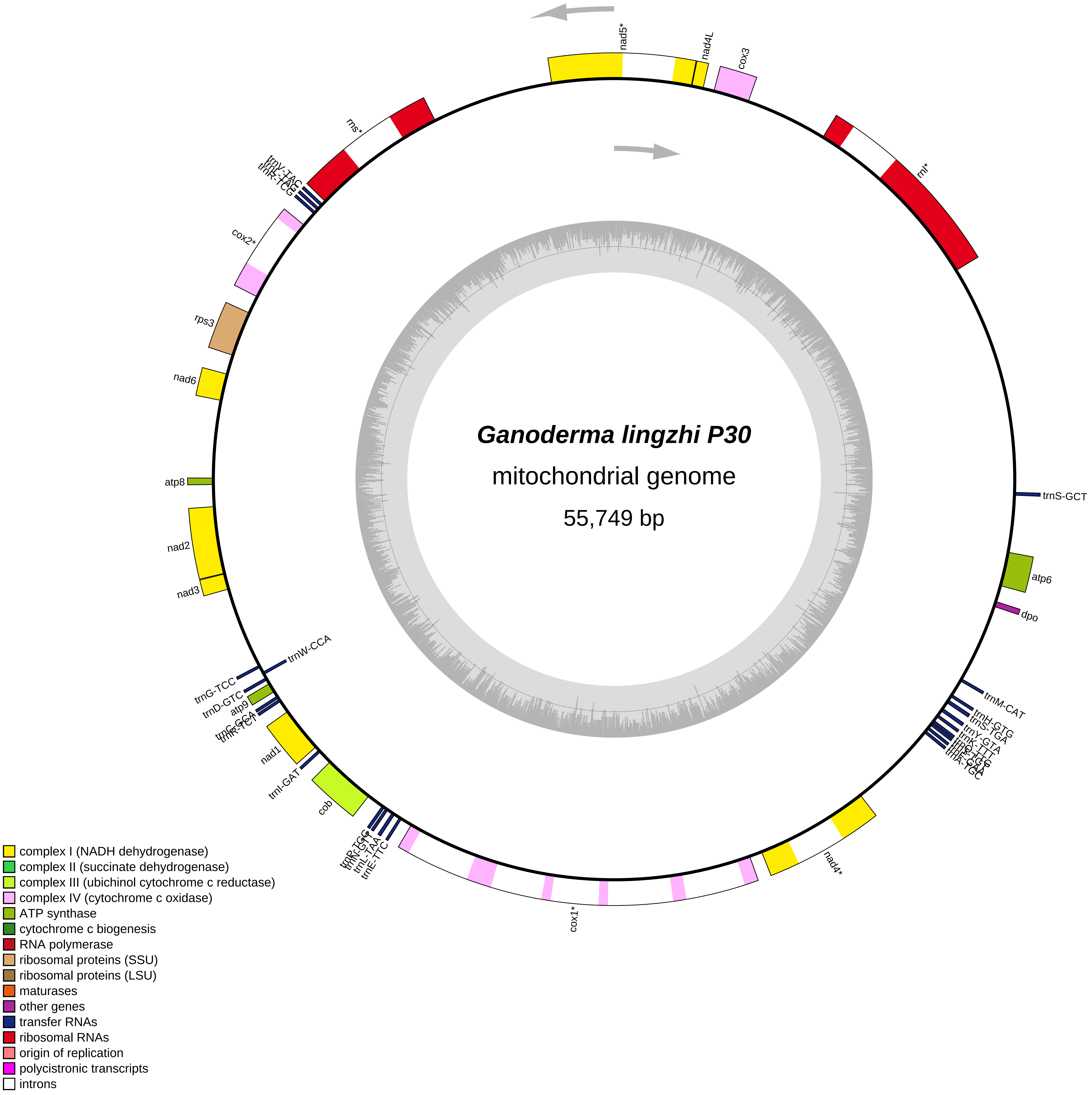
Ganoderma lingzhi P30
mitochondrial genome
55,749 bp
nad5*
nad4L
cox3
rnl*
trnS-GCT
atp6
dpo
trnM-CAT
trnH-GTG
trnS-TGA
trnY-GTA
nad4*
cox1*
trnE-TTC
trnL-TAA
cob
trnI-GAT
nad1
trnR-TCT
atp9
trnD-GTC
trnG-TCC
trnW-CCA
nad3
nad2
atp8
nad6
rps3
cox2*
trnV-TAC
trnR-TCG
rns*
complex I (NADH dehydrogenase)
complex II (succinate dehydrogenase)
complex III (ubichinol cytochrome c reductase)
complex IV (cytochrome c oxidase)
ATP synthase
cytochrome c biogenesis
RNA polymerase
ribosomal proteins (SSU)
ribosomal proteins (LSU)
maturases
other genes
transfer RNAs
ribosomal RNAs
origin of replication
polycistronic transcripts
introns

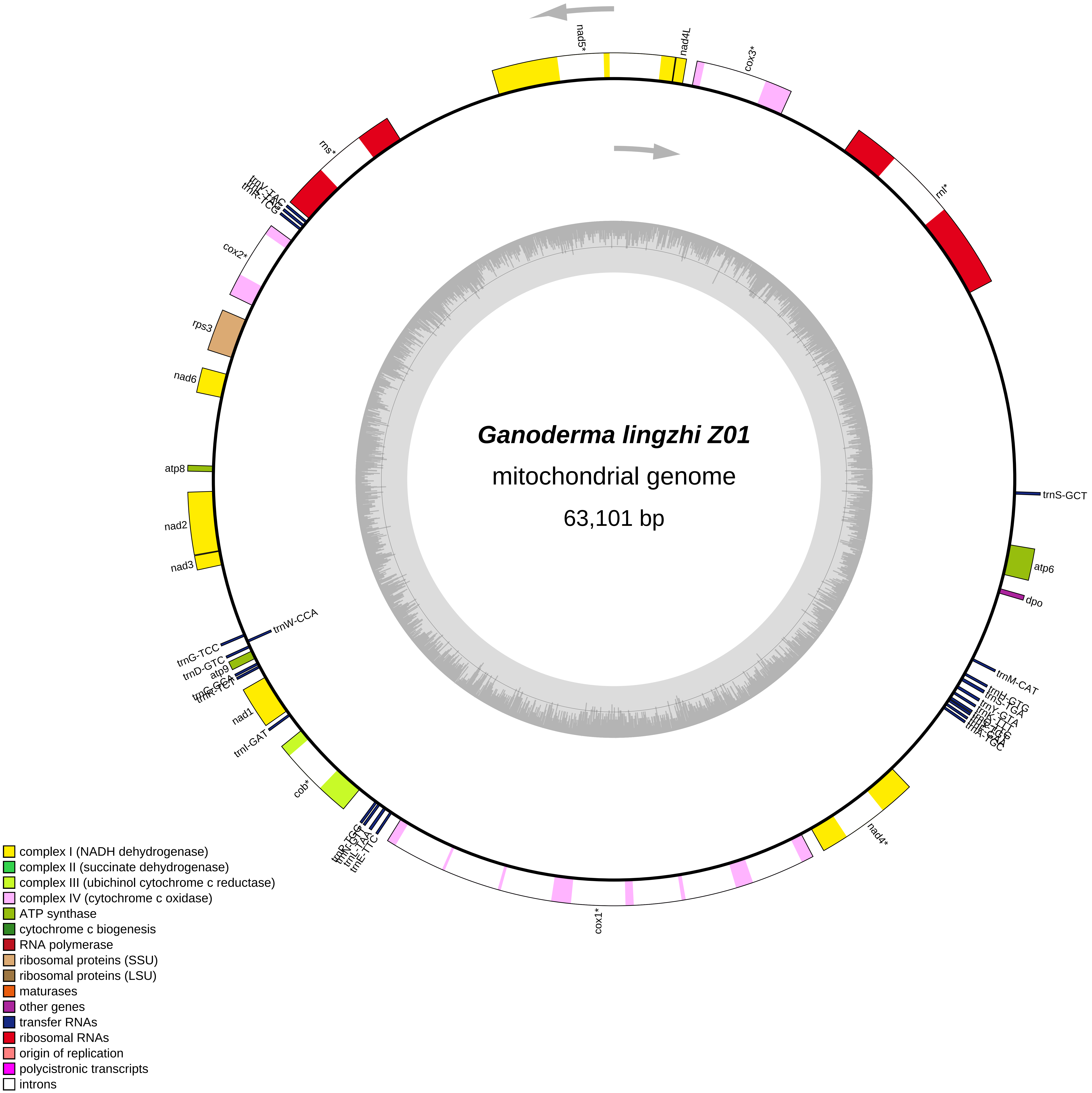

Ganoderma lingzhi Z01
mitochondrial genome
63,101 bp
nad5*
nad4L
cox3*
rnl*
rns*
cox2*
rps3
nad6
atp8
nad2
nad3
trnW-CCA
trnG-TCC
trnD-GTC
atp9
nad1
trnI-GAT
cob*
cox1*
nad4*
trnM-CAT
dpo
atp6
trnS-GCT
complex I (NADH dehydrogenase)
complex II (succinate dehydrogenase)
complex III (ubichinol cytochrome c reductase)
complex IV (cytochrome c oxidase)
ATP synthase
cytochrome c biogenesis
RNA polymerase
ribosomal proteins (SSU)
ribosomal proteins (LSU)
maturases
other genes
transfer RNAs
ribosomal RNAs
origin of replication
polycistronic transcripts
introns

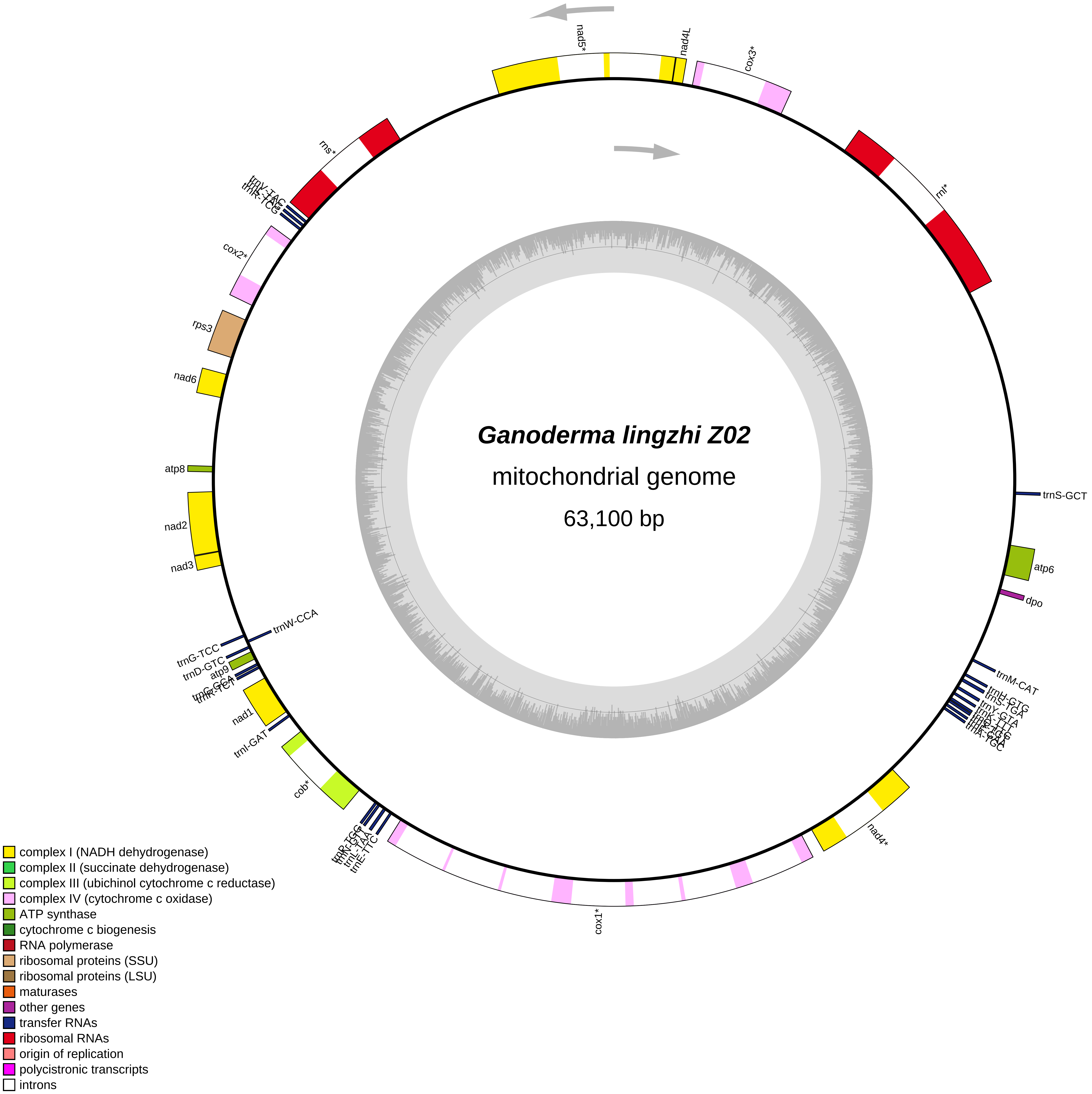

Ganoderma lingzhi Z02
mitochondrial genome
63,100 bp
nad5*
nad4L
cox3*
rnl*
trnS-GCT
atp6
dpo
trnM-CAT
trnH-GTG
nad4*
cox1*
trnE-TTC
cob*
trnI-GAT
nad1
atp9
trnD-GTC
trnG-TCC
trnW-CCA
nad3
nad2
atp8
nad6
rps3
cox2*
trnV-TAC
rns*
complex I (NADH dehydrogenase)
complex II (succinate dehydrogenase)
complex III (ubichinol cytochrome c reductase)
complex IV (cytochrome c oxidase)
ATP synthase
cytochrome c biogenesis
RNA polymerase
ribosomal proteins (SSU)
ribosomal proteins (LSU)
maturases
other genes
transfer RNAs
ribosomal RNAs
origin of replication
polycistronic transcripts
introns

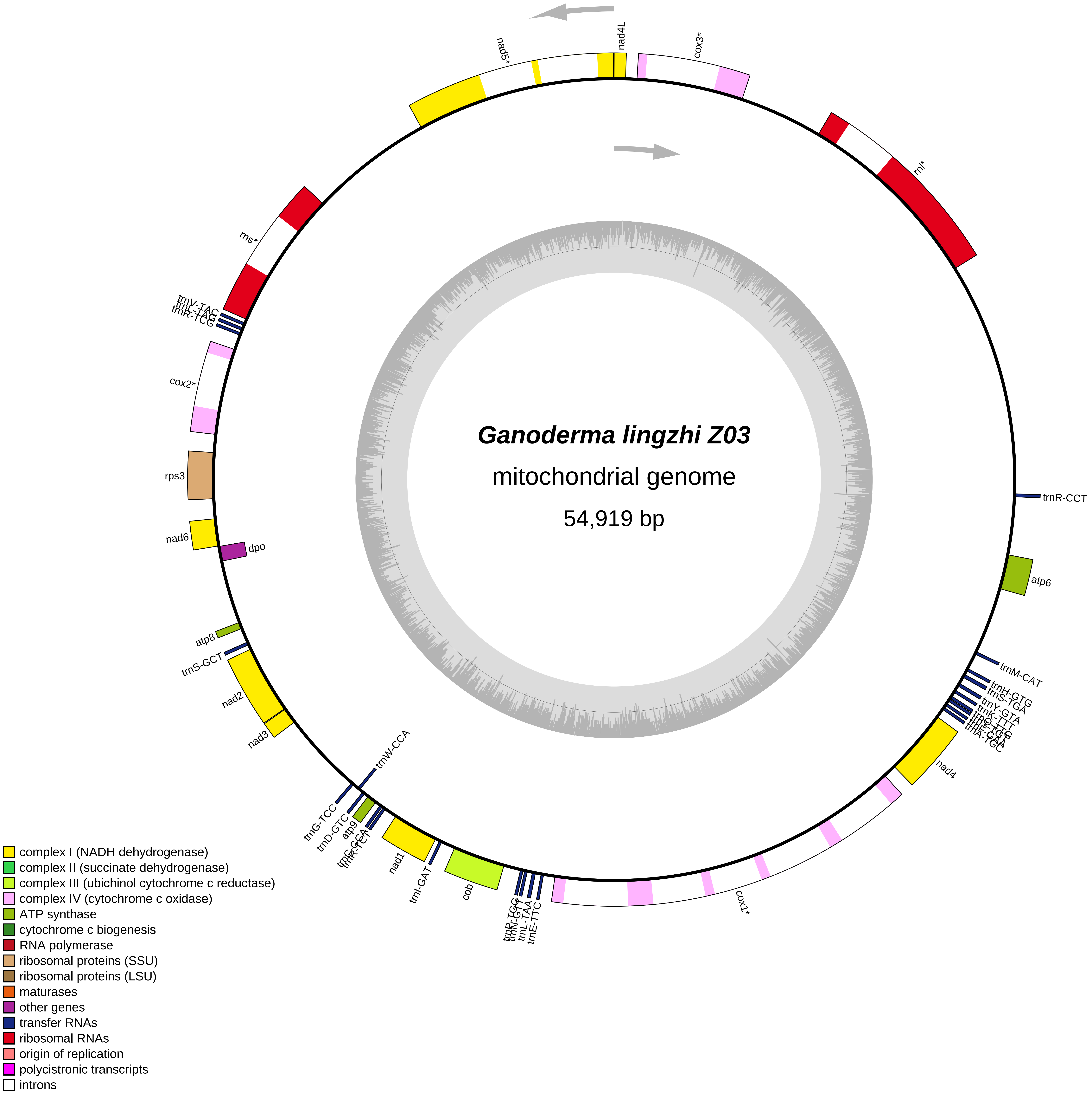

Ganoderma lingzhi Z03
mitochondrial genome
54,919 bp
nad5*
nad4L
cox3*
rnl*
rns*
trnV-TAC
trnR-TCG
cox2*
rps3
nad6
dpo
atp8
trnS-GCT
nad2
nad3
trnW-CCA
trnG-TCC
trnD-GTC
atp9
nad1
trnI-GAT
cob
trnL-TAA
trnE-TTC
cox1*
nad4
trnA-TGC
trnY-GTA
trnS-TGA
trnH-GTG
trnM-CAT
atp6
trnR-CCT
complex I (NADH dehydrogenase)
complex II (succinate dehydrogenase)
complex III (ubichinol cytochrome c reductase)
complex IV (cytochrome c oxidase)
ATP synthase
cytochrome c biogenesis
RNA polymerase
ribosomal proteins (SSU)
ribosomal proteins (LSU)
maturases
other genes
transfer RNAs
ribosomal RNAs
origin of replication
polycistronic transcripts
introns

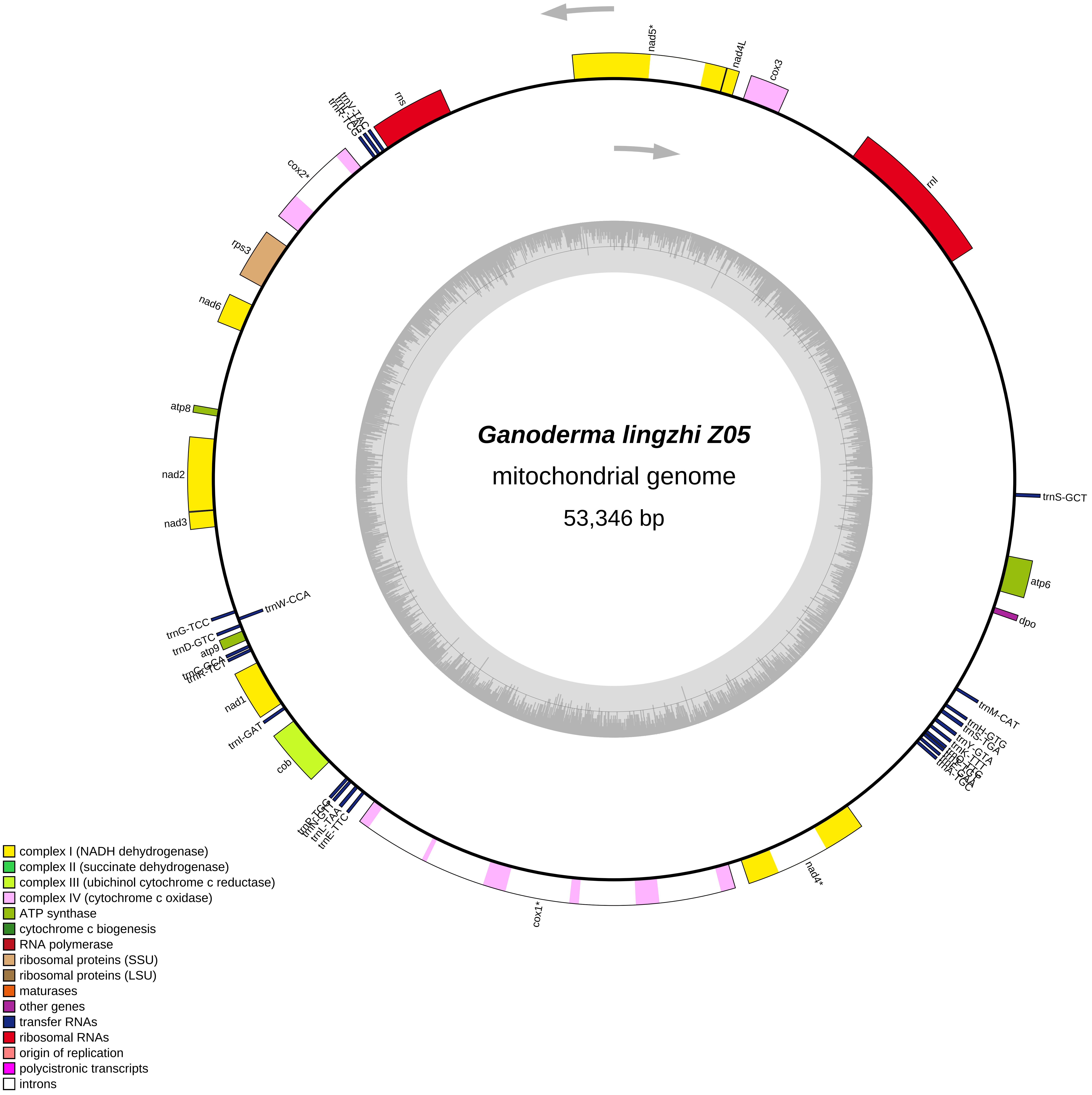
Ganoderma lingzhi Z05
mitochondrial genome
53,346 bp
nad5*
nad4L
cox3
rnl
trnS-GCT
atp6
dpo
trnM-CAT
trnH-GTG
trnS-TGA
trnY-GTA
nad4*
cox1*
trnE-TTC
trnL-TAA
cob
trnI-GAT
nad1
trnR-TCT
atp9
trnD-GTC
trnG-TCC
trnW-CCA
nad3
nad2
atp8
nad6
rps3
cox2*
rns
trnV-TAC
complex I (NADH dehydrogenase)
complex II (succinate dehydrogenase)
complex III (ubichinol cytochrome c reductase)
complex IV (cytochrome c oxidase)
ATP synthase
cytochrome c biogenesis
RNA polymerase
ribosomal proteins (SSU)
ribosomal proteins (LSU)
maturases
other genes
transfer RNAs
ribosomal RNAs
origin of replication
polycistronic transcripts
introns

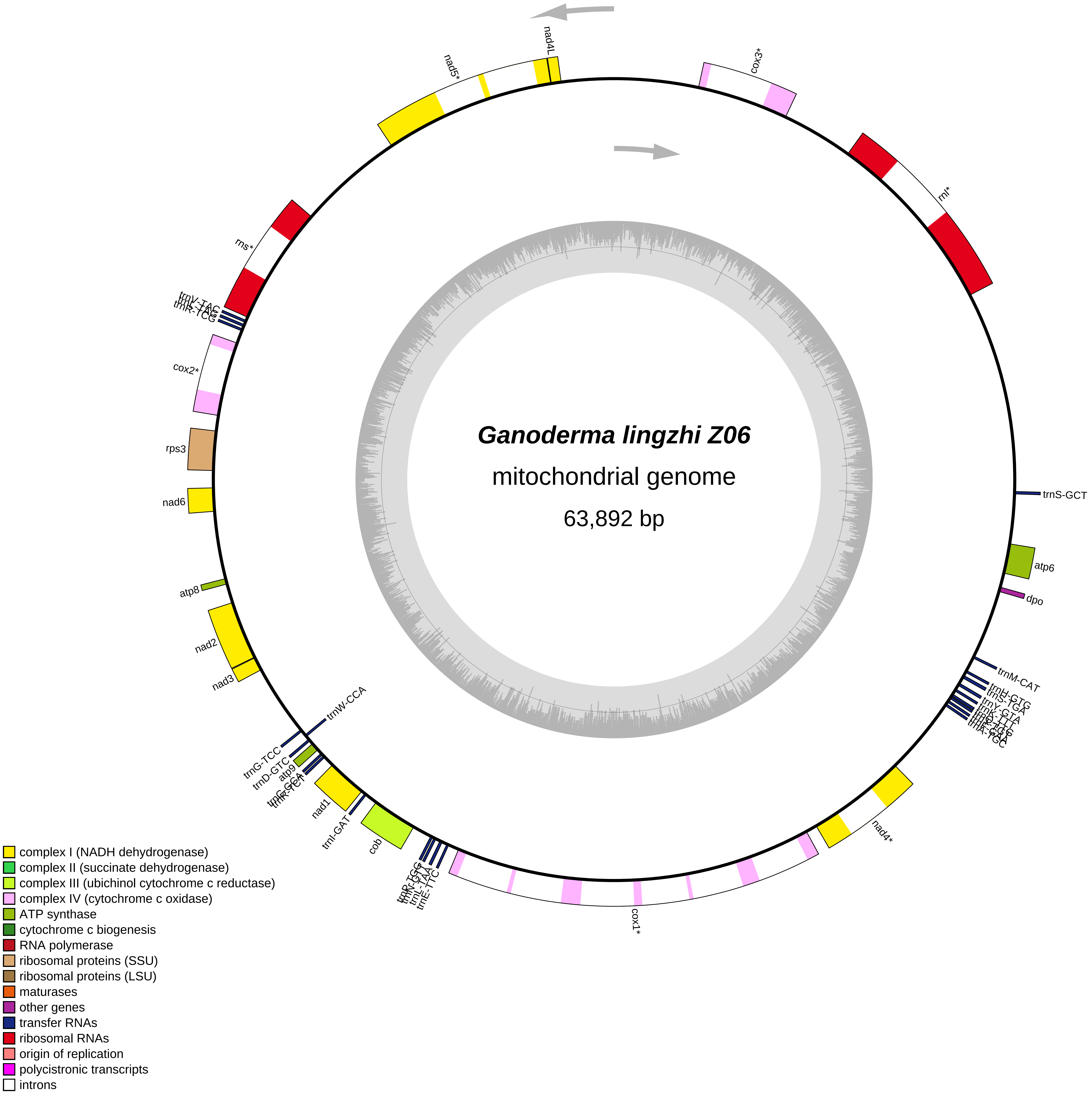

Ganoderma lingzhi Z06
mitochondrial genome
63,892 bp
nad4L
nad5*
cox3*
rnl*
rns*
cox2*
rps3
nad6
atp8
nad2
nad3
trnW-CCA
trnG-TCC
trnD-GTC
atp9
nad1
trnI-GAT
cob
cox1*
nad4*
trnM-CAT
dpo
atp6
trnS-GCT
complex I (NADH dehydrogenase)
complex II (succinate dehydrogenase)
complex III (ubichinol cytochrome c reductase)
complex IV (cytochrome c oxidase)
ATP synthase
cytochrome c biogenesis
RNA polymerase
ribosomal proteins (SSU)
ribosomal proteins (LSU)
maturases
other genes
transfer RNAs
ribosomal RNAs
origin of replication
polycistronic transcripts
introns

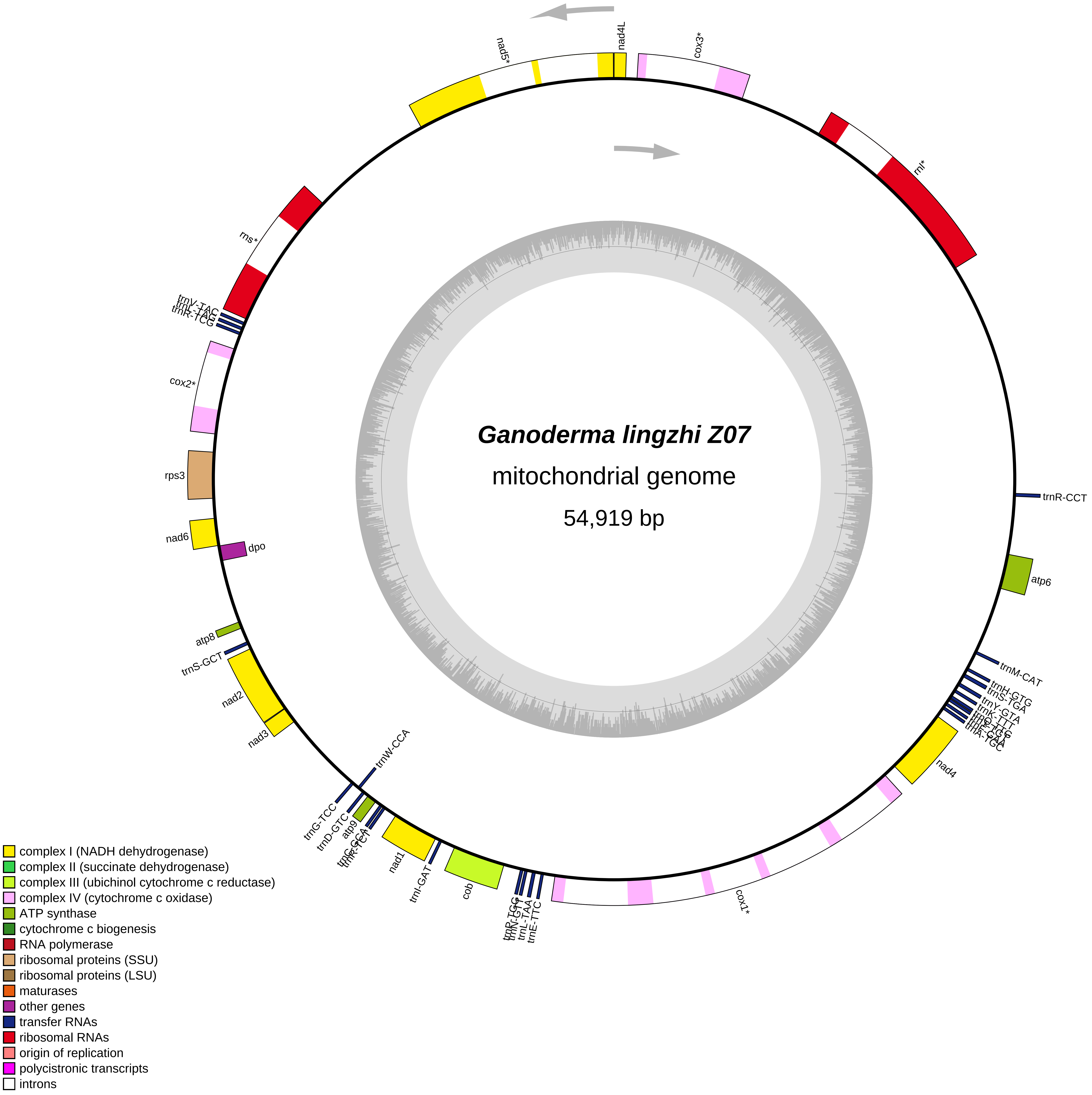
Ganoderma lingzhi Z07
mitochondrial genome
54,919 bp
nad5*
nad4L
cox3*
rnl*
trnR-CCT
atp6
trnM-CAT
trnH-GTG
trnS-TGA
trnY-GTA
nad4
cox1*
trnE-TTC
trnL-TAA
cob
trnI-GAT
nad1
atp9
trnD-GTC
trnG-TCC
trnW-CCA
nad3
nad2
trnS-GCT
atp8
dpo
nad6
rps3
cox2*
trnV-TAC
trnR-TCG
rns*
complex I (NADH dehydrogenase)
complex II (succinate dehydrogenase)
complex III (ubichinol cytochrome c reductase)
complex IV (cytochrome c oxidase)
ATP synthase
cytochrome c biogenesis
RNA polymerase
ribosomal proteins (SSU)
ribosomal proteins (LSU)
maturases
other genes
transfer RNAs
ribosomal RNAs
origin of replication
polycistronic transcripts
introns

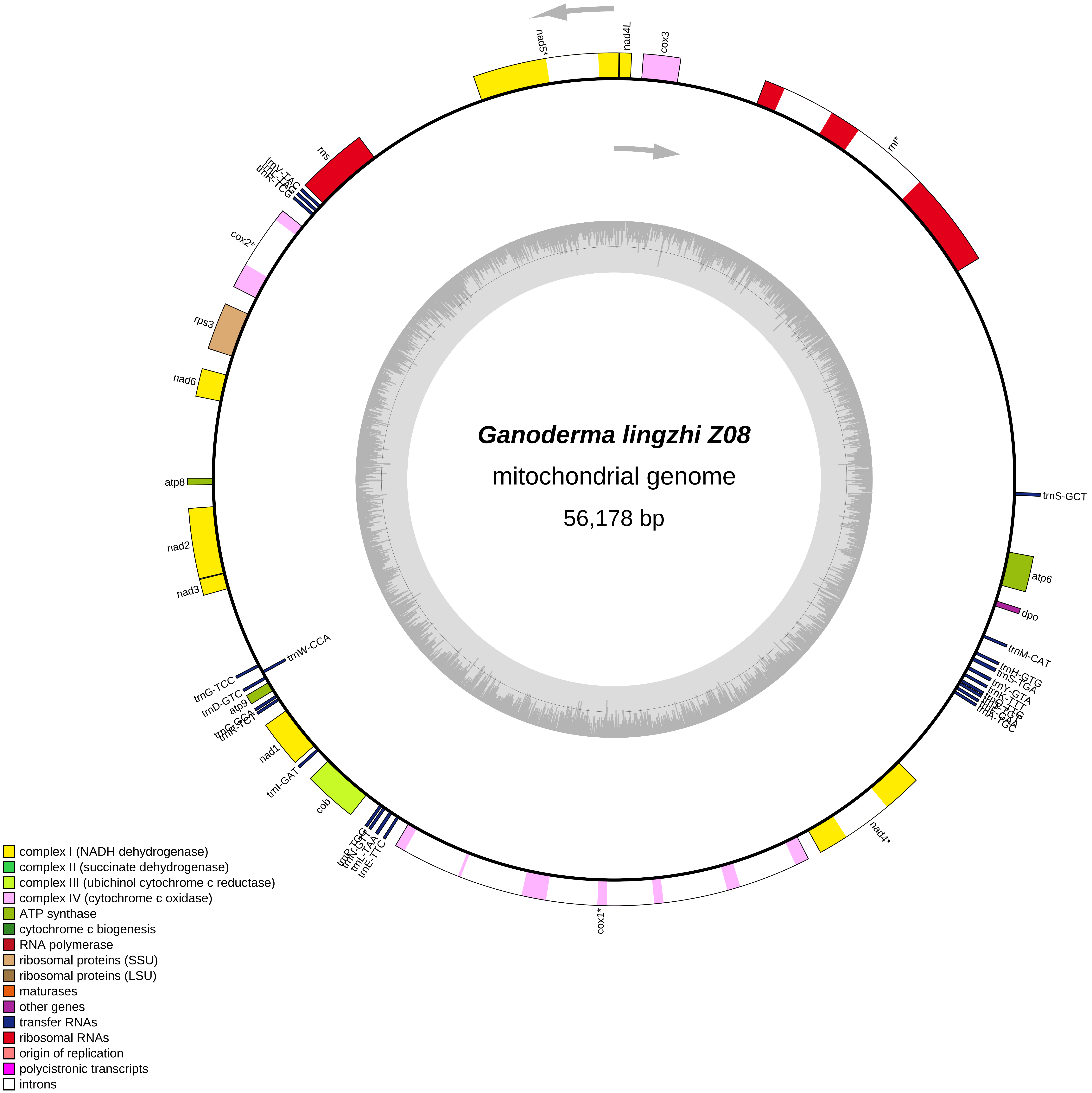

Ganoderma lingzhi Z08
mitochondrial genome
56,178 bp
nad5*
nad4L
cox3
rnl*
rns
cox2*
rps3
nad6
atp8
nad2
nad3
trnW-CCA
trnG-TCC
trnD-GTC
atp9
nad1
trnI-GAT
cob
cox1*
nad4*
trnS-GCT
atp6
dpo
trnM-CAT
trnH-GTG
trnS-TGA
trnY-GTA
complex I (NADH dehydrogenase)
complex II (succinate dehydrogenase)
complex III (ubichinol cytochrome c reductase)
complex IV (cytochrome c oxidase)
ATP synthase
cytochrome c biogenesis
RNA polymerase
ribosomal proteins (SSU)
ribosomal proteins (LSU)
maturases
other genes
transfer RNAs
ribosomal RNAs
origin of replication
polycistronic transcripts
introns

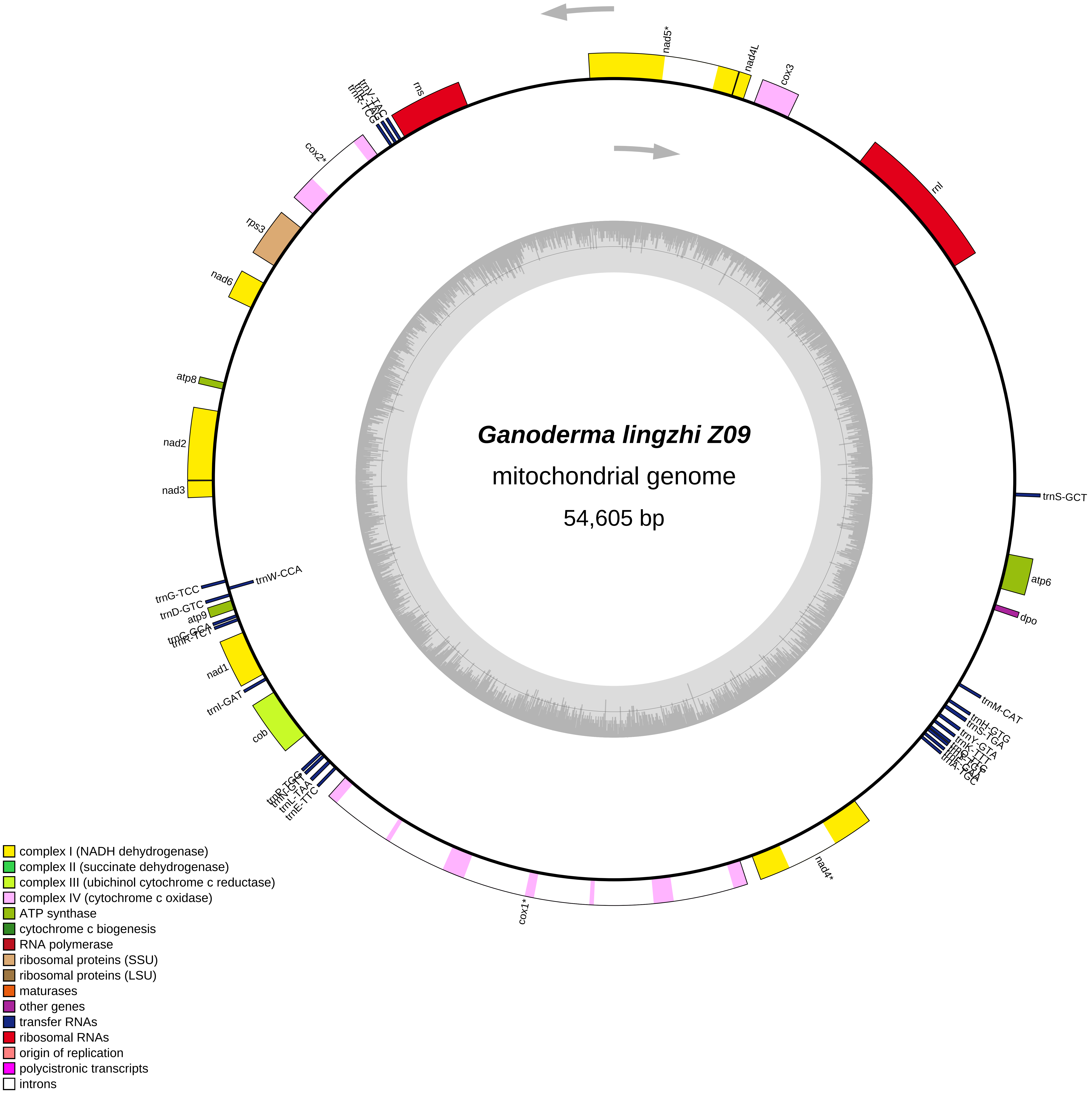
Ganoderma lingzhi Z09
mitochondrial genome
54,605 bp
nad5*
nad4L
cox3
rnl
rns
cox2*
rps3
nad6
atp8
nad2
nad3
trnS-GCT
atp6
dpo
trnM-CAT
trnH-GTG
trnS-TGA
trnY-GTA
trnW-CCA
trnG-TCC
trnD-GTC
atp9
nad1
trnI-GAT
cob
trnE-TTC
cox1*
nad4*
complex I (NADH dehydrogenase)
complex II (succinate dehydrogenase)
complex III (ubichinol cytochrome c reductase)
complex IV (cytochrome c oxidase)
ATP synthase
cytochrome c biogenesis
RNA polymerase
ribosomal proteins (SSU)
ribosomal proteins (LSU)
maturases
other genes
transfer RNAs
ribosomal RNAs
origin of replication
polycistronic transcripts
introns

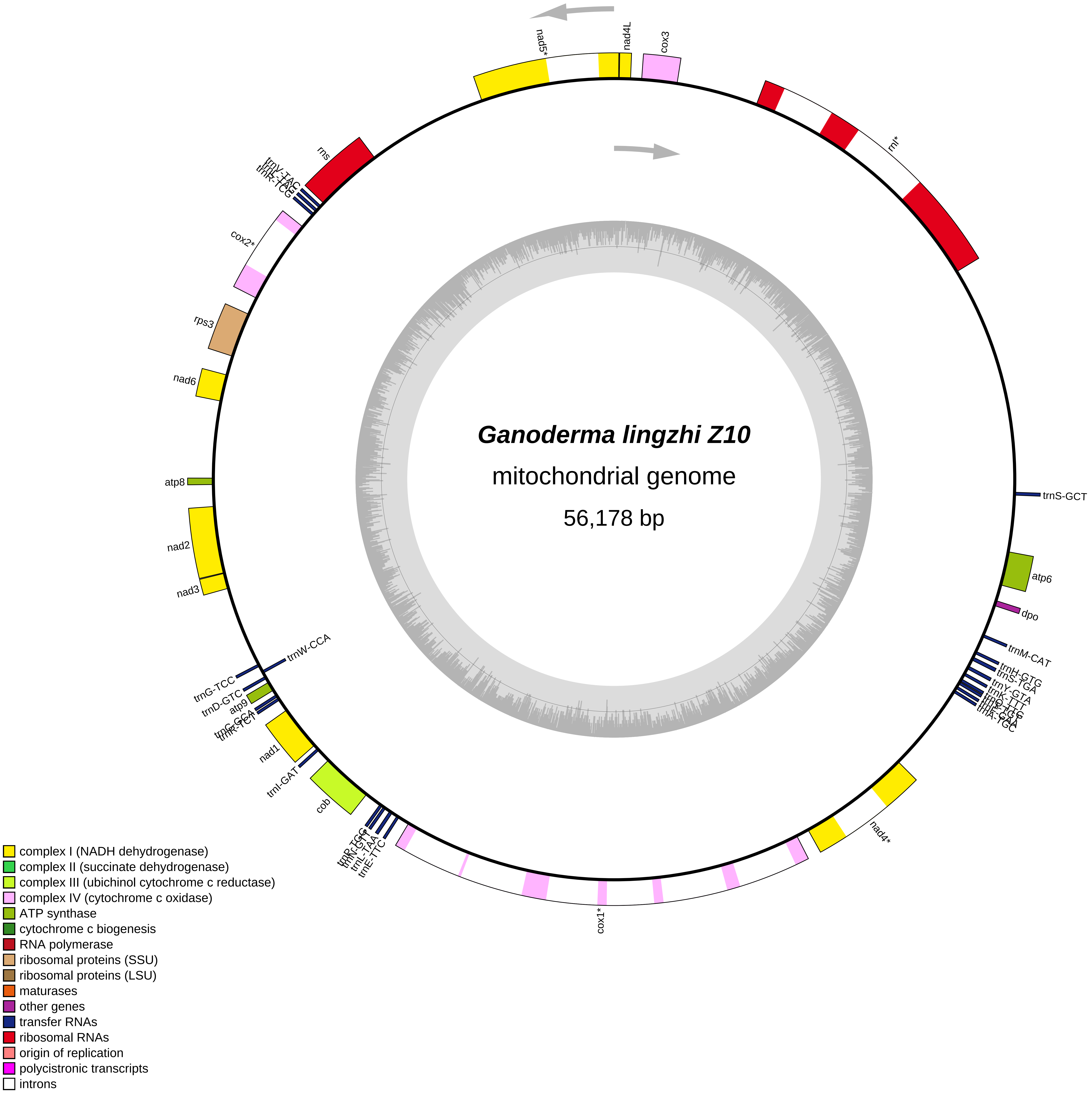

Ganoderma lingzhi Z10
mitochondrial genome
56,178 bp
nad5*
nad4L
cox3
rnl*
trnS-GCT
atp6
dpo
trnM-CAT
trnH-GTG
trnS-TGA
trnY-GTA
nad4*
cox1*
trnE-TTC
trnL-TAA
cob
trnI-GAT
nad1
atp9
trnD-GTC
trnG-TCC
trnW-CCA
nad3
nad2
atp8
nad6
rps3
cox2*
trnV-TAC
rns
complex I (NADH dehydrogenase)
complex II (succinate dehydrogenase)
complex III (ubichinol cytochrome c reductase)
complex IV (cytochrome c oxidase)
ATP synthase
cytochrome c biogenesis
RNA polymerase
ribosomal proteins (SSU)
ribosomal proteins (LSU)
maturases
other genes
transfer RNAs
ribosomal RNAs
origin of replication
polycistronic transcripts
introns

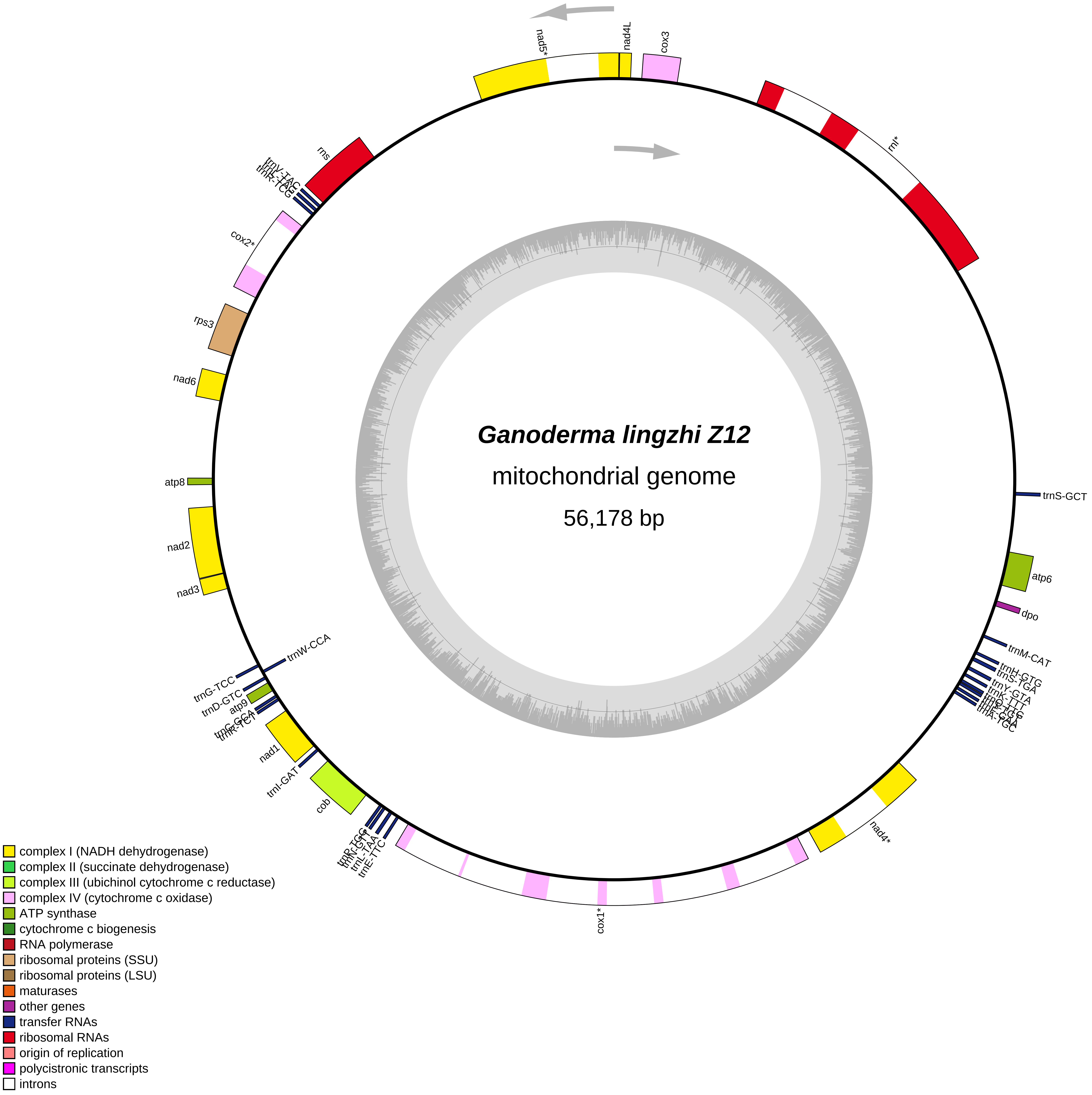

Ganoderma lingzhi Z12
mitochondrial genome
56,178 bp
nad5*
nad4L
cox3
rnl*
rns
trnV-TAC
cox2*
rps3
nad6
atp8
nad2
nad3
trnW-CCA
trnG-TCC
trnD-GTC
atp9
trnC-GCA
nad1
trnI-GAT
cob
trnR-TCG
trnE-TTC
cox1*
nad4*
trnS-GCT
atp6
dpo
trnM-CAT
trnH-GTG
trnS-TGA
trnY-GTA
trnA-TGC
complex I (NADH dehydrogenase)
complex II (succinate dehydrogenase)
complex III (ubichinol cytochrome c reductase)
complex IV (cytochrome c oxidase)
ATP synthase
cytochrome c biogenesis
RNA polymerase
ribosomal proteins (SSU)
ribosomal proteins (LSU)
maturases
other genes
transfer RNAs
ribosomal RNAs
origin of replication
polycistronic transcripts
introns

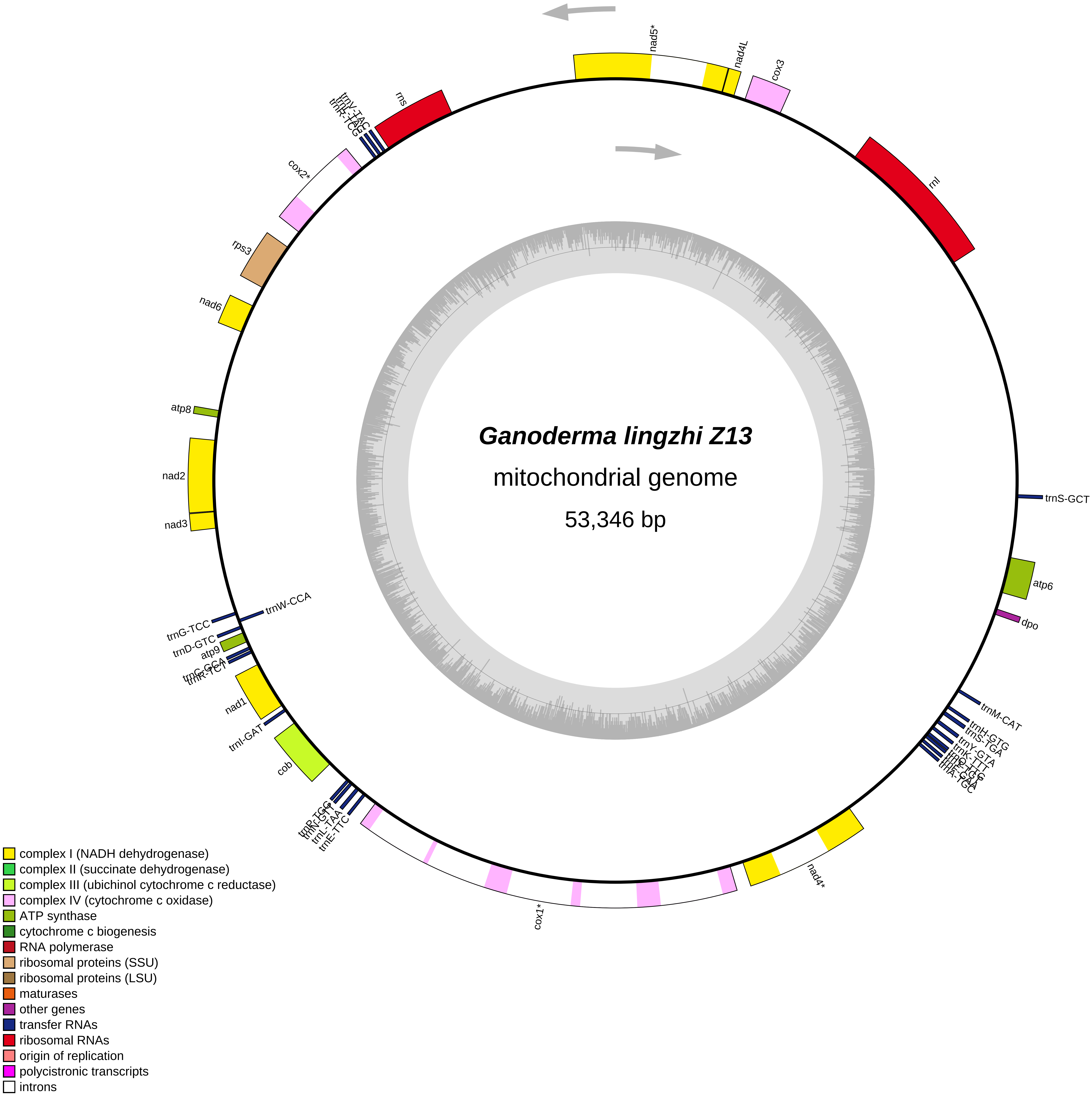

Ganoderma lingzhi Z13
mitochondrial genome
53,346 bp
nad5*
nad4L
cox3
rnl
trnS-GCT
atp6
dpo
trnM-CAT
trnH-GTG
trnS-TGA
trnY-GTA
nad4*
cox1*
trnE-TTC
trnL-TAA
cob
trnI-GAT
nad1
trnR-TCT
atp9
trnD-GTC
trnG-TCC
trnW-CCA
nad3
nad2
atp8
nad6
rps3
cox2*
rns
trnV-TAC
complex I (NADH dehydrogenase)
complex II (succinate dehydrogenase)
complex III (ubichinol cytochrome c reductase)
complex IV (cytochrome c oxidase)
ATP synthase
cytochrome c biogenesis
RNA polymerase
ribosomal proteins (SSU)
ribosomal proteins (LSU)
maturases
other genes
transfer RNAs
ribosomal RNAs
origin of replication
polycistronic transcripts
introns

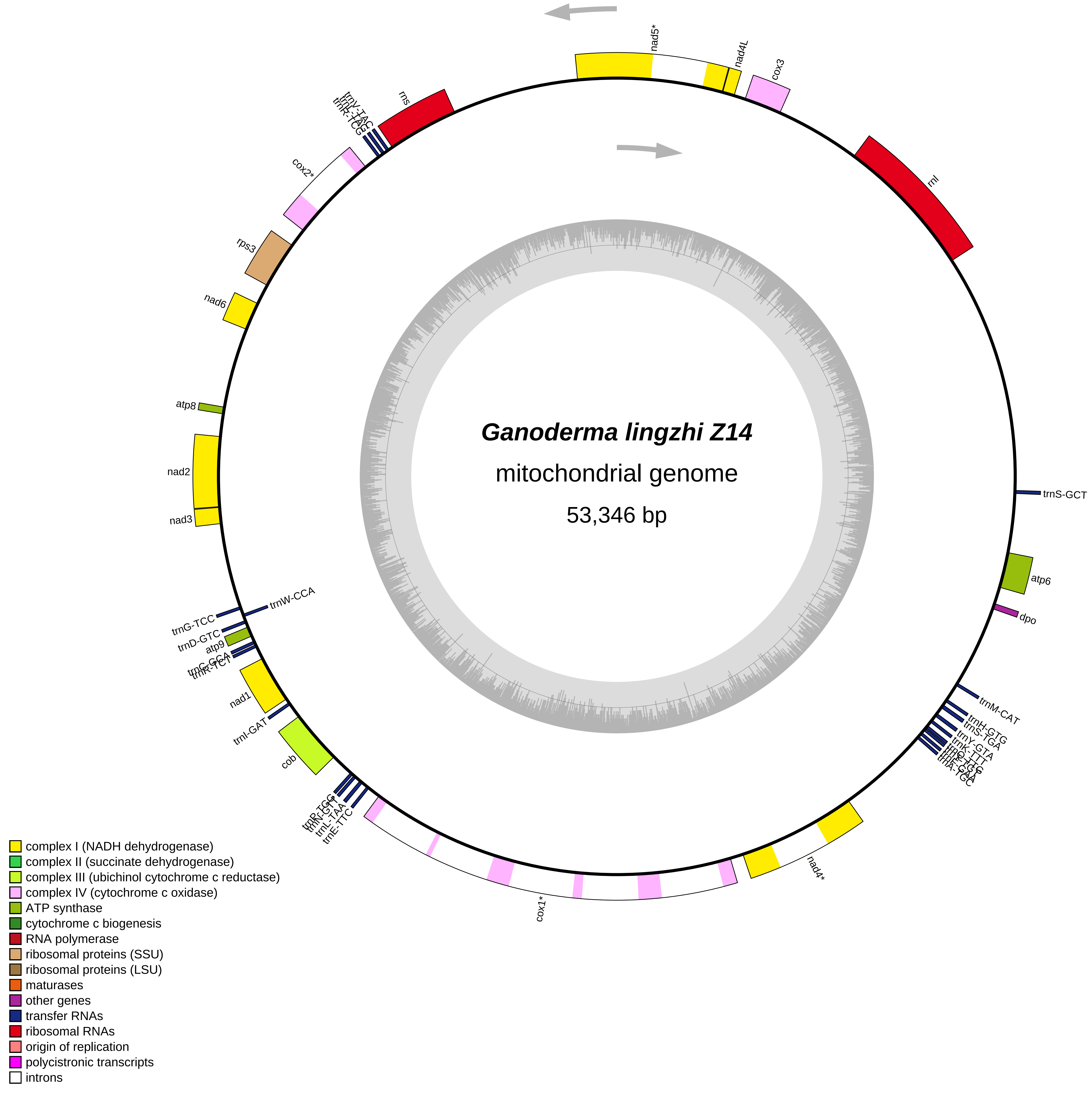

Ganoderma lingzhi Z14
mitochondrial genome
53,346 bp
nad5*
nad4L
cox3
rnl
trnS-GCT
atp6
dpo
trnM-CAT
trnH-GTG
trnS-TGA
trnY-GTA
nad4*
cox1*
trnE-TTC
trnL-TAA
cob
trnI-GAT
nad1
trnR-TCT
atp9
trnD-GTC
trnG-TCC
trnW-CCA
nad3
nad2
atp8
nad6
rps3
cox2*
rns
trnV-TAC
complex I (NADH dehydrogenase)
complex II (succinate dehydrogenase)
complex III (ubichinol cytochrome c reductase)
complex IV (cytochrome c oxidase)
ATP synthase
cytochrome c biogenesis
RNA polymerase
ribosomal proteins (SSU)
ribosomal proteins (LSU)
maturases
other genes
transfer RNAs
ribosomal RNAs
origin of replication
polycistronic transcripts
introns

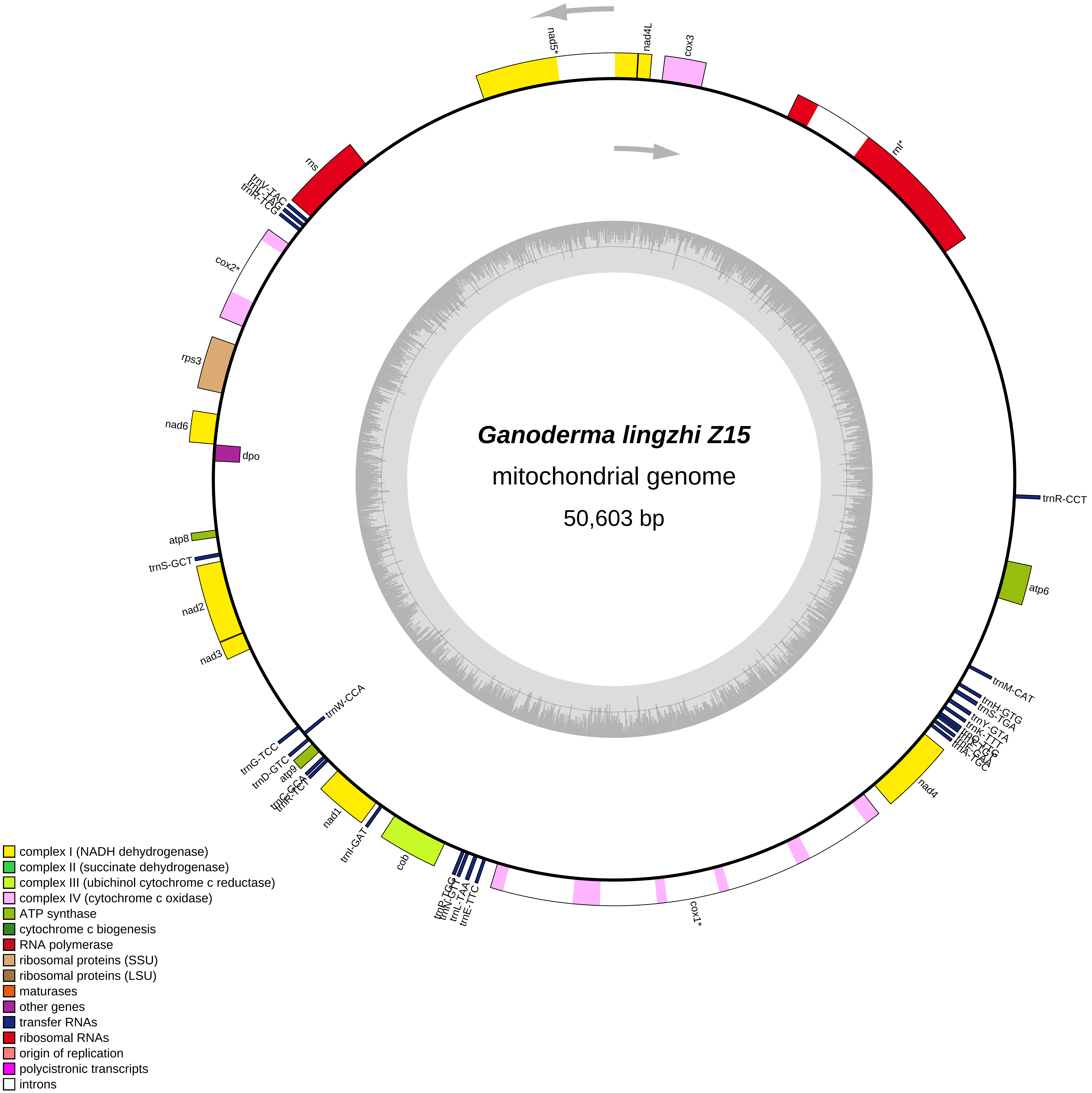

Ganoderma lingzhi Z15
mitochondrial genome
50,603 bp
nad5*
nad4L
cox3
rnl*
rns
cox2*
rps3
nad6
dpo
trnR-CCT
atp8
trnS-GCT
atp6
nad2
nad3
trnW-CCA
trnM-CAT
trnG-TCC
trnD-GTC
atp9
trnH-GTG
trnS-TGA
trnY-GTA
trnK-TTT
trnA-TGC
nad4
nad1
trnI-GAT
cob
trnL-TAA
trnE-TTC
cox1*
complex I (NADH dehydrogenase)
complex II (succinate dehydrogenase)
complex III (ubichinol cytochrome c reductase)
complex IV (cytochrome c oxidase)
ATP synthase
cytochrome c biogenesis
RNA polymerase
ribosomal proteins (SSU)
ribosomal proteins (LSU)
maturases
other genes
transfer RNAs
ribosomal RNAs
origin of replication
polycistronic transcripts
introns

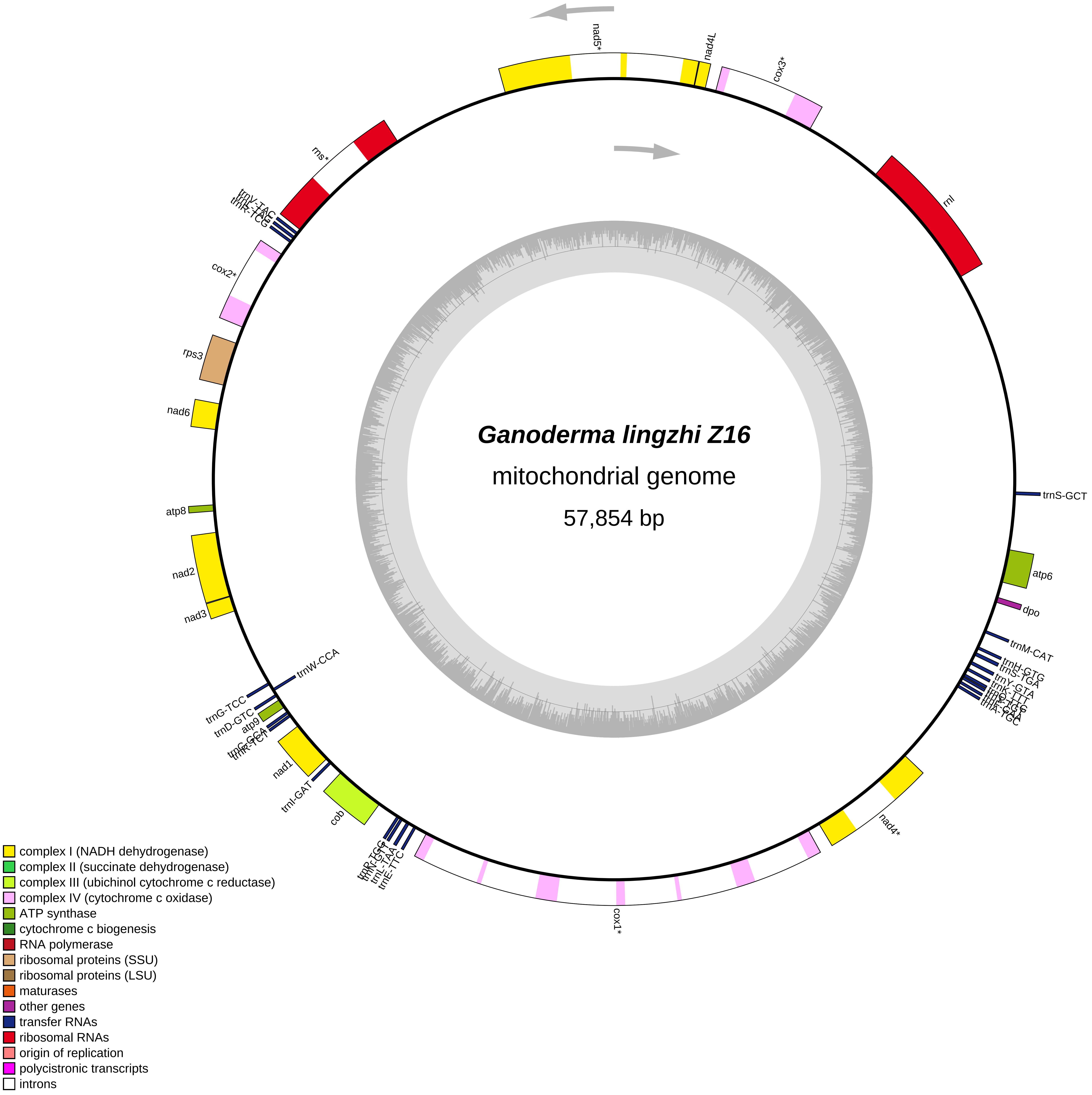

Ganoderma lingzhi Z16
mitochondrial genome
57,854 bp
nad5*
nad4L
cox3*
rnl
trnS-GCT
atp6
dpo
trnM-CAT
nad4*
cox1*
cob
trnI-GAT
nad1
atp9
trnD-GTC
trnG-TCC
trnW-CCA
nad3
nad2
atp8
nad6
rps3
cox2*
rns*
complex I (NADH dehydrogenase)
complex II (succinate dehydrogenase)
complex III (ubichinol cytochrome c reductase)
complex IV (cytochrome c oxidase)
ATP synthase
cytochrome c biogenesis
RNA polymerase
ribosomal proteins (SSU)
ribosomal proteins (LSU)
maturases
other genes
transfer RNAs
ribosomal RNAs
origin of replication
polycistronic transcripts
introns

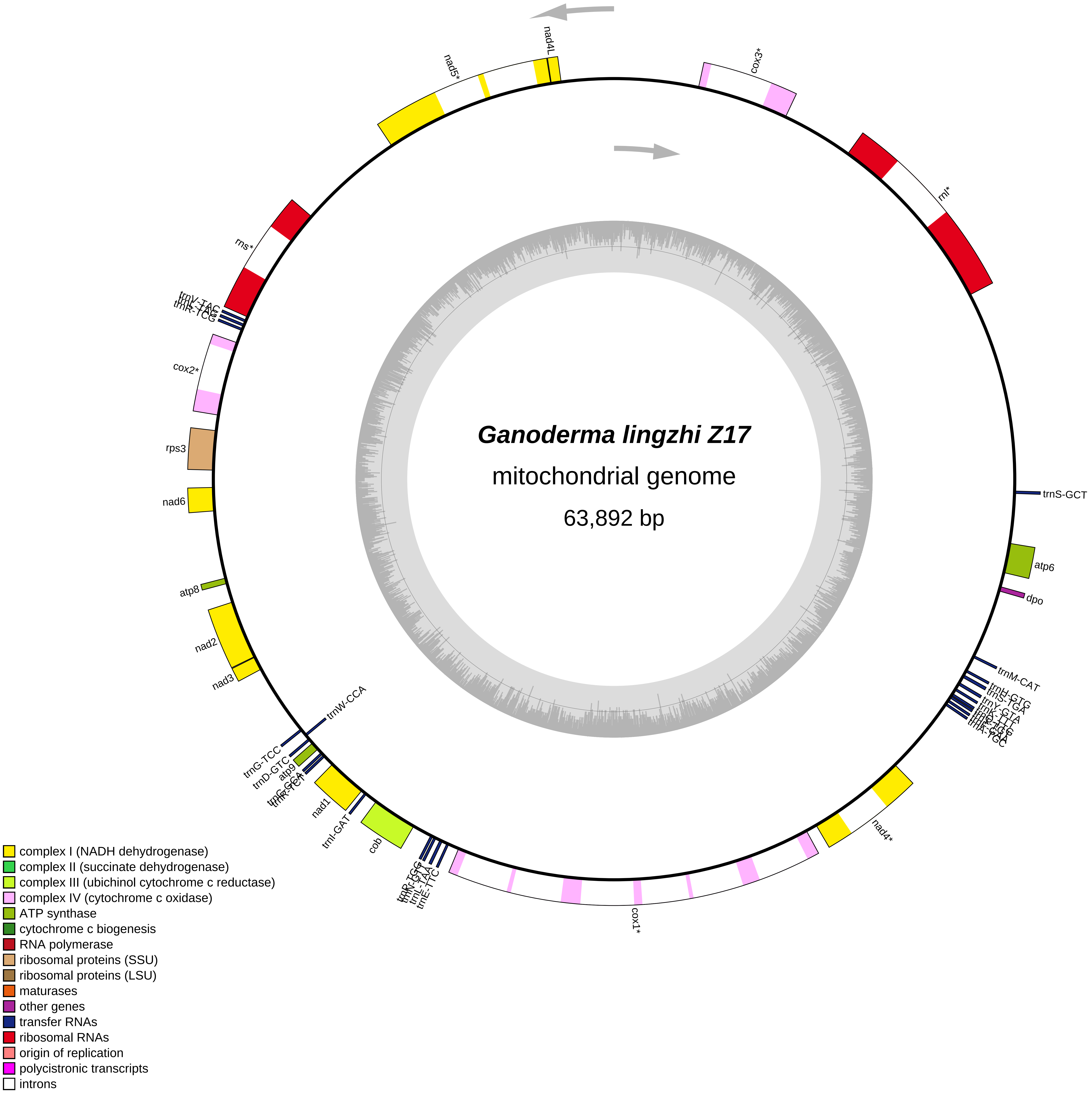

Ganoderma lingzhi Z17
mitochondrial genome
63,892 bp
nad4L
nad5*
cox3*
rnl*
rns*
cox2*
rps3
nad6
atp8
nad2
nad3
trnW-CCA
trnG-TCC
trnD-GTC
atp9
nad1
trnI-GAT
cob
trnE-TTC
cox1*
nad4*
trnM-CAT
dpo
atp6
trnS-GCT
complex I (NADH dehydrogenase)
complex II (succinate dehydrogenase)
complex III (ubichinol cytochrome c reductase)
complex IV (cytochrome c oxidase)
ATP synthase
cytochrome c biogenesis
RNA polymerase
ribosomal proteins (SSU)
ribosomal proteins (LSU)
maturases
other genes
transfer RNAs
ribosomal RNAs
origin of replication
polycistronic transcripts
introns

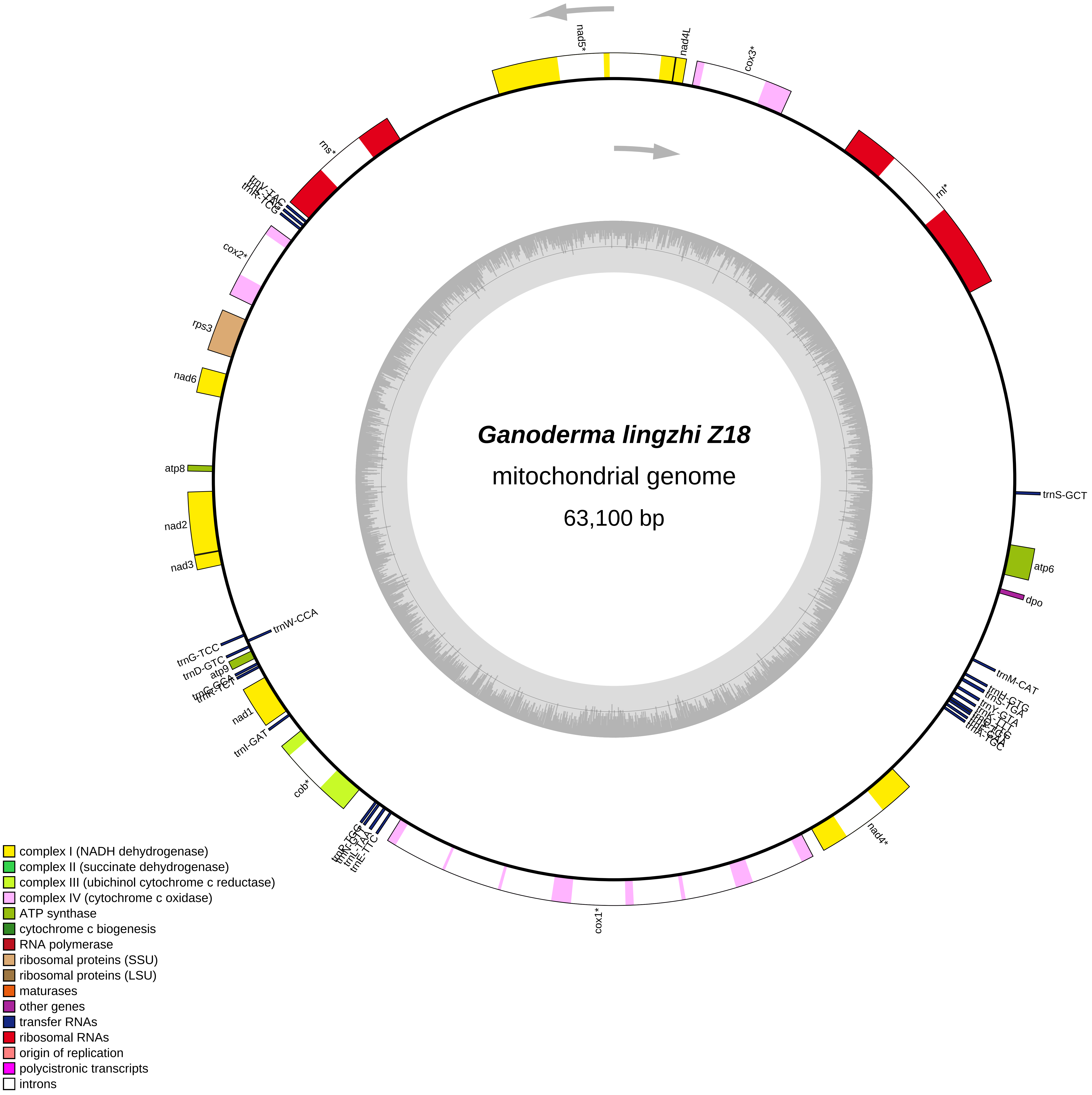

Ganoderma lingzhi Z18
mitochondrial genome
63,100 bp
nad5*
nad4L
cox3*
rnl*
rns*
cox2*
rps3
nad6
atp8
nad2
nad3
trnW-CCA
trnG-TCC
trnD-GTC
atp9
nad1
trnI-GAT
cob*
cox1*
nad4*
trnM-CAT
trnH-GTG
trnS-TGA
trnS-GCT
atp6
dpo
complex I (NADH dehydrogenase)
complex II (succinate dehydrogenase)
complex III (ubichinol cytochrome c reductase)
complex IV (cytochrome c oxidase)
ATP synthase
cytochrome c biogenesis
RNA polymerase
ribosomal proteins (SSU)
ribosomal proteins (LSU)
maturases
other genes
transfer RNAs
ribosomal RNAs
origin of replication
polycistronic transcripts
introns

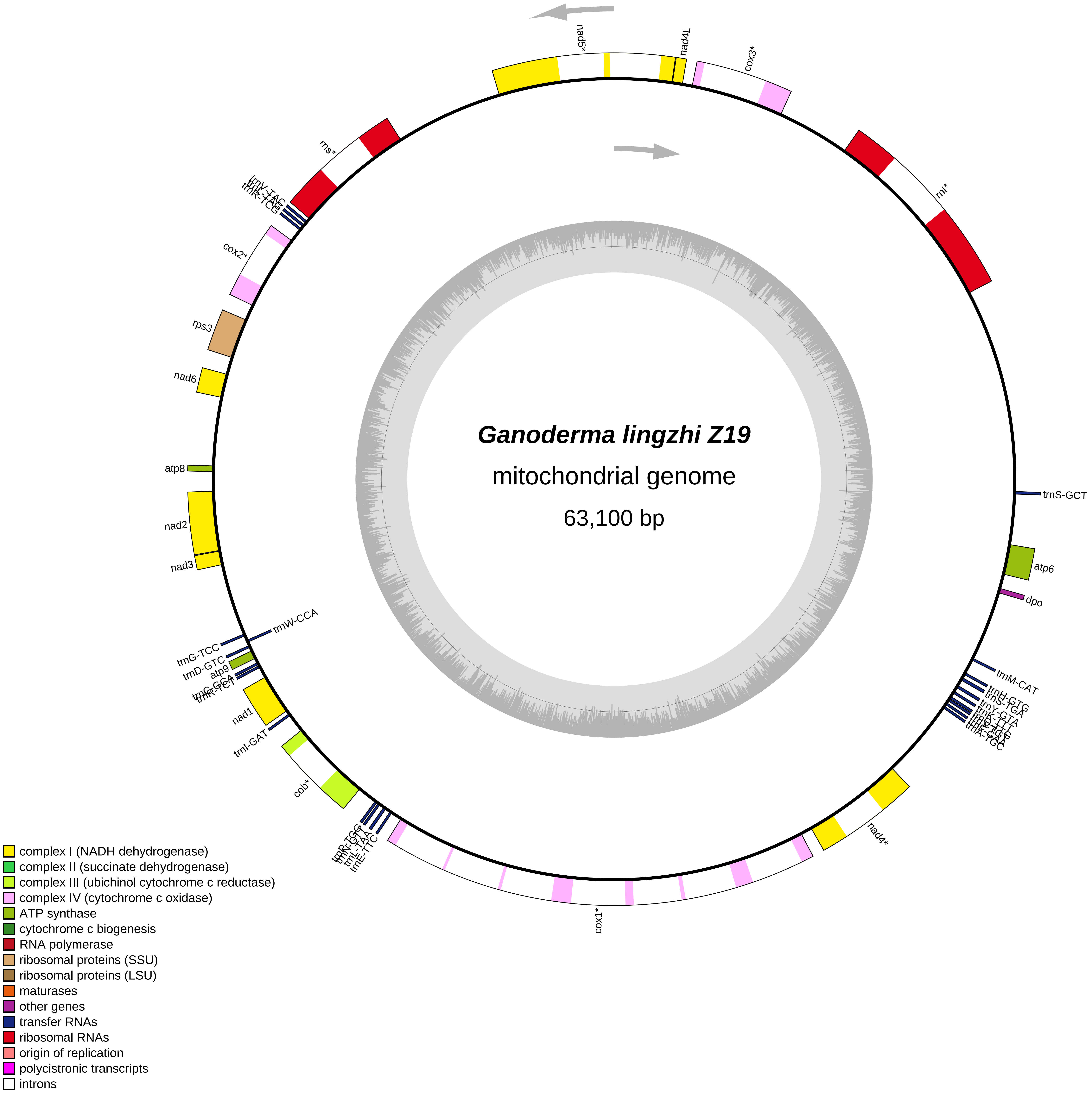

Ganoderma lingzhi Z19
mitochondrial genome
63,100 bp
nad5*
nad4L
cox3*
rnl*
trnS-GCT
atp6
dpo
trnM-CAT
trnH-GTG
trnS-TGA
trnY-GTA
nad4*
cox1*
trnE-TTC
trnL-TAA
cob*
trnI-GAT
nad1
trnR-TCT
atp9
trnD-GTC
trnG-TCC
trnW-CCA
nad3
nad2
atp8
nad6
rps3
cox2*
trnR-TCG
trnV-TAC
rns*
complex I (NADH dehydrogenase)
complex II (succinate dehydrogenase)
complex III (ubichinol cytochrome c reductase)
complex IV (cytochrome c oxidase)
ATP synthase
cytochrome c biogenesis
RNA polymerase
ribosomal proteins (SSU)
ribosomal proteins (LSU)
maturases
other genes
transfer RNAs
ribosomal RNAs
origin of replication
polycistronic transcripts
introns

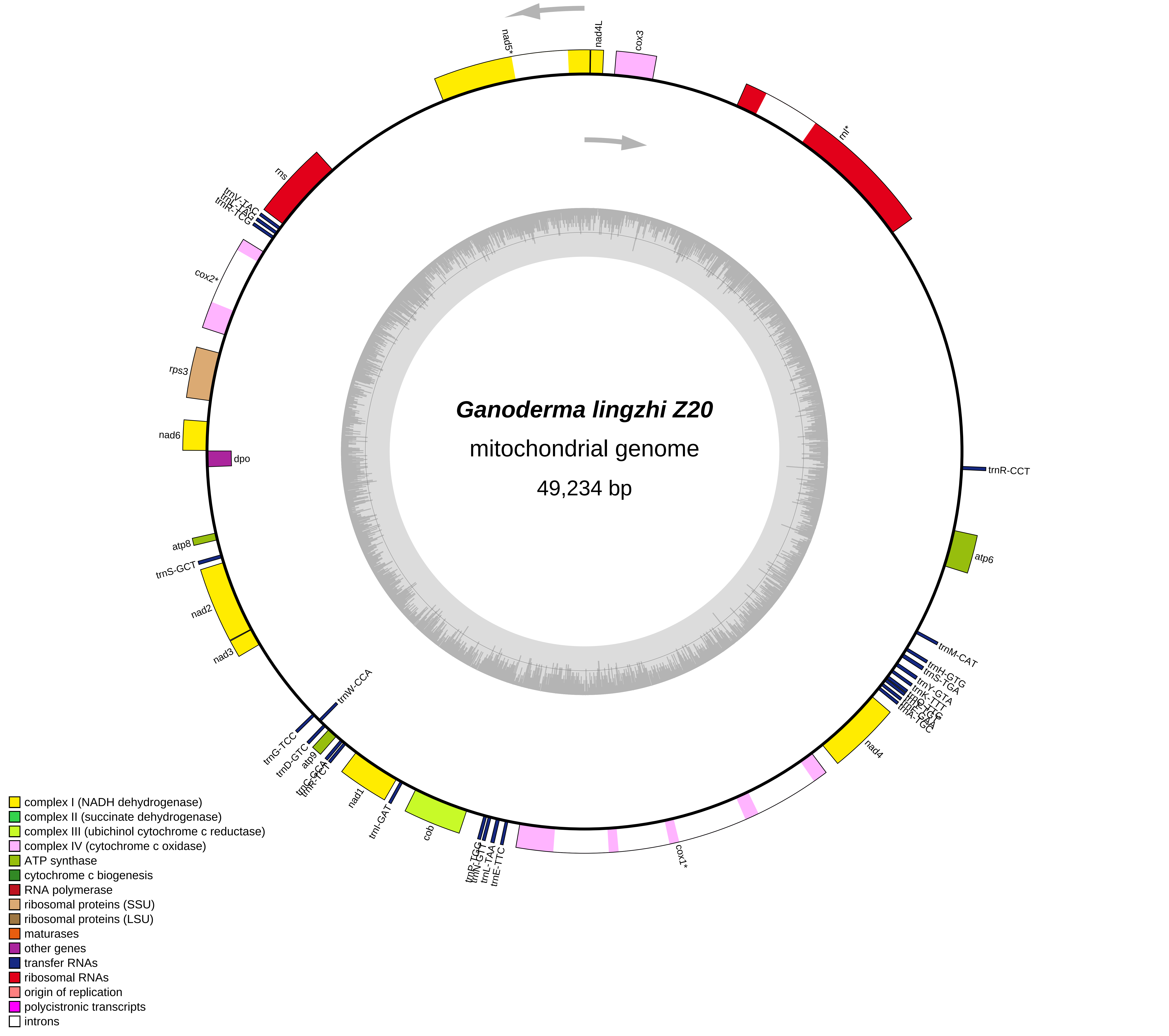
Ganoderma lingzhi Z20
mitochondrial genome
49,234 bp
nad5*
nad4L
cox3
rnl*
rns
trnV-TAC
trnL-TAG
trnR-TCG
cox2*
rps3
nad6
dpo
trnR-CCT
atp6
atp8
trnS-GCT
nad2
nad3
trnM-CAT
trnH-GTG
trnS-TGA
trnY-GTA
trnK-TTT
trnA-TGC
nad4
trnW-CCA
trnG-TCC
trnD-GTC
atp9
nad1
trnI-GAT
cob
trnP-TGG
trnL-TAA
trnE-TTC
cox1*
complex I (NADH dehydrogenase)
complex II (succinate dehydrogenase)
complex III (ubichinol cytochrome c reductase)
complex IV (cytochrome c oxidase)
ATP synthase
cytochrome c biogenesis
RNA polymerase
ribosomal proteins (SSU)
ribosomal proteins (LSU)
maturases
other genes
transfer RNAs
ribosomal RNAs
origin of replication
polycistronic transcripts
introns

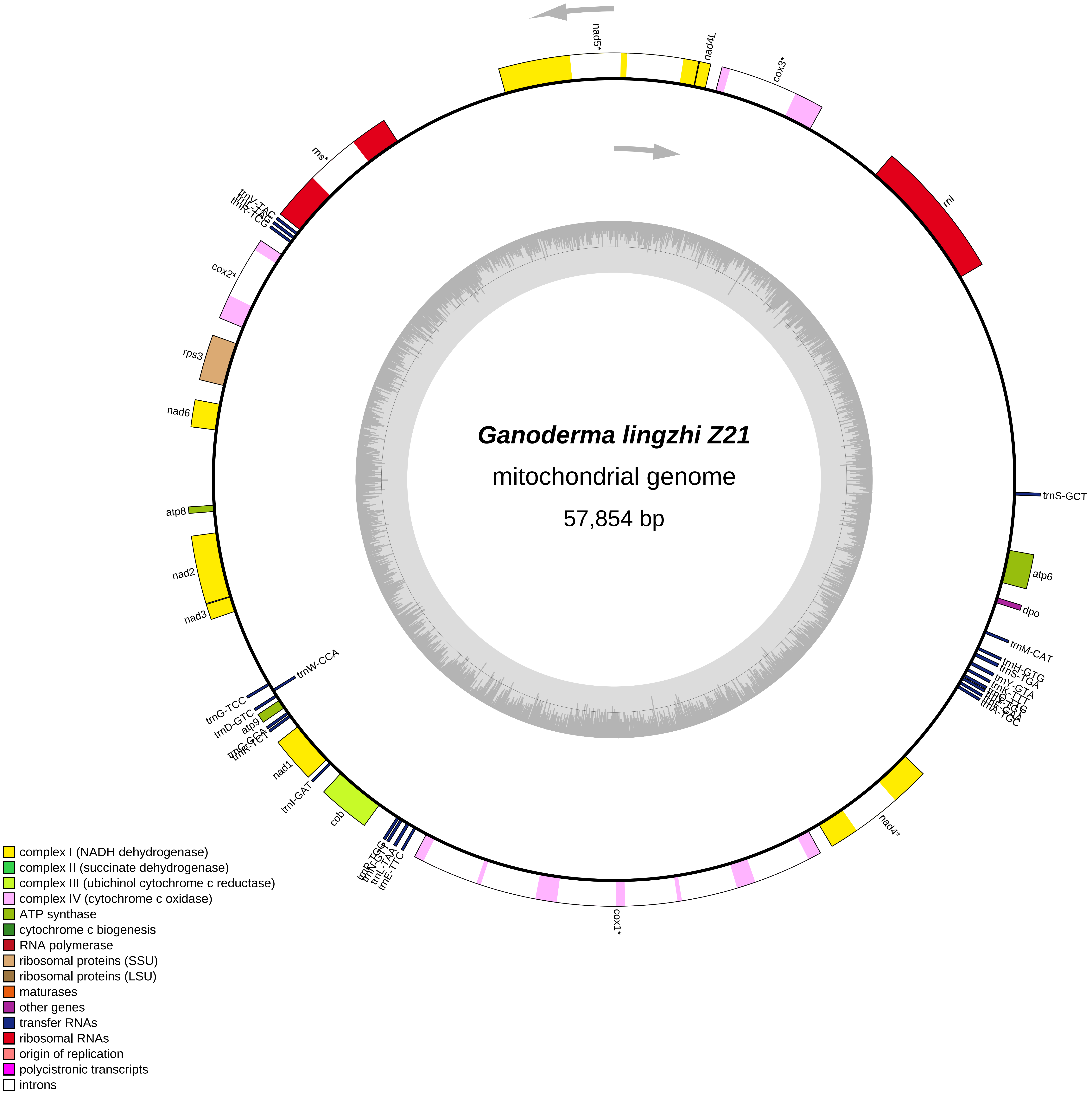
Ganoderma lingzhi Z21
mitochondrial genome
57,854 bp
nad5*
nad4L
cox3*
rnl
trnS-GCT
atp6
dpo
trnM-CAT
trnH-GTG
trnS-TGA
trnY-GTA
nad4*
cox1*
trnE-TTC
trnL-TAA
cob
trnI-GAT
nad1
atp9
trnD-GTC
trnG-TCC
trnW-CCA
nad3
nad2
atp8
nad6
rps3
cox2*
trnR-TCG
rns*
trnV-TAC
complex I (NADH dehydrogenase)
complex II (succinate dehydrogenase)
complex III (ubichinol cytochrome c reductase)
complex IV (cytochrome c oxidase)
ATP synthase
cytochrome c biogenesis
RNA polymerase
ribosomal proteins (SSU)
ribosomal proteins (LSU)
maturases
other genes
transfer RNAs
ribosomal RNAs
origin of replication
polycistronic transcripts
introns

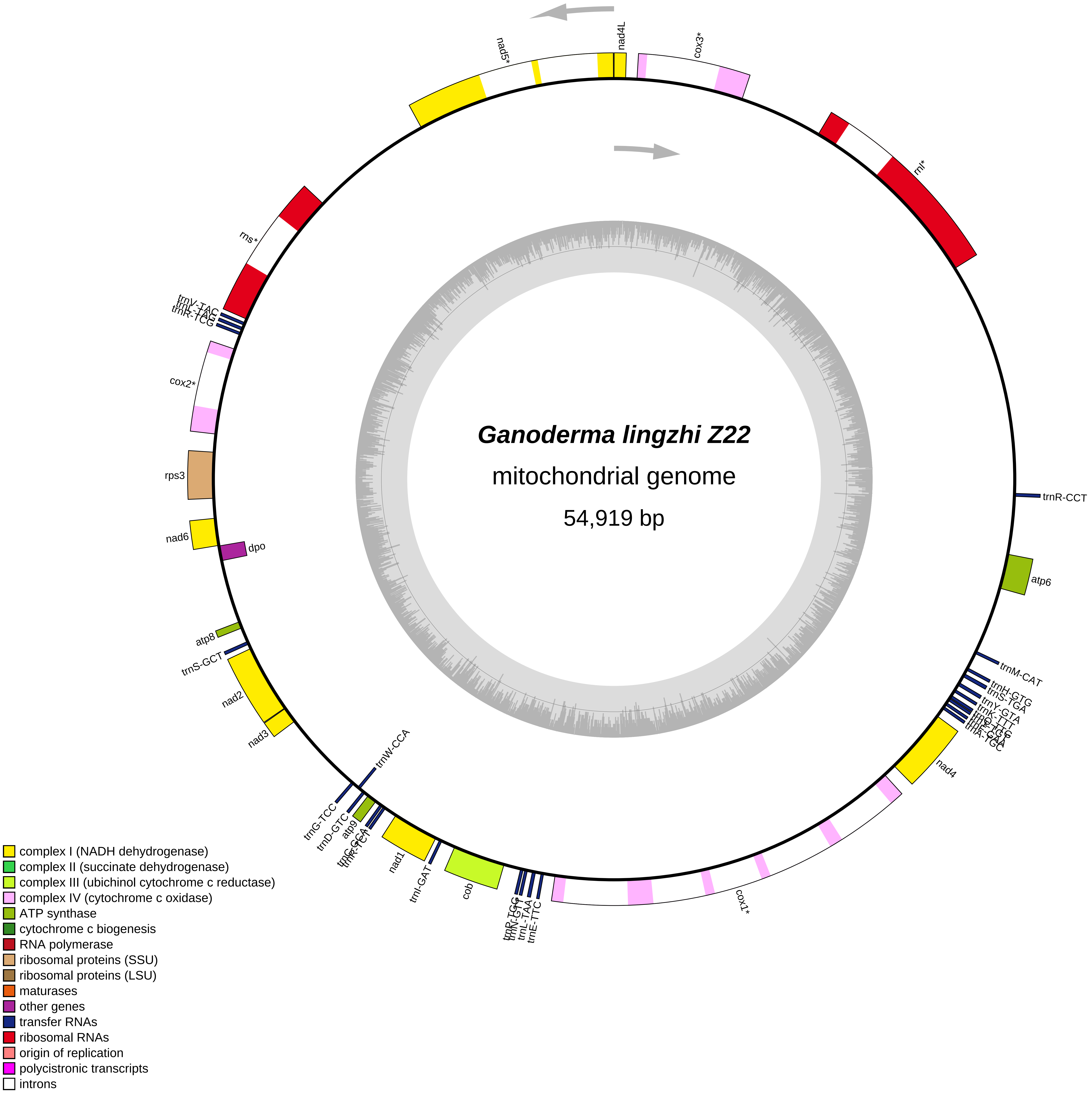

Ganoderma lingzhi Z22
mitochondrial genome
54,919 bp
nad5*
nad4L
cox3*
rnl*
trnR-CCT
atp6
trnM-CAT
trnH-GTG
trnS-TGA
trnY-GTA
nad4
cox1*
trnE-TTC
trnL-TAA
cob
trnI-GAT
nad1
atp9
trnD-GTC
trnG-TCC
trnW-CCA
nad3
nad2
trnS-GCT
atp8
dpo
nad6
rps3
cox2*
trnV-TAC
trnR-TCG
rns*
complex I (NADH dehydrogenase)
complex II (succinate dehydrogenase)
complex III (ubichinol cytochrome c reductase)
complex IV (cytochrome c oxidase)
ATP synthase
cytochrome c biogenesis
RNA polymerase
ribosomal proteins (SSU)
ribosomal proteins (LSU)
maturases
other genes
transfer RNAs
ribosomal RNAs
origin of replication
polycistronic transcripts
introns

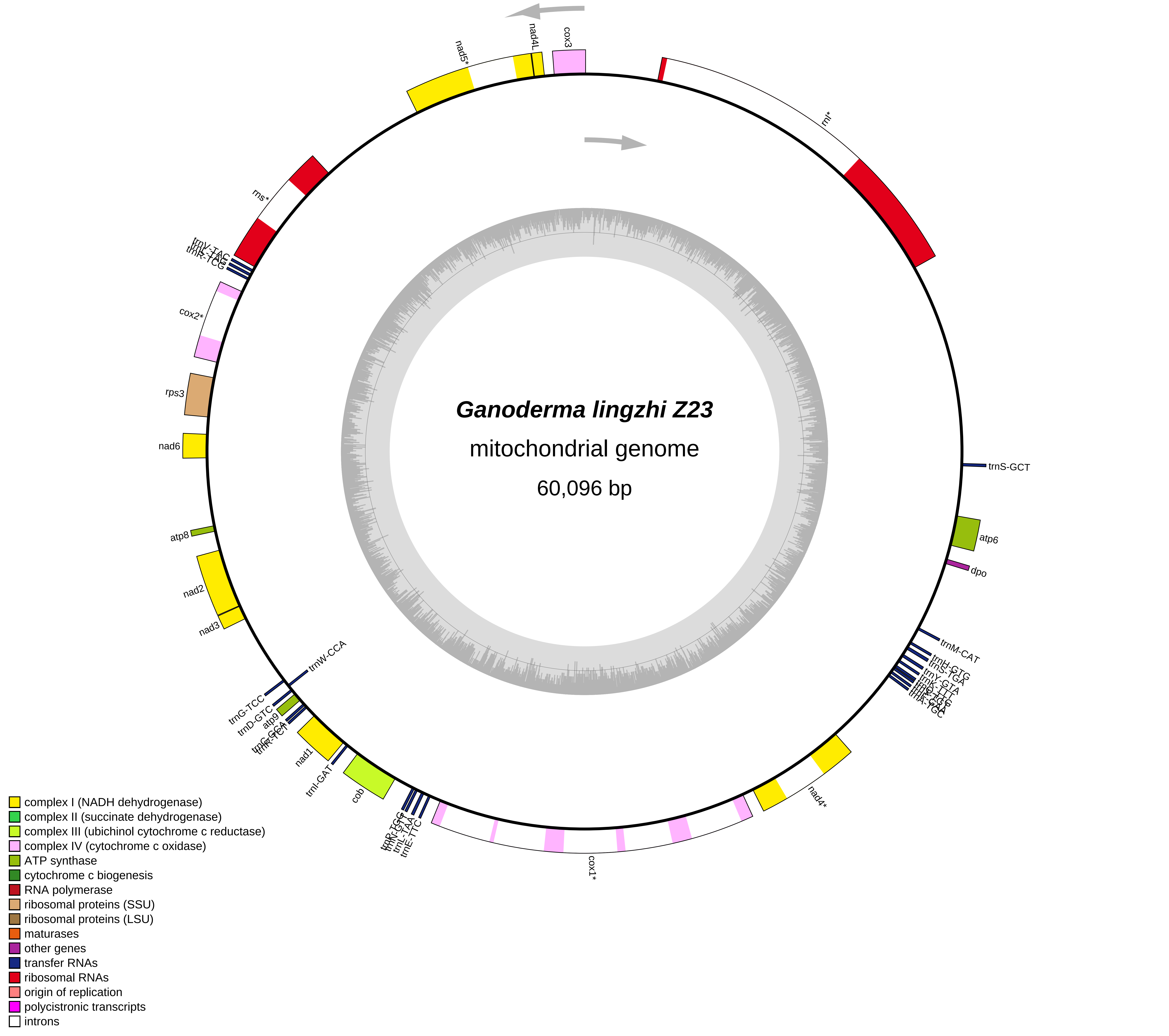

Ganoderma lingzhi Z23
mitochondrial genome
60,096 bp
nad5*
nad4L
cox3
rnl*
rns*
trnV-TAC
trnR-TCG
cox2*
rps3
nad6
atp8
nad2
nad3
trnW-CCA
trnG-TCC
trnD-GTC
atp9
trnC-GCA
nad1
trnI-GAT
cob
trnL-TAA
trnE-TTC
cox1*
nad4*
trnM-CAT
trnH-GTG
trnS-TGA
trnY-GTA
dpo
atp6
trnS-GCT
complex I (NADH dehydrogenase)
complex II (succinate dehydrogenase)
complex III (ubichinol cytochrome c reductase)
complex IV (cytochrome c oxidase)
ATP synthase
cytochrome c biogenesis
RNA polymerase
ribosomal proteins (SSU)
ribosomal proteins (LSU)
maturases
other genes
transfer RNAs
ribosomal RNAs
origin of replication
polycistronic transcripts
introns

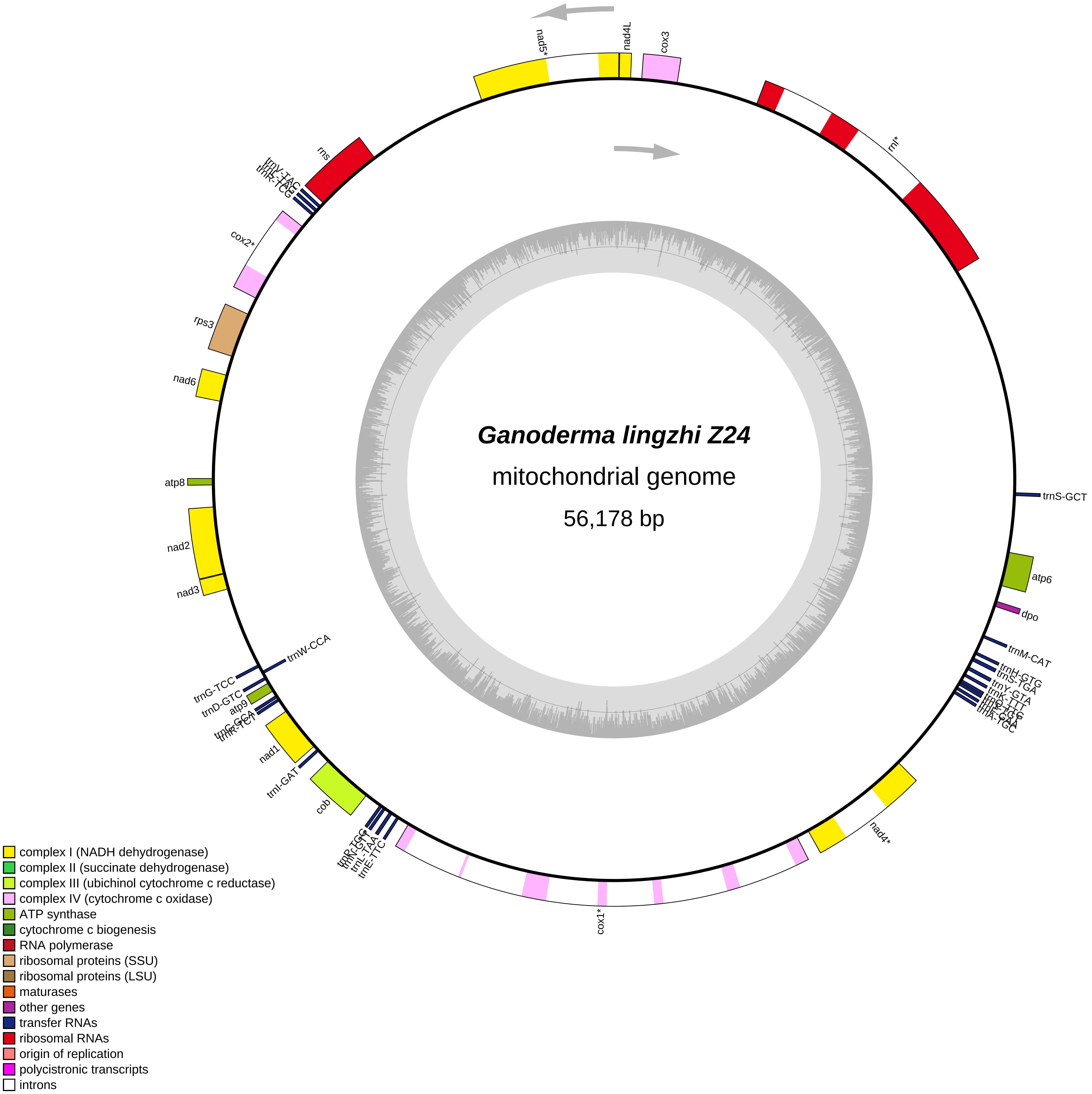

Ganoderma lingzhi Z24
mitochondrial genome
56,178 bp
nad5*
nad4L
cox3
rnl*
rns
cox2*
rps3
nad6
atp8
nad2
nad3
trnW-CCA
trnG-TCC
trnD-GTC
atp9
nad1
trnI-GAT
cob
cox1*
nad4*
trnS-GCT
atp6
dpo
trnM-CAT
trnH-GTG
trnS-TGA
trnY-GTA
trnK-TTT
trnE-TTC
trnL-TAA
complex I (NADH dehydrogenase)
complex II (succinate dehydrogenase)
complex III (ubichinol cytochrome c reductase)
complex IV (cytochrome c oxidase)
ATP synthase
cytochrome c biogenesis
RNA polymerase
ribosomal proteins (SSU)
ribosomal proteins (LSU)
maturases
other genes
transfer RNAs
ribosomal RNAs
origin of replication
polycistronic transcripts
introns

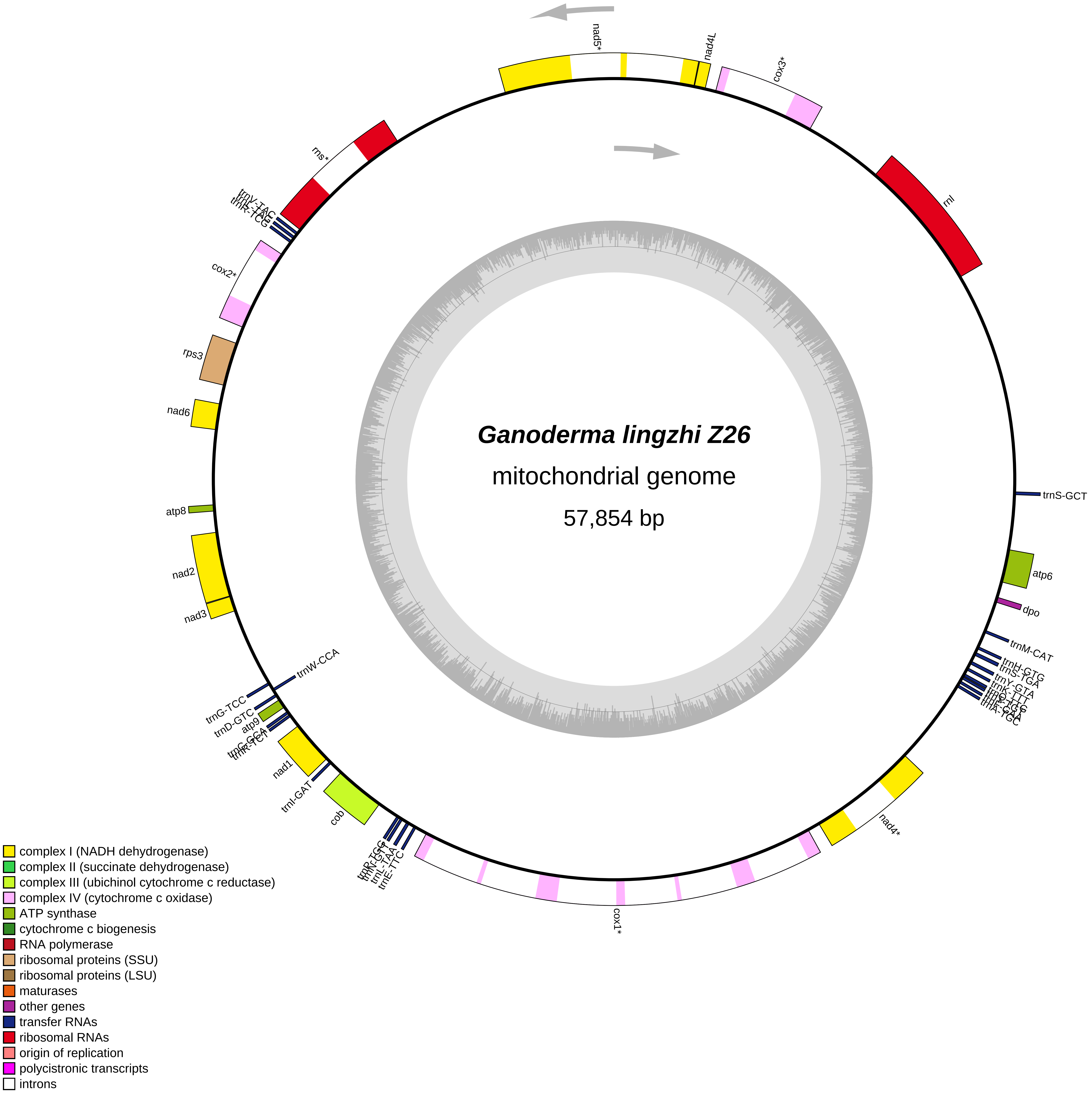

Ganoderma lingzhi Z26
mitochondrial genome
57,854 bp
nad5*
nad4L
cox3*
rnl
trnS-GCT
atp6
dpo
trnM-CAT
trnH-GTG
trnS-TGA
trnY-GTA
nad4*
cox1*
trnE-TTC
trnL-TAA
cob
trnI-GAT
nad1
trnR-TCT
atp9
trnD-GTC
trnG-TCC
trnW-CCA
nad3
nad2
atp8
nad6
rps3
cox2*
trnR-TCG
trnV-TAC
rns*
complex I (NADH dehydrogenase)
complex II (succinate dehydrogenase)
complex III (ubichinol cytochrome c reductase)
complex IV (cytochrome c oxidase)
ATP synthase
cytochrome c biogenesis
RNA polymerase
ribosomal proteins (SSU)
ribosomal proteins (LSU)
maturases
other genes
transfer RNAs
ribosomal RNAs
origin of replication
polycistronic transcripts
introns

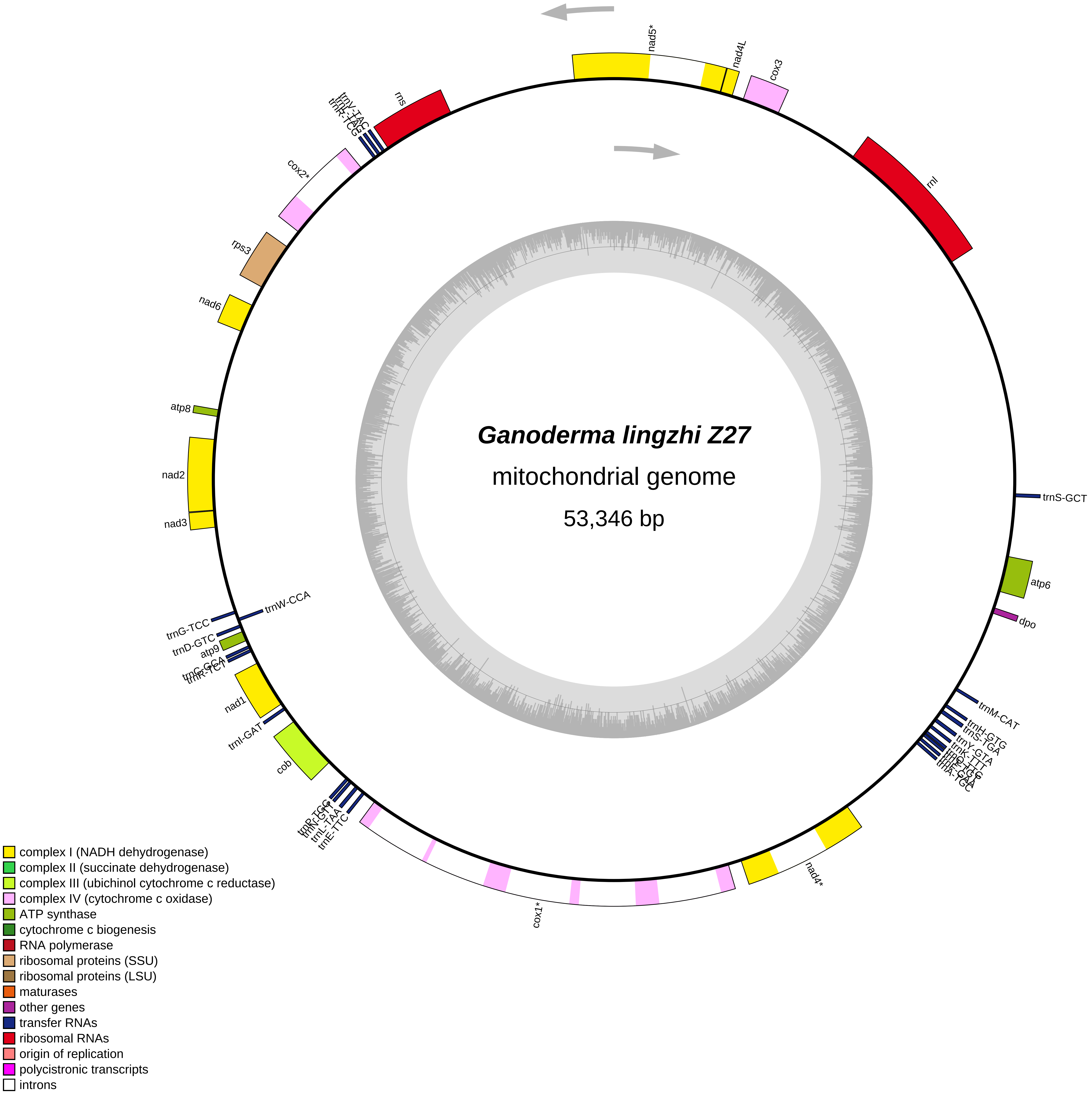
Ganoderma lingzhi Z27
mitochondrial genome
53,346 bp
nad5*
nad4L
cox3
rnl
trnS-GCT
atp6
dpo
trnM-CAT
trnH-GTG
trnS-TGA
trnY-GTA
nad4*
cox1*
trnE-TTC
trnL-TAA
cob
trnI-GAT
nad1
atp9
trnD-GTC
trnG-TCC
trnW-CCA
nad3
nad2
atp8
nad6
rps3
cox2*
rns
trnV-TAC
complex I (NADH dehydrogenase)
complex II (succinate dehydrogenase)
complex III (ubichinol cytochrome c reductase)
complex IV (cytochrome c oxidase)
ATP synthase
cytochrome c biogenesis
RNA polymerase
ribosomal proteins (SSU)
ribosomal proteins (LSU)
maturases
other genes
transfer RNAs
ribosomal RNAs
origin of replication
polycistronic transcripts
introns

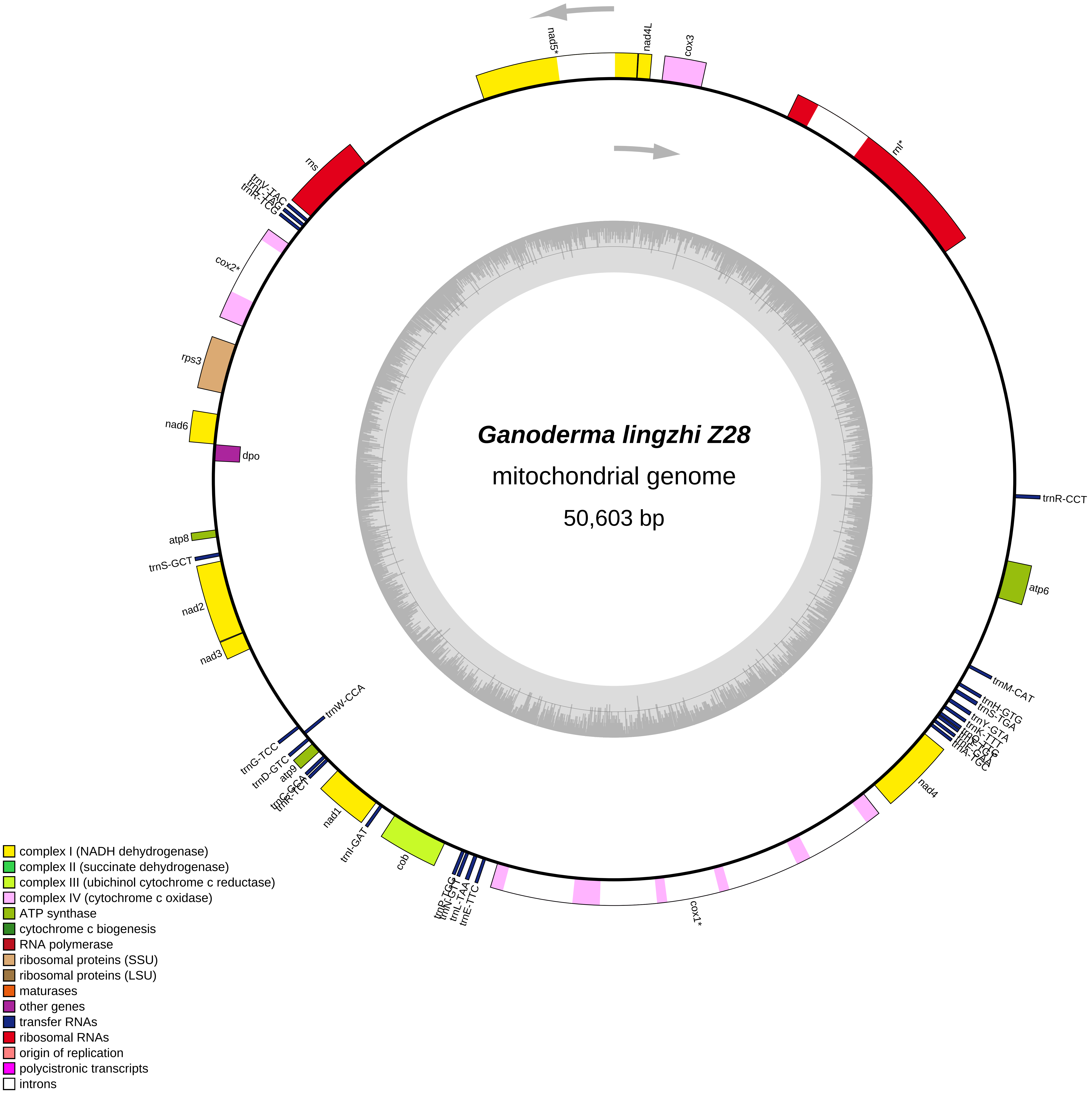
Ganoderma lingzhi Z28
mitochondrial genome
50,603 bp
nad5*
nad4L
cox3
rnl*
trnR-CCT
atp6
trnM-CAT
trnH-GTG
trnS-TGA
trnY-GTA
trnK-TTT
trnA-TGC
nad4
cox1*
trnE-TTC
trnL-TAA
cob
trnI-GAT
nad1
trnR-TCT
atp9
trnD-GTC
trnG-TCC
trnW-CCA
nad3
nad2
trnS-GCT
atp8
dpo
nad6
rps3
cox2*
trnR-TCG
trnV-TAC
rns
complex I (NADH dehydrogenase)
complex II (succinate dehydrogenase)
complex III (ubichinol cytochrome c reductase)
complex IV (cytochrome c oxidase)
ATP synthase
cytochrome c biogenesis
RNA polymerase
ribosomal proteins (SSU)
ribosomal proteins (LSU)
maturases
other genes
transfer RNAs
ribosomal RNAs
origin of replication
polycistronic transcripts
introns

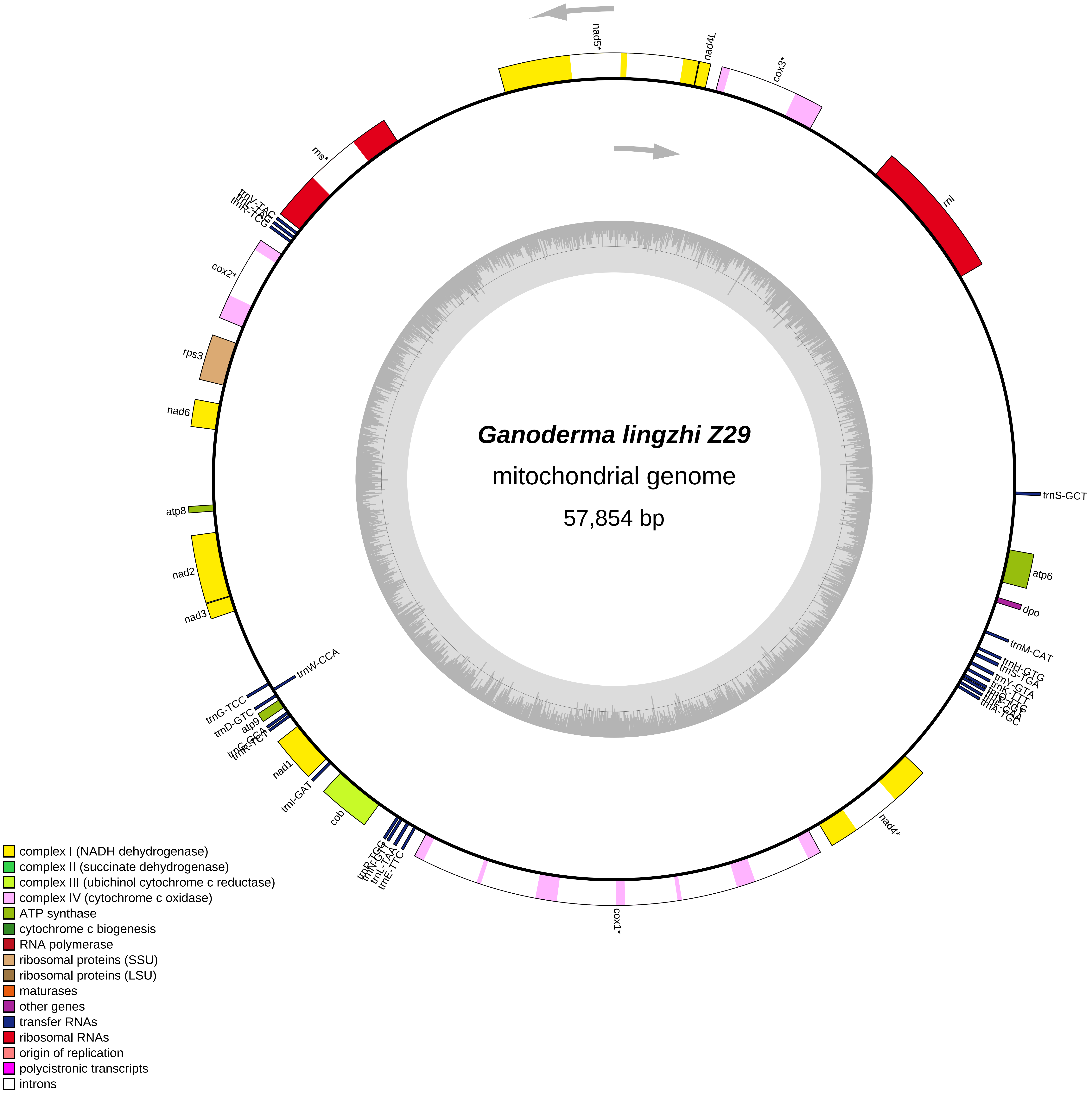

Ganoderma lingzhi Z29
mitochondrial genome
57,854 bp
nad5*
nad4L
cox3*
rnl
rns*
cox2*
rps3
nad6
atp8
nad2
nad3
trnW-CCA
trnG-TCC
trnD-GTC
atp9
nad1
trnI-GAT
cob
cox1*
nad4*
trnS-GCT
atp6
dpo
trnM-CAT
trnH-GTG
trnS-TGA
trnY-GTA
trnA-TGC
trnE-TTC
trnL-TAA
complex I (NADH dehydrogenase)
complex II (succinate dehydrogenase)
complex III (ubichinol cytochrome c reductase)
complex IV (cytochrome c oxidase)
ATP synthase
cytochrome c biogenesis
RNA polymerase
ribosomal proteins (SSU)
ribosomal proteins (LSU)
maturases
other genes
transfer RNAs
ribosomal RNAs
origin of replication
polycistronic transcripts
introns

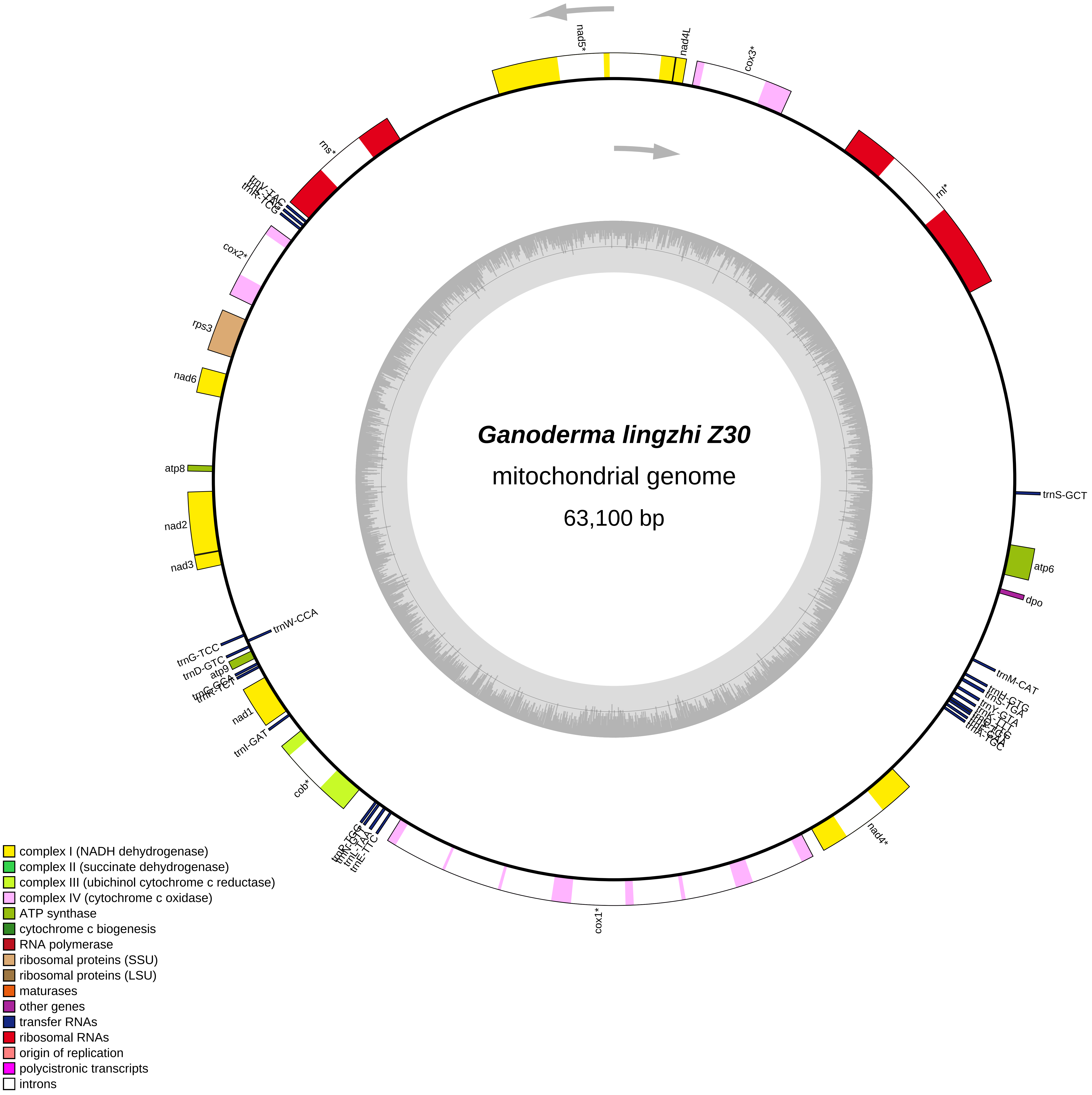

Ganoderma lingzhi Z30
mitochondrial genome
63,100 bp
nad5*
nad4L
cox3*
rnl*
trnS-GCT
atp6
dpo
trnM-CAT
trnH-GTG
trnS-TGA
trnY-GTA
nad4*
cox1*
trnE-TTC
trnL-TAA
trnP-TGG
cob*
trnI-GAT
nad1
trnR-TCT
trnC-GCA
atp9
trnD-GTC
trnG-TCC
trnW-CCA
nad3
nad2
atp8
nad6
rps3
cox2*
trnR-TCG
trnV-TAC
rns*
complex I (NADH dehydrogenase)
complex II (succinate dehydrogenase)
complex III (ubichinol cytochrome c reductase)
complex IV (cytochrome c oxidase)
ATP synthase
cytochrome c biogenesis
RNA polymerase
ribosomal proteins (SSU)
ribosomal proteins (LSU)
maturases
other genes
transfer RNAs
ribosomal RNAs
origin of replication
polycistronic transcripts
introns

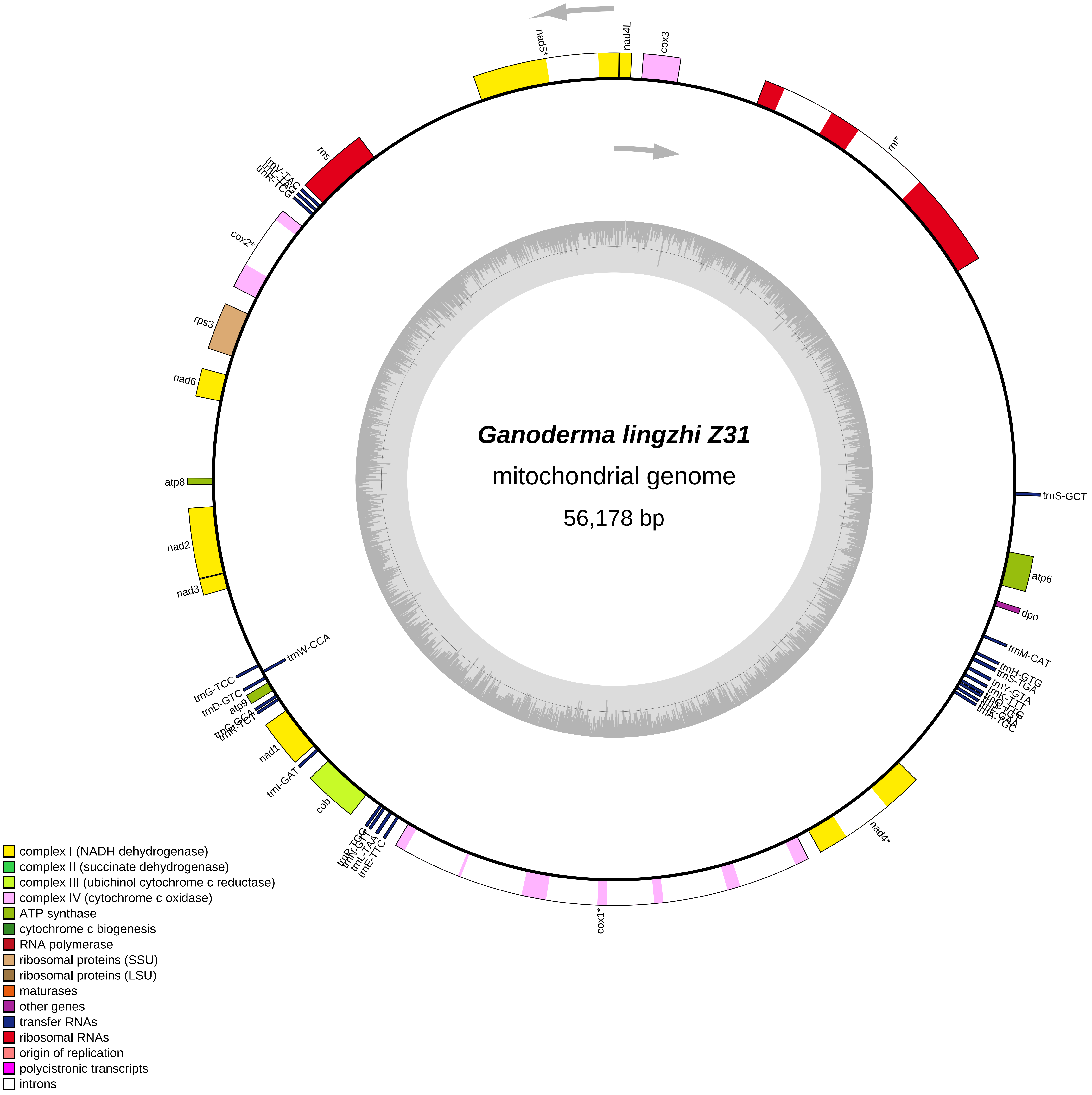

Ganoderma lingzhi Z31
mitochondrial genome
56,178 bp
nad5*
nad4L
cox3
rnl*
rns
trnV-TAC
cox2*
rps3
nad6
atp8
nad2
nad3
trnW-CCA
trnG-TCC
trnD-GTC
atp9
trnC-GCA
nad1
trnI-GAT
cob
trnL-TAA
trnE-TTC
cox1*
nad4*
trnS-GCT
atp6
dpo
trnM-CAT
trnH-GTG
trnS-TGA
trnY-GTA
trnA-TGC
complex I (NADH dehydrogenase)
complex II (succinate dehydrogenase)
complex III (ubichinol cytochrome c reductase)
complex IV (cytochrome c oxidase)
ATP synthase
cytochrome c biogenesis
RNA polymerase
ribosomal proteins (SSU)
ribosomal proteins (LSU)
maturases
other genes
transfer RNAs
ribosomal RNAs
origin of replication
polycistronic transcripts
introns

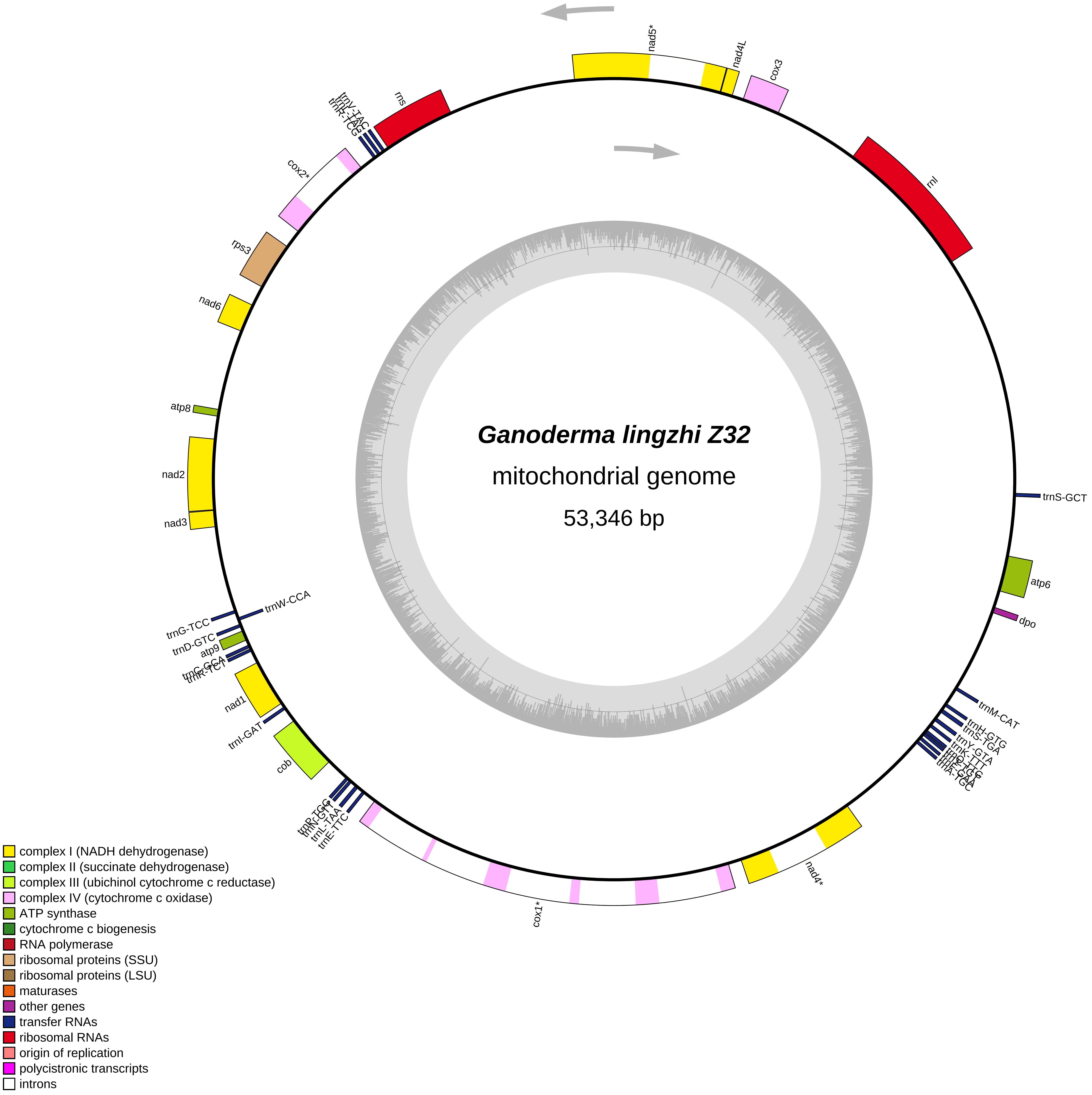

Ganoderma lingzhi Z32
mitochondrial genome
53,346 bp
nad5*
nad4L
cox3
rnl
trnS-GCT
atp6
dpo
trnM-CAT
trnH-GTG
trnS-TGA
trnY-GTA
nad4*
cox1*
trnE-TTC
trnL-TAA
cob
trnI-GAT
nad1
trnR-TCT
atp9
trnD-GTC
trnG-TCC
trnW-CCA
nad3
nad2
atp8
nad6
rps3
cox2*
rns
trnV-TAC
complex I (NADH dehydrogenase)
complex II (succinate dehydrogenase)
complex III (ubichinol cytochrome c reductase)
complex IV (cytochrome c oxidase)
ATP synthase
cytochrome c biogenesis
RNA polymerase
ribosomal proteins (SSU)
ribosomal proteins (LSU)
maturases
other genes
transfer RNAs
ribosomal RNAs
origin of replication
polycistronic transcripts
introns

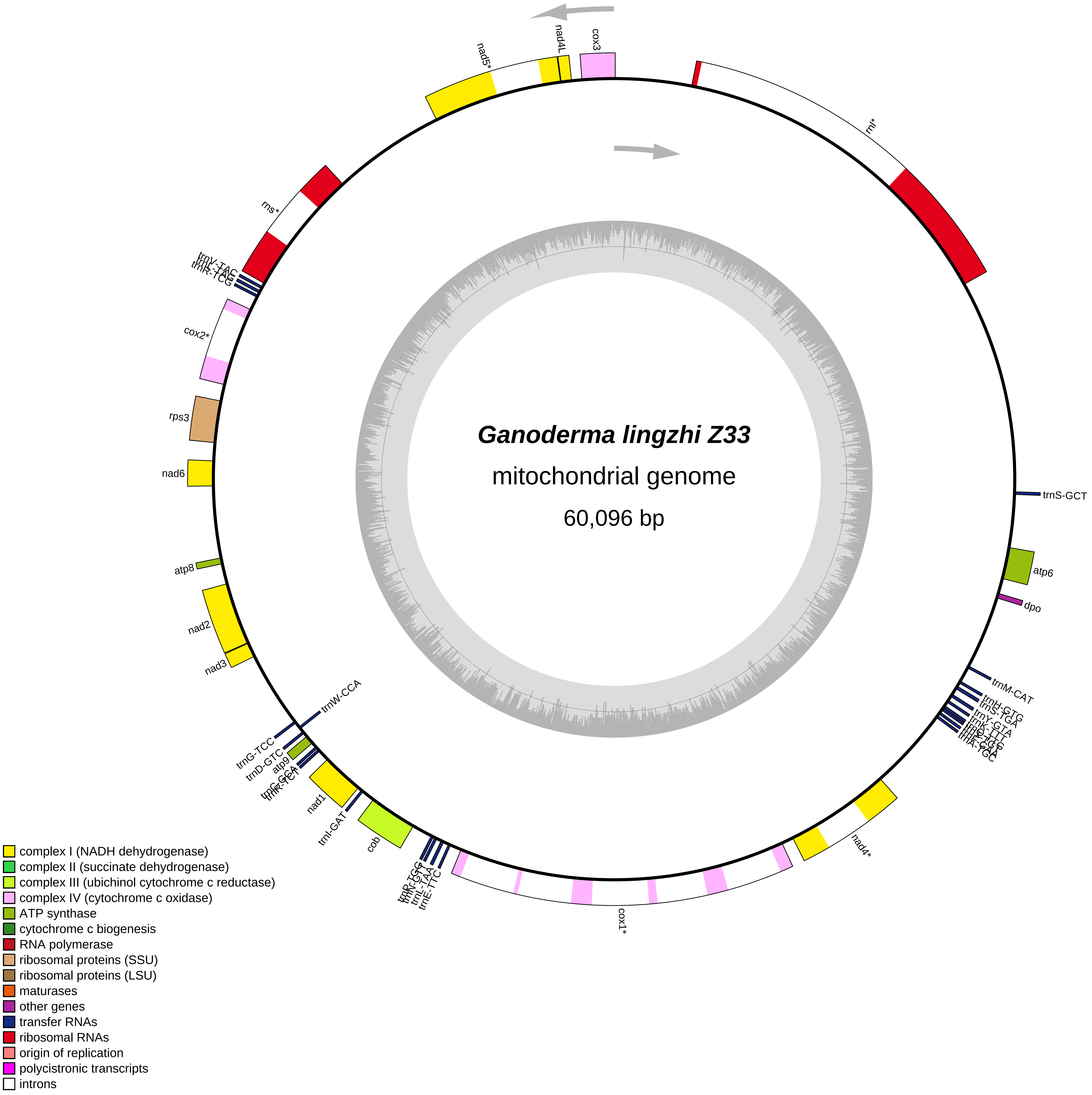
Ganoderma lingzhi Z33
mitochondrial genome
60,096 bp
nad5*
nad4L
cox3
rnl*
rns*
trnV-TAC
trnR-TCG
cox2*
rps3
nad6
trnS-GCT
atp6
dpo
atp8
nad2
nad3
trnM-CAT
trnH-GTG
trnS-TGA
trnY-GTA
trnW-CCA
trnG-TCC
trnD-GTC
atp9
trnC-GCA
nad1
trnI-GAT
cob
trnP-TGG
trnL-TAA
trnE-TTC
cox1*
nad4*
complex I (NADH dehydrogenase)
complex II (succinate dehydrogenase)
complex III (ubichinol cytochrome c reductase)
complex IV (cytochrome c oxidase)
ATP synthase
cytochrome c biogenesis
RNA polymerase
ribosomal proteins (SSU)
ribosomal proteins (LSU)
maturases
other genes
transfer RNAs
ribosomal RNAs
origin of replication
polycistronic transcripts
introns

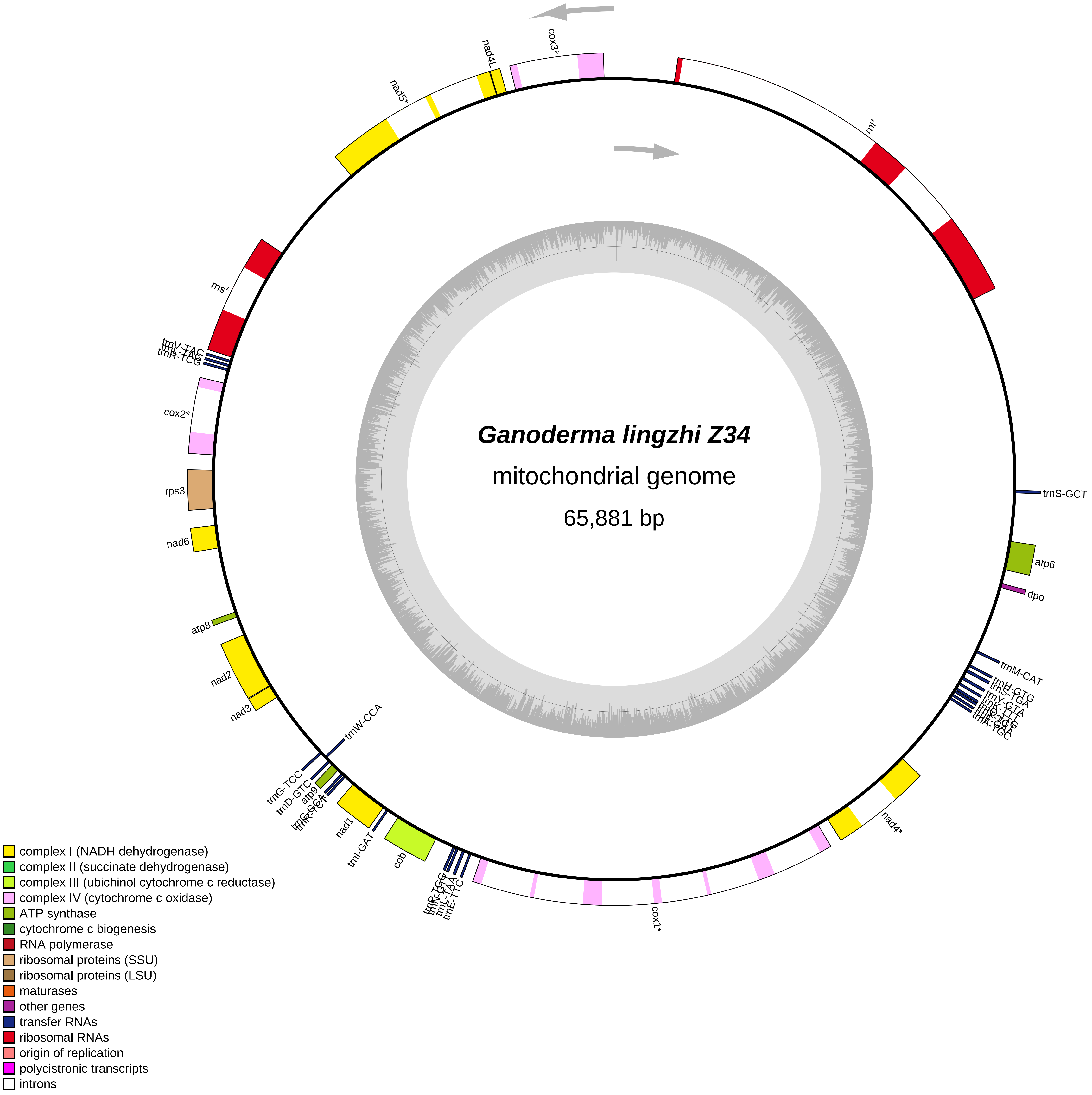

Ganoderma lingzhi Z34
mitochondrial genome
65,881 bp
cox3*
nad4L
nad5*
rnl*
rns*
trnV-TAC
trnR-TCG
cox2*
rps3
nad6
atp8
nad2
nad3
trnW-CCA
trnG-TCC
trnD-GTC
atp9
nad1
trnI-GAT
cob
trnE-TTC
cox1*
nad4*
trnM-CAT
trnH-GTG
trnS-TGA
trnY-GTA
trnA-TGC
dpo
atp6
trnS-GCT
complex I (NADH dehydrogenase)
complex II (succinate dehydrogenase)
complex III (ubichinol cytochrome c reductase)
complex IV (cytochrome c oxidase)
ATP synthase
cytochrome c biogenesis
RNA polymerase
ribosomal proteins (SSU)
ribosomal proteins (LSU)
maturases
other genes
transfer RNAs
ribosomal RNAs
origin of replication
polycistronic transcripts
introns

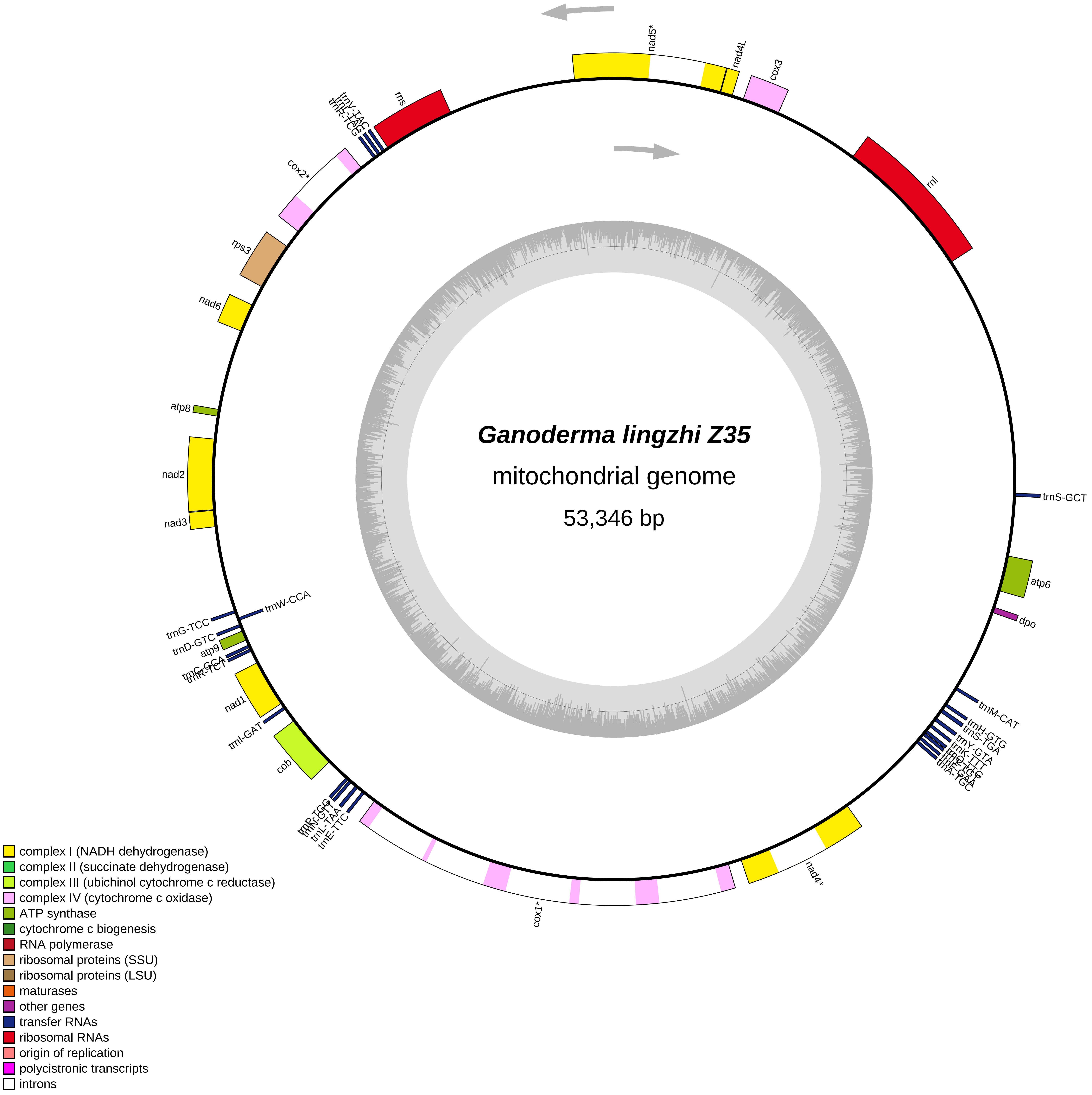

Ganoderma lingzhi Z35
mitochondrial genome
53,346 bp
nad5*
nad4L
cox3
rnl
trnS-GCT
atp6
dpo
trnM-CAT
trnH-GTG
trnS-TGA
trnY-GTA
nad4*
cox1*
trnE-TTC
trnL-TAA
cob
trnI-GAT
nad1
trnR-TCT
atp9
trnD-GTC
trnG-TCC
trnW-CCA
nad3
nad2
atp8
nad6
rps3
cox2*
rns
trnV-TAC
complex I (NADH dehydrogenase)
complex II (succinate dehydrogenase)
complex III (ubichinol cytochrome c reductase)
complex IV (cytochrome c oxidase)
ATP synthase
cytochrome c biogenesis
RNA polymerase
ribosomal proteins (SSU)
ribosomal proteins (LSU)
maturases
other genes
transfer RNAs
ribosomal RNAs
origin of replication
polycistronic transcripts
introns

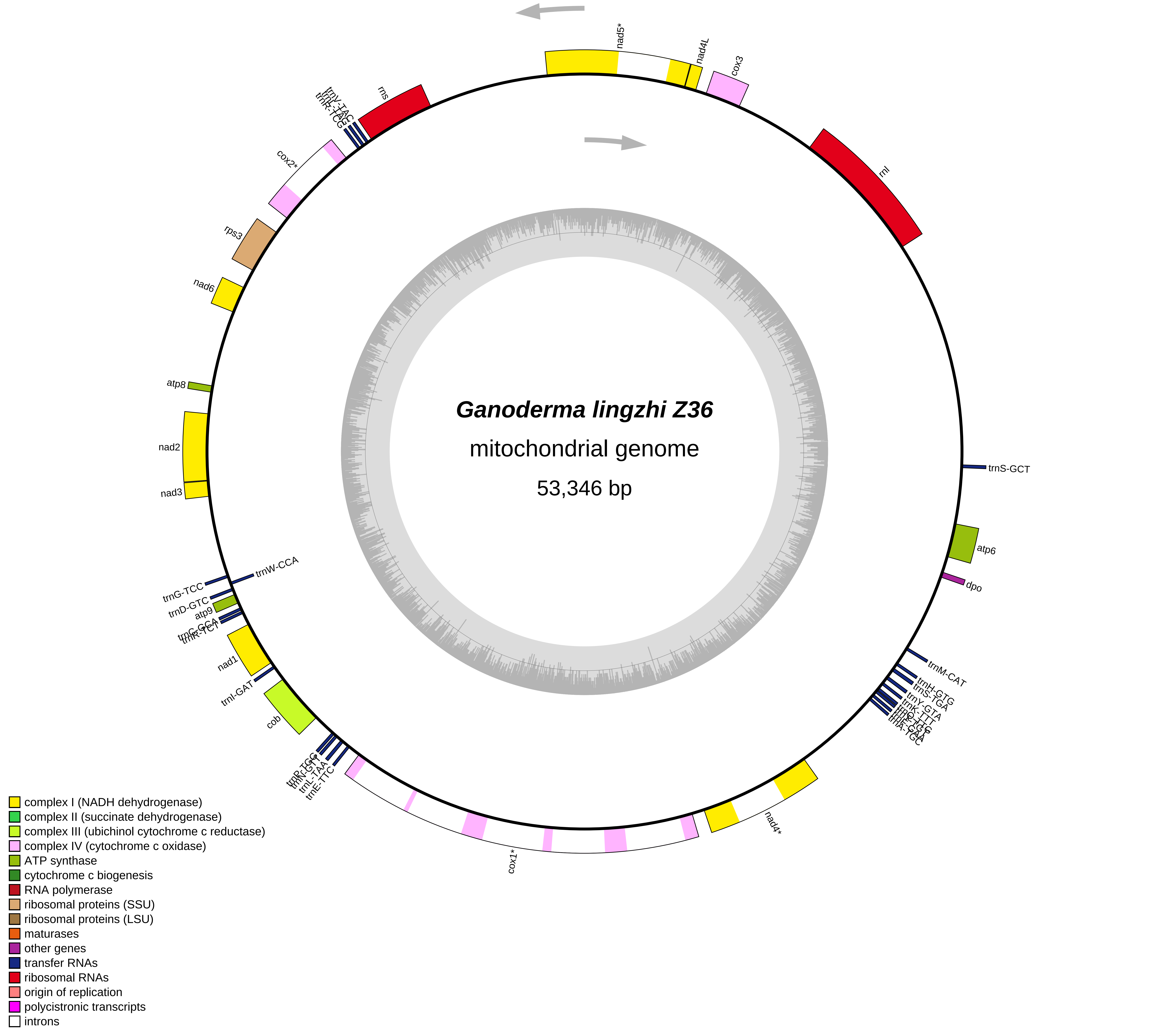

Ganoderma lingzhi Z36
mitochondrial genome
53,346 bp
nad5*
nad4L
cox3
rnl
trnS-GCT
atp6
dpo
trnM-CAT
trnH-GTG
trnS-TGA
trnY-GTA
nad4*
cox1*
trnE-TTC
trnL-TAA
cob
trnI-GAT
nad1
trnR-TCT
atp9
trnD-GTC
trnG-TCC
trnW-CCA
nad3
nad2
atp8
nad6
rps3
cox2*
rns
complex I (NADH dehydrogenase)
complex II (succinate dehydrogenase)
complex III (ubichinol cytochrome c reductase)
complex IV (cytochrome c oxidase)
ATP synthase
cytochrome c biogenesis
RNA polymerase
ribosomal proteins (SSU)
ribosomal proteins (LSU)
maturases
other genes
transfer RNAs
ribosomal RNAs
origin of replication
polycistronic transcripts
introns

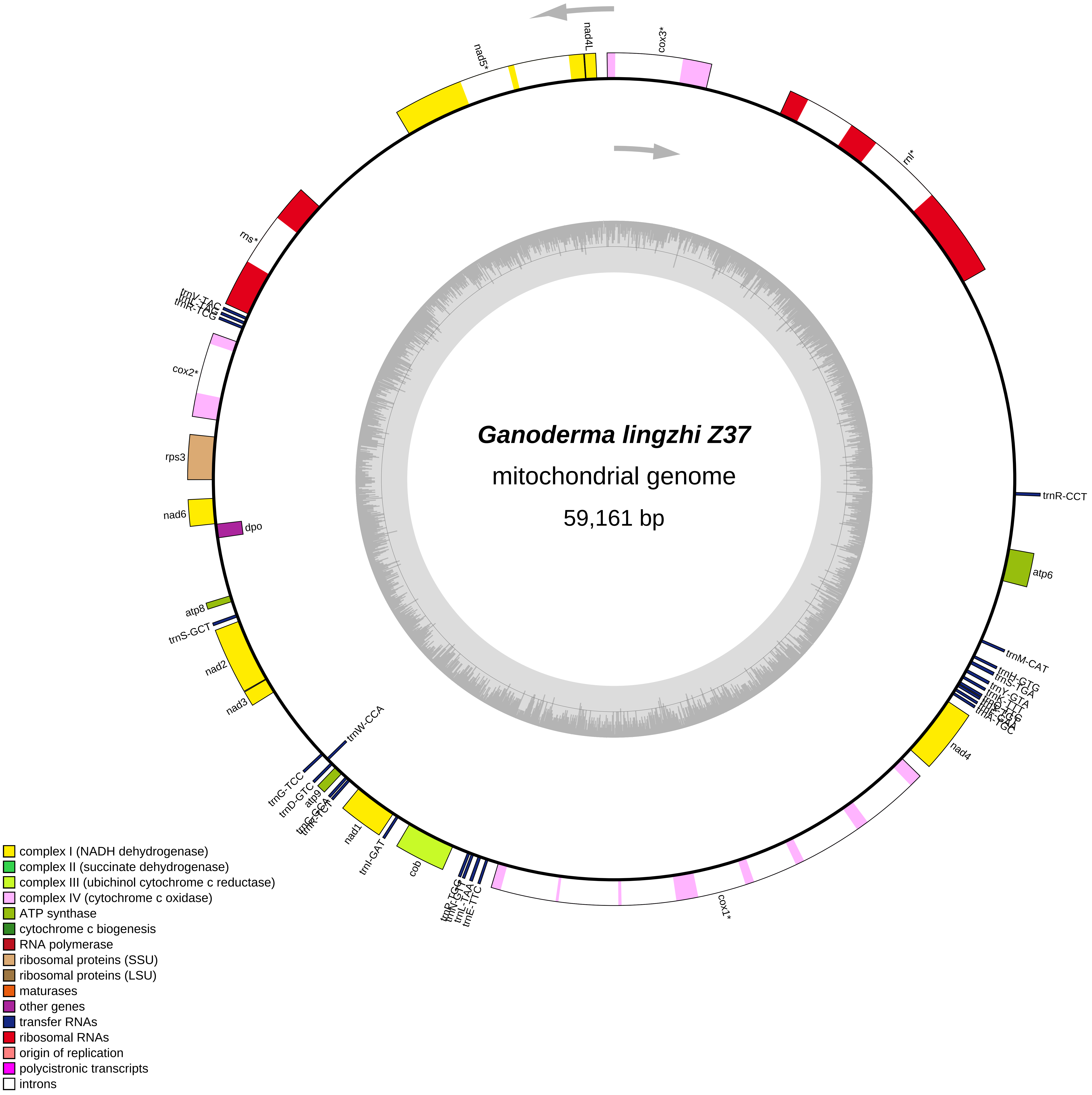
Ganoderma lingzhi Z37
mitochondrial genome
59,161 bp
nad4L
cox3*
nad5*
rnl*
rns*
trnV-TAC
trnR-TCG
cox2*
rps3
nad6
dpo
trnR-CCT
atp6
atp8
trnS-GCT
nad2
nad3
trnW-CCA
trnM-CAT
trnH-GTG
trnS-TGA
trnY-GTA
nad4
trnG-TCC
trnD-GTC
atp9
nad1
trnI-GAT
cob
trnL-TAA
trnE-TTC
cox1*
complex I (NADH dehydrogenase)
complex II (succinate dehydrogenase)
complex III (ubichinol cytochrome c reductase)
complex IV (cytochrome c oxidase)
ATP synthase
cytochrome c biogenesis
RNA polymerase
ribosomal proteins (SSU)
ribosomal proteins (LSU)
maturases
other genes
transfer RNAs
ribosomal RNAs
origin of replication
polycistronic transcripts
introns

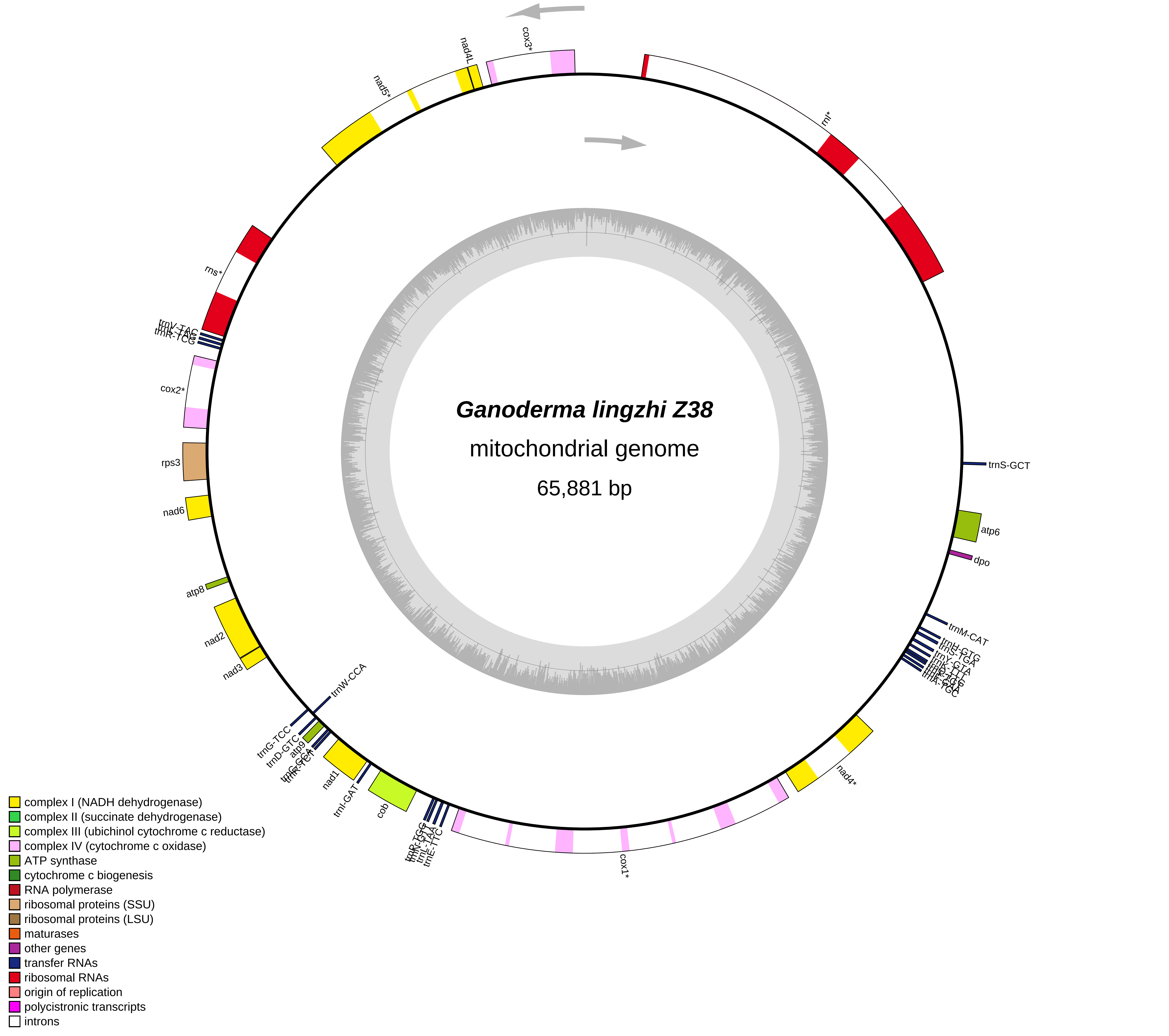
Ganoderma lingzhi Z38
mitochondrial genome
65,881 bp
rnl*
trnS-GCT
atp6
dpo
trnM-CAT
nad4*
cox1*
cob
trnI-GAT
nad1
atp9
trnD-GTC
trnG-TCC
trnW-CCA
nad3
nad2
atp8
nad6
rps3
cox2*
trnR-TCG
trnV-TAC
rns*
nad5*
nad4L
cox3*
complex I (NADH dehydrogenase)
complex II (succinate dehydrogenase)
complex III (ubichinol cytochrome c reductase)
complex IV (cytochrome c oxidase)
ATP synthase
cytochrome c biogenesis
RNA polymerase
ribosomal proteins (SSU)
ribosomal proteins (LSU)
maturases
other genes
transfer RNAs
ribosomal RNAs
origin of replication
polycistronic transcripts
introns

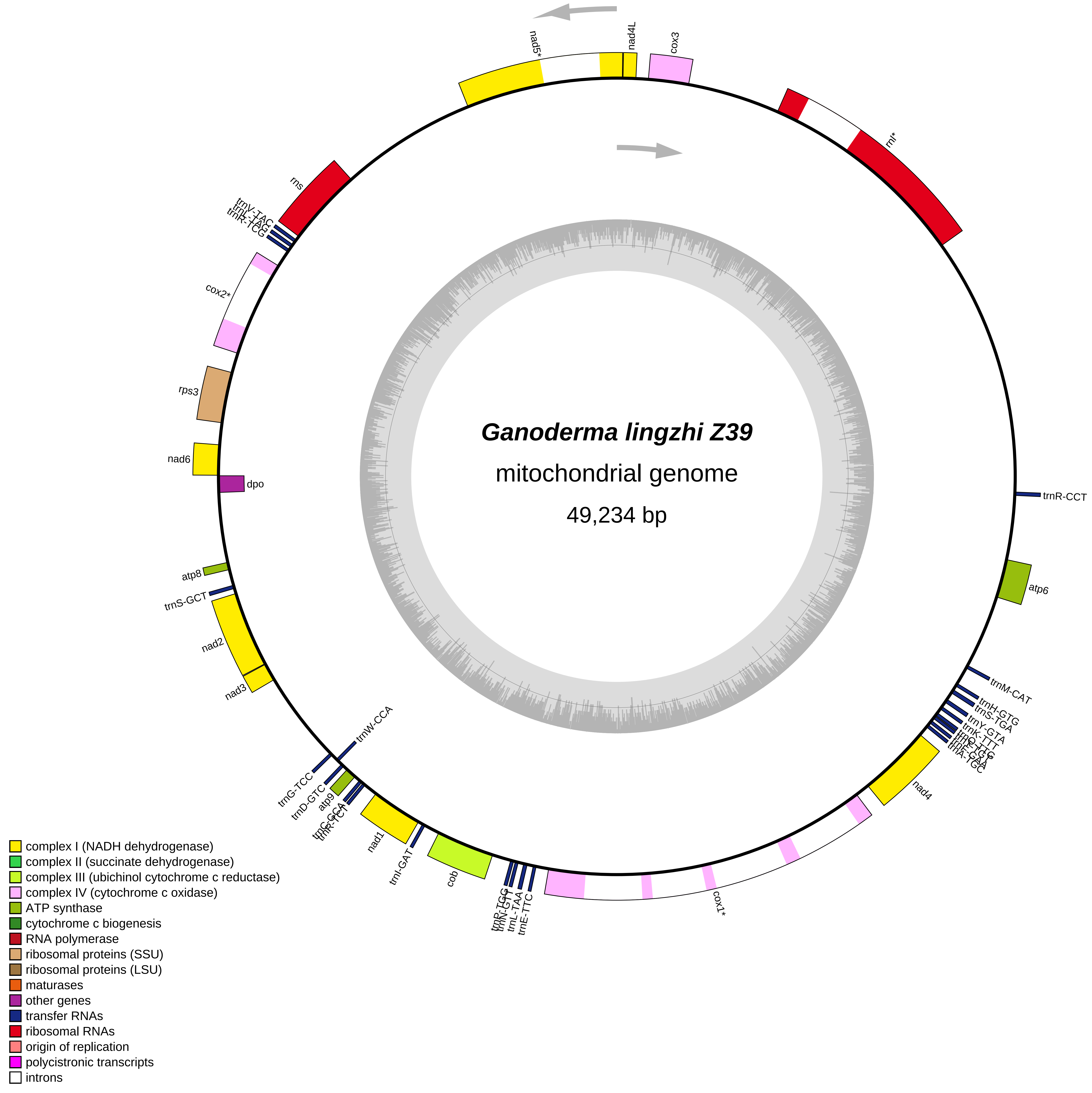

Ganoderma lingzhi Z39
mitochondrial genome
49,234 bp
nad5*
nad4L
cox3
rnl*
rns
trnV-TAC
trnR-TCG
cox2*
rps3
nad6
dpo
trnR-CCT
atp6
atp8
trnS-GCT
nad2
nad3
trnM-CAT
trnH-GTG
trnS-TGA
trnY-GTA
trnK-TTT
trnA-TGC
nad4
trnW-CCA
trnG-TCC
trnD-GTC
atp9
trnC-GCA
nad1
trnI-GAT
cob
trnP-TGG
trnL-TAA
trnE-TTC
cox1*
complex I (NADH dehydrogenase)
complex II (succinate dehydrogenase)
complex III (ubichinol cytochrome c reductase)
complex IV (cytochrome c oxidase)
ATP synthase
cytochrome c biogenesis
RNA polymerase
ribosomal proteins (SSU)
ribosomal proteins (LSU)
maturases
other genes
transfer RNAs
ribosomal RNAs
origin of replication
polycistronic transcripts
introns

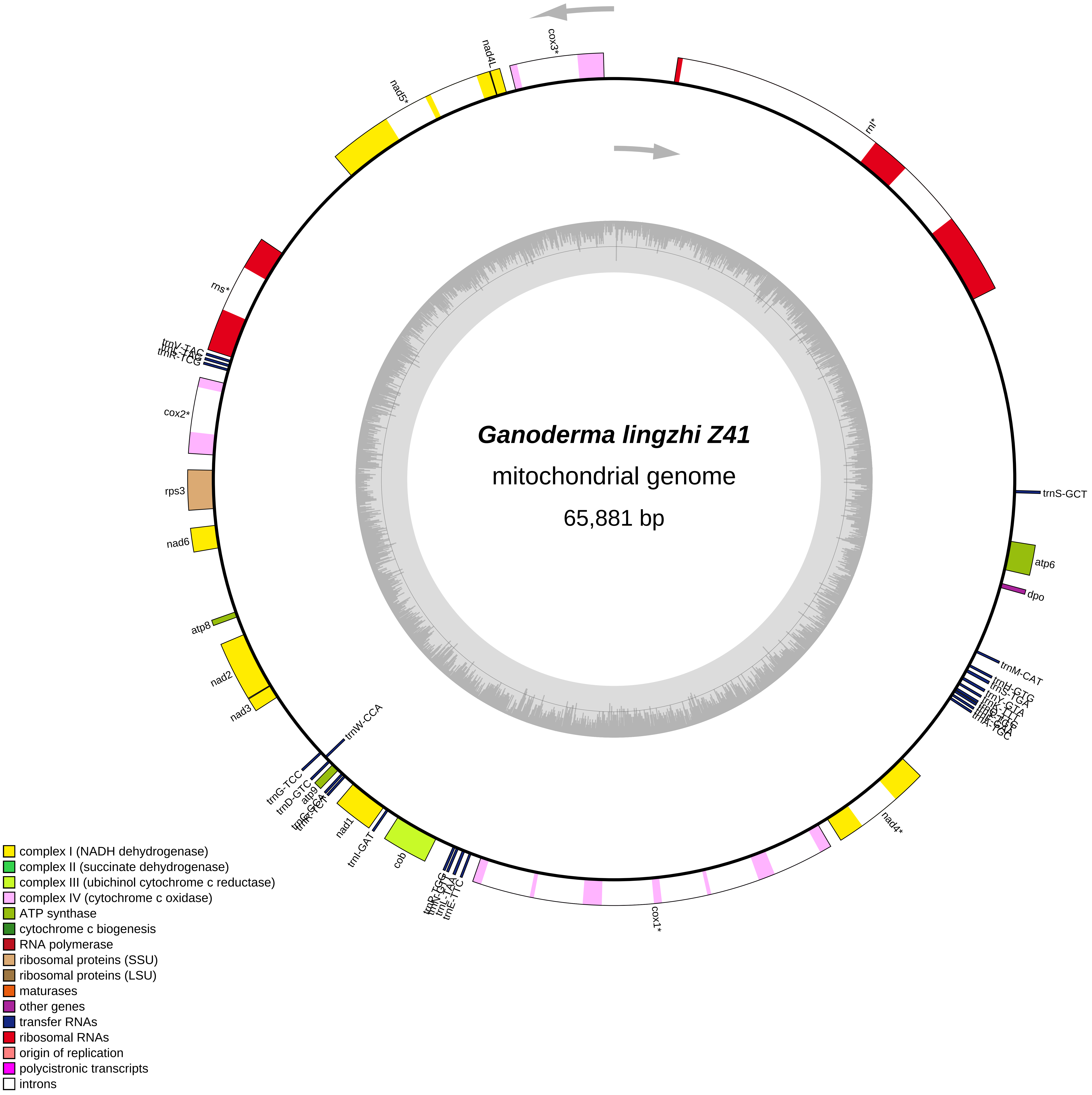

Ganoderma lingzhi Z41
mitochondrial genome
65,881 bp
cox3*
nad4L
nad5*
rnl*
rns*
cox2*
rps3
nad6
atp8
nad2
nad3
trnW-CCA
trnG-TCC
trnD-GTC
atp9
nad1
trnI-GAT
cob
cox1*
nad4*
trnM-CAT
dpo
atp6
trnS-GCT
complex I (NADH dehydrogenase)
complex II (succinate dehydrogenase)
complex III (ubichinol cytochrome c reductase)
complex IV (cytochrome c oxidase)
ATP synthase
cytochrome c biogenesis
RNA polymerase
ribosomal proteins (SSU)
ribosomal proteins (LSU)
maturases
other genes
transfer RNAs
ribosomal RNAs
origin of replication
polycistronic transcripts
introns

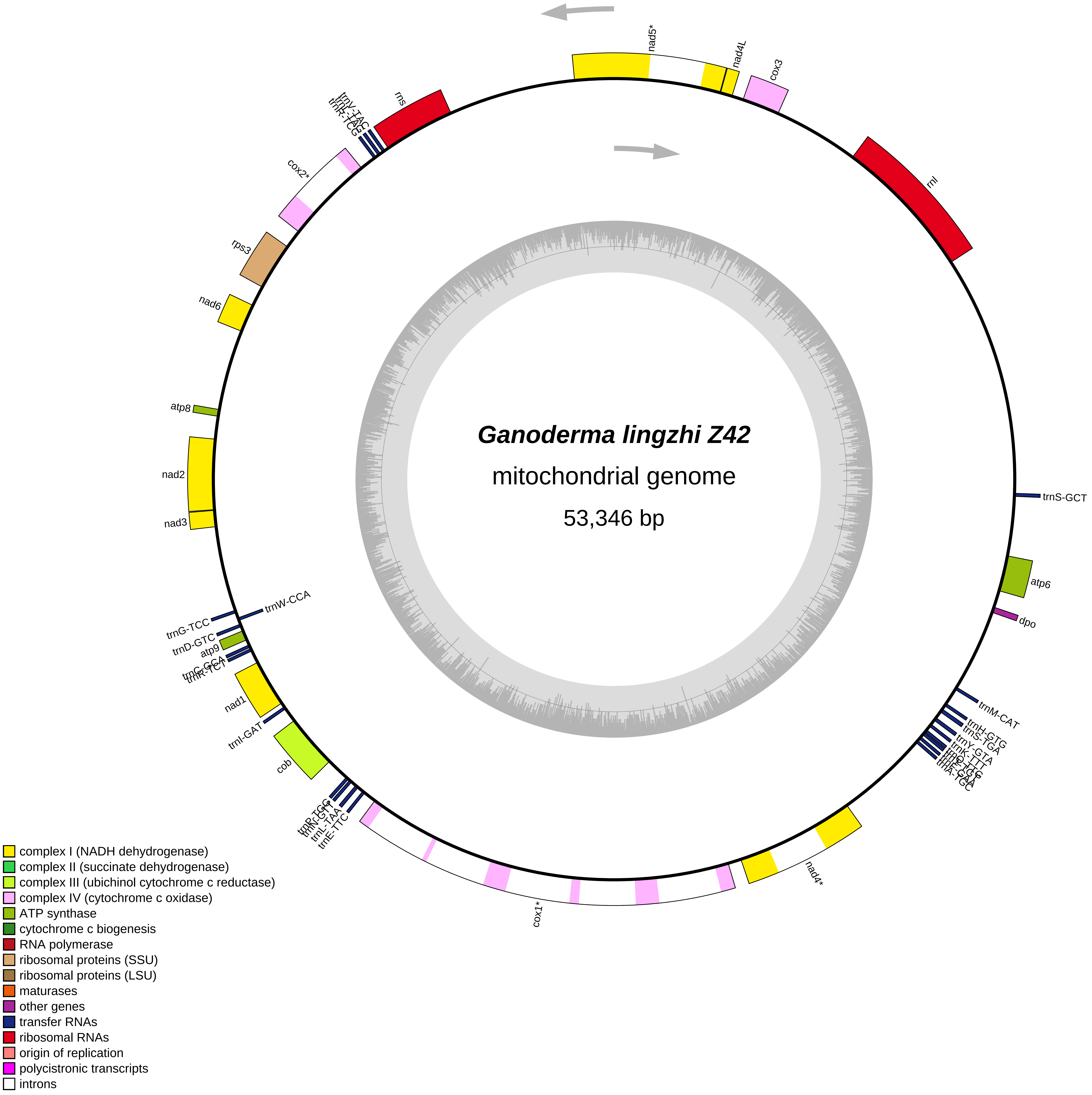
Ganoderma lingzhi Z42
mitochondrial genome
53,346 bp
nad5*
nad4L
cox3
rnl
trnS-GCT
atp6
dpo
trnM-CAT
trnH-GTG
trnS-TGA
trnY-GTA
nad4*
cox1*
trnE-TTC
trnL-TAA
cob
trnI-GAT
nad1
atp9
trnD-GTC
trnG-TCC
trnW-CCA
nad3
nad2
atp8
nad6
rps3
cox2*
rns
trnV-TAC
complex I (NADH dehydrogenase)
complex II (succinate dehydrogenase)
complex III (ubichinol cytochrome c reductase)
complex IV (cytochrome c oxidase)
ATP synthase
cytochrome c biogenesis
RNA polymerase
ribosomal proteins (SSU)
ribosomal proteins (LSU)
maturases
other genes
transfer RNAs
ribosomal RNAs
origin of replication
polycistronic transcripts
introns

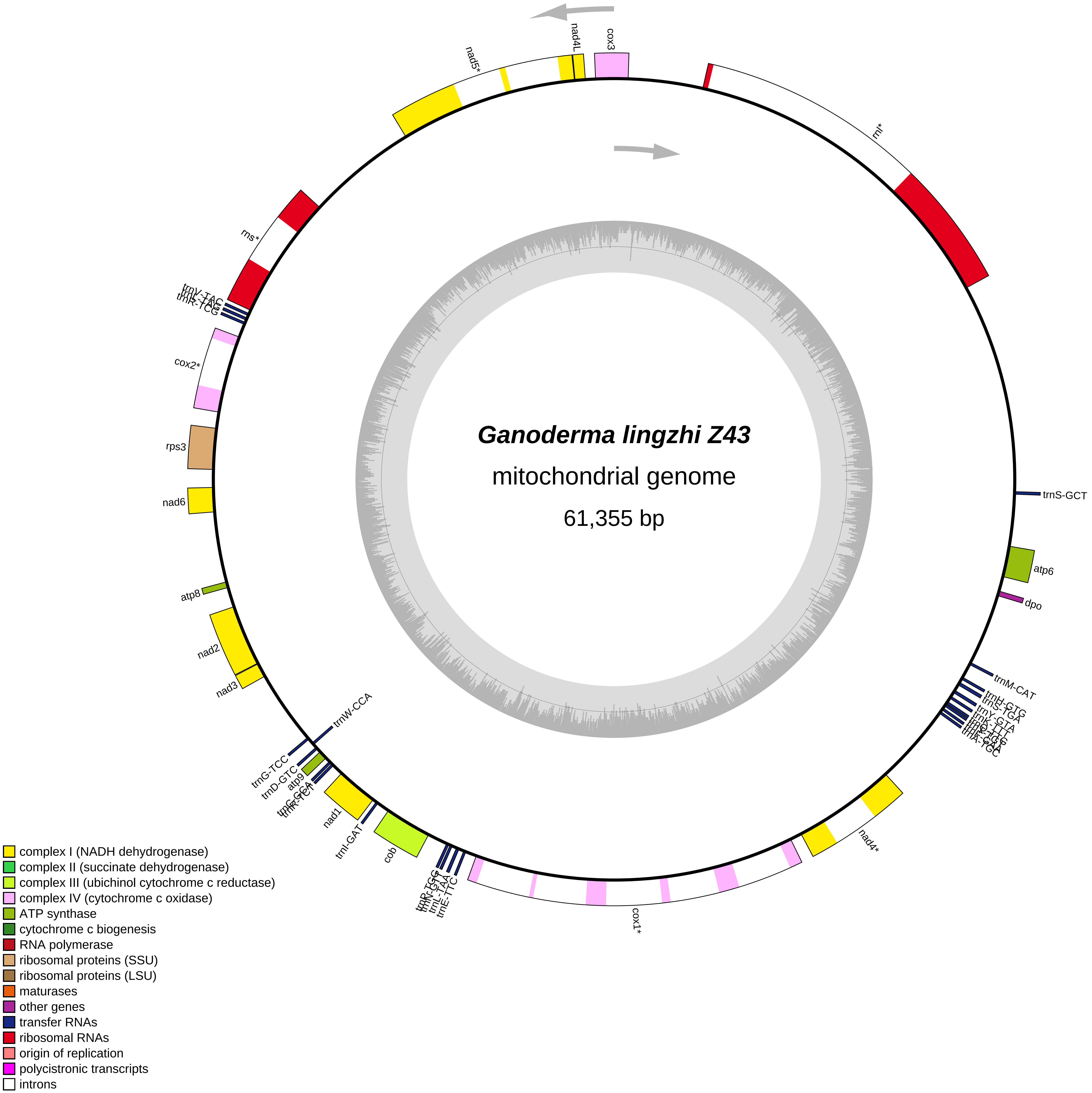

Ganoderma lingzhi Z43
mitochondrial genome
61,355 bp
nad4L
cox3
nad5*
rnl*
rns*
trnV-TAC
trnR-TCG
cox2*
rps3
nad6
atp8
nad2
nad3
trnW-CCA
trnG-TCC
trnD-GTC
atp9
nad1
trnI-GAT
cob
trnE-TTC
cox1*
nad4*
trnM-CAT
trnH-GTG
trnS-TGA
trnY-GTA
trnS-GCT
atp6
dpo
complex I (NADH dehydrogenase)
complex II (succinate dehydrogenase)
complex III (ubichinol cytochrome c reductase)
complex IV (cytochrome c oxidase)
ATP synthase
cytochrome c biogenesis
RNA polymerase
ribosomal proteins (SSU)
ribosomal proteins (LSU)
maturases
other genes
transfer RNAs
ribosomal RNAs
origin of replication
polycistronic transcripts
introns

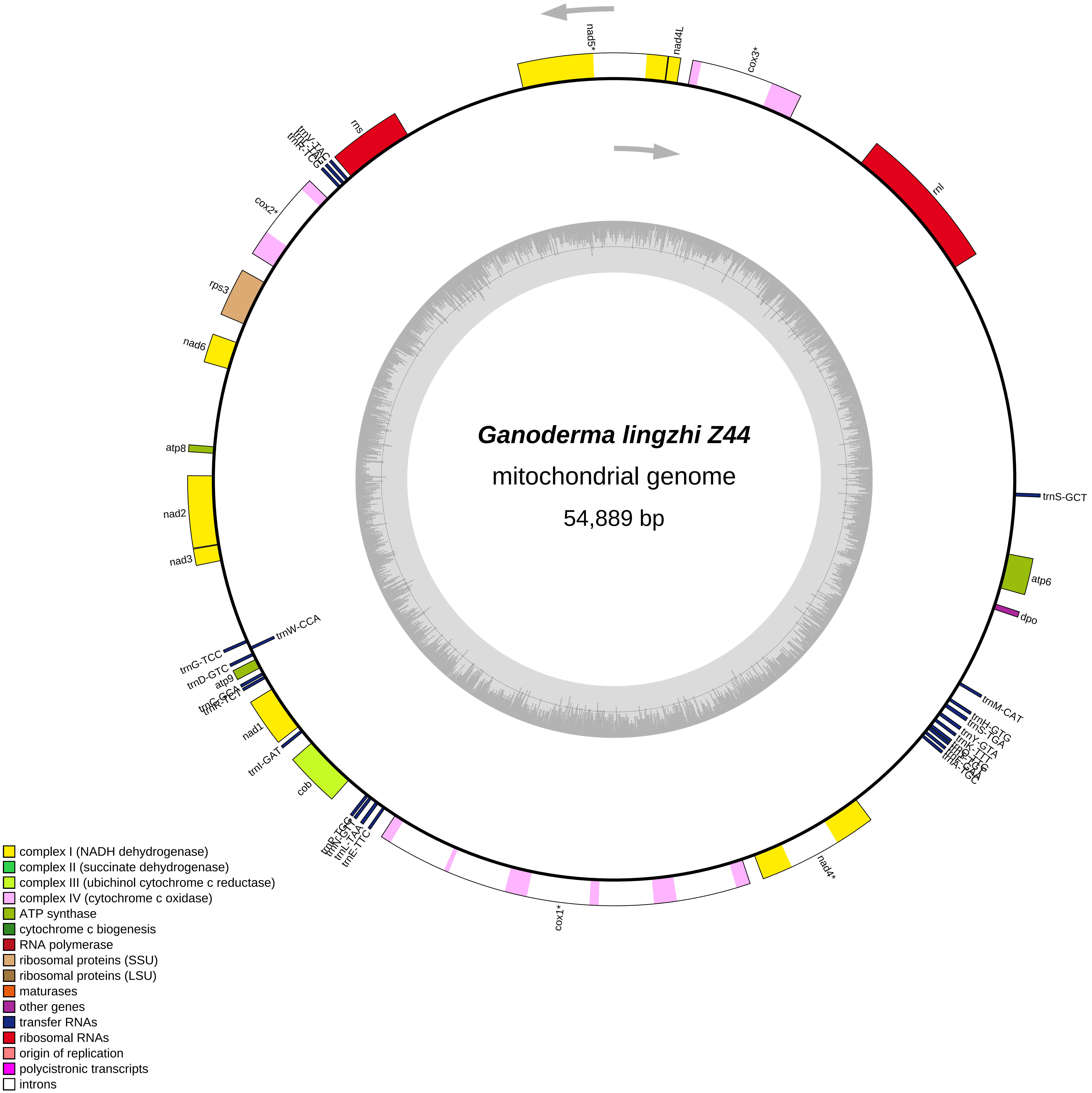

Ganoderma lingzhi Z44
mitochondrial genome
54,889 bp
nad5*
nad4L
cox3*
rnl
trnS-GCT
atp6
dpo
trnM-CAT
trnH-GTG
trnS-TGA
trnY-GTA
nad4*
cox1*
trnE-TTC
trnL-TAA
cob
trnI-GAT
nad1
trnR-TCT
atp9
trnD-GTC
trnG-TCC
trnW-CCA
nad3
nad2
atp8
nad6
rps3
cox2*
trnV-TAC
rns
complex I (NADH dehydrogenase)
complex II (succinate dehydrogenase)
complex III (ubichinol cytochrome c reductase)
complex IV (cytochrome c oxidase)
ATP synthase
cytochrome c biogenesis
RNA polymerase
ribosomal proteins (SSU)
ribosomal proteins (LSU)
maturases
other genes
transfer RNAs
ribosomal RNAs
origin of replication
polycistronic transcripts
introns

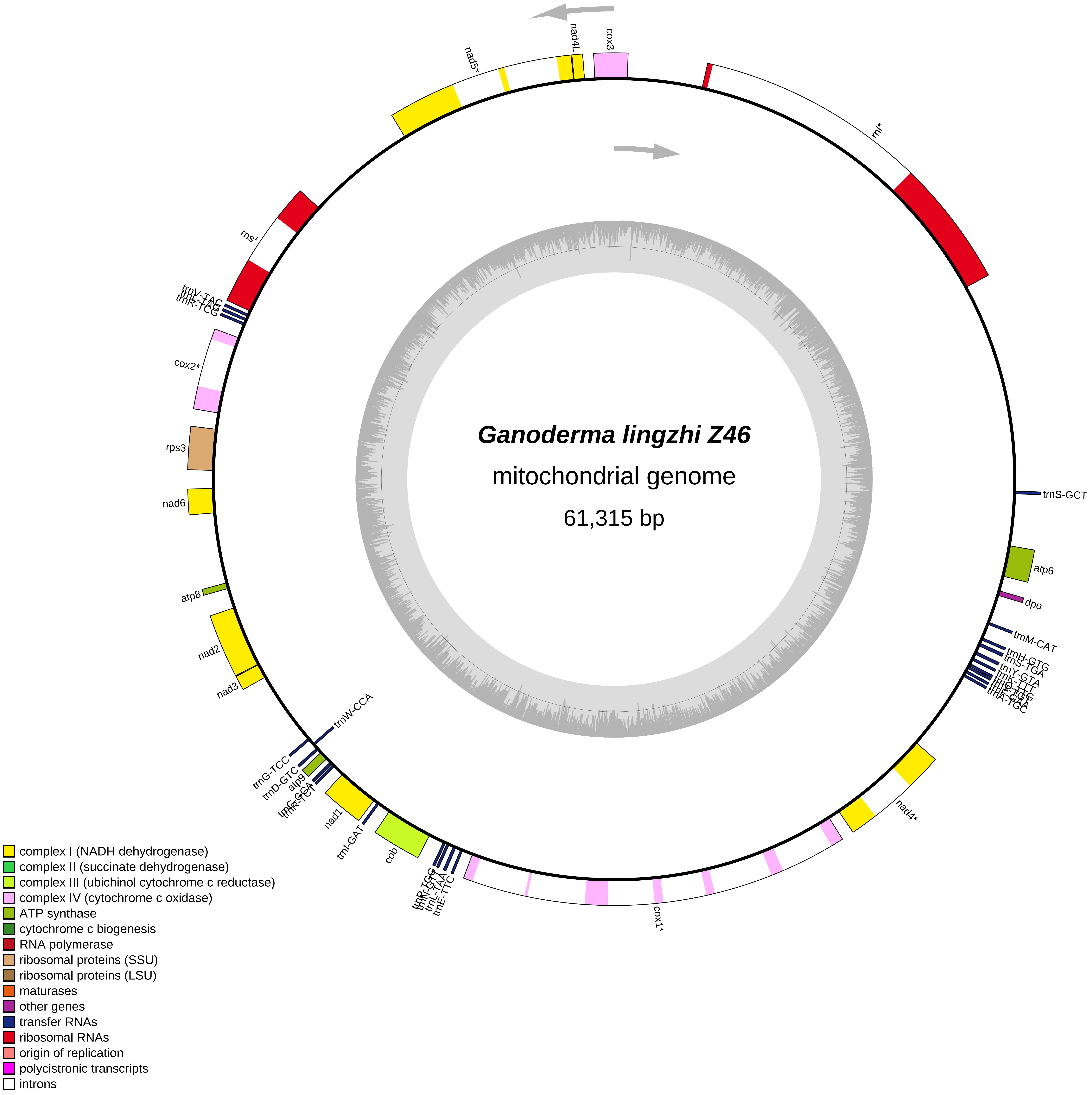

Ganoderma lingzhi Z46
mitochondrial genome
61,315 bp
nad5*
nad4L
cox3
rnl*
rns*
trnV-TAC
trnR-TCG
cox2*
rps3
nad6
atp8
nad2
nad3
trnW-CCA
trnG-TCC
trnD-GTC
atp9
nad1
trnI-GAT
cob
trnE-TTC
cox1*
nad4*
trnA-TGC
trnM-CAT
trnH-GTG
trnS-TGA
trnY-GTA
dpo
atp6
trnS-GCT
complex I (NADH dehydrogenase)
complex II (succinate dehydrogenase)
complex III (ubichinol cytochrome c reductase)
complex IV (cytochrome c oxidase)
ATP synthase
cytochrome c biogenesis
RNA polymerase
ribosomal proteins (SSU)
ribosomal proteins (LSU)
maturases
other genes
transfer RNAs
ribosomal RNAs
origin of replication
polycistronic transcripts
introns

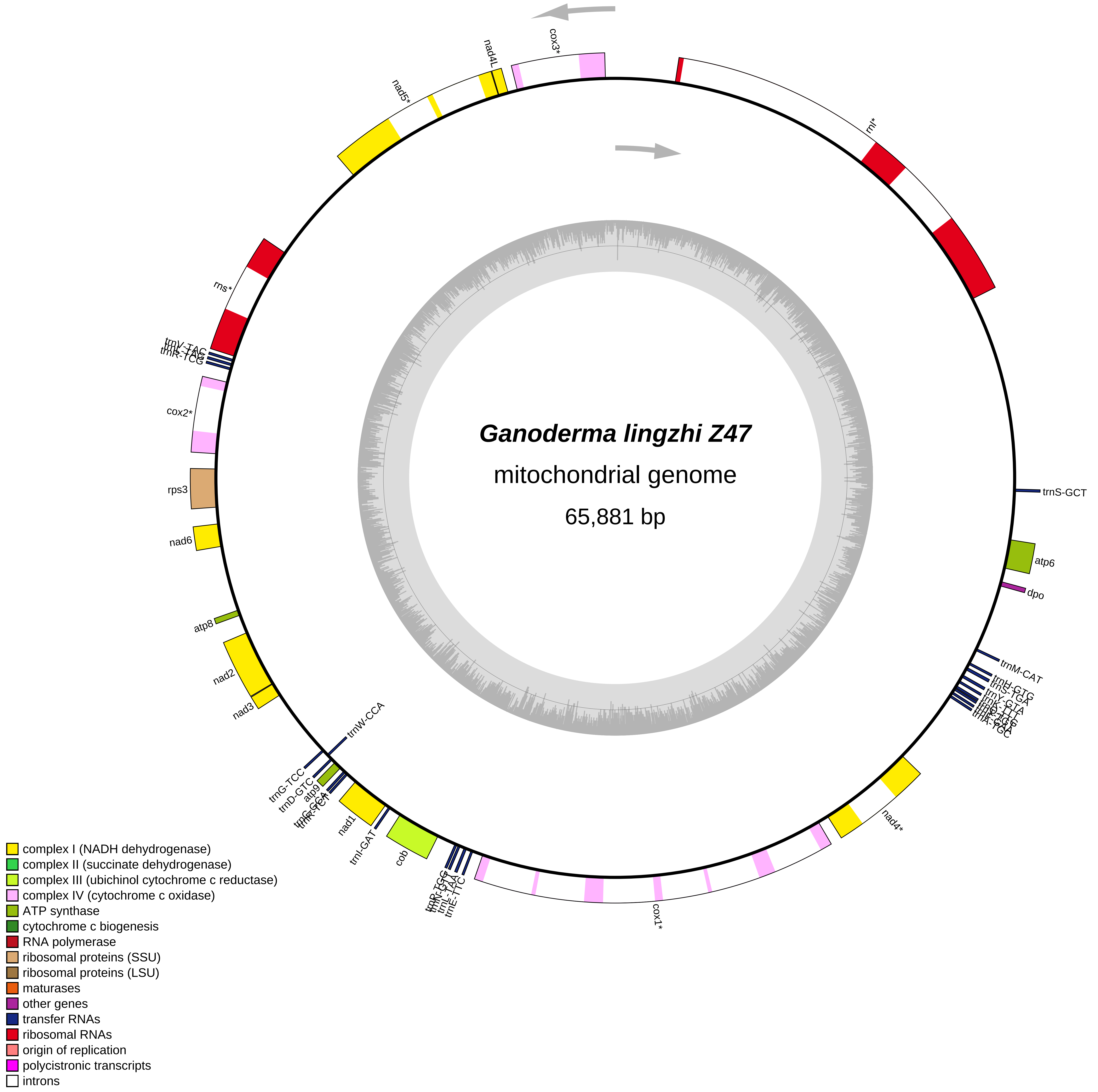
Ganoderma lingzhi Z47
mitochondrial genome
65,881 bp
rnl*
trnS-GCT
atp6
dpo
trnM-CAT
nad4*
cox1*
trnE-TTC
trnL-TAA
cob
trnI-GAT
nad1
atp9
trnD-GTC
trnG-TCC
trnW-CCA
nad3
nad2
atp8
nad6
rps3
cox2*
trnR-TCG
rns*
nad5*
nad4L
cox3*
complex I (NADH dehydrogenase)
complex II (succinate dehydrogenase)
complex III (ubichinol cytochrome c reductase)
complex IV (cytochrome c oxidase)
ATP synthase
cytochrome c biogenesis
RNA polymerase
ribosomal proteins (SSU)
ribosomal proteins (LSU)
maturases
other genes
transfer RNAs
ribosomal RNAs
origin of replication
polycistronic transcripts
introns

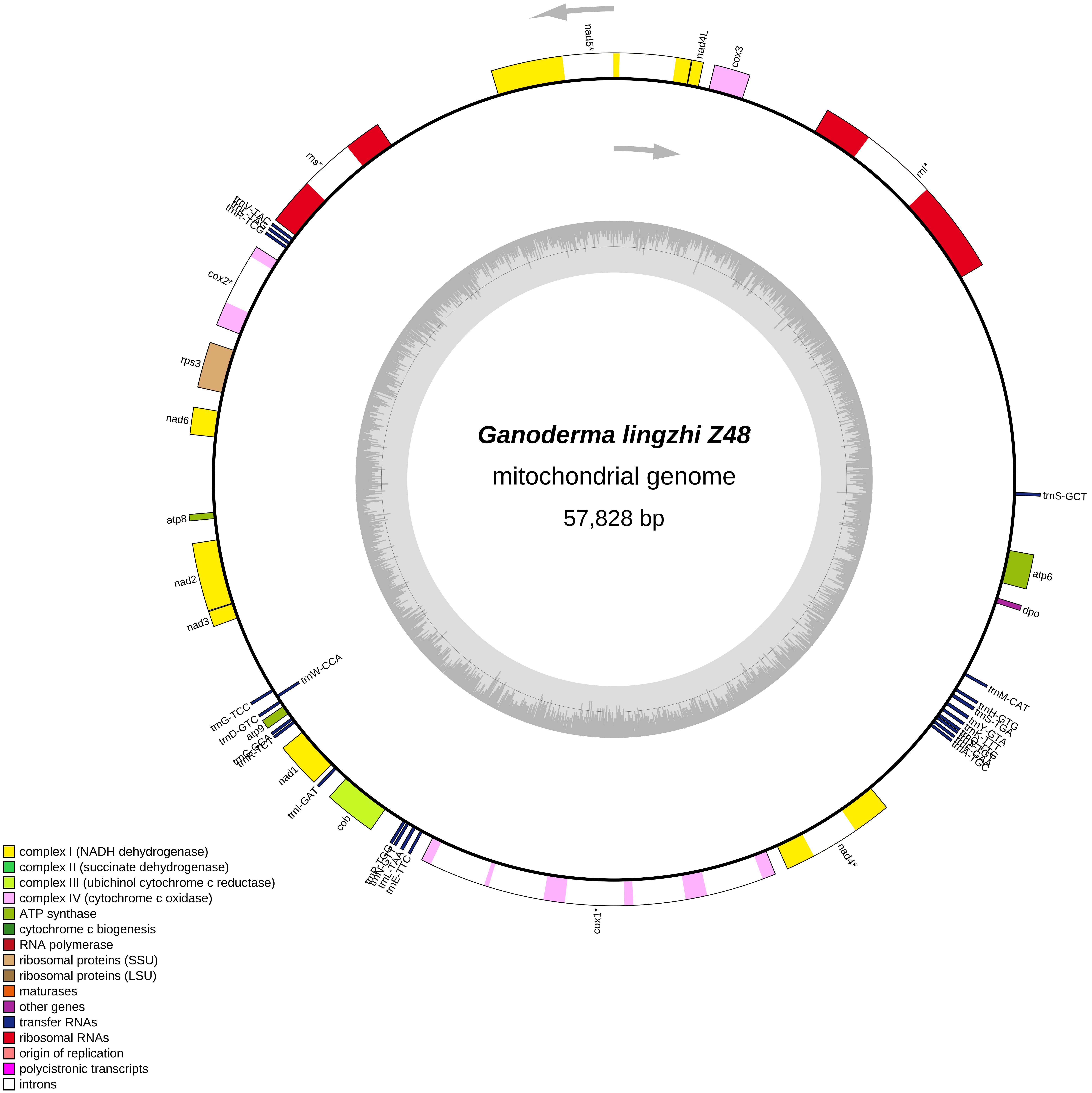

Ganoderma lingzhi Z48
mitochondrial genome
57,828 bp
nad5*
nad4L
cox3
rnl*
trnS-GCT
atp6
dpo
trnM-CAT
trnH-GTG
trnS-TGA
trnY-GTA
nad4*
cox1*
trnE-TTC
trnL-TAA
cob
trnI-GAT
nad1
atp9
trnD-GTC
trnG-TCC
trnW-CCA
nad3
nad2
atp8
nad6
rps3
cox2*
trnV-TAC
trnR-TCG
rns*
complex I (NADH dehydrogenase)
complex II (succinate dehydrogenase)
complex III (ubichinol cytochrome c reductase)
complex IV (cytochrome c oxidase)
ATP synthase
cytochrome c biogenesis
RNA polymerase
ribosomal proteins (SSU)
ribosomal proteins (LSU)
maturases
other genes
transfer RNAs
ribosomal RNAs
origin of replication
polycistronic transcripts
introns

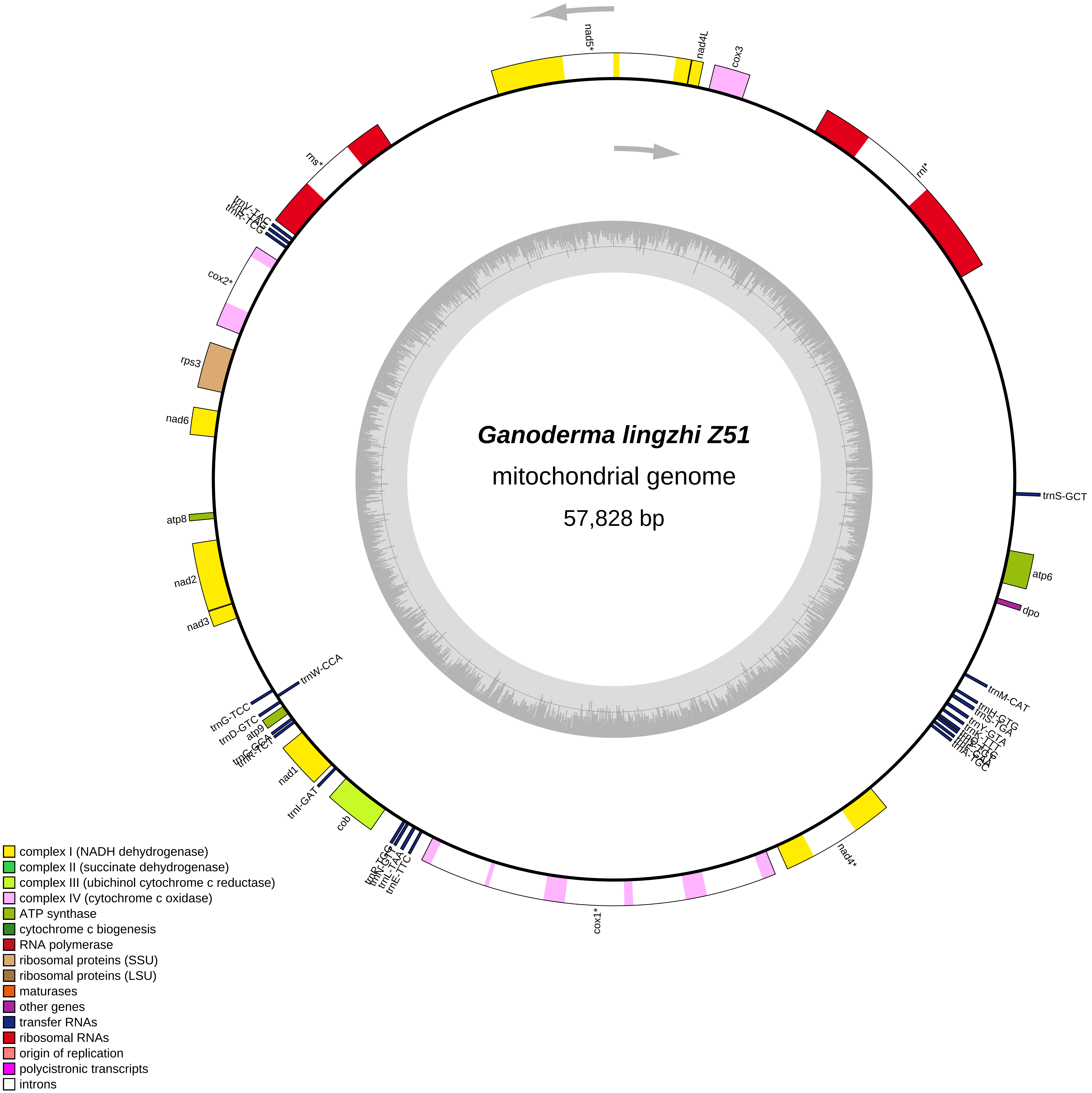

Ganoderma lingzhi Z51
mitochondrial genome
57,828 bp
nad5*
nad4L
cox3
rnl*
rns*
trnV-TAC
trnR-TCG
cox2*
rps3
nad6
atp8
nad2
nad3
trnS-GCT
atp6
dpo
trnM-CAT
trnH-GTG
trnS-TGA
trnY-GTA
trnA-TGC
trnW-CCA
trnG-TCC
trnD-GTC
atp9
trnC-GCA
trnR-TCT
nad1
trnI-GAT
cob
trnR-TCG
trnL-TAA
trnE-TTC
cox1*
nad4*
complex I (NADH dehydrogenase)
complex II (succinate dehydrogenase)
complex III (ubichinol cytochrome c reductase)
complex IV (cytochrome c oxidase)
ATP synthase
cytochrome c biogenesis
RNA polymerase
ribosomal proteins (SSU)
ribosomal proteins (LSU)
maturases
other genes
transfer RNAs
ribosomal RNAs
origin of replication
polycistronic transcripts
introns

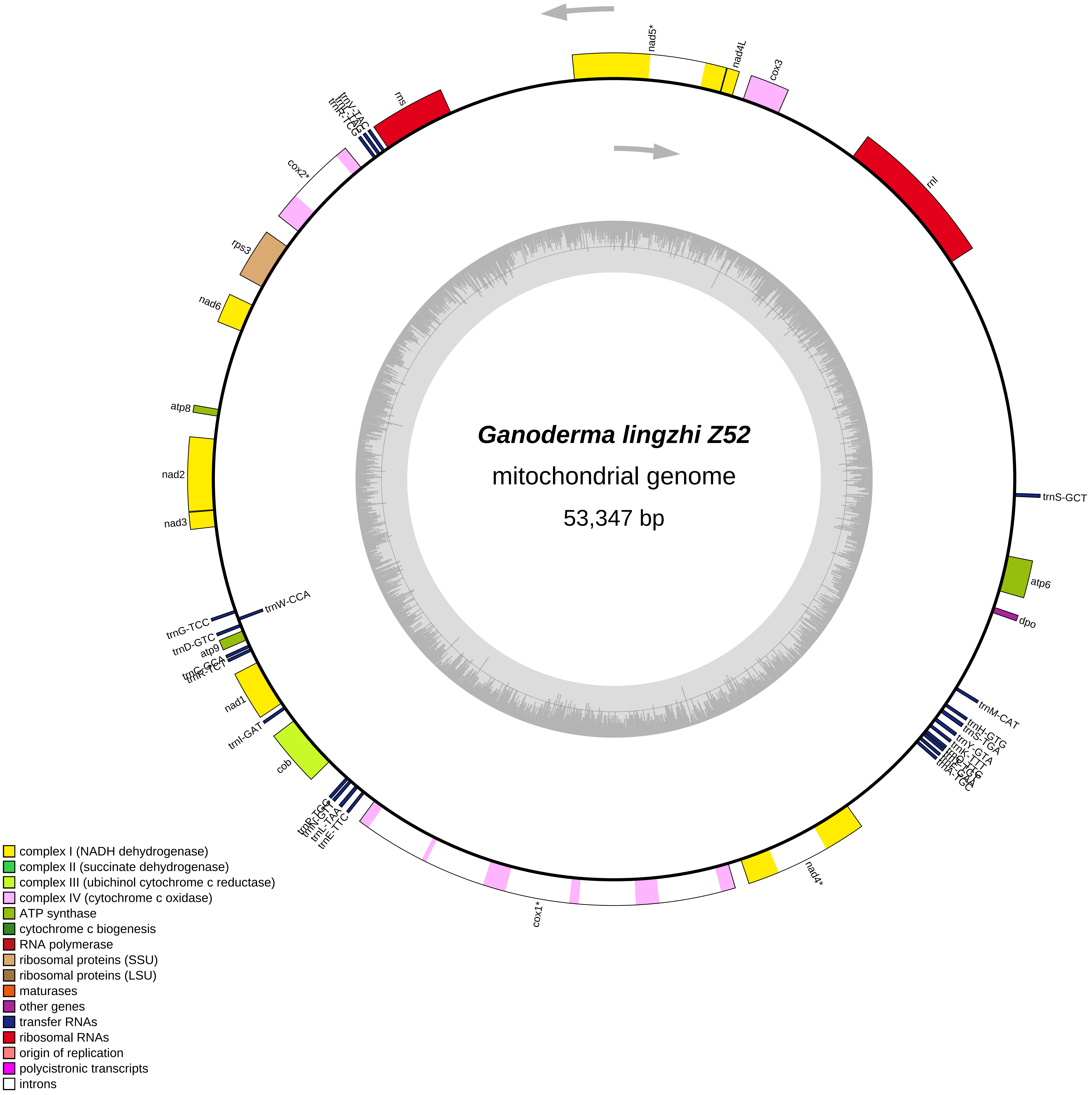

Ganoderma lingzhi Z52
mitochondrial genome
53,347 bp
nad5*
nad4L
cox3
rnl
trnS-GCT
atp6
dpo
trnM-CAT
trnH-GTG
trnS-TGA
trnY-GTA
trnK-TTT
nad4*
cox1*
trnE-TTC
trnL-TAA
trnP-TGG
cob
trnI-GAT
nad1
trnR-TCT
trnC-GCA
atp9
trnD-GTC
trnG-TCC
trnW-CCA
nad3
nad2
atp8
nad6
rps3
cox2*
trnV-TAC
rns
complex I (NADH dehydrogenase)
complex II (succinate dehydrogenase)
complex III (ubichinol cytochrome c reductase)
complex IV (cytochrome c oxidase)
ATP synthase
cytochrome c biogenesis
RNA polymerase
ribosomal proteins (SSU)
ribosomal proteins (LSU)
maturases
other genes
transfer RNAs
ribosomal RNAs
origin of replication
polycistronic transcripts
introns

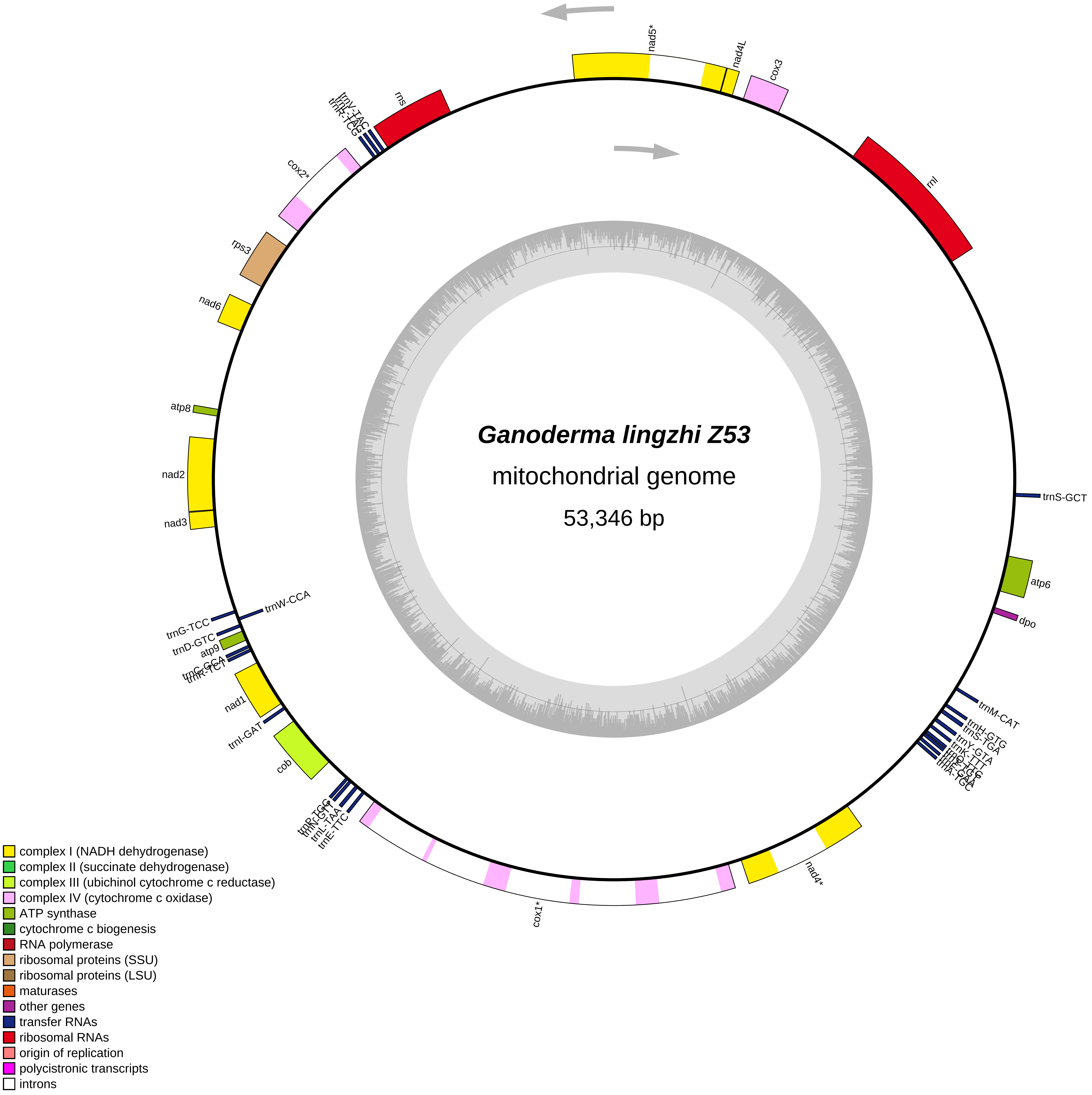
Ganoderma lingzhi Z53
mitochondrial genome
53,346 bp
nad5*
nad4L
cox3
rnl
trnS-GCT
atp6
dpo
trnM-CAT
trnH-GTG
trnS-TGA
trnY-GTA
nad4*
cox1*
trnE-TTC
trnL-TAA
cob
trnI-GAT
nad1
trnR-TCT
atp9
trnD-GTC
trnG-TCC
trnW-CCA
nad3
nad2
atp8
nad6
rps3
cox2*
rns
trnV-TAC
complex I (NADH dehydrogenase)
complex II (succinate dehydrogenase)
complex III (ubichinol cytochrome c reductase)
complex IV (cytochrome c oxidase)
ATP synthase
cytochrome c biogenesis
RNA polymerase
ribosomal proteins (SSU)
ribosomal proteins (LSU)
maturases
other genes
transfer RNAs
ribosomal RNAs
origin of replication
polycistronic transcripts
introns

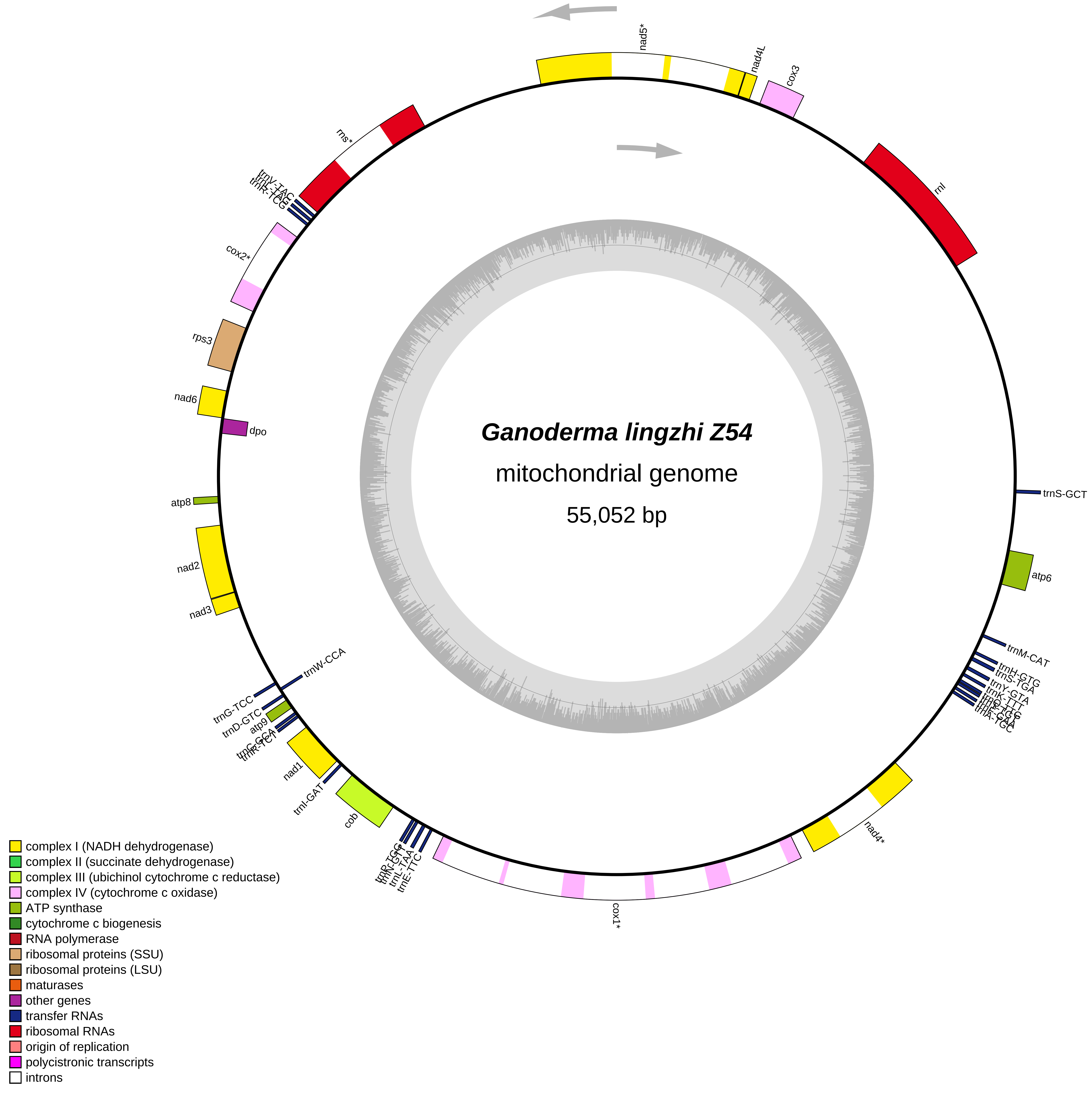
Ganoderma lingzhi Z54
mitochondrial genome
55,052 bp
nad5*
nad4L
cox3
rnl
trnS-GCT
atp6
trnM-CAT
trnH-GTG
trnS-TGA
trnY-GTA
trnK-TTT
nad4*
cox1*
trnE-TTC
trnL-TAA
cob
trnI-GAT
nad1
trnR-TCT
atp9
trnD-GTC
trnG-TCC
trnW-CCA
nad3
nad2
atp8
dpo
nad6
rps3
cox2*
trnR-TCG
trnV-TAC
rns*
complex I (NADH dehydrogenase)
complex II (succinate dehydrogenase)
complex III (ubichinol cytochrome c reductase)
complex IV (cytochrome c oxidase)
ATP synthase
cytochrome c biogenesis
RNA polymerase
ribosomal proteins (SSU)
ribosomal proteins (LSU)
maturases
other genes
transfer RNAs
ribosomal RNAs
origin of replication
polycistronic transcripts
introns

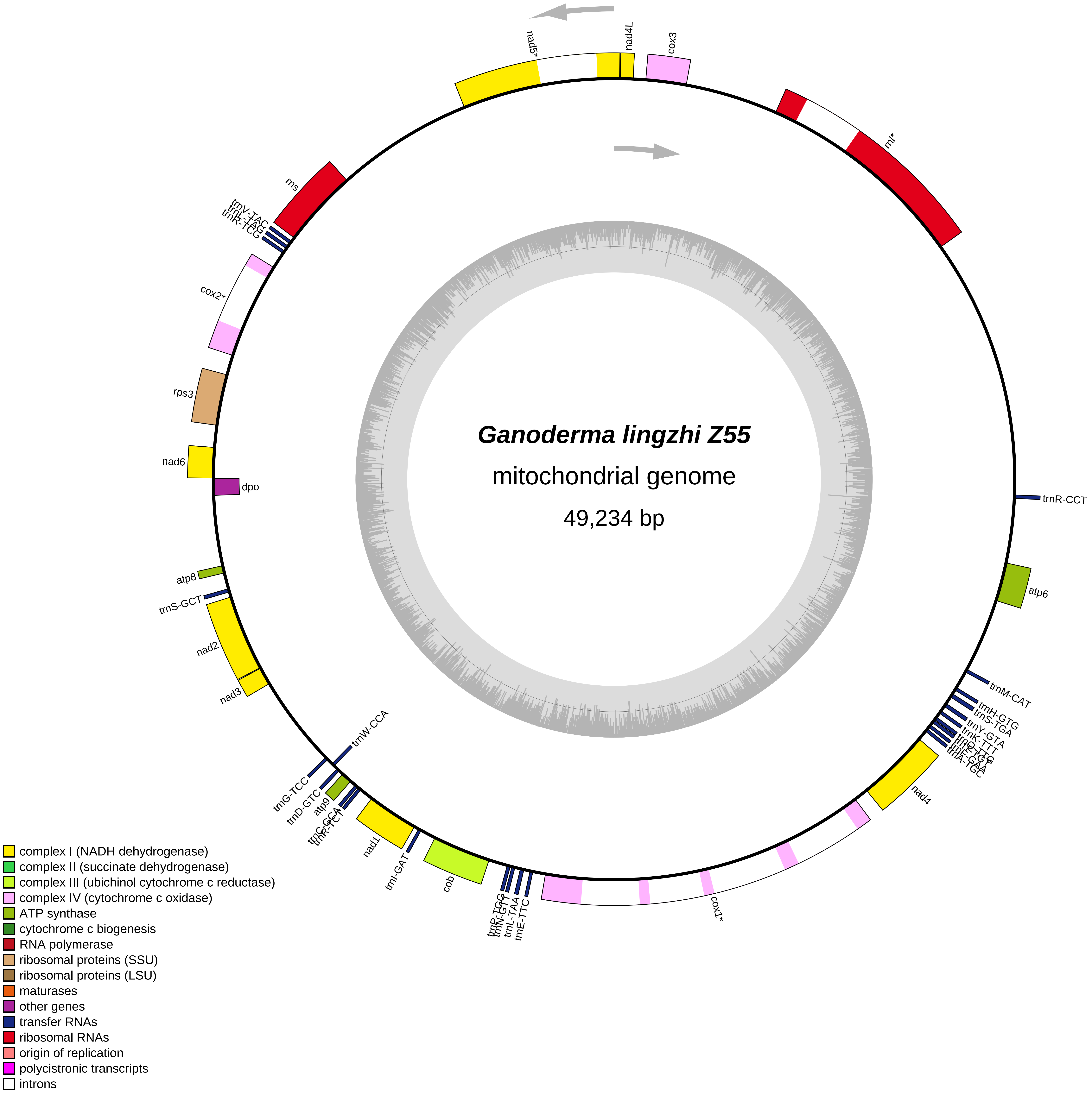

Ganoderma lingzhi Z55
mitochondrial genome
49,234 bp
nad5*
nad4L
cox3
rnl*
rns
trnV-TAC
trnR-TCG
cox2*
rps3
nad6
dpo
trnR-CCT
atp6
atp8
trnS-GCT
nad2
nad3
trnM-CAT
trnH-GTG
trnS-TGA
trnY-GTA
trnK-TTT
trnA-TGC
nad4
cox1*
trnE-TTC
trnL-TAA
trnP-TGG
cob
trnI-GAT
nad1
trnW-CCA
trnG-TCC
trnD-GTC
atp9
complex I (NADH dehydrogenase)
complex II (succinate dehydrogenase)
complex III (ubichinol cytochrome c reductase)
complex IV (cytochrome c oxidase)
ATP synthase
cytochrome c biogenesis
RNA polymerase
ribosomal proteins (SSU)
ribosomal proteins (LSU)
maturases
other genes
transfer RNAs
ribosomal RNAs
origin of replication
polycistronic transcripts
introns

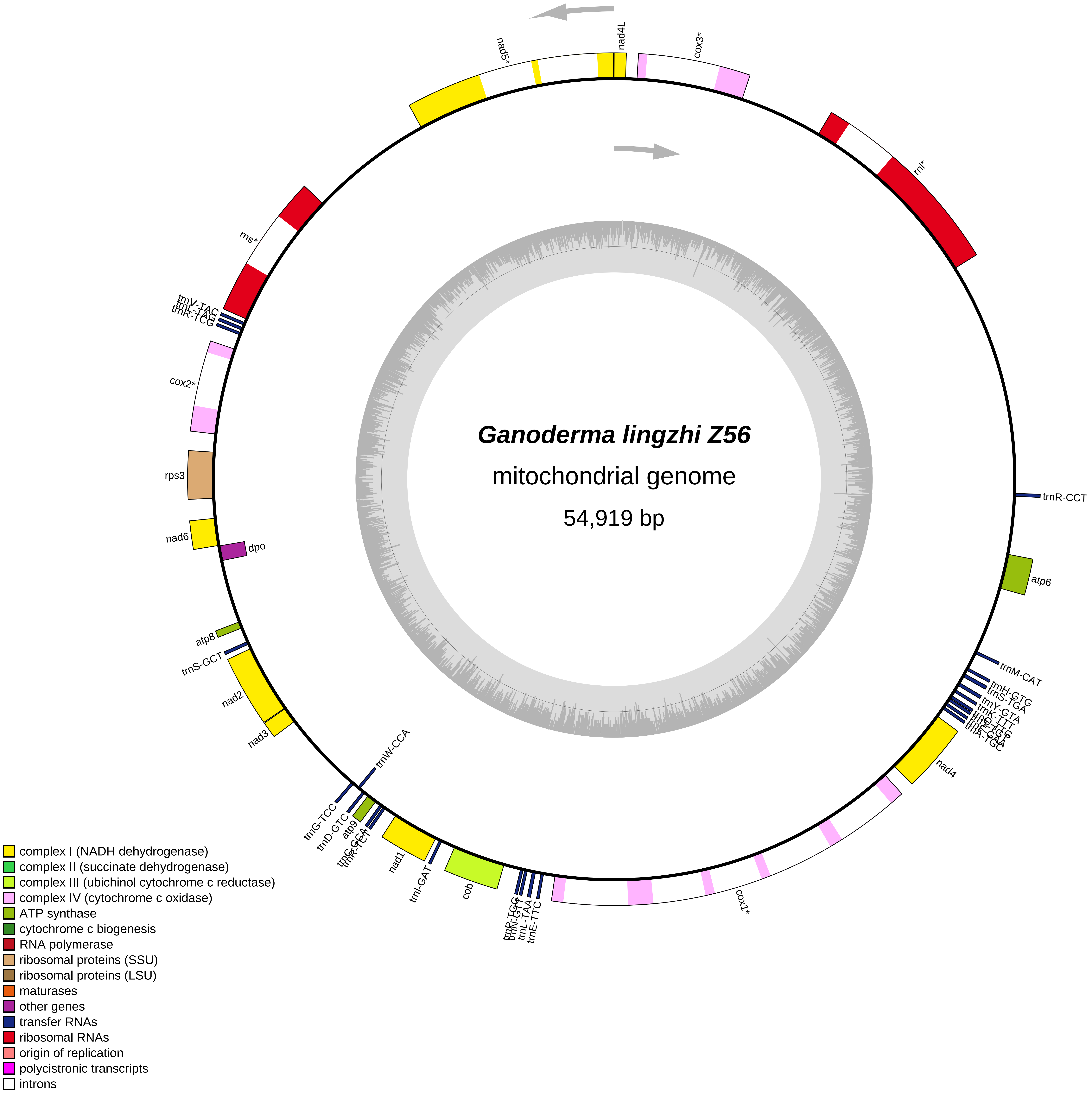
Ganoderma lingzhi Z56
mitochondrial genome
54,919 bp
nad4L
nad5*
cox3*
rnl*
rns*
trnV-TAC
trnL-TAG
trnR-TCG
cox2*
rps3
nad6
dpo
atp8
trnS-GCT
nad2
nad3
trnW-CCA
trnG-TCC
trnD-GTC
atp9
nad1
trnI-GAT
cob
trnN-GTT
trnL-TAA
trnE-TTC
cox1*
nad4
trnA-TGC
trnY-GTA
trnS-TGA
trnH-GTG
trnM-CAT
atp6
trnR-CCT
complex I (NADH dehydrogenase)
complex II (succinate dehydrogenase)
complex III (ubichinol cytochrome c reductase)
complex IV (cytochrome c oxidase)
ATP synthase
cytochrome c biogenesis
RNA polymerase
ribosomal proteins (SSU)
ribosomal proteins (LSU)
maturases
other genes
transfer RNAs
ribosomal RNAs
origin of replication
polycistronic transcripts
introns

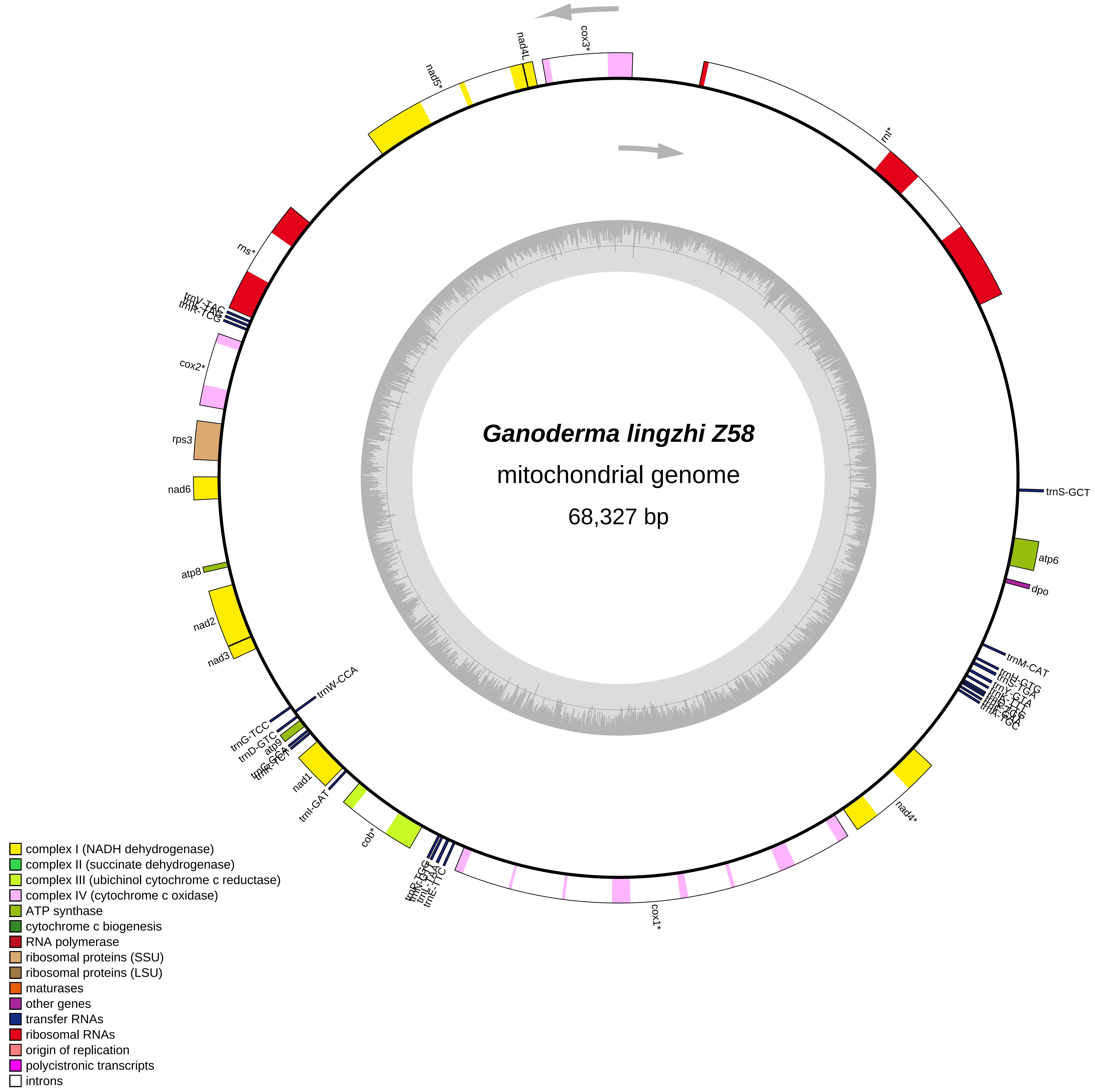
Ganoderma lingzhi Z58
mitochondrial genome
68,327 bp
cox3*
nad4L
nad5*
rnl*
rns*
cox2*
rps3
nad6
atp8
nad2
nad3
trnW-CCA
trnG-TCC
trnD-GTC
atp9
nad1
trnI-GAT
cob*
trnE-TTC
cox1*
nad4*
trnM-CAT
dpo
atp6
trnS-GCT
complex I (NADH dehydrogenase)
complex II (succinate dehydrogenase)
complex III (ubichinol cytochrome c reductase)
complex IV (cytochrome c oxidase)
ATP synthase
cytochrome c biogenesis
RNA polymerase
ribosomal proteins (SSU)
ribosomal proteins (LSU)
maturases
other genes
transfer RNAs
ribosomal RNAs
origin of replication
polycistronic transcripts
introns

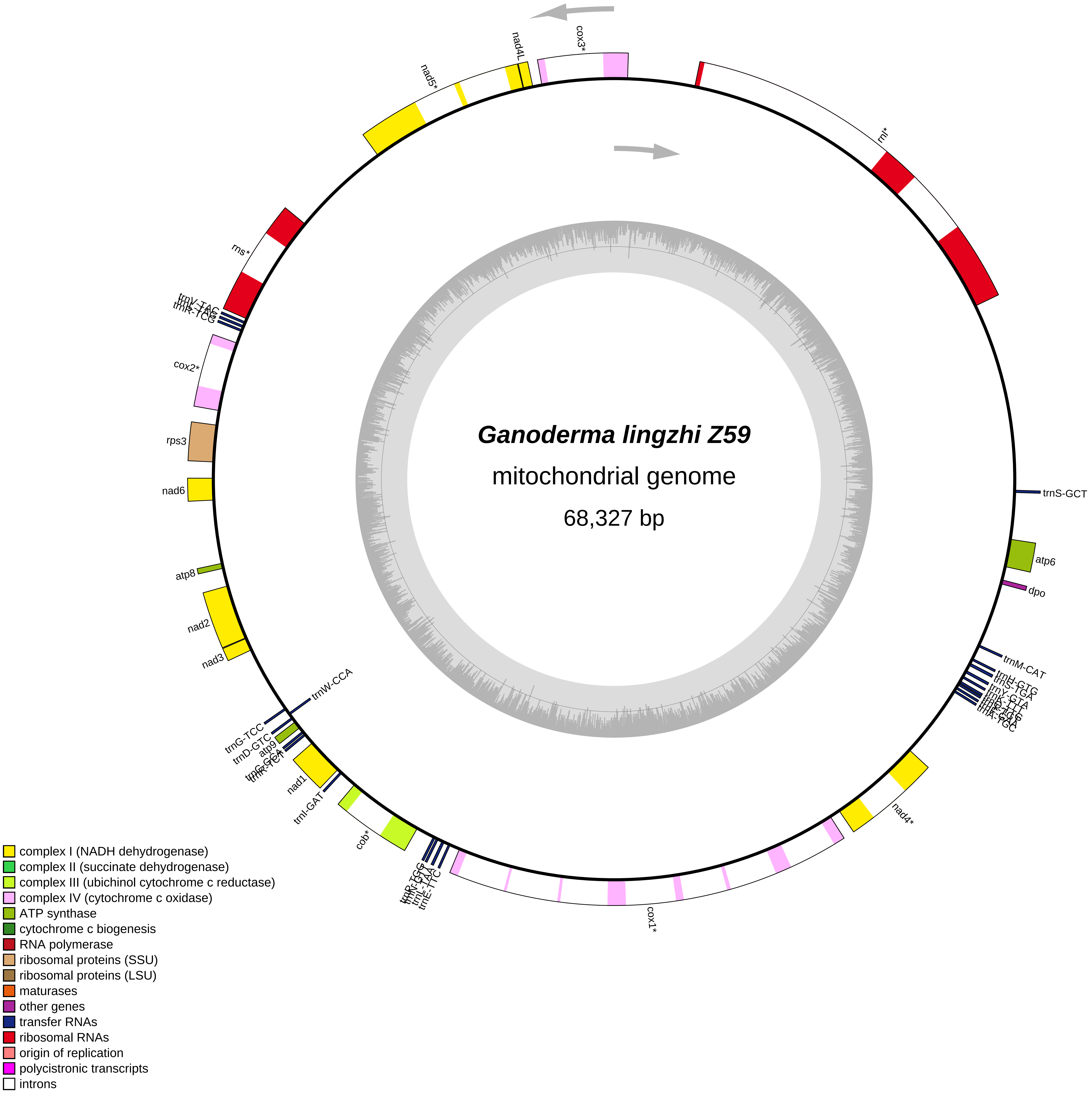

Ganoderma lingzhi Z59
mitochondrial genome
68,327 bp
cox3*
nad4L
nad5*
rnl*
rns*
trnV-TAC
trnR-TCG
cox2*
rps3
nad6
atp8
nad2
nad3
trnW-CCA
trnG-TCC
trnD-GTC
atp9
nad1
trnI-GAT
cob*
trnP-TGG
trnL-TAA
trnE-TTC
cox1*
nad4*
trnM-CAT
trnH-GTG
trnS-TGA
trnS-GCT
atp6
dpo
complex I (NADH dehydrogenase)
complex II (succinate dehydrogenase)
complex III (ubichinol cytochrome c reductase)
complex IV (cytochrome c oxidase)
ATP synthase
cytochrome c biogenesis
RNA polymerase
ribosomal proteins (SSU)
ribosomal proteins (LSU)
maturases
other genes
transfer RNAs
ribosomal RNAs
origin of replication
polycistronic transcripts
introns

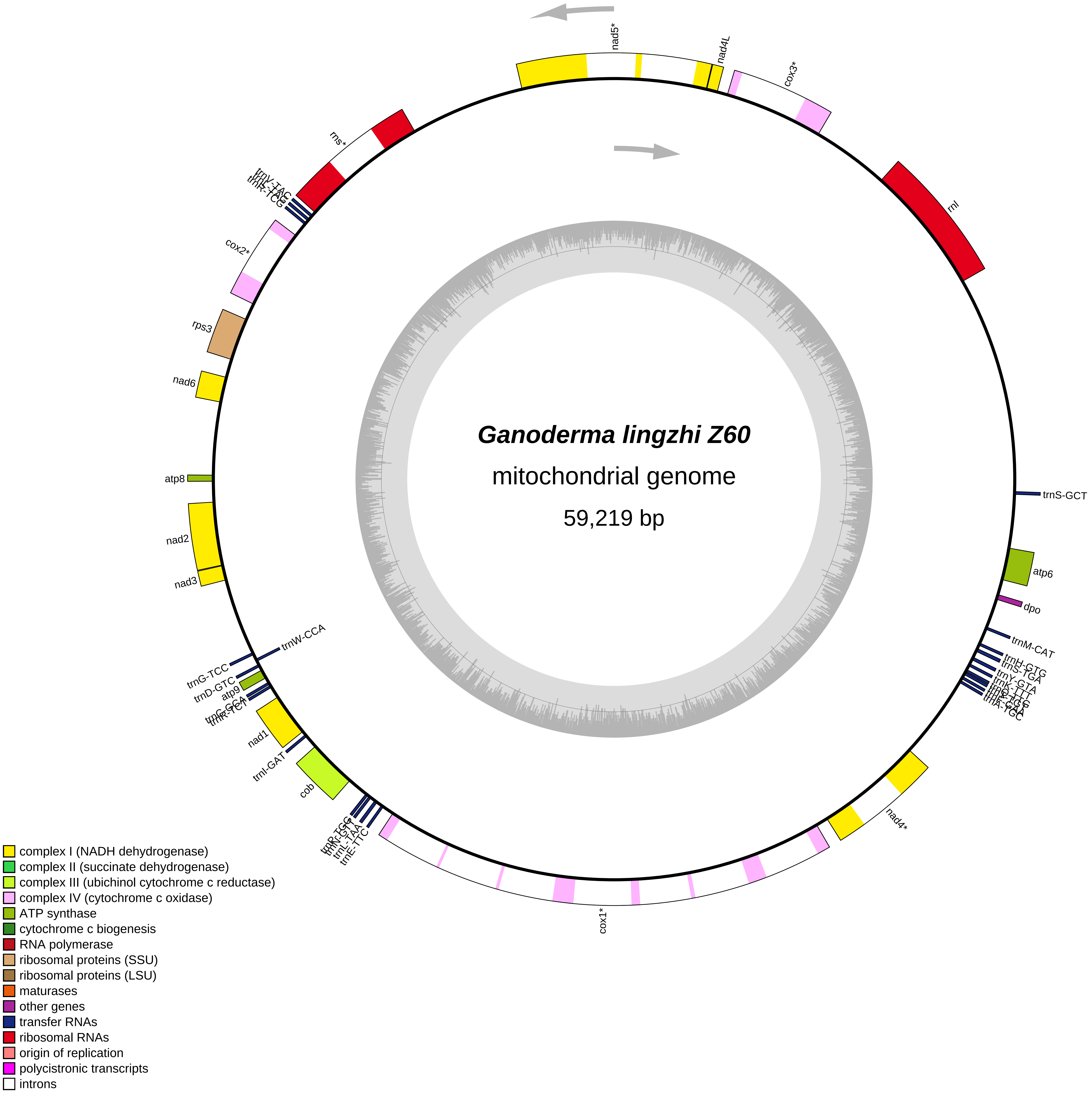
Ganoderma lingzhi Z60
mitochondrial genome
59,219 bp
nad5*
nad4L
cox3*
rnl
trnS-GCT
atp6
dpo
trnM-CAT
trnH-GTG
trnS-TGA
trnY-GTA
nad4*
cox1*
trnE-TTC
trnL-TAA
trnN-GTT
trnP-TGG
cob
trnI-GAT
nad1
trnR-TCT
trnC-GCA
atp9
trnD-GTC
trnG-TCC
trnW-CCA
nad3
nad2
atp8
nad6
rps3
cox2*
trnV-TAC
trnR-TCG
rns*
complex I (NADH dehydrogenase)
complex II (succinate dehydrogenase)
complex III (ubichinol cytochrome c reductase)
complex IV (cytochrome c oxidase)
ATP synthase
cytochrome c biogenesis
RNA polymerase
ribosomal proteins (SSU)
ribosomal proteins (LSU)
maturases
other genes
transfer RNAs
ribosomal RNAs
origin of replication
polycistronic transcripts
introns

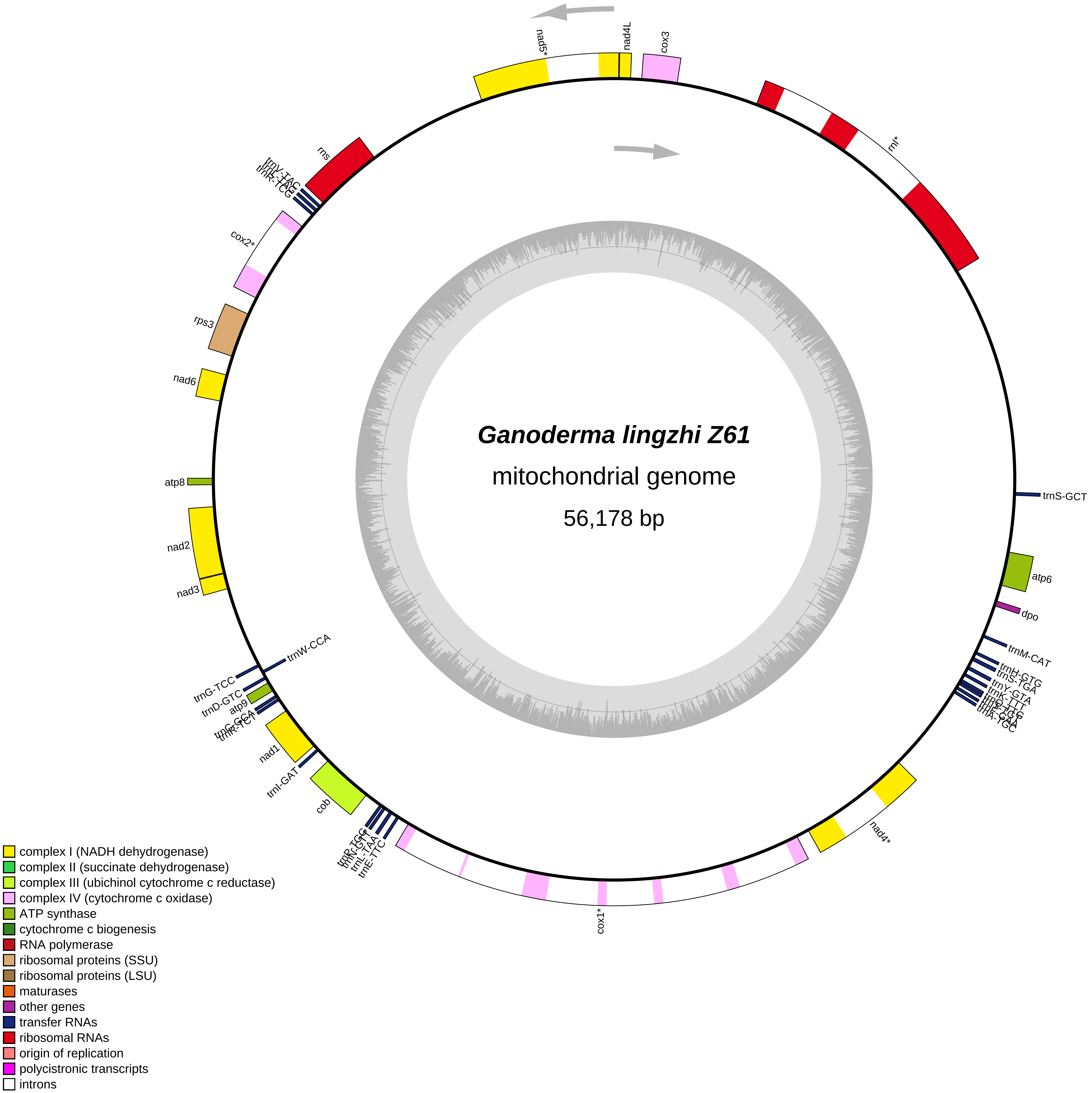

Ganoderma lingzhi Z61
mitochondrial genome
56,178 bp
nad5*
nad4L
cox3
rnl*
trnS-GCT
atp6
dpo
trnM-CAT
trnH-GTG
trnS-TGA
trnY-GTA
nad4*
cox1*
trnE-TTC
trnL-TAA
cob
trnI-GAT
nad1
trnR-TCT
atp9
trnD-GTC
trnG-TCC
trnW-CCA
nad3
nad2
atp8
nad6
rps3
cox2*
trnR-TGG
trnV-TAC
rns
complex I (NADH dehydrogenase)
complex II (succinate dehydrogenase)
complex III (ubichinol cytochrome c reductase)
complex IV (cytochrome c oxidase)
ATP synthase
cytochrome c biogenesis
RNA polymerase
ribosomal proteins (SSU)
ribosomal proteins (LSU)
maturases
other genes
transfer RNAs
ribosomal RNAs
origin of replication
polycistronic transcripts
introns

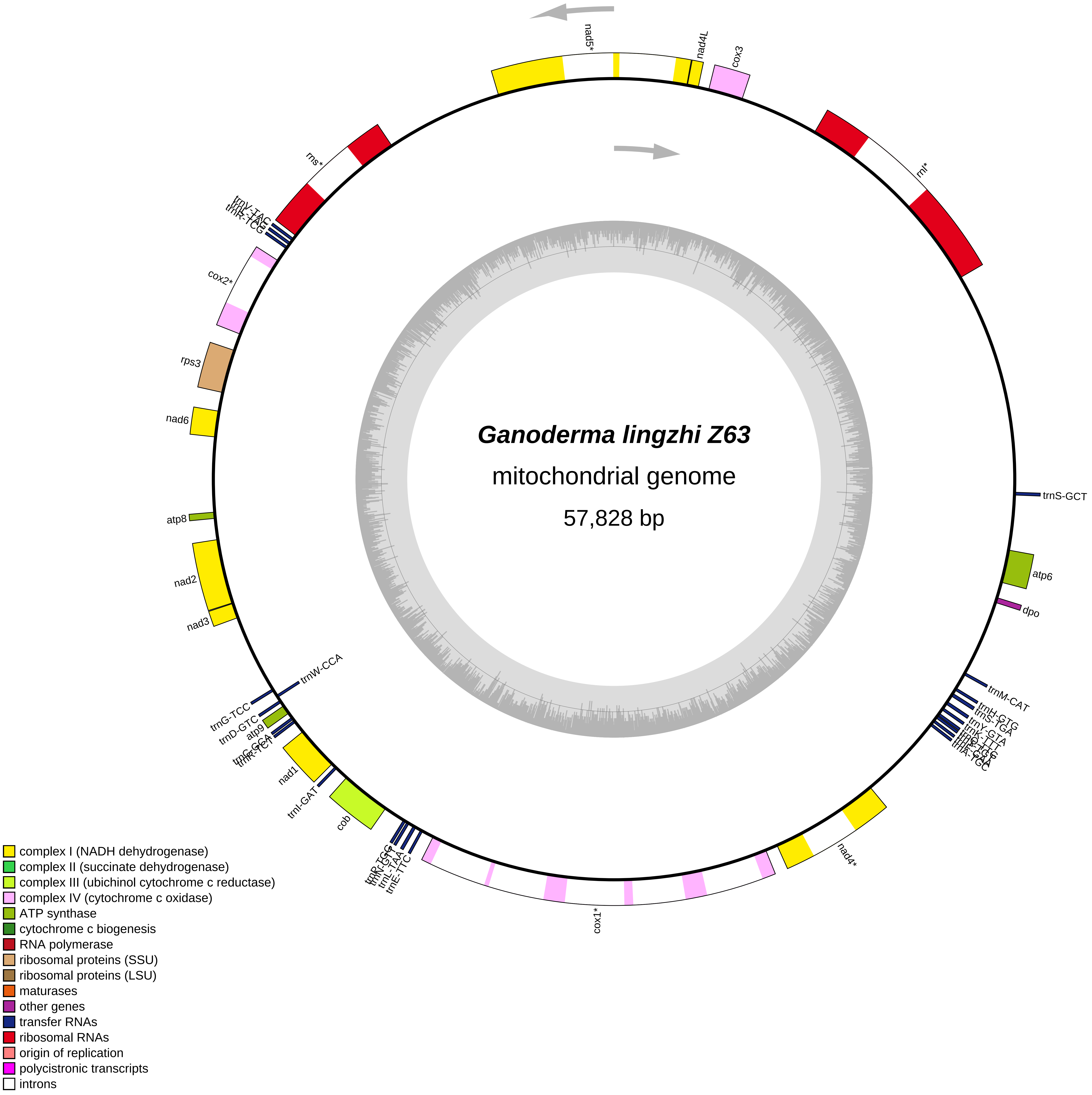

Ganoderma lingzhi Z63
mitochondrial genome
57,828 bp
nad5*
nad4L
cox3
rnl*
trnS-GCT
atp6
dpo
trnM-CAT
trnH-GTG
trnS-TGA
trnY-GTA
trnA-TGC
nad4*
cox1*
trnE-TTC
trnL-TAA
cob
trnI-GAT
nad1
trnR-TCT
atp9
trnD-GTC
trnG-TCC
trnW-CCA
nad3
nad2
atp8
nad6
rps3
cox2*
trnR-TCG
trnV-TAC
rns*
complex I (NADH dehydrogenase)
complex II (succinate dehydrogenase)
complex III (ubichinol cytochrome c reductase)
complex IV (cytochrome c oxidase)
ATP synthase
cytochrome c biogenesis
RNA polymerase
ribosomal proteins (SSU)
ribosomal proteins (LSU)
maturases
other genes
transfer RNAs
ribosomal RNAs
origin of replication
polycistronic transcripts
introns

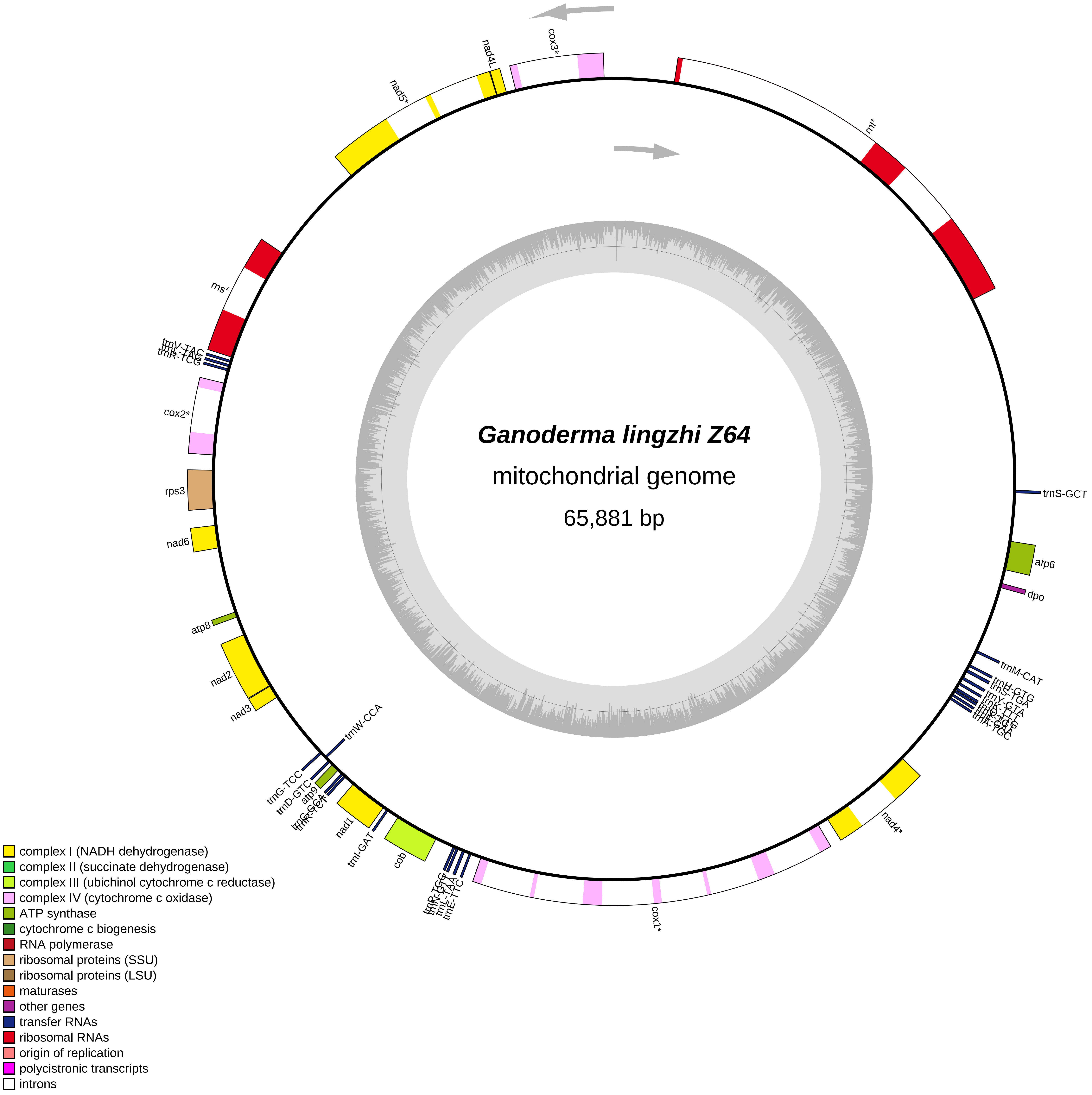
Ganoderma lingzhi Z64
mitochondrial genome
65,881 bp
cox3*
nad4L
nad5*
rnl*
rns*
trnV-TAC
trnR-TCG
cox2*
rps3
nad6
atp8
nad2
nad3
trnW-CCA
trnG-TCC
trnD-GTC
atp9
nad1
trnI-GAT
cob
trnE-TTC
cox1*
nad4*
trnM-CAT
trnH-GTG
trnS-TGA
trnY-GTA
dpo
atp6
trnS-GCT
complex I (NADH dehydrogenase)
complex II (succinate dehydrogenase)
complex III (ubichinol cytochrome c reductase)
complex IV (cytochrome c oxidase)
ATP synthase
cytochrome c biogenesis
RNA polymerase
ribosomal proteins (SSU)
ribosomal proteins (LSU)
maturases
other genes
transfer RNAs
ribosomal RNAs
origin of replication
polycistronic transcripts
introns

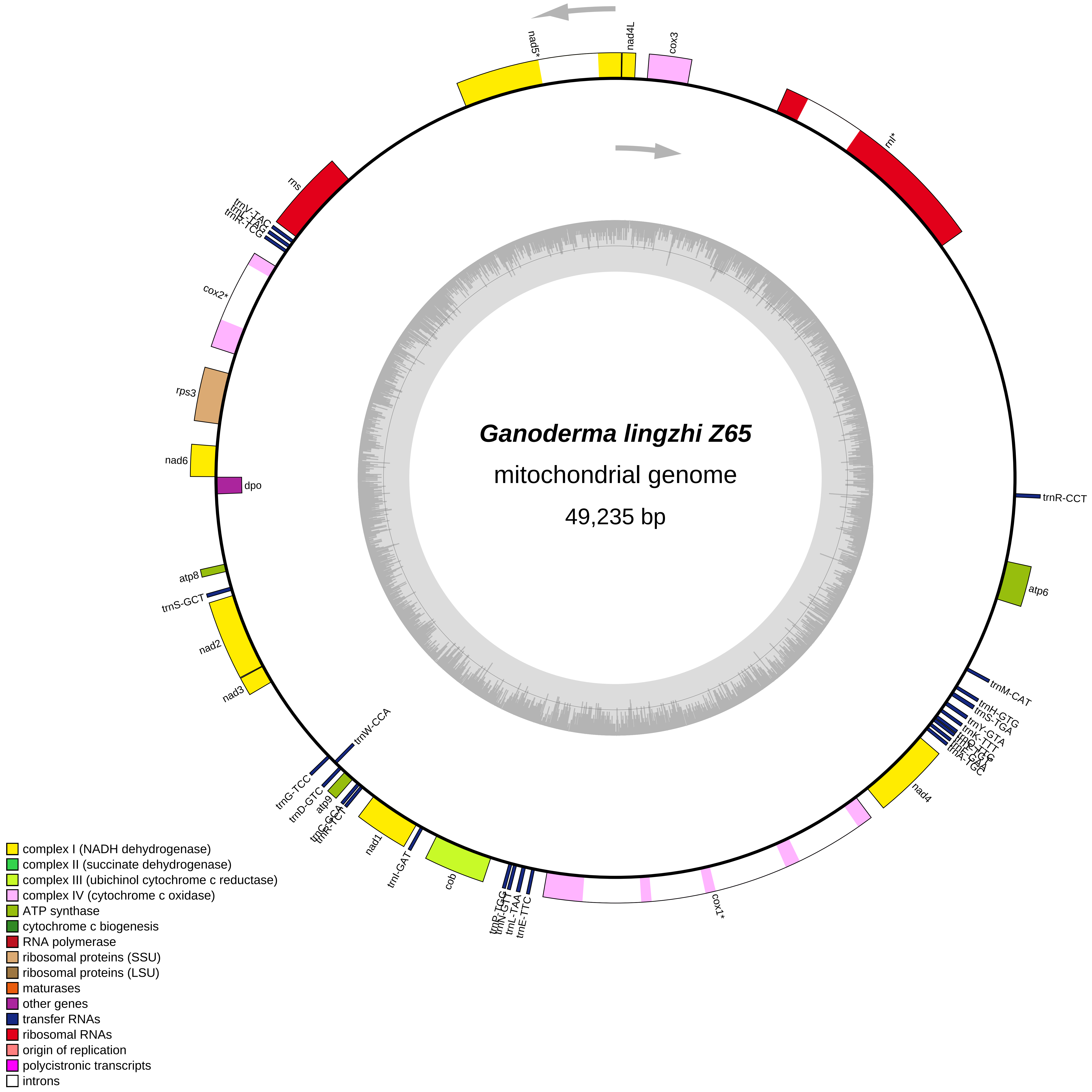
Ganoderma lingzhi Z65
mitochondrial genome
49,235 bp
nad5*
nad4L
cox3
rnl*
rns
trnV-TAC
trnL-TAG
trnR-TCG
cox2*
rps3
nad6
dpo
trnR-CCT
atp6
atp8
trnS-GCT
nad2
nad3
trnW-CCA
trnM-CAT
trnH-GTG
trnS-TGA
trnY-GTA
trnK-TTT
trnA-TGC
nad4
cox1*
trnE-TTC
trnL-TAA
trnP-TGG
cob
trnI-GAT
nad1
atp9
trnD-GTC
trnG-TCC
complex I (NADH dehydrogenase)
complex II (succinate dehydrogenase)
complex III (ubichinol cytochrome c reductase)
complex IV (cytochrome c oxidase)
ATP synthase
cytochrome c biogenesis
RNA polymerase
ribosomal proteins (SSU)
ribosomal proteins (LSU)
maturases
other genes
transfer RNAs
ribosomal RNAs
origin of replication
polycistronic transcripts
introns

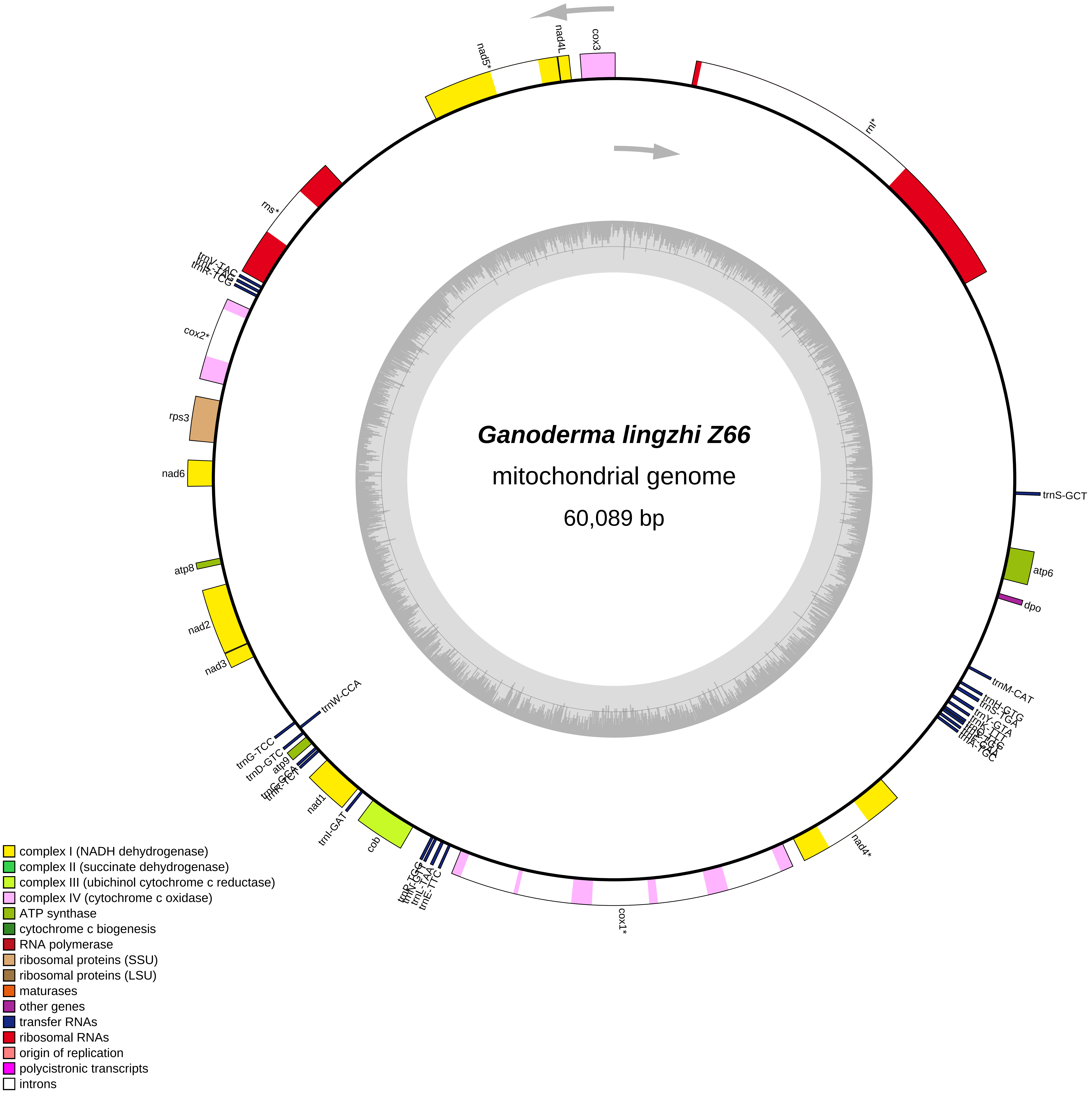

Ganoderma lingzhi Z66
mitochondrial genome
60,089 bp
nad5*
nad4L
cox3
rnl*
rns*
trnV-TAC
trnR-TCG
cox2*
rps3
nad6
atp8
nad2
nad3
trnW-CCA
trnG-TCC
trnD-GTC
atp9
nad1
trnI-GAT
cob
trnL-TAA
trnE-TTC
cox1*
nad4*
trnM-CAT
trnH-GTG
trnS-TGA
trnY-GTA
trnA-TGC
dpo
atp6
trnS-GCT
complex I (NADH dehydrogenase)
complex II (succinate dehydrogenase)
complex III (ubichinol cytochrome c reductase)
complex IV (cytochrome c oxidase)
ATP synthase
cytochrome c biogenesis
RNA polymerase
ribosomal proteins (SSU)
ribosomal proteins (LSU)
maturases
other genes
transfer RNAs
ribosomal RNAs
origin of replication
polycistronic transcripts
introns

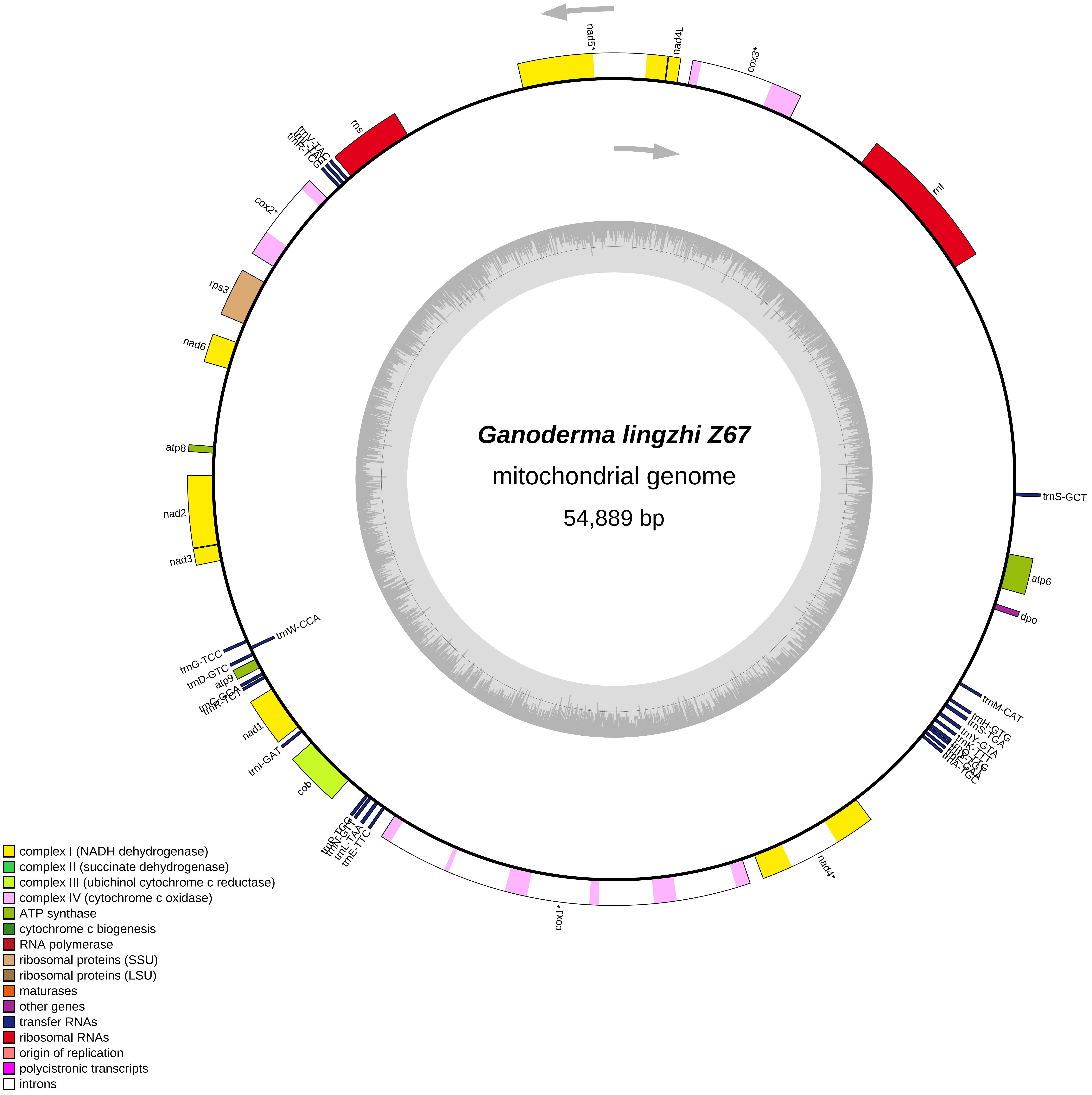

Ganoderma lingzhi Z67
mitochondrial genome
54,889 bp
nad5*
nad4L
cox3*
rnl
rns
trnV-TAC
cox2*
rps3
nad6
atp8
nad2
nad3
trnS-GCT
atp6
dpo
trnM-CAT
trnH-GTG
trnS-TGA
trnY-GTA
trnW-CCA
trnG-TCC
trnD-GTC
atp9
trnC-GCA
trnR-TCT
nad1
trnI-GAT
cob
trnP-TGG
trnN-GTT
trnL-TAA
trnE-TTC
cox1*
nad4*
complex I (NADH dehydrogenase)
complex II (succinate dehydrogenase)
complex III (ubichinol cytochrome c reductase)
complex IV (cytochrome c oxidase)
ATP synthase
cytochrome c biogenesis
RNA polymerase
ribosomal proteins (SSU)
ribosomal proteins (LSU)
maturases
other genes
transfer RNAs
ribosomal RNAs
origin of replication
polycistronic transcripts
introns

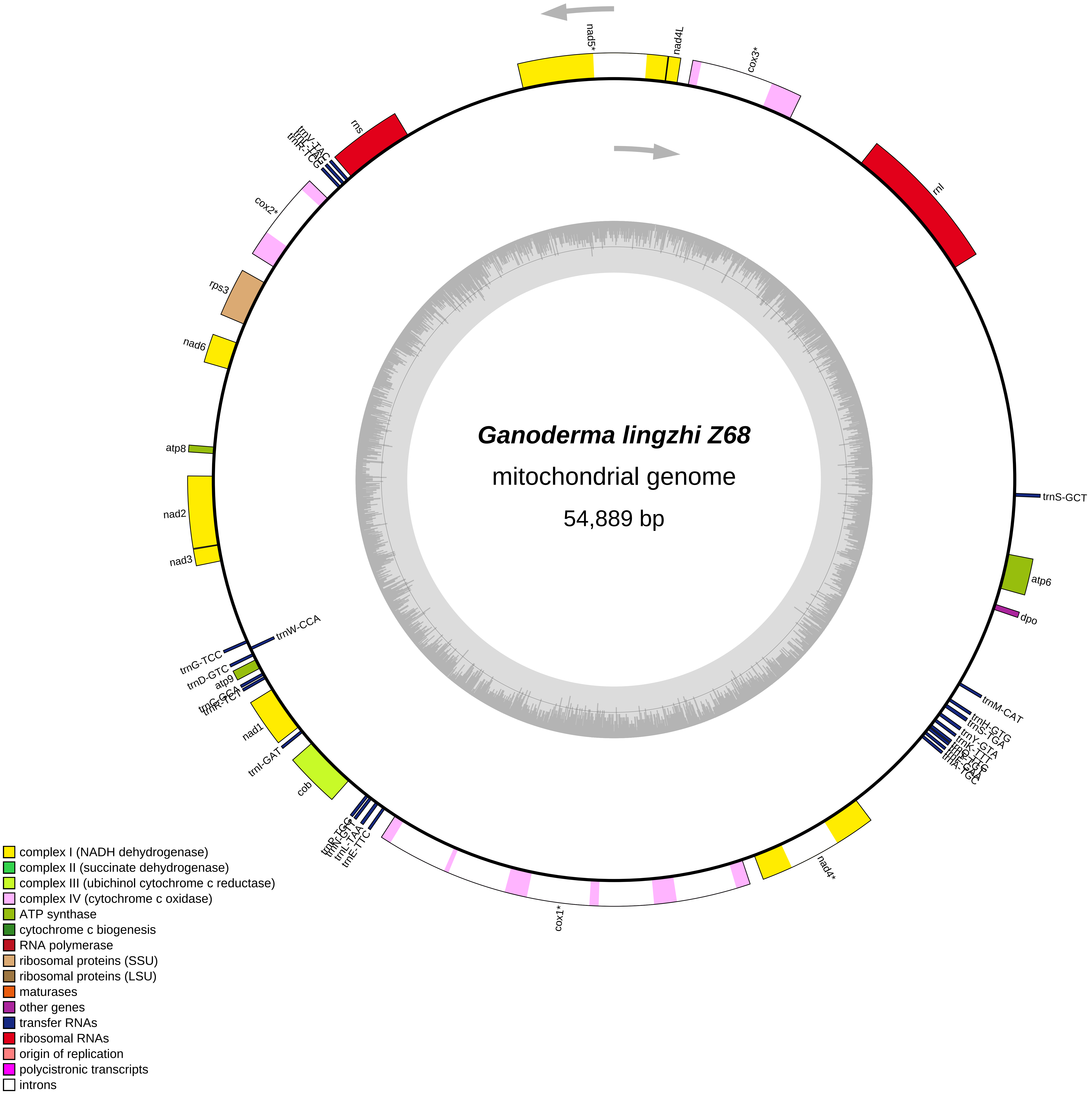

Ganoderma lingzhi Z68
mitochondrial genome
54,889 bp
nad5*
nad4L
cox3*
rnl
trnS-GCT
atp6
dpo
trnM-CAT
trnH-GTG
trnS-TGA
trnY-GTA
nad4*
cox1*
trnE-TTC
trnL-TAA
cob
trnI-GAT
nad1
trnR-TCT
trnC-GCA
atp9
trnD-GTC
trnG-TCC
trnW-CCA
nad3
nad2
atp8
nad6
rps3
cox2*
rns
trnV-TAC
complex I (NADH dehydrogenase)
complex II (succinate dehydrogenase)
complex III (ubichinol cytochrome c reductase)
complex IV (cytochrome c oxidase)
ATP synthase
cytochrome c biogenesis
RNA polymerase
ribosomal proteins (SSU)
ribosomal proteins (LSU)
maturases
other genes
transfer RNAs
ribosomal RNAs
origin of replication
polycistronic transcripts
introns

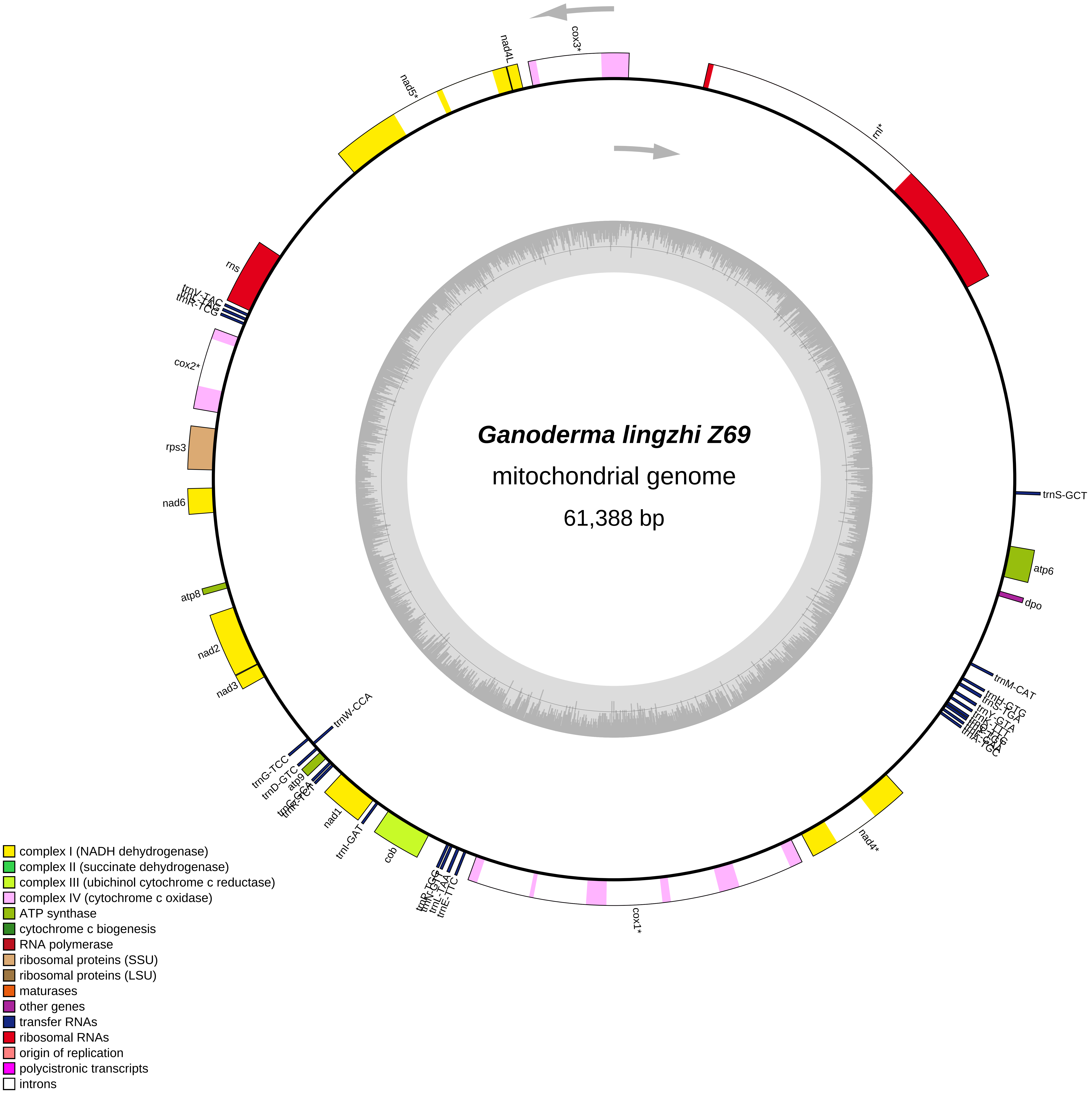

Ganoderma lingzhi Z69
mitochondrial genome
61,388 bp
cox3*
nad4L
nad5*
rnl*
rns
trnV-TAC
trnR-TCG
cox2*
rps3
nad6
atp8
nad2
nad3
trnW-CCA
trnG-TCC
trnD-GTC
atp9
nad1
trnI-GAT
cob
trnE-TTC
cox1*
nad4*
trnM-CAT
trnH-GTG
trnS-TGA
trnS-GCT
atp6
dpo
complex I (NADH dehydrogenase)
complex II (succinate dehydrogenase)
complex III (ubichinol cytochrome c reductase)
complex IV (cytochrome c oxidase)
ATP synthase
cytochrome c biogenesis
RNA polymerase
ribosomal proteins (SSU)
ribosomal proteins (LSU)
maturases
other genes
transfer RNAs
ribosomal RNAs
origin of replication
polycistronic transcripts
introns

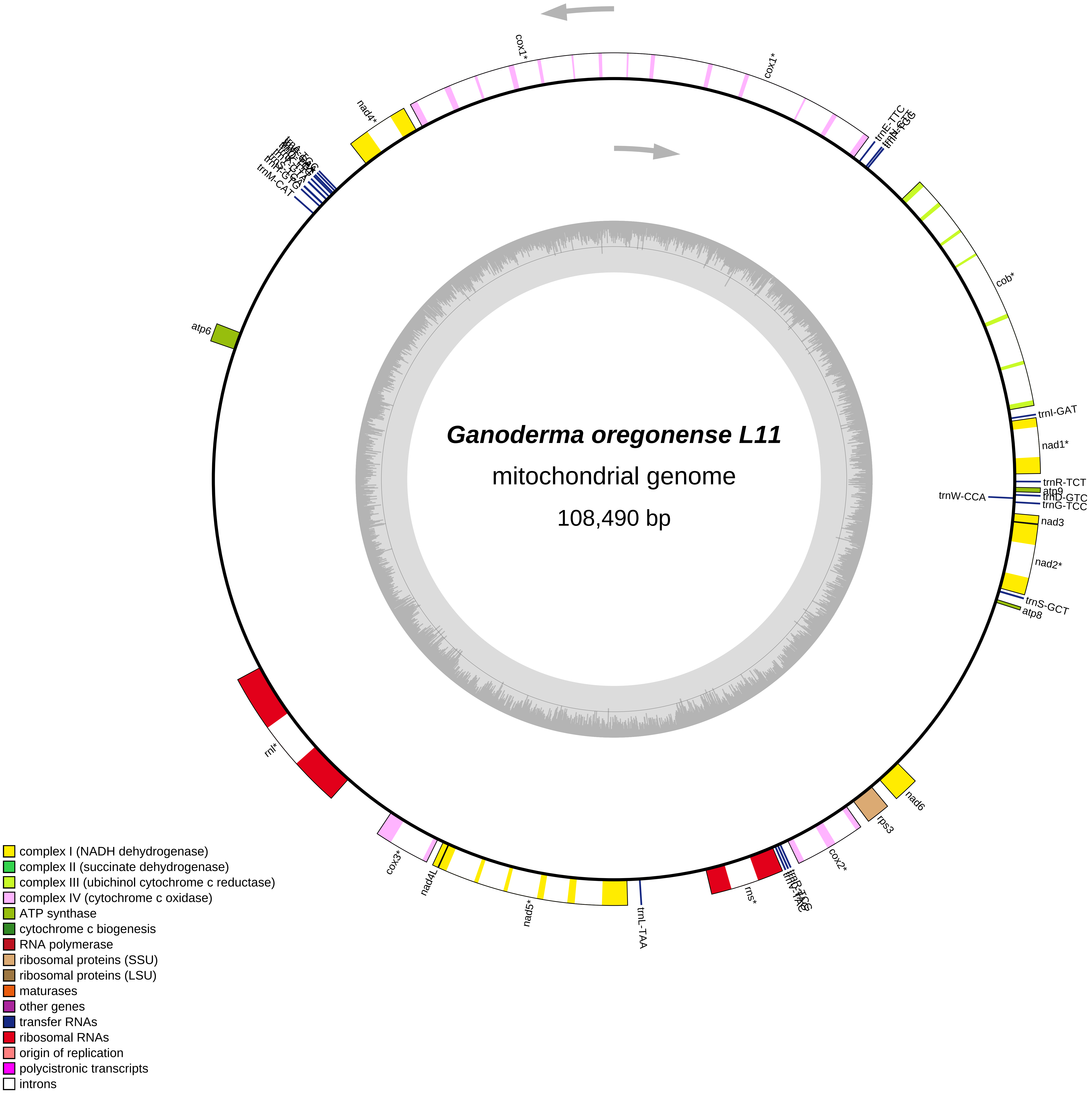

Ganoderma oregonense L11
mitochondrial genome
108,490 bp
cox1*
cox1*
nad4*
trnE-TTC
cob*
trnI-GAT
nad1*
trnR-TCT
atp9
trnW-CCA
trnD-GTC
trnG-TCC
nad3
nad2*
trnS-GCT
atp8
atp6
trnM-CAT
nad6
rps3
cox2*
rns*
trnL-TAA
nad5*
nad4L
cox3*
rnl*
complex I (NADH dehydrogenase)
complex II (succinate dehydrogenase)
complex III (ubichinol cytochrome c reductase)
complex IV (cytochrome c oxidase)
ATP synthase
cytochrome c biogenesis
RNA polymerase
ribosomal proteins (SSU)
ribosomal proteins (LSU)
maturases
other genes
transfer RNAs
ribosomal RNAs
origin of replication
polycistronic transcripts
introns

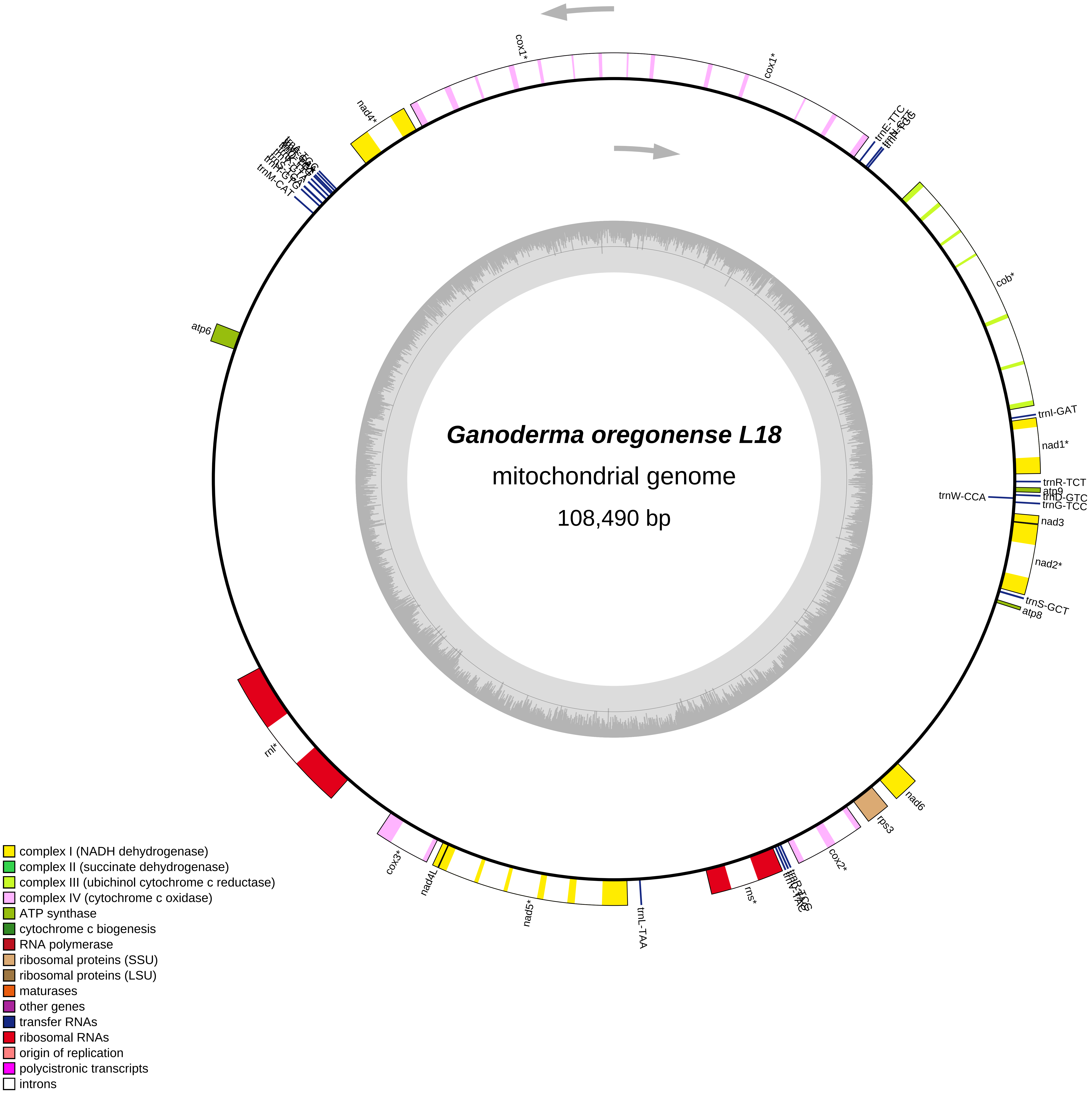
Ganoderma oregonense L18
mitochondrial genome
108,490 bp
cox1*
cox1*
trnE-TTC
nad4*
trnM-CAT
atp6
cob*
trnI-GAT
nad1*
trnR-TCT
atp9
trnW-CCA
trnD-GTC
trnG-TCC
nad3
nad2*
trnS-GCT
atp8
nad6
rps3
cox2*
rns*
trnL-TAA
nad5*
nad4L
cox3*
rnl*
complex I (NADH dehydrogenase)
complex II (succinate dehydrogenase)
complex III (ubichinol cytochrome c reductase)
complex IV (cytochrome c oxidase)
ATP synthase
cytochrome c biogenesis
RNA polymerase
ribosomal proteins (SSU)
ribosomal proteins (LSU)
maturases
other genes
transfer RNAs
ribosomal RNAs
origin of replication
polycistronic transcripts
introns

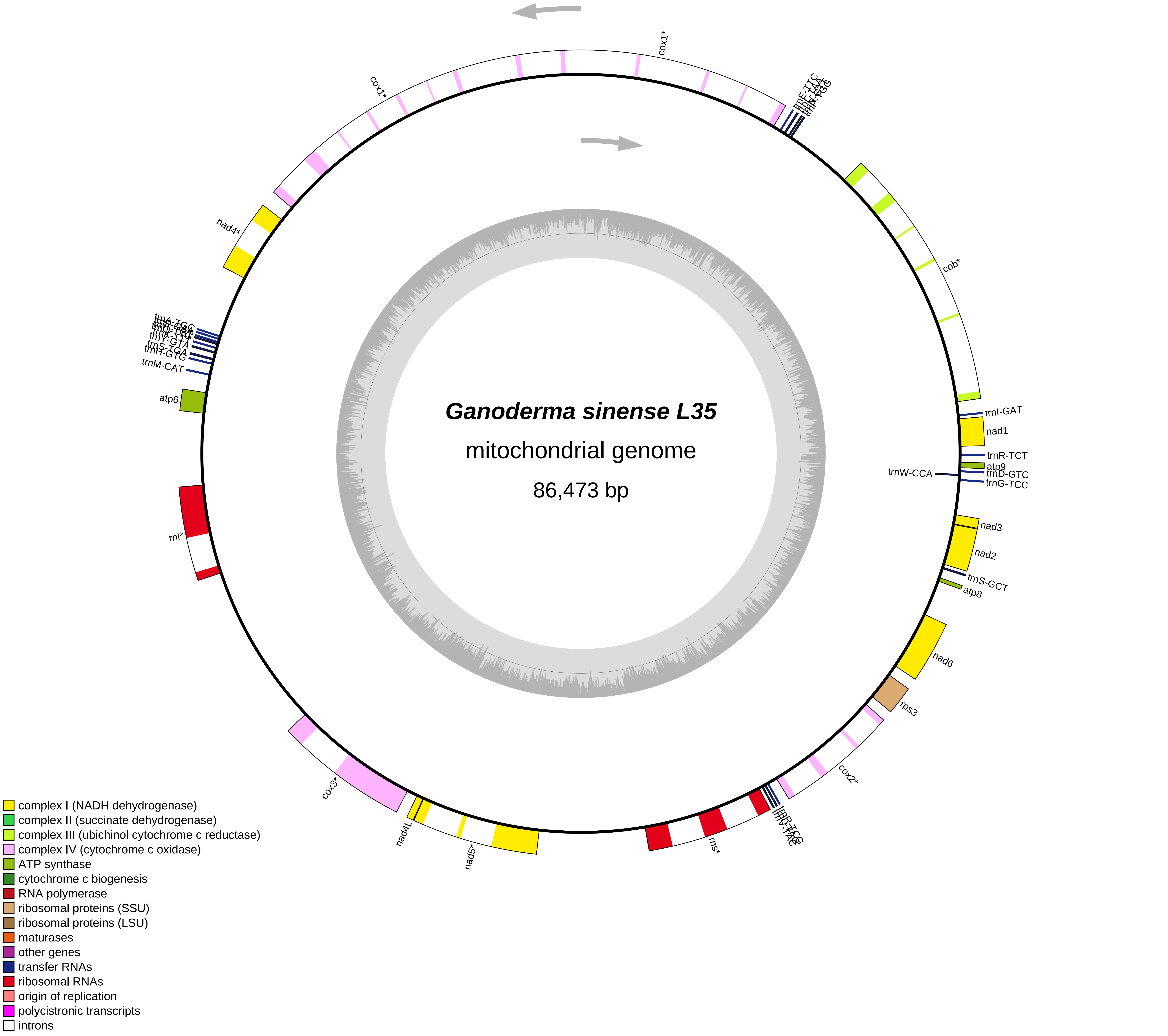

Ganoderma sinense L35
mitochondrial genome
86,473 bp
cox1*
cox1*
nad4*
trnM-CAT
atp6
rnl*
cox3*
nad4L
nad5*
rns*
cox2*
rps3
nad6
atp8
trnS-GCT
nad2
nad3
trnG-TCC
trnD-GTC
trnW-CCA
atp9
trnR-TCT
nad1
trnI-GAT
cob*
complex I (NADH dehydrogenase)
complex II (succinate dehydrogenase)
complex III (ubichinol cytochrome c reductase)
complex IV (cytochrome c oxidase)
ATP synthase
cytochrome c biogenesis
RNA polymerase
ribosomal proteins (SSU)
ribosomal proteins (LSU)
maturases
other genes
transfer RNAs
ribosomal RNAs
origin of replication
polycistronic transcripts
introns

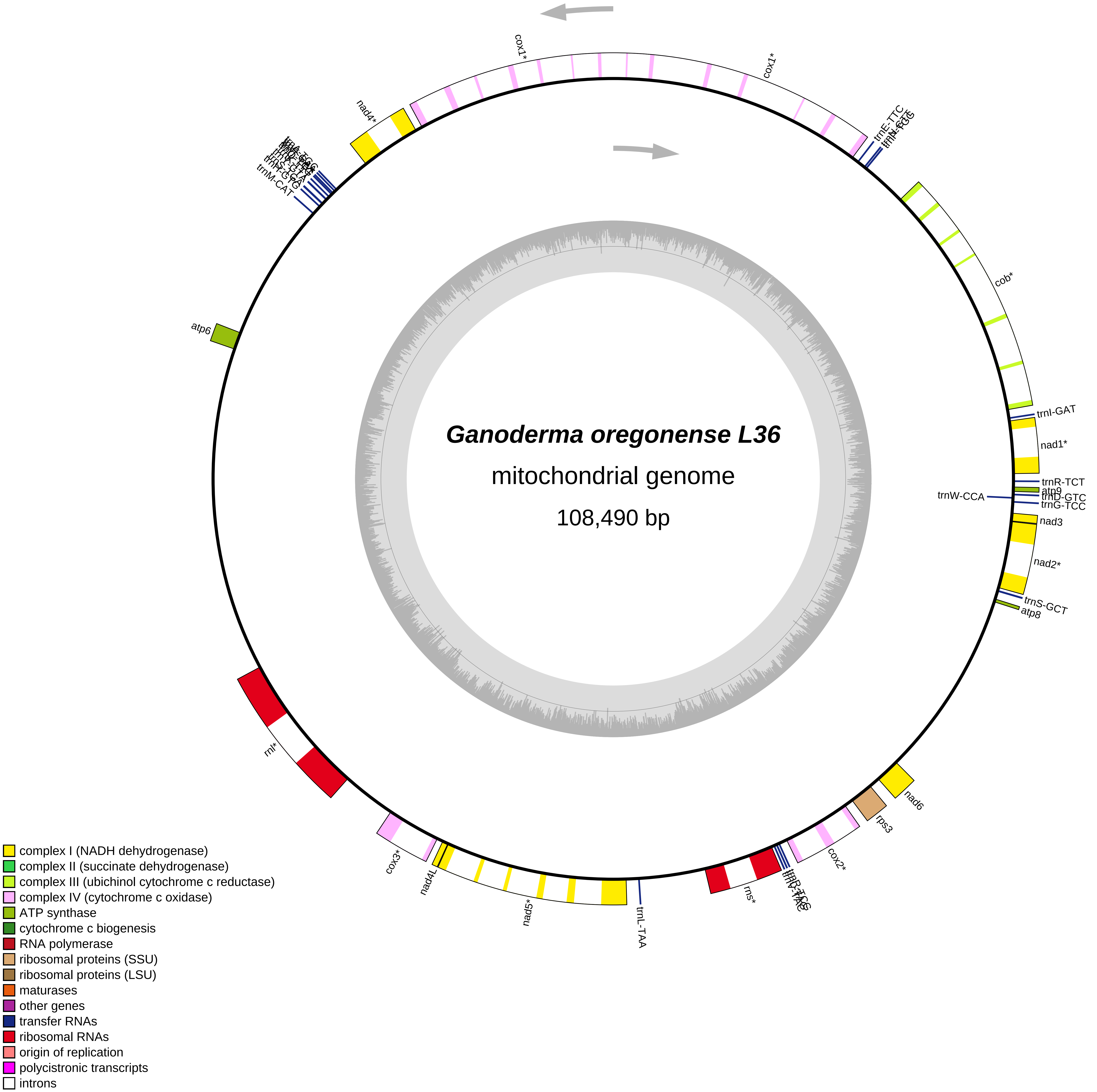
Ganoderma oregonense L36
mitochondrial genome
108,490 bp
cox1*
cox1*
trnE-TTC
nad4*
cob*
trnI-GAT
nad1*
trnR-TCT
atp9
trnW-CCA
trnG-TCC
nad3
nad2*
trnS-GCT
atp8
trnM-CAT
atp6
rnl*
cox3*
nad4L
nad5*
trnL-TAA
rns*
cox2*
rps3
nad6
complex I (NADH dehydrogenase)
complex II (succinate dehydrogenase)
complex III (ubichinol cytochrome c reductase)
complex IV (cytochrome c oxidase)
ATP synthase
cytochrome c biogenesis
RNA polymerase
ribosomal proteins (SSU)
ribosomal proteins (LSU)
maturases
other genes
transfer RNAs
ribosomal RNAs
origin of replication
polycistronic transcripts
introns

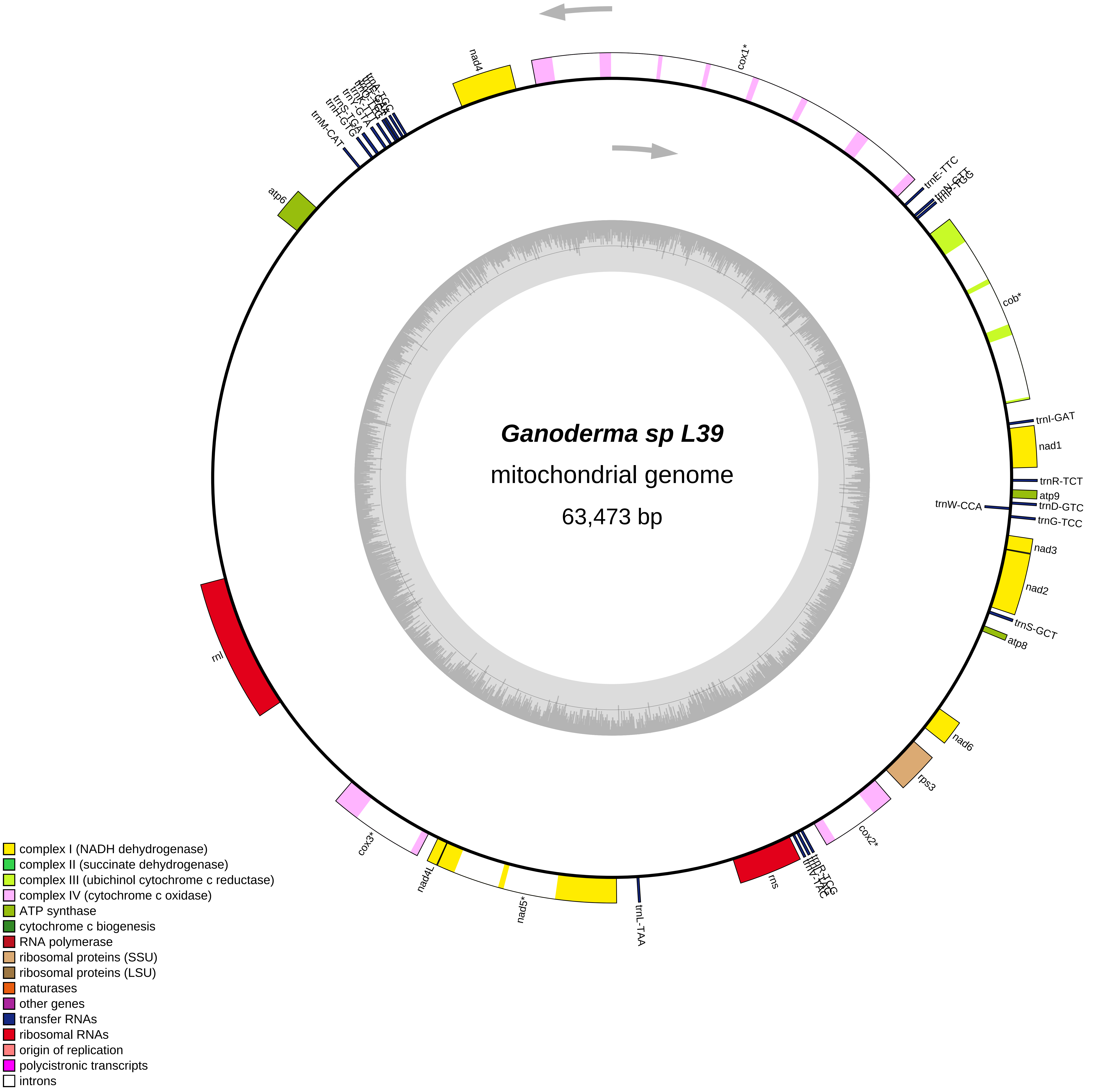
Ganoderma sp L39
mitochondrial genome
63,473 bp
nad4
cox1*
trnE-TTC
trnN-GTT
trnP-TGG
cob*
trnI-GAT
nad1
trnR-TCT
atp9
trnD-GTC
trnW-CCA
trnG-TCC
nad3
nad2
trnS-GCT
atp8
nad6
rps3
cox2*
rns
trnL-TAA
nad5*
nad4L
cox3*
rnl
atp6
trnM-CAT
trnH-GTG
trnS-TGA
complex I (NADH dehydrogenase)
complex II (succinate dehydrogenase)
complex III (ubichinol cytochrome c reductase)
complex IV (cytochrome c oxidase)
ATP synthase
cytochrome c biogenesis
RNA polymerase
ribosomal proteins (SSU)
ribosomal proteins (LSU)
maturases
other genes
transfer RNAs
ribosomal RNAs
origin of replication
polycistronic transcripts
introns

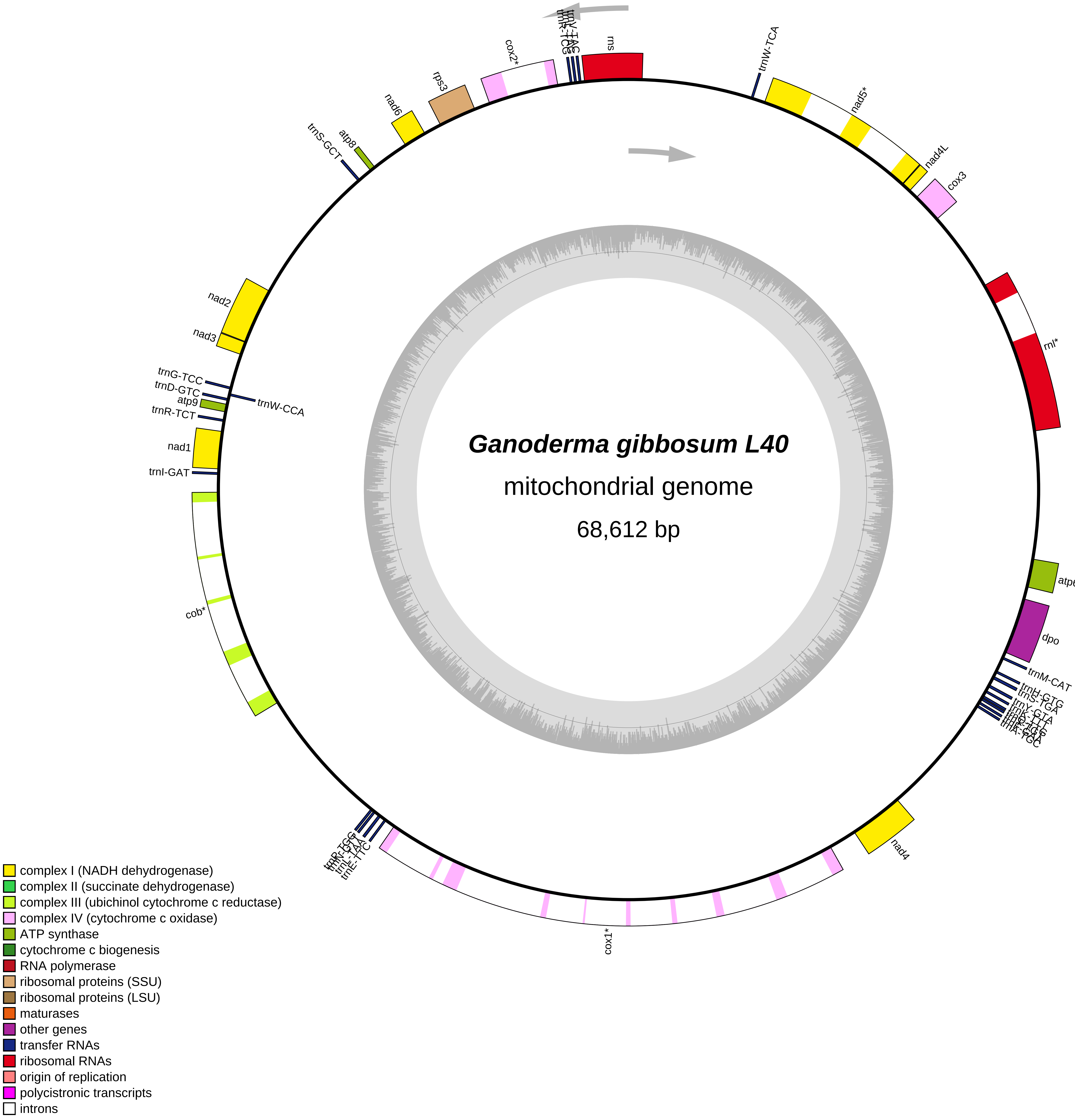

Ganoderma gibbosum L40
mitochondrial genome
68,612 bp
rns
trnW-TCA
nad5*
nad4L
cox3
rnl*
atp6
dpo
trnM-CAT
trnH-GTG
trnS-TGA
nad4
cox1*
trnE-TTC
cob*
trnI-GAT
nad1
trnR-TCT
atp9
trnD-GTC
trnG-TCC
trnW-CCA
nad3
nad2
trnS-GCT
atp8
nad6
rps3
cox2*
complex I (NADH dehydrogenase)
complex II (succinate dehydrogenase)
complex III (ubichinol cytochrome c reductase)
complex IV (cytochrome c oxidase)
ATP synthase
cytochrome c biogenesis
RNA polymerase
ribosomal proteins (SSU)
ribosomal proteins (LSU)
maturases
other genes
transfer RNAs
ribosomal RNAs
origin of replication
polycistronic transcripts
introns

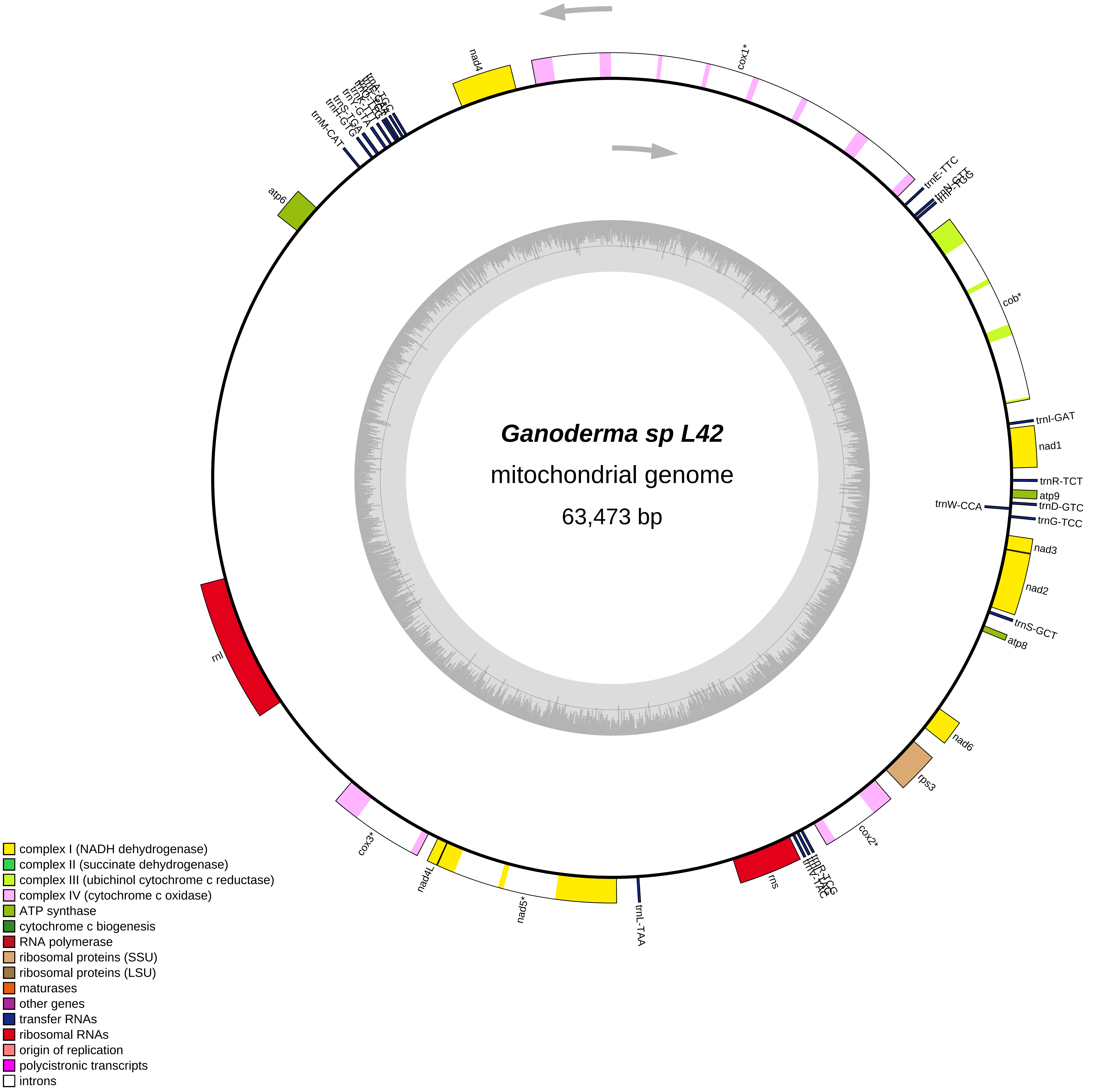

Ganoderma sp L42
mitochondrial genome
63,473 bp
nad4
cox1*
trnE-TTC
cob*
trnI-GAT
nad1
trnR-TCT
atp9
trnD-GTC
trnW-CCA
trnG-TCC
nad3
nad2
trnS-GCT
atp8
nad6
rps3
cox2*
rns
trnL-TAA
nad5*
nad4L
cox3*
rnl
atp6
trnM-CAT
trnH-GTG
trnS-TGA
complex I (NADH dehydrogenase)
complex II (succinate dehydrogenase)
complex III (ubichinol cytochrome c reductase)
complex IV (cytochrome c oxidase)
ATP synthase
cytochrome c biogenesis
RNA polymerase
ribosomal proteins (SSU)
ribosomal proteins (LSU)
maturases
other genes
transfer RNAs
ribosomal RNAs
origin of replication
polycistronic transcripts
introns

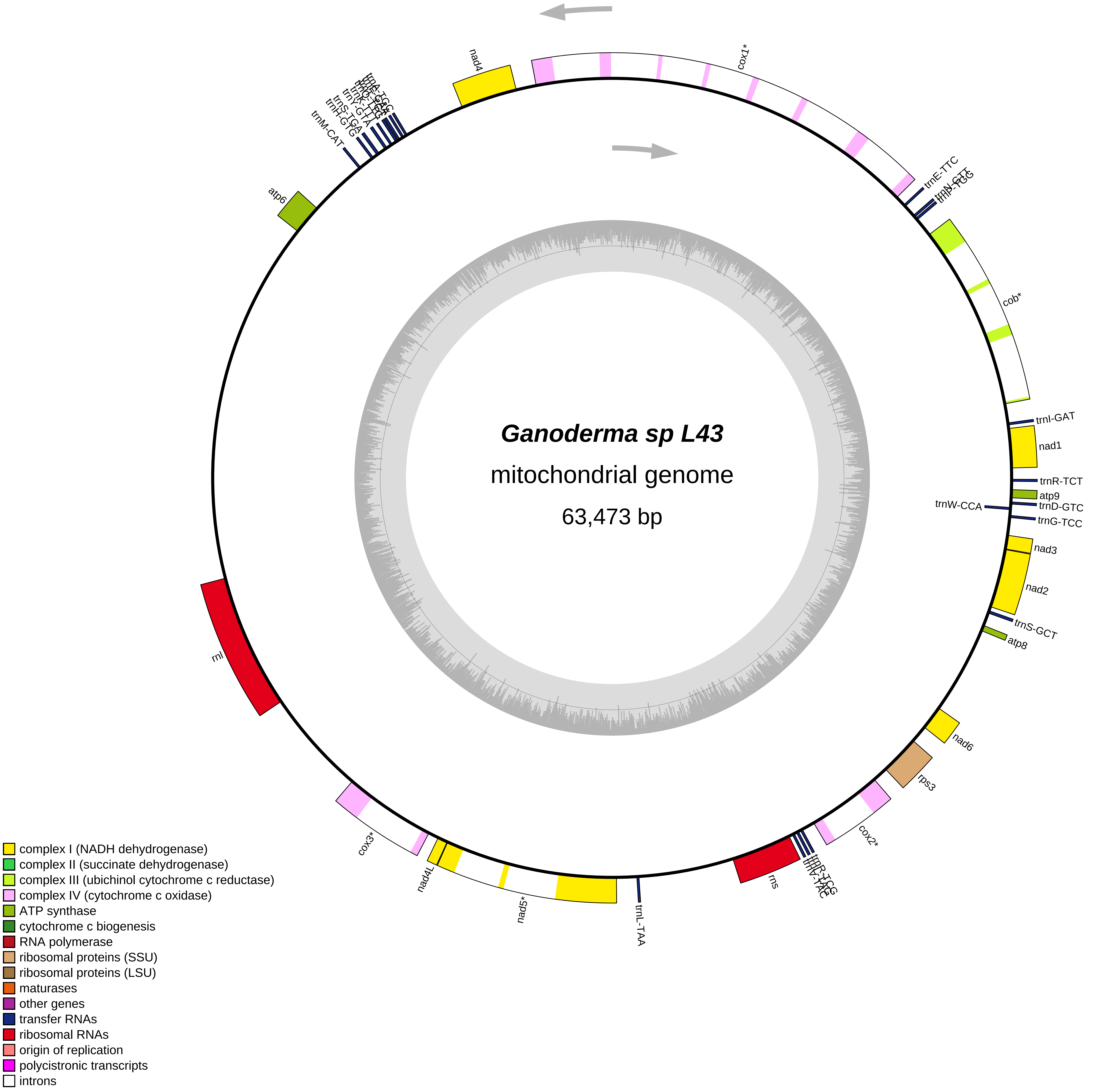

Ganoderma sp L43
mitochondrial genome
63,473 bp
cox1*
nad4
trnM-CAT
atp6
trnE-TTC
cob*
trnI-GAT
nad1
trnR-TCT
atp9
trnD-GTC
trnW-CCA
trnG-TCC
nad3
nad2
trnS-GCT
atp8
nad6
rps3
cox2*
rns
trnL-TAA
nad5*
nad4L
cox3*
rnl
complex I (NADH dehydrogenase)
complex II (succinate dehydrogenase)
complex III (ubichinol cytochrome c reductase)
complex IV (cytochrome c oxidase)
ATP synthase
cytochrome c biogenesis
RNA polymerase
ribosomal proteins (SSU)
ribosomal proteins (LSU)
maturases
other genes
transfer RNAs
ribosomal RNAs
origin of replication
polycistronic transcripts
introns

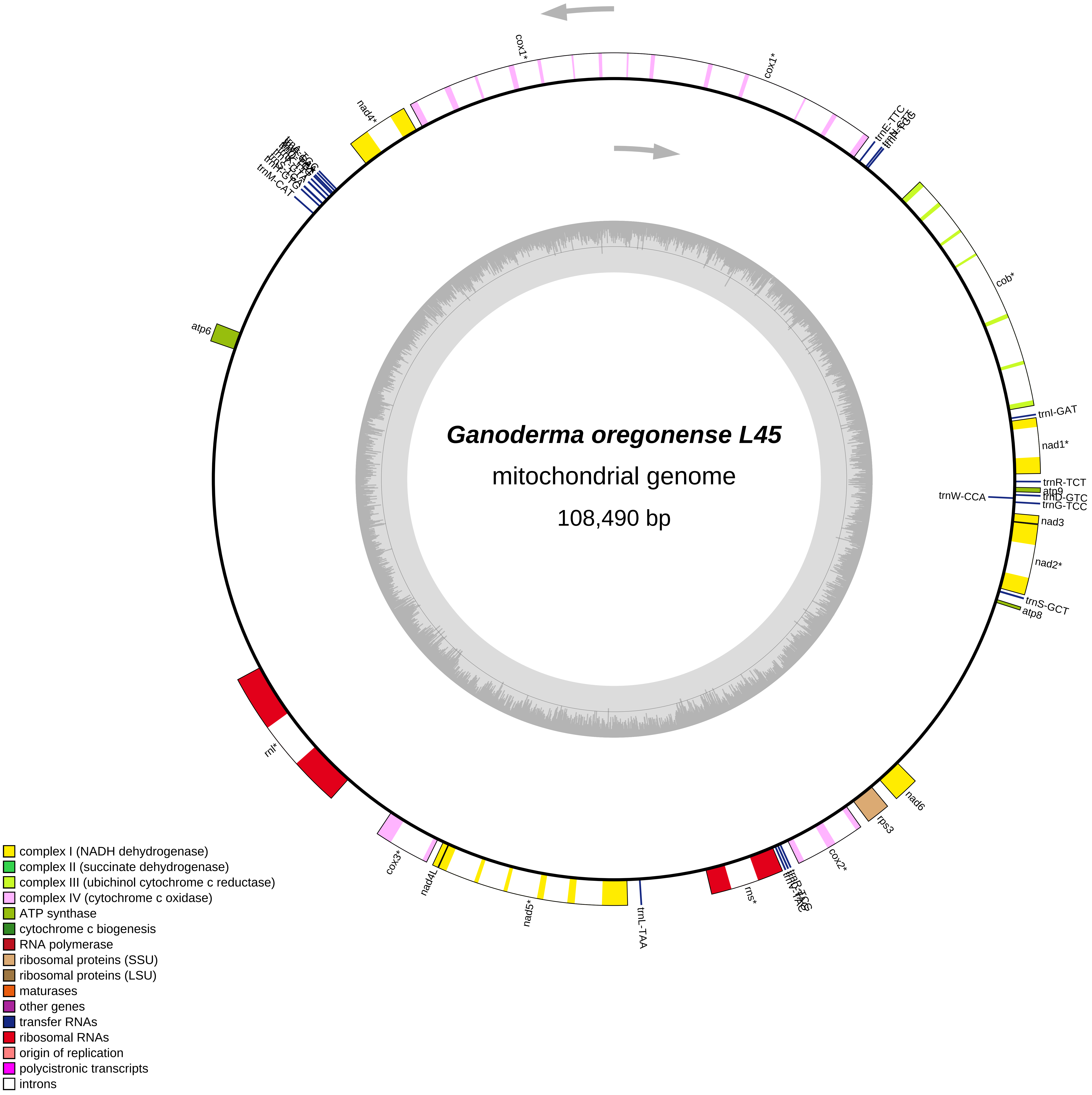

Ganoderma oregonense L45
mitochondrial genome
108,490 bp
cox1*
cox1*
nad4*
trnE-TTC
atp6
cob*
trnI-GAT
nad1*
trnR-TCT
atp9
trnW-CCA
trnD-GTC
trnG-TCC
nad3
nad2*
trnS-GCT
atp8
trnM-CAT
rnl*
nad6
rps3
cox2*
rns*
cox3*
nad4L
nad5*
trnL-TAA
complex I (NADH dehydrogenase)
complex II (succinate dehydrogenase)
complex III (ubichinol cytochrome c reductase)
complex IV (cytochrome c oxidase)
ATP synthase
cytochrome c biogenesis
RNA polymerase
ribosomal proteins (SSU)
ribosomal proteins (LSU)
maturases
other genes
transfer RNAs
ribosomal RNAs
origin of replication
polycistronic transcripts
introns

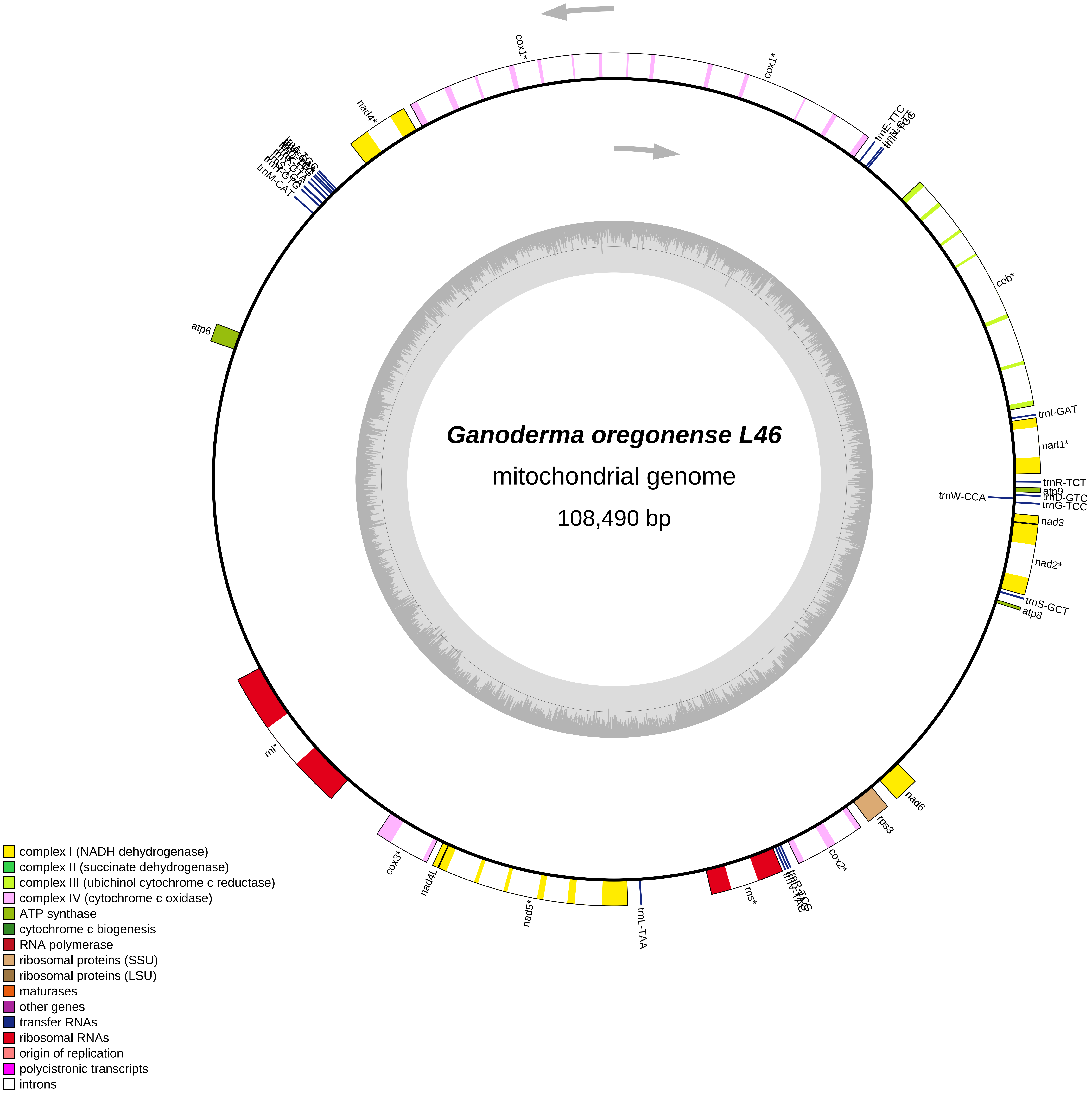

Ganoderma oregonense L46
mitochondrial genome
108,490 bp
cox1*
cox1*
trnE-TTC
nad4*
trnM-CAT
atp6
cob*
trnI-GAT
nad1*
trnR-TCT
atp9
trnW-CCA
trnG-TCC
nad3
nad2*
trnS-GCT
atp8
nad6
rps3
cox2*
rns*
trnL-TAA
nad5*
nad4L
cox3*
rnl*
complex I (NADH dehydrogenase)
complex II (succinate dehydrogenase)
complex III (ubichinol cytochrome c reductase)
complex IV (cytochrome c oxidase)
ATP synthase
cytochrome c biogenesis
RNA polymerase
ribosomal proteins (SSU)
ribosomal proteins (LSU)
maturases
other genes
transfer RNAs
ribosomal RNAs
origin of replication
polycistronic transcripts
introns

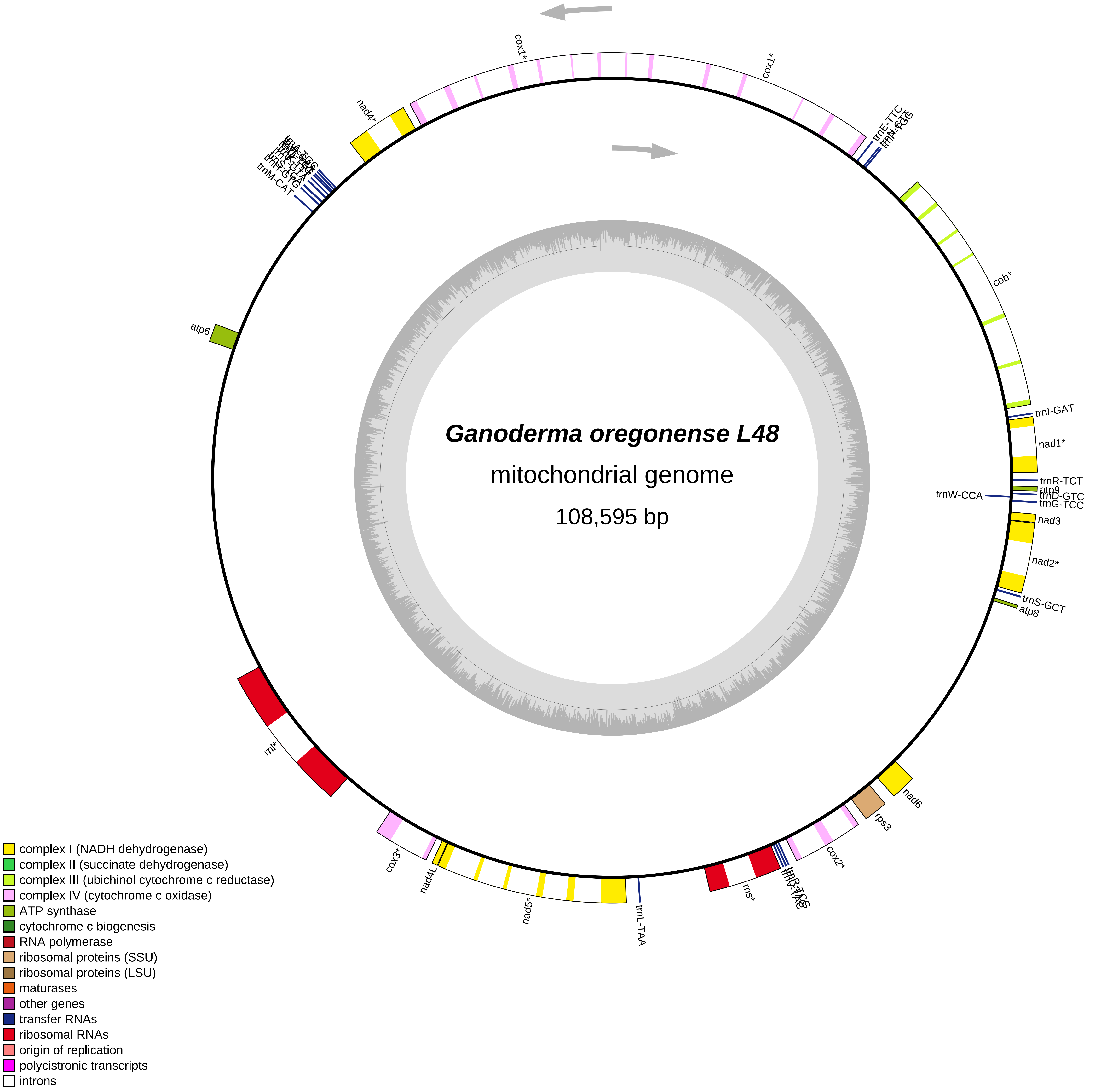

Ganoderma oregonense L48
mitochondrial genome
108,595 bp
cox1*
cox1*
nad4*
trnE-TTC
cob*
trnI-GAT
nad1*
trnR-TCT
atp9
trnD-GTC
trnG-TCC
trnW-CCA
nad3
nad2*
trnS-GCT
atp8
nad6
rps3
cox2*
rns*
trnL-TAA
nad5*
nad4L
cox3*
rnl*
atp6
trnM-CAT
complex I (NADH dehydrogenase)
complex II (succinate dehydrogenase)
complex III (ubichinol cytochrome c reductase)
complex IV (cytochrome c oxidase)
ATP synthase
cytochrome c biogenesis
RNA polymerase
ribosomal proteins (SSU)
ribosomal proteins (LSU)
maturases
other genes
transfer RNAs
ribosomal RNAs
origin of replication
polycistronic transcripts
introns

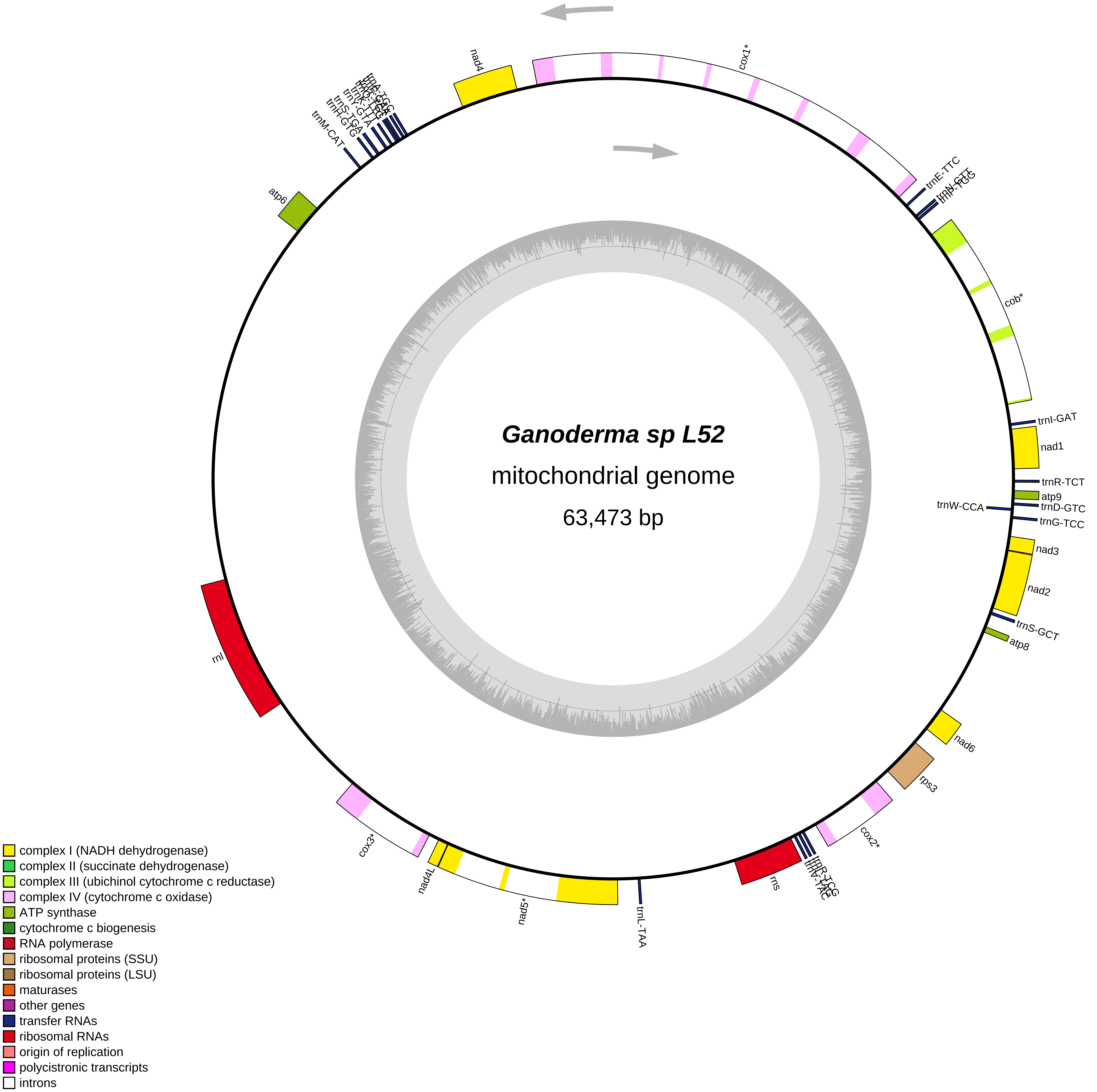
Ganoderma sp L52
mitochondrial genome
63,473 bp
cox1*
nad4
trnM-CAT
trnH-GTG
trnS-TGA
atp6
trnE-TTC
cob*
trnI-GAT
nad1
trnR-TCT
atp9
trnD-GTC
trnW-CCA
trnG-TCC
nad3
nad2
trnS-GCT
atp8
nad6
rps3
cox2*
rns
trnL-TAA
nad5*
nad4L
cox3*
rnl
complex I (NADH dehydrogenase)
complex II (succinate dehydrogenase)
complex III (ubichinol cytochrome c reductase)
complex IV (cytochrome c oxidase)
ATP synthase
cytochrome c biogenesis
RNA polymerase
ribosomal proteins (SSU)
ribosomal proteins (LSU)
maturases
other genes
transfer RNAs
ribosomal RNAs
origin of replication
polycistronic transcripts
introns

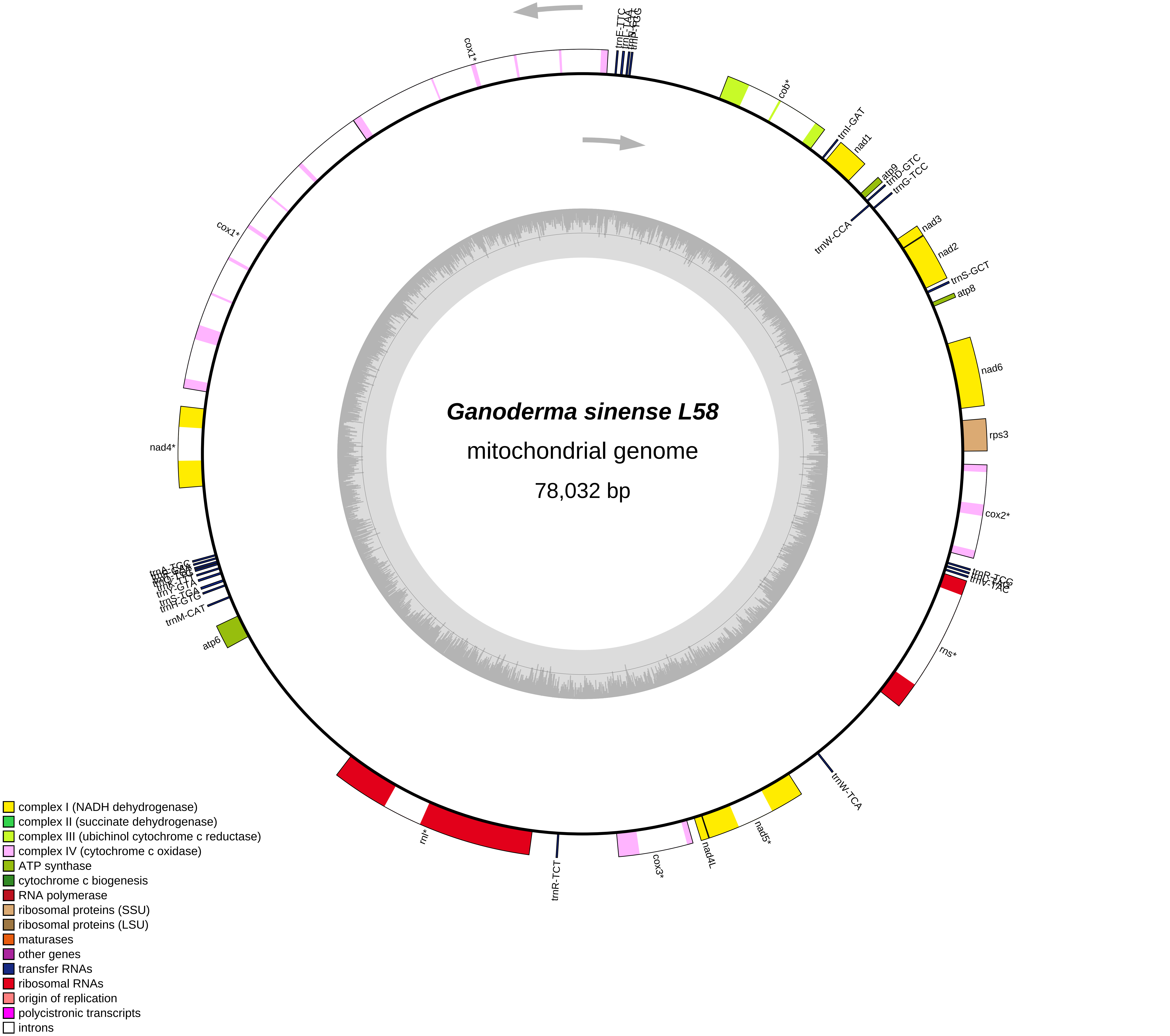
Ganoderma sinense L58
mitochondrial genome
78,032 bp
trnE-TTC
cob*
trnI-GAT
nad1
atp9
trnD-GTC
trnG-TCC
trnW-CCA
nad3
nad2
trnS-GCT
atp8
nad6
rps3
cox2*
trnR-TCG
trnV-TAC
rns*
trnW-TCA
nad5*
nad4L
cox3*
trnR-TCT
rnl*
atp6
trnM-CAT
trnH-GTG
trnS-TGA
trnY-GTA
trnA-TGC
nad4*
cox1*
cox1*
complex I (NADH dehydrogenase)
complex II (succinate dehydrogenase)
complex III (ubichinol cytochrome c reductase)
complex IV (cytochrome c oxidase)
ATP synthase
cytochrome c biogenesis
RNA polymerase
ribosomal proteins (SSU)
ribosomal proteins (LSU)
maturases
other genes
transfer RNAs
ribosomal RNAs
origin of replication
polycistronic transcripts
introns

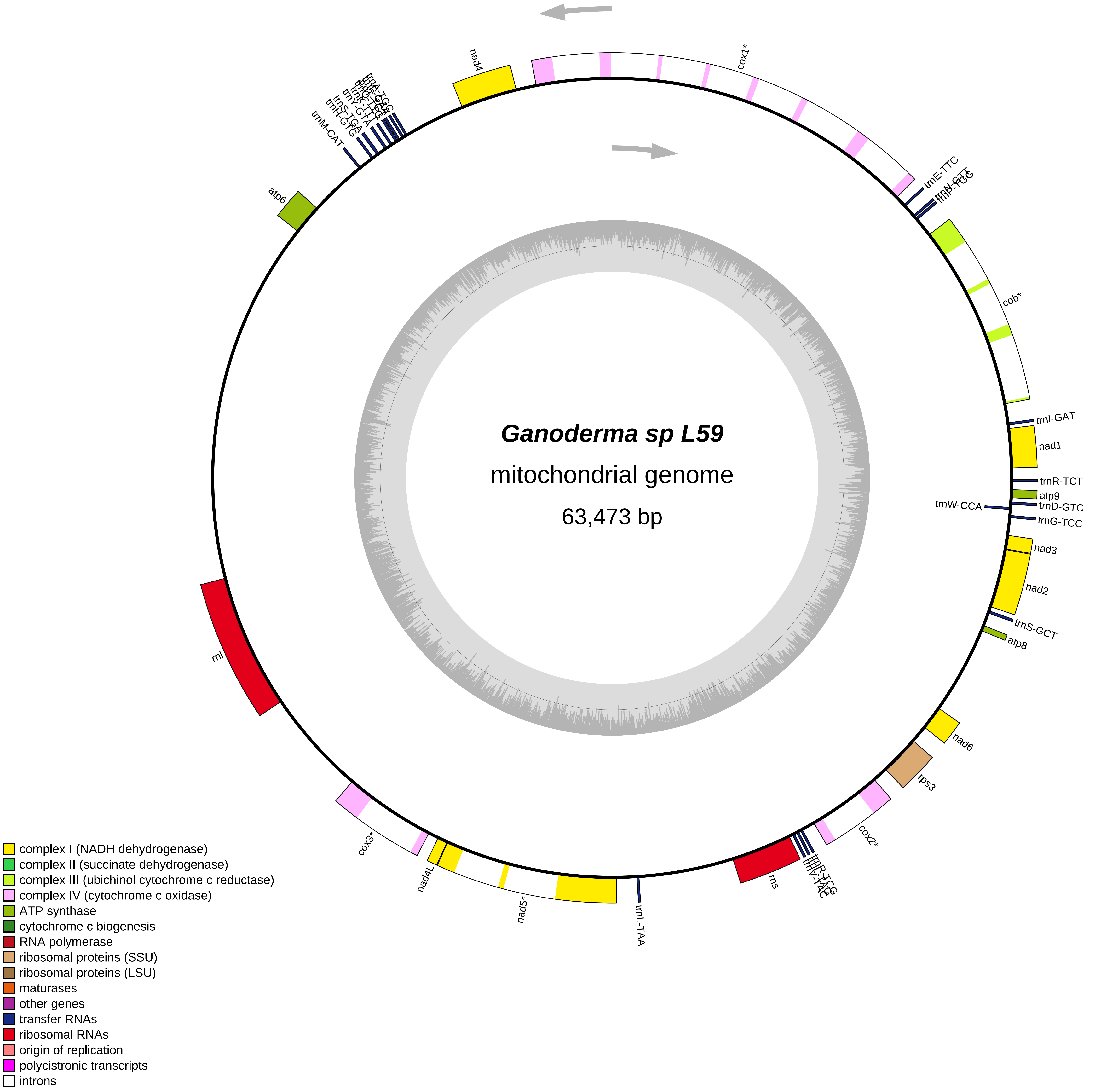
Ganoderma sp L59
mitochondrial genome
63,473 bp
cox1*
nad4
trnM-CAT
atp6
trnE-TTC
cob*
trnI-GAT
nad1
trnR-TCT
atp9
trnW-CCA
trnD-GTC
trnG-TCC
nad3
nad2
trnS-GCT
atp8
nad6
rps3
cox2*
rns
trnL-TAA
nad5*
nad4L
cox3*
rnl
complex I (NADH dehydrogenase)
complex II (succinate dehydrogenase)
complex III (ubichinol cytochrome c reductase)
complex IV (cytochrome c oxidase)
ATP synthase
cytochrome c biogenesis
RNA polymerase
ribosomal proteins (SSU)
ribosomal proteins (LSU)
maturases
other genes
transfer RNAs
ribosomal RNAs
origin of replication
polycistronic transcripts
introns

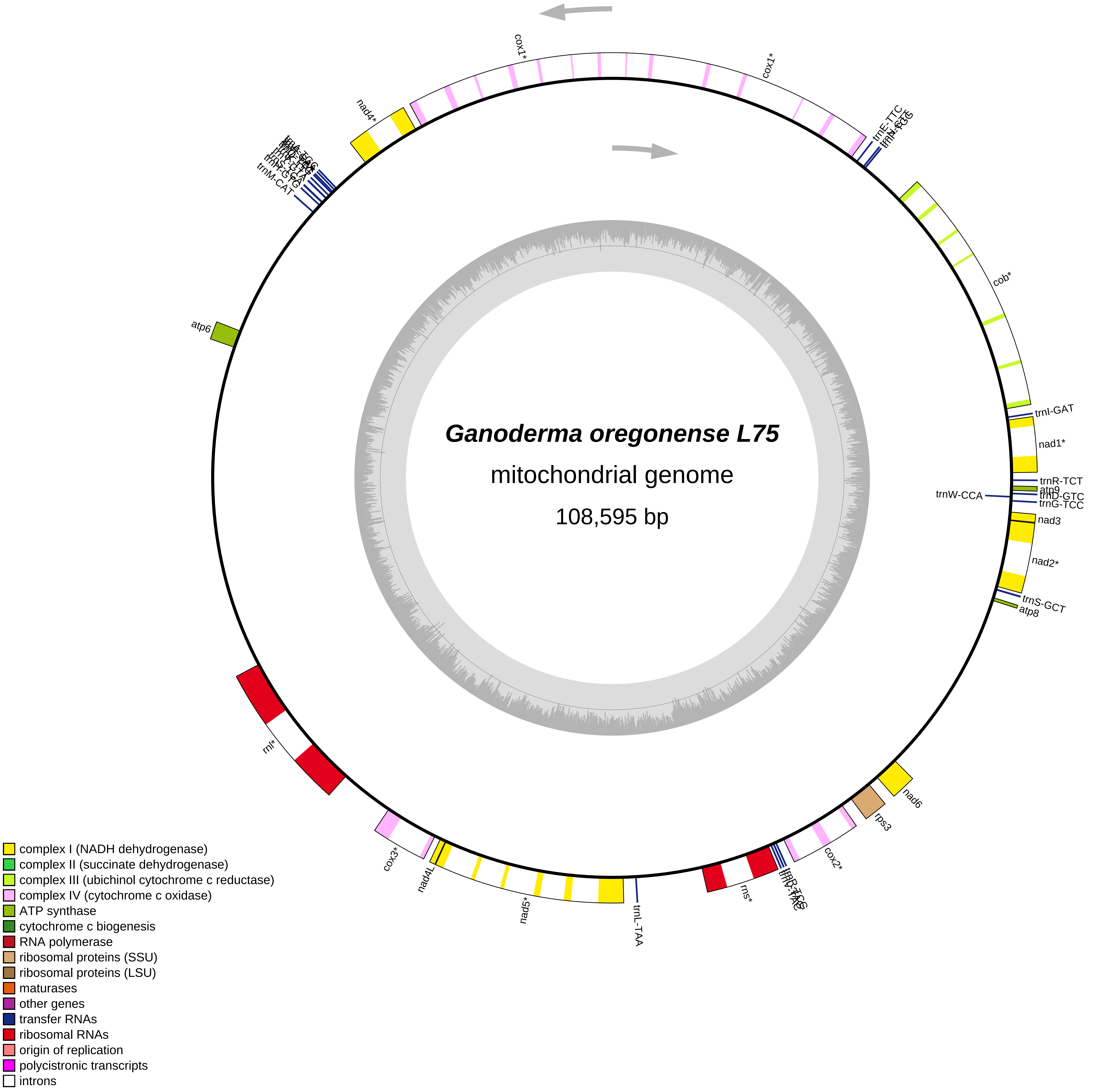
Ganoderma oregonense L75
mitochondrial genome
108,595 bp
cox1*
cox1*
nad4*
trnE-TTC
trnM-CAT
cob*
trnI-GAT
nad1*
trnR-TCT
atp9
trnW-CCA
trnD-GTC
trnG-TCC
nad3
nad2*
trnS-GCT
atp8
atp6
rnl*
nad6
rps3
cox2*
rns*
trnL-TAA
nad5*
nad4L
cox3*
complex I (NADH dehydrogenase)
complex II (succinate dehydrogenase)
complex III (ubichinol cytochrome c reductase)
complex IV (cytochrome c oxidase)
ATP synthase
cytochrome c biogenesis
RNA polymerase
ribosomal proteins (SSU)
ribosomal proteins (LSU)
maturases
other genes
transfer RNAs
ribosomal RNAs
origin of replication
polycistronic transcripts
introns

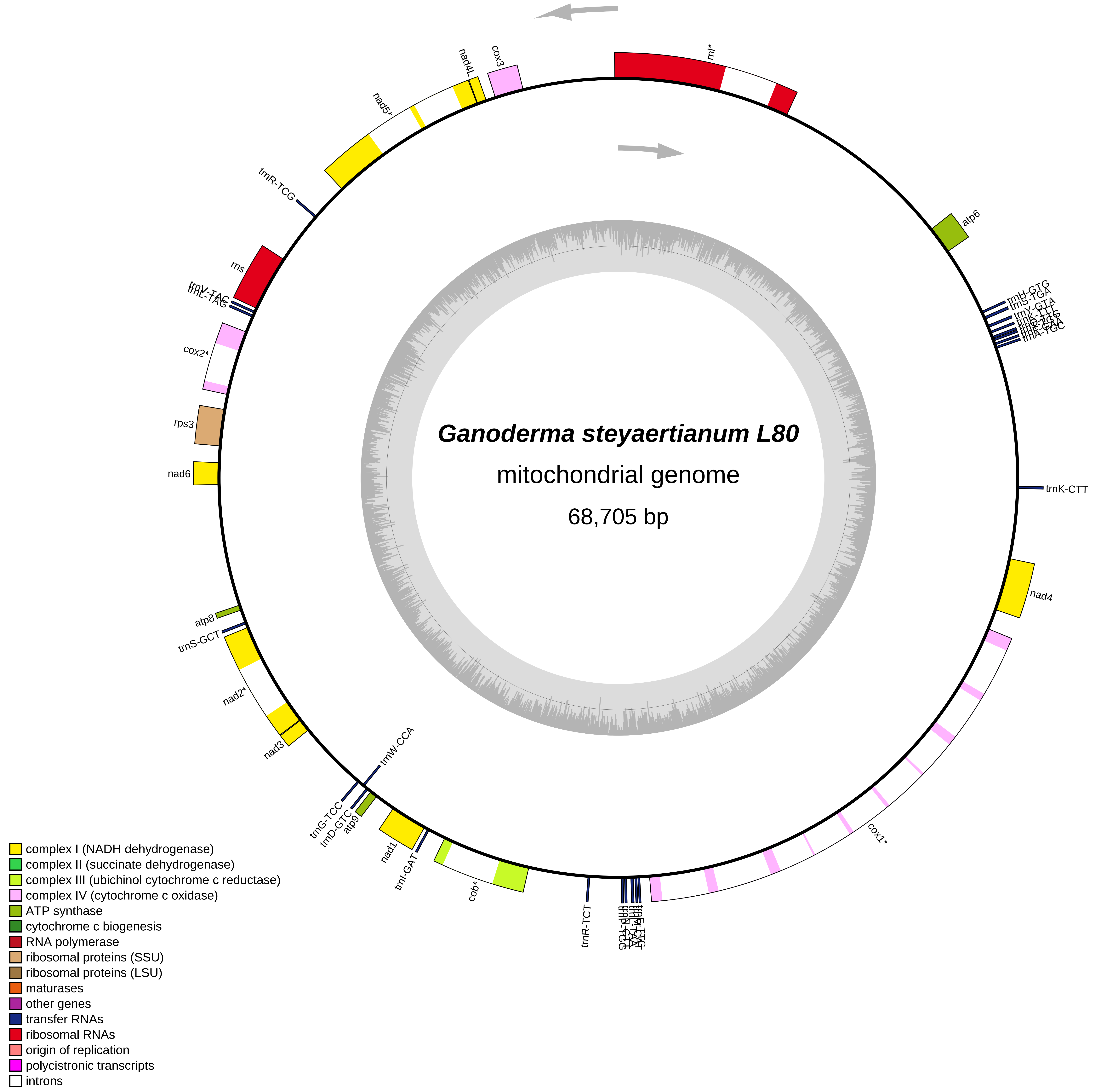
Ganoderma steyaertianum L80
mitochondrial genome
68,705 bp
rnl*
atp6
trnH-GTG
trnS-TGA
trnY-GTA
trnA-TGC
trnK-CTT
nad4
cox1*
trnR-TCT
cob*
trnI-GAT
nad1
atp9
trnD-GTC
trnG-TCC
trnW-CCA
nad3
nad2*
trnS-GCT
atp8
nad6
rps3
cox2*
trnV-TAC
trnL-TAG
rns
trnR-TCG
nad5*
nad4L
cox3
complex I (NADH dehydrogenase)
complex II (succinate dehydrogenase)
complex III (ubichinol cytochrome c reductase)
complex IV (cytochrome c oxidase)
ATP synthase
cytochrome c biogenesis
RNA polymerase
ribosomal proteins (SSU)
ribosomal proteins (LSU)
maturases
other genes
transfer RNAs
ribosomal RNAs
origin of replication
polycistronic transcripts
introns

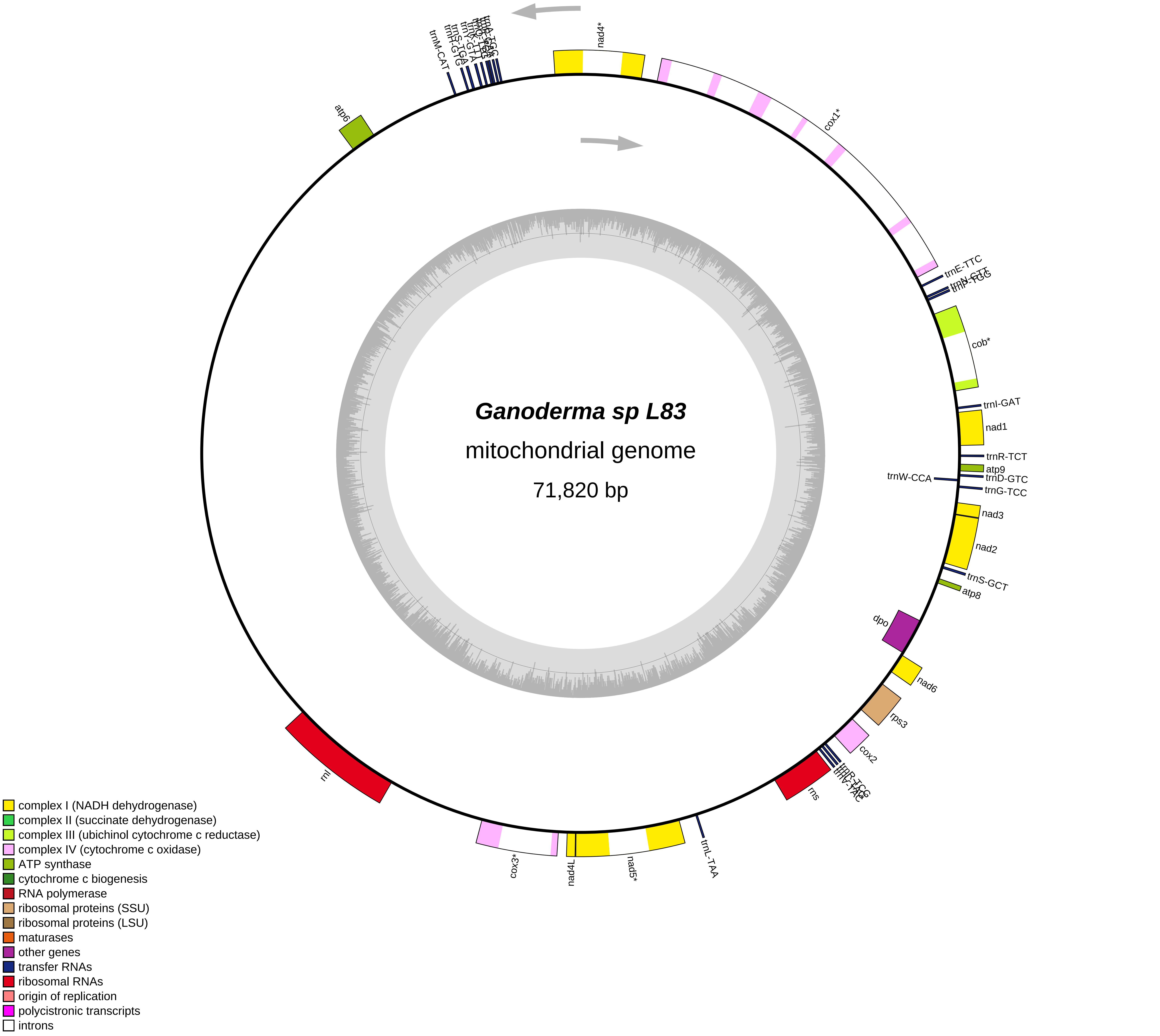
Ganoderma sp L83
mitochondrial genome
71,820 bp
nad4*
cox1*
trnE-TTC
cob*
trnI-GAT
nad1
trnR-TCT
atp9
trnD-GTC
trnW-CCA
trnG-TCC
nad3
nad2
trnS-GCT
atp8
dpo
nad6
rps3
cox2
rns
trnL-TAA
nad5*
nad4L
cox3*
rnl
atp6
trnM-CAT
complex I (NADH dehydrogenase)
complex II (succinate dehydrogenase)
complex III (ubichinol cytochrome c reductase)
complex IV (cytochrome c oxidase)
ATP synthase
cytochrome c biogenesis
RNA polymerase
ribosomal proteins (SSU)
ribosomal proteins (LSU)
maturases
other genes
transfer RNAs
ribosomal RNAs
origin of replication
polycistronic transcripts
introns

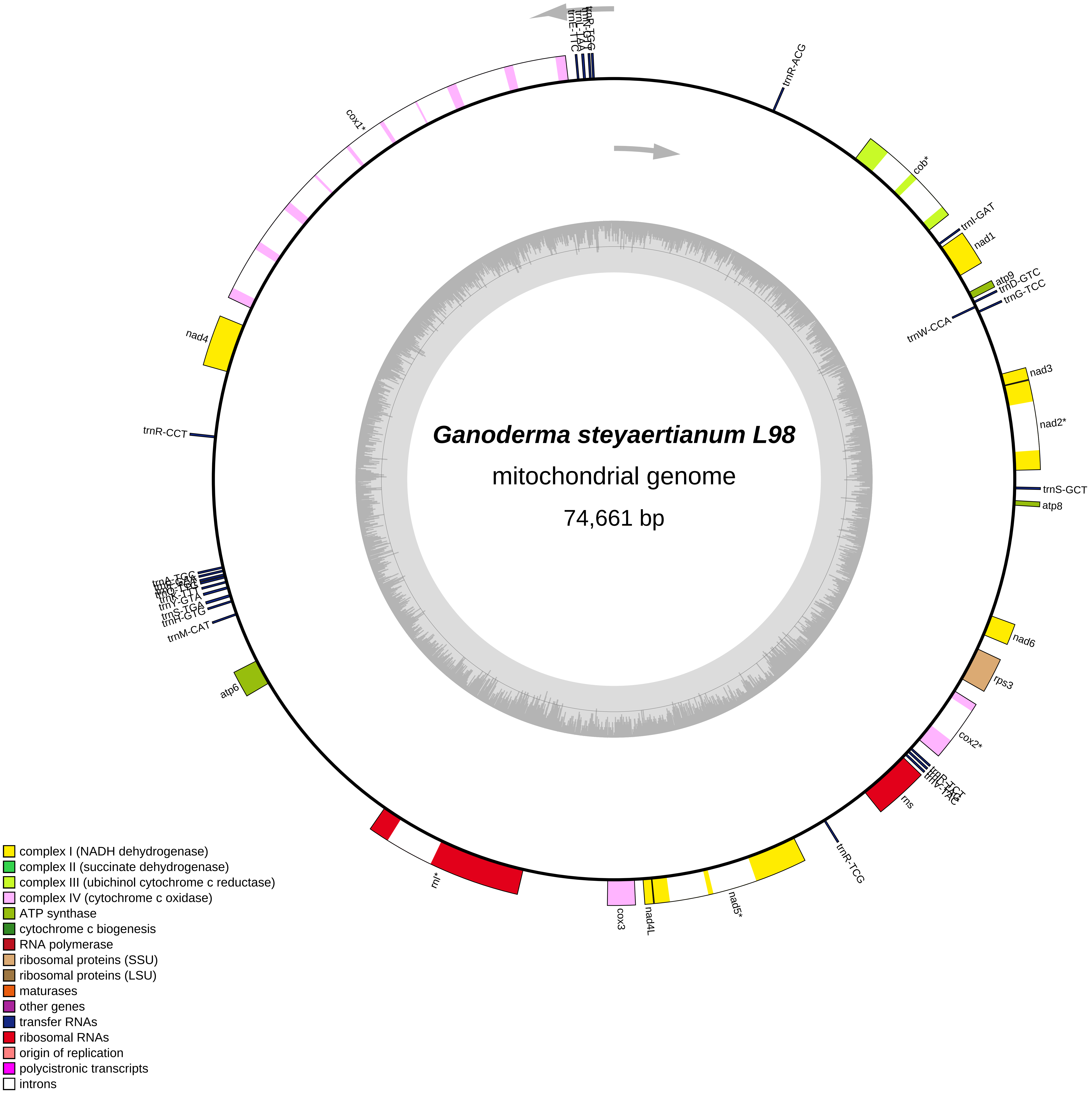
Ganoderma steyaertianum L98
mitochondrial genome
74,661 bp
trnR-ACG
cob*
trnI-GAT
nad1
atp9
trnD-GTC
trnG-TCC
trnW-CCA
nad3
nad2*
trnS-GCT
atp8
nad6
rps3
cox2*
rns
trnR-TCG
nad5*
nad4L
cox3
rnl*
atp6
trnM-CAT
trnH-GTG
trnR-CCT
nad4
cox1*
complex I (NADH dehydrogenase)
complex II (succinate dehydrogenase)
complex III (ubichinol cytochrome c reductase)
complex IV (cytochrome c oxidase)
ATP synthase
cytochrome c biogenesis
RNA polymerase
ribosomal proteins (SSU)
ribosomal proteins (LSU)
maturases
other genes
transfer RNAs
ribosomal RNAs
origin of replication
polycistronic transcripts
introns

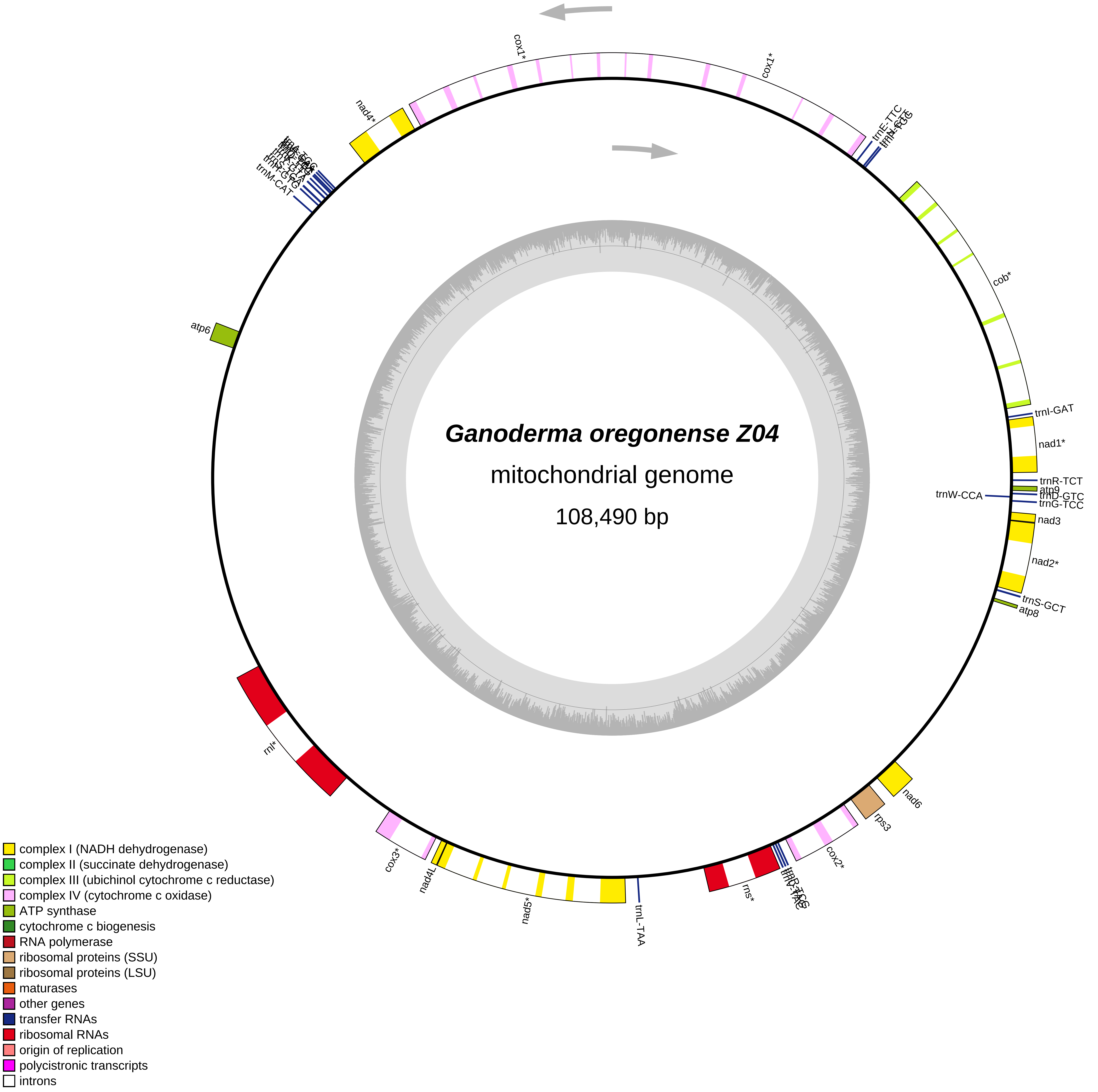
Ganoderma oregonense Z04
mitochondrial genome
108,490 bp
cox1*
cox1*
nad4*
trnE-TTC
trnI-GAT
cob*
nad1*
trnR-TCT
atp9
trnD-GTC
trnG-TCC
trnW-CCA
nad3
nad2*
trnS-GCT
atp8
atp6
trnM-CAT
nad6
rps3
cox2*
rns*
trnL-TAA
nad5*
nad4L
cox3*
rnl*
complex I (NADH dehydrogenase)
complex II (succinate dehydrogenase)
complex III (ubichinol cytochrome c reductase)
complex IV (cytochrome c oxidase)
ATP synthase
cytochrome c biogenesis
RNA polymerase
ribosomal proteins (SSU)
ribosomal proteins (LSU)
maturases
other genes
transfer RNAs
ribosomal RNAs
origin of replication
polycistronic transcripts
introns

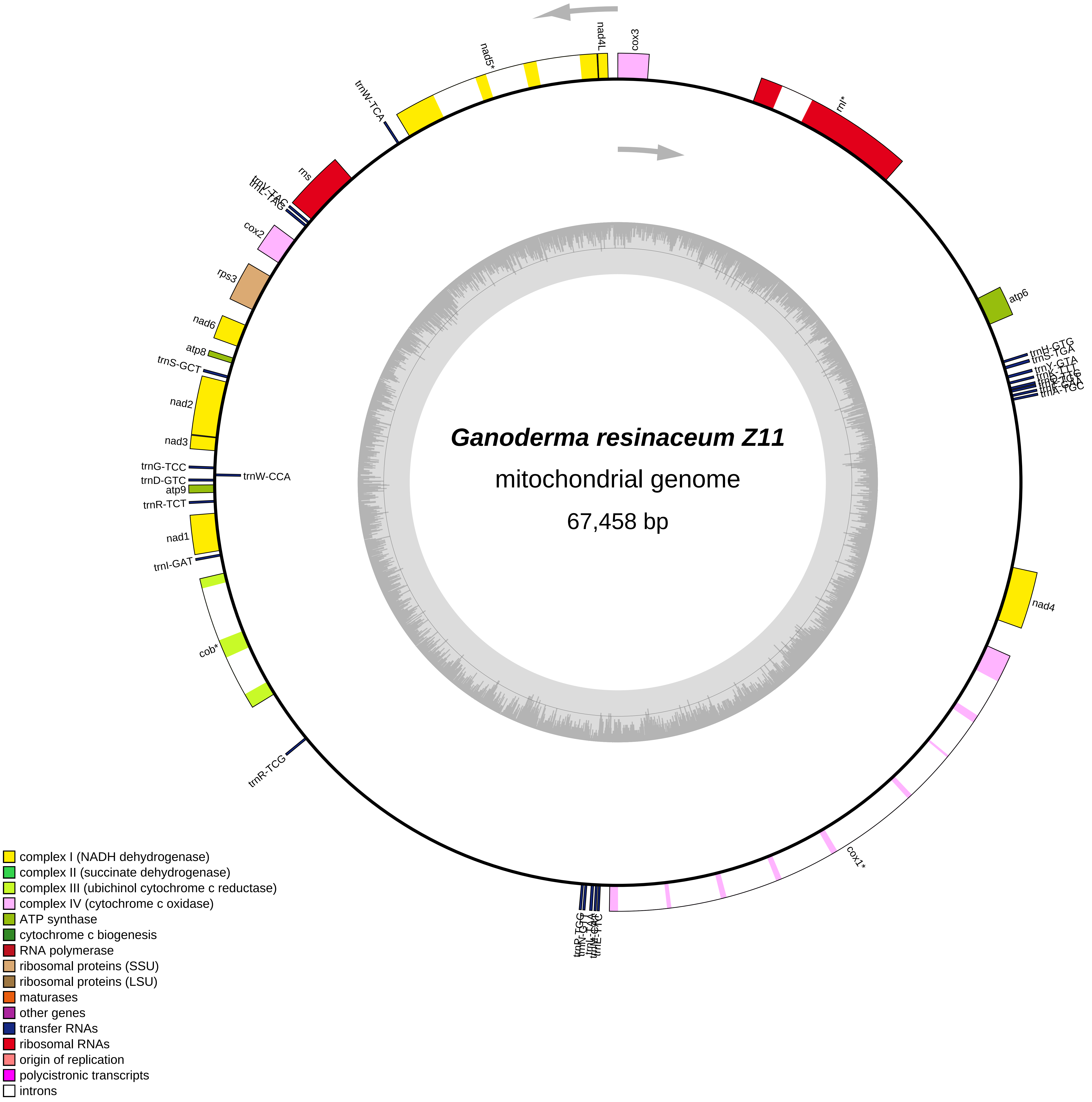
Ganoderma resinaceum Z11
mitochondrial genome
67,458 bp
cox3
nad4L
nad5*
trnW-TCA
rns
cox2
rps3
nad6
atp8
trnS-GCT
nad2
nad3
trnG-TCC
trnW-CCA
trnD-GTC
atp9
trnR-TCT
nad1
trnI-GAT
cob*
trnR-TCG
cox1*
nad4
trnH-GTG
trnS-TGA
trnY-GTA
trnA-TGC
atp6
rnl*
complex I (NADH dehydrogenase)
complex II (succinate dehydrogenase)
complex III (ubichinol cytochrome c reductase)
complex IV (cytochrome c oxidase)
ATP synthase
cytochrome c biogenesis
RNA polymerase
ribosomal proteins (SSU)
ribosomal proteins (LSU)
maturases
other genes
transfer RNAs
ribosomal RNAs
origin of replication
polycistronic transcripts
introns

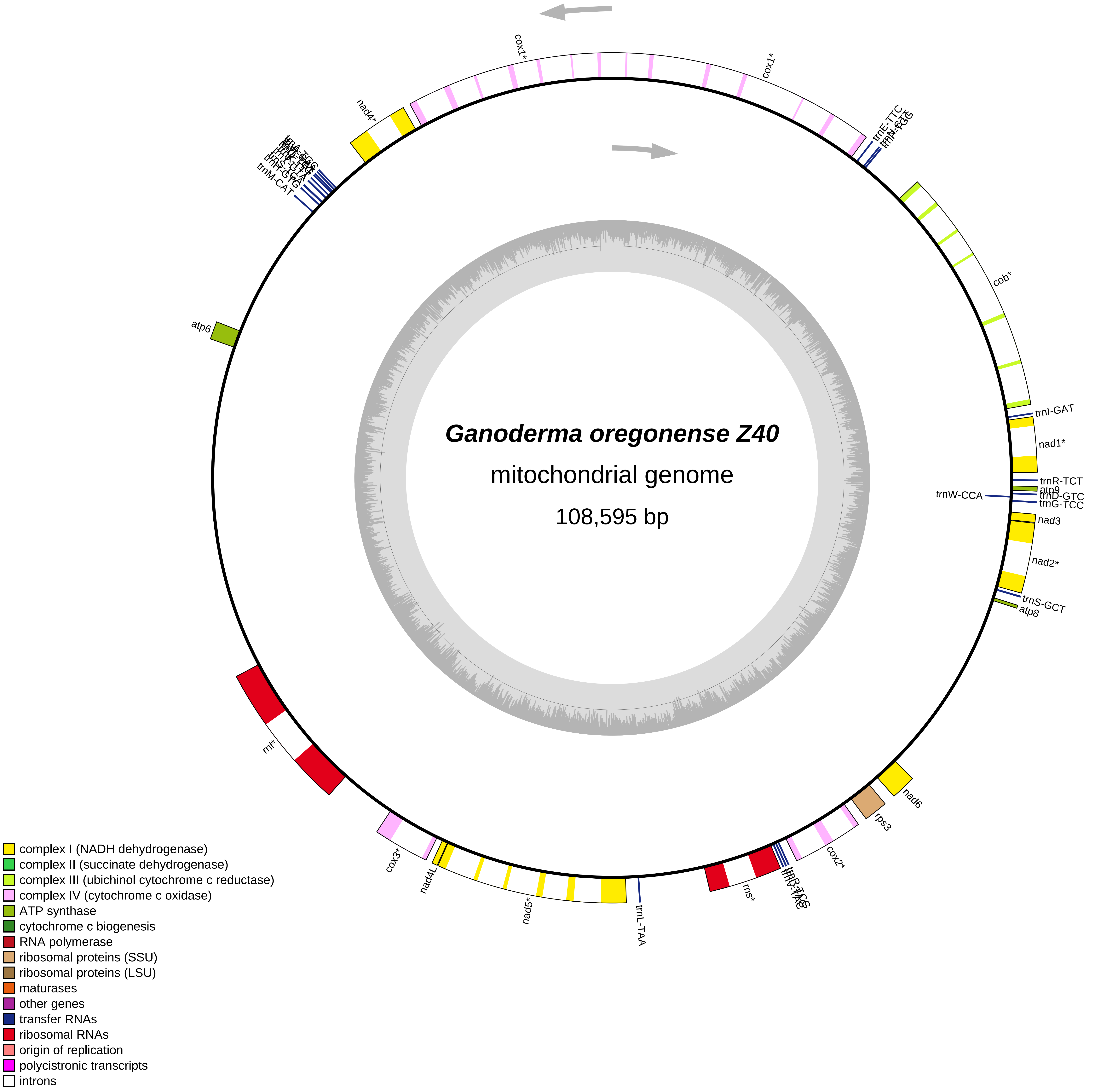
Ganoderma oregonense Z40
mitochondrial genome
108,595 bp
cox1*
cox1*
trnE-TTC
nad4*
trnM-CAT
atp6
cob*
trnI-GAT
nad1*
trnR-TCT
atp9
trnW-CCA
trnG-TCC
nad3
nad2*
trnS-GCT
atp8
nad6
rps3
cox2*
rns*
trnL-TAA
nad5*
nad4L
cox3*
rnl*
complex I (NADH dehydrogenase)
complex II (succinate dehydrogenase)
complex III (ubichinol cytochrome c reductase)
complex IV (cytochrome c oxidase)
ATP synthase
cytochrome c biogenesis
RNA polymerase
ribosomal proteins (SSU)
ribosomal proteins (LSU)
maturases
other genes
transfer RNAs
ribosomal RNAs
origin of replication
polycistronic transcripts
introns

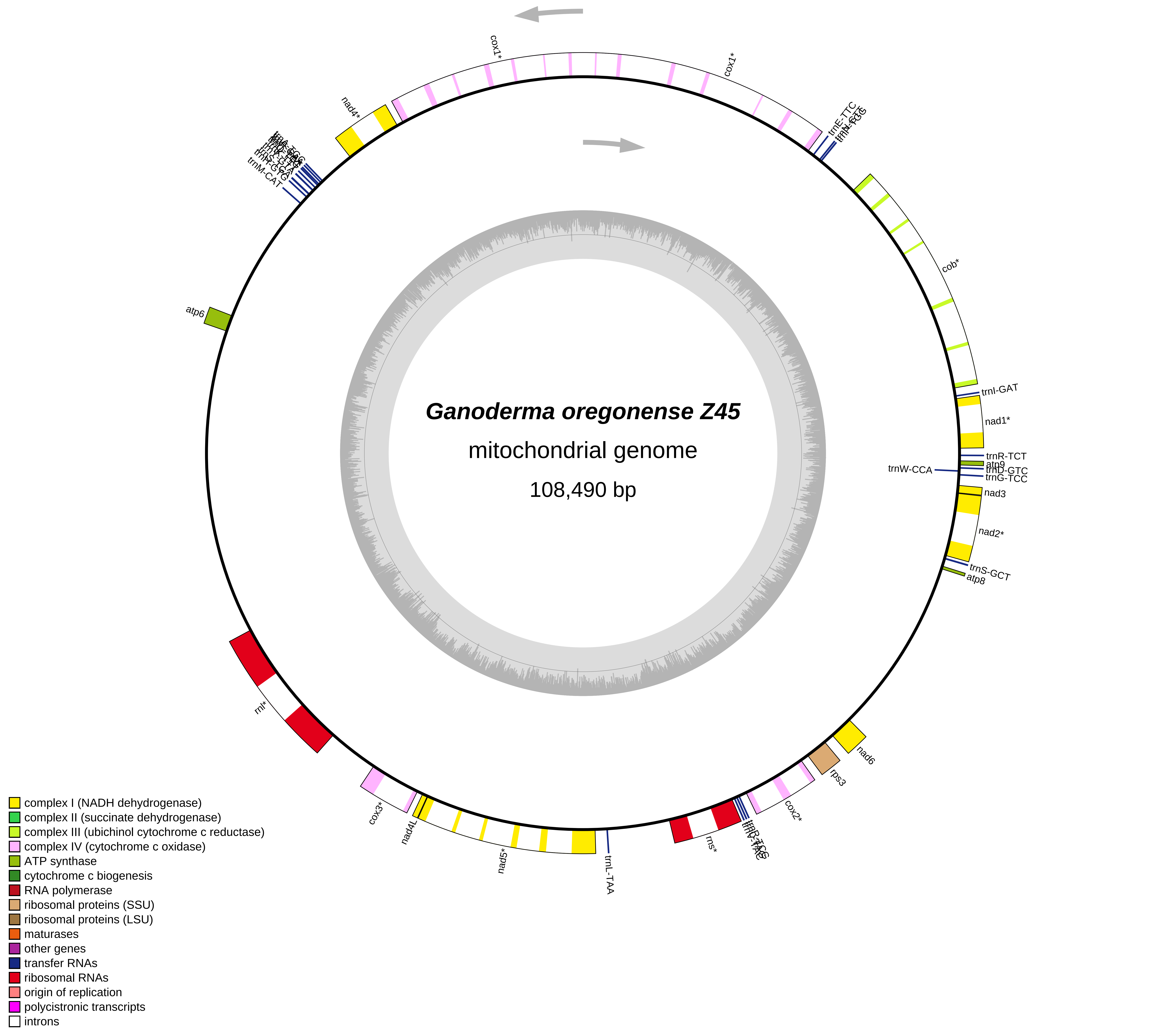

Ganoderma oregonense Z45
mitochondrial genome
108,490 bp
cox1*
cox1*
nad4*
trnE-TTC
cob*
trnI-GAT
nad1*
trnR-TCT
atp9
trnD-GTC
trnG-TCC
trnW-CCA
nad3
nad2*
trnS-GCT
atp8
nad6
rps3
cox2*
rns*
trnL-TAA
nad5*
nad4L
cox3*
rnl*
atp6
trnM-CAT
complex I (NADH dehydrogenase)
complex II (succinate dehydrogenase)
complex III (ubichinol cytochrome c reductase)
complex IV (cytochrome c oxidase)
ATP synthase
cytochrome c biogenesis
RNA polymerase
ribosomal proteins (SSU)
ribosomal proteins (LSU)
maturases
other genes
transfer RNAs
ribosomal RNAs
origin of replication
polycistronic transcripts
introns

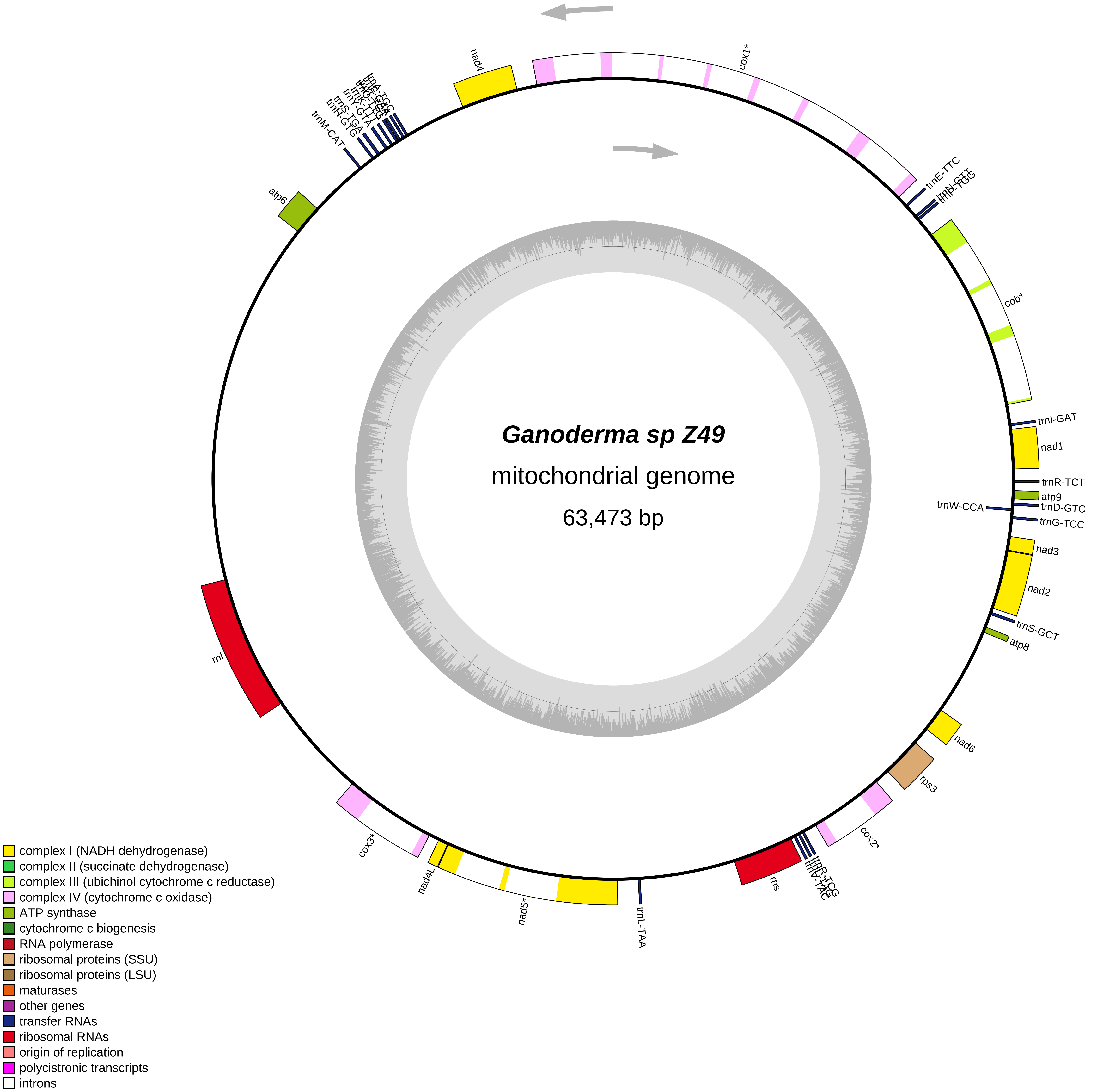
Ganoderma sp Z49
mitochondrial genome
63,473 bp
cox1*
nad4
trnM-CAT
atp6
trnE-TTC
cob*
trnI-GAT
nad1
trnR-TCT
atp9
trnW-CCA
trnD-GTC
trnG-TCC
nad3
nad2
trnS-GCT
atp8
nad6
rps3
cox2*
rns
trnL-TAA
nad5*
nad4L
cox3*
rnl
complex I (NADH dehydrogenase)
complex II (succinate dehydrogenase)
complex III (ubichinol cytochrome c reductase)
complex IV (cytochrome c oxidase)
ATP synthase
cytochrome c biogenesis
RNA polymerase
ribosomal proteins (SSU)
ribosomal proteins (LSU)
maturases
other genes
transfer RNAs
ribosomal RNAs
origin of replication
polycistronic transcripts
introns

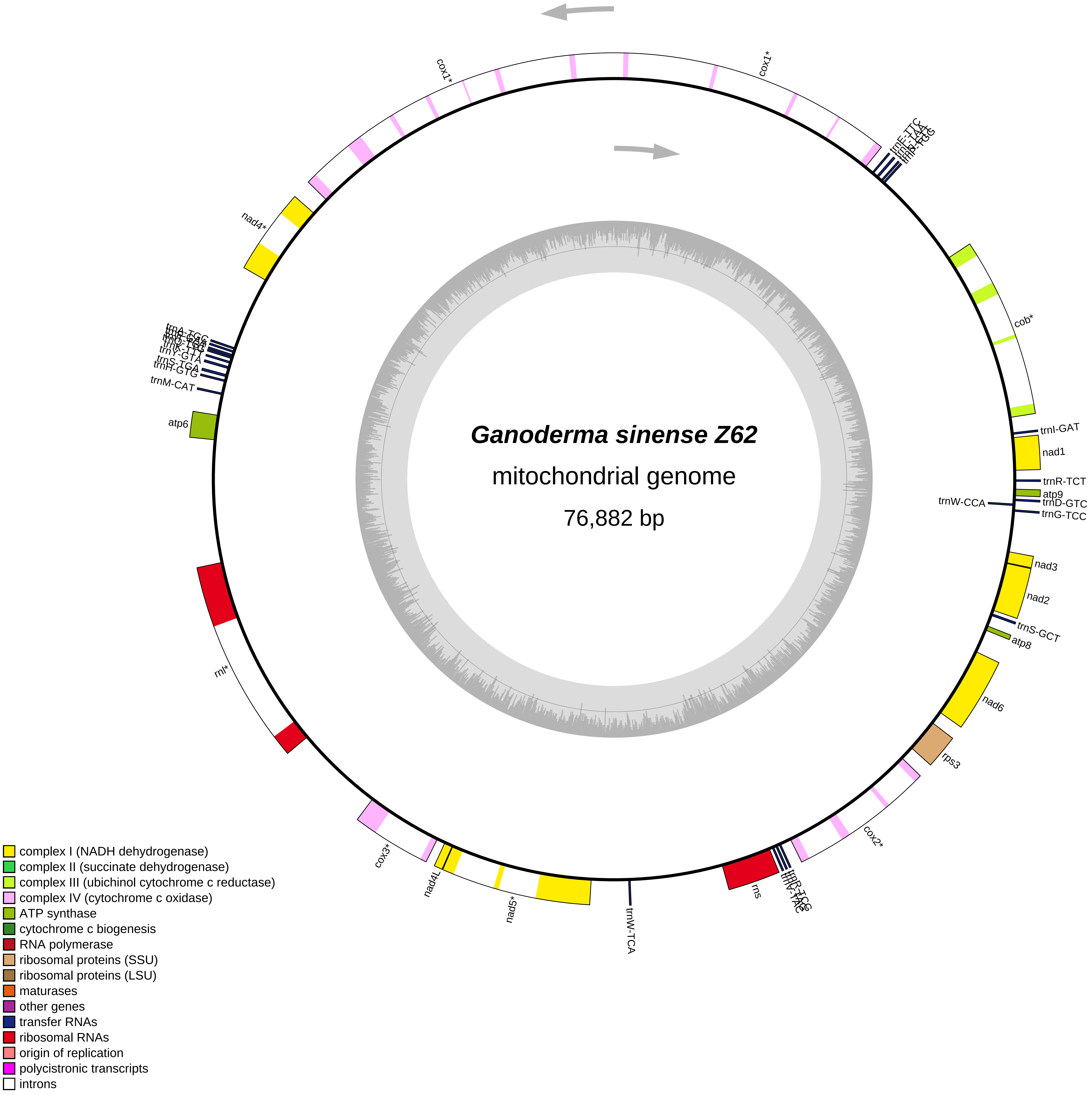

Ganoderma sinense Z62
mitochondrial genome
76,882 bp
cox1*
cox1*
trnF-TTC
nad4*
trnA-TGC
trnY-GTA
trnS-TGA
trnH-GTG
trnM-CAT
atp6
cob*
trnI-GAT
nad1
trnR-TCT
atp9
trnW-CCA
trnD-GTC
trnG-TCC
nad3
nad2
trnS-GCT
atp8
nad6
rps3
cox2*
rns
rnl*
cox3*
nad4L
nad5*
trnW-TCA
complex I (NADH dehydrogenase)
complex II (succinate dehydrogenase)
complex III (ubichinol cytochrome c reductase)
complex IV (cytochrome c oxidase)
ATP synthase
cytochrome c biogenesis
RNA polymerase
ribosomal proteins (SSU)
ribosomal proteins (LSU)
maturases
other genes
transfer RNAs
ribosomal RNAs
origin of replication
polycistronic transcripts
introns
