# Supplementary material for: Insights into intraspecific variation and genotyping of Ganoderma lingzhi through pan-mitogenome analysis
Source: IMA Fungus. 2026 Jun 3;17:e184941. doi: 10.3897/imafungus.17.184941 (PMC13254553; doi:10.3897/imafungus.17.184941)
Supplement: Supplementary material 4 — Supplementary image 3 [file imafungus-17-e184941-s004.pdf]

Figure S3 Lineage-specific intron gain/loss events. The phylogenetic tree used here is consistent with that shown in Figure 7. Lineage-specific intron gain/loss events are indicated at the three basal nodes. Including the introns *cox2P228*, *nad5P426*, and *cox1P1305* shared by all 157 strains; the introns *cox1P731*, *cox1P894*, and *cox1P1057* specific to Group 2; and the lineage-specific introns *cox1P276*, *cox1P709*, and *cox1P867* found in Group 1.

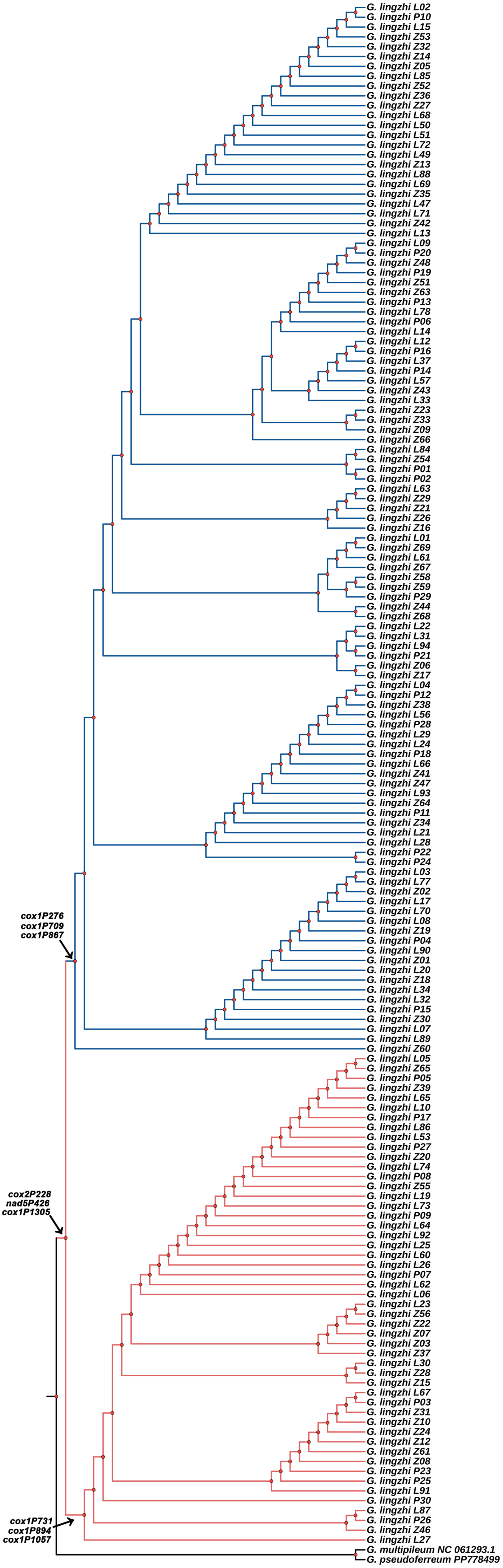
G. lingzhi L02
G. lingzhi P10
G. lingzhi L15
G. lingzhi Z53
G. lingzhi Z32
G. lingzhi Z14
G. lingzhi Z05
G. lingzhi L85
G. lingzhi Z52
G. lingzhi Z36
G. lingzhi Z27
G. lingzhi L68
G. lingzhi L50
G. lingzhi L51
G. lingzhi L72
G. lingzhi L49
G. lingzhi Z13
G. lingzhi L88
G. lingzhi L69
G. lingzhi Z35
G. lingzhi L47
G. lingzhi L71
G. lingzhi Z42
G. lingzhi L13
G. lingzhi L09
G. lingzhi P20
G. lingzhi Z48
G. lingzhi P19
G. lingzhi Z51
G. lingzhi Z63
G. lingzhi P13
G. lingzhi L78
G. lingzhi P06
G. lingzhi L14
G. lingzhi L12
G. lingzhi P16
G. lingzhi L37
G. lingzhi P14
G. lingzhi L57
G. lingzhi Z43
G. lingzhi L33
G. lingzhi Z23
G. lingzhi Z33
G. lingzhi Z09
G. lingzhi Z66
G. lingzhi L84
G. lingzhi Z54
G. lingzhi P01
G. lingzhi P02
G. lingzhi L63
G. lingzhi Z29
G. lingzhi Z21
G. lingzhi Z26
G. lingzhi Z16
G. lingzhi L01
G. lingzhi Z69
G. lingzhi L61
G. lingzhi Z67
G. lingzhi Z58
G. lingzhi Z59
G. lingzhi P29
G. lingzhi Z44
G. lingzhi Z68
G. lingzhi L22
G. lingzhi L31
G. lingzhi L94
G. lingzhi P21
G. lingzhi Z06
G. lingzhi Z17
G. lingzhi L04
G. lingzhi P12
G. lingzhi Z38
G. lingzhi L56
G. lingzhi P28
G. lingzhi L29
G. lingzhi L24
G. lingzhi P18
G. lingzhi L66
G. lingzhi Z41
G. lingzhi Z47
G. lingzhi L93
G. lingzhi Z64
G. lingzhi P11
G. lingzhi Z34
G. lingzhi L21
G. lingzhi L28
G. lingzhi P22
G. lingzhi P24
cox1P276
cox1P709
cox1P867
G. lingzhi L03
G. lingzhi L77
G. lingzhi Z02
G. lingzhi L17
G. lingzhi L70
G. lingzhi L08
G. lingzhi Z19
G. lingzhi P04
G. lingzhi L90
G. lingzhi Z01
G. lingzhi L20
G. lingzhi Z18
G. lingzhi L34
G. lingzhi L32
G. lingzhi P15
G. lingzhi Z30
G. lingzhi L07
G. lingzhi L89
G. lingzhi Z60
G. lingzhi L05
G. lingzhi Z65
G. lingzhi P05
G. lingzhi Z39
G. lingzhi L65
G. lingzhi L10
G. lingzhi P17
G. lingzhi L86
G. lingzhi L53
G. lingzhi P27
G. lingzhi Z20
G. lingzhi L74
G. lingzhi P08
G. lingzhi Z55
G. lingzhi L19
G. lingzhi L73
G. lingzhi P09
cox2P228
nad5P426
cox1P1305
G. lingzhi L64
G. lingzhi L92
G. lingzhi L25
G. lingzhi L60
G. lingzhi L26
G. lingzhi P07
G. lingzhi L62
G. lingzhi L06
G. lingzhi L23
G. lingzhi Z56
G. lingzhi Z22
G. lingzhi Z07
G. lingzhi Z03
G. lingzhi Z37
G. lingzhi L30
G. lingzhi Z28
G. lingzhi Z15
G. lingzhi L67
G. lingzhi P03
G. lingzhi Z31
G. lingzhi Z10
G. lingzhi Z24
G. lingzhi Z12
G. lingzhi Z61
G. lingzhi Z08
G. lingzhi P23
G. lingzhi P25
G. lingzhi L91
G. lingzhi P30
G. lingzhi L87
G. lingzhi P26
G. lingzhi Z46
G. lingzhi L27
cox1P731
cox1P894
cox1P1057
G. multipileum NC 061293.1
G. pseudoferreum PP778499
